# Supplementary material for: Double boron–oxygen-fused polycyclic aromatic hydrocarbons: skeletal editing and applications as organic optoelectronic materials
Source: Nat Commun. 2023 Nov 4;14:7089. doi: 10.1038/s41467-023-42973-1 (PMC10625603; doi:10.1038/s41467-023-42973-1)
Supplement: Supplementary file 1 — Supplementary Information [file 41467_2023_42973_MOESM1_ESM.pdf]

Supplementary Information

Double Boron–Oxygen-Fused Polycyclic Aromatic Hydrocarbons: Skeletal Editing and Applications as Organic Optoelectronic Materials

Guijie Li[1]*, Kewei Xu[1], Jianbing Zheng[1], Xiaoli Fang[1], Yun-Fang Yang[1], Weiwei Lou[1], Qingshan Chu[1], Jianxin Dai[1], Qidong Chen[1], Yuning Yang[1], Yuanbin She[1]*

[1]College of Chemical Engineering, State Key Laboratory Breeding Base of Green-Chemical Synthesis Technology, Zhejiang University of Technology, Hangzhou, Zhejiang 310014, P. R. China

E-mail: guijieli@zjut.edu.cn; sheyb@zjut.edu.cn

Table of Contents

1	**Supplementary Methods**	S8−S10
2	**Supplementary Fig. 1. Theoretical calculation, chemical structures and photophysical properties of Pyrene, BN1, BO1b and DABNA-1**. Comparison of calculated frontier orbital distributions, energy levels/gaps, calculated and experimental E_{T1}, MO character, aromaticity.	S11
3	**Supplementary Fig. 2. Reaction condition optimization.** Template reactions and selected optimization of reaction conditions.	S12
4	**Supplementary Discussion**<br>Reaction Condition Optimization	S13
5	Synthesis of dBO-PAHs	S14−S43
6	**Supplementary Fig. 3. $AlCl_3$ induced de-*tert*-butylation reactions.** The observed de-*tert*-butylation reactions catalyzed by $AlCl_3$.	S43
7	**Supplementary Fig. 4.** ORTEP drawing of X-ray single crystal diffraction structures of **BO1a**, **BO2**, **BO3a**, **BO3b-OH**, **BO3d**, **BO4b**, **BO4d**, **BO4e**, **BO5** and **BO6**.	S44
8	**Supplementary Fig. 5.** Spacefill drawing of X-ray single crystal diffraction structures of **BO1a**, **BO2**, **BO3a**, **BO3d**, **BO4b**, **BO4d**, **BO4e** and **BO6**.	S45
9	**Supplementary Table 1. Crystal data and structure refinements of BO1a and BO2.**	S46
10	**Supplementary Table 2. Crystal data and structure refinements of BO3a, BO3d and BO3b-OH.**	S47
11	**Supplementary Table 3. Crystal data and structure refinements for BO4b, BO4d and BO4e·CH_2Cl_2.**	S48
12	**Supplementary Table 4. Crystal data and structure refinements of BO5 and BO6.**	S49
13	**Supplementary Fig. 6: Single crystal structure.** X-ray single crystal diffraction structures and crystal packings of **BO1a**. **a** Front View. **b** Top View. **c** Side View. **d** Solide-State Packing Structure.	S50
14	**Supplementary Fig. 7: Single crystal structure.** X-ray single crystal diffraction structures and crystal packings of **BO2**. **a** Front View. **b** Top View. **c** Side View. **d** Solide-State Packing Structure.	S51
15	**Supplementary Fig. 8: Single crystal structure.** X-ray single crystal diffraction structures and crystal packings of **BO3a**. **a** Front View. **b** Top View. **c** Side View. **d** Solide-State Packing Structure.	S52
16	**Supplementary Fig. 9: Single crystal structure.** X-ray single crystal diffraction structures and crystal packings of **BO3d**. **a** Front View. **b** Top View. **c** Side View. **d** Solide-State Packing Structure.	S53
17	**Supplementary Fig. 10: Single crystal structure.** X-ray single crystal diffraction structures and crystal packings of **BO4b**. **a** Front View. **b** Top View. **c** Side View. **d** Solide-State Packing Structure.	S54
18	**Supplementary Fig. 11: Single crystal structure.** X-ray single crystal diffraction structures and crystal packings of **BO4d**. **a** Front View. **b** Top View. **c** Side View. **d** Solide-State Packing Structure.	S55
19	**Supplementary Fig. 12: Single crystal structure.** X-ray single crystal diffraction structures and crystal packings of **BO4e**. **a** Front View. **b** Top View. **c** Side View. **d**	S56

	Solide-State Packing Structure.	
20	**Supplementary Fig. 13: Single crystal structure.** X-ray single crystal diffraction structures and crystal packings of **BO5**. **a** Front View. **b** Top View. **c** Side View. **d** Solide-State Packing Structure.	S57
21	**Supplementary Fig. 14: Single crystal structure.** X-ray single crystal diffraction structures and crystal packings of **BO6**. **a** Front View. **b** Top View. **c** Side View. **d** Solide-State Packing Structure.	S58
22	**Supplementary Fig. 15. NICS(1) values of dBO-PAHs.** Comparison of the calculated NICS(1) values of dBO-PAHs **BO1a**, **BO2**, **BO3a**, **BO4b**, **BO5**, **BO6** and their corresponding PAH analogues **CC1a**, **CC2**, CC**3a**, **CC4b**, **CC5**, **CC6**.	S59
23	**Supplementary Table 5. Comparison of calculated HOMO and LUMO levels and energy gaps of the dBO-PAHs and their corresponding carbon analogues.**	S60
24	**Supplementary Fig. 16. Theoretical calculation.** Comparison of frontier orbital distributions, energy levels, and NICS(1) values of dBO-PAHs **BO1a**, **BO1b**, **BO1c**, **BO1d**, **BO1e** and **BO1g**, as well as their correspounding carbon-based PAHs **CC1a**, **CC1b**, **CC1c**, **CC1d**, **CC1e** and **CC1g**. The calculations were performed at B3LYP/6-31G(d) level.	S61
25	**Supplementary Fig. 17. Theoretical calculation.** Comparison of frontier orbital distributions, energy levels, and NICS(1) values of dBO-PAHs **BO1a**, **BO2**, **BO3a**, **BO4a**, **BO5** and **BO6**, as well as their correspounding carbon-based PAHs **CC1a**, **CC2**, **CC3a**, **CC4a**, **CC5** and **CC6**. The calculations were performed at B3LYP/6-31G(d) level.	S62
26	**Supplementary Fig. 18. Theoretical calculation.** Comparison of frontier orbital distributions, energy levels, and NICS(1) values of dBO-PAHs **BO3a**, **BO3b**, **BO3c**, **BO3d** and **BO3e**, as well as their correspounding carbon-based PAHs **CC3a**, **CC3b**, **CC3c**, **CC3d** and **CC3e**. The calculations were performed at B3LYP/6-31G(d) level.	S63
27	**Supplementary Fig. 19. Theoretical calculation.** Comparison of frontier orbital distributions, energy levels, and NICS(1) values of dBO-PAHs **BO4a**, **BO4b**, **BO4c**, **BO4d** and **BO4e**, as well as their correspounding carbon-based PAHs **CC5a**, **CC5b**, **CC5c**, **CC5d** and **CC4e**. The calculations were performed at B3LYP/6-31G(d) level.	S64
28	**Supplementary Fig. 20. Photophysical properties.** Comparison of room-temperature absorption spectra for **a**, **BO1a**, **BO2**, **BO3a**, **BO4a**, **BO5** and **BO6**; **b**, **BO1** series; **c**, **BO3** series; and **d**, **BO4** series in dichloromethane solution.	S65
29	**Supplementary Fig. 21. Photophysical properties.** Comparison of room-temperature (RT) PL spectra for **a**, **BO1a**, **BO2**, **BO3a**, **BO4a**, **BO5** and **BO6**; **b**, **BO1** series; **c**, **BO3** series; and **d**, **BO4** series in dichloromethane solution.	S66
30	**Supplementary Fig. 22. Photophysical properties.** Comparison of low-temperature (77 K) phosphorescent spectra for **a**, **BO1a**, **BO2**, **BO3a**, **BO4a**, **BO5** and **BO6**; **b**, **BO1** series; **c**, **BO3** series; and **d**, **BO4** series in 2-MeTHF.	S67
31	**Supplementary Fig. 23. Photophysical properties.** Room-temperature absorption spectrum (red solid lines) and flluorescent spectrum (blue solid line) in dichloromethane, and low-temperature (77 K) flluorescent and phosphorescent spectra (blue dash-dotted and navy dotted lines) in 2-MeTHF of **BO1a**. The chemical structure is shown in the inset.	S68
32	**Supplementary Fig. 24. Photophysical properties.** Room-temperature absorption spectrum (red solid lines) and flluorescent spectrum (blue solid line) in dichloromethane,	S68

	and low-temperature (77 K) flluorescent and phosphorescent spectra (blue dash-dotted and navy dotted lines) in 2-MeTHF of **BO1b**. The chemical structure is shown in the inset.	
33	**Supplementary Fig. 25. Photophysical properties.** Room-temperature absorption spectrum (red solid lines) and flluorescent spectrum (blue solid line) in dichloromethane, and low-temperature (77 K) flluorescent and phosphorescent spectra (blue dash-dotted and navy dotted lines) in 2-MeTHF of **BO1c**. The chemical structure is shown in the inset.	S69
34	**Supplementary Fig. 26. Photophysical properties.** Room-temperature absorption spectrum (red solid lines) and flluorescent spectrum (blue solid line) in dichloromethane, and low-temperature (77 K) flluorescent and phosphorescent spectra (blue dash-dotted and navy dotted lines) in 2-MeTHF and their corresponding excited spectra of **BO1d**. The chemical structure is shown in the inset.	S69
35	**Supplementary Fig. 27. Photophysical properties.** Room-temperature absorption spectrum (red solid lines) and flluorescent spectrum (blue solid line) in dichloromethane, and low-temperature (77 K) flluorescent and phosphorescent spectra (blue dash-dotted and navy dotted lines) in 2-MeTHF of **BO1e**. The chemical structure is shown in the inset.	S70
36	**Supplementary Fig. 28. Photophysical properties.** Room-temperature absorption spectrum (red solid lines) and flluorescent spectrum (blue solid line) in dichloromethane, and low-temperature (77 K) flluorescent and phosphorescent spectra (blue dash-dotted and navy dotted lines) in 2-MeTHF of **BO1f**. The chemical structure is shown in the inset.	S70
37	**Supplementary Fig. 29. Photophysical properties.** Room-temperature absorption spectrum (red solid lines) and flluorescent spectrum (blue solid line) in dichloromethane, and low-temperature (77 K) flluorescent and phosphorescent spectra (blue dash-dotted and navy dotted lines) in 2-MeTHF of **BO1g**. The chemical structure is shown in the inset.	S71
38	**Supplementary Fig. 30. Photophysical properties.** Room-temperature absorption spectrum (red solid lines) and flluorescent spectrum (blue solid line) in dichloromethane, and low-temperature (77 K) flluorescent and phosphorescent spectra (blue dash-dotted and navy dotted lines) in 2-MeTHF of **BO2**. The chemical structure is shown in the inset.	S71
39	**Supplementary Fig. 31. Photophysical properties.** Room-temperature absorption spectrum (red solid lines) and flluorescent spectrum (blue solid line) in dichloromethane, and low-temperature (77 K) flluorescent and phosphorescent spectra (blue dash-dotted and navy dotted lines) in 2-MeTHF of **BO3a**. The chemical structure is shown in the inset.	S72
40	**Supplementary Fig. 32. Photophysical properties.** Room-temperature absorption spectrum (red solid lines) and flluorescent spectrum (blue solid line) in dichloromethane, and low-temperature (77 K) flluorescent and phosphorescent spectra (blue dash-dotted and navy dotted lines) in 2-MeTHF of **BO3b**. The chemical structure is shown in the inset.	S72
41	**Supplementary Fig. 33. Photophysical properties.** Room-temperature absorption spectrum (red solid lines) and flluorescent spectrum (blue solid line) in dichloromethane, and low-temperature (77 K) flluorescent and phosphorescent spectra (blue dash-dotted and navy dotted lines) in 2-MeTHF of **BO3c**. The chemical structure is shown in the inset.	S73
42	**Supplementary Fig. 34. Photophysical properties.** Room-temperature absorption spectrum (red solid lines) and flluorescent spectrum (blue solid line) in dichloromethane, and low-temperature (77 K) flluorescent and phosphorescent spectra (blue dash-dotted and navy dotted lines) in 2-MeTHF of **BO3d**. The chemical structure is shown in the inset.	S73

43	**Supplementary Fig. 35. Photophysical properties.** Room-temperature absorption spectrum (red solid lines) and flluorescent spectrum (blue solid line) in dichloromethane, and low-temperature (77 K) flluorescent and phosphorescent spectra (blue dash-dotted and navy dotted lines) in 2-MeTHF of **BO3e**. The chemical structure is shown in the inset.	S74
44	**Supplementary Fig. 36. Photophysical properties.** Room-temperature absorption spectrum (red solid lines) and flluorescent spectrum (blue solid line) in dichloromethane, and low-temperature (77 K) flluorescent and phosphorescent spectra (blue dash-dotted and navy dotted lines) in 2-MeTHF of **BO4a**. The chemical structure is shown in the inset.	S74
45	**Supplementary Fig. 37. Photophysical properties.** Room-temperature absorption spectrum (red solid lines) and flluorescent spectrum (blue solid line) in dichloromethane, and low-temperature (77 K) flluorescent and phosphorescent spectra (blue dash-dotted and navy dotted lines) in 2-MeTHF of **BO4b**. The chemical structure is shown in the inset.	S75
46	**Supplementary Fig. 38. Photophysical properties.** Room-temperature absorption spectrum (red solid line) and luminescent spectrum in dichloromethane (blue solid line), and low-temperature (77 K) phosphorescent spectrum (blue dash-dotted line) in 2-MeTHF of **BO4c**. The chemical structure is shown in the inset.	S75
47	**Supplementary Fig. 39. Photophysical properties.** Room-temperature absorption spectrum (red solid line) and luminescent spectrum in dichloromethane (blue solid line), and low-temperature (77 K) phosphorescent spectrum (blue dash-dotted line) in 2-MeTHF of **BO4d**. The chemical structure is shown in the inset.	S76
48	**Supplementary Fig. 40. Photophysical properties.** Room-temperature absorption spectrum (red solid lines) and flluorescent spectrum (blue solid line) in dichloromethane, and low-temperature (77 K) flluorescent and phosphorescent spectra (blue dash-dotted and navy dotted lines) in 2-MeTHF of **BO4e**. The chemical structure is shown in the inset.	S76
49	**Supplementary Fig. 41. Photophysical properties.** Room-temperature absorption spectrum (red solid lines) and flluorescent spectrum (blue solid line) in dichloromethane, and low-temperature (77 K) flluorescent and phosphorescent spectra (blue dash-dotted and navy dotted lines) in 2-MeTHF of **BO5**. The chemical structure is shown in the inset.	S77
50	**Supplementary Fig. 42. Photophysical properties.** Room-temperature absorption spectrum (red solid lines) and flluorescent spectrum (blue solid line) in dichloromethane, and low-temperature (77 K) flluorescent and phosphorescent spectra (blue dash-dotted and navy dotted lines) in 2-MeTHF of **BO6**. The chemical structure is shown in the inset.	S77
51	**Supplementary Fig. 43. Photophysical properties.** Selected PL decay of **a**, **BO1c**, **BO1f**; **b**, **BO2**; **c**, **BO3b**, **BO3c**, **BO3d**, **BO3e**; and **d**, **BO4b**, **BO4c**, **BO4d**, **BO4e** in 2-MeTHF at 77 K excited at their corresponding first fluorescent peak.	S78
52	**Supplementary Fig. 44. Photophysical properties.** Selected PL decay of **a**, **BO1b**, **BO1c**, **BO1d**, **BO1f**; **b**, **BO3a**, **BO3b**, **BO3c**, **BO3d**, **BO3e**; **c**, **BO4a**, **BO4b**, **BO4c**, **BO4d**, **BO4e**; and **d**, **BO2**, **BO5**, **BO6** in 2-MeTHF at 77 K excited at their corresponding first phosphorescent peak.	S79
53	**Supplementary Fig. 45. Ultralong phosphorescence.** Ultralong phosphorescent photographs of **BO1a**, **BO1b**, **BO1d**, **BO1e**, **BO2**, **BO3b** and **BO3c** at 77K in THF.	S80
54	**Supplementary Fig. 46. Ultralong phosphorescence.** Ultralong phosphorescent photographs of **BO4a**, **BO4c**, **BO4d**, **BO4e**, **BO5** and **BO6** at 77K in THF.	S81
55	**Supplementary Fig. 47. Theoretical calculation.** TD-DFT calculated singlet and triplet	S82

	energy levels, main transition configurations, spin–orbit coupling (SOC) values and natural transition orbital (NTO) analyses of **a**, **BO1a**; **b**, **BO1d**; **c**, **BO6** at B3LYP/6-31G(d) level based on optimized S_0 geometry. Selected frontier orbital distributions and energy levels are also illustrated.	
56	**Supplementary Fig. 48. Theoretical calculation.** TD-DFT calculated singlet and triplet energy levels, main transition configurations of **BO1b**, **BO1c** and **BO1e** at B3LYP/6-31G(d) level based on optimized S_0 geometry. Selected frontier orbital distributions and energy levels are also illustrated.	S83
57	**Supplementary Fig. 49. Theoretical calculation.** TD-DFT calculated singlet and triplet energy levels, main transition configurations of **BO2**, **BO3a**, **B3d** and **BO3e** at B3LYP/6-31G(d) level based on optimized S_0 geometry. Selected frontier orbital distributions and energy levels are also illustrated.	S84
58	**Supplementary Fig. 50. Theoretical calculation.** TD-DFT calculated singlet and triplet energy levels, main transition configurations of **BO4a**, **BO4b** and **BO4c** at B3LYP/6-31G(d) level based on optimized S_0 geometry. Selected frontier orbital distributions and energy levels are also illustrated.	S85
59	**Supplementary Fig. 51. Theoretical calculation.** TD-DFT calculated singlet and triplet energy levels, main transition configurations of **BO4d**, **BO4e** and **BO5** at B3LYP/6-31G(d) level based on optimized S_0 geometry. Selected frontier orbital distributions and energy levels are also illustrated.	S86
60	**Supplementary Fig. 52. Thermal properties. a**, TGA; and **b**, DSC curves of **BO1b**, **BO2**, **BO4a**, **BO5** and **BO6**. The strongly endothermic peaks at 276 °C for **BO1b**, 287 °C for **BO2**, 385 °C for **BO4a**, 323 °C for **BO5**, and 273 °C for **BO6** could be attributed to their melting processes, in good agreement with their corresponding melting temperatures (T_m) of 276.2–277.9, 287.1–288.2, >350, 320.1–322.5 and 271.8–272.9 °C, respectively.	S87
61	**Supplementary Fig. 53. Electrochemical properties.** Cyclic voltammogram (CV) of **BO1b** in anhydrous *N*,*N*-dimethylformamide (DMF).	S88
62	**Supplementary Table 6. Electrochemical properties and energy levels of selected dBO-PAHs.**	S88
63	**Supplementary Fig. 54. Single carrier devices. a** Current density−voltage (*J*–*V*) characteristics of hole-only device with a structure of ITO/TAPC (10 nm)/dBO-PAH (60 nm)/TAPC (10 nm)/Al. **b** electron-only device with a structure of ITO/TmPyPB (10 nm)/ dBO-PAH (60 nm)/TmPyPB (10 nm)/LiF (1 nm)/Al.	S89
64	**Supplementary Fig. 55. Device and material structures.** Energy level diagram of deep-blue OLEDs and chemical structures of materials used.	S90
65	**Supplementary Fig. 56. PL and EL spectral comparison.** The PL of PtON1-doped thin films versus the EL of PtON1-doped devices. The Ref. is the device with a structure of ITO/HATCN/NPD/TAPC/6%PtON1:26mCPy/DPPS/LiF/Al.	S90
66	**Supplementary Fig. 57. EL properties of deep-blue OLEDs. a, b** Fitted EQE *vs.* luminance plots.	S90
67	**Supplementary Fig. 58. Low-temperature spectrum of PtON-TBBI.**	S91
68	**Supplementary Fig. 59. Deep-blue Pt(II) emitters.** Chemical structures of dopants for Pt(II)-based deep-blue OLEDs discussed in this work.	S93

69	**Supplementary Table 7. Performance data for Pt(II)-based deep-blue OLEDs with CIE_y < 0.20.**	S94
70	**Supplementary Fig. 60. Deep-blue Ir(II) emitters.** Chemical structures of dopants for Ir(III)-based deep-blue OLEDs discussed in this work.	S95
71	**Supplementary Table 8. Performance data for Ir(III)-based deep-blue OLEDs with CIE_y < 0.20.**	S96−S97
72	**Supplementary Fig. 61. Electrochemical properties.** Cyclic voltammogram (CV) of **a**, **BO1c**; **b**, **BO1g**; **c**, **BO2**; **e**, **BO3a**; **f**, **BO6**; **d**, differential pulse voltammetry (DPV) of **BO2** in anhydrous *N*,*N*-dimethylformamide (DMF).	S98
73	**Supplementary Fig. 62. Deep-blue MR-BN-PAH emitters.** Chemical structures of dopants for MR-BN-PAH-based deep-blue OLEDs discussed in this work.	S99
74	**Supplementary Fig. 63. Deep-blue MR-BN-PAH emitters.** Chemical structures of dopants for MR-BN-PAH-based deep-blue OLEDs discussed in this work.	S100
75	**Supplementary Table 9. Performance data for MR-BN-PAH-based deep-blue OLEDs with CIE_y < 0.20.**	S101−S102
76	**Supplementary Fig. 64. Operational lifetimes of the deep-blue OLEDs** (L_0 = 500 cd/cm^2)	S103
77	^{1}H NMR, ^{13}C NMR, ^{11}B NMR and HRMS spectra of dBO-PAHs	S104−S167
78	Cartesian coordinates of the optimized structures	S168−S279
79	**Supplementary References**	S279−S284

Supplementary Methods

Synthesis and Structure Characterization. Unless noted, all commercial reagents were purchased and used as received without further purification. ^{1}H NMR spectra were recorded at 400 or 500 MHz, and ^{13}C NMR spectra were recorded at 100 or 150 MHz NMR instruments in $CDCl_3$ or DMSO-d_6 solutions and chemical shifts were referenced to tetramethylsilane (TMS) or residual protiated solvent. If $CDCl_3$ was used as solvent, ^{1}H and ^{13}C NMR spectra were recorded with TMS (δ = 0.00 ppm) and $CDCl_3$ (δ = 77.00 ppm) as internal references, respectively. If DMSO-d_6 was used as solvent, ^{1}H and ^{13}C NMR spectra were recorded with TMS (δ = 0.00 ppm) and DMSO-d_6 (δ = 39.52 ppm) as internal references, respectively. The following abbreviations (or combinations thereof) were used to explain ^{1}H NMR ultiplicities: s = singlet, d = doublet, t = triplet, q = quartet, p = quintet, m = multiplet, br = broad. All of the new compounds were analyzed for HRMS on a Waters mass spectrometer using electrospray ionization in positive ion mode of ESI-Q-TOF.

X-ray Crystallography. X-ray diffraction data were collected at 170 K on a Bruker D8 Venture diffractometer using graphite-monochromated Mo-Kα radiation (λ = 0.71073 Å) from a rotating anode generator.

Quantum Chemical Calculations. The theoretical calculations were performed using Gaussian 09. The molecular geometries of ground states (S_0) were optimized with the density functional theory (DFT) method. The DFT calculations were performed using a B3LYP function with a basis set of 6-31G(d) for C, H, O and B atoms. The spin-orbit coupling (SOC) matrix elements between S_1 and T_n states of **BO1a** and **BO1d** and **BO6** were evaluated using PySOC at the B3LYP/6-31G(d,p) level [Gao. X., Bai, D., Fazzi, D., Niehaus, T., Barbatti, M., Thiel, W. *J. Chem. Theory Comput.* **13**, 515−524 (2017)].

Electrochemistry. Cyclic voltammetry and different pulsed voltammetry were performed using a CH1760E electrochemical analyzeraccording previous report. 0.1 M tetra-*n*-butylammonium hexafluorophosphate was used as the supporting electrolyte, anhydrous *N*,*N*-dimethylformamide, was used as the solvents for the E_{ox} and E_{red} measurements, and the solutions were bubbled with nitrogen for 15 min prior to the test. Silver wire, platinum wire and glassy carbon were used as pseudoreference electrode, counter electrode, and working electrode respectively. Scan rate was 300 mV/s. The redox potentials are based on the values measured from different pulsed voltammetry and are reported

relative to an internal reference ferrocenium/ferrocene (Cp_2Fe/Cp_2Fe^+). The reversibility of reduction or oxidation was determined using CV. As defined, if the magnitudes of the peak anodic and the peak cathodic current have an equal magnitude as scan speeds of 100 mV/s or slower, then the process is considered reversible; if the magnitudes of the peak anodic and the peak cathodic currents are not equal, but the return sweeps are nonzero, the process is considered quasi-reversible; otherwise, the process is considered irreversible.

Photophysical Measurements. The absorption spectra were measured on an Agilent 8453 UV−VS Spectrometer. Steady state emission experiments and lifetime measurements were performed on a Horiba Jobin Yvon FluoroLog-3 spectrometer. Low temperature (77 K) emission spectra and lifetimes were measured in 2-MeTHF cooled with liquid nitrogen.

OLED Fabrication and Characterization. All devices were fabricated by vacuum thermal evaporation, and were tested outside glove box after encapsulation. Prior to deposition, the prepatterned ITO coated glass substrates were cleaned by subsequent sonication in deionized water, acetone, and isopropanol. The metal layer and organic layers were fabricated by vacuum thermal evaporation on the cleaned indium-tin-oxide (ITO) glass substrate under vacuum ($< 4 \times 10^{-4}$ Pa) with 4 Å/s deposition rate for aluminum cathode and 2 Å/s for organic layers. The device areas were 9 mm^2 (3 mm × 3 mm). The current density-voltage-luminance characteristics of OLEDs were measured using a Keithey 2400 Source meter and a Keithey 2000 Source multimeter equipped with a calibrated silicon photodiode. The electroluminescence (EL) spectra were recorded with a multichannel spectrometer (PMA12, Hamamatsu Photonics).

Estimation of experimental oscillator strength, and the relationship with k_r and Φ_{PL}. [Turro, N. J., Ramamurthy, V., Scaiano, J. C. Modern Molecular Photochemistry of Organic Molecules; University Science Books; pp195−200, (2009).]

$$\Phi_{PL} = k_r / (k_r + k_{nr}) \qquad (S1)$$

$$k_r = v^2 f \qquad (S2)$$

Where the Φ_{PL} is photoluminescence efficiency, k_r is radiative rate, k_{nr} is non-radiative rate, v is the wavenumber corresponding to the maximum wavelength of absorption, f is oscillator strength. From the **Equation S1** and **Equation S2**, we can see that the f plays an critical role in the increase of Φ_{PL}.

The theoretical quantity of the oscillator strength f in the classical theory of light absorption is

related to the extinction coefficient ε of absorption by the expression:

$$f \equiv 4.3 \times 10^{-9} \int \varepsilon \, d\bar{v} \qquad \text{(S3)}$$

Where ε is the experimental extinction coefficient and $\bar{v}$ is the energy of the absorption. With the assumption that the absorption spectrum is a smooth Gaussian curve which can be approximated by an isosceles triangle, we can have $\int \varepsilon d\bar{v} \sim \varepsilon_{\max} \Delta\bar{v}_{1/2}$, where $\varepsilon_{\max}$ is the value of ε at the absorption maximum and $\Delta\bar{v}_{1/2}$ (in cm^{-1}) is the full-width half-maximum (FWHM) of the absorption band.

With the experiment data extracted from the UV-Vis spectrum and **Equation S3**, the approximate of *f* can be obtained as **Equation S4**:

$$f \sim 4.3 \times 10^{-9}\, \varepsilon_{\max} \Delta\bar{v}_{1/2} \qquad \text{(S4)}$$

Therefore, the *f* of **BO4a**, **BO4b** and **BO4e** can be calculated as:

f (**BO4a**) = $(4.3 \times 10^{-9}) \times (2.95 \times 10^{4}) \times [(1/(348.5 \times 10^{-7}) - (1/(357.5 \times 10^{-7})] = 0.092$.

f (**BO4b**) = $(4.3 \times 10^{-9}) \times (4.15 \times 10^{4}) \times [(1/(352.5 \times 10^{-7}) - (1/(361.0 \times 10^{-7})] = 0.12$.

f (**BO4e**) = $(4.3 \times 10^{-9}) \times (7.57 \times 10^{4}) \times [(1/(379.0 \times 10^{-7}) - (1/(390.0 \times 10^{-7})] = 0.24$.

Moreover, the increase of the *f* from **BO4a**, **BO4b** and **BO4e** can be explained by the relationship between the classical concept of oscillator strength and the quantum mechanical transition dipole moment (**Equation S5**).

$$\text{Oscillator strength } f \propto \boldsymbol{\mu}_{\mathrm{i}}^{2} = (\mathrm{e}\mathbf{r})^{2} \text{ Transition dipole moment} \qquad \text{(S5)}$$

Where $\boldsymbol{\mu}_i$ is the induced transition dipole moment (or dipole strength) corresponding to electronic transition (absorption or emission). The dipole strength of a transition may be set equal to e**r**, which can be viewed as the average size of the transition dipole, where **r** is the diploe length. By combining the classical oscillator strength with the quantization of oscillation of electrons, we have the expression relating *f* and $\boldsymbol{\mu}_i$, which is given by **Equation S6**,

$$f = \left(\frac{8\pi m_{e}\bar{v}}{3he^{2}}\right)\mu_{\mathrm{i}}^{2} \cong 10^{-5}\bar{v}|\mathrm{e}\mathbf{r}_{i}|^{2} \qquad \text{(S6)}$$

The *f* is a function of the $\boldsymbol{\mu}_i$, where m_e is the mess of electron, $\bar{v}$ is the energy of the transition (in cm^{-1}), *h* is Planck's constant. The $\boldsymbol{\mu}_i$ and **r** should be much larger for the delocalized ICT transition in **BO4e** than those in **BO4a** and **BO4b**, this is because **BO4e** with two fluorenyl units possesses longer linear conjugation system compared to **BO4a** and **BO4b**.

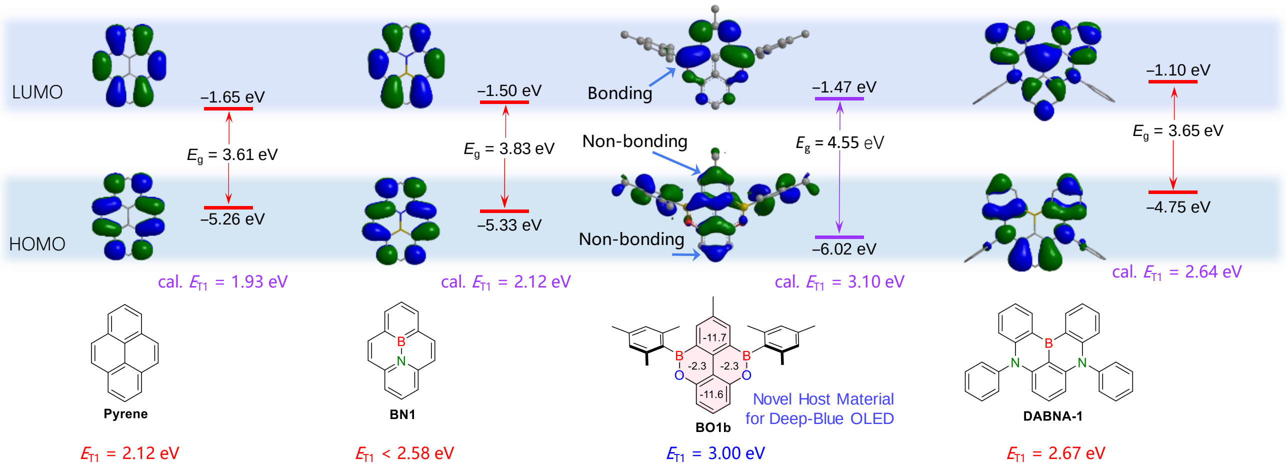

	Pyrene	BN1	BO1b	DABNA-1
MO character	bonding	bonding	bonding/non-bonding	non-bonding
Vibronic coupling	strong/stretching	strong/stretching	moderate	weak/twisting
Emission band	broad/structural	broad/structural	broad/structural	narrow/unstrctural
Aromaticity (heterocycle)	strong	strong	weak	strong
E_{T1}	low	low	high	low

Supplementary Fig. 1. Theoretical calculation, chemical structures and photophysical properties of Pyrene, BN1, BO1b and DABNA-1. Comparison of calculated frontier orbital distributions, energy levels/gaps, calculated and experimental E_{T1}, MO character, aromaticity.

Scheme S1

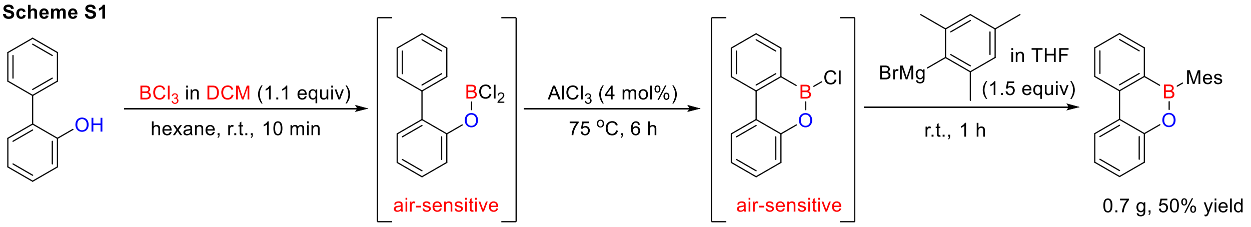

Scheme S2

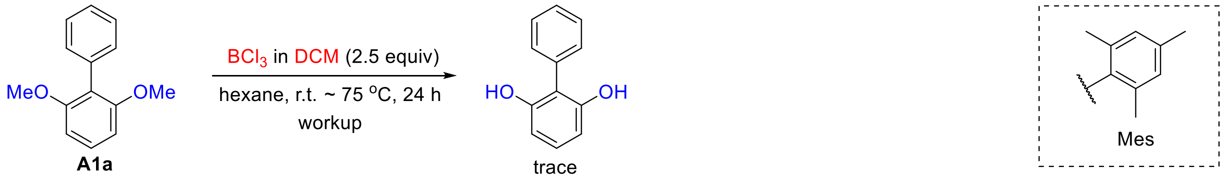

Scheme S3

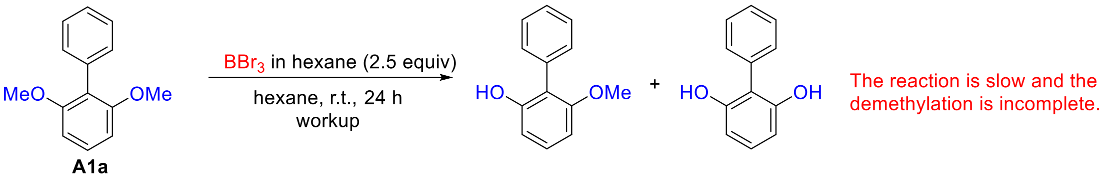

Scheme S4

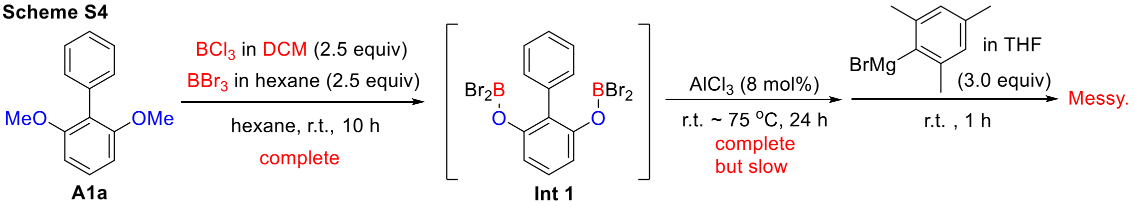

Scheme S5

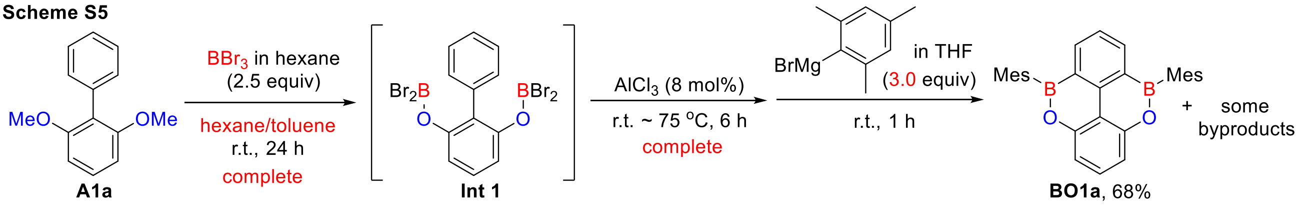

Scheme S6

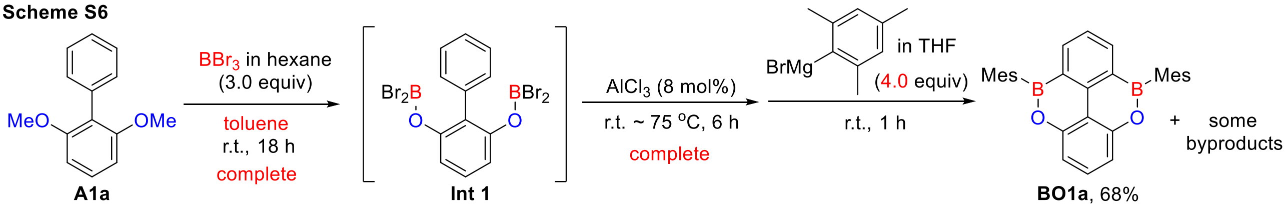

Scheme S7

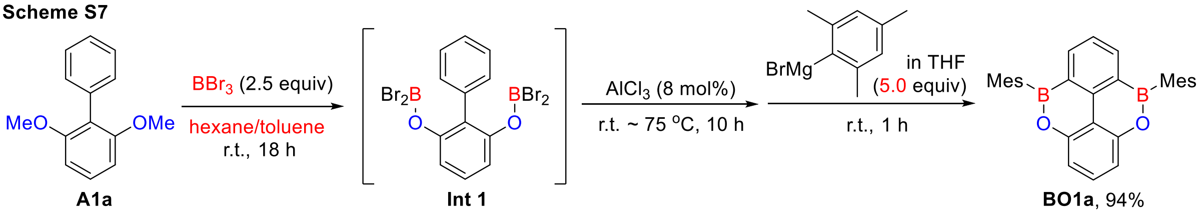

Supplementary Fig. 2. Reaction condition optimization. Template reactions and selected optimization of reaction conditions

Supplementary Discussion

Reaction Condition Optimization. We initiated our examination by carrying out template reactions (see Supporting Information Figure S1, Scheme S1). To our delight, [1,1'-Biphenyl]-2-ol smoothly transformed into 9,10-boroxarophenanthrene in hexane with 50% isolated yield using BCl_3-dichloromethane solution, $AlCl_3$ and mesitylmagnesium bromide (MesMgBr) as boron source, Lewis acid catalyst and nucleophilic reagent, respectively (Scheme S1). Then, we investigated the demethylation reaction of 2,6-dimethoxy-1,1'-biphenyl (**A1a**) using BCl_3-dichloromethane solution, however, only trace desired product [1,1'-biphenyl]-2,6-diol was obtained (Scheme S2), however, the demethylation reaction could be significantly improved through employing BBr_3-hexane solution, although the reaction proceeded slowly and the demethylation was incomplete (Scheme S3). After many optimized reactions, it was found that the demethylation could proceed completely by using a combine of 2.5 equiv BBr_3-dichloromethane and 2.5 equiv BBr_3-hexane solutions, however, this reaction system decelerated the followed electrophilic borylation and also made the final products messy (Scheme S4). This result revealed that the dichloromethane molecules had a great influence on the electrophilic borylation and nucleophilic substitution, which was unfavourable to the B−C bond formation owing to the strong coordination of the chlorine atoms. Thus, as might be expected, 68% isolated yield of **BO1a** could be obtained using only 2.5 equiv BBr_3-hexane solution as demethylation reagent (Scheme S5). Moreover, it was demonstrated that pure BBr_3 also accelerated the demethylation, and nonpolar solvents (hexane/toluene) improved the solubility of the intermediates and enhanced the reaction rate, additionally, the reaction time of the electrophilic borylation was also critical, carefully monitoring was necessary, prolonging the reaction time was not beneficial to the final product (Schemes S5−S7). Finally, 94% isolated yield of **BO1a** in gram scale could be achieved using 2.5 equiv pure BBr_3, 8 mol% $AlCl_3$ and 5.0 equiv MesMgBr for the corresponding demethylation, electrophilic borylation and nucleophilic substitution steps, respectively (Scheme S7). Notably, bulky Mes groups were incorporated to enhance molecular stability, which protected the boron from nucleophilic reagents. The attempt for the nucleophilic substitution of using (2,4,6-triisopropylphenyl)lithium was unsuccessful typically owing to its excessive steric hindrance.

General Experimental Procedure for the Synthesis of Dimethoxy Compounds and Double BO-Fused Polycyclic Aromatic Hydrocarbons (dBO-PAHs).

Aryl bromide or aryl iodide, aryl boronic acid and $Pd(PPh_3)_4$ were added to a dry three-necked flask equipped with a magnetic stir bar and a condensor. The flask was then evacuated and backfilled with nitrogen, this evacuation and backfill procedure was repeated twice. Then solvent dioxane and a solution of K_2CO_3 in H_2O were added under nitrogen atmosphere. The mixture was bubbled with nitrogen for 20 minutes and the flask was placed in oil bath (95 or 100 ºC) and stirred for 24 hour until the starting material was consumed completely monitoring by TLC. Then the mixture was cooled down, diluted with ethyl acetate. The organic layer was then separated, and aqueous layer was extracted with ethyl acetate twice. The combined organic layer was dried over anhydrous Na_2SO_4, filtered and concentrated under reduced pressure. The residue was purified through column chromatography on silica gel using petroleum ether/ethyl acetate as eluent to afford the desired dimethoxy compound.

The dimethoxy compound was added to a dry three-necked flask equipped with a magnetic stir bar and a condensor. The flask was then evacuated and backfilled with nitrogen, this evacuation and backfill procedure was repeated twice. Then solvent toluene and *n*-hexane were added under nitrogen atmosphere, then BBr_3 was added dropwise. The mixture was stirred at room temperature for 18–40 hours monitoring by TLC until the demethylation was completely. Then $AlCl_3$ was added quickly (The $AlCl_3$ is highly hygroscopic and should be weighed quickly!), the flask was placed in oil bath (75 ºC) and stirred for 8–10 hours, and then the mixture was cooled down to room temperature. Mesitylmagnesium bromide (MesMgBr) was then added dropwise, and the mixture was then stirred at room temperature for another 1–10 hours monitoring by TLC until the reaction was completely. The reaction mixture was concentrated under reduced pressure, and the residue was purified through column chromatography on silica gel using petroleum ether/dichloromethane as eluent to afford the desired double BO-fused polycyclic aromatic hydrocarbon.

Synthesis of **BO1a**:

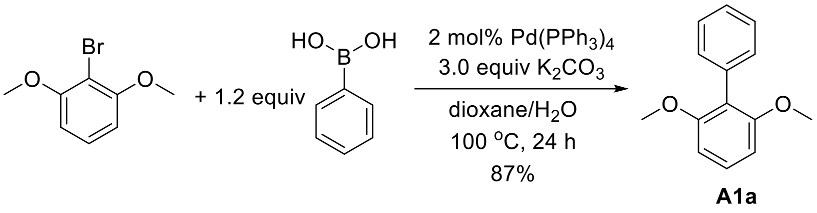

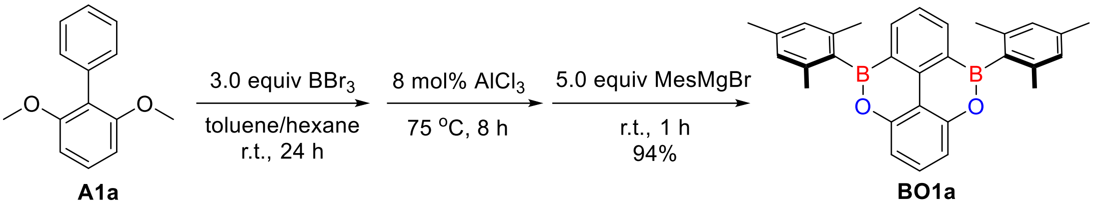

Synthesis of **A1a**: Following the general procedure, the reaction of 2-bromo-1,3-dimethoxybenzene (2.17 g, 10.00 mmol, 1.0 equiv), phenylboronic acid (1.46 g, 12.00 mmol, 1.2 equiv), $Pd(PPh_3)_4$ (213 mg, 0.20 mmol, 0.02 equiv) and K_2CO_3 (4.15 g, 30.00 mmol, 3.0 equiv, in 15 mL H_2,) in dioxane (60 mL) at 100 °C for 24 hours afforded **A1a** (eluent: petroleum ether/ethyl acetate = 10:1–5:1) as a white solid 1.86 g in 87% yield. ^{1}H NMR (400 MHz, $CDCl_3$): δ (ppm) 3.78 (s, 6H), 6.71 (d, J = 8.4 Hz, 2H), 7.34 (t, J = 8.4 Hz, 2H), 7.37−7.49 (m, 4H). ^{13}C NMR (125 MHz, $CDCl_3$): δ (ppm) 55.81, 104.14, 119.49, 126.68, 127.59, 128.57, 130.83, 134.08, 157.60. The ^{1}H and ^{13}C NMR data are agreement with the previous reoprt (Truong, T., Daugulis, O. Base-mediated intermolecular sp^2 C-H bond arylation via benzyne intermediates. *J. Am. Chem. Soc.* **133**, 4243–4245 (2011).

Synthesis of **BO1a**: Following the general procedure, BBr_3 (1.16 mL, d = 2.6 g/mL, 12.00 mmol, 3.0 equiv) was added dropwise to a mixture of **A1a** (857 mg, 4.00 mmol, 1.0 equiv) in toluene (40 mL) and *n*-hexane (40 mL) and stirred at room temperature for 24 hours, added $AlCl_3$ (42 mg, 0.32 mmol, 0.08 equiv) and stirred at 75 °C for 8 hours, cooled down to room temperature, added MesMgBr (20 mL, 1.0 M in THF, 20 mmol, 5.0 equiv) and stirred at room temperature for another 1 hour to afford **BO1a** (eluent: petroleum ether/dichloromethane = 100:1–10:1) as a white solid 1.66 g in 94% yield. ^{1}H NMR (500 MHz, DMSO-d_6): δ (ppm) 2.15 (s, 12H), 2.33 (s, 6H), 6.95 (s, 4H), 7.57 (d, J = 8.5 Hz, 2H), 7.75−7.79 (m, 2H), 8.08 (d, J = 7.0 Hz, 2H). ^{13}C NMR (125 MHz, $CDCl_3$): δ (ppm) 21.31, 22.56, 112.91, 113.69, 126.80, 127.18, 127.43, 128.91, 133.89, 138.52, 140.09, 140.15, 141.65, 151.50. ^{11}B NMR (160 MHz, $CDCl_3$): δ (ppm) 46.31. HRMS (ESI): calcd for $C_{30}H_{28}[^{11}B]_2NaO_2^+$ $[M+Na]^+$ 465.2168, found 465.2185.

Synthesis of **BO1b**:

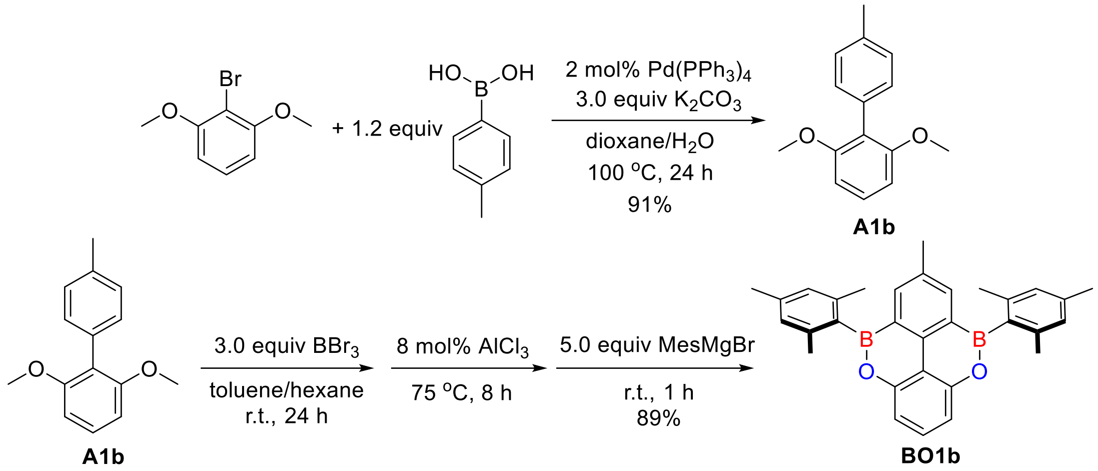

Synthesis of **A1b**: Following the general procedure, the reaction of 2-bromo-1,3-dimethoxybenzene (2.17 g, 10.00 mmol, 1.0 equiv), 4-methylphenylboronic acid (1.63 g, 12.00 mmol, 1.2 equiv), $Pd(PPh_3)_4$ (213 mg, 0.20 mmol, 0.02 equiv) and K_2CO_3 (4.15 g, , 30.00 mmol, 3.0 equiv, in 15 mL H_2O) in dioxane (60 mL) at 100 °C for 24 hours afforded **A1b** (eluent: petroleum ether/ethyl acetate = 10:1–5:1) as a white solid 2.08 g in 91% yield. ^{1}H NMR (500 MHz, DMSO-d_6): δ (ppm) 2.32 (s, 3H), 3.64 (s, 6H), 6.72 (d, J = 8.5 Hz, 2H), 7.10−7.03 (m, 2H), 7.14 (d, J = 8.0 Hz, 2H), 7.27 (t, J = 8.5 Hz, 1H). The ^{1}H NMR is agreement with the previous reoprt (Dai, J.-J., Liu, J.-H., Luo, D.-F., Liu, L. Pd-catalysed decarboxylative Suzuki reactions and orthogonal Cu-based O-arylation of aromatic carboxylic acids. *Chem. Commun.* **47***,* 677–679 (2011).

Synthesis of **BO1b**: Following the general procedure, BBr_3 (1.16 mL, d = 2.6 g/mL, 12.00 mmol, 3.0 equiv) was added dropwise to a mixture of **A1b** (913 mg, 4.00 mmol, 1.0 equiv) in toluene (40 mL) and *n*-hexane (40 mL) and stirred at room temperature for 24 hours, added $AlCl_3$ (42 mg, 0.32 mmol, 0.08 equiv) and stirred at 75 °C for 8 hours, cooled down to room temperature, added MesMgBr (20 mL, 1.0 M in THF, 20 mmol, 5.0 equiv) and stirred at room temperature for another 1 hour to afford **BO1b** (eluent: petroleum ether/dichloromethane = 100:1–10:1) as a white solid 1.62 g in 89% yield. m.p.: 276.2−277.9 °C. ^{1}H NMR (500 MHz, $CDCl_3$): δ (ppm) 2.26 (s, 12H), 2.40 (s, 6H), 2.46 (s, 3H), 6.99 (s, 4H), 7.49 (d, J = 8.0 Hz, 2H), 7.61−7.64 (m, 1H), 8.00 (s, 2H). ^{13}C NMR (125 MHz, $CDCl_3$): δ (ppm) 21.33, 21.52, 22.57, 112.97, 113.56, 126.85, 127.41, 128.33, 134.11, 137.11, 137.79, 138.49, 140.20, 142.12, 151.28. ^{11}B NMR (160 MHz, $CDCl_3$): δ (ppm) 49.59. HRMS (ESI): calcd for $C_{31}H_{31}[^{11}B]_2O_2^+$ $[M+H]^+$ 457.2505, found 457.2521.

Large scale synthesis of **BO1b**:

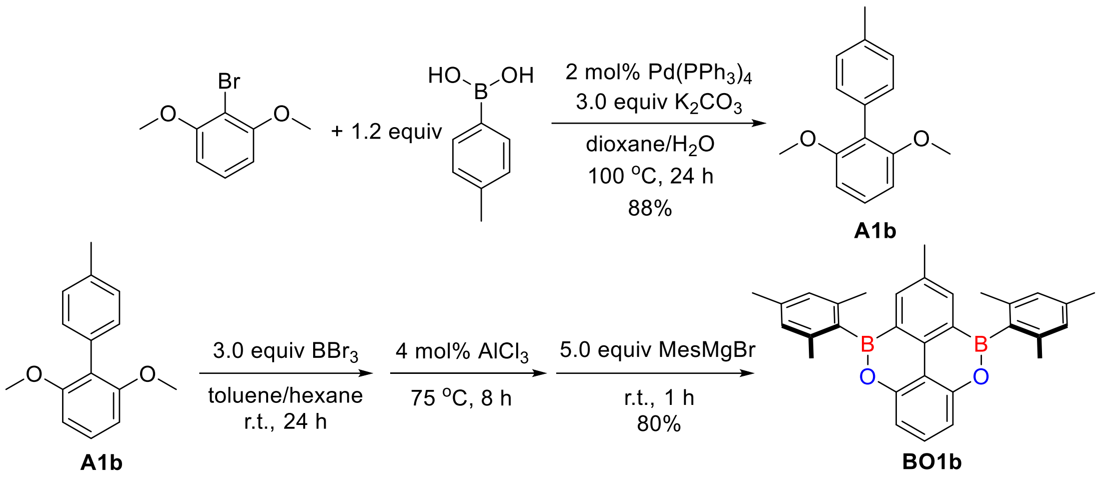

Synthesis of **A1b**: Following the general procedure, the reaction of 2-bromo-1,3-dimethoxybenzene (13.02 g, 60.00 mmol, 1.0 equiv), 4-methylphenylboronic acid (9.79 g, 72.00 mmol, 1.2 equiv), $Pd(PPh_3)_4$ (1.39 mg, 1.20 mmol, 0.02 equiv) and K_2CO_3 (24.89 g, 180.00 mmol, 3.0 equiv, in 40 mL H_2O) in dioxane (160 mL) at 100 °C for 24 hours afforded **A1b** (eluent: petroleum ether/ethyl acetate = 20:1–10:1) as a white solid 12.00 g in 88% yield. The structure was confirmed by ^{1}H NMR, which was in agreement with the above result.

Synthesis of **BO1b**: Following the general procedure, BBr_3 (7.60 mL, d = 2.6 g/mL, 78.84 mmol, 3.0 equiv) was added dropwise to a mixture of **A1b** (6.00 g, 26.28 mmol, 1.0 equiv) in toluene (150 mL) and *n*-hexane (150 mL) and stirred at room temperature for 24 hours, added $AlCl_3$ (140 mg, 1.05 mmol, 0.04 equiv) and stirred at 75 °C for 8 hours, cooled down to room temperature, added MesMgBr (131.40 mL, 1.0 M in THF, 131.40 mmol, 5.0 equiv) and stirred at room temperature for another 1 hour to afford **BO1b** (eluent: petroleum ether/dichloromethane = 100:1–10:1) as a white solid 9.56 g in 80% yield. The structure was confirmed by ^{1}H NMR, which was in agreement with the above result. The obtained **BO1b** was further purified by recrystallization in toluene/methanol; and then 2.5 g **BO1b** was sublimated in a seven zone thermal gradient sublimator at 40/100/130/150/250/250/270 ºC, 1.6×10^{-6} Torr to obtain white solid 2.1 g in 84% yield; purity, 99.90% by HPLC, HPLC analysis condition: column: Masiall® C18-BIO, 5μm, 250 × 4.6 mm; mobile phase: methanol/tetrahydrofuran = 95/5(v/v); flow rate: 1.0 mL/min; Abs. detector: 254 nm.

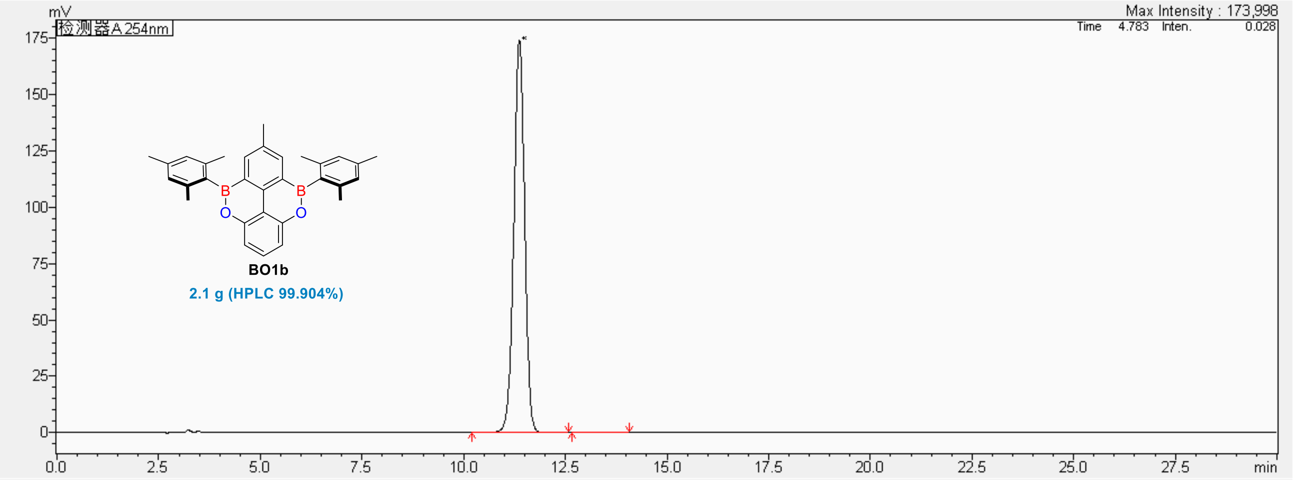

Peak	RetTime /[min]	Area /[mAU*s]	Area /%	Height /[mAU]	Start Time /[min]	End Time/ [min]
1	11.371	3235687	99.904	173993	10.190	12.590
2	13.357	3120	0.096	112	12.670	14.070
Total		3238807	100.000	174105		

Synthesis of **BO1c**:

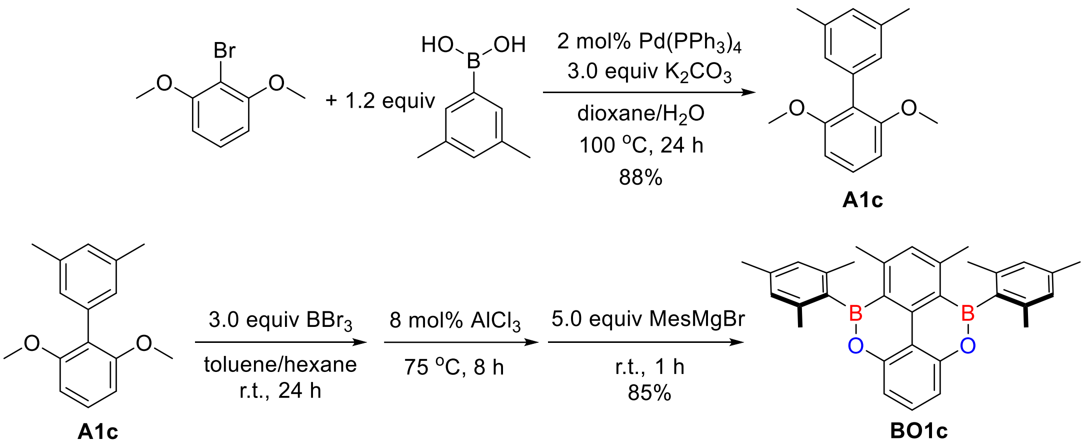

Synthesis of **A1c**: Following the general procedure, the reaction of 2-bromo-1,3-dimethoxybenzene (2.17 g, 10.00 mmol, 1.0 equiv), 3,5-dimethylphenylboronic acid (1.80 g, 12.00 mmol, 1.2 equiv), $Pd(PPh_3)_4$ (213 mg, 0.20 mmol, 0.02 equiv) and K_2CO_3 (4.15 g, 30.00 mmol, 3.0 equiv, in 15 mL H_2O) in dioxane (60 mL) at 100 °C for 24 hours afforded **A1c** (eluent: petroleum ether/ethyl acetate = 10:1–5:1) as a white solid 2.13 g in 88% yield. ^{1}H NMR (500 MHz, DMSO-d_6): δ (ppm) 2.26 (s, 6H), 3.63 (s, 6H), 6.70 (d, J = 8.5 Hz, 2H), 6.75 (s, 2H), 6.88 (s, 1H), 7.27 (t, J = 8.5 Hz, 1H).

Synthesis of **BO1c**: Following the general procedure, BBr_3 (1.16 mL, d = 2.6 g/mL, 12.00 mmol, 3.0 equiv) was added dropwise to a mixture of **A1c** (969 mg, 4.00 mmol, 1.0 equiv) in toluene (40 mL) and *n*-hexane (40 mL) and stirred at room temperature for 24 hours, added $AlCl_3$ (42 mg, 0.32 mmol, 0.08 equiv) and stirred at 75 °C for 8 hours, cooled down to room temperature, added MesMgBr (20 mL, 1.0 M in THF, 20 mmol, 5.0 equiv) and stirred at room temperature for another 1 hour to afford

BO1c (eluent: petroleum ether/dichloromethane = 100:1–10:1) as a white solid 1.62 g in 85% yield. m.p.: 209.8−210.0 °C. ^{1}H NMR (500 MHz, $CDCl_3$): δ (ppm) 2.21 (s, 12H), 2.28 (s, 6H), 2.36 (s, 6H), 6.93 (s, 4H), 7.27 (s, 1H), 7.41 (d, J = 8.0 Hz, 2H), 7.57 (t, J = 8.0 Hz, 1H). ^{13}C NMR (125 MHz, $CDCl_3$): δ (ppm) 21.31, 22.13, 23.24, 112.87, 113.15, 123.50, 127.58, 128.67, 133.31, 137.94, 138.31, 139.30, 143.01, 151.45, 152.98. ^{11}B NMR (160 MHz, $CDCl_3$): δ (ppm) 45.80. HRMS (ESI): calcd for $C_{32}H_{33}[^{11}B]_2O_2^+$ $[M+H]^+$ 471.2661, found 471.2677.

Large scale synthesis of **BO1c**:

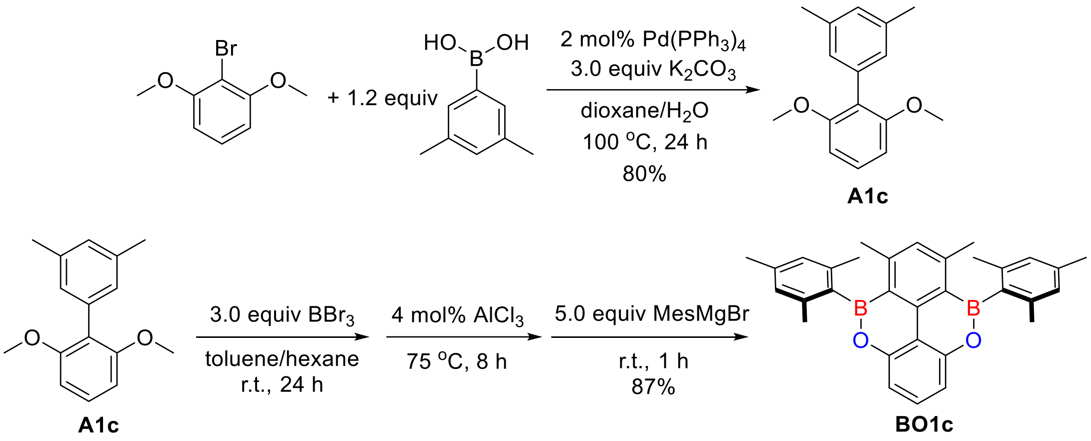

Synthesis of **A1c**: Following the general procedure, the reaction of 2-bromo-1,3-dimethoxybenzene (10.85 g, 50.00 mmol, 1.0 equiv), 3,5-dimethylphenylboronic acid (9.00 g, 60.00 mmol, 1.2 equiv), $Pd(PPh_3)_4$ (1.16 g, 1.00 mmol, 0.02 equiv) and K_2CO_3 (20.73 g, 150.00 mmol, 3.0 equiv, in 20 mL H_2O) in dioxane (80 mL) at 100 °C for 24 hours afforded **A1c** (eluent: petroleum ether/ethyl acetate = 10:1–5:1) as a white solid 9.69 g in 80% yield. The structure was confirmed by ^{1}H NMR, which was in agreement with the above result.

Synthesis of **BO1c**: Following the general procedure, BBr_3 (11.57 mL, d = 2.6 g/mL, 120.00 mmol, 3.0 equiv) was added dropwise to a mixture of **A1c** (9.69 g, 40.00 mmol, 1.0 equiv) in toluene (200 mL) and *n*-hexane (200 mL) and stirred at room temperature for 24 hours, added $AlCl_3$ (213 mg, 1.60 mmol, 0.04 equiv) and stirred at 75 °C for 8 hours, cooled down to room temperature, added MesMgBr (200 mL, 1.0 M in THF, 200 mmol, 5.0 equiv) and stirred at room temperature for another 1 hour to afford **BO1c** (eluent: petroleum ether/dichloromethane = 100:1–10:1) as a white solid 16.30 g in 87% yield. The structure was confirmed by ^{1}H NMR, which was in agreement with the above result. The obtained **BO1c** was further purified by recrystallization in toluene/methanol; and then 6.4 g **BO1c** was sublimated in a seven zone thermal gradient sublimator at 40/100/130/150/250/250/270 °C, 5.0×10^{-5} Torr to obtain white solid 6.0 g in 94% yield; purity, 99.56% by HPLC, HPLC analysis condition: column: Masiall® C18-BlO, 5μm, 250 × 4.6 mm; mobile phase: methanol/tetrahydrofuran = 95/5(v/v); flow rate: 1.0 mL/min; Abs. detector: 254 nm.

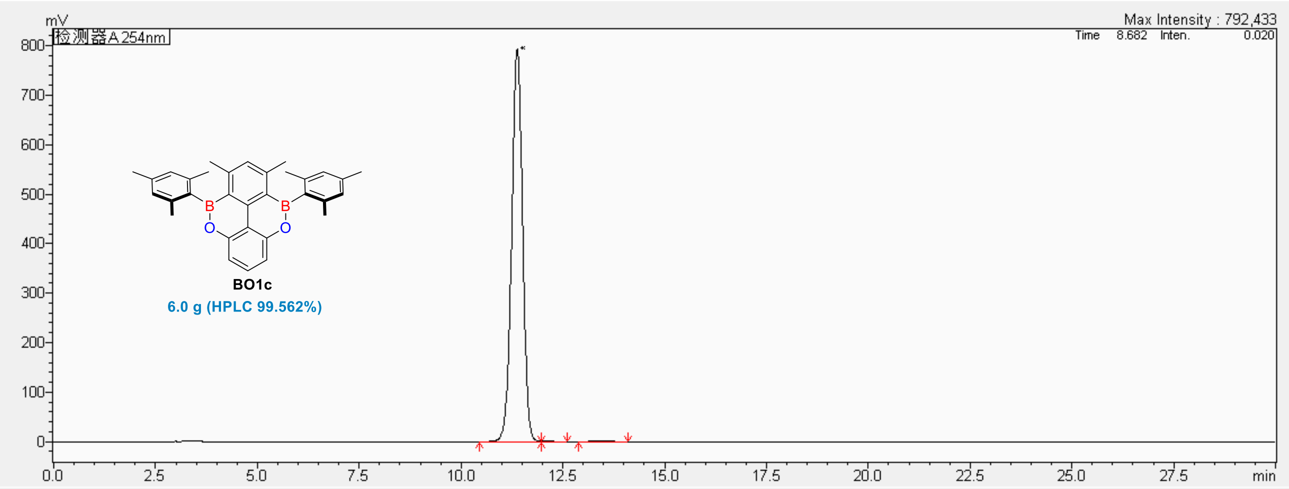

Peak	RetTime /[min]	Area /[mAU*s]	Area /%	Height /[mAU]	Start Time /[min]	End Time/ [min]
1	11.383	14821692	99.562	792395	10.450	11.960
2	11.975	11425	0.077	821	11.960	12.600
3	13.448	53777	0.361	2488	12.890	14.090
Total		14886894	100.000	795704		

Synthesis of **BO1d**:

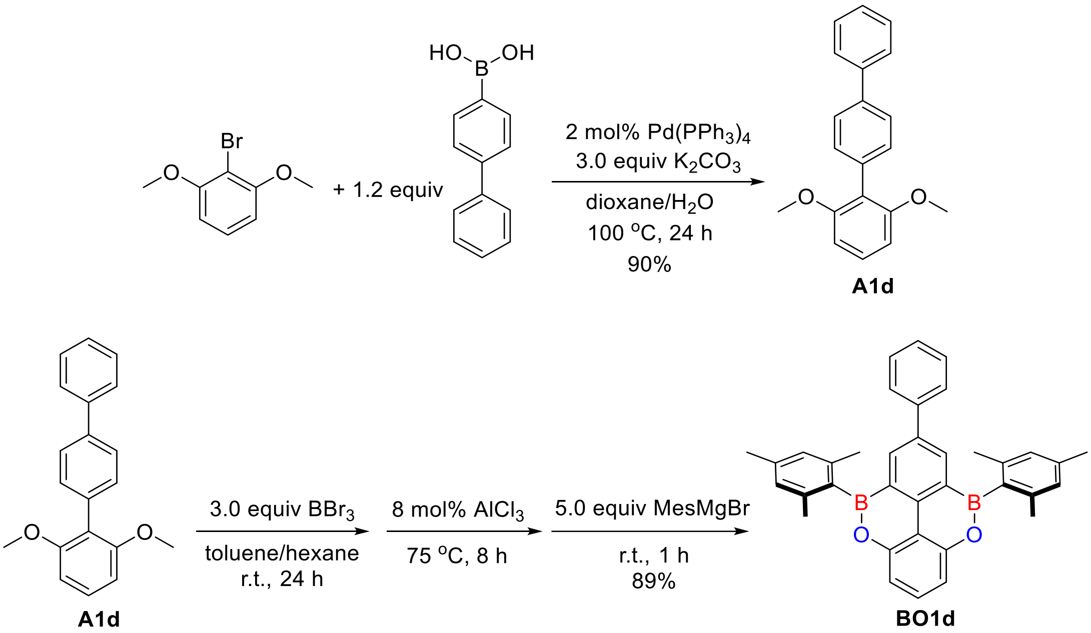

Synthesis of **A1d**: Following the general procedure, the reaction of 2-bromo-1,3-dimethoxybenzene (2.17 g, 10.00 mmol, 1.0 equiv), [1,1'-biphenyl]-4-ylboronic acid (2.38 g, 12.00 mmol, 1.2 equiv), $Pd(PPh_3)_4$ (213 mg, 0.20 mmol, 0.02 equiv) and K_2CO_3 (4.15 g, 30.00 mmol, 3.0 equiv, in 15 mL H_2O) in dioxane (60 mL) at 100 °C for 24 hours afforded **A1d** (eluent: petroleum ether/ethyl acetate = 10:1–5:1) as a white solid 2.61 g in 90% yield. m.p.: 205.7−206.2 °C. ^{1}H NMR (500 MHz, DMSO-d_6): δ (ppm) 3.68 (s, 6H), 6.76 (d, J = 8.5 Hz, 2H), 7.26−7.34 (m, 3H), 7.37 (t, J = 7.5 Hz, 1H), 7.48 (t, J = 7.5 Hz, 2H), 7.64 (d, J = 8.5 Hz, 2H), 7.70 (d, J = 7.0 Hz, 2H). ^{13}C NMR (125 MHz, $CDCl_3$): δ (ppm) 55.88, 104.18, 118.99, 126.41, 126.97, 127.11, 128.61, 128.68, 131.30, 133.07,

139.39, 141.24, 157.67. The ^{13}C NMR is agreement with the previous reoprt (Crowley , B. M., Potteiger, C. M., Deng, J. Z., Prier, C. K., Paone, D. V., Burgey, C. S. Expanding the scope of the Cu assisted Suzuki–Miyaura reaction. *Tetrahedron Lett*. **52**, 5055–5059 (2011).

Synthesis of **BO1d**: Following the general procedure, BBr_3 (1.16 mL, d = 2.6 g/mL, 12.00 mmol, 3.0 equiv) was added dropwise to a mixture of **A1d** (1.16 g, 4.00 mmol, 1.0 equiv) in toluene (40 mL) and *n*-hexane (40 mL) and stirred at room temperature for 24 hours, added $AlCl_3$ (42 mg, 0.32 mmol, 0.08 equiv) and stirred at 75 °C for 8 hours, cooled down to room temperature, added MesMgBr (20 mL, 1.0 M in THF, 20 mmol, 5.0 equiv) and stirred at room temperature for another 1 hour to afford **BO1d** (eluent: petroleum ether/dichloromethane = 100:1–10:1) as a white solid 1.85 g in 89% yield. ^1H NMR (500 MHz, DMSO-d_6): δ (ppm)2.20 (s, 12H), 2.33 (s, 6H), 6.96 (s, 4H), 7.36−7.40 (m, 1H), 7.45−7.48 (m, 2H), 7.51−7.53 (m, 2H), 7.59 (d, J = 8.0 Hz, 2H), 7.78 (t, J = 8.0 Hz, 1H), 8.20 (s, 2H). ^{13}C NMR (125 MHz, $CDCl_3$): δ (ppm) 21.31, 22.65, 112.74, 113.71, 125.75, 127.53, 127.65, 128.79, 128.94, 129.54, 133.79, 138.53, 139.15, 140.16, 140.19, 140.30, 140.44, 151.45. ^{11}B NMR (160 MHz, $CDCl_3$): δ (ppm) 48.79. HRMS (ESI): calcd for $C_{36}H_{33}[^{11}B]_2O_2^+$ $[M+H]^+$ 519.2661, found 519.2673.

Synthesis of **BO1e**:

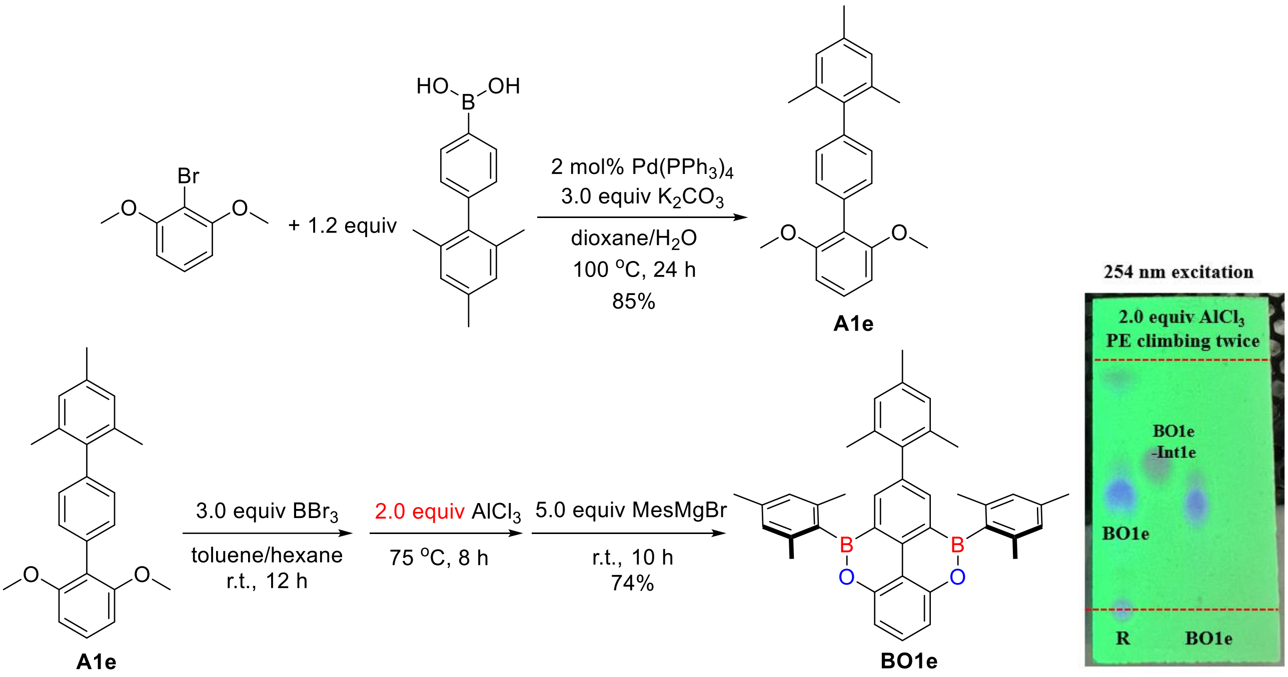

Synthesis of **A1e**: Following the general procedure, the reaction of 2-bromo-1,3-dimethoxybenzene (2.17 g, 10.00 mmol, 1.0 equiv), (2',4',6'-trimethyl-[1,1'-biphenyl] -4-yl)boronic acid (2.89 g, 12.00 mmol, 1.2 equiv), $Pd(PPh_3)_4$ (213 mg, 0.20 mmol, 0.02 equiv) and K_2CO_3 (4.15 g, 30.00 mmol, 3.0 equiv, in 15 mL H_2O) in dioxane (60 mL) at 100 °C for 24 hours afforded **A1e** (eluent: petroleum ether/ethyl acetate = 10:1–5:1) as a white solid 2.83 g in 85% yield. ^1H NMR (500 MHz, $CDCl_3$): δ (ppm) 2.10 (s, 6H), 2.34 (s, 3H), 3.76 (s, 6H), 6.68 (d, J = 8.5 Hz, 2H), 6.96 (s, 2H), 7.16 (dt , J = 8.5,

1.5 Hz, 2H), 7.28 (t, J = 8.5 Hz, 1H), 7.38 (dt , J = 8.0, 2.0 Hz, 2H). ^{13}C NMR (125 MHz, $CDCl_3$): δ (ppm) 20.91, 21.00, 55.98, 104.49, 119.66, 127.99, 128.53, 130.79, 132.16, 136.24, 136.32, 139.06, 139.20, 157.80.Synthesis of **BO1e**: Following the general procedure, BBr_3 (0.29 mL, d = 2.6 g/mL, 3.00 mmol, 3.0 equiv) was added dropwise to a mixture of **A1e** (332 mg, 1.00 mmol, 1.0 equiv) in toluene (5 mL) and *n*-hexane (5 mL) and stirred at room temperature for 12 hours, added $AlCl_3$ (267 mg, 2.00 mmol, 2.00 equiv) and stirred at 75 °C for 8 hours, cooled down to room temperature, added MesMgBr (5.0 mL, 1.0 M in THF, 5.00 mmol, 5.0 equiv) and stirred at room temperature for another 10 hour to afford **BO1e** (eluent: petroleum ether/dichloromethane = 100:1–10:1) as a white solid 417 mg in 74% yield. ^1H NMR (500 MHz, $CDCl_3$): δ (ppm) 2.18 (s, 3H), 2.28 (s, 12H), 2.31 (s, 6H), 2.38 (s, 6H), 6.97 (s, 4H), 7.12 (s, 2H), 7.49 (d, J = 8.5 Hz, 2H), 7.62–7.65 (m, 1H), 8.33 (s, 2H). ^{13}C NMR (125 MHz, $CDCl_3$): δ (ppm) 15.13, 20.65, 21.32, 22.65, 112.83, 113.62, 126.96, 127.27, 127.49, 128.73, 133.98, 134.64, 136.90, 137.65, 138.47, 138.84, 140.26, 140.80, 151.41. ^{11}B NMR (160 MHz, $CDCl_3$): δ (ppm) 47.17. HRMS (ESI): calcd for $C_{39}H_{39}[^{11}B]_2O_2^+$ $[M+H]^+$ 561.3131, found 561.3127.

Synthesis of **BO1f**:

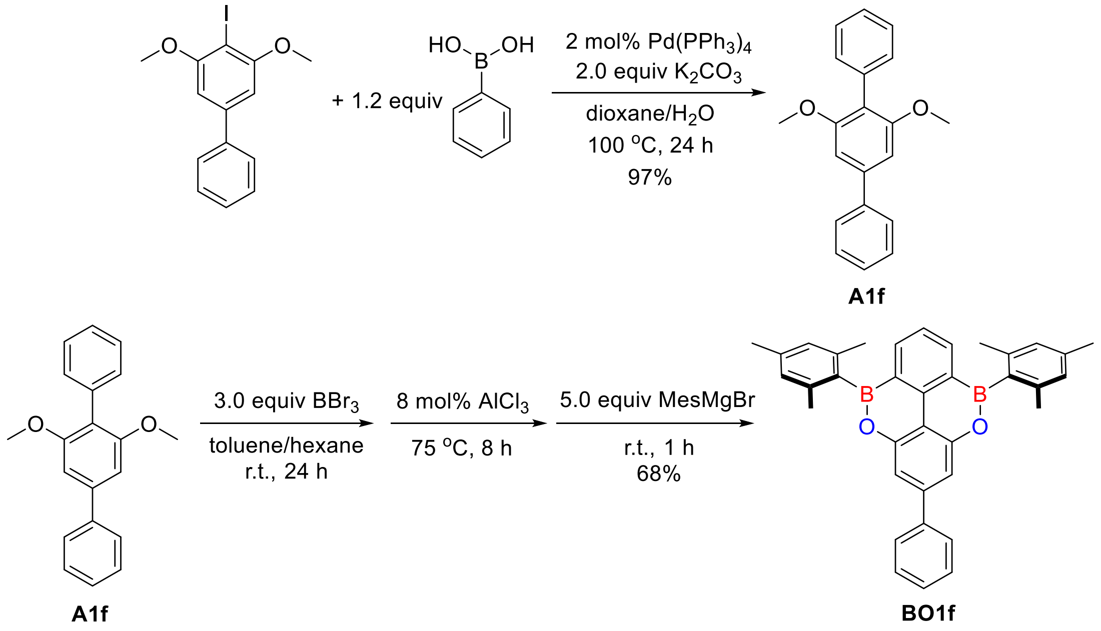

Synthesis of **A1f**: Following the general procedure, the reaction of 4-iodo-3,5-dimethoxy-1,1'-biphenyl (680 mg, 2.00 mmol, 1.0 equiv), phenylboronic acid (293 mg, 2.40 mmol, 1.2 equiv), $Pd(PPh_3)_4$ (46 mg, 0.04 mmol, 0.02 equiv) and K_2CO_3 (552 mg, 4.00 mmol, 2.0 equiv, in 5 mL H_2O) in dioxane (20 mL) at 100 °C for 24 hours afforded **A1f** (eluent: petroleum ether/ethyl acetate = 10:1) as a white solid 564 mg in 97% yield. ^1H NMR (500 MHz, $CDCl_3$): δ (ppm) 3.80 (s, 6H), 6.86 (s, 2H), 7.31–7.35 (m, 1H), 7.37–7.44 (m, 5H), 7.46–7.49 (m, 2H), 7.64–7.66 (m, 2H). ^{13}C NMR (125 MHz, $CDCl_3$): δ (ppm) 55.96, 103.45, 118.52, 126.80, 127.17, 127.55, 127.66, 128.75, 130.92, 133.87, 141.51, 142.15, 157.79.

Synthesis of **BO1f**: Following the general procedure, BBr_3 (0.29 mL, d = 2.6 g/mL, 3.00 mmol, 3.0 equiv) was added dropwise to a mixture of **A1f** (290 mg, 1.00 mmol, 1.0 equiv) in toluene (20 mL) and *n*-hexane (20 mL) and stirred at room temperature for 24 hours, added $AlCl_3$ (12 mg, 0.08 mmol, 0.08 equiv) and stirred at 75 °C for 8 hours, cooled down to room temperature, added MesMgBr (5 mL, 1.0 M in THF, 5.00 mmol, 5.0 equiv) and stirred at room temperature for another 1 hour to afford **BO1f** (eluent: petroleum ether/dichloromethane = 100:1–10:1) as a white solid 355 mg in 68% yield. ^{1}H NMR (500 MHz, $CDCl_3$): δ (ppm) 2.26 (s, 12H), 2.38 (s, 6H), 6.97 (s, 4H), 7.41 (t, J = 6.0 Hz, 1H), 7.50 (t, J = 6.5 Hz, 2H), 7.61 (t, J = 6.0 Hz, 1H), 7.75 (s, 1H), 7.76 (s, 3H), 8.18 (d, J = 6.0 Hz, 2H). ^{13}C NMR (125 MHz, $CDCl_3$): δ (ppm) 21.32, 22.58, 111.86, 112.31, 126.71, 127.16, 127.35, 127.44, 128.00, 129.02, 133.82, 138.54, 139.99, 140.07, 140.14, 141.74, 142.31, 151.69. ^{11}B NMR (160 MHz, $CDCl_3$): δ (ppm) 46.99. HRMS (ESI): calcd for $C_{36}H_{33}[^{11}B]_2O_2^+$ $[M+H]^+$ 519.2661, found 519.2672.

Synthesis of **BO1g**:

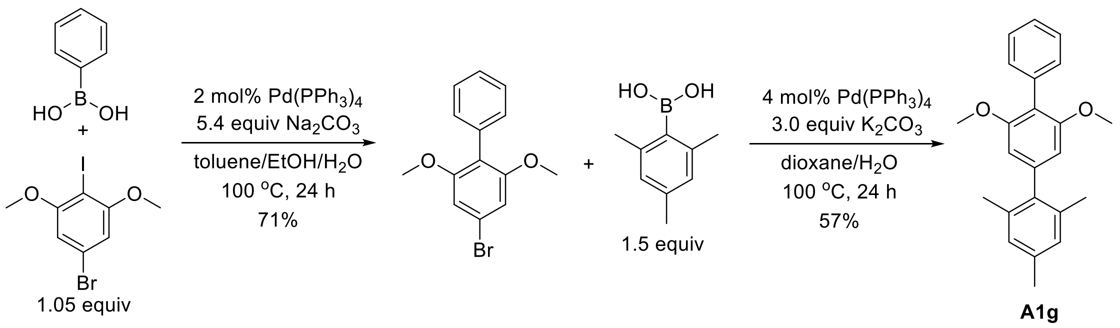

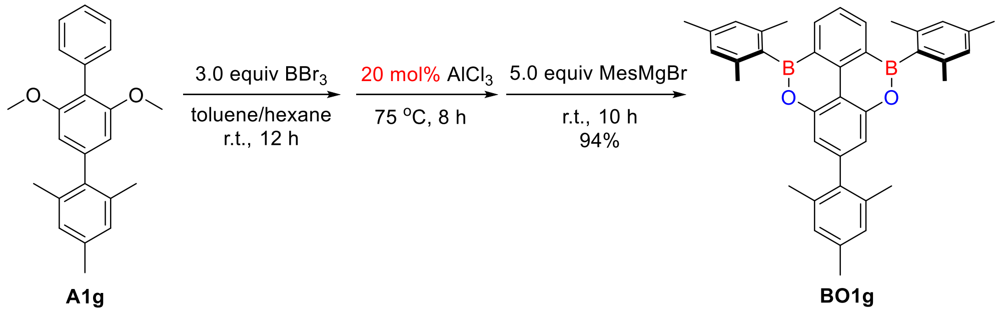

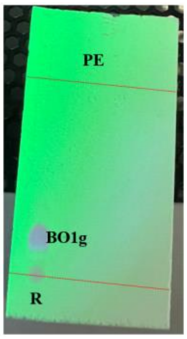

Synthesis of 4-bromo-2,6-dimethoxy-1,1'-biphenyl: Following the general procedure, the reaction of 5-bromo-2-iodo-1,3-dimethoxybenzene (3.6 g, 10.5 mmol, 1.05 equiv), phenylboronic acid (1.21 g, 11.70 mmol, 1.0 equiv), $Pd(PPh_3)_4$ (270 mg, 0.23 mmol, 0.02 equiv) and Na_2CO_3 (6.70 g, 63.18 mmol, 5.4 equiv, in 10 mL H_2O) in dioxane (40 mL) and EtOH (10 mL) at 100 ºC for 24 hours afforded 4-bromo-2,6-dimethoxy-1,1'-biphenyl (eluent: petroleum ether/dichloromethane = 50:1–30:1) as a white solid 2.08 g in 71% yield, which was used directly for the next step. The 5-bromo-2-iodo-1,3-dimethoxybenzene was synthesized according the previous report (Wang, G., Chen, M., Wang, J., Jiang, Z., Liu, D., Lou, D., Zhao, H., Li, K., Li, S., Wu, T., Jiang, Z., Sun, X., Wang, P. Reinforced

topological nanoassemblies: 2D hexagon-fused wheel to 3D prismatic metallo-lamellar structure with molecular weight of 119 K daltons. *J. Am. Chem. Soc.* **1421**, 7690–7698 (2020).

Synthesis of **A1g**: Following the general procedure, the reaction of 4-bromo-2,6-dimethoxy-1,1'-biphenyl (1.99 g, 7.00 mmol, 1.0 equiv), phenylboronic acid (1.64 g, 10.50 mmol, 1.5 equiv), $Pd(PPh_3)_4$ (324 mg, 0.28 mmol, 0.04 equiv) and K_2CO_3 (2.90 g, 21.00 mmol, 2.0 equiv, in 5 mL H_2O) in dioxane (20 mL) at 100 °C for 24 hours afforded **A1g** (eluent: petroleum ether/dichloromethane = 40:1–10:1) as a white solid 1.27 g in 57% yield. ^1H NMR (500 MHz, $CDCl_3$): δ (ppm) 2.13 (s, 6H), 2.35 (s, 3H), 3.69 (s, 6H), 6.43 (s, 2H), 6.98 (s, 2H), 7.31−7.33 (m, 1H), 7.42 (d, J = 4.5 Hz, 4H). ^{13}C NMR (125 MHz, $CDCl_3$): δ (ppm) 20.64, 20.99, 55.93, 105.29, 117.52, 126.66, 127.61, 128.05, 131.07, 134.17, 136.02, 136.76, 139.28, 141.68, 157.51.

Synthesis of **BO1g**: Following the general procedure, BBr_3 (0.63 mL, d = 2.6 g/mL, 6.51 mmol, 3.0 equiv) was added dropwise to a mixture of **A1g** (720 mg, 1.00 mmol, 1.0 equiv) in toluene (20 mL) and *n*-hexane (20 mL) and stirred at room temperature for 24 hours, added $AlCl_3$ (57 mg, 0.43 mmol, 0.20 equiv) and stirred at 75 °C for 8 hours, cooled down to room temperature, added MesMgBr (10.85 mL, 1.0 M in THF, 10.85 mmol, 5.0 equiv) and stirred at room temperature for another 10 hour to afford **BO1g** (eluent: petroleum ether ~ petroleum ether/dichloromethane = 10:1) as a white solid 1.15 g in 96% yield. m.p.: 151.8−152.4 °C. ^1H NMR (500 MHz, $CDCl_3$): δ (ppm) 2.12 (s, 6H), 2.27 (s, 12H), 2.36 (s, 3H), 2.38 (s, 6H), 6.97 (s, 4H), 6.99 (s, 2H), 7.28 (s, 2H), 7.60 (t, J = 7.5 Hz, 1H), 8.18 (d, J = 7.0 Hz, 2H). ^{13}C NMR (125 MHz, $CDCl_3$): δ (ppm) 20.88, 21.04, 21.31, 22.66, 111.34, 114.80, 126.70, 126.98, 127.44, 128.25, 133.90, 135.81, 136.96, 138.07, 138.54, 140.17, 140.24, 141.67, 142.62, 151.38. ^{11}B NMR (160 MHz, $CDCl_3$): δ (ppm) 47.63. HRMS (ESI): calcd for $C_{39}H_{39}[^{11}B]_2O_2^+$ $[M+H]^+$ 561.3131, found 561.3147.

Large scale synthesis of **BO2**:

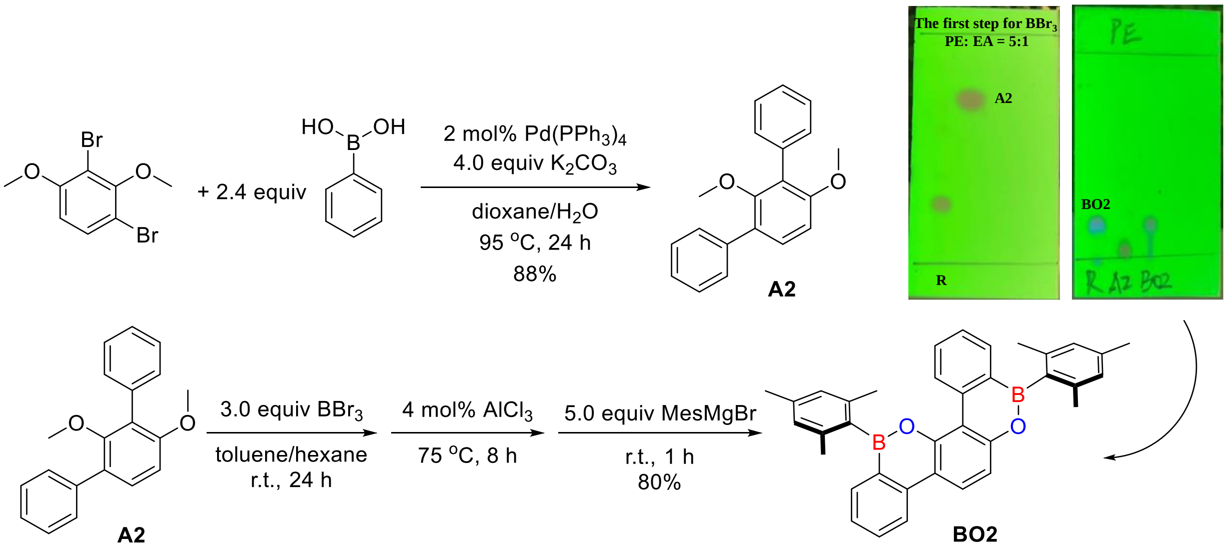

Synthesis of **A2**: Following the general procedure, the reaction of 1,3-dibromo-2,4-dimethoxybenzene (7.37 g, 24.90 mmol, 1.0 equiv), boronic acid (7.29 g, 59.76 mmol, 2.4 equiv), $Pd(PPh_3)_4$ (578 mg, 0.50 mmol, 0.02 equiv) and K_2CO_3 (13.76 g, 99.60 mmol, 4.0 equiv, in 20 mL H_2O) in dioxane (80 mL) at 100 °C for 24 hours afforded **A2** (eluent: petroleum ether/ethyl acetate = 20:1−10:1) as a white solid 6.36 mg in 88% yield. ^1H NMR (500 MHz, $CDCl_3$): δ (ppm) 3.09 (s, 3H), 3.77 (s, 3H), 6.83 (d, J = 8.5 Hz, 1H), 7.29−7.36 (m, 3H), 7.38−7.44 (m, 6H), 7.56−7.58 (m, 2H). ^{13}C NMR (125 MHz, $CDCl_3$): δ (ppm) 55.91, 60.37, 106.94, 125.03, 126.65, 126.91, 127.75, 128.07, 128.15, 129.13, 130.23, 130.62, 134.30, 138.67, 155.92, 157.15.

Synthesis of **BO2**: Following the general procedure, BBr_3 (4.68 mL, d = 2.6 g/mL, 48.57 mmol, 3.0 equiv) was added dropwise to a mixture of **A2** (4.72 g, 16.19 mmol, 1.0 equiv) in toluene (50 mL) and *n*-hexane (50 mL) and stirred at room temperature for 24 hours, added $AlCl_3$ (87 mg, 0.65 mmol, 0.04 equiv) and stirred at 75 °C for 8 hours, cooled down to room temperature, added MesMgBr (80.95 mL, 1.0 M in THF, 80.95 mmol, 5.0 equiv) and stirred at room temperature for another 1 hour to afford **BO2** (eluent: eluent: petroleum ether ~ petroleum ether/dichloromethane = 5:1~ toluene) as a white solid 6.71 g in 80% yield. (Note: **BO2** has a very poor solubility, and toluene was used to dissolve all the product in the column chromatography.) m.p.: 277.1−278.2 °C. ^1H NMR (500 MHz, $CDCl_3$): δ (ppm) 2.22 (s, 6H), 2.27 (s, 6H), 2.38 (s, 3H), 2.43 (s, 3H), 6.95 (s, 2H), 7.00 (s, 2H), 7.42−7.49 (m, 2H), 7.63 (d, J = 9.0 Hz, 1H), 7.71−7.74 (m, 1H), 7.83−7.86 (m, 3H), 8.41 (d, J = 8.0 Hz, 1H), 8.47 (d, J = 9.0 Hz, 1H), 9.61 (d, J = 8.0 Hz, 1H). ^{13}C NMR (125 MHz, $CDCl_3$): δ (ppm) 21.30, 21.36, 22.51, 22.78, 115.02, 116.47, 118.84, 121.78, 123.56, 126.91, 127.04, 127.33, 127.40, 128.00, 128.18, 129.79, 130.37, 133.57, 133.89, 134.27, 137.06, 137.23, 138.21, 138.31, 139.08, 140.18, 140.24, 150.49, 153.25. ^{11}B NMR (160 MHz, $CDCl_3$): δ (ppm) 47.21. HRMS (ESI): calcd for $C_{36}H_{33}[^{11}B]_2O_2^+$ $[M+H]^+$ 519.2661, found 519.2655. The obtained **BO2** was further purified by recrystallization in toluene/methanol; and then 3.4 g **BO2** was sublimated in a seven zone thermal gradient sublimator at 40/100/130/150/250/250/280 °C, 5.0×10^{-5} Torr to obtain white solid 3.0 g in 88% yield; purity, 99.68% by HPLC, HPLC analysis condition: column: Masiall® C18-BlO, 5μm, 250 × 4.6 mm; mobile phase: methanol/tetrahydrofuran = 95/5(v/v); flow rate: 1.0 mL/min; Abs. detector: 254 nm.

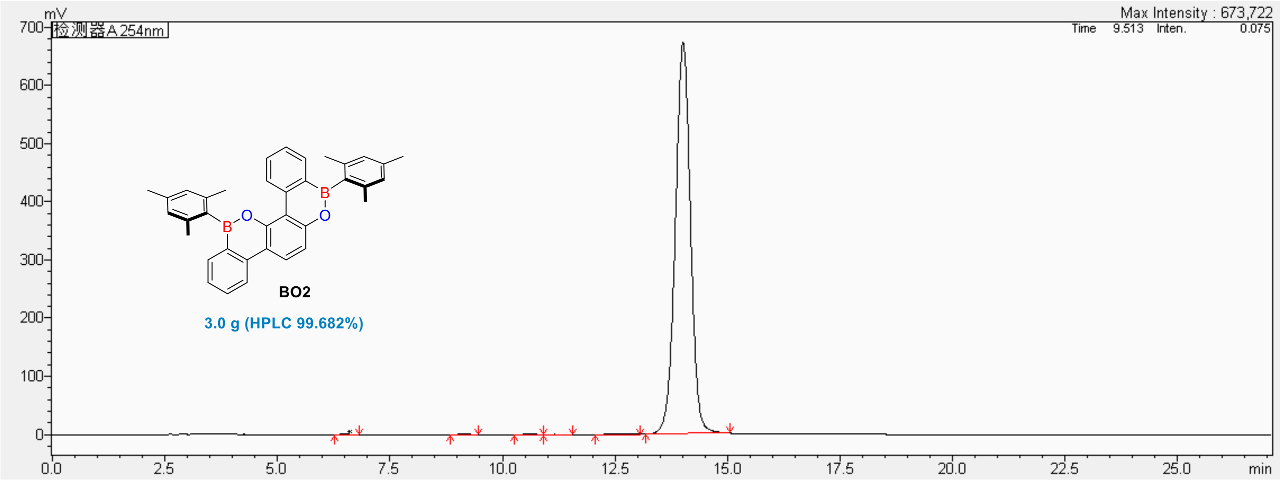

Peak	RetTime /[min]	Area /[mAU*s]	Area /%	Height /[mAU]	Start Time /[min]	End Time/ [min]
1	6.489	1978	0.012	169	6.260	6.820
2	9.147	1803	0.011	114	11.960	9.470
3	10.592	3051	0.019	175	10.270	10.920
4	11.169	1401	0.009	71	10.920	11.560
5	12.659	42189	0.266	1996	12.050	13.050
6	14.013	15814763	99.682	672528	13.190	15.050
Total		15865184	100.000	675054		

Synthesis of **BO3a**:

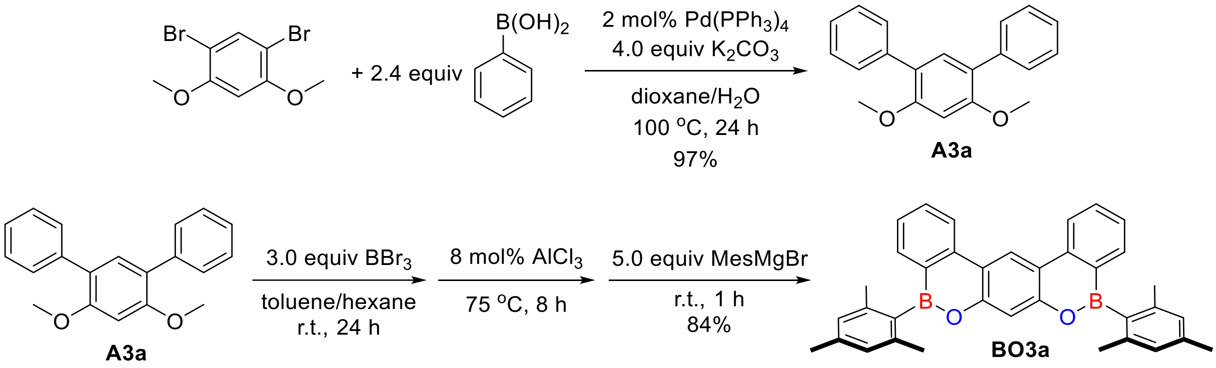

Synthesis of **A3a**: Following the general procedure, the reaction of 1,5-dibromo-2,4-dimethoxybenzene (1.18 g, 4.00 mmol, 1.0 equiv), boronic acid (1.17 g, 9.60 mmol, 2.4 equiv), $Pd(PPh_3)_4$ (92 mg, 0.08 mmol, 0.02 equiv) and K_2CO_3 (2.21 g, 16.00 mmol, 4.0 equiv, in 10 mL H_2O) in dioxane (40 mL) at 100 °C for 24 hours afforded **A3a** (eluent: petroleum ether/ethyl acetate = 10:1–3:1) as a white solid 1.13 g in 97% yield. ^{1}H NMR (500 MHz, $CDCl_3$): δ (ppm) 3.88 (s, 6H), 6.67 (s, 1H), 7.27–7.36 (m, 3H), 7.40 (t, J = 7.5 Hz, 4H), 7.55 (d, J = 7.5 Hz, 4H). ^{13}C NMR (125 MHz, $CDCl_3$): δ (ppm) 55.48, 111.14, 120.75, 127.49, 128.15, 128.49, 130.72, 130.75, 130.96, 138.11, 156.48. The ^{1}H NMR is agreement with the previous reoprt (Ciana, C.-L., Phipps, R. J., Brandt, J. R., Meyer, F.-M., Gaunt, M. J. A highly *para*-selective copper(II)-catalyzed direct arylation of aniline and phenol derivatives. *Angew. Chem. Int. Ed.* **50**, 458–462 (2011).

Synthesis of **BO3a**: Following the general procedure, BBr_3 (0.87 mL, d = 2.6 g/mL, 9.00 mmol,

3.0 equiv) was added dropwise to a mixture of **A3a** (871 mg, 3.00 mmol, 1.0 equiv) in toluene (30 mL) and *n*-hexane (30 mL) and stirred at room temperature for 24 hours, added $AlCl_3$ (32 mg, 0.24 mmol, 0.08 equiv) and stirred at 75 °C for 8 hours, cooled down to room temperature, added MesMgBr (15 mL, 1.0 M in THF, 15 mmol, 5.0 equiv) and stirred at room temperature for another 1 hour to afford **BO3a** (eluent: petroleum ether/dichloromethane = 100:1–10:1) as a white solid 1.30 g in 84% yield. m.p.: 235.5−236.2 °C. ^{1}H NMR (500 MHz, DMSO-d_6): δ (ppm) 2.13 (s, 12H), 2.32 (s, 6H), 6.93 (s, 4H), 7.57 (t, J = 7.5 Hz, 2H), 7.67 (dd, J = 8.0, 1.5 Hz, 2H), 7.76 (s, 1H), 7.97 (td, J = 7.5, 1.5 Hz, 2H), 9.02 (d, J = 8.0 Hz, 2H), 9.58 (s, 1H). ^{13}C NMR (125 MHz, $CDCl_3$): δ (ppm) 21.28, 22.46, 111.29, 118.30, 119.56, 121.31, 127.18, 127.34, 128.88, 133.54, 134.09, 137.63, 138.40, 138.44, 140.06, 152.30. ^{11}B NMR (160 MHz, $CDCl_3$): δ (ppm) 47.36. HRMS (ESI): calcd for $C_{36}H_{33}[^{11}B]_2O_2^+$ [M+H]$^+$ 519.2661, found 519.2671.

Large scale synthesis of **BO3a**:

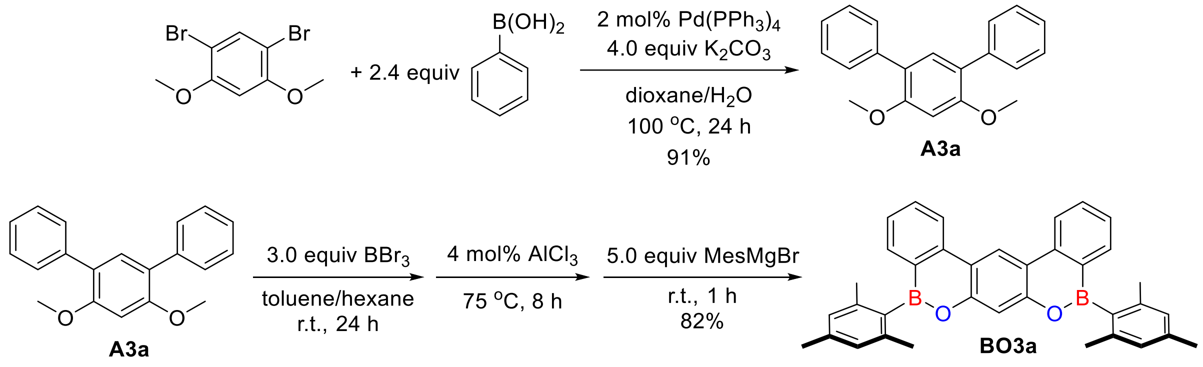

Synthesis of **A3a**: Following the general procedure, the reaction of 1,5-dibromo-2,4-dimethoxybenzene (8.88 g, 30.00 mmol, 1.0 equiv), boronic acid (8.78 g, 72 mmol, 2.4 equiv), $Pd(PPh_3)_4$ (693 mg, 0.60 mmol, 0.02 equiv) and K_2CO_3 (16.58 g, 120.00 mmol, 4.0 equiv, in 20 mL H_2O) in dioxane (80 mL) at 100 °C for 24 hours afforded **A3a** (eluent: petroleum ether/ethyl acetate = 20:1–10:1) as a white solid 7.93 g in 91% yield. The structure was confirmed by ^{1}H NMR, which was in agreement with the above result.

Synthesis of **BO3a**: Following the general procedure, BBr_3 (7.47 mL, d = 2.6 g/mL, 77.49 mmol, 3.0 equiv) was added dropwise to a mixture of **A3a** (7.50 g, 25.83 mmol, 1.0 equiv) in toluene (100 mL) and *n*-hexane (100 mL) and stirred at room temperature for 24 hours, added $AlCl_3$ (137 mg, 1.03 mmol, 0.04 equiv) and stirred at 75 °C for 8 hours, cooled down to room temperature, added MesMgBr (129.15 mL, 1.0 M in THF, 129.15 mmol, 5.0 equiv) and stirred at room temperature for another 1 hour to afford **BO3a** (eluent: petroleum ether/dichloromethane = 100:1–10:1) as a white

solid 10.98 g in 82% yield. The structure was confirmed by ^{1}H NMR, which was in agreement with the above result. The obtained **BO3a** was further purified by recrystallization in toluene/methanol; and then 6.1 g **BO3a** was sublimated in a seven zone thermal gradient sublimator at 40/100/130/150/250/250/280 °C, 5.6×10^{-5} Torr to obtain white solid 5.5 g in 90% yield; purity, 99.94% by HPLC, HPLC analysis condition: column: Masiall® C18-BIO, 5μm, 250 × 4.6 mm; mobile phase: methanol/tetrahydrofuran = 95/5(v/v); flow rate: 1.0 mL/min; Abs. detector: 254 nm.

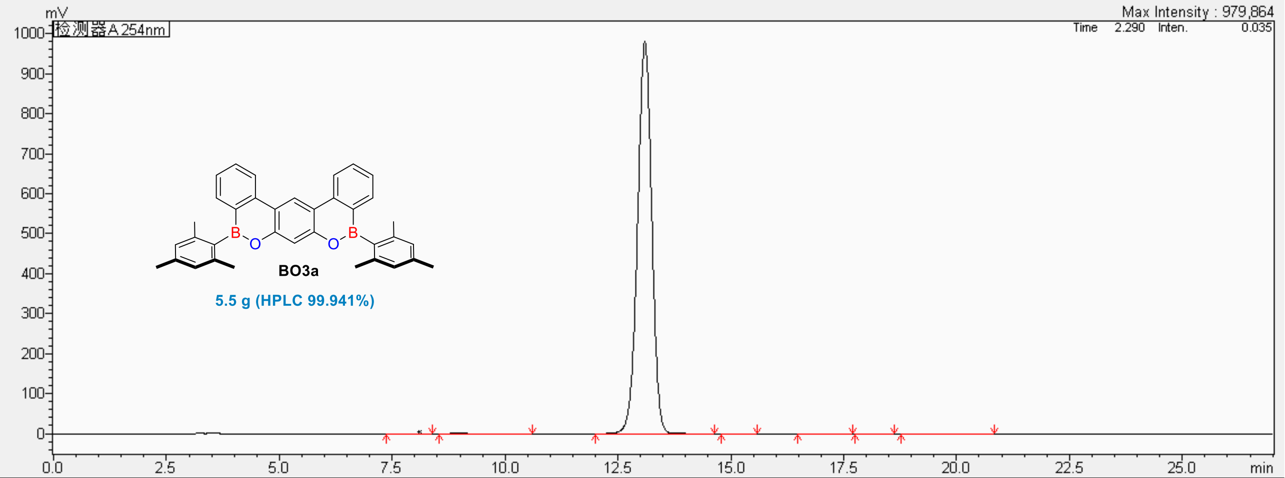

Peak	RetTime /[min]	Area /[mAU*s]	Area /%	Height /[mAU]	Start Time /[min]	End Time/ [min]
1	7.981	1352	0.006	49	7.380	8.380
2	8.929	8646	0.040	273	8.530	10.610
3	13.098	21554842	99.941	979795	11.990	14.640
4	15.040	279	0.001	16	14.790	15.580
5	17.109	795	0.004	34	16.490	17.710
6	18.113	655	0.003	27	17.750	18.610
7	19.379	955	0.004	30	18.770	20.840
Total		21567524	100.000	980225		

Synthesis of **BO3b**:

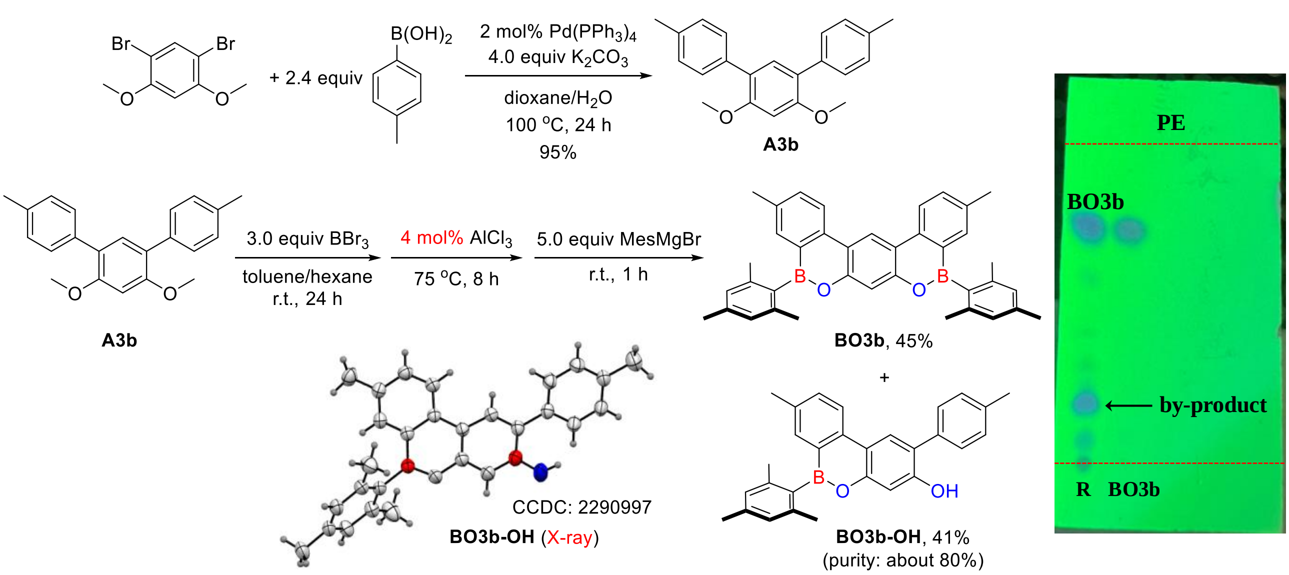

Synthesis of **A3b**: Following the general procedure, the reaction of 1,5-dibromo-2,4-

dimethoxybenzene (4.44 g, 15.00 mmol, 1.0 equiv), 4-methylboronic acid (4.89 g, 36.00 mmol, 2.4 equiv), $Pd(PPh_3)_4$ (347 mg, 0.30 mmol, 0.02 equiv) and K_2CO_3 (8.29 g, 60.00 mmol, 4.0 equiv, in 15 mL H_2O) in dioxane (60 mL) at 100 °C for 24 hours afforded **A3b** (eluent: petroleum ether/ethyl acetate = 20:1–10:1) as a white solid 4.52 g in 95% yield. ^{1}H NMR (500 MHz, $CDCl_3$): δ (ppm) 2.40 (s, 6H), 3.89 (s, 6H), 6.67 (s, 1H), 7.23 (d, J = 8.0 Hz, 4H), 7.31 (s, 1H), 7.44−7.46 (m, 4H). ^{13}C NMR (125 MHz, $CDCl_3$): δ (ppm) 21.13, 55.81, 96.23, 123.13, 128.73, 129.28, 132.66, 135.21, 136.15, 156.58.

Synthesis of **BO3b**: Following the general procedure, BBr_3 (4.10 mL, d = 2.6 g/mL, 42.60 mmol, 3.0 equiv) was added dropwise to a mixture of **A3b** (4.52 g, 14.20 mmol, 1.0 equiv) in toluene (50 mL) and *n*-hexane (50 mL) and stirred at room temperature for 24 hours, added $AlCl_3$ (76 mg, 0.57 mmol, 0.04 equiv) and stirred at 75 °C for 8 hours, cooled down to room temperature, added MesMgBr (71 mL, 1.0 M in THF, 71 mmol, 5.0 equiv) and stirred at room temperature for another 1 hour to afford **BO3b** (eluent: petroleum ether/dichloromethane = 100:1–10:1) as a white solid 3.52 g in 45% yield; and a by-product **BO3b-OH** as a white solid 3.22 g in 41% yield, which was not very pure, the purity was about 80% on the basis of ^{1}H NMR spectrum. **BO3b**: m.p.: 243.2−244.0 °C. ^{1}H NMR (500 MHz, $CDCl_3$): δ (ppm) 2.22 (s, 12H), 2.37 (s, 6H), 2.43 (s, 6H), 6.95 (s, 4H), 7.62 (s, 2H), 7.66 (s, 1H), 7.67 (dd, J = 8.0, 1.5 Hz, 2H), 8.42 (d, J = 8.0 Hz, 2H), 9.17 (s, 1H). ^{13}C NMR (125 MHz, $CDCl_3$): δ (ppm) 21.19, 21.30, 22.47, 111.05, 117.68, 119.58, 121.34, 127.32, 128.87, 134.37, 134.77, 135.99, 136.92, 137.42, 138.33, 140.12, 151.74. ^{11}B NMR (160 MHz, $CDCl_3$): δ (ppm) 46.36. HRMS (ESI): calcd for $C_{38}H_{37}[^{11}B]_2O_2^+$ [M+H]$^+$ 547.2974, found 547.2990. **BO3b-OH**: ^{1}H NMR (500 MHz, DMSO-d_6) δ 2.09 (s, 6H), 2.31 (s, 3H), 2.32 (s, 3H), 2.37 (s, 3H), 6.90 (s, 2H), 7.07 (s, 1H), 7.26 (d, J = 8.0 Hz, 2H), 7.37 (s, 1H), 7.54–7.57 (m, 2H), 7.61 (dd, J = 8.0, 2.0 Hz, 1H), 8.22 (s, 1H), 8.33 (d, J = 8.5 Hz, 1H), 10.17 (s, 1H). HRMS (ESI): calcd for $C_{29}H_{28}{}^{11}BO_2^+$ [M+H]$^+$ 419.2177, found 419.2172.

Synthesis of **BO3b** from **BO3h-OH**:

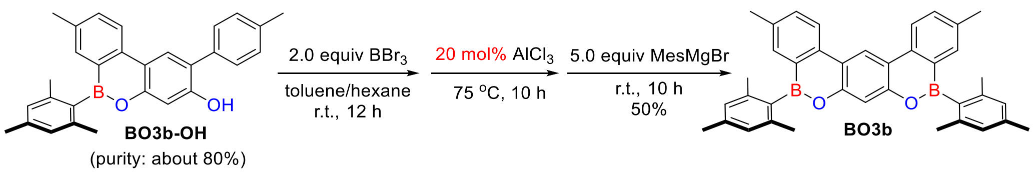

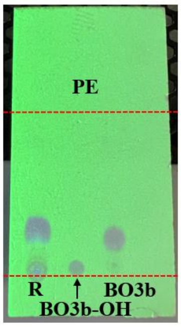

Synthesis of **BO3b**: Following the general procedure, BBr_3 (0.19 mL, d = 2.6 g/mL, 2.0 mmol, 2.0 equiv) was added dropwise to a mixture of **BO3b-OH** (418 mg, 1.0 mmol, 1.0 equiv) in toluene (5 mL) and *n*-hexane (5 mL) and the mixture was stirred at room temperature for 12 hours, then $AlCl_3$ (26 mg, 0.20 mmol, 0.2 equiv) was added and the mixture was stirred at 75 °C for 10 hours, cooled down to room temperature; then MesMgBr (5 mL, 1.0 M in THF, 5.00 mmol, 5.0 equiv) was added

and the mixture was stirred at room temperature for another 10 hour to afford **BO3b** (eluent: petroleum ether ~ petroleum ether/dichloromethane = 100:1–10:1) as a white solid 273 mg in 50% yield. The structure was confirmed by ^{1}H NMR, which was in agreement with the above result.

Improved synthesis of **BO3b**:

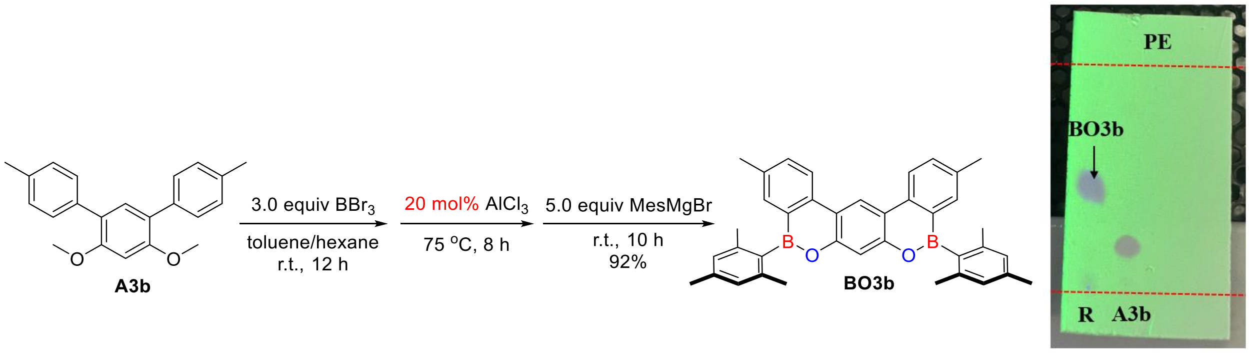

Synthesis of **BO3b**: Following the general procedure, BBr_3 (0.91 mL, d = 2.6 g/mL, 9.42 mmol, 3.0 equiv) was added dropwise to a mixture of **A3b** (1.00 g, 3.14 mmol, 1.0 equiv) in toluene (10 mL) and *n*-hexane (10 mL) and the mixture was stirred at room temperature for 12 hours; then $AlCl_3$ (84 mg, 0.63 mmol, 0.20 equiv) was added and the mixture was stirred at 75 °C for 8 hours, cooled down to room temperature; MesMgBr (15.7 mL, 1.0 M in THF, 15.70 mmol, 5.0 equiv) was added and the mixture was stirred at room temperature for another 10 hour to afford **BO3b** (eluent: petroleum ether ~ petroleum ether/dichloromethane = 100:1–10:1) as a white solid 1.59 g in 92% yield. The structure was confirmed by ^{1}H NMR, which was in agreement with the above result.

Synthesis of **BO3c**:

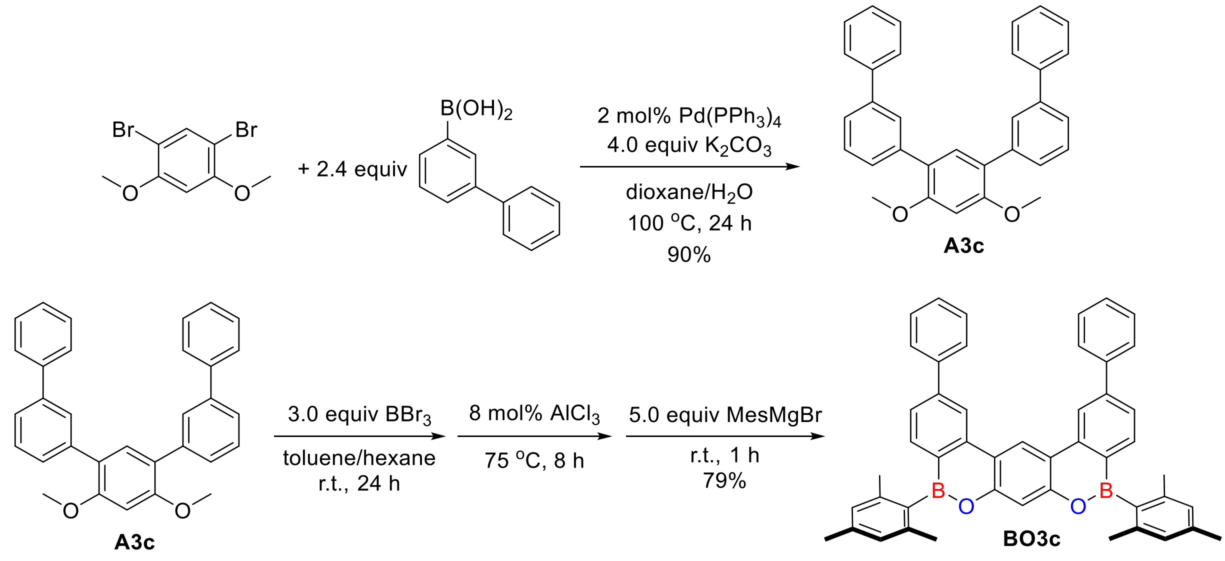

Synthesis of **A3c**: Following the general procedure, the reaction of 1,5-dibromo-2,4-

dimethoxybenzene (1.18 g, 4.00 mmol, 1.0 equiv), [1,1'-biphenyl]-3-ylboronic acid (1.90 g, 9.60 mmol, 2.4 equiv), $Pd(PPh_3)_4$ (92 mg, 0.08 mmol, 0.02 equiv) and K_2CO_3 (2.21 g, 16.00 mmol, 4.0 equiv, in 10 mL H_2O) in dioxane (40 mL) at 100 °C for 24 hours afforded **A3c** (eluent: petroleum ether/ethyl acetate = 10:1–3:1) as a white solid 1.59 g in 90% yield. ^{1}H NMR (500 MHz, DMSO-d_6): δ (ppm) 3.90 (s, 6H), 6.91 (s, 1H), 7.36 (t, J = 7.5 Hz, 2H), 7.40 (s, 1H), 7.43−7.55 (m, 8H), 7.57 (d, J = 7.5, 2H), 7.69 (d, J = 7.5, 4H), 7.75 (s, 2H).

Synthesis of **BO3c**: Following the general procedure, BBr_3 (0.87 mL, d = 2.6 g/mL, 9.00 mmol, 3.0 equiv) was added dropwise to a mixture of **A3c** (1.33 g, 3.00 mmol, 1.0 equiv) in toluene (30 mL) and *n*-hexane (30 mL) and stirred at room temperature for 24 hours, added $AlCl_3$ (32 mg, 0.24 mmol, 0.08 equiv) and stirred at 75 °C for 8 hours, cooled down to room temperature, added MesMgBr (15 mL, 1.0 M in THF, 15 mmol, 5.0 equiv) and stirred at room temperature for another 1 hour to afford **BO3c** (eluent: petroleum ether/dichloromethane = 100:1–10:1) as a white solid 1.59 g in 79% yield. m.p.: 220.9−221.2 °C. ^{1}H NMR (500 MHz, DMSO-d_6): δ (ppm) 2.16 (s, 12H), 2.33 (s, 6H), 6.94 (s, 4H), 7.49−7.52 (m, 2H), 7.59−7.62 (m, 4H), 7.74 (d, J = 8.0 Hz, 2H), 7.79 (s, 1H), 7.81 (dd, J = 8.0, 1.5 Hz, 2H), 7.95−7.97 (m, 4H), 9.23 (s, 2H), 9.83 (s, 1H). ^{13}C NMR (125 MHz, $CDCl_3$): δ (ppm) 21.30, 22.52, 111.59, 118.22, 119.67, 120.07, 126.66, 127.38, 127.64, 127.79, 128.10, 129.02, 134.11, 138.16, 138.43, 138.88, 140.10, 141.23, 146.68, 152.66. ^{11}B NMR (160 MHz, $CDCl_3$): δ (ppm) 47.62. HRMS (ESI): calcd for $C_{48}H_{41}[^{11}B]_2O_2^+$ $[M+H]^+$ 671.3287, found 671.3283.

Synthesis of **BO3d**:

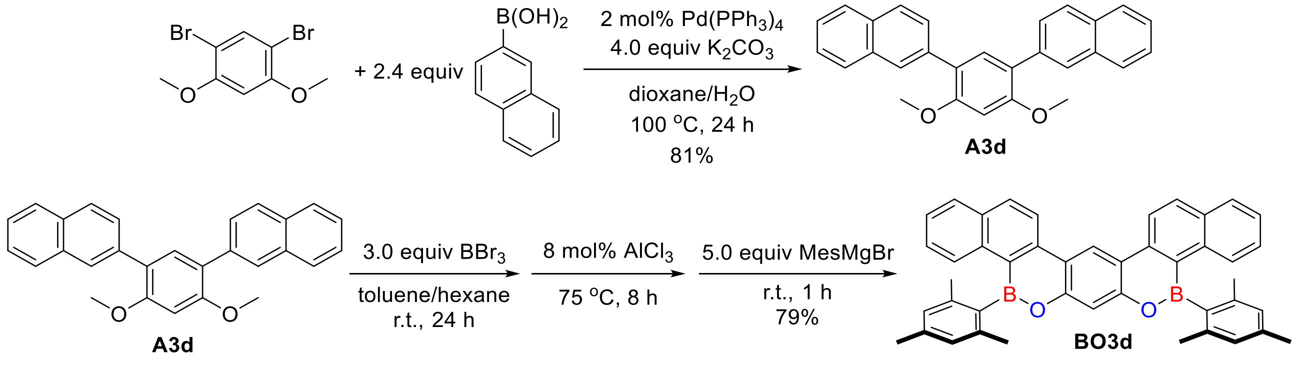

Synthesis of **A3d**: Following the general procedure, the reaction of 1,5-dibromo-2,4-dimethoxybenzene (1.18 g, 4.00 mmol, 1.0 equiv), naphthalen-2-ylboronic acid (1.65 g, 9.60 mmol, 2.4 equiv), $Pd(PPh_3)_4$ (92 mg, 0.08 mmol, 0.02 equiv) and K_2CO_3 (2.21 g, 16.00 mmol, 4.0 equiv, in 20 mL H_2O) in dioxane (60 mL) at 100 °C for 24 hours afforded **A3d** (eluent: petroleum ether/ethyl

acetate = 10:1–3:1) as a white solid 1.26 g in 81% yield. ^{1}H NMR (500 MHz, $CDCl_3$): δ (ppm) 3.91 (s, 6H), 6.73 (s, 1H), 7.43−7.54 (m, 4H), 7.54 (s, 1H), 7.74 (dd, J = 8.5, 1.0 Hz, 2H), 7.83−7.87 (m, 6H), 7.99 (s, 2H). ^{13}C NMR (125 MHz, $CDCl_3$): δ (ppm) 56.00, 96.54, 123.41, 125.62, 125.87, 127.24, 127.55, 127.87, 128.03, 128.13, 132.29, 133.41, 133.52, 135.81, 157.18.

Synthesis of **BO3d**: Following the general procedure, BBr_3 (0.87 mL, d = 2.6 g/mL, 9.00 mmol, 3.0 equiv) was added dropwise to a mixture of **A3d** (1.17 g, 3.00 mmol, 1.0 equiv) in toluene (40 mL) and *n*-hexane (40 mL) and stirred at room temperature for 24 hours, added $AlCl_3$ (32 mg, 0.24 mmol, 0.08 equiv) and stirred at 75 °C for 8 hours, cooled down to room temperature, added MesMgBr (15 mL, 1.0 M in THF, 15 mmol, 5.0 equiv) and stirred at room temperature for another 1 hour to afford **BO3d** (eluent: petroleum ether/dichloromethane = 100:1–10:1) as a white solid 1.47 g in 79% yield. ^{1}H NMR (500 MHz, $CDCl_3$): δ (ppm) 2.19 (s, 12H), 2.42 (s, 6H), 7.00 (s, 4H), 7.32−7.35 (m, 2H), 7.49−7.52 (m, 2H), 7.77 (s, 1H), 7.95 (d, J = 8.0 Hz, 2H), 8.25 (d, J = 8.5 Hz, 2H), 8.34 (d, J = 8.5 Hz, 2H), 8.75 (d, J = 9.0 Hz, 2H), 9.53 (s, 1H). ^{13}C NMR (125 MHz, $CDCl_3$): δ (ppm) 21.40, 22.08, 110.08, 119.72, 119.95, 120.00, 125.49, 126.10, 127.09, 127.75, 127.88, 128.59, 132.36, 134.87, 137.88, 138.29, 138.86, 139.18, 139.68, 152.47. ^{11}B NMR (160 MHz, $CDCl_3$): δ (ppm) 45.48. HRMS (ESI): calcd for $C_{44}H_{36}[^{11}B]_2NaO_2^+$ $[M+Na]^+$ 641.2794, found 641.2783.

Synthesis of **BO3e**:

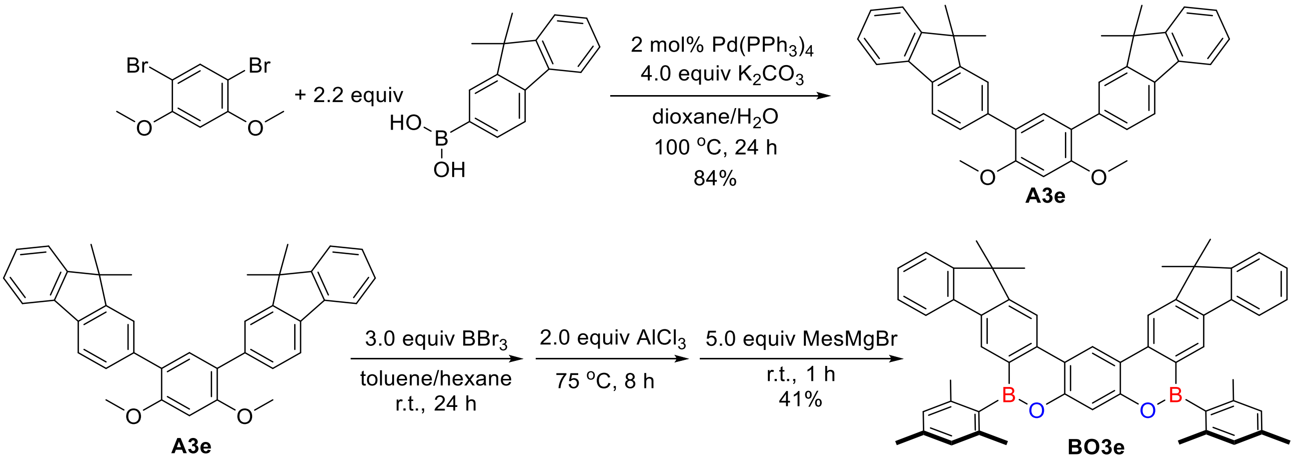

Synthesis of **A3e**: Following the general procedure, the reaction of 1,5-dibromo-2,4-dimethoxybenzene (1.26 g, 4.26 mmol, 1.0 equiv), (9,9-dimethyl-9*H*-fluoren-2-yl) boronic acid (2.23 g, 9.37 mmol, 2.2 equiv), $Pd(PPh_3)_4$ (100 mg, 0.087 mmol, 0.02 equiv) and K_2CO_3 (2.35 g, 17.03 mmol, 4.0 equiv, in 20 mL H_2O) in dioxane (60 mL) at 100 °C for 24 hours afforded **A3e** (eluent:

petroleum ether/ethyl acetate = 10:1–3:1) as a white solid 1.88 g in 84% yield. ^{1}H NMR (500 MHz, $CDCl_3$): δ (ppm) 1.52 (s, 12H), 3.92 (s, 6H), 6.71 (s, 1H), 7.29−7.35 (m, 4H), 7.43 (dd, J = 7.5, 1.0 Hz, 2H), 7.46 (s, 1H), 7.57 (dd, J = 8.0, 1.5 Hz, 2H), 7.62 (d, J = 1.0 Hz, 2H), 7.73−7.77 (m, 4H). ^{13}C NMR (125 MHz, $CDCl_3$): δ (ppm) 55.91, 60.37, 106.94, 125.03, 126.65, 126.91, 127.75, 128.07, 128.15, 129.13, 130.23, 130.62, 134.30, 138.67, 155.92, 157.15. HRMS (ESI): calcd for $C_{38}H_{35}O_2^+$ $[M+H]^+$ 523.2632, found 523.2644.

Synthesis of **BO3e**: Following the general procedure, BBr_3 (0.29 mL, d = 2.6 g/mL, 3.00 mmol, 3.0 equiv) was added dropwise to a mixture of **A3e** (523 mg, 1.0 mmol, 1.0 equiv) in toluene (20 mL) and *n*-hexane (20 mL) and stirred at room temperature for 24 hours, added $AlCl_3$ (267 mg, 2.0 mmol, 2.0 equiv) and stirred at 75 °C for 8 hours, cooled down to room temperature, added MesMgBr (5 mL, 1.0 M in THF, 5.0 mmol, 5.0 equiv) and stirred at room temperature for another 1 hour to afford **BO3e** (eluent: petroleum ether/dichloromethane = 100:1–10:1) as a yellow solid 306 mg in 41% yield. ^{1}H NMR (500 MHz, $CDCl_3$): δ (ppm) 1.75 (s, 12H), 2.27 (s, 12H), 2.41 (s, 6H), 7.02 (s, 4H), 7.33−7.38 (m, 4H), 7.52 (d, J = 5.5 Hz, 2H), 7.71 (s, 1H), 7.77 (dd, J = 5.5, 1.0 Hz, 2H), 8.15 (s, 2H), 8.55 (s, 2H), 9.31 (s, 1H). ^{13}C NMR (125 MHz, $CDCl_3$): δ (ppm) 21.35, 22.59, 27.59, 47.51, 111.34, 115.40, 117.90, 120.01, 120.71, 122.64, 127.23, 127.43, 127.83, 128.36, 128.73, 134.49, 138.01, 138.35, 138.38, 138.91, 140.25, 152.19, 153.45, 159.44. ^{11}B NMR (160 MHz, $CDCl_3$): δ (ppm) 45.52. HRMS (ESI): calcd for $C_{54}H_{48}[^{11}B]_2NaO_2^+$ $[M+Na]^+$ 773.3733, found 773.3720.

Synthesis of **BO4a**:

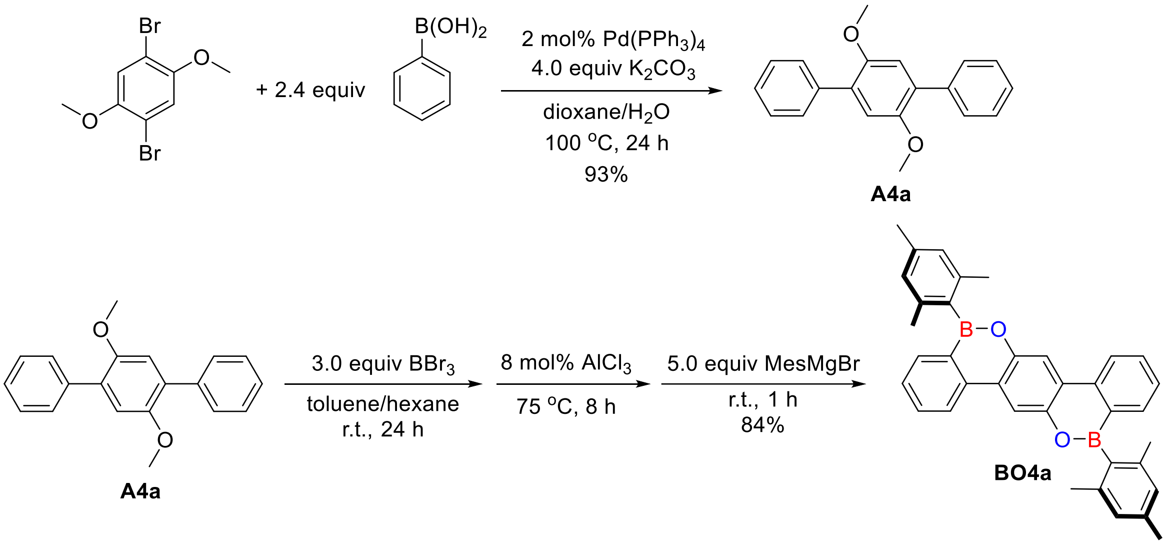

Synthesis of **A4a**: Following the general procedure, the reaction of 1,4-dibromo-2,5-dimethoxybenzene (2.37 g, 8.00 mmol, 1.0 equiv), boronic acid (2.34 g, 19.20 mmol, 2.4 equiv), $Pd(PPh_3)_4$ (185 mg, 0.16 mmol, 0.02 equiv) and K_2CO_3 (4.42 g, 32.00 mmol, 4.0 equiv, in 20 mL H_2O)

in dioxane (80 mL) at 100 °C for 24 hours afforded **A4a** (eluent: petroleum ether/ethyl acetate = 10:1–5:1) as a white solid 2.16 g in 93% yield. ^{1}H NMR (500 MHz, DMSO-*d*$_6$): δ (ppm) 3.76 (s, 6H), 7.03 (s, 2H), 7.34 (t, *J* = 7.5 Hz, 2H), 7.43 (t, *J* = 7.5 Hz, 4H), 7.56 (d, *J* = 8.0, 4H). ^{13}C NMR (125 MHz, $CDCl_3$): δ (ppm) 56.35, 114.73, 127.07, 128.05, 129.41, 130.36, 138.28, 150.59. The ^{13}C NMR is agreement with the previous reoprt (Bolligera, J. L., Frech, C. M. Polyaromatic the 1,3-diaminobenzene-derived aminophosphine palladium pincer complex {C_6H_3ACHTUNGTRENUNG[NHP(piperidinyl)$_2$]$_2$Pd(Cl)}–a highly active Suzuki–Miyaura catalyst with excellent functional group tolerance. *Adv. Synth. Catal.* **352**, 1075–1080 (2010).

Synthesis of **BO4a**: Following the general procedure, BBr_3 (1.16 mL, d = 2.6 g/mL, 12.00 mmol, 3.0 equiv) was added dropwise to a mixture of **A4a** (1.16 g, 4.00 mmol, 1.0 equiv) in toluene (30 mL) and *n*-hexane (30 mL) and stirred at room temperature for 24 hours, added $AlCl_3$ (42 mg, 0.32 mmol, 0.08 equiv) and stirred at 75 °C for 8 hours, cooled down to room temperature, added MesMgBr (20 mL, 1.0 M in THF, 20 mmol, 5.0 equiv) and stirred at room temperature for another 1 hour to afford **BO4a** (eluent: petroleum ether/dichloromethane = 100:1–10:1) as a white solid 1.74 g in 84% yield. m.p.: >350 °C. ^{1}H NMR (500 MHz, $CDCl_3$): δ (ppm) 2.24 (s, 12H), 2.39 (s, 6H), 6.96 (s, 4H), 7.49 (t, *J* = 7.5 Hz, 2H), 7.81−7.84 (m, 4H), 8.34 (d, *J* = 8.0 Hz, 2H), 8.45 (s, 2H). ^{13}C NMR (125 MHz, $CDCl_3$): δ (ppm) 21.32, 22.47, 114.38, 121.99, 124.13, 127.35, 127.87, 129.33, 133.52, 137.44, 137.94, 138.37, 140.11, 141.79. ^{11}B NMR (160 MHz, $CDCl_3$): δ (ppm) 44.68. HRMS (ESI): calcd for $C_{36}H_{33}[^{11}B]_2O_2^+$ $[M+H]^+$ 519.2661, found 519.2672.

Synthesis of **BO4b**:

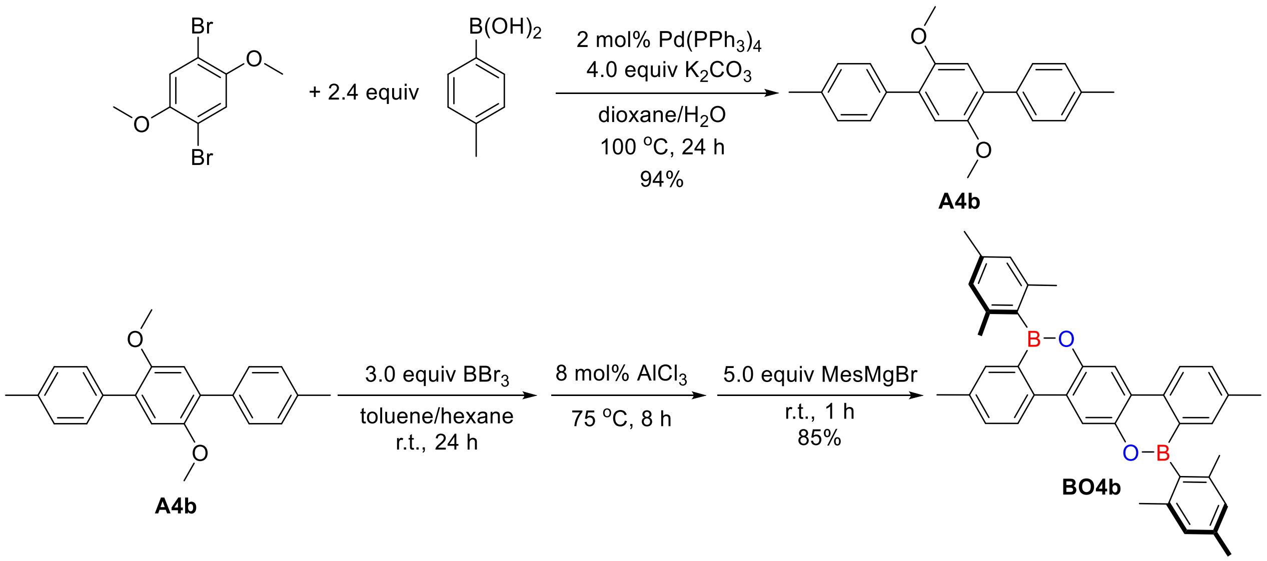

Synthesis of **A4b**: Following the general procedure, the reaction of 1,4-dibromo-2,5-dimethoxybenzene (2.37 g, 8.00 mmol, 1.0 equiv), 4-methylboronic acid (2.61 g, 19.20 mmol, 2.4

equiv), $Pd(PPh_3)_4$ (185 mg, 0.16 mmol, 0.02 equiv) and K_2CO_3 (4.42 g, 32.00 mmol, 4.0 equiv, in 20 mL H_2O) in dioxane (80 mL) at 100 °C for 24 hours afforded **A4b** (eluent: petroleum ether/ethyl acetate = 10:1–5:1) as a white solid 2.40 g in 94% yield. ^1H NMR (500 MHz, DMSO-d_6): δ (ppm) 2.35 (s, 6H), 3.74 (s, 6H), 6.98 (s, 2H), 7.23 (d, J = 8.0 Hz, 4H), 7.45 (d, J = 8.0 Hz, 4H). ^{13}C NMR (125 MHz, $CDCl_3$): δ (ppm) 21.19, 56.35, 114.60, 128.81, 129.28, 130.11, 135.40, 136.78, 150.61.

Synthesis of **BO4b**: Following the general procedure, BBr_3 (1.16 mL, d = 2.6 g/mL, 12.00 mmol, 3.0 equiv) was added dropwise to a mixture of **A4b** (1.27 g, 4.00 mmol, 1.0 equiv) in toluene (30 mL) and n-hexane (30 mL) and stirred at room temperature for 24 hours, added $AlCl_3$ (42 mg, 0.32 mmol, 0.08 equiv) and stirred at 75 °C for 8 hours, cooled down to room temperature, added MesMgBr (20 mL, 1.0 M in THF, 20 mmol, 5.0 equiv) and stirred at room temperature for another 1 hour to afford **BO4b** (eluent: petroleum ether/dichloromethane = 100:1–10:1) as a white solid 1.86 g in 85% yield. ^1H NMR (500 MHz, $CDCl_3$): δ (ppm) 2.23 (s, 12H), 2.39 (s, 6H), 2.41 (s, 6H), 6.97 (s, 4H), 7.62−7.64 (m, 4H), 8.22 (d, J = 8.0 Hz, 2H), 8.38 (s, 2H). ^{13}C NMR (125 MHz, $CDCl_3$): δ (ppm) 21.29, 21.33, 22.47, 113.98, 121.96, 123.83, 127.33, 129.32, 134.77, 135.55, 137.27, 137.69, 138.27, 140.15, 147.58. ^{11}B NMR (160 MHz, $CDCl_3$): δ (ppm) 46.97. HRMS (ESI): calcd for $C_{38}H_{37}[^{11}B]_2O_2^+$ $[M+H]^+$ 547.2974, found 547.2992.

Synthesis of **BO4c**:

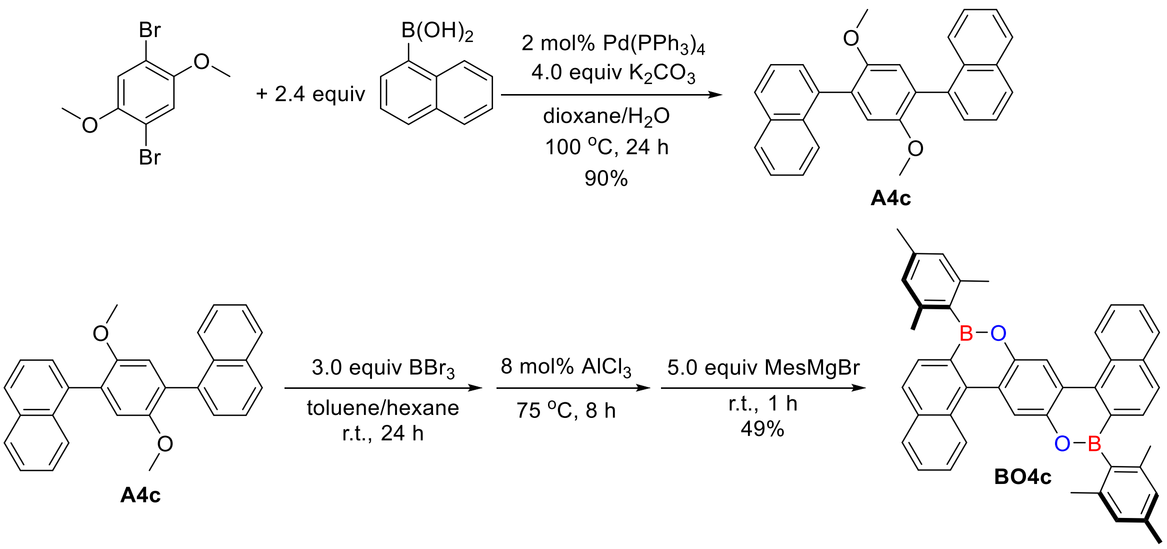

Synthesis of **A4c**: Following the general procedure, the reaction of 1,4-dibromo-2,5-dimethoxybenzene (1.18 g, 4.00 mmol, 1.0 equiv), naphthalen-1-ylboronic acid (1.66 g, 9.60 mmol, 2.4 equiv), $Pd(PPh_3)_4$ (92 mg, 0.08 mmol, 0.02 equiv) and K_2CO_3 (2.21 g, 16.00 mmol, 4.0 equiv, in 20 mL H_2O) in dioxane (60 mL) at 100 °C for 24 hours afforded **A4c** (eluent: petroleum ether/ethyl acetate = 10:1–3:1) as a white solid 1.41 g in 90% yield. ^1H NMR (500 MHz, $CDCl_3$): δ (ppm) 3.63

(s, 6H), 7.01 (d, J = 4.0 Hz, 2H), 7.46−7.60 (m, 8H), 7.76−7.81 (m, 2H), 7.90−7.94 (m, 4H). ^{13}C NMR (125 MHz, $CDCl_3$): δ (ppm) 55.91, 60.37, 106.94, 125.03, 126.65, 126.91, 127.75, 128.07, 128.15, 129.13, 130.23, 130.62, 134.30, 138.67, 155.92, 157.15. HRMS (ESI): calcd for $C_{28}H_{26}NO_2^+$ $[M+NH_4]^+$ 408.1958, found 408.1976.

Synthesis of **BO4c**: Following the general procedure, BBr_3 (0.58 mL, d = 2.6 g/mL, 6.00 mmol, 3.0 equiv) was added dropwise to a mixture of **A4c** (781 mg, 2.00 mmol, 1.0 equiv) in toluene (30 mL) and *n*-hexane (30 mL) and stirred at room temperature for 24 hours, added $AlCl_3$ (22 mg, 0.16 mmol, 0.08 equiv) and stirred at 75 °C for 8 hours, cooled down to room temperature, added MesMgBr (10 mL, 1.0 M in THF, 10 mmol, 5.0 equiv) and stirred at room temperature for another 1 hour to afford **BO4c** (eluent: petroleum ether/dichloromethane = 100:1–10:1) as a white solid 610 mg in 49% yield. ^{1}H NMR (500 MHz, $CDCl_3$): δ (ppm) 2.22 (s, 12H), 2.38 (s, 6H), 6.97 (s, 4H), 7.69−7.74 (m, 4H), 7.77 (d, J = 8.0 Hz, 2H), 7.88 (d, J = 8.5 Hz, 2H), 8.00−8.02 (m, 2H), 8.97 (s, 2H), 9.15 (d, J = 7.0 Hz, 2H). ^{13}C NMR (125 MHz, $CDCl_3$): δ (ppm) 21.32, 22.52, 119.87, 124.09, 126.88, 127.44, 127.94, 128.21, 128.42, 128.91, 129.02, 129.18, 131.29, 134.31, 137.09, 137.88, 138.52, 140.17, 148.42. ^{11}B NMR (160 MHz, $CDCl_3$): δ (ppm) 45.44. HRMS (ESI): calcd for $C_{44}H_{36}[^{11}B]_2NaO_2^+$ $[M+Na]^+$ 641.2794, found 641.2780.

Synthesis of **BO4d**:

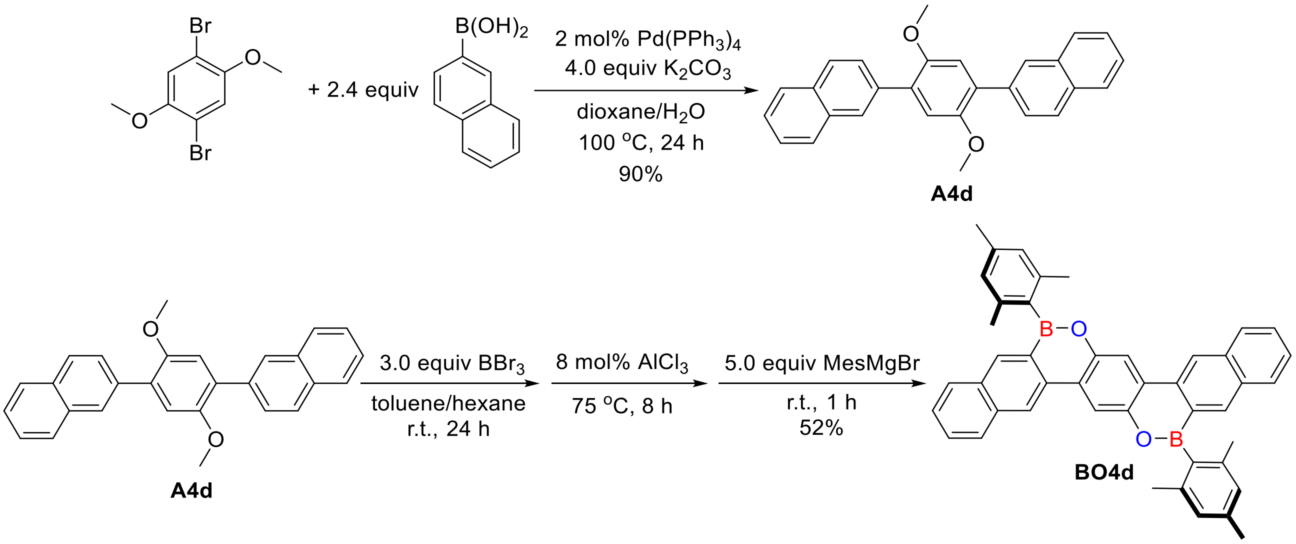

Synthesis of **A4d**: Following the general procedure, the reaction of 1,4-dibromo-2,5-dimethoxybenzene (1.18 g, 4.00 mmol, 1.0 equiv), naphthalen-2-ylboronic acid (1.65 g, 9.60 mmol, 2.4 equiv), $Pd(PPh_3)_4$ (92 mg, 0.08 mmol, 0.02 equiv) and K_2CO_3 (2.21 g, 16.00 mmol, 4.0 equiv, in 20 mL H_2O) in dioxane (60 mL) at 100 °C for 24 hours afforded **A4d** (eluent: petroleum ether/ethyl acetate = 10:1–3:1) as a white solid 1.40 g in 90% yield. ^{1}H NMR (500 MHz, $CDCl_3$): δ (ppm) 3.84

(s, 6H), 7.13 (s, 2H), 7.48−7.53 (m, 4H), 7.37 (dd, J = 8.5, 1.0 Hz, 2H), 7.87−7.92 (m, 6H), 8.05 (d, J = 0.5 Hz, 2H). ^{13}C NMR (125 MHz, $CDCl_3$): δ (ppm) 56.62, 115.20, 125.92, 126.03, 127.39, 127.63, 127.92, 128.06, 128.14, 130.63, 132.56, 133.45, 135.99, 151.02. HRMS (ESI): calcd for $C_{28}H_{26}NO_2^+$ $[M+NH_4]^+$ 408.1958, found 408.1972.

Synthesis of **BO4d**: Following the general procedure, BBr_3 (0.29 mL, d = 2.6 g/mL, 3.00 mmol, 3.0 equiv) was added dropwise to a mixture of **A4d** (390 mg, 1.00 mmol, 1.0 equiv) in toluene (20 mL) and *n*-hexane (20 mL) and stirred at room temperature for 24 hours, added $AlCl_3$ (12 mg, 0.08 mmol, 0.08 equiv) and stirred at 75 °C for 8 hours, cooled down to room temperature, added MesMgBr (5 mL, 1.0 M in THF, 5 mmol, 5.0 equiv) and stirred at room temperature for another 1 hour to afford **BO4d** (eluent: petroleum ether/dichloromethane = 100:1–10:1) as a white solid 322 mg in 52% yield. ^{1}H NMR (500 MHz, $CDCl_3$): δ (ppm) 2.29 (s, 12H), 2.43 (s, 6H), 7.02 (s, 4H), 7.53 (t, J = 7.0 Hz, 2H), 7.64 (d, J = 7.0 Hz, 2H), 7.93 (d, J = 8.0 Hz, 2H), 8.05 (d, J = 8.5 Hz, 2H), 8.41 (s, 2H), 8.58 (s, 2H), 8.76 (s, 2H). ^{13}C NMR (125 MHz, $CDCl_3$): δ (ppm) 21.39, 22.54, 114.82, 120.79, 124.37, 126.28, 127.40, 128.37, 128.46, 128.99, 132.64, 133.40, 136.09, 138.41, 139.86, 140.25, 147.80. ^{11}B NMR (160 MHz, $CDCl_3$): δ (ppm) 46.76. HRMS (ESI): calcd for $C_{44}H_{36}[^{11}B]_2O_2^-$ $[M]^-$ 618.2907, found 618.2988.

Synthesis of **BO4e**:

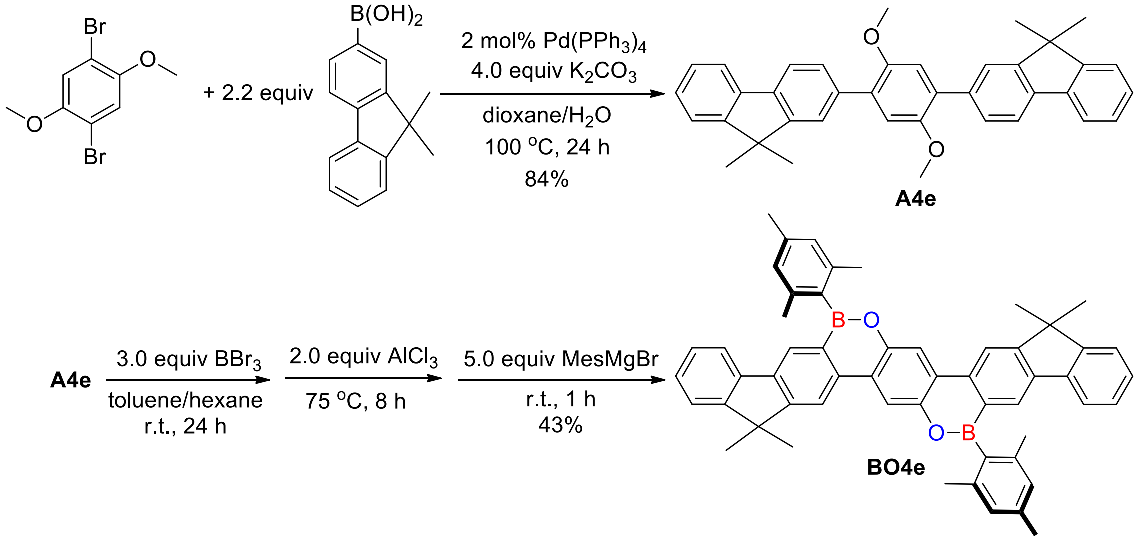

Synthesis of **A4e**: Following the general procedure, the reaction of 1,4-dibromo-2,5-dimethoxybenzene (1.26 g, 4.26 mmol, 1.0 equiv), (9,9-dimethyl-9*H*-fluoren-2-yl) boronic acid (2.23 g, 9.37 mmol, 2.2 equiv), $Pd(PPh_3)_4$ (98 mg, 0.085 mmol, 0.02 equiv) and K_2CO_3 (2.36 g, 17.04 mmol, 4.0 equiv, in 20 mL H_2O) in dioxane (60 mL) at 100 °C for 24 hours afforded **A4e** (eluent: petroleum ether/ethyl acetate = 10:1–3:1) as a white solid 1.87 g in 84% yield. ^{1}H NMR (500 MHz, $CDCl_3$): δ

(ppm) 1.24 (s, 12H), 3.85 (s, 6H), 7.08 (s, 2H), 7.31−7.37 (m, 4H), 7.46 (dd, J = 6.5, 1.5 Hz, 2H), 7.59 (dd, J = 8.0, 1.5 Hz, 2H), 7.68 (d, J = 1.0 Hz, 2H), 7.76 (dd, J = 6.5, 1.5 Hz, 2H), 7.79 (d, J = 8.0 Hz, 2H). ^{13}C NMR (125 MHz, $CDCl_3$): δ (ppm) 24.81, 27.19, 46.89, 56.61, 75.01, 115.07, 119.59, 120.01, 122.54, 123.78, 126.94, 127.15, 128.31, 130.80, 137.27, 138.26, 139.02, 150.84, 153.39, 153.89. HRMS (ESI): calcd for $C_{38}H_{38}NO_2^+$ $[M+NH_4]^+$ 540.2897, found 540.2910.

Synthesis of **BO4e**: Following the general procedure, BBr_3 (0.29 mL, d = 2.6 g/mL, 3.00 mmol, 3.0 equiv) was added dropwise to a mixture of **A4e** (523 mg, 1.00 mmol, 1.0 equiv) in toluene (20 mL) and *n*-hexane (20 mL) and stirred at room temperature for 24 hours, added $AlCl_3$ (267 mg, 2.00 mmol, 2.0 equiv) and stirred at 75 °C for 8 hours, cooled down to room temperature, added MesMgBr (5 mL, 1.0 M in THF, 5 mmol, 5.0 equiv) and stirred at room temperature for another 1 hour to afford **BO4e** (eluent: petroleum ether/dichloromethane = 100:1–10:1) as a slight yellow solid 325 mg in 43% yield. ^{1}H NMR (500 MHz, $CDCl_3$): δ (ppm) 1.62 (s, 12H), 2.29 (s, 12H), 2.43 (s, 6H), 7.00 (s, 4H), 7.31−7.36 (m, 4H), 7.47−7.49 (m, 2H), 7.76−7.77 (m, 2H), 8.14 (s, 2H), 8.42 (s, 2H), 8.56 (s, 2H). ^{13}C NMR (125 MHz, $CDCl_3$): δ (ppm) 21.38, 22.57, 27.39, 47.35, 114.21, 116.24, 120.79, 122.72, 124.31, 127.19, 127.42 (2C), 127.93, 128.44, 128.77, 137.49, 138.26, 138.37, 139.52, 140.17, 147.79, 153.57, 159.43. ^{11}B NMR (160 MHz, $CDCl_3$): δ (ppm) 45.87. HRMS (ESI): calcd for $C_{54}H_{48}[^{11}B]_2NaO_2$ $[M+Na]^+$ 773.3733, found 773.3712.

Synthesis of **BO5**:

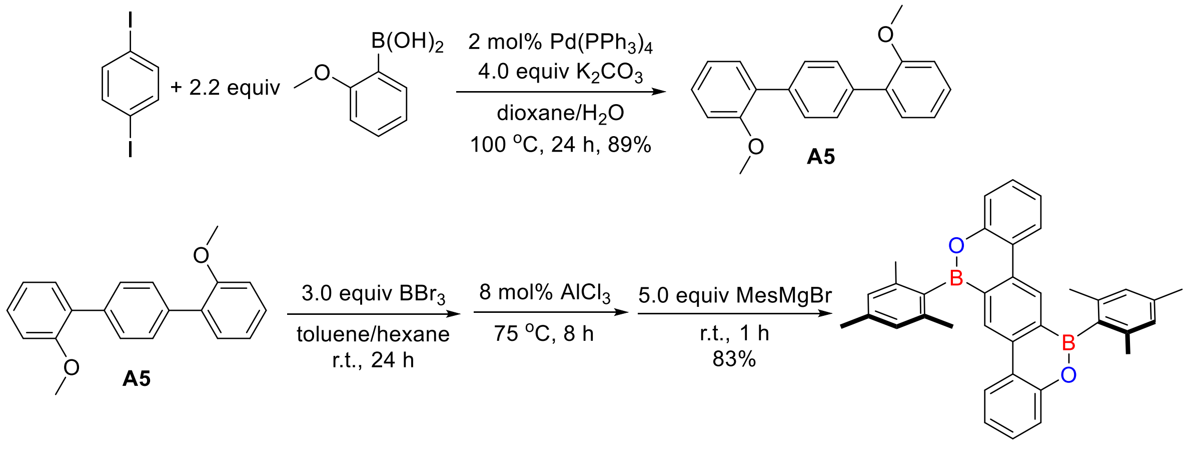

BO5

Synthesis of **A5**: Following the general procedure, the reaction of 1,4-diiodobenzene (6.60 g, 20.00 mmol, 1.0 equiv), 2-methoxylboronic acid (6.69 g, 44.00 mmol, 2.2 equiv), $Pd(PPh_3)_4$ (462 mg, 0.40 mmol, 0.02 equiv) and K_2CO_3 (11.06 g, 80.00 mmol, 4.0 equiv, in 30 mL H_2O) in dioxane (120 mL) at 100 °C for 24 hours afforded **A5** (eluent: petroleum ether/ethyl acetate = 10:1–3:1) as a white solid 5.17 g in 89% yield. ^{1}H NMR(500 MHz, DMSO-d_6): δ (ppm) 3.79 (s, 6H), 7.05 (t, J = 7.5 Hz,

2H), 7.13 (d, J = 7.5 Hz, 2H), 7.31−7.38 (m, 4H), 7.50 (s, 4H).

Synthesis of **BO5**: Following the general procedure, BBr_3 (1.16 mL, d = 2.6 g/mL, 12.00 mmol, 3.0 equiv) was added dropwise to a mixture of **A5** (1.16 g, 4.00 mmol, 1.0 equiv) in toluene (40 mL) and *n*-hexane (40 mL) and stirred at room temperature for 24 hours, added $AlCl_3$ (21 mg, 0.16 mmol, 0.04 equiv) and stirred at 75 °C for 8 hours, cooled down to room temperature, added MesMgBr (20 mL, 1.0 M in THF, 20 mmol, 5.0 equiv) and stirred at room temperature for another 1 hour to afford **BO5** (eluent: petroleum ether/dichloromethane = 100:1–10:1) as a white solid 1.72 g in 83% yield. m.p.: 320.1−322.5 °C. ^{1}H NMR (500 MHz, $CDCl_3$): δ (ppm) 2.26 (s, 12H), 2.43 (s, 6H), 7.01 (s, 4H), 7.29−7.33 (m, 2H), 7.45−7.48 (m, 2H), 7.55 (dd, J = 8.5, 1.0 Hz, 2H), 8.20 (dd, J = 8.0, 1.5 Hz, 2H), 8.74 (s, 2H). ^{13}C NMR (125 MHz, $CDCl_3$): δ (ppm) 21.38, 22.64, 110.57, 122.86, 123.63, 123.83, 127.56, 129.20, 130.60, 133.15, 136.53, 138.56, 140.26, 151.53. ^{11}B NMR (160 MHz, $CDCl_3$): δ (ppm) 46.85. HRMS (ESI): calcd for $C_{36}H_{32}[^{11}B]_2O_2]^+$ [M]$^+$ 518.2583, found 518.2586.

Synthesis of **BO6**:

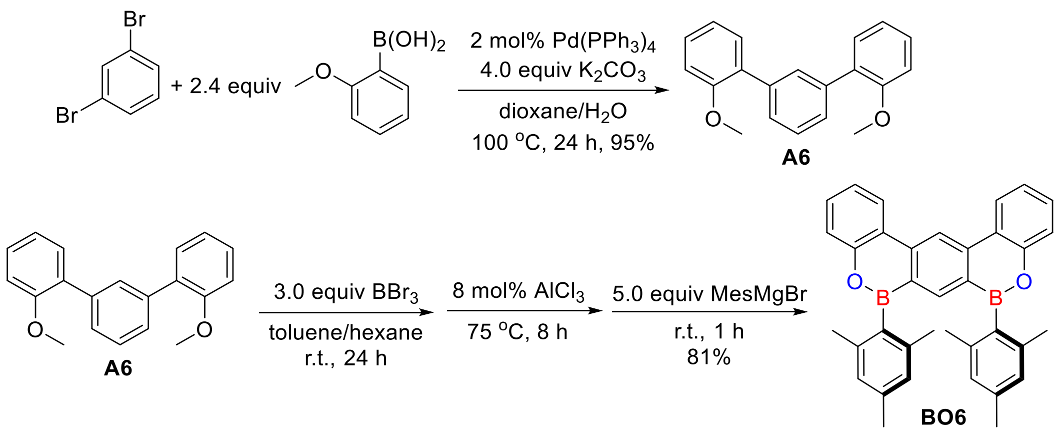

Synthesis of **A6**: Following the general procedure, the reaction of 1,3-dibromobenzene (1.18 g, 5.00 mmol, 1.0 equiv), 2-methoxylboronic acid (1.82 g, 12.00 mmol, 2.4 equiv), $Pd(PPh_3)_4$ (116 mg, 0.10 mmol, 0.02 equiv) and K_2CO_3 (2.76 g, 20.00 mmol, 4.0 equiv, in 20 mL H_2O) in dioxane (60 mL) at 100 °C for 24 hours afforded **A6** (eluent: petroleum ether/ethyl acetate = 20:1–10:1) as a white solid 1.38 g in 95% yield. ^{1}H NMR (500 MHz, $CDCl_3$): δ (ppm) 3.82 (s, 6H), 6.99 (d, J = 8.0 Hz, 2H), 7.02−7.05 (m, 2H), 7.30−7.34 (m, 2H), 7.38 (d, J = 7.5, 2.0 Hz, 2H), 7.43−7.46 (m, 1H), 7.50−7.52 (m, 2H), 7.69 (t, J = 1.5 Hz, 1H). ^{13}C NMR (125 MHz, $CDCl_3$): δ (ppm) 55.48, 111.14, 120.75, 127.49, 128.15, 128.49, 130.72, 130.75, 130.96, 138.11, 156.48. The ^{1}H NMR and ^{13}C NMR are agreement with the previous reoprt (Shukla, R., Lindeman, S. V., Rathore, R. Terphenyl Crowns: A new family

of receptors containing ethereal canopies that direct potassium cation onto benzenoid platforms for cation–π interactions. *Chem. Commun.* 5600–5602 (2009).

Synthesis of **BO6**: Following the general procedure, BBr_3 (1.16 mL, d = 2.6 g/mL, 12.00 mmol, 3.0 equiv) was added dropwise to a mixture of **A6** (581 mg, 2.00 mmol, 1.0 equiv) in toluene (30 mL) and *n*-hexane (30 mL) and stirred at room temperature for 24 hours, added $AlCl_3$ (42 mg, 0.32 mmol, 0.08 equiv) and stirred at 75 °C for 8 hours, cooled down to room temperature, added MesMgBr (10 mL, 1.0 M in THF, 10 mmol, 5.0 equiv) and stirred at room temperature for another 1 hour to afford **BO6** (eluent: petroleum ether/dichloromethane = 100:1–10:1) as a white solid 839 mg in 81% yield. m.p.: 271.8–272.9 °C. ^{1}H NMR (500 MHz, $CDCl_3$): δ (ppm) 2.15 (s, 12H), 2.30 (s, 6H), 6.84 (s, 4H), 7.43–7.47 (m, 2H), 7.54–7.58 (m, 4H), 8.32 (s, 1H), 8.59 (d, J = 6.5 Hz, 2H), 9.26 (s, 1H). ^{13}C NMR (125 MHz, $CDCl_3$): δ (ppm) 21.21, 22.50, 113.80, 120.88, 122.93, 123.55, 124.00, 127.38, 128.03, 130.16, 133.79, 138.25, 140.02, 142.07, 148.71, 152.39. ^{11}B NMR (160 MHz, $CDCl_3$): δ (ppm) 46.23. HRMS (ESI): calcd for $C_{36}H_{33}[^{11}B]_2O_2^+$ $[M+H]^+$ 519.2661, found 519.2676. The obtained **BO6** was further purified by recrystallization in toluene/methanol; and then 0.60 g **BO6** was sublimated in a seven zone thermal gradient sublimator at 130/150/160/200/240/240/260 °C, 5.0×10^{-5} Torr to obtain white solid 0.53 g in 88% yield; purity, 99.28% by HPLC, HPLC analysis condition: column: Masiall® C18-BIO, 5μm, 250 × 4.6 mm; mobile phase: methanol/tetrahydrofuran = 95/5(v/v); flow rate: 1.0 mL/min; Abs. detector: 254 nm.

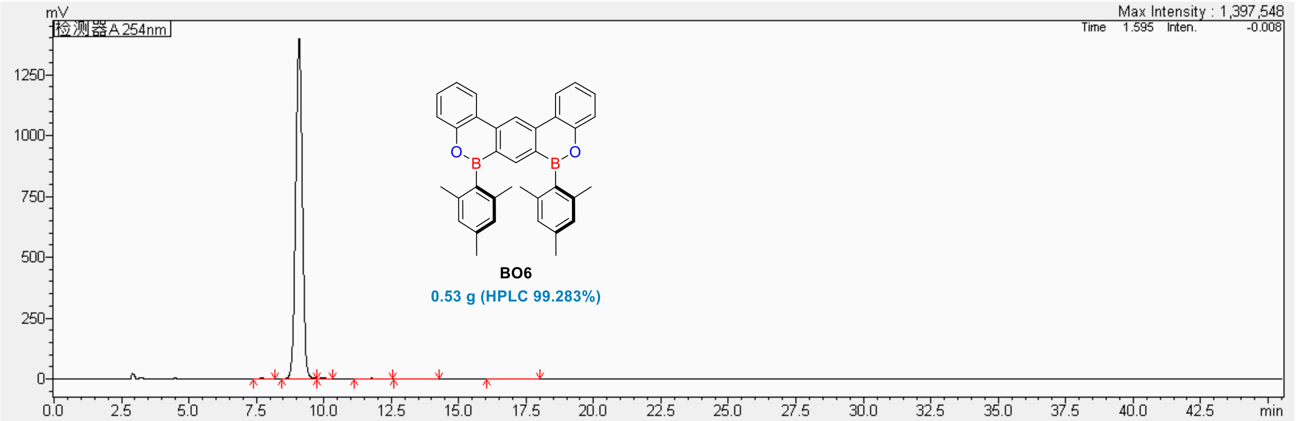

Peak	RetTime /[min]	Area /[mAU*s]	Area /%	Height /[mAU]	Start Time /[min]	End Time/ [min]
1	7.702	54167	0.224	2746	7.390	8.190
2	9.085	23980021	99.283	1397140	8.440	9.760
3	9.962	55699	0.231	3036	9.760	10.330
4	11.765	47246	0.196	2341	11.130	12.550
5	12.991	7421	0.031	154	12.590	14.270
6	16.911	8648	0.036	260	16.040	18.010
Total		24153201	100.000	1405677		

Attempt for the synthesis of **BO7a**:

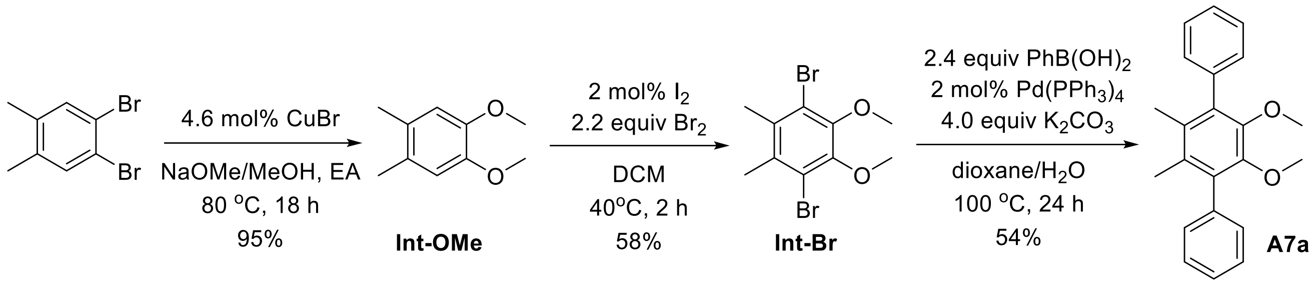

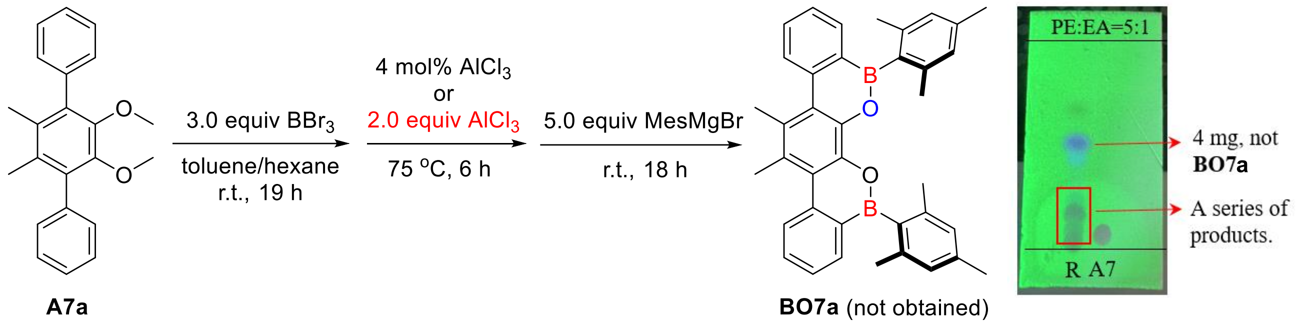

Synthesis of **Int-OMe**: 1,2-Dibromo-4,5-dimethylbenzene (10.0 g, 37.9 mmol, 1.0 equiv), CuBr (250 mg, 1.74 mmol, 4.6 mol%), NaOMe/MeOH (30 wt%, 300 mL), and ethyl acetate (3 mL) were added sequentially to a dry three-neck round-bottom flask equipped with a magnetic stir bar. The flask was evacuated and backfilled with nitrogen, this evacuation and backfill procedure was repeated twice. The deep-blue solution was then heated at 80 °C for 18 hours. The solution was cooled to room temperature, poured into H_2O (300 mL), and extracted with dichloromethane. The combined organic layers were dried with Na_2SO_4 and concentrated under reduced pressure, then the residue was purified through column chromatography on silica gel (eluent: petroleum ether–petroleum ether/acetic ether =10:1) to obtain the desired product as a white solid 6.0 g in 95% yield. ^{1}H NMR (500 MHz, $CDCl_3$): δ (ppm) 2.00 (s, 6H), 3.84 (s, 6H), 6.67 (s, 2H).

Synthesis of **Int-Br**: **Int-OMe** (4.90 g, 29.48 mmol, 1.0 equiv) was dissolved in 30 mL of dichloromethane. To this stirred solution was added I_2 (150 mg, 0.59 mmol, 0.02 equiv) followed by a slow dropwise addition of a solution of Br_2 (10.37 g, 64.86 mmol, 2.2 equiv) in 30 mL of dichloromethane. After the addition was completed, the resulting solution was heated to 40 °C for 2 hours. Upon cooling, 5M aq. NaOH (30 mL) was added to the reaction mixture. The filtrate was washed with water (30 mL), dried over anhydrous sodium sulfate, and concentrated under reduced pressure, then the residue was purified through column chromatography on silica gel (eluent: petroleum ether) to obtain the desired product as a white solid 5.54 g in 58% yield. ^{1}H NMR (500 MHz, $CDCl_3$): δ (ppm) 2.44 (s, 6H), 3.87 (s, 6H).

Synthesis of **A7a**: Following the general procedure, the reaction of **Int-Br** (5.00 g, 15.43 mmol, 1.0 equiv), phenylboronic acid (4.52 g, 37.03 mmol, 2.4 equiv), $Pd(PPh_3)_4$ (358 mg, 0.31 mmol, 0.02 equiv) and K_2CO_3 (8.53 g, 61.72 mmol, 4.0 equiv, in 10 mL H_2O) in dioxane (40 mL) at 100 °C for 24

hours afforded **A7a** (eluent: petroleum ether– petroleum ether/acetic ether =100:1) as a white solid 2.65 g in 54% yield. ^{1}H NMR (500 MHz, $CDCl_3$): δ (ppm) 2.00 (s, 6H), 3.59 (s, 6H), 7.28−7.30(m, 4H), 7.35−7.38 (m, 2H), 7.44−7.46(m, 4H). ^{13}C NMR (125 MHz, $CDCl_3$): δ (ppm) 17.55, 60.58, 126.76, 128.01, 129.79, 130.95, 136.20, 138.19, 148.40.

Synthesis of **BO7a**: Following the general procedure, BBr_3 (0.29 mL, d = 2.6 g/mL, 3.00 mmol, 3.0 equiv) was added dropwise to a mixture of **A7a** (318 mg, 1.00 mmol, 1.0 equiv) in toluene (5 mL) and *n*-hexane (5 mL) and stirred at room temperature for 19 hours, added $AlCl_3$ (5 mg, 0.04 mmol, 0.04 equiv) and stirred at 75 °C for 6 hours, cooled down to room temperature, added MesMgBr (5 mL, 1.0 M in THF, 5 mmol, 5.0 equiv) and stirred at room temperature for another 18 hours; TLC showed that the reaction was messy, no desired **BO7a** was isolated. We also tried to used more $AlCl_3$ (267 mg, 2.00 mmol, 2.0 equiv), but the products shown by TLC were similar.

Attempt for the synthesis of **BO7b**:

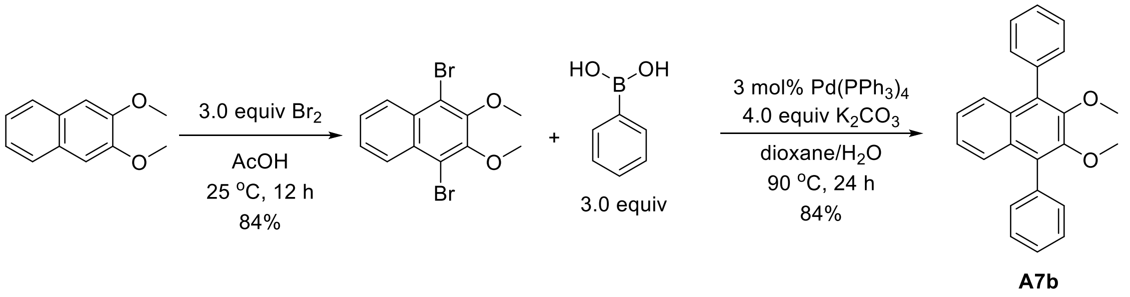

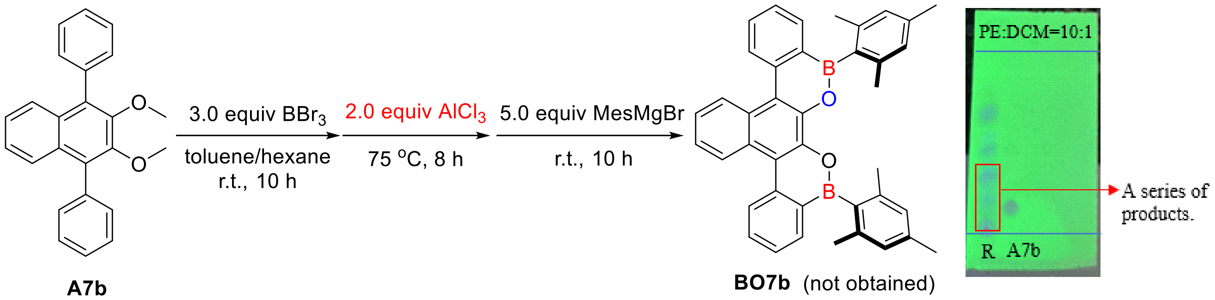

Synthesis of 1,4-dibromo-2,3-dimethoxynaphthalene: 2,3-dimethoxynaphthalene (8.17 g, 43.40 mmol, 1.0 equiv, in 500 mL AcOH) was added sequentially to a dry one -neck round-bottom flask and cooled to 0°C. To this stirred solution was added Br_2 (20.81 g, 130.20 mmol, 3.0 equiv), and the reaction was stirred at room temperature for 12 hours. The solution was poured into 5M aq. NaOH (150 mL) and extracted with acetic ether, and dried over anhydrous sodium sulfate, and concentrated under reduced pressure, then the residue was purified through column chromatography on silica gel (eluent: petroleum ether/dichloromethane = 50:1–30:1) to obtain the desired product as a white solid 12.60 g in 84% yield. ^{1}H NMR (500 MHz, $CDCl_3$): δ (ppm) 4.00 (s, 6H), 7.55−7.59 (m, 2H), 8.22−8.26 (m, 2H).

Synthesis of **A7b**: Following the general procedure, the reaction of 2,3-dimethoxynaphthalene (4.00 g, 11.60 mmol, 1.0 equiv), phenylboronic acid (4.24 g, 34.80 mmol, 3.0 equiv), $Pd(PPh_3)_4$ (402 mg, 0.35 mmol, 0.03 equiv) and K_2CO_3 (6.41 g, 46.40 mmol, 4.0 equiv, in 10 mL H_2O) in dioxane (40 mL) at 90 °C for 24 hours afforded **A7b** (eluent: petroleum ether– petroleum ether/ dichloromethane =20:1) as a white solid 3.3 g (impurity) in 84% yield. (1.5 g of crude product was further purified by sublimation to obtain **A7b** as a white solid 620 mg) ^{1}H NMR (500 MHz, $CDCl_3$): δ (ppm) 3.72 (s, 6H), 7.25−7.29 (m, 2H), 7.44−7.48 (m, 6H), 7.51−7.55 (m, 6H).^{13}C NMR (125 MHz, $CDCl_3$): δ (ppm) 61.07, 124.95, 125.92, 127.34, 128.21, 130.51, 130.59, 132.16, 136.17, 149.41.

Synthesis of **BO7b**: Following the general procedure, BBr_3 (0.29 mL, d = 2.6 g/mL, 3.00 mmol, 3.0 equiv) was added dropwise to a mixture of **A7b** (340 mg, 1.00 mmol, 1.0 equiv) in toluene (5 mL) and *n*-hexane (5 mL) and stirred at room temperature for 10 hours, added $AlCl_3$ (267 mg, 2.00 mmol, 2.0 equiv) and stirred at 75 °C for 8 hours, cooled down to room temperature, added MesMgBr (5 mL, 1.0 M in THF, 5.00 mmol, 5.0 equiv) and stirred at room temperature for another 10 hours; TLC showed that the reaction was messy, no desired **BO7b** was isolated.

Scheme S1

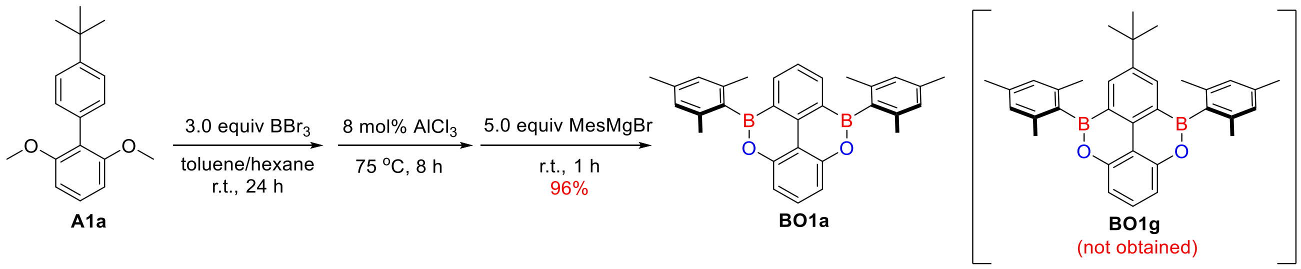

Scheme S2

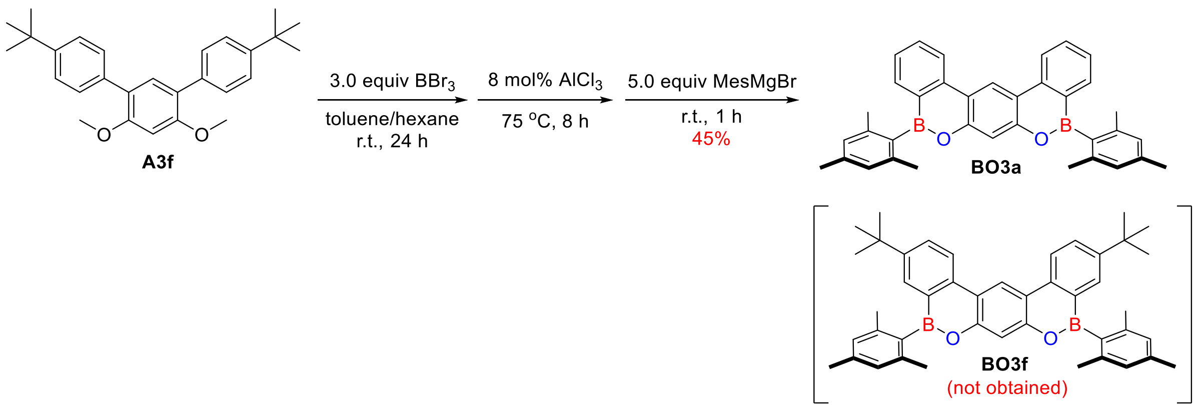

Supplementary Fig. 3. $AlCl_3$ induced de-*tert*-butylation reactions. The observed de-*tert*-butylation reactions catalyzed by $AlCl_3$

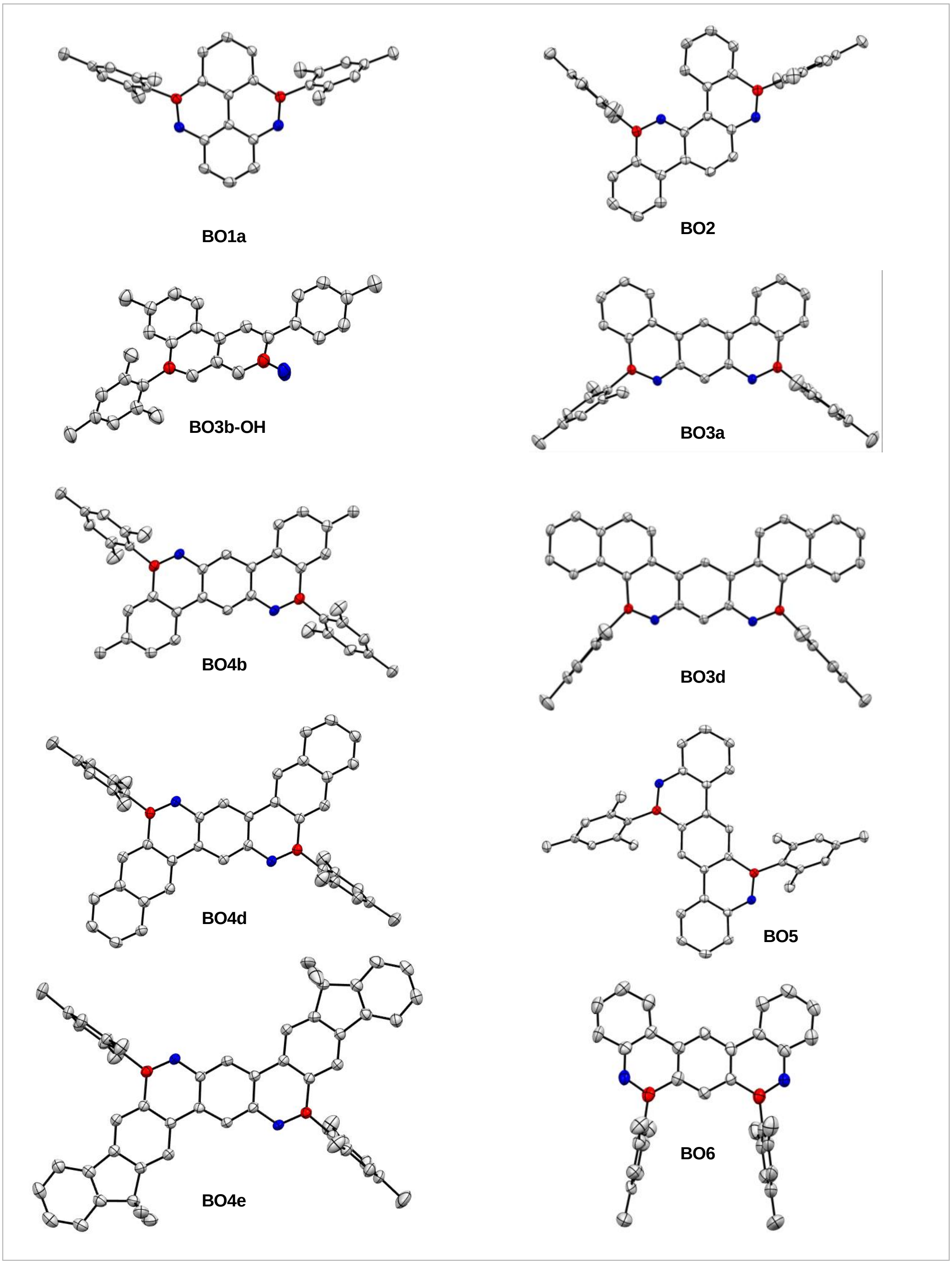

Supplementary Fig. 4. Single crystal diffraction structures of BO-PAHs. ORTEP drawing of X-ray single crystal diffraction structures of **BO1a**, **BO2**, **BO3a**, **BO3b-OH**, **BO3d**, **BO4b**, **BO4d**, **BO4e**, **BO5** and **BO6**. Hydrogen atoms were omitted for clarity. Ellipsoids are shown at the 35% or 50% probability level.

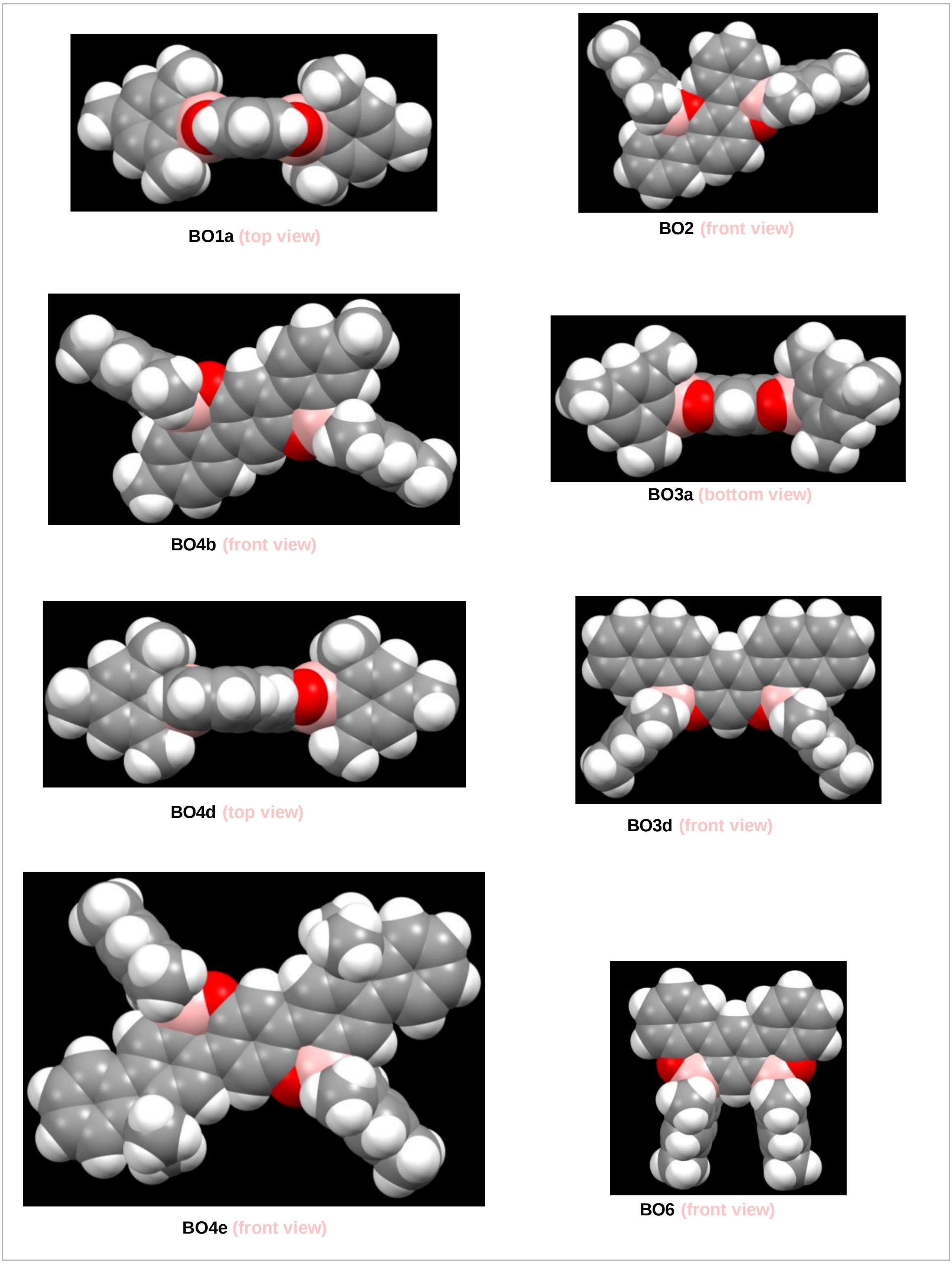

Supplementary Fig. 5. Spacefill drawing of X-ray single crystal diffraction structures of **BO1a**, **BO2**, **BO3a**, **BO3d**, **BO4b**, **BO4d**, **BO4e** and **BO6**. B, pink; O, red.

Supplementary Table 1. Crystal data and structure refinements for BO1a and BO2

Compound	**BO1a**	**BO2**
CCDC number	1954663	1954662
Empirical formula	$C_{30}H_{28}B_2O_2$	$C_{36}H_{32}B_2O_2$
Formula weight	442.14	518.23
Temperature (K)	293(2) K	193(2)
Wavelength (Å)	0.71073	0.71073
Crystal system	monoclinic	orthorhombic
Space group	P 21/n	P 21 21 21
a (Å)	12.3732(4)	8.0150(3)
b (Å)	8.0799(3)	14.7515(6)
c (Å)	25.7096(10)	24.6982(8)
α (°)	90	90
β (°)	100.6030(10)	90
γ (°)	90	90
Volume ($Å^3$)	2526.41(16)	2920.15(19)
Z	4	4
F(000)	936	1096
Crystal size (mm^3)	0.200×0.150×0.120	0.200×0.160×0.130
θ (deg) for collection (°)	2.647 to 25.498	2.672 to 25.985
Index ranges	–14<=h<=14, –9<=k<=9, –31<=l<=31	–9<=h<=9, –18<=k<=14, –30<=l<=27
Reflections collected	23738	14696
Unique (R_{int})	4665 (0.0349)	5706 (0.0593)
Data / restraints / parameters	4665 / 0 / 314	5706 / 0 / 368
Goodness-of-fit on F^2	1.050	1.032
Final R indices [I>2σ(I)]	R_1 = 0.0493<br>wR_2 = 0.1275	R_1 = 0.0483<br>wR_2 = 0.1208
R indices (all data)	R_1 = 0.0722<br>wR_2 = 0.1462	R_1 = 0.0586<br>wR_2 = 0.1313
Largest diff. peak and hole ($Å^{-3}$)	0.155/–0.125	0.201/–0.178

Supplementary Table 2. Crystal data and structure refinements for BO3a, BO3d and BO3b-OH

Compound	**BO3a**	**BO3d**	**BO3b-OH**
CCDC number	1954665	1954664	2290997
Empirical formula	$C_{36}H_{32}B_2O_2$	$C_{44}H_{36}B_2O_2$	$C_{29}H_{27}BO_2$
Formula weight	518.23	618.35	418.31
Temperature (K)	293.04	293(2)	293(2)
Wavelength (Å)	0.71073	0.71073	0.71073
Crystal system	monoclinic	monoclinic	monoclinic
Space group	C 2/c	C 2	P21/n
a (Å)	34.7317(9)	30.8251(8)	17.5960(5)
b (Å)	7.7855(2)	7.5638(2)	7.5552(2)
c (Å)	24.5964(6)	17.0835(4)	19.2892(5)
α (°)	90	90	90
β (°)	98.6690(10)	92.9740(10)	114.5900(10)
γ (°)	90	90	90
Volume ($Å^3$)	6575.0(3)	3977.74(17)	2331.77(11)
Z	8	4	4
F(000)	2192	1304	888.0
Crystal size (mm^3)	0.2×0.17×0.13	0.200×0.120×0.100	0.2 × 0.14 × 0.1
θ (deg) for collection (°)	2.784 to 25.996	2.388 to 25.499	4.448 to 66.996
Index ranges	$-42 \le h \le 42$, $-9 \le k \le 9$, $-30 \le l \le 30$	$-36 \le h \le 37$, $-9 \le k \le 9$, $-19 \le l \le 20$	$-19 \le h \le 21$, $-8 \le k \le 8$, $-22 \le l \le 23$
Reflections collected	37277	22620	19160
Unique (R_{int})	6409 (0.0409)	9027 (0.0355)	0.0735
Data / restraints / parameters	6409 / 0 / 368	9.27 / 1 / 439	4083 / 1 / 297
Goodness-of-fit on F^2	1.020	1.034	1.031
Final R indices [I>2σ(I)]	R_1 = 0.0509 wR_2 = 0.1354	R_1 = 0.0607 wR_2 = 0.1635	R_1 = 0.0718, wR_2 = 0.2011
R indices (all data)	R_1 = 0.0793 wR_2 = 0.1561	R_1 = 0.0928 wR_2 = 0.1863	R_1 = 0.0924, wR_2 = 0.2241
Largest diff. peak and hole ($Å^{-3}$)	0.21/–0.14	0.25/–0.20	0.39/–0.24

Supplementary Table 3. Crystal data and structure refinements for BO4b, BO4d and BO4e·CH_2Cl_2

Compound	**BO4b**	**BO4d**	**BO4e·CH_2Cl_2**
CCDC number	1954661	1966566	1968760
Empirical formula	$C_{38}H_{36}B_2O_2$	$C_{44}H_{36}B_2O_2$	$C_{55}H_{50}B_2Cl_2O_2$
Formula weight	546.29	618.35	835.47
Temperature (K)	192(2)	170	170
Wavelength (Å)	0.71073	0.71073	0.71073
Crystal system	orthorhombic	monoclinic	monoclinic
Space group	P b c a	$P2_1$-C	$P2_1/n$
a (Å)	18.752(2)	7.3765(3)	14.345(6)
b (Å)	7.4170(10)	28.2294(12)	15.450(6)
c (Å)	21.713(2)	8.0685(3)	20.591(9)
α (°)	90	90	90
β (°)	90	95.884(2)	99.88(2)
γ (°)	90	90	90
Volume ($Å^3$)	3019.9(6)	1671.29(12)	4496(3)
Z	4	2	4
F(000)	1160	652.0	1760.0
Crystal size (mm^3)	0.180×0.150×0.120	0.15×0.05×0.03	0.39×0.23×0.19
θ (deg) for collection (°)	2.871 to 26.000	2.638 to 34.051	2.306 to 26.405
Index ranges	–23<=h<=18, –9<=k<=9, –26<=l<=25	–11<=h<=10, –40<=k<=40, –12<=l<=12	–17<=h<=17, –18<=k<=19, –25<=l<=25
Reflections collected	13983	39127	62483
Unique (R_{int})	2956 (0.0628)	5819 (0.0867)	9215 (0.0619)
Data / restraints / parameters	2956 / 0 / 195	5819 / 0 / 220	9215 / 99 / 588
Goodness-of-fit on F^2	1.027	1.022	1.024
Final R indices [I>2σ(I)]	R_1 = 0.0537 wR_2 = 0.1312	R_1 = 0.0740 wR_2 = 0.1696	R_1 = 0.0735 wR_2 = 0.2018
R indices (all data)	R_1 = 0.0760 wR_2 = 0.1487	R_1 = 0.1644 wR_2 = 0.2208	R_1 = 0.1017 wR_2 = 0.2315
Largest diff. peak and hole ($Å^{-3}$)	0.266/–0.184	0.34/–0.27	0.67/–0.87

Supplementary Table 4. Crystal data and structure refinements for BO5 and BO6

Compound	**BO5**	**BO6**
CCDC number	2280610	2029823
Empirical formula	$C_{36}H_{32}B_2O_2$	$C_{36}H_{32}B_2O_2$
Formula weight	518.23	518.23
Temperature (K)	170.00	192(2)
Wavelength (Å)	0.71073	0.71073
Crystal system	monoclinic	monoclinic
Space group	$P2_1/c$	P c
a (Å)	9.1072(6)	19.776(2)
b (Å)	10.3791(7)	18.2753(15)
c (Å)	15.0015(10)	16.4263(15)
α (°)	90	90
β (°)	105.825(2)	90.339(3)
γ (°)	90	90
Volume (Å^3)	1364.27(16)	5936.7(9)
Z	2	8
F(000)	548.0	2192.0
Crystal size (mm^3)	0.06 × 0.04 × 0.03	0.18×0.14×0.1
θ (deg) for collection (°)	4.39 to 60.561	1.612 to 24.999
Index ranges	–11≤ h ≤ 11, –13≤ k ≤ 13, –19 ≤ l ≤ 19	–23<=h<=22, –21<=k<=21, –19<=l<=19
Reflections collected	11533	82048
Unique (R_{int})		19509 (0.1172)
Data / restraints / parameters	3107/0/184	19509 / 2 / 1467
Goodness-of-fit on F^2	1.072	1.016
Final R indices [I>2σ(I)]	R_1 = 0.0528, wR_2 = 0.1341	R_1 = 0.0731 wR_2 = 0.747
R indices (all data)	R_1 = 0.0738, wR_2 = 0.1453	R_1 = 0.1233 wR_2 = 0.2127
Largest diff. peak and hole (Å^{-3})	0.26/–0.28	0.22/–0.22

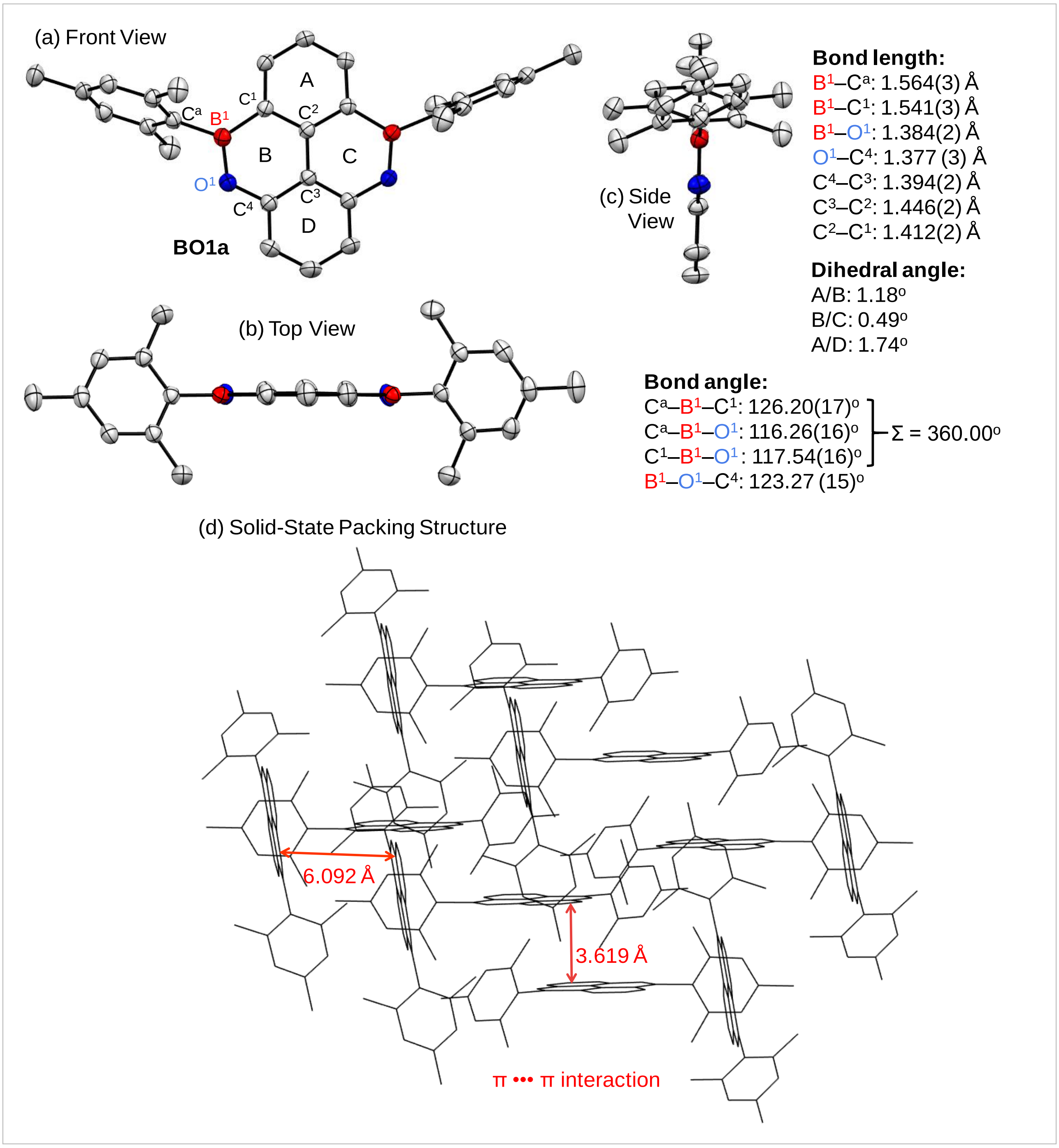

Supplementary Fig. 6. Single crystal structure. X-ray single crystal diffraction structure and crystal packing of **BO1a**. **a** Front View. **b** Top View. **c** Side View. **d** Solide-State Packing Structure. Hydrogen atoms were omitted for clarity. Ellipsoids are shown at the 50% probability level.

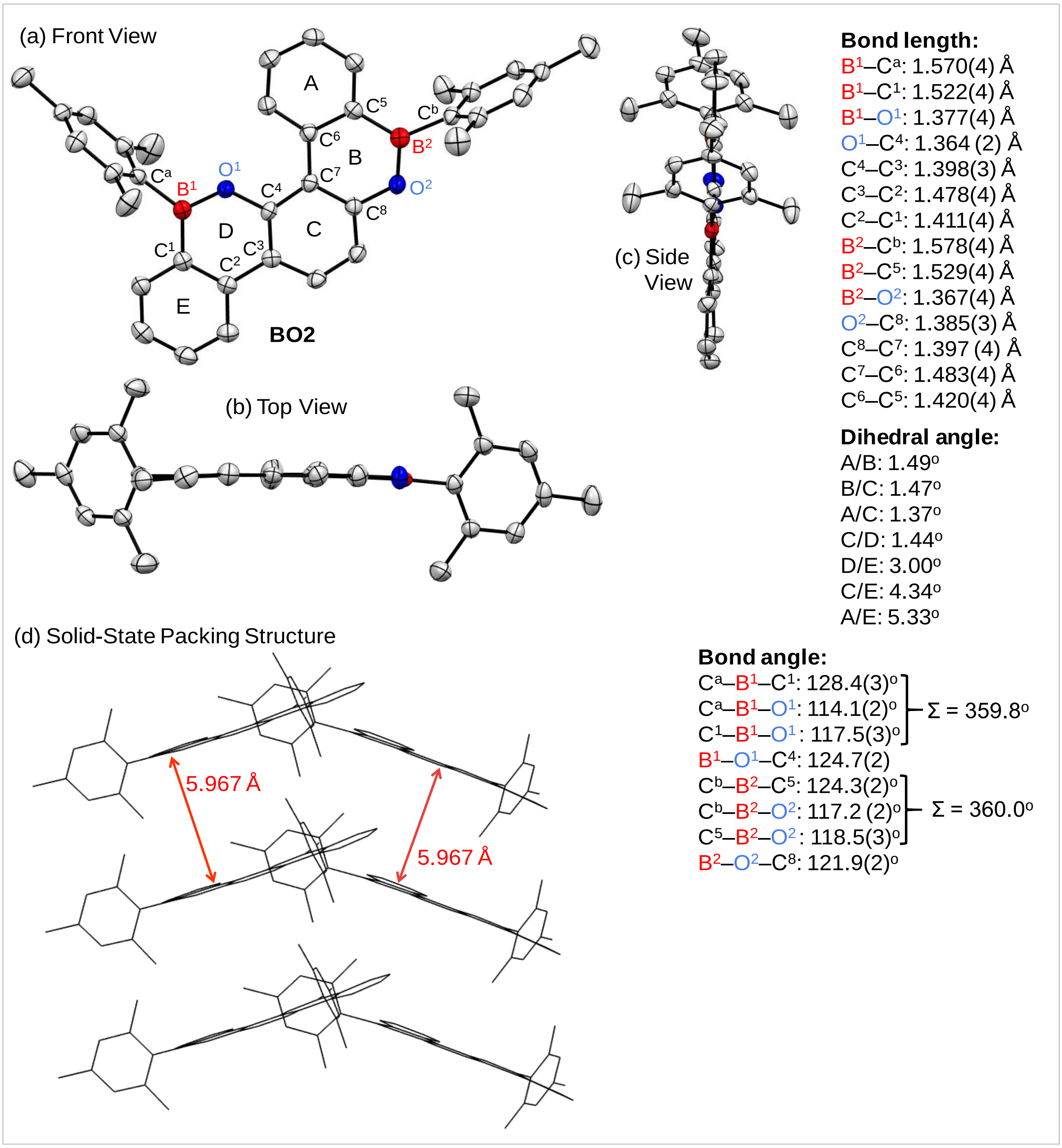

Supplementary Fig. 7. Single crystal structure. X-ray single crystal diffraction structure and crystal packing of **BO2**. **a** Front View. **b** Top View. **c** Side View. **d** Solide-State Packing Structure. Hydrogen atoms were omitted for clarity. Ellipsoids are shown at the 50% probability level.

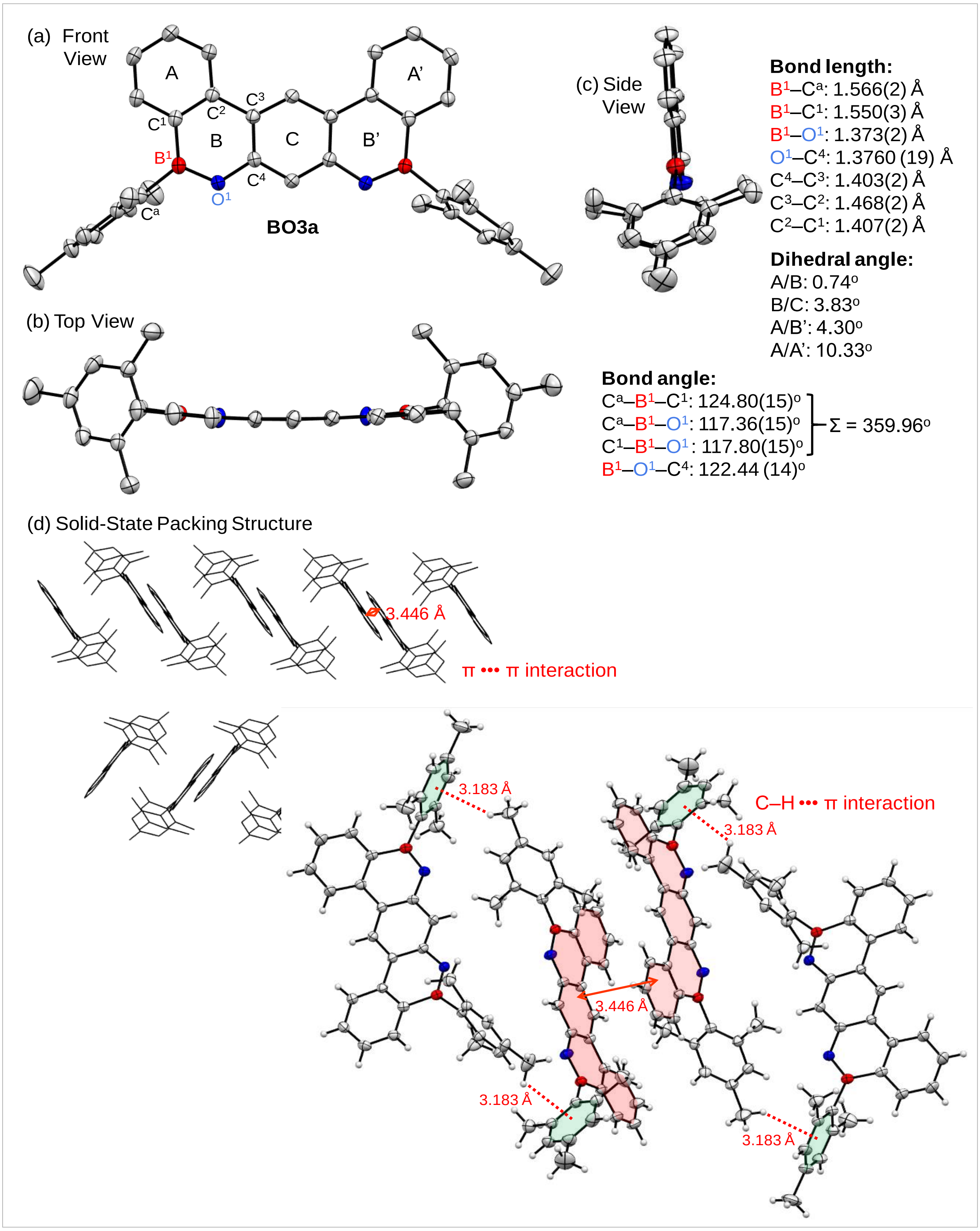

Supplementary Fig. 8. Single crystal structure. X-ray single crystal diffraction structure and crystal packing of **BO3a**. **a** Front View. **b** Top View. **c** Side View. **d** Solide-State Packing Structure. Hydrogen atoms were omitted for clarity. Ellipsoids are shown at the 50% probability level.

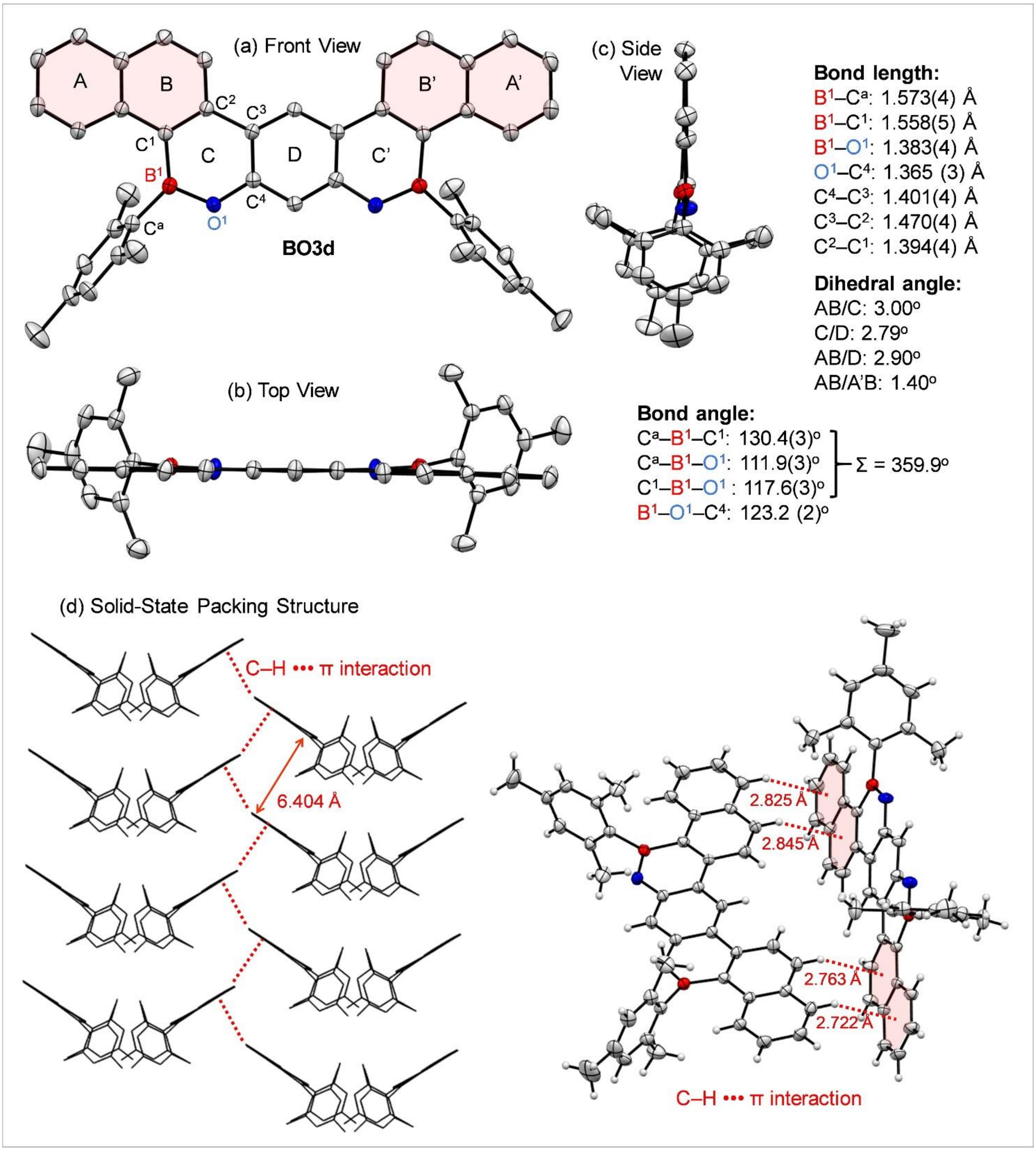

Supplementary Fig. 9. Single crystal structure. X-ray single crystal diffraction structure and crystal packing of **BO3d**. **a** Front View. **b** Top View. **c** Side View. **d** Solide-State Packing Structure. Hydrogen atoms were omitted for clarity. Ellipsoids are shown at the 50% probability level.

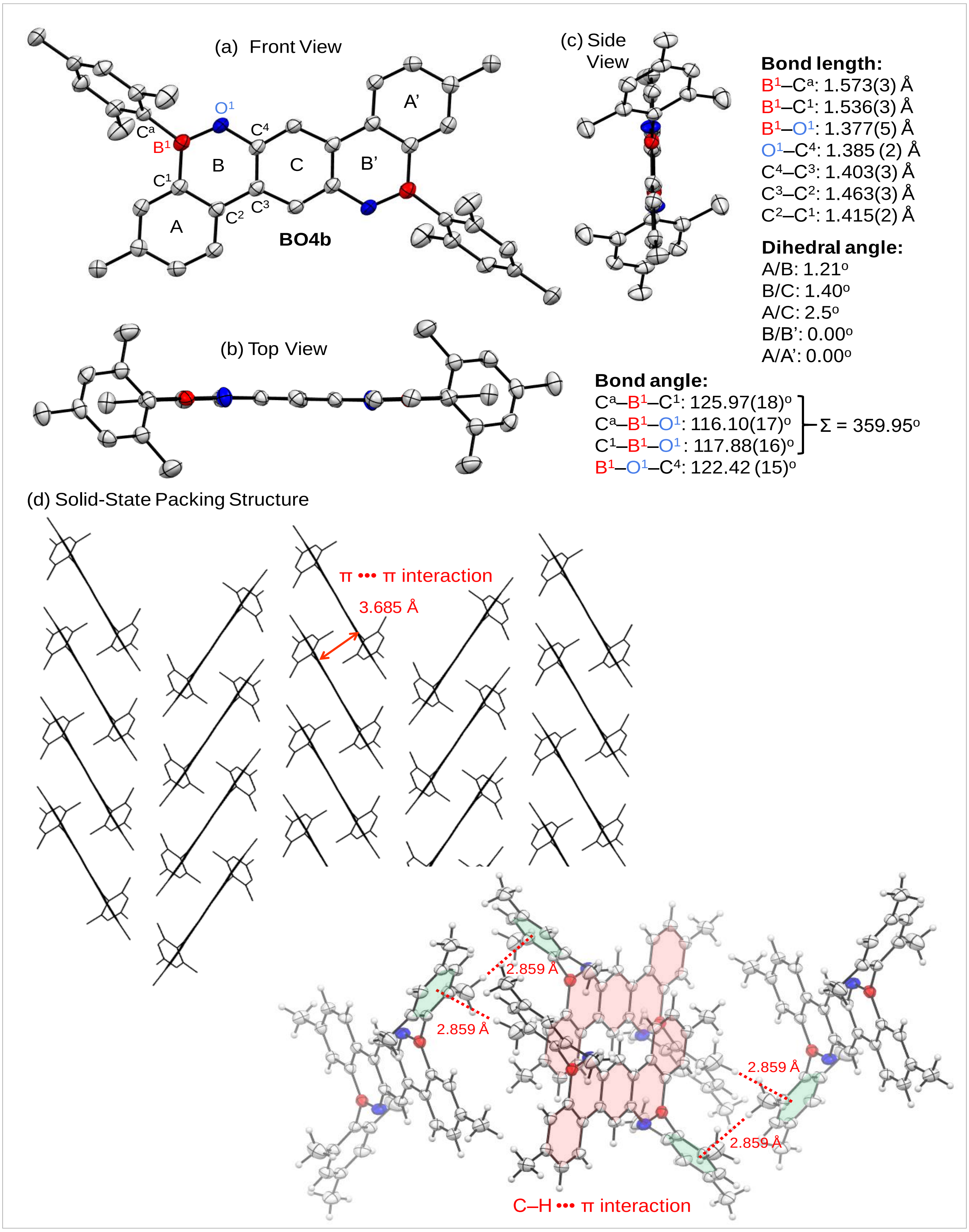

Supplementary Fig. 10. Single crystal structure. X-ray single crystal diffraction structure and crystal packing of **BO4b**. **a** Front View. **b** Top View. **c** Side View. **d** Solide-State Packing Structure. Hydrogen atoms were omitted for clarity. Ellipsoids are shown at the 50% probability level.

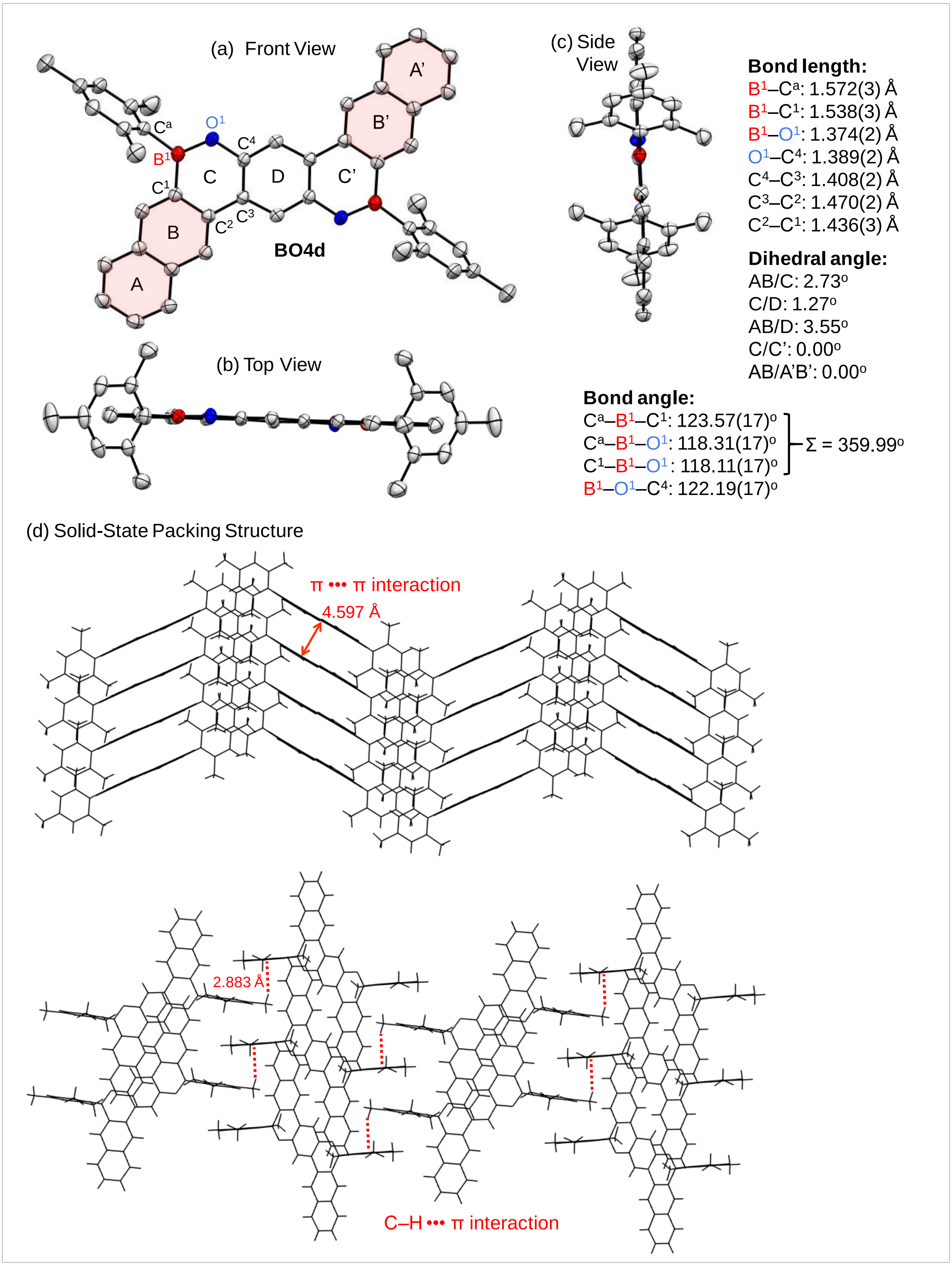

Supplementary Fig. 11. Single crystal structure. X-ray single crystal diffraction structure and crystal packing of **BO4d**. **a** Front View. **b** Top View. **c** Side View. **d** Solide-State Packing Structure. Hydrogen atoms were omitted for clarity. Ellipsoids are shown at the 50% probability level.

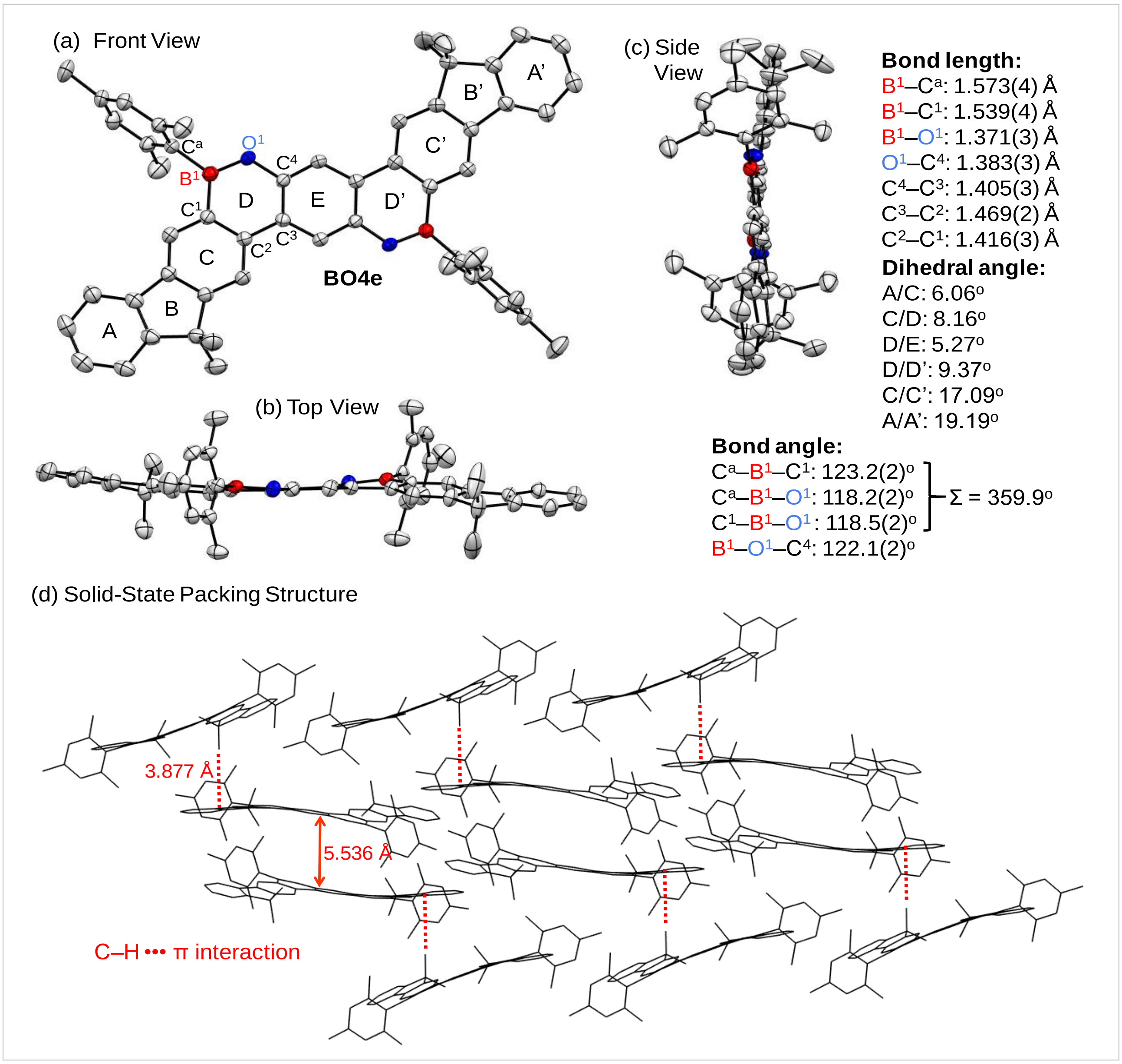

Supplementary Fig. 12. Single crystal structure. X-ray single crystal diffraction structure and crystal packing of **BO4e**. **a** Front View. **b** Top View. **c** Side View. **d** Solide-State Packing Structure. Hydrogen atoms and solvent dichloromethane molecules were omitted for clarity. Ellipsoids are shown at the 50% probability level.

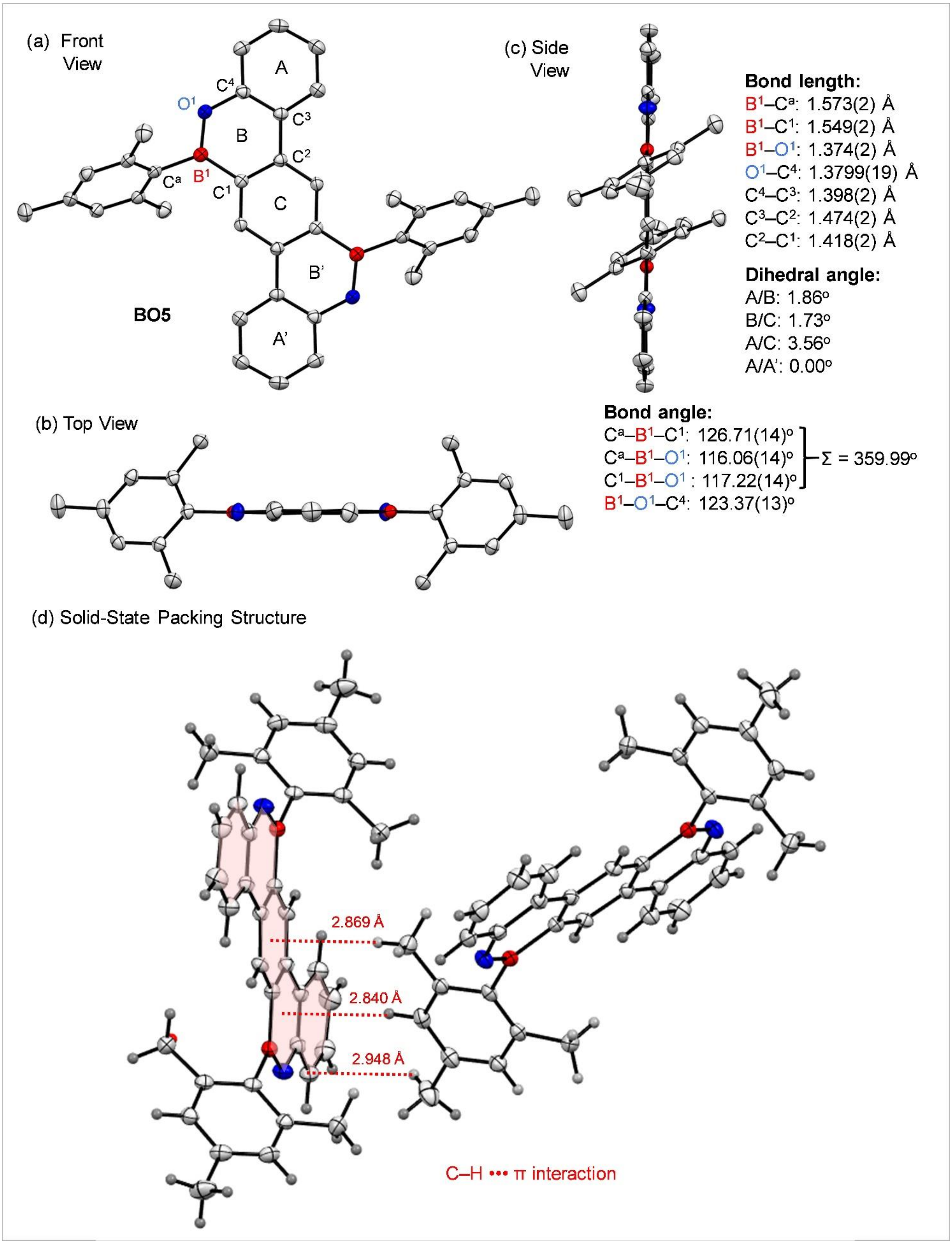

Supplementary Fig. 13. Single crystal structure. X-ray single crystal diffraction structure and crystal packing of **BO5**. **a** Front View. **b** Top View. **c** Side View. **d** Solide-State Packing Structure. Hydrogen atoms were omitted for clarity. Ellipsoids are shown at the 50% probability level.

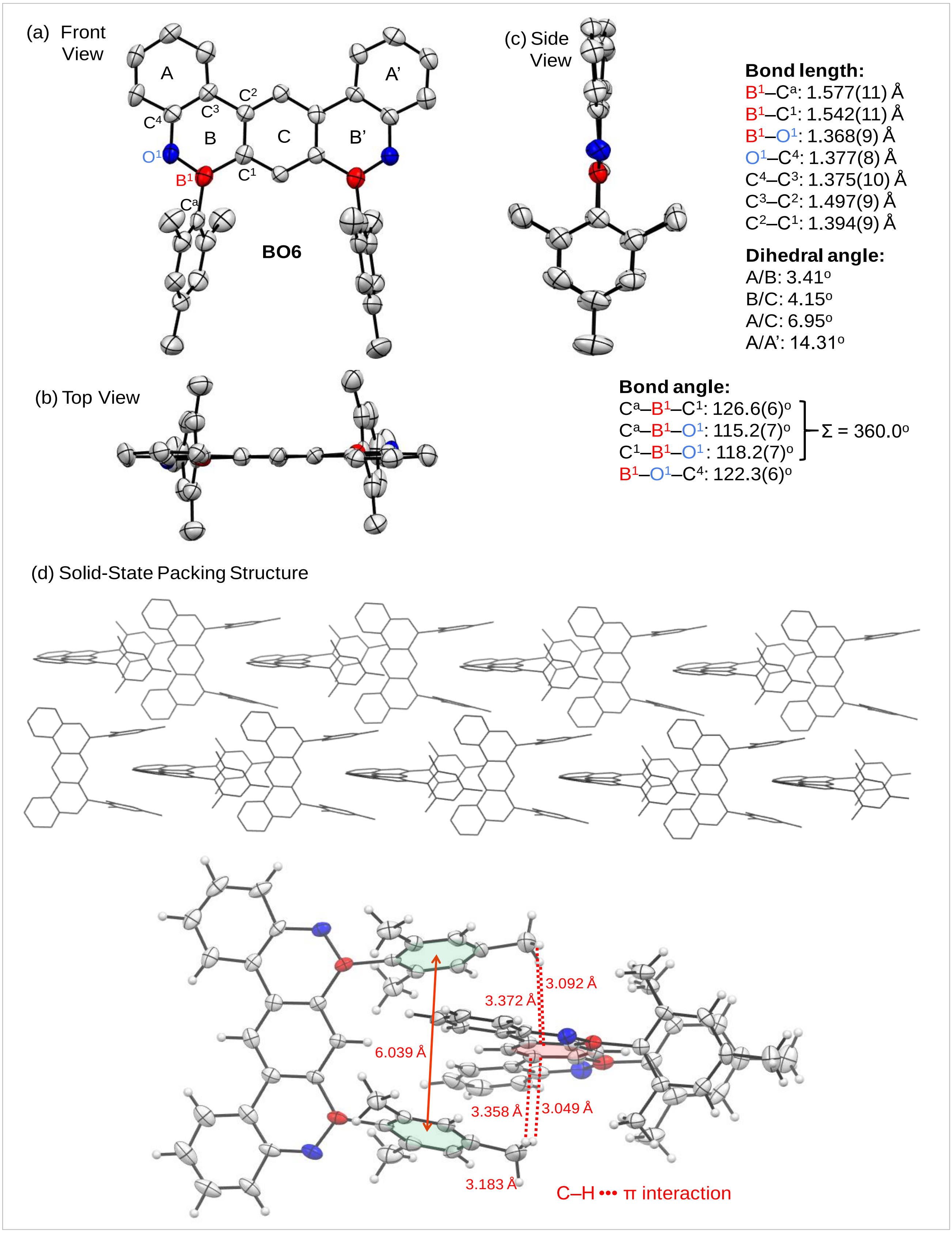

Supplementary Fig. 14. Single crystal structure. X-ray single crystal diffraction structure and crystal packing of **BO6**. **a** Front View. **b** Top View. **c** Side View. **d** Solide-State Packing Structure. Hydrogen atoms were omitted for clarity. Ellipsoids are shown at the 50% probability level.

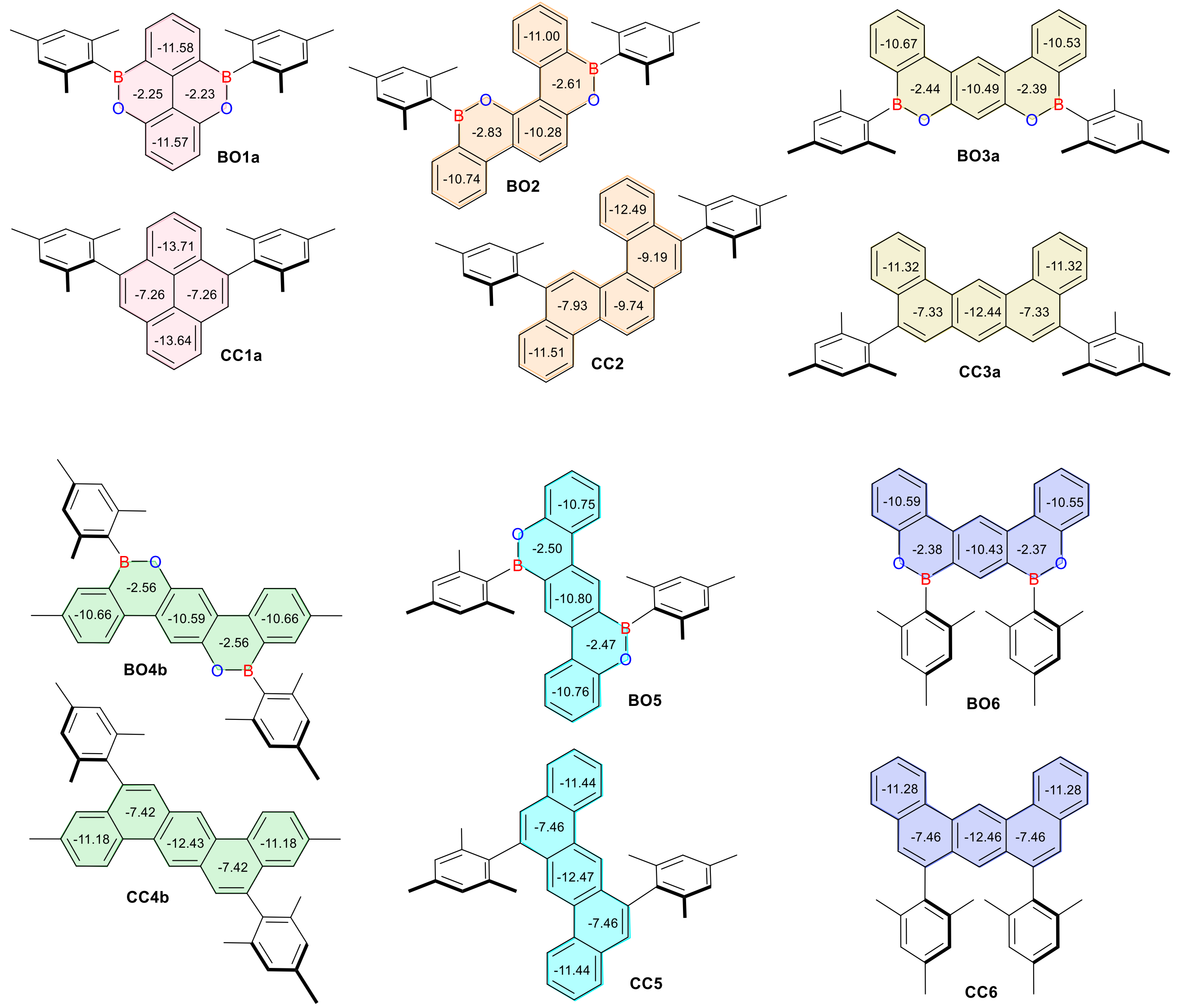

Supplementary Fig. 15. NICS(1) values of dBO-PAHs. Comparison of the calculated NICS(1) values of dBO-PAHs **BO1a**, **BO2**, **BO3a**, **BO4b**, **BO5**, **BO6** and their corresponding PAH analogues **CC1a**, **CC2**, CC**3a**, **CC4b**, **CC5**, **CC6**. The calculations were performed at B3LYP/6-31G* level.

Supplementary Table 5. Comparison of calculated HOMO and LUMO levels and energy gaps of the dBO-PAHs and their corresponding carbon analogues

**BO-PAH**	*cat.* HOMO/LUMO/E_g [eV]	**BO-PAH**	*cat.* HOMO/LUMO/E_g [eV]	**BO-PAH**	*cat.* HOMO/LUMO/E_g [eV]
**CC1a**	–5.28/–1.47/3.81	**CC2**	–5.47/–1.28/4.19	**CC4a**	–5.32/–1.49/3.83
**BO1a**	–6.06–1.50/4.56	**BO2**	–5.87/–1.32/4.55	**BO4a**	–5.82/–1.64/4.18
**CC1b**	–5.24/–1.44/3.80	**CC3a**	–5.38/–1.46/3.92	**CC4b**	–5.24/–1.41/3.83
**BO1b**	–6.02/–1.47/4.55	**BO3a**	–5.84/–1.45/4.39	**BO4b**	–5.65/–1.55/4.10
**CC1c**	–5.06/–1.41/3.65	**CC3b**	–5.27/–1.41/3.86	**CC4c**	–5.12/–1.76/3.36
**BO1c**	–6.02/–1.43/4.59	**BO3b**	–5.69/–1.38/4.31	**BO4c**	–5.49/–2.00/3.49
**CC1d**	–5.30/–1.50/3.80	**CC3c**	–5.31/–1.57/3.74	**CC4d**	–5.11/–1.75/3.36
**BO1d**	–5.90/–1.53/4.37	**BO3c**	–5.84/–1.57/4.27	**BO4d**	–5.45/–1.78/3.67
**CC1e**	–5.29/–1.50/3.79	**CC3d**	–5.12/–1.78/3.34	**CC4e**	–5.17/–1.52/3.65
**BO1e**	–6.04/–1.51/4.53	**BO3d**	–5.76/–1.78/3.98	**BO4e**	–5.38/–1.73/3.65
**CC1f**	–5.30/–1.51/3.39	**CC3e**	–5.18/–1.51/3.67	**CC5**	–5.31/–1.44/3.87
**BO1f**	–5.92/–1.53/4.39	**BO3e**	–5.47/–1.49/3.98	**BO5**	–5.76/–1.69/4.07
**CC1g**	–5.29/–1.49/3.80			**CC6**	–5.35/–1.45/3.90
**BO1g**	–6.05/–1.50/4.55			**BO6**	–5.99/–1.65/4.34

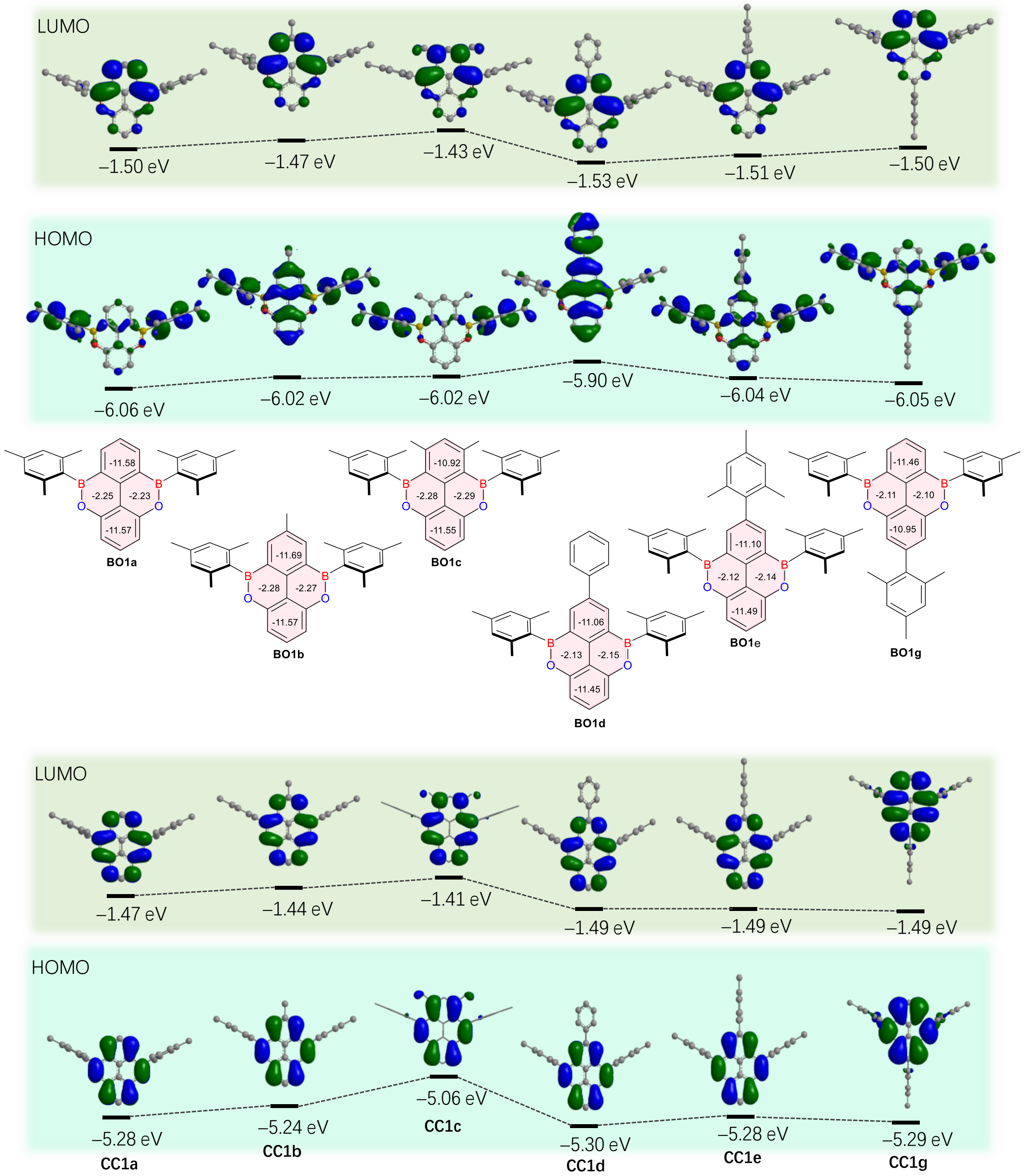

Supplementary Fig. 16. Theoretical calculation. Comparison of frontier orbital distributions, energy levels, and NICS(1) values of dBO-PAHs **BO1a**, **BO1b**, **BO1c**, **BO1d**, **BO1e** and **BO1g**, as well as their correspounding carbon-based PAHs **CC1a**, **CC1b**, **CC1c**, **CC1d**, **CC1e** and **CC1g**. The calculations were performed at B3LYP/6-31G(d) level.

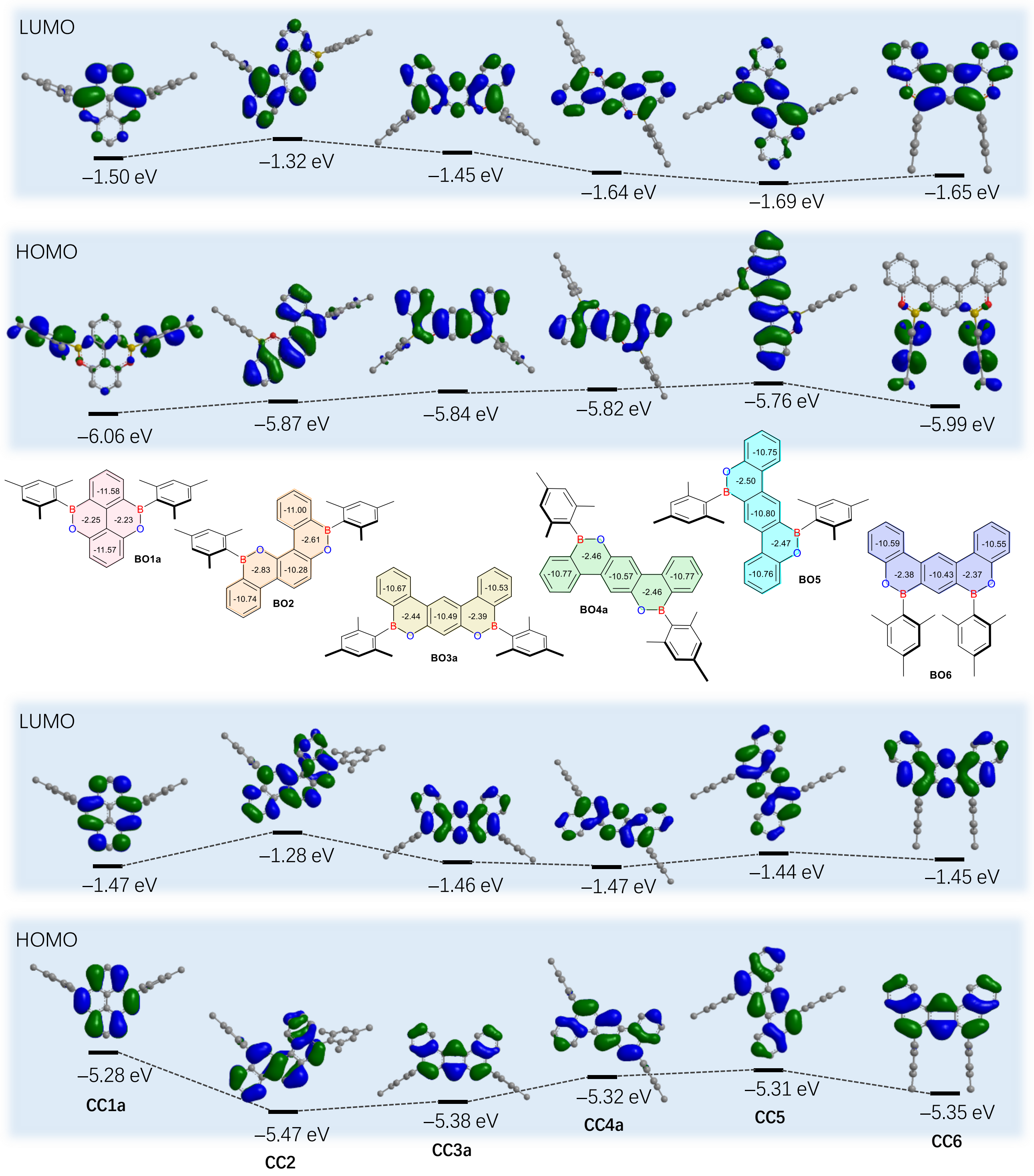

Supplementary Fig. 17. Theoretical calculation. Comparison of frontier orbital distributions, energy levels, and NICS(1) values of dBO-PAHs **BO1a**, **BO2**, **BO3a**, **BO4a**, **BO5** and **BO6**, as well as their correspounding carbon-based PAHs **CC1a**, **CC2**, **CC3a**, **CC4a**, **CC5** and **CC6**. The calculations were performed at B3LYP/6-31G(d) level.

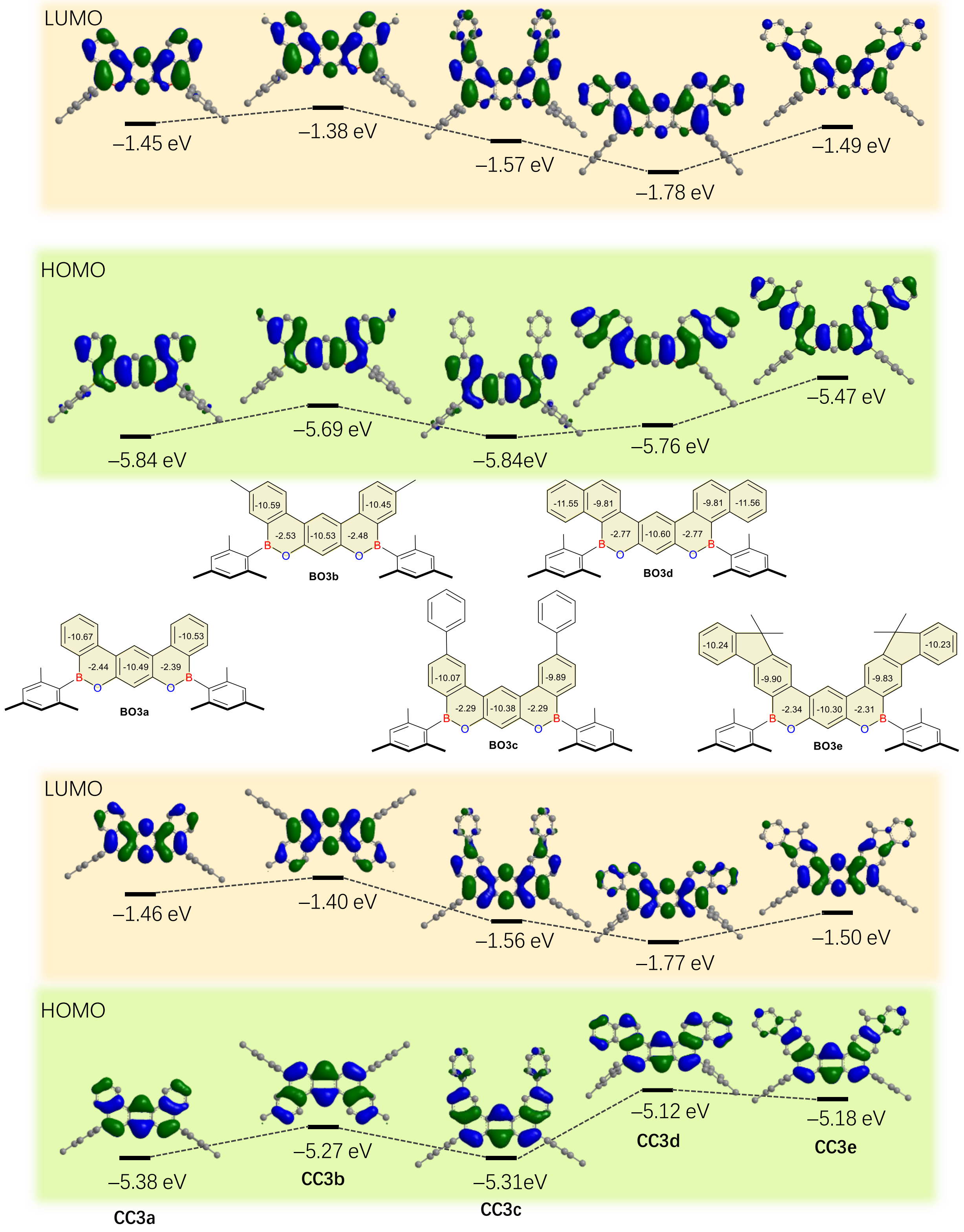

Supplementary Fig. 18. Theoretical calculation. Comparison of frontier orbital distributions, energy levels, and NICS(1) values of dBO-PAHs **BO3a**, **BO3b**, **BO3c**, **BO3d** and **BO3e**, as well as their correspounding carbon-based PAHs **CC3a**, **CC3b**, **CC3c**, **CC3d** and **CC3e**. The calculations were performed at B3LYP/6-31G(d) level.

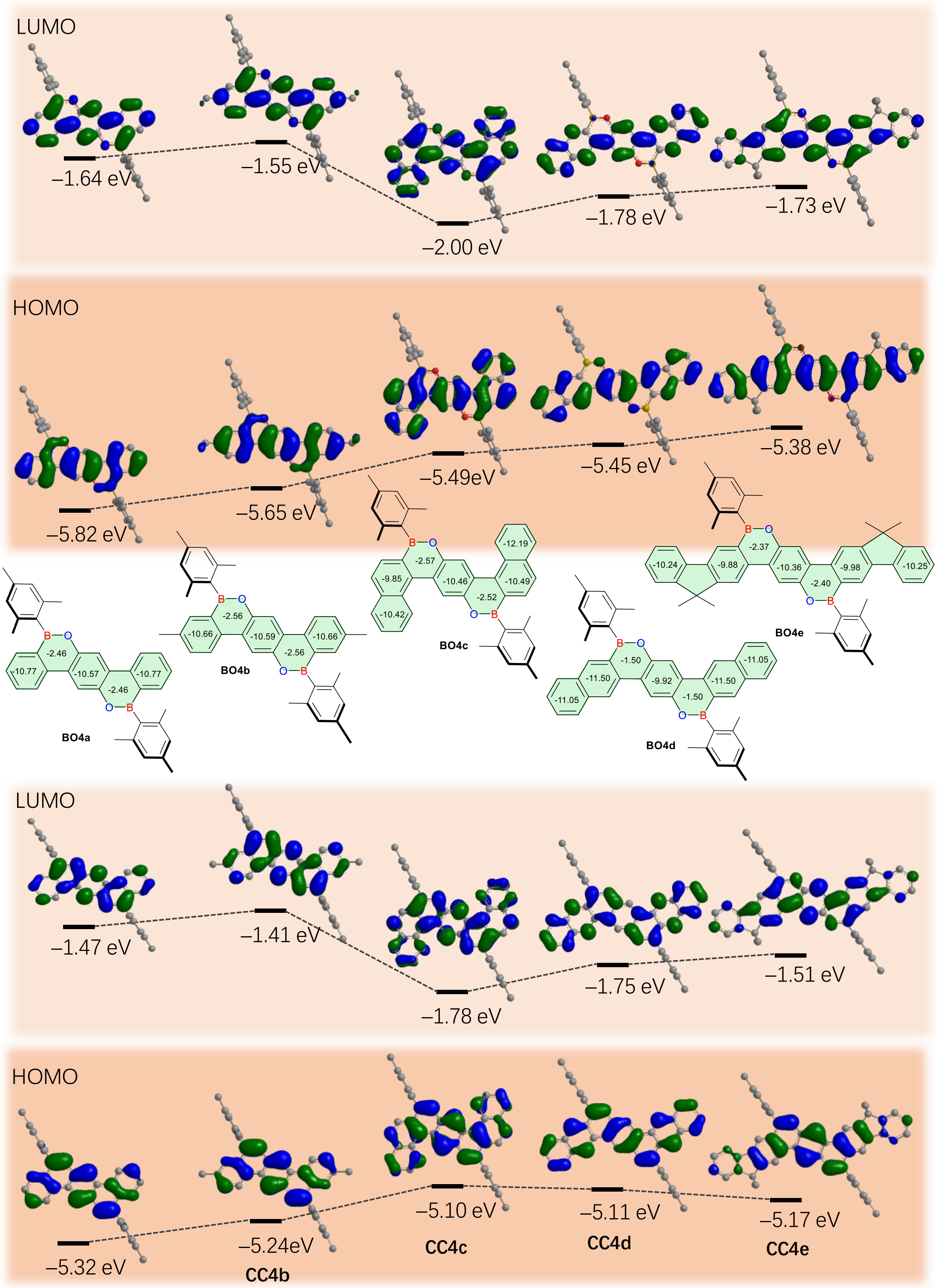

Supplementary Fig. 19. Theoretical calculation. Comparison of frontier orbital distributions, energy levels, and NICS(1) values of dBO-PAHs **BO4a**, **BO4b**, **BO4c**, **BO4d** and **BO4e**, as well as their correspounding carbon-based PAHs **CC5a**, **CC5b**, **CC5c**, **CC5d** and **CC4e**. The calculations were performed at B3LYP/6-31G(d) level.

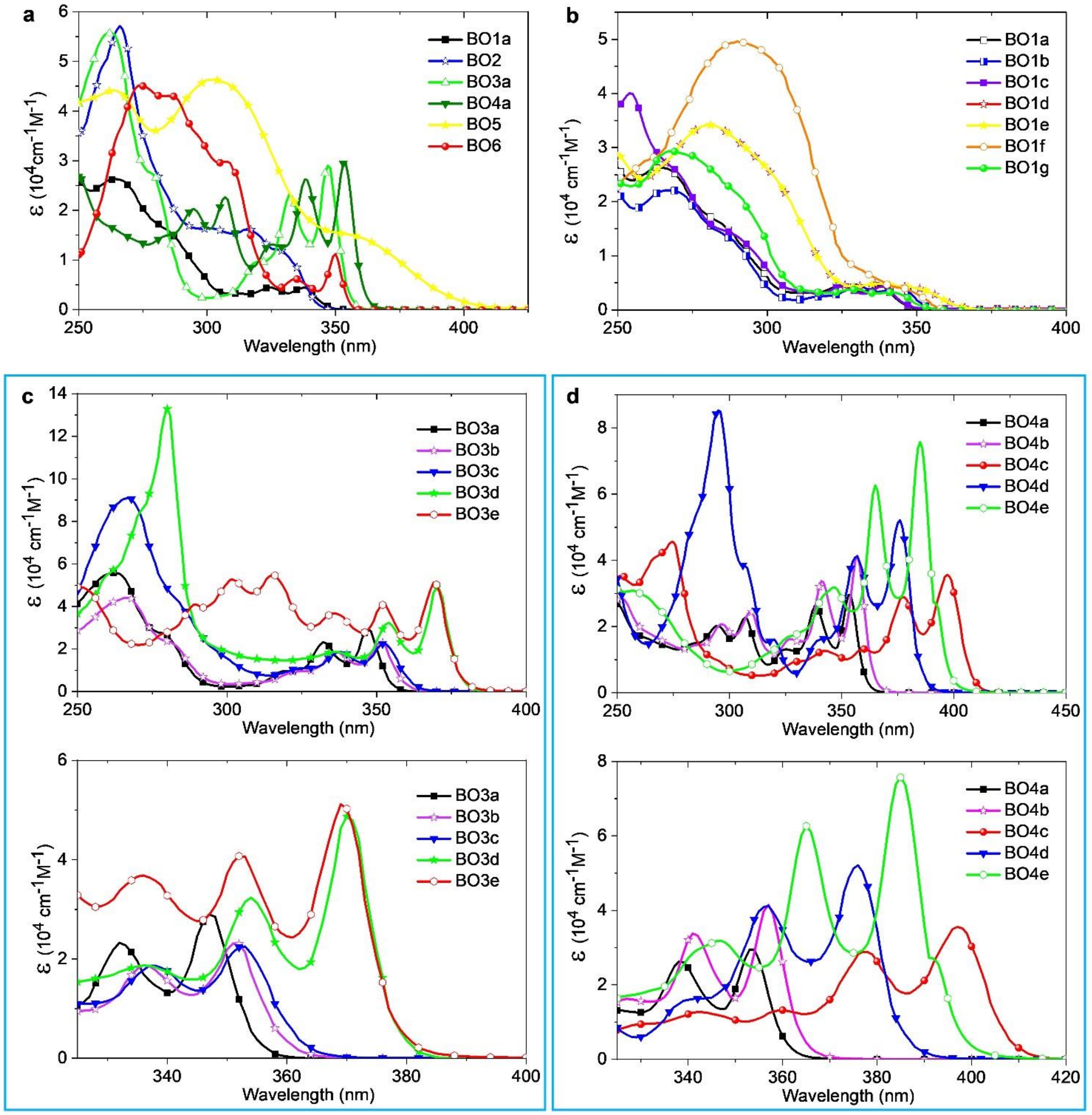

Supplementary Fig. 20. Photophysical properties. Comparison of room-temperature absorption spectra for **a**, **BO1a**, **BO2**, **BO3a**, **BO4a**, **BO5** and **BO6**; **b**, **BO1** series; **c**, **BO3** series; **d**, **BO4** series in dichloromethane solution.

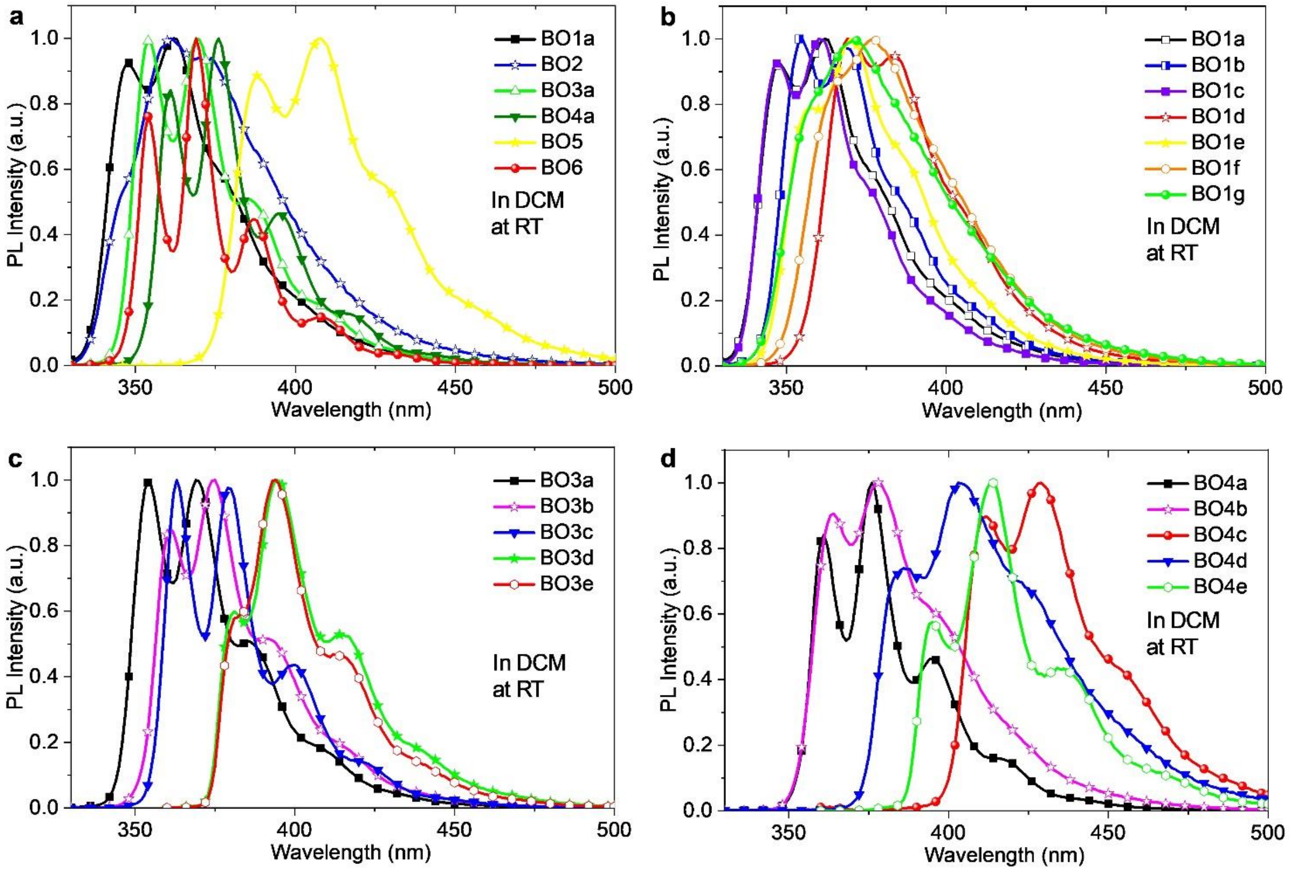

Supplementary Fig. 21. Photophysical properties. Comparison of room-temperature (RT) PL spectra for **a**, **BO1a**, **BO2**, **BO3a**, **BO4a**, **BO5** and **BO6**; **b**, **BO1** series; **c**, **BO3** series; **d**, **BO4** series in dichloromethane solution.

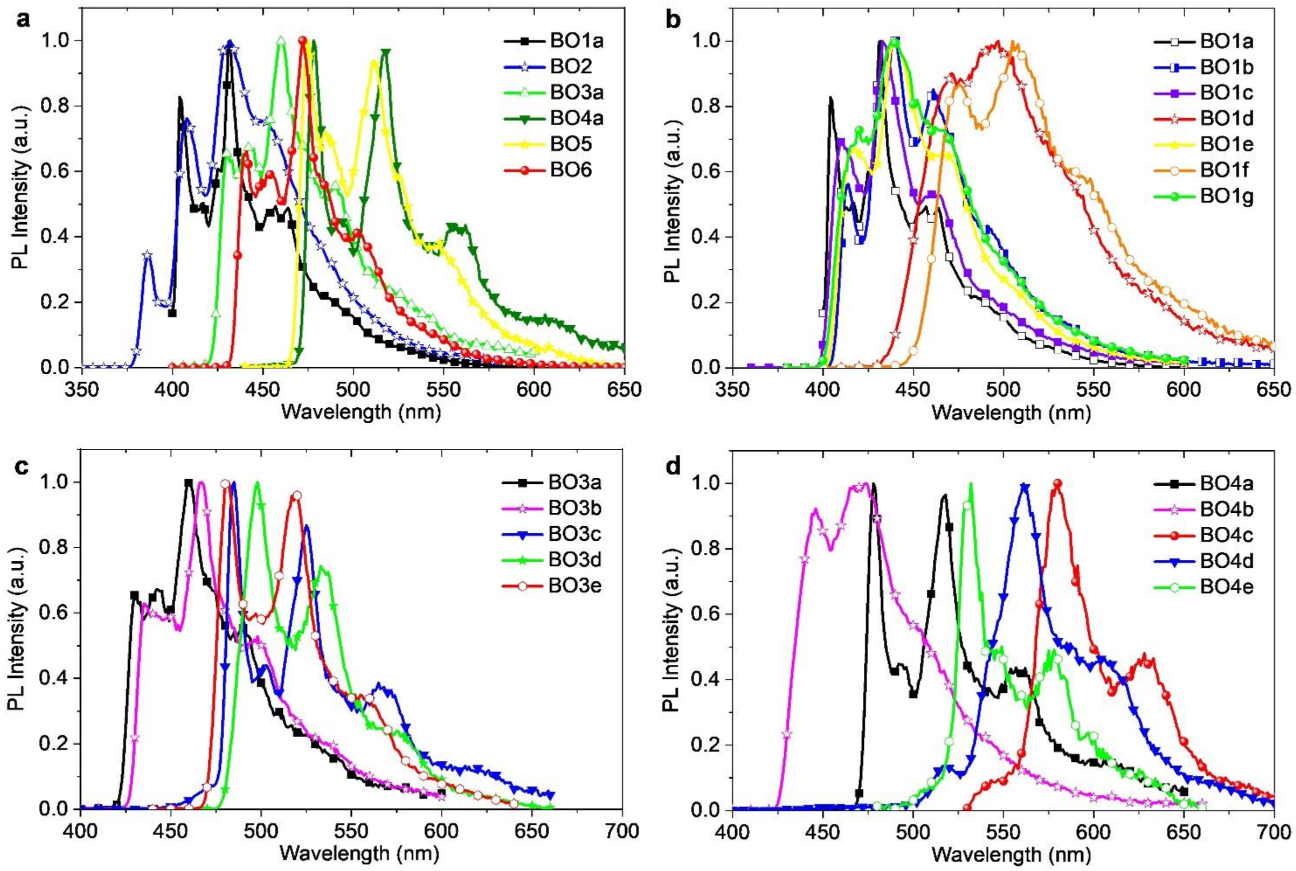

Supplementary Fig. 22. Photophysical properties. Comparison of low-temperature (77 K) phosphorescent spectra for **a**, **BO1a**, **BO2**, **BO3a**, **BO4a**, **BO5** and **BO6**; **b**, **BO1** series, **c**, **BO3** series; **d**, **BO4** series in 2-MeTHF.

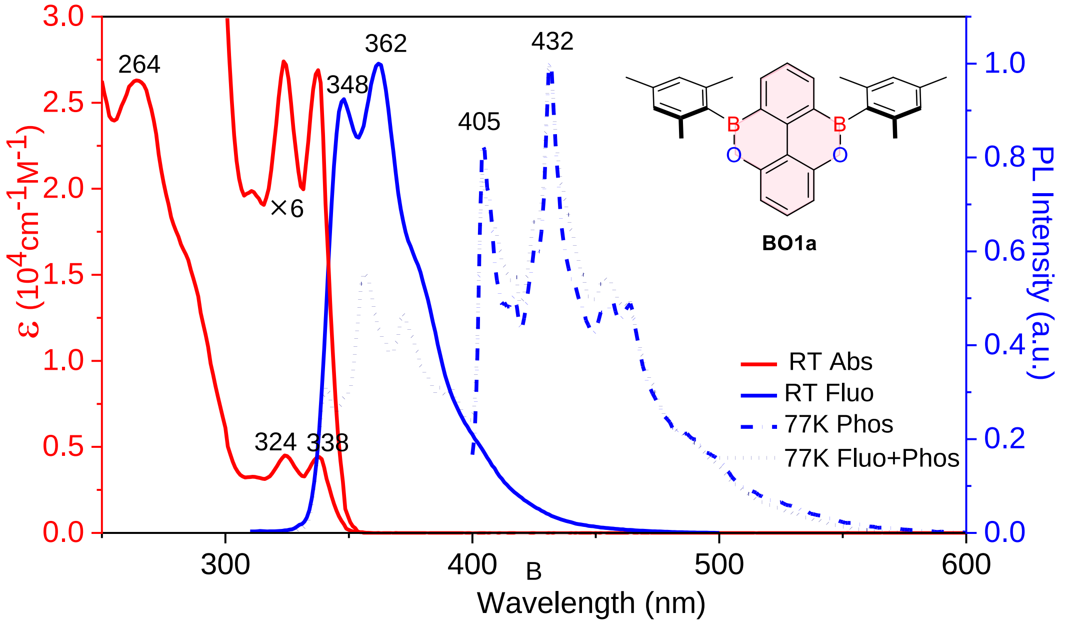

Supplementary Fig. 23. Photophysical properties. Room-temperature absorption spectrum (red solid lines) and flluorescent spectrum (blue solid line) in dichloromethane, and low-temperature (77 K) flluorescent and phosphorescent spectra (blue dash-dotted and navy dotted lines) in 2-MeTHF of **BO1a**. The chemical structure is shown in the inset.

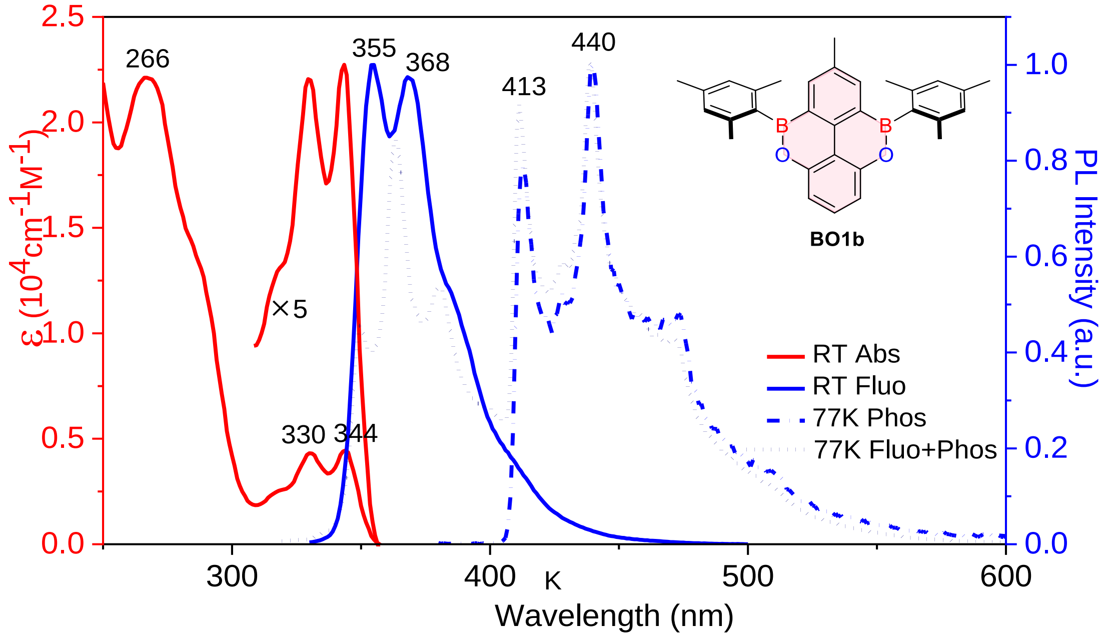

Supplementary Fig. 24. Photophysical properties. Room-temperature absorption spectrum (red solid lines) and flluorescent spectrum (blue solid line) in dichloromethane, and low-temperature (77 K) flluorescent and phosphorescent spectra (blue dash-dotted and navy dotted lines) in 2-MeTHF of **BO1b**. The chemical structure is shown in the inset.

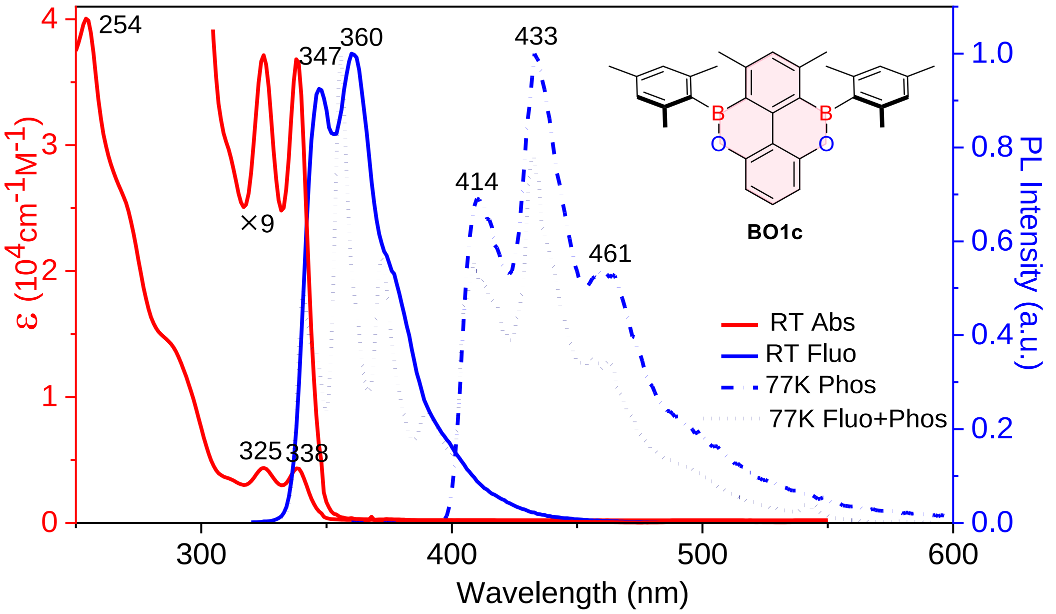

Supplementary Fig. 25. Photophysical properties. Room-temperature absorption spectrum (red solid lines) and flluorescent spectrum (blue solid line) in dichloromethane, and low-temperature (77 K) flluorescent and phosphorescent spectra (blue dash-dotted and navy dotted lines) in 2-MeTHF of **BO1c**. The chemical structure is shown in the inset.

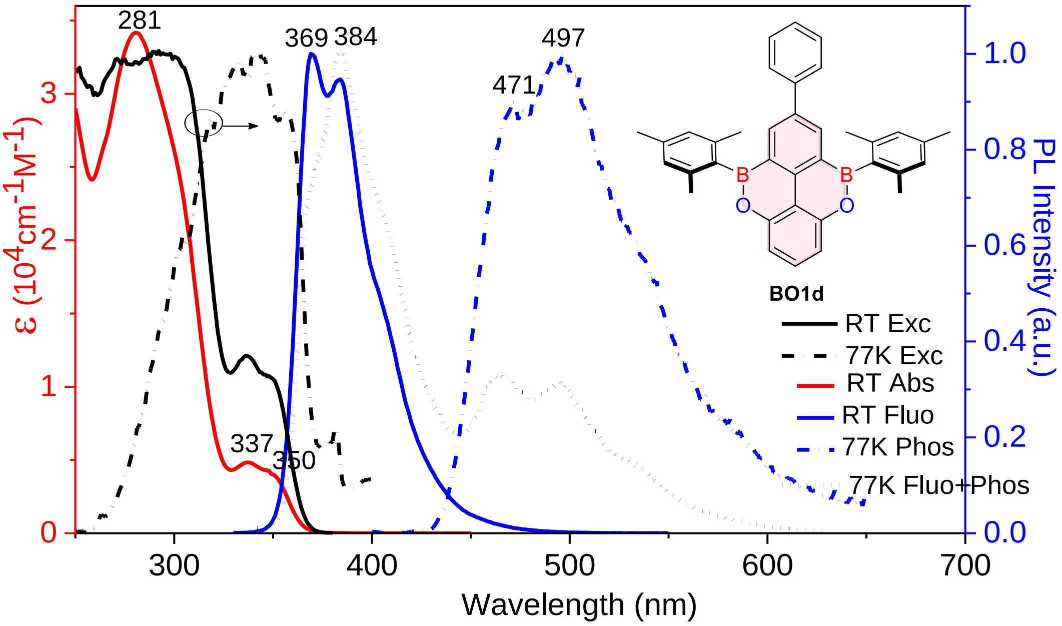

Supplementary Fig. 26. Photophysical properties. Room-temperature absorption spectrum (red solid lines) and flluorescent spectrum (blue solid line) in dichloromethane, and low-temperature (77 K) flluorescent and phosphorescent spectra (blue dash-dotted and navy dotted lines) in 2-MeTHF and their corresponding excited spectra of **BO1d**. The chemical structure is shown in the inset.

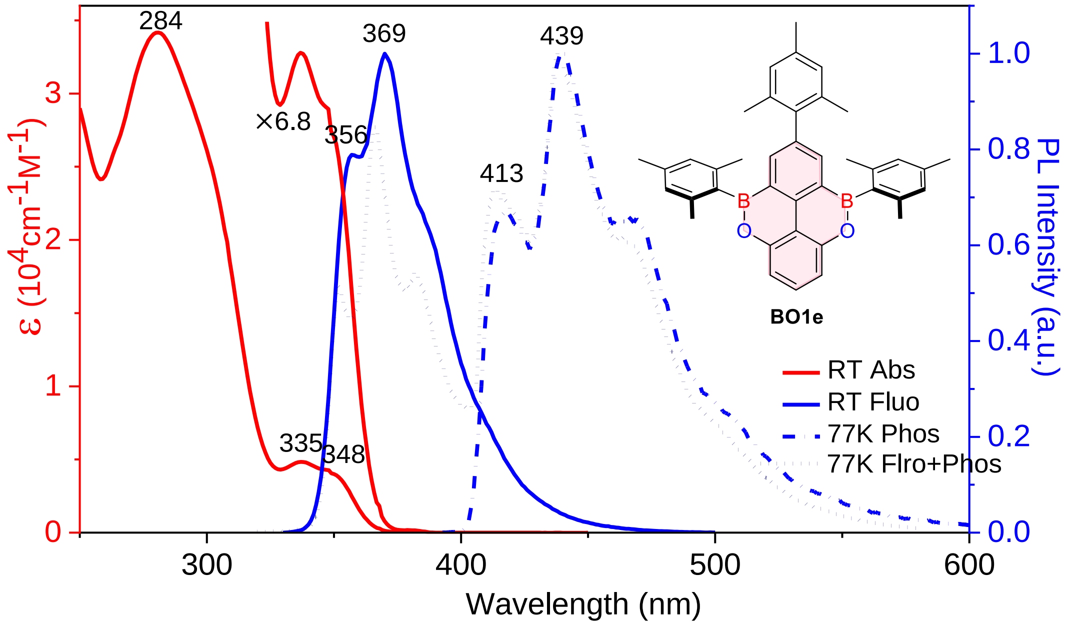

Supplementary Fig. 27. Photophysical properties. Room-temperature absorption spectrum (red solid lines) and flluorescent spectrum (blue solid line) in dichloromethane, and low-temperature (77 K) flluorescent and phosphorescent spectra (blue dash-dotted and navy dotted lines) in 2-MeTHF of **BO1e**. The chemical structure is shown in the inset.

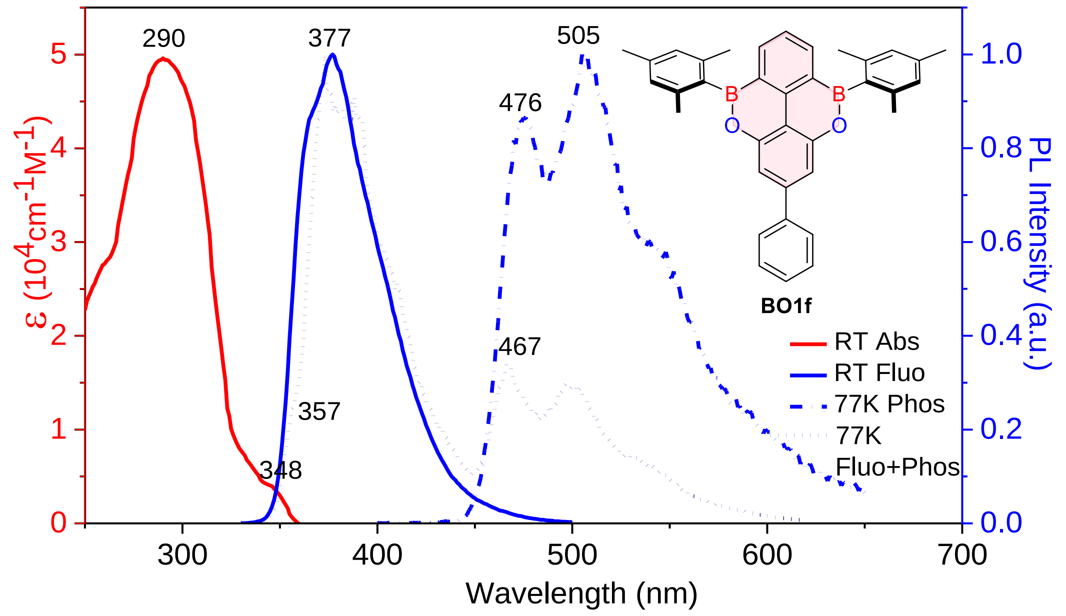

Supplementary Fig. 28. Photophysical properties. Room-temperature absorption spectrum (red solid lines) and flluorescent spectrum (blue solid line) in dichloromethane, and low-temperature (77 K) flluorescent and phosphorescent spectra (blue dash-dotted and navy dotted lines) in 2-MeTHF of **BO1f**. The chemical structure is shown in the inset.

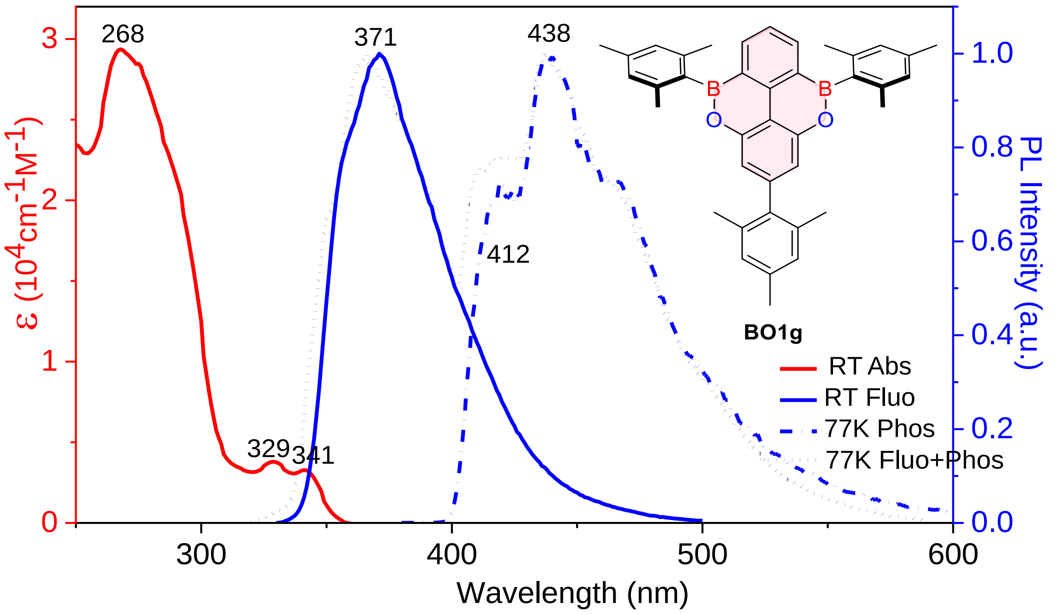

Supplementary Fig. 29. Photophysical properties. Room-temperature absorption spectrum (red solid lines) and flluorescent spectrum (blue solid line) in dichloromethane, and low-temperature (77 K) flluorescent and phosphorescent spectra (blue dash-dotted and navy dotted lines) in 2-MeTHF of **BO1g**. The chemical structure is shown in the inset.

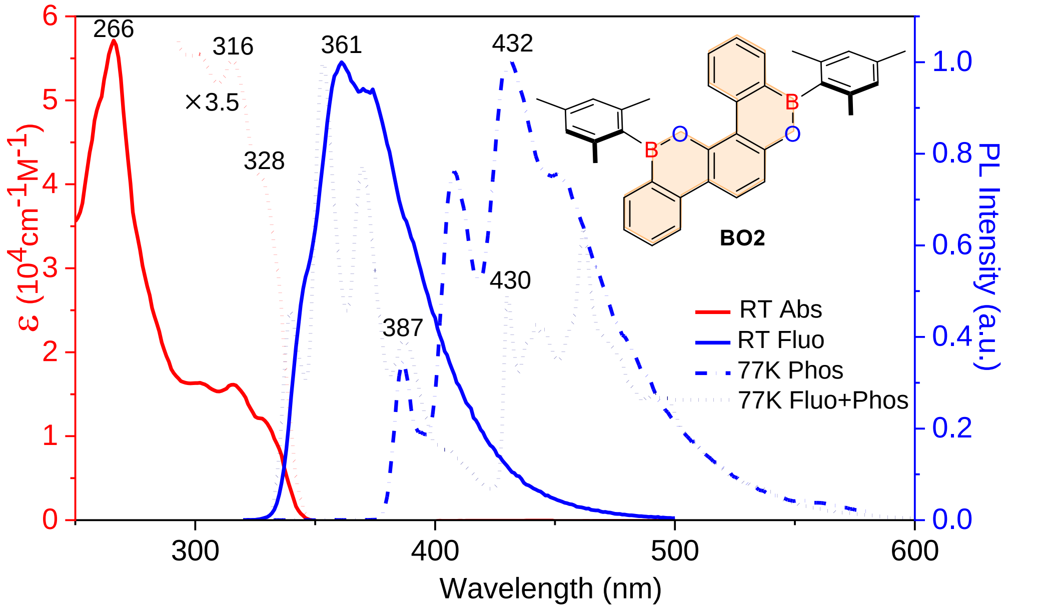

Supplementary Fig. 30. Photophysical properties. Room-temperature absorption spectrum (red solid lines) and flluorescent spectrum (blue solid line) in dichloromethane, and low-temperature (77 K) flluorescent and phosphorescent spectra (blue dash-dotted and navy dotted lines) in 2-MeTHF of **BO2**. The chemical structure is shown in the inset.

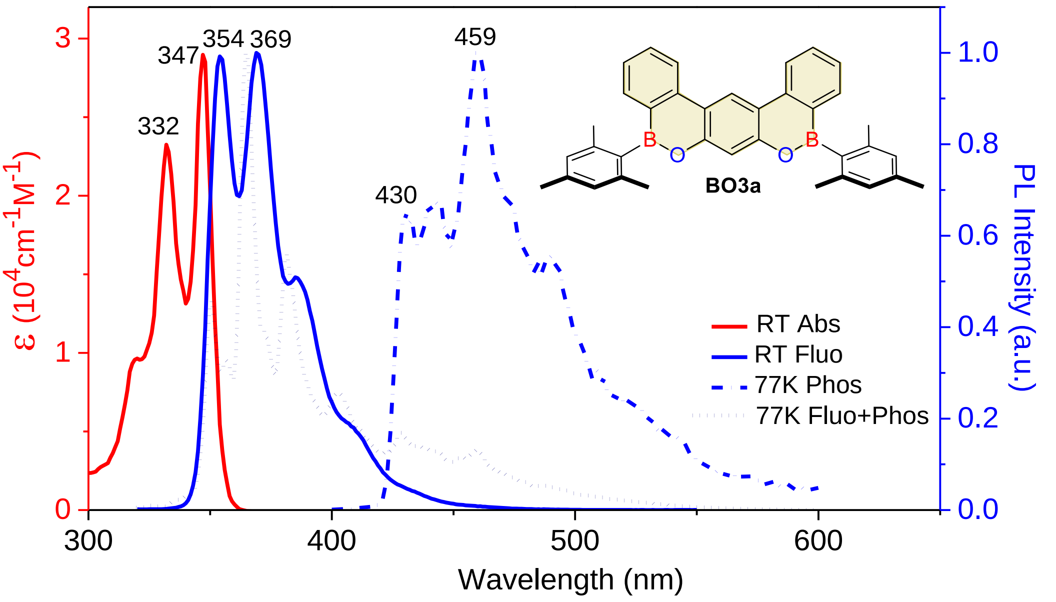

Supplementary Fig. 31. Photophysical properties. Room-temperature absorption spectrum (red solid lines) and flluorescent spectrum (blue solid line) in dichloromethane, and low-temperature (77 K) flluorescent and phosphorescent spectra (blue dash-dotted and navy dotted lines) in 2-MeTHF of **BO3a**. The chemical structure is shown in the inset.

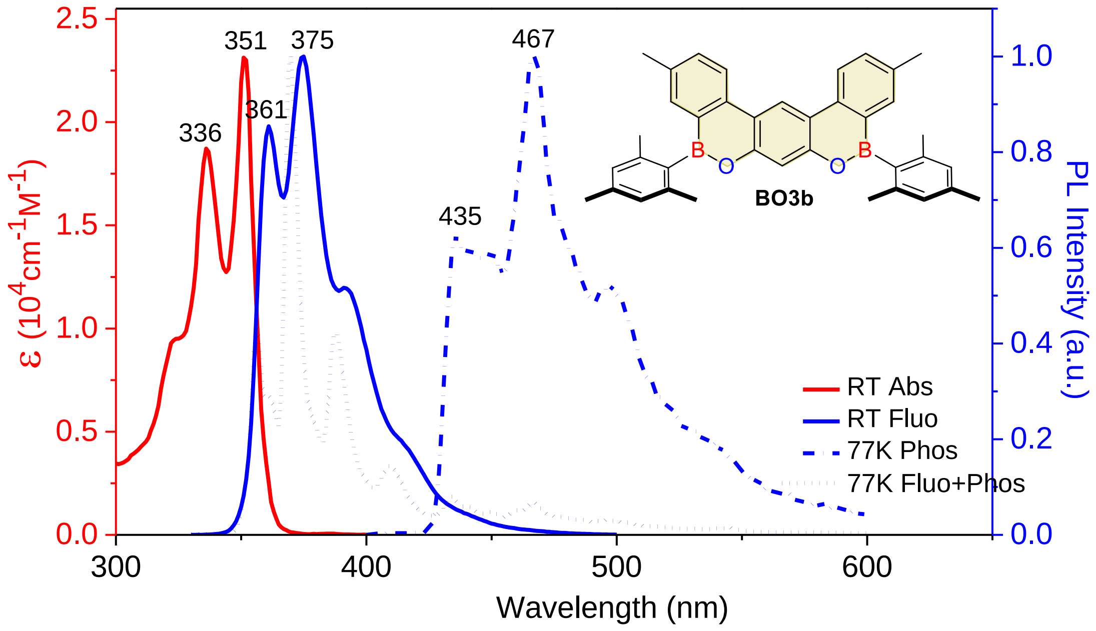

Supplementary Fig. 32. Photophysical properties. Room-temperature absorption spectrum (red solid lines) and flluorescent spectrum (blue solid line) in dichloromethane, and low-temperature (77 K) flluorescent and phosphorescent spectra (blue dash-dotted and navy dotted lines) in 2-MeTHF of **BO3b**. The chemical structure is shown in the inset.

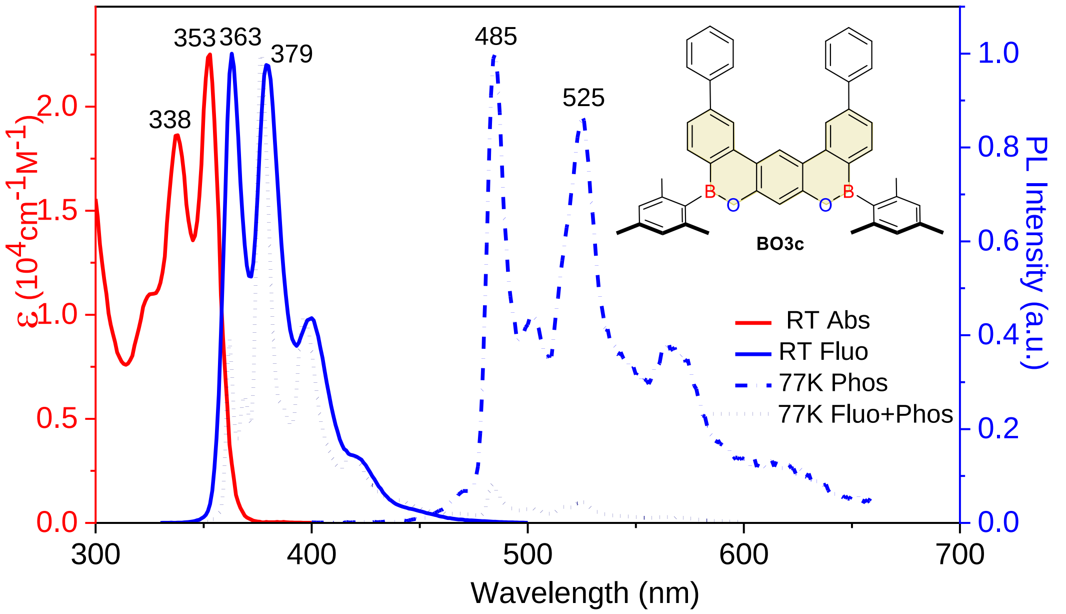

Supplementary Fig. 33. Photophysical properties. Room-temperature absorption spectrum (red solid lines) and flluorescent spectrum (blue solid line) in dichloromethane, and low-temperature (77 K) flluorescent and phosphorescent spectra (blue dash-dotted and navy dotted lines) in 2-MeTHF of **BO3c**. The chemical structure is shown in the inset.

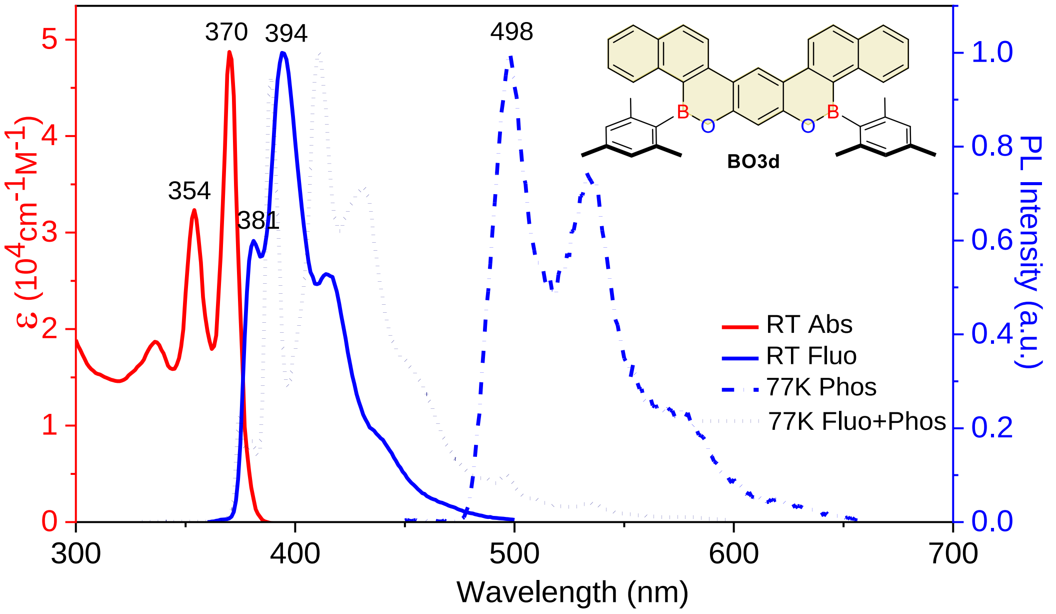

Supplementary Fig. 34. Photophysical properties. Room-temperature absorption spectrum (red solid lines) and flluorescent spectrum (blue solid line) in dichloromethane, and low-temperature (77 K) flluorescent and phosphorescent spectra (blue dash-dotted and navy dotted lines) in 2-MeTHF of **BO3d**. The chemical structure is shown in the inset.

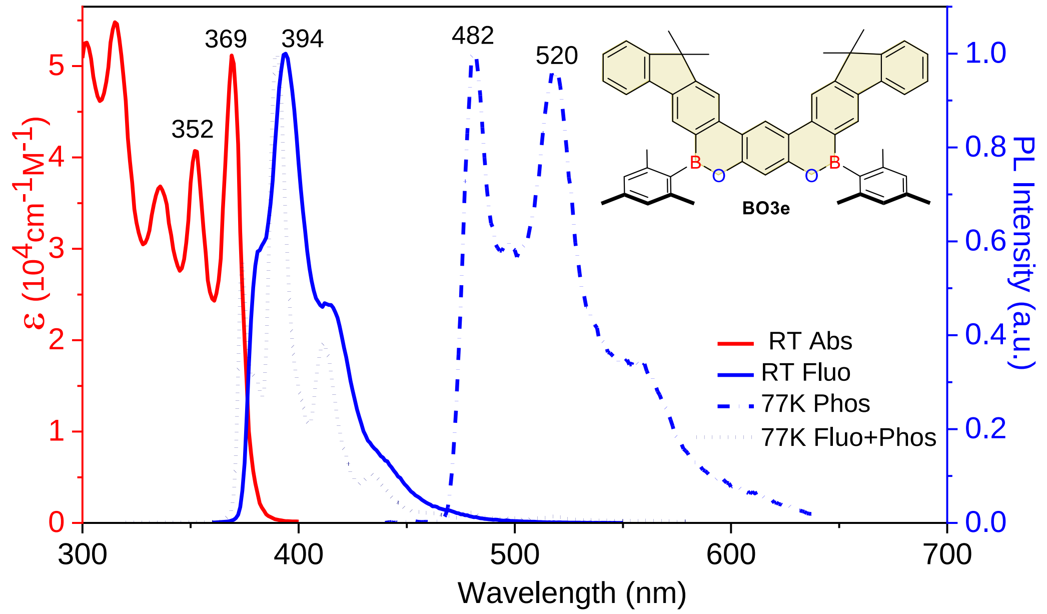

Supplementary Fig. 35. Photophysical properties. Room-temperature absorption spectrum (red solid lines) and flluorescent spectrum (blue solid line) in dichloromethane, and low-temperature (77 K) flluorescent and phosphorescent spectra (blue dash-dotted and navy dotted lines) in 2-MeTHF of **BO3e**. The chemical structure is shown in the inset.

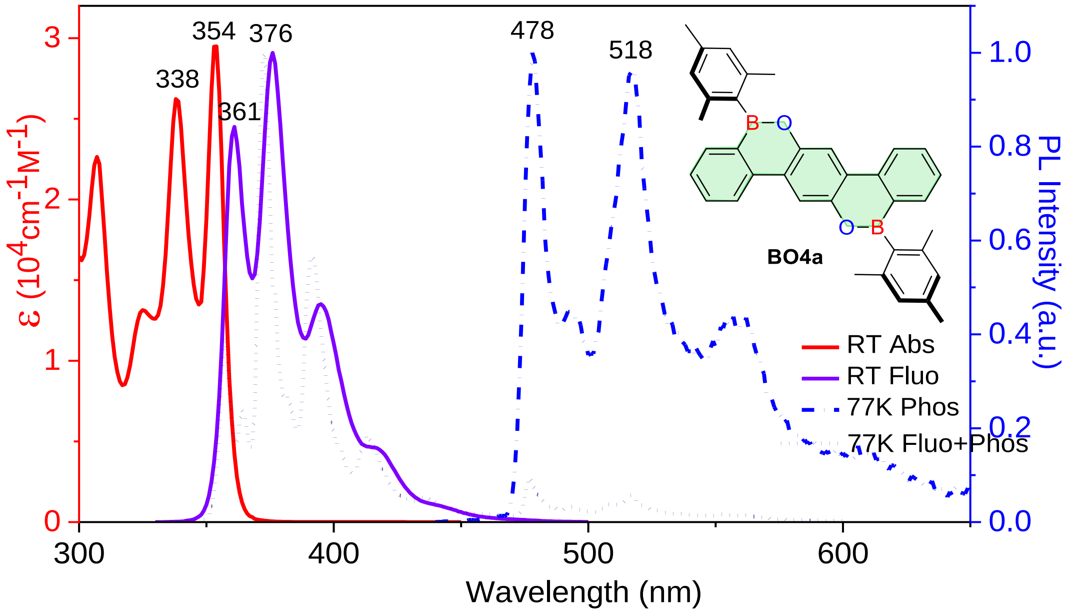

Supplementary Fig. 36. Photophysical properties. Room-temperature absorption spectrum (red solid lines) and flluorescent spectrum (blue solid line) in dichloromethane, and low-temperature (77 K) flluorescent and phosphorescent spectra (blue dash-dotted and navy dotted lines) in 2-MeTHF of **BO4a**. The chemical structure is shown in the inset.

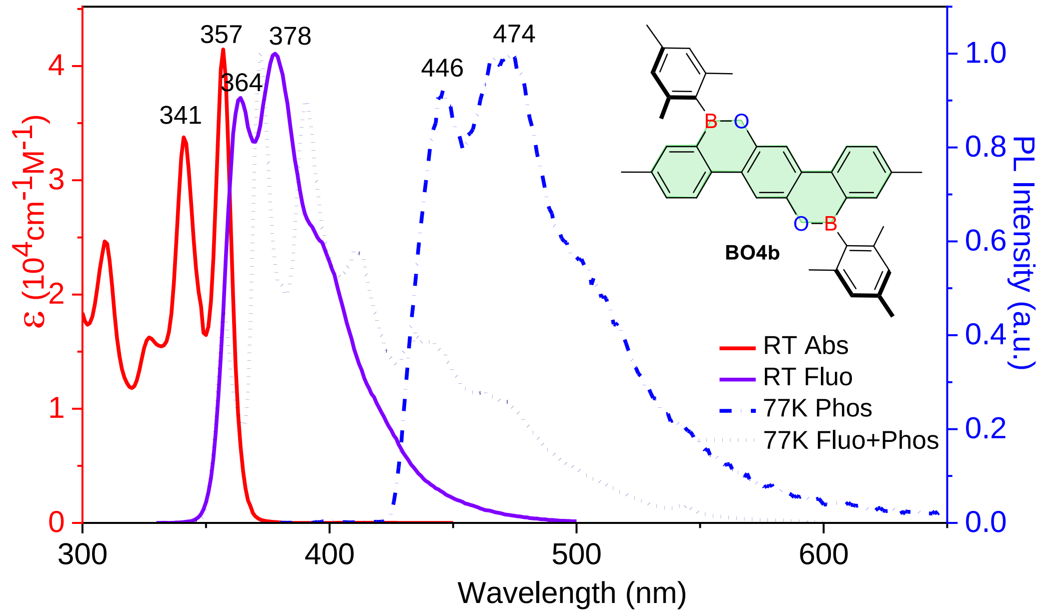

Supplementary Fig. 37. Photophysical properties. Room-temperature absorption spectrum (red solid lines) and flluorescent spectrum (blue solid line) in dichloromethane, and low-temperature (77 K) flluorescent and phosphorescent spectra (blue dash-dotted and navy dotted lines) in 2-MeTHF of **BO4b**. The chemical structure is shown in the inset.

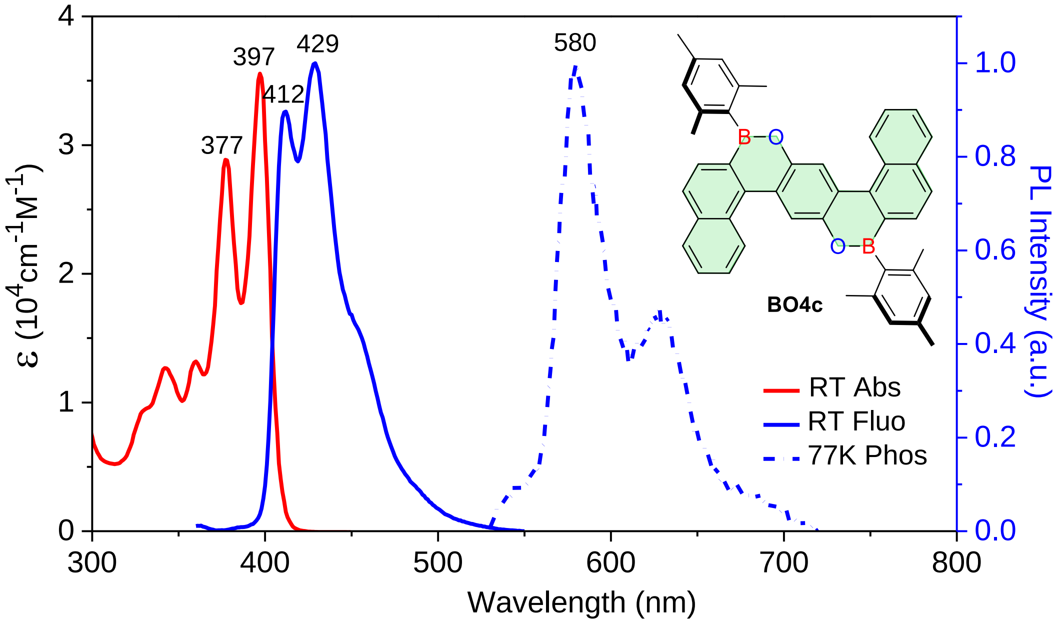

Supplementary Fig. 38. Photophysical properties. Room-temperature absorption spectrum (red solid line) and luminescent spectrum in dichloromethane (blue solid line), and low-temperature (77 K) phosphorescent spectrum (blue dash-dotted line) in 2-MeTHF of **BO4c**. The chemical structure is shown in the inset.

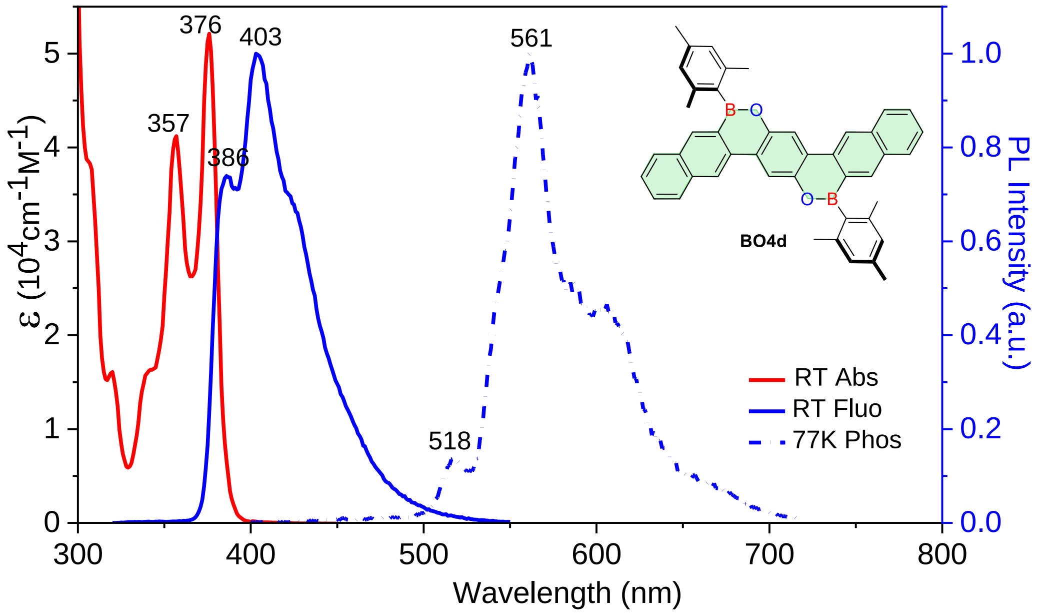

Supplementary Fig. 39. Photophysical properties. Room-temperature absorption spectrum (red solid line) and luminescent spectrum in dichloromethane (blue solid line), and low-temperature (77 K) phosphorescent spectrum (blue dash-dotted line) in 2-MeTHF of **BO4d**. The chemical structure is shown in the inset.

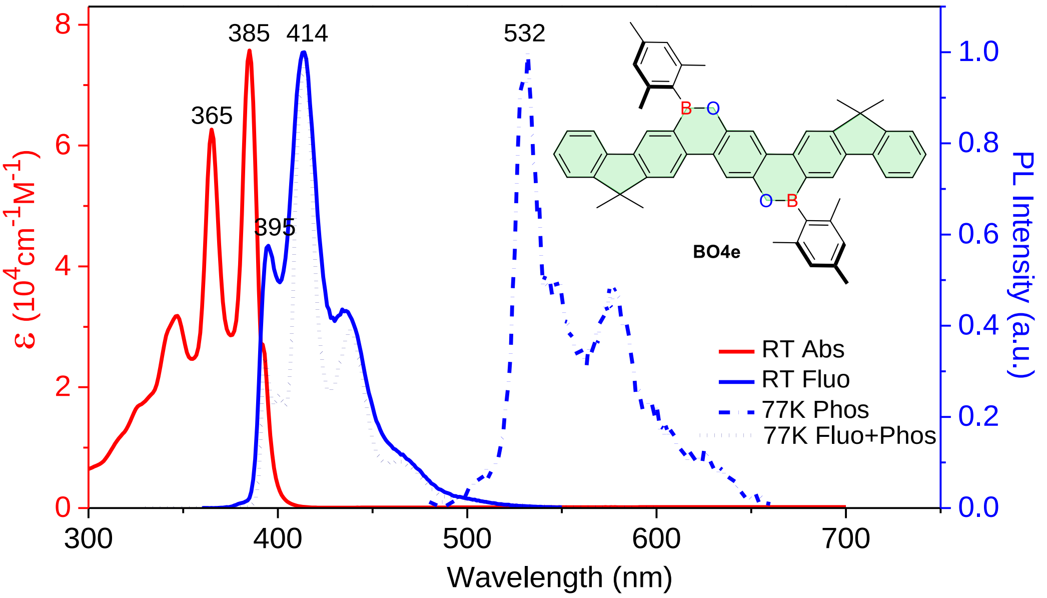

Supplementary Fig. 40. Photophysical properties. Room-temperature absorption spectrum (red solid lines) and flluorescent spectrum (blue solid line) in dichloromethane, and low-temperature (77 K) flluorescent and phosphorescent spectra (blue dash-dotted and navy dotted lines) in 2-MeTHF of **BO4e**. The chemical structure is shown in the inset.

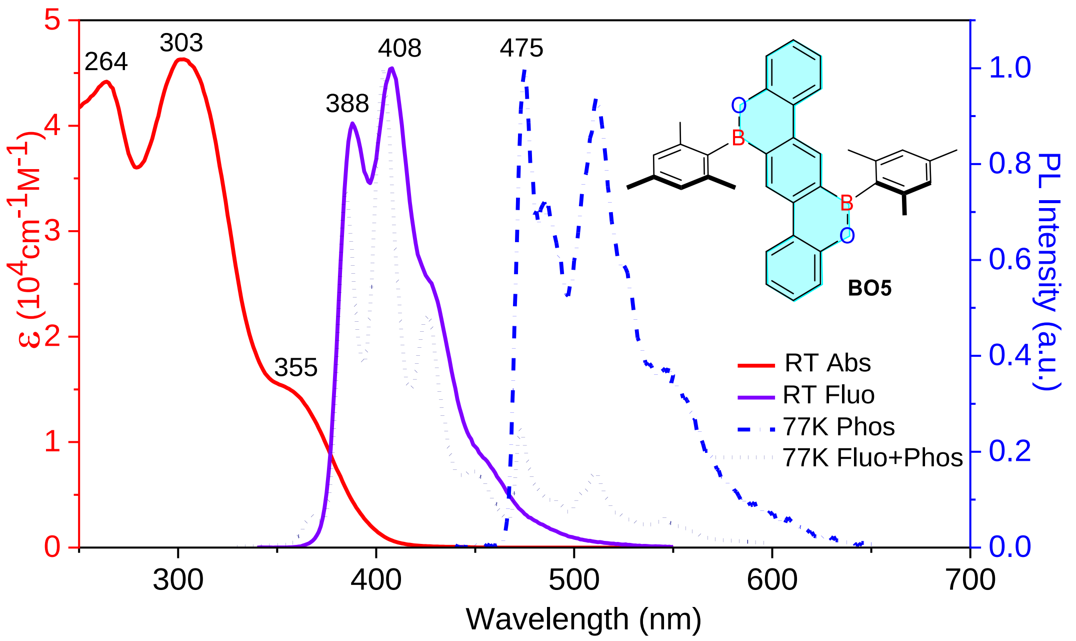

Supplementary Fig. 41. Photophysical properties. Room-temperature absorption spectrum (red solid lines) and flluorescent spectrum (blue solid line) in dichloromethane, and low-temperature (77 K) flluorescent and phosphorescent spectra (blue dash-dotted and navy dotted lines) in 2-MeTHF of **BO5**. The chemical structure is shown in the inset.

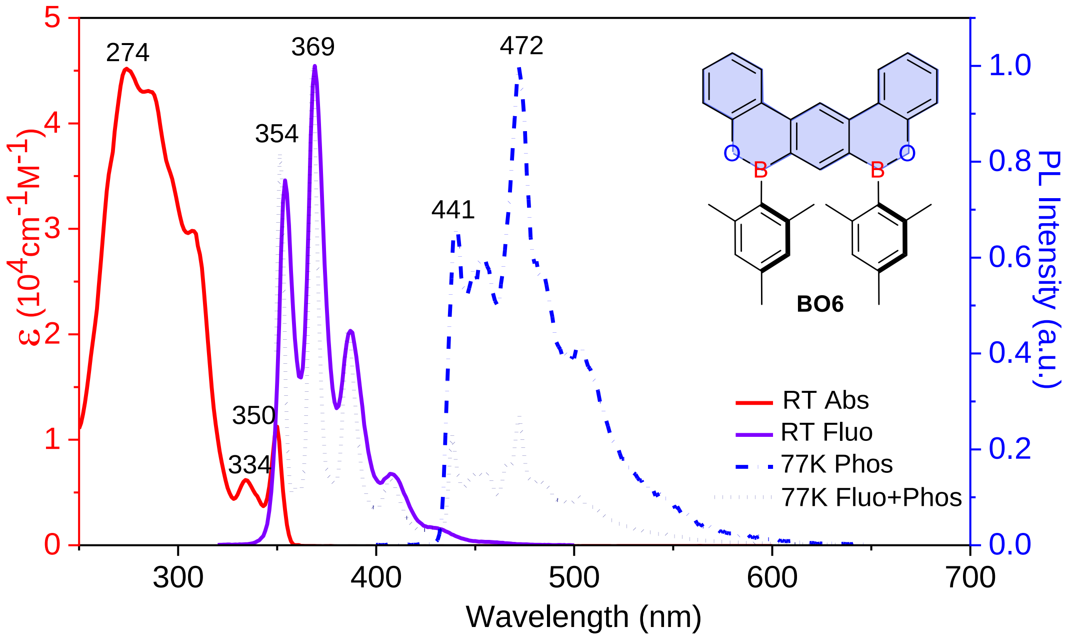

Supplementary Fig. 42. Photophysical properties. Room-temperature absorption spectrum (red solid lines) and flluorescent spectrum (blue solid line) in dichloromethane, and low-temperature (77 K) flluorescent and phosphorescent spectra (blue dash-dotted and navy dotted lines) in 2-MeTHF of **BO6**. The chemical structure is shown in the inset.

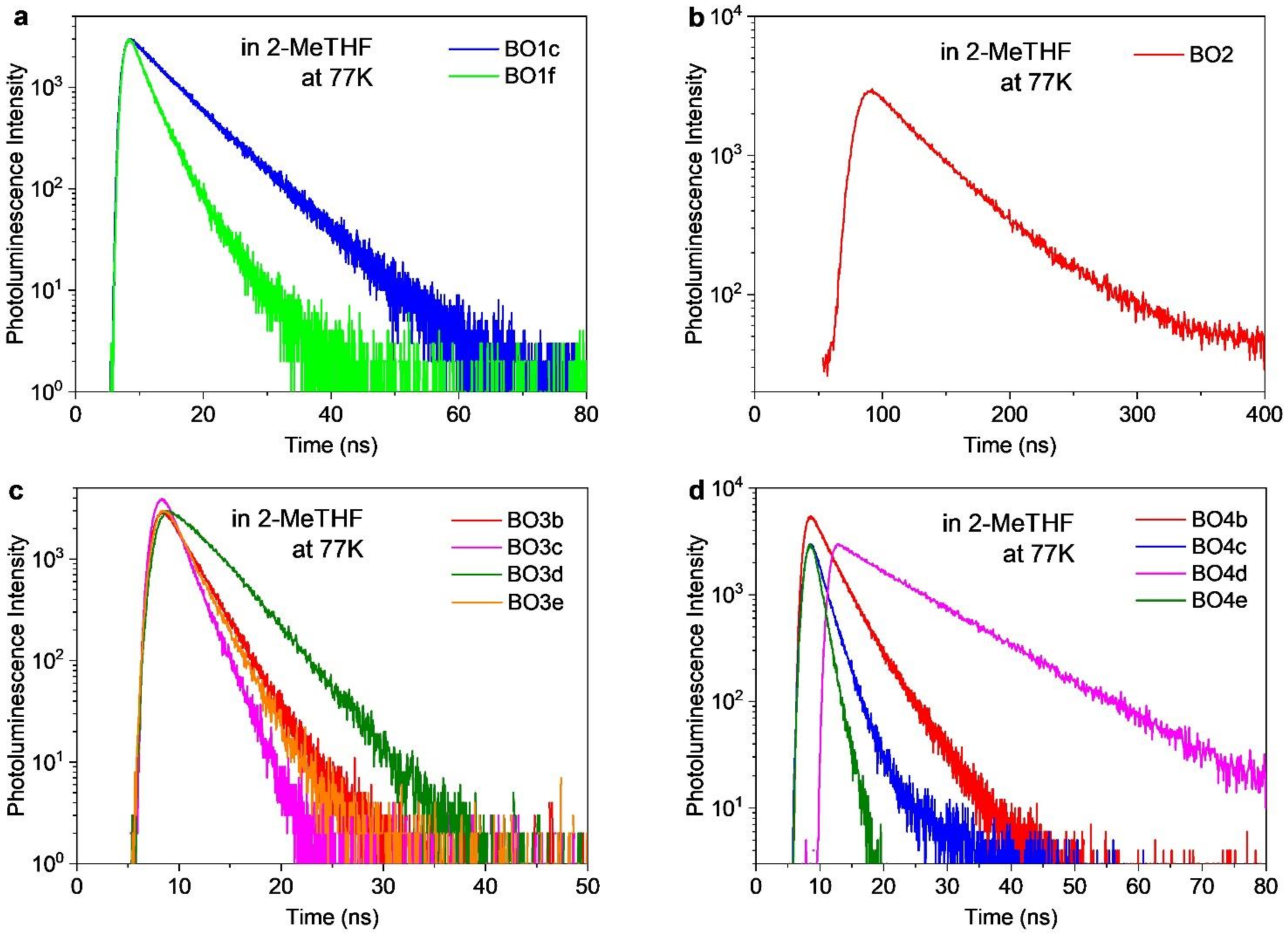

Supplementary Fig. 43. Photophysical properties. Selected PL decay of **a**, **BO1c**, **BO1f**; **b**, **BO2**, **c**, **BO3b**, **BO3c**, **BO3d**, **BO3e**; **d**, **BO4b**, **BO4c**, **BO4d**, **BO4e** in 2-MeTHF at 77 K excited at their corresponding first fluorescent peak.

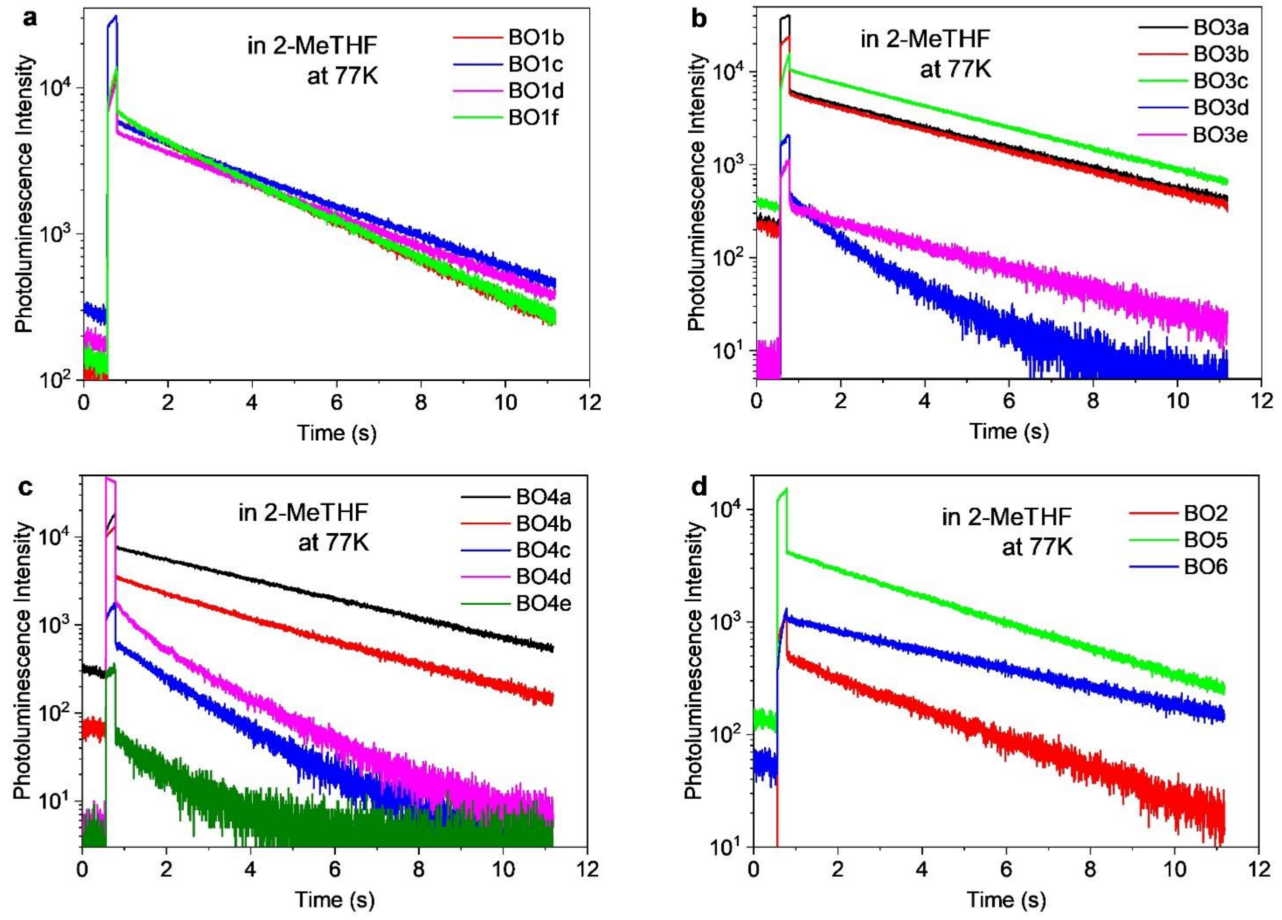

Supplementary Fig. 44. Photophysical properties. Selected PL decay of **a**, **BO1b**, **BO1c**, **BO1d**, **BO1f**; **b**, **BO3a**, **BO3b**, **BO3c**, **BO3d**, **BO3e**; **c**, **BO4a**, **BO4b**, **BO4c**, **BO4d**, **BO4e**; **d**, **BO2**, **BO5**, **BO6** in 2-MeTHF at 77 K excited at their corresponding first phosphorescent peak.

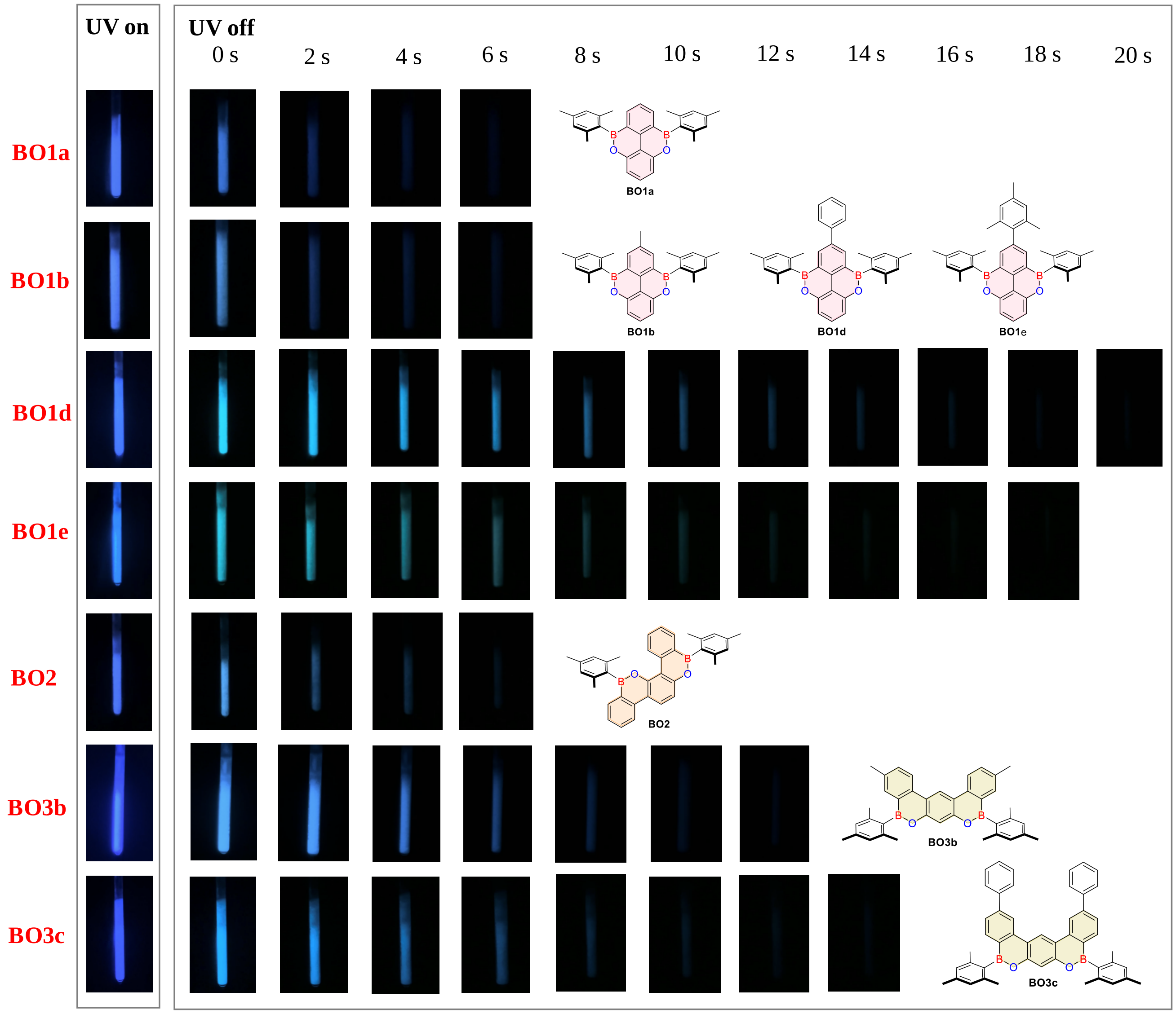

Supplementary Fig. 45. Ultralong phosphorescence. Ultralong phosphorescent photographs of **BO1a**, **BO1b**, **BO1d**, **BO1e**, **BO2**, **BO3b** and **BO3c** at 77K in THF.

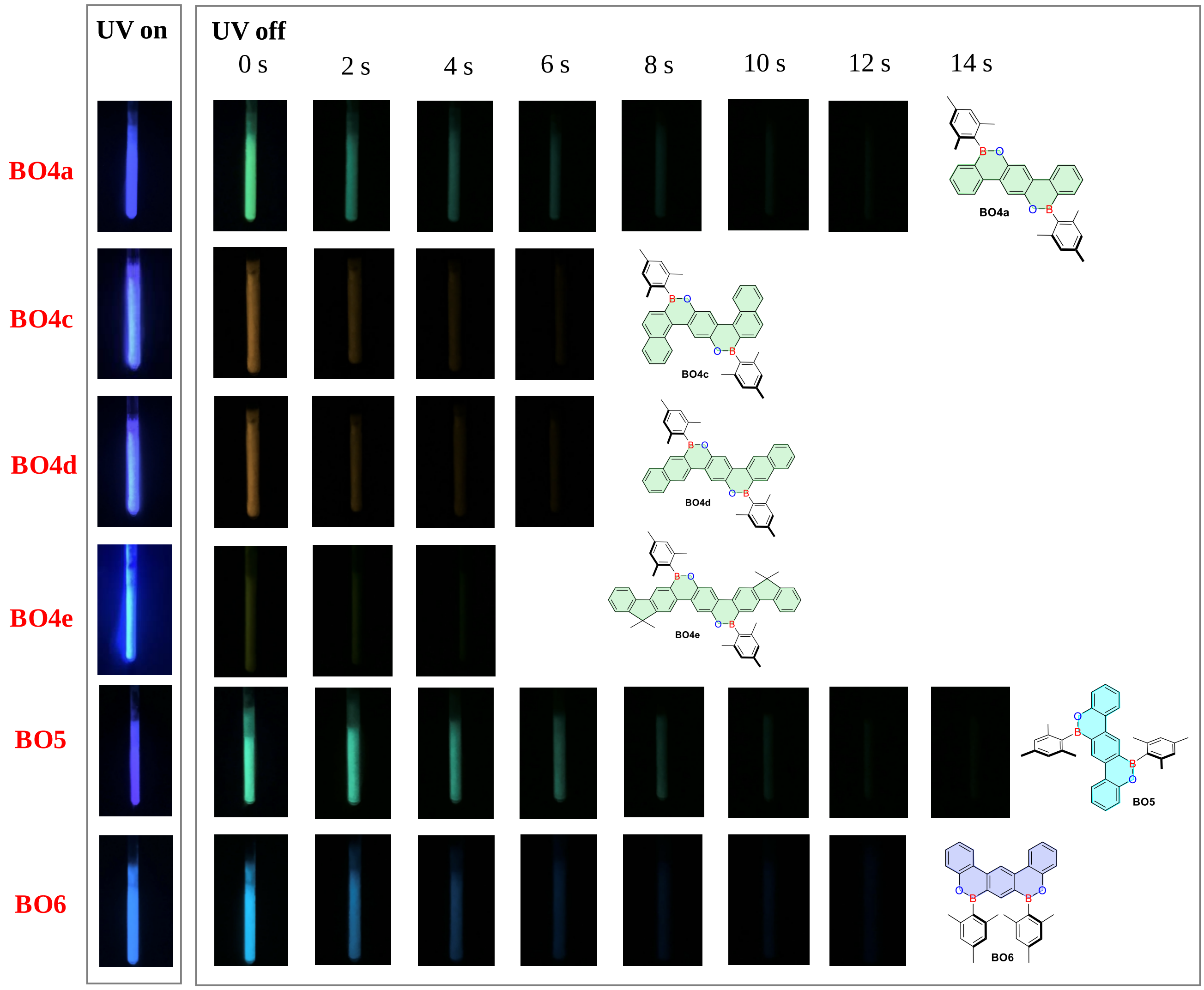

Supplementary Fig. 46. Ultralong phosphorescence. Ultralong phosphorescent photographs of **BO4a**, **BO4c**, **BO4d**, **BO4e**, **BO5** and **BO6** at 77K in THF.

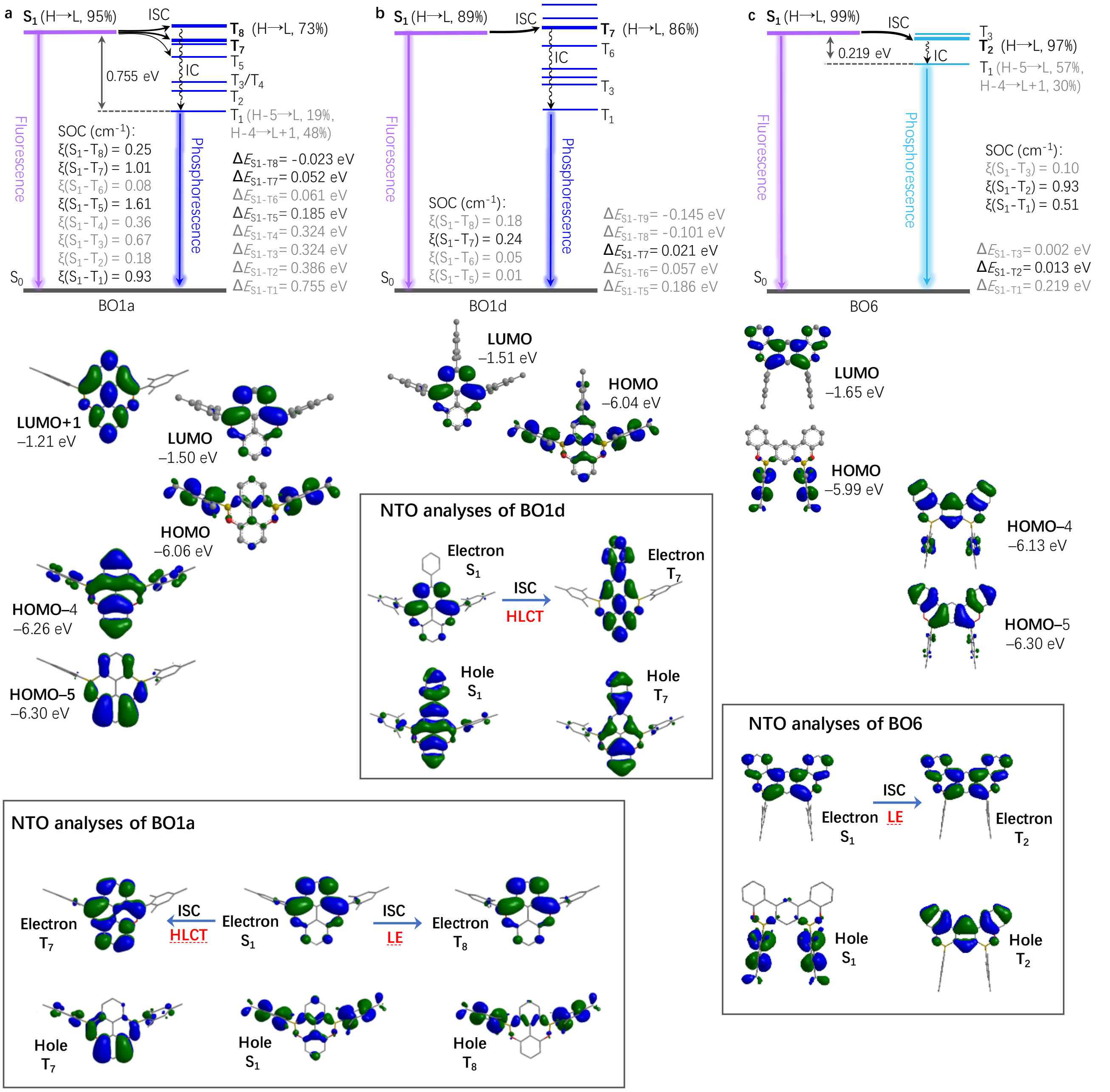

Supplementary Fig. 47. Theoretical calculation. TD-DFT calculated singlet and triplet energy levels, main transition configurations, spin–orbit coupling (SOC) values and natural transition orbital (NTO) analyses of **a**, **BO1a**; **b**, **BO1d; c**, **BO6** at B3LYP/6-31G(d) level based on optimized S_0 geometry. Selected frontier orbital distributions and energy levels are also illustrated.

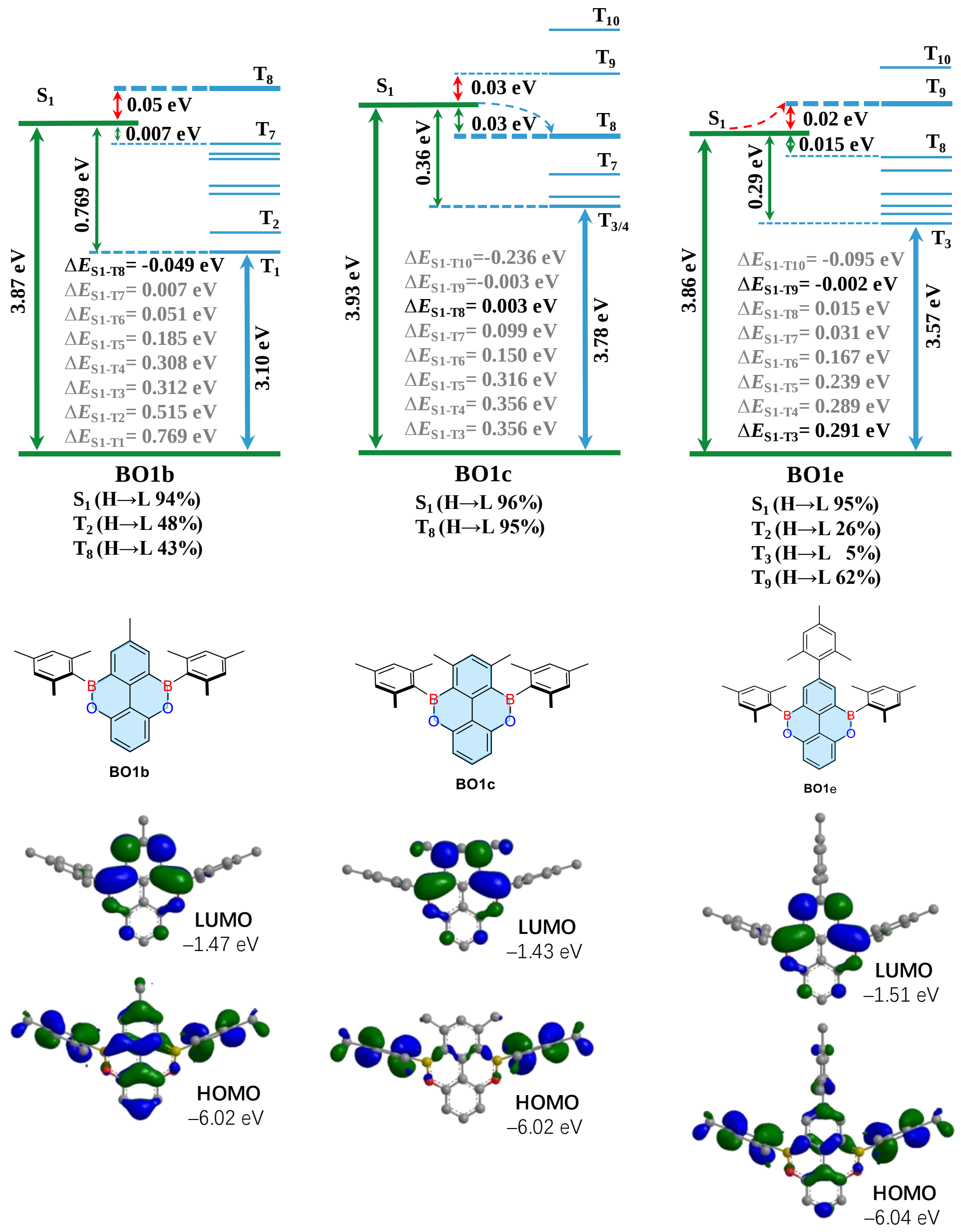

Supplementary Fig. 48. Theoretical calculation. TD-DFT calculated singlet and triplet energy levels, main transition configurations of **BO1b**, **BO1c** and **BO1e** at B3LYP/6-31G(d) level based on optimized S_0 geometry. Selected frontier orbital distributions and energy levels are also illustrated.

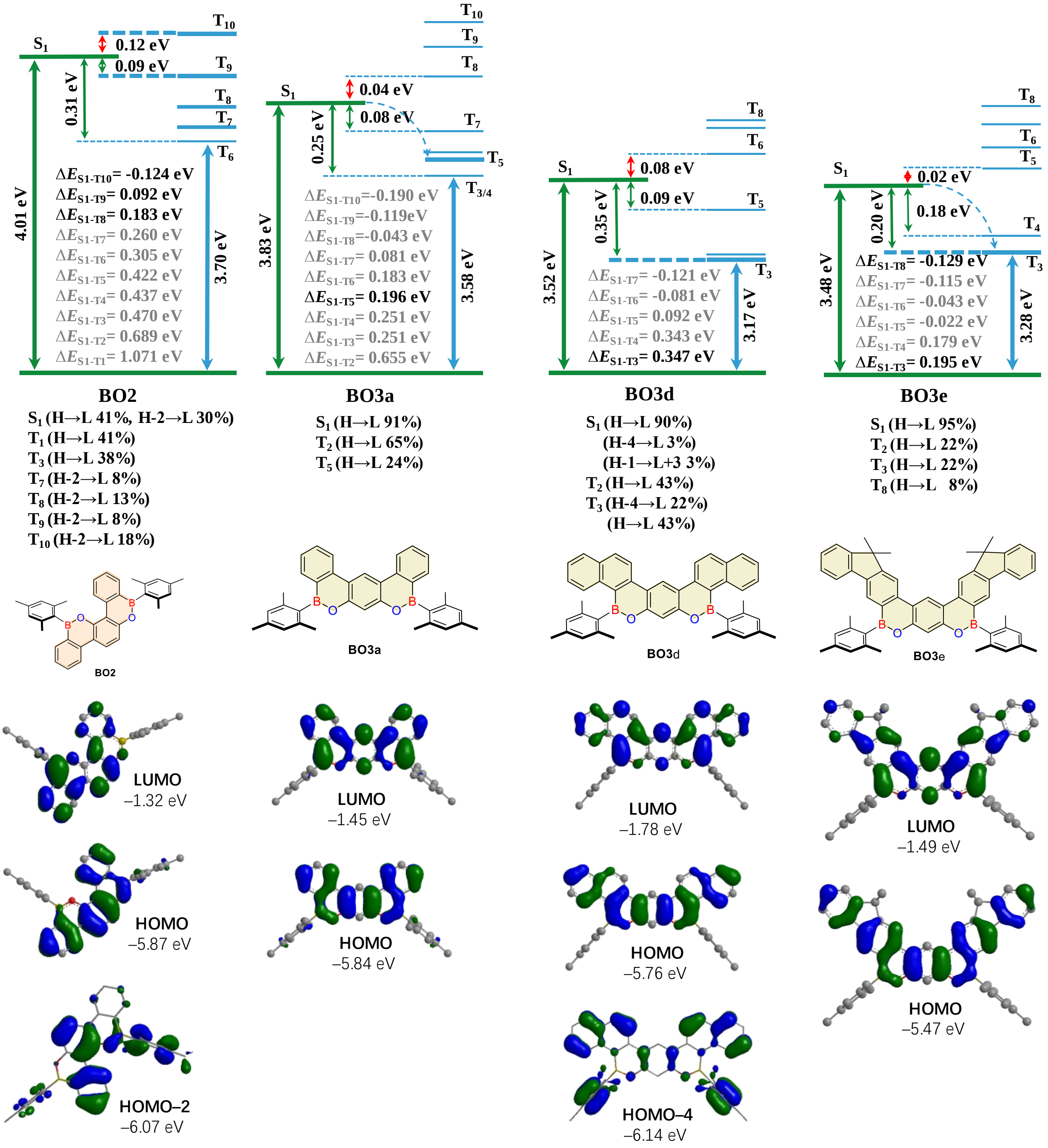

Supplementary Fig. 49. Theoretical calculation. TD-DFT calculated singlet and triplet energy levels, main transition configurations of **BO2**, **BO3a**, **B3d** and **BO3e** at B3LYP/6-31G(d) level based on optimized S_0 geometry. Selected frontier orbital distributions and energy levels are also illustrated.

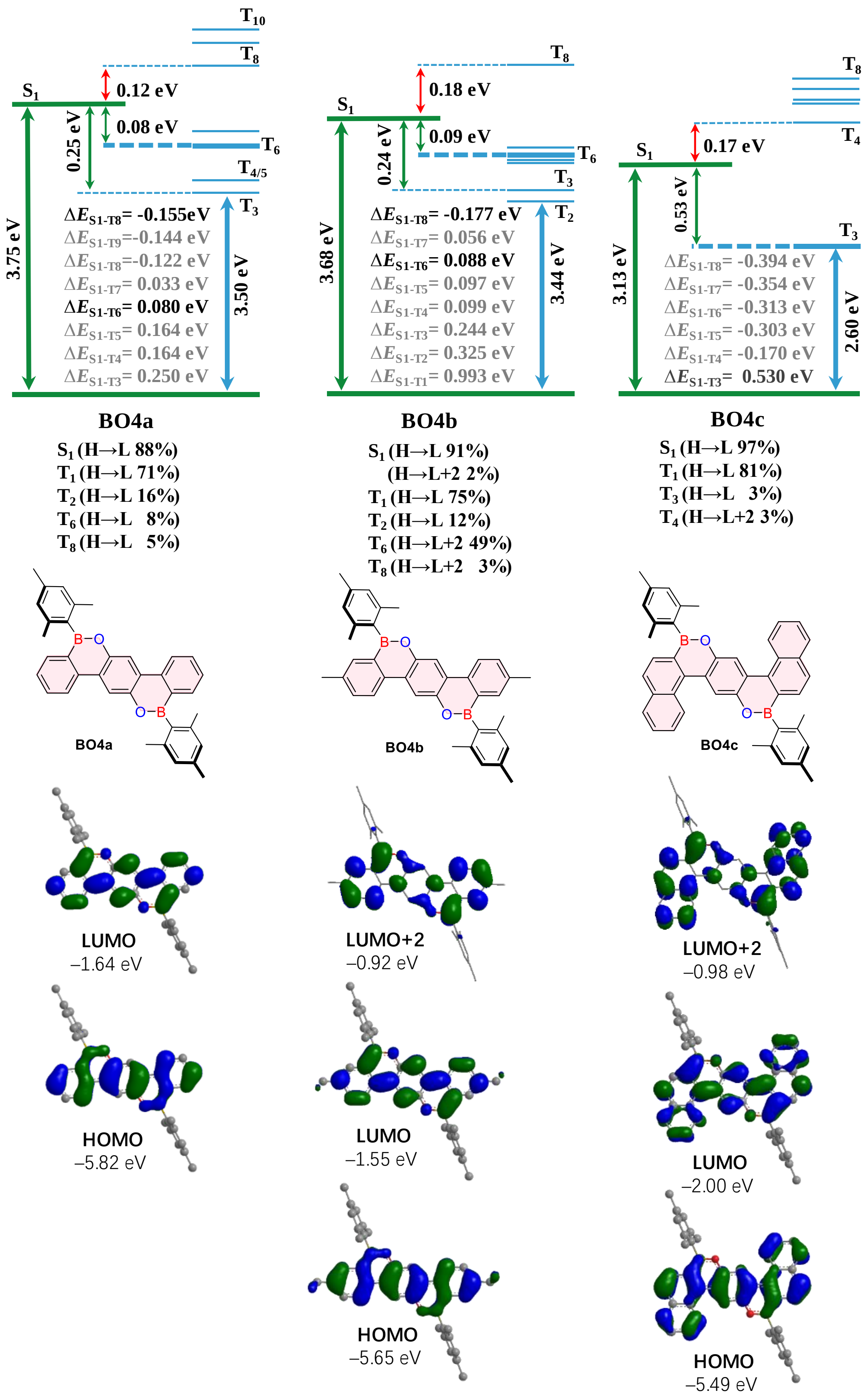

Supplementary Fig. 50. Theoretical calculation. TD-DFT calculated singlet and triplet energy levels, main transition configurations of **BO4a**, **BO4b** and **BO4c** at B3LYP/6-31G(d) level based on optimized S_0 geometry. Selected frontier orbital distributions and energy levels are also illustrated.

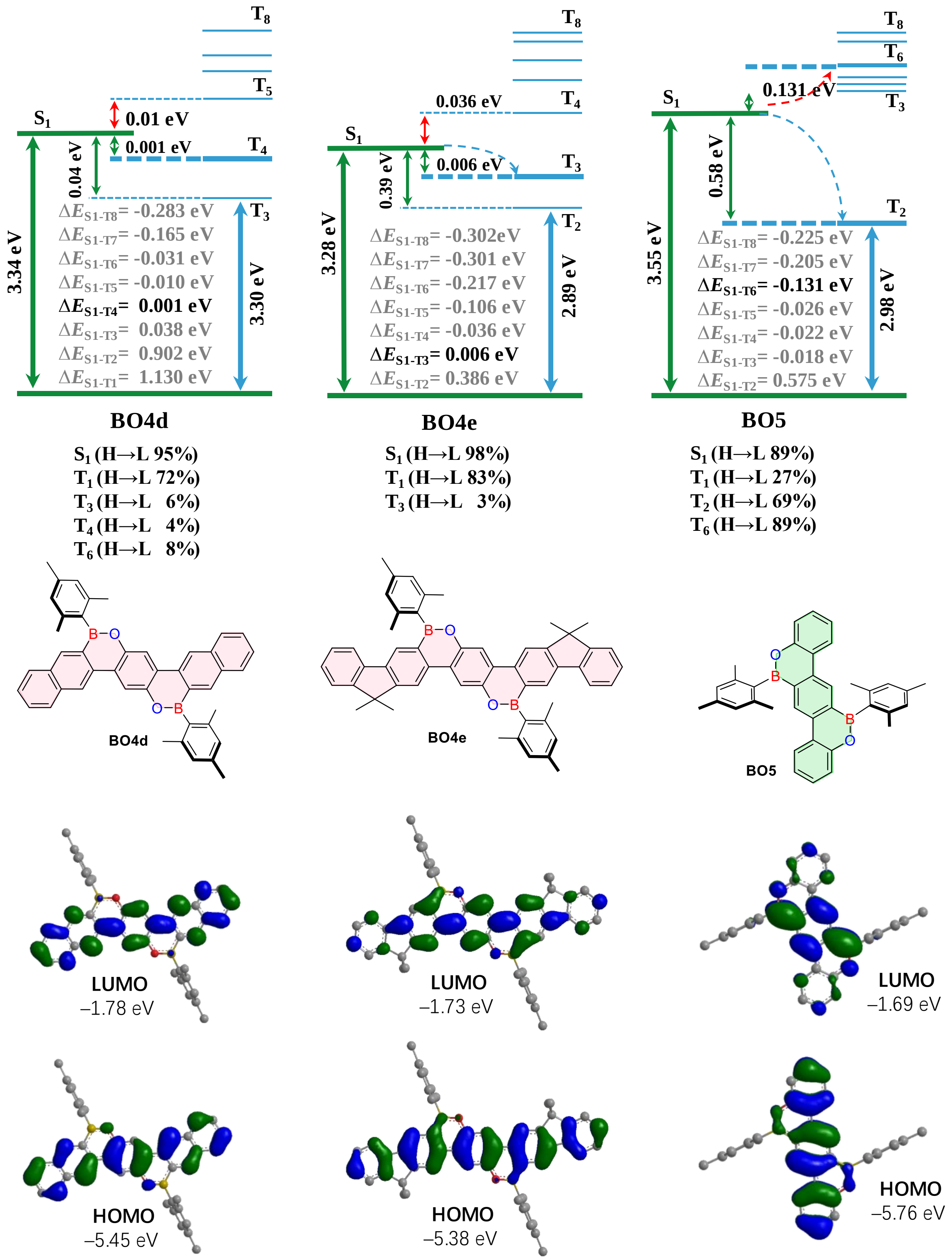

Supplementary Fig. 51. Theoretical calculation. TD-DFT calculated singlet and triplet energy levels, main transition configurations of **BO4d**, **BO4e** and **BO5** at B3LYP/6-31G(d) level based on optimized S_0 geometry. Selected frontier orbital distributions and energy levels are also illustrated.

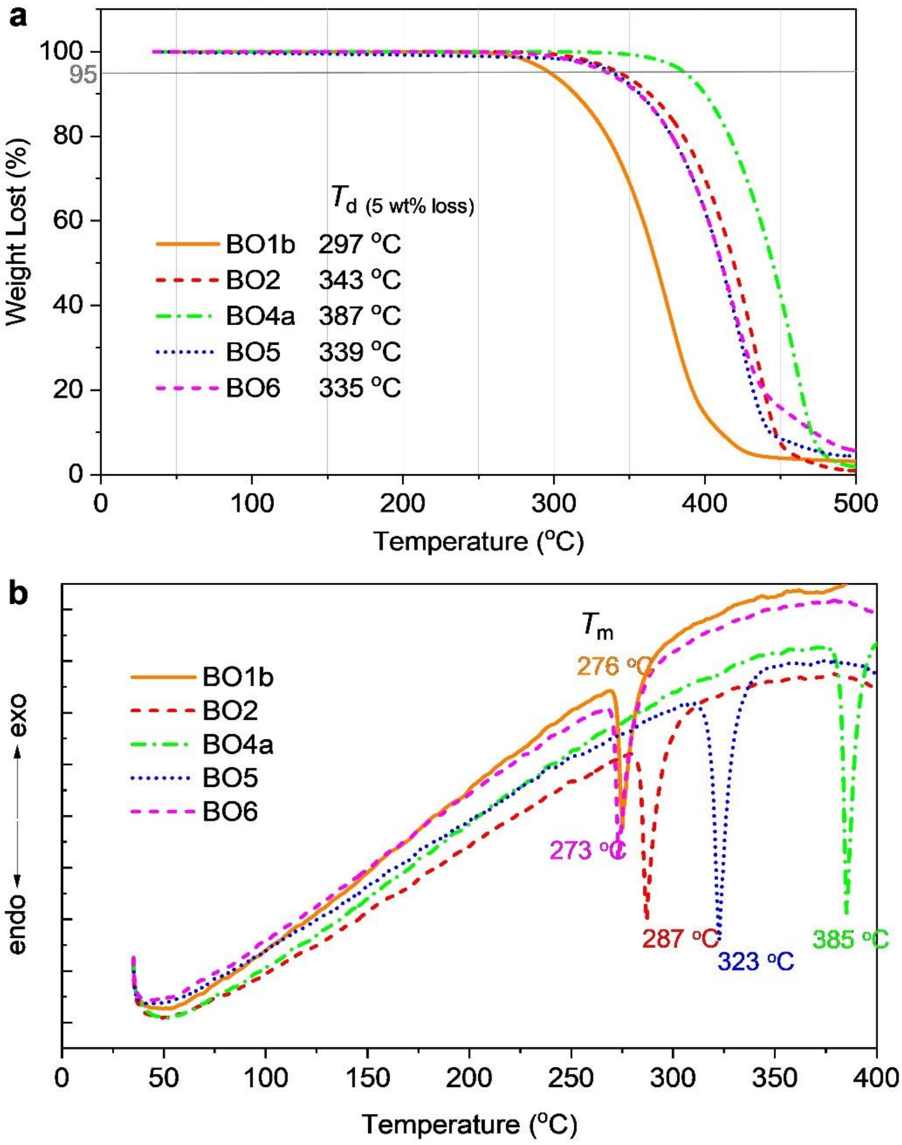

Supplementary Fig. 52. Thermal properties. a, TGA; **b**, DSC curves of **BO1b**, **BO2**, **BO4a**, **BO5** and **BO6**. The strongly endothermic peaks at 276 °C for **BO1b**, 287 °C for **BO2**, 385 °C for **BO4a**, 323 °C for **BO5**, and 273 °C for **BO6** could be attributed to their melting processes, in good agreement with their corresponding melting temperatures (T_m) of 276.2–277.9, 287.1–288.2, >350, 320.1–322.5 and 271.8–272.9 °C, respectively.

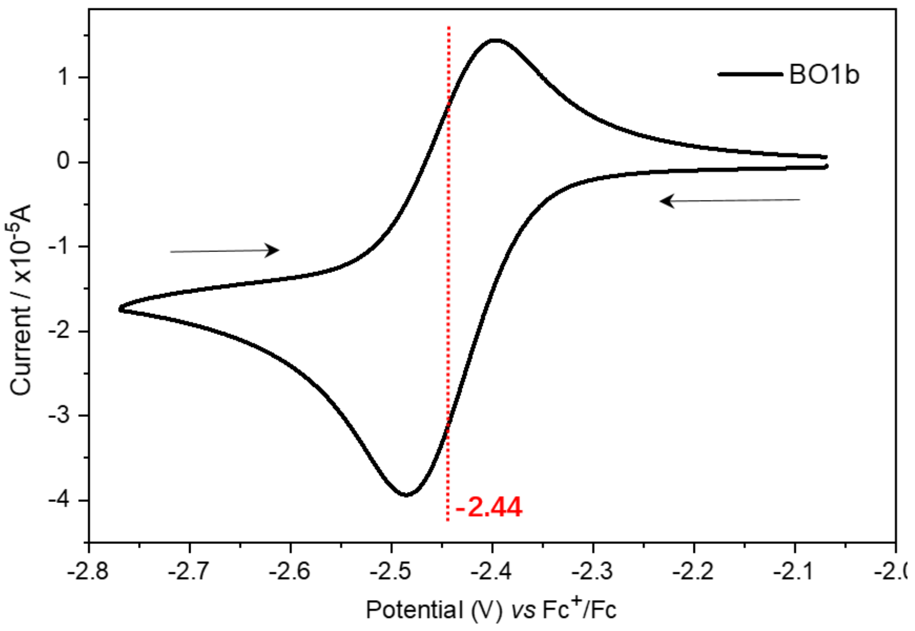

Supplementary Fig. 53. Electrochemical properties. Cyclic voltammogram (CV) of **BO1b** in anhydrous *N*,*N*-dimethylformamide (DMF).

Supplementary Table 6. Electrochemical properties and energy levels of selected dBO-PAHs

Compound	E_{red} (V)	LUMO[a] (eV)	E_g [b] (eV)	HOMO[c] (eV)
**BO1b**	–2.44	–2.36	3.50	–5.86
**BO1c**	–2.57	–2.23	3.54	–5.77
**BO1g**	–2.44	–2.36	3.50	–5.86
**BO2**	–2.66	–2.14	3.60	–5.74
**BO3a**	–2.61	–2.19	3.49	–5.68
**BO6**	–2.33	–2.47	3.48	–5.95

[a]LUMO = – (E_{red} + 4.8) eV. [b]Estimated from the onset wavelength of absorption spectrum measured in dichloromethane. [c]HOMO = LUMO – E_g.

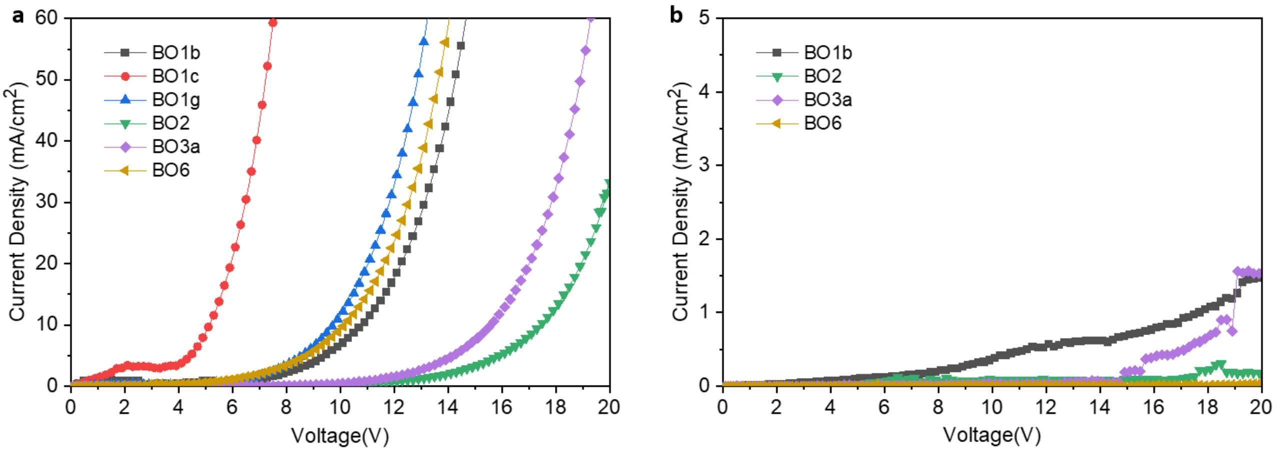

Supplementary Fig. 54. Single carrier devices. a Current density−voltage (*J*−*V*) characteristics of hole-only device with a structure of ITO/TAPC (10 nm)/dBO-PAH (60 nm)/TAPC (10 nm)/Al. **b** electron-only device with a structure of ITO/TmPyPB (10 nm)/ dBO-PAH (60 nm)/TmPyPB (10 nm)/LiF (1 nm)/Al.

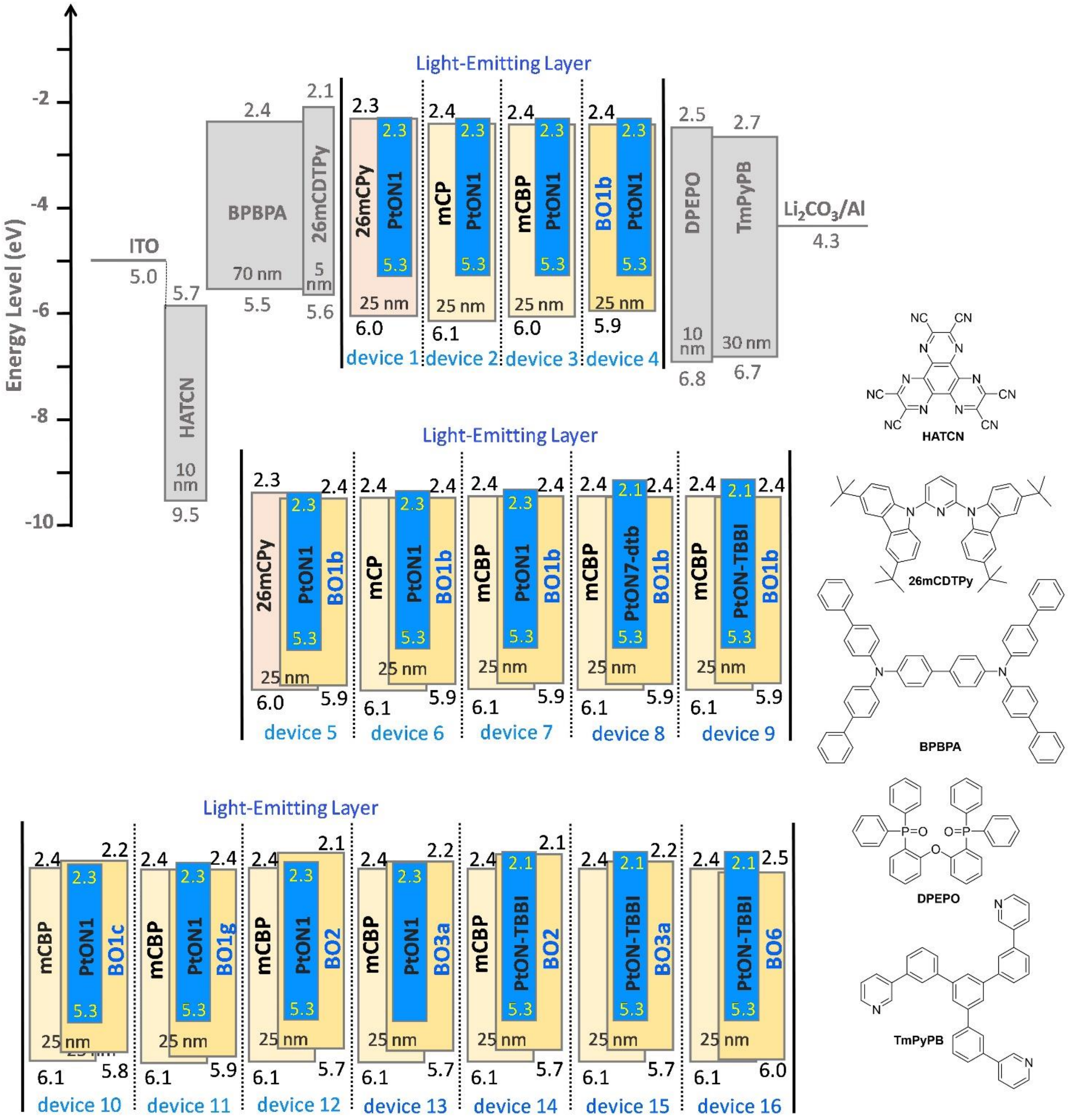

Supplementary Fig. 55. Device and material structures. Energy level diagram of deep-blue OLEDs and chemical structures of materials used.

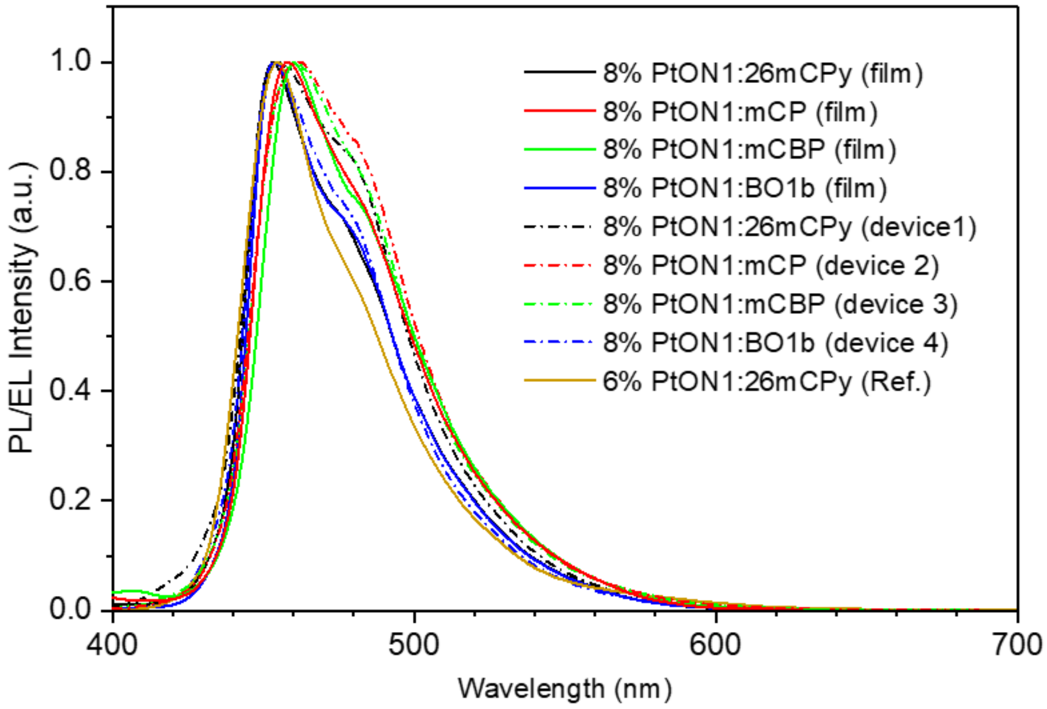

Supplementary Fig. 56. PL and EL spectral comparison. The PL of PtON1-doped thin films versus the EL of PtON1-doped devices. The Ref. is the device with a structure of ITO/HATCN/NPD/TAPC/6%PtON1:26mCPy/DPPS/LiF/Al [Hang, X. C., Fleetham, T., Turner, E., Brooks, J., Li, J. *Angew. Chem. Int. Ed.* **52**, 6753−6756 (2013).

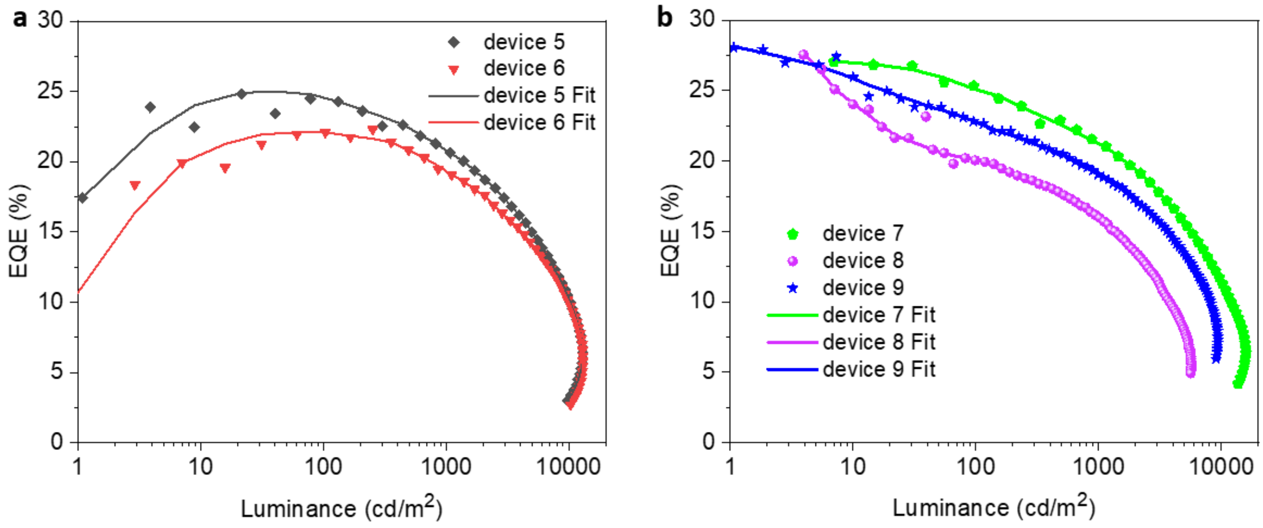

Supplementary Fig. 57. EL properties of deep-blue OLEDs. a, b Fitted EQE *vs.* luminance plots.

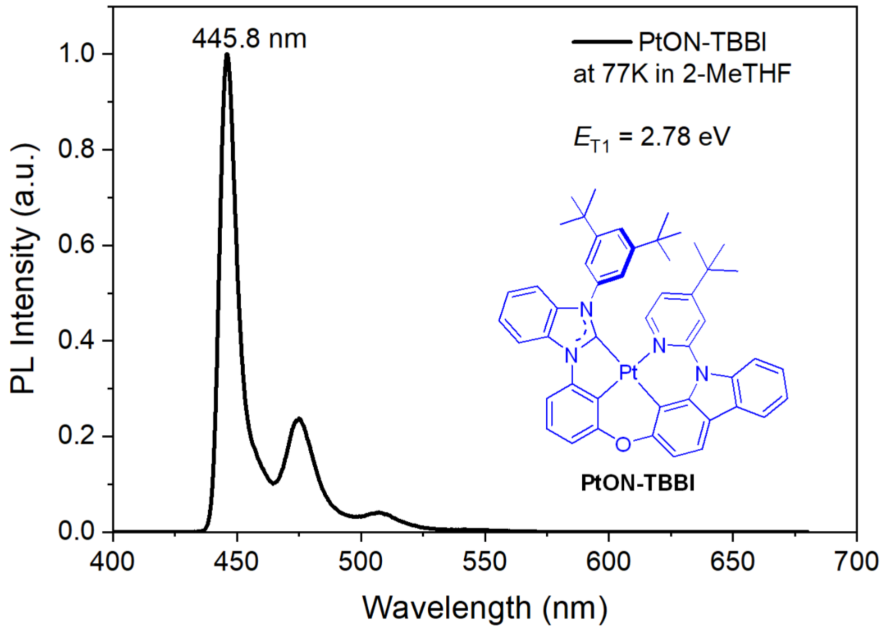

Supplementary Fig. 58. Low-temperature spectrum of PtON-TBBI

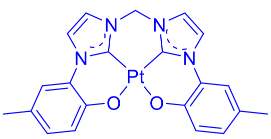

Pt(tetra-NHC) [1]
Chem. Commun.
2011, *47*, 9075.

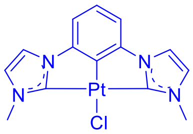

Pt-16 [2]
Org. Electron.
2012, *13*, 1430.

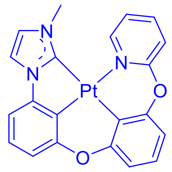

PtOO7 [3]
Angew. Chem. Int. Ed.
2013, *52*, 6753.

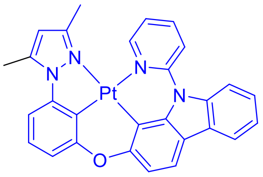

PtON1 [3]
Angew. Chem. Int. Ed.
2013, *52*, 6753.

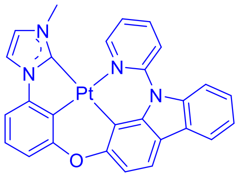

PtON7 [3]
Angew. Chem. Int. Ed.
2013, *52*, 6753.

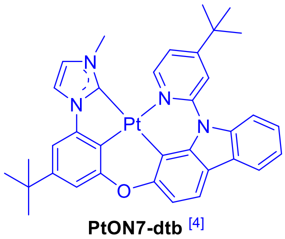

PtON7-dtb [4]
Adv. Mater.
2014, *26*, 7116.

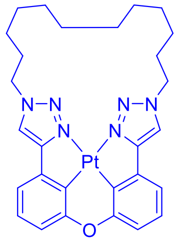

Pt6 [5]
Adv. Funct. Mater.
2017, *27*, 1604318.

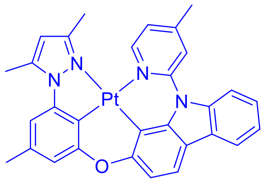

Pt(pzpyOczpy-4m) [6]
Mater. Chem. Front.
2019, *3*, 2448.

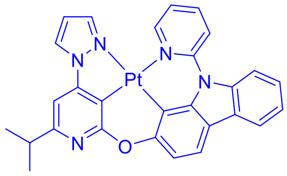

Pt(pzpyOczpy-^{i}Pr) [7]
Adv. Optical. Mater.
2020, *8*, 2000406.

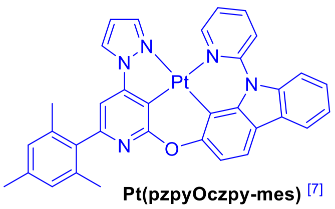

Pt(pzpyOczpy-mes) [7]
Adv. Optical. Mater.
2020, *8*, 2000406.

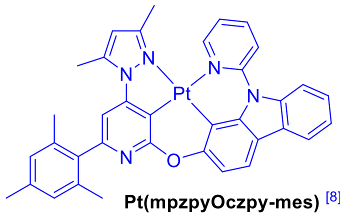

Pt(mpzpyOczpy-mes) [8]
ACS Appl. Mater. Interfaces
2021, *13*, 52833.

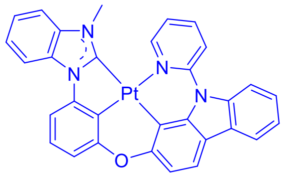

Pt-R [9]
Adv. Funct. Mater.
2021, *31*, 2100967.

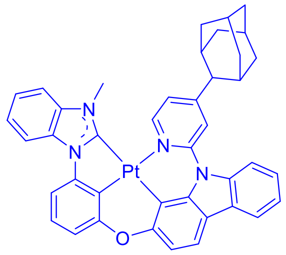

Pt-Ada [9]
Adv. Funct. Mater.
2021, *31*, 2100967.

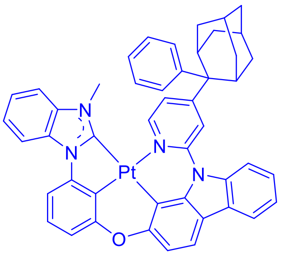

Pt-AdaPh [9]
Adv. Funct. Mater.
2021, *31*, 2100967.

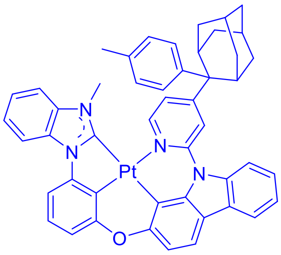

Pt-AdaTol [9]
Adv. Funct. Mater.
2021, *31*, 2100967.

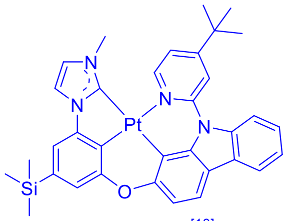

PtON7-TMS [10]
ACS Appl. Mater. Interfaces
2022, *14*, 34901.

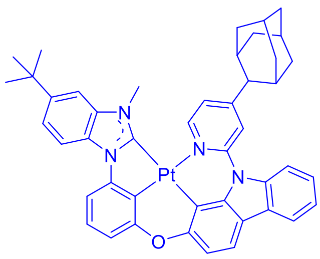

t-Pt-Ad [11]
Chem. Eng. J.
2022, *450*, 137836.

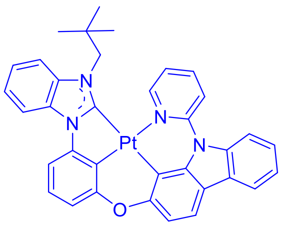

Pt-NPT [11]
Chem. Eng. J.
2022, *450*, 137836.

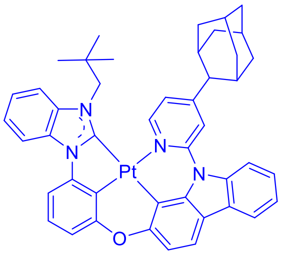

Pt-adNPT [11]
Chem. Eng. J.
2022, *450*, 137836.

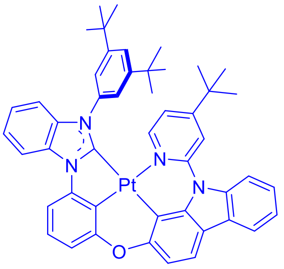

PtON-TBBI [12]
Nat. Photonics
2022, *16*, 212.

Supplementary Fig. 59. Deep-blue Pt(II) emitters. Chemical structures of dopants for Pt(II)-based deep-blue OLEDs discussed in this work.

Supplementary Table 7. Performance data for Pt(II)-based deep-blue OLEDs with CIE$_y$ < 0.20

emitter/host	CIE (x, y)	λ_{EL} (nm)	L_{max} (cd/m^2)	EQE(%)[a] max/100/1000	Reference/year
PtON1/**BO1b** (device 4)	(0.138, 0.134)	**453**	**8007**	**22.8/21.5/16.3**	**This work**
PtON1/**BO1b**/26mCPy (device 5)	(0.138, 0.130)	**454**	**12982**	**24.8/24.4/20.9**	**This work**
PtON1/**BO1b**/mCP (device 6)	(0.138, 0.154)	**460**	**13098**	**22.3/20.0/19.2**	**This work**
PtON1/**BO1b**/mCBP (device 7)	(0.138, 0.142)	**456**	**15722**	**27.1/25.2/21.8**	**This work**
PtON7-dtb/**BO1b**/mCBP (device 8)	(0.138, **0.088**)	**453**	**5670**	**27.6/20.0/16.0**	**This work**
PtON-TBBI/**BO1b**/mCBP (device 9)	(0.134, **0.104**)	**458**	**9377**	**28.0/22.8/19.0**	**This work**
PtON1/**BO1c**/mCBP (device 10)	(0.141, 0.168)	**456**	**27219**	**27.8/24.3/20.0**	**This work**
PtON1/**BO1g**/mCBP (device 11)	(0.140, 0.170)	**458**	**22638**	**25.1/21.9/19.2**	**This work**
PtON-TBBI/**BO2**/mCBP (device 14)	(0.135, **0.103**)	**458**	**14481**	**22.2/18.0/14.9**	**This work**
PtON-TBBI/**BO3a**/mCBP(device15)	(0.134, **0.107**)	**459**	**15765**	**19.6/17.0/14.8**	**This work**
PtON-TBBI/**BO6**/mCBP (device 16)	(0.133, **0.111**)	**459**	**10071**	**21.7/16.2/13.4**	**This work**
Pt(tetra-NHC)/DP4	(0.16, 0.16)	460	1200	---/---/---	[1]/2011
Pt-16/26mCPy	(0.16, 0.15)	450	~3000	15.7/10.1/<5	[2]/2012
PtOO7/26mCPy	(0.15, 0.10)	448	1000	<9/4.1/0.5	[3]/2013
PtON1/26mCPy	(0.15, 0.13)	454	1000	25.2/23.3/16.8	[3]/2013
PtON7/26mCPy	(0.15, 0.14)	456	1000	23.7/20.4/15.4	[3]/2013
PtON7-dtb/TAPC:PO15	(0.148, 0.079)	452	1555	24.8/22.7/11.0	[4]/2014
Pt6/BCPO	(0.14, 0.14)	452	10676	9.7/9.5/7.6	[5]/2017
Pt(ppzOczpy-4m)/CzSi:CzAcSF	(0.159, 0.166)	450	~4000	19.5/<10/12.1	[6]/2019
Pt(pzpyOczpy-^{i}Pr)/DPEPO	(0.14, 0.15)	454	1000	<20/17.2/11.2	[7]/2020
Pt(pzpyOczpy-mes)/DPEPO	(0.14, 0.15)	454	400	<20/15.0/6.6	[7]/2020
Pt(mpzpyOczpy-mes) /26mCPy:mCBP	(0.145, 0.195)	461	6518	---/12.1/---	[8]/2021
Pt-R/mCP	(0.138, 0.122)	~453	1645	22.0/---/---	[9]/2021
Pt-Ada/mCP	(0.145, 0.110)	~451	1078	21.2/---/---	[9]/2021
Pt-AdaPh/mCP	(0.137, 0.122)	~453	4528	23.5/---/---	[9]/2021
Pt-AdaTol/mCP	(0.140, 0.120)	~453	1593	22.0/---/---	[9]/2021
PtON7-TMS/mCBP	(0.142, 0.099)	452	2722	15.6/---/10.7	[10]/2022
t-Pt-Ad/mCP	(0.141, 0.092)	~453	3258	20.3/---/16.3	[11]/2022
Pt-NPT/mCP	(0.139, 0.118)	~451	3389	19.8/---/19.8	[11]/2022
Pt-adNPT/mCP	(0.143, 0.090)	~453	2512	11.2/---/15.7	[11]/2022
PtON-TBBI/SiCzCz:SiTrzCz2	(0.141, 0.197)	~455	~2000	25.4/24.6/23.4	[12]/2022

[a] EQE @ Max/100/1000 cd m^{-2}.

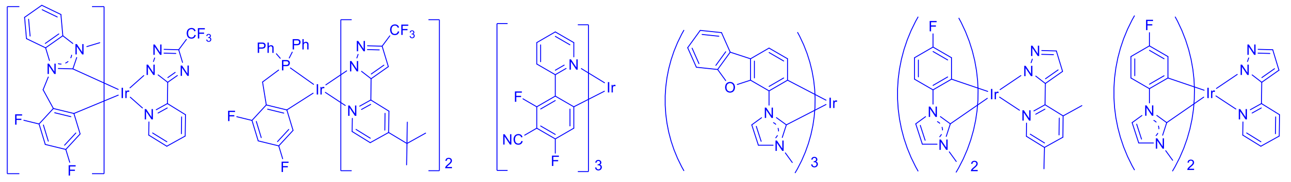

[(dfbmb)$_2$Ir(fptz)] *Angew. Chem. Int. Ed.* **2008**, *47*, 4512.

Ir(fppz)$_2$(dfbdp) *Adv. Mater.* **2009**, *21*, 2221.

FCNIr *Adv. Funct. Mater.* **2009**, *19*, 3644.

Ir(dbfmi) *Adv. Mater.* **2010**, *22*, 5003.

(fpmi)$_2$Ir(dmpypz) *Adv. Mater.* **2011**, *23*, 4933.

Ir(fpmi)$_2$(pypz) *Chem. Eur. J.* **2011**, *17*, 9180

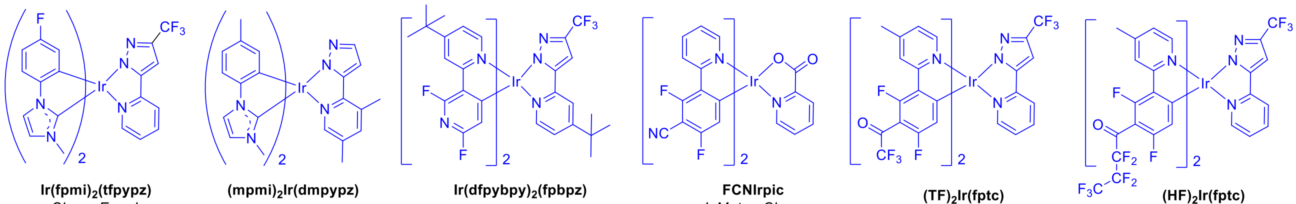

Ir(fpmi)$_2$(tfpypz) *Chem. Eur. J.* **2011**, *17*, 9180

(mpmi)$_2$Ir(dmpypz) *Adv. Mater.* **2011**, *23*, 4933.

Ir(dfpybpy)$_2$(fpbpz) *Chem. Mater.* **2012**, *24*, 3684.

FCNIrpic *J. Mater. Chem.* **2012**, *22*, 3099.

(TF)$_2$Ir(fptc) *J. Am. Chem. Soc.* **2013**, *135*, 14321.

(HF)$_2$Ir(fptc) *J. Am. Chem. Soc.* **2013**, *135*, 14321.

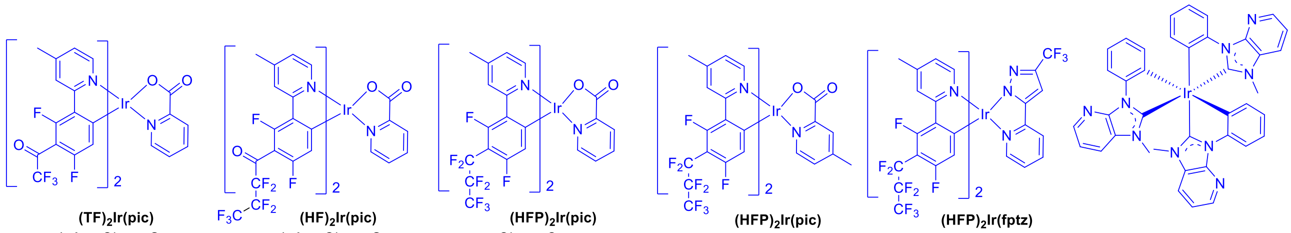

(TF)$_2$Ir(pic) *J. Am. Chem. Soc.* **2013**, *135*, 14321.

(HF)$_2$Ir(pic) *J. Am. Chem. Soc.* **2013**, *135*, 14321.

(HFP)$_2$Ir(pic) *Chem. Commun.* **2015**, *51*, 58.

(HFP)$_2$Ir(pic) *Chem. Commun.* **2015**, *51*, 58.

(HFP)$_2$Ir(fptz) *Chem. Commun.* **2015**, *51*, 58.

***mer*-Ir(pmp)$_3$** *Nat. Mater.* **2016**, *15*, 92.

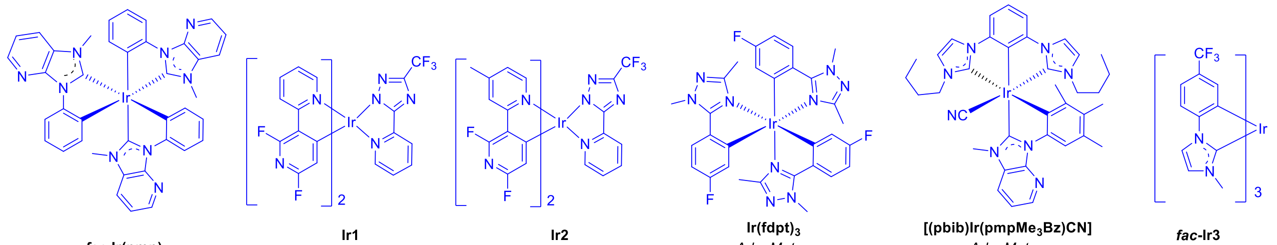

***fac*-Ir(pmp)$_3$** *Nat. Mater.* **2016**, *15*, 92.

Ir1 *J. Mater. Chem. C* **2016**, *4*, 10246.

Ir2 *J. Mater. Chem. C* **2016**, *4*, 10246.

Ir(fdpt)$_3$ *Adv. Mater.* **2018**, *30*, 1705005.

[(pbib)Ir(pmpMe$_3$Bz)CN] *Adv. Mater.* **2018**, *30*, 1705005.

***fac*-Ir3** *Adv. Mater.* **2018**, *30*, 1804231.

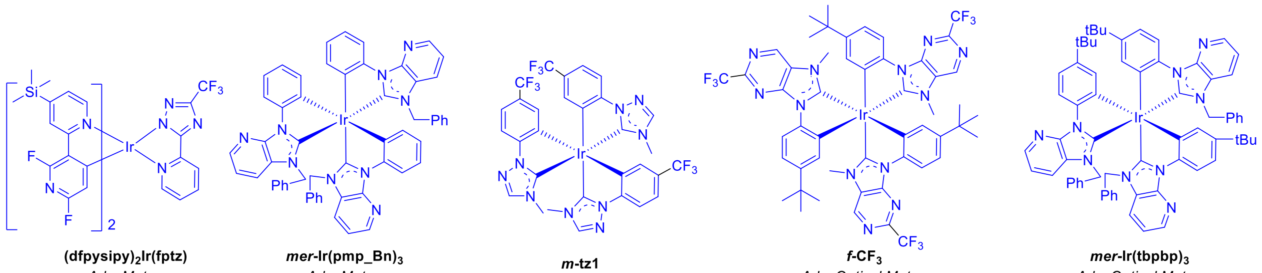

(dfpysipy)$_2$Ir(fptz) *Adv. Mater.* **2019**, *31*, 1808102.

***mer*-Ir(pmp_Bn)$_3$** *Adv. Mater.* **2020**, *32*, 2002120.

***m*-tz1** *ACS Appl. Mater. interfaces* **2021**, *13*, 59023.

***f*-CF$_3$** *Adv. Optical Mater.* **2022**, *10*, 2201633.

***mer*-Ir(tbpbp)$_3$** *Adv. Optical Mater.* **2022**, *10*, 2101686.

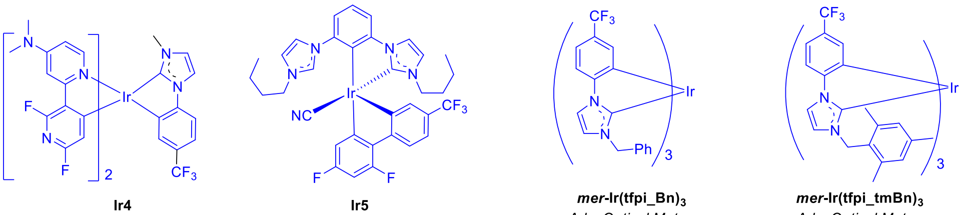

Ir4 *J. Mater. Chem. C* **2022**, *10*, 17965.

Ir5 *Adv. Optical Mater.* **2023**, *11*, 2201998.

***mer*-Ir(tfpi_Bn)$_3$** *Adv. Optical Mater.* **2023**, *11*, 2201495.

***mer*-Ir(tfpi_tmBn)$_3$** *Adv. Optical Mater.* **2023**, *11*, 2201495.

Supplementary Fig. 60. Deep-blue Ir(II) emitters. Chemical structures of dopants for Ir(III)-based deep-blue OLEDs discussed in this work.

Supplementary Table 8. Performance data for Ir(III)-based deep-blue OLEDs with CIE$_y$ < 0.20

emitter/host	CIE (x, y)	λ_{EL} (nm)	L_{max} (cd/m^2)	EQE(%) max /100/1000	Reference/year
PtON1/**BO1b** (device 4)	(0.138, 0.134)	**453**	**8007**	**22.8/21.5/16.3**	**This work**
PtON1/**BO1b**/26mCPy (device 5)	(0.138, 0.130)	**454**	**12982**	**24.8/24.4/20.9**	**This work**
PtON1/**BO1b**/mCP (device 6)	(0.138, 0.154)	**460**	**13098**	**22.3/20.0/19.2**	**This work**
PtON1/**BO1b**/mCBP (device 7)	(0.138, 0.142)	**456**	**15722**	**27.1/25.2/21.8**	**This work**
PtON7-dtb/**BO1b**/mCBP (device 8)	(0.138, **0.088**)	**453**	**5670**	**27.6/20.0/16.0**	**This work**
PtON-TBBI/**BO1b**/mCBP (device 9)	(0.134, **0.104**)	**458**	**9377**	**28.0/22.8/19.0**	**This work**
PtON1/**BO1c**/mCBP (device 10)	(0.141, 0.168)	**456**	**27219**	**27.8/24.3/20.0**	**This work**
PtON1/**BO1g**/mCBP (device 11)	(0.140, 0.170)	**458**	**22638**	**25.1/21.9/19.2**	**This work**
PtON-TBBI/**BO2**/mCBP (device 14)	(0.135, **0.103**)	**458**	**14481**	**22.2/18.0/14.9**	**This work**
PtON-TBBI/**BO3a**/mCBP(device15)	(0.134, **0.107**)	**459**	**15765**	**19.6/17.0/14.8**	**This work**
PtON-TBBI/**BO6**/mCBP (device 16)	(0.133, **0.111**)	**459**	**10071**	**21.7/16.2/13.4**	**This work**
Ir(dfbmb)$_2$(fptz)/UGH2	(0.16, 0.13)	434	<10000	6.0/~3/~1	[13]/2008
Ir(fppz)$_2$(dfbdp)/UGH2:CzSi	(0.15, 0.11)	---	1817	11.9/7.2/---	[14]/2009
FCNIr/PPO2	(0.14, 0.15)	452	<10000	18.4/13.5/~8	[15]/2009
FCNIr/PPO1	(0.14, 0.16)	452	<10000	17.1/12.2/<12	[15]/2009
Ir(dbfmi)/PPO2	(0.15, 0.19)	~450	<2500	18.6/13.3/6.2	[16]/2010
Ir(fpmi)$_2$(tfpypz)/UGH2:CzSi	(0.14, 0.10)	454	3446	7.6/~6/<6	[17]/2011
Ir(fpmi)$_2$(pypz)/UGH2	(0.14, 0.16)	466	5668	9.1/~9/<8	[17]/2011
Ir(fpmi)$_2$(pypz)/UGH2:CzSi	(0.14, 0.18)	470	8161	14.1/<12/<9	[17]/2011
(fpmi)$_2$Ir(dmpypz)/BCPO	(0.13, 0.16)	458	**20649**	17.1/16.5/15.1	**[18]/2011**
(mpmi)$_2$Ir(dmpypz)/BCPO	(0.13, 0.18)	464	**23727**	15.4/14.3/13.6	**[18]/2011**
FCNIrpic/mCPPO1	(0.14, 0.18)	~450	<5000	~25/25.1/24.0	[19]/2011
FCNIrpic/mCPPO1	(0.14, 0.17)	~460	<3500	22.0/~20/19.7	[20]/2011
FCNIrpic/DCPPO	(0.14, 0.17)	458	<8000	22.4/~20/18.4	[21]/2011
FCNIrpic/TSPC	(0.14, 0.18)	~450	<8000	22.4/18.4/---	[21]/2011
FCNIrpic/DFCzPO	(0.14, 0.18)	457	<5000	~21/~20/---	[22]/2011
FCNIrpic/mCPPO1	(0.14, 0.18)	458	<10000	24.7/~24/22.3	[23]/2012
Ir(dfpybpy)$_2$(fpbpz)/SimCP	(0.16, 0.19)	~420	<4000	4.9/---/---	[24]/2012
(TF)$_2$Ir(pic)/mCPPO1	(0.14, 0.16)	460	<3000	17.1/16.8/---	[25]/2013
(TF)$_2$Ir(fptc)/mCPPO1	(0.15, 0.12)	448	<2000	8.4/7.4/---	[25]/2013
(HF)$_2$Ir(pic)/mCPPO1	(0.14, 0.17)	460	<800	12.6/11.5/---	[25]/2013
(HF)$_2$Ir(fptc)/mCPPO1	(0.15, 0.13)	448	<400	8.4/7.10/---	[25]/2013
(dfpypy)$_2$Ir(pic)/CDBP	(0.16, 0.20)	~450	2373	<5/3.9/2.9	[26]/2013
(HFP)$_2$Ir(pic)/mCPPO1	(0.15, 0.16)	452	<1000	19.7/---/---	[27]/2015
(HFP)$_2$lr(mpic)/mCPPO1	(0.15, 0.17)	452	<1000	21.4/---/---	[27]/2015
(HFP)$_2$Ir(fptz)/mCPPO1	(0.15, 0.15)	447	<1000	14.2/---/---	[27]/2015
fac-Ir(pmp)$_3$/TSPO1	(0.16, 0.09)	---	>7800	<15/---/---	[28]/2016
mer-Ir(pmp)$_3$/TSPO1	(0.16, 0.15)	---	**~22000**	<15/---/---	**[28]/2016**
Ir1/*t*-BuCPO	(0.15, 0.13)	430	5080	11.2/10.8/8.3	[29]/2016
Ir2/*t*-BuCPO	(0.14, 0.11)	440	4710	13.0/12.6/10.1	[29]/2016
fac-Ir(dbfmi)$_3$/TSPO1	(0.14, 0.11)	---	<2000	18.5/---/---	[30]/2017

mer-Ir(dbfmi)$_3$/TSPO1	(0.14, 0.14)	---	<2000	18.2/---/---	[30]/2017
fac-Ir3/DPEPO	(0.15, 0.05)	430	<500	13.4/12.5/---	[31]/2018
Ir(fdpt)$_3$(10 wt%)/DPEPO	(0.15, 0.11)	458	2727	22.5/16.5/11.1	[32]/2018
Ir(fdpt)$_3$(12 wt%)/DPEPO	(0.15, 0.11)	458	3195	19.4/17.8/12.6	[32]/2018
(dfpysipy)$_2$Ir(fptz)/mCBP:TSPO1	(0.15, 0.18)	---	870	19.5/17.5/----	[33]/2019
(dfpysipy)$_2$Ir(pic)/mCBP:TSPO1	(0.14, 0.19)	449	3000	29.0/28.0/23.2	[33]/2019
(dfpysipy)$_2$Ir(mpic)/mCBP:TSPO1	(0.14, 0.19)	450	2800	31.9/30.4/24.7	[33]/2019
mer-Ir(pmp_Bn)$_3$/TSPO1	(0.15, 0.09)	442	6453	24.8/16.6/13.1	[34]/2020
Ir(cb)$_3$//mCBP:SiCzTrz	(0.14, 0.19)	~470	<10000	20.3/20.2/18.6	[35]/2020
m-tz1/DPEPO:30%	(0.15, 0.06)	432	<1000	10.0/---/---	[36]/2021
Ir2/PPF	(0.16, 0.20)	465	4350	16.4/11.3/7.2	[37]/2022
[(pbib)Ir(pmpMe$_3$Bz)CN]/ DPEPO	(0.17, 0.19)	469	1128	15.3/11.4/3.6	[38]/2022
f-CF$_3$/*t*-DABNA/mCBP^b	(0.13, 0.14)	464	8188	23.8/16.4/10.4	[39]/2022
mer-Ir(tbpbp)$_3$/ DPEPO	(0.16, 0.13)	449	2386	24.9/22.1/<10	[40]/2022
Ir/*v*-DABNA/mCBP^b	(0.13, 0.19)	---	11100	15.8/<15.0/10.5	[41]/2023
mer-Ir(tfpi_Bn)$_3$/TSPO1	(0.16, 0.08)	424	632	12.2/10.8/<5	[42]/2023
mer-Ir(tfpi_tmBn)$_3$/TSPO1	(0.16, 0.08)	424	940	14.9/11.7/<5	[42]/2023

^{a} EQE @ Max/100/1000 cd m^{-2}. ^{b} Hyperfluorescent OLED.

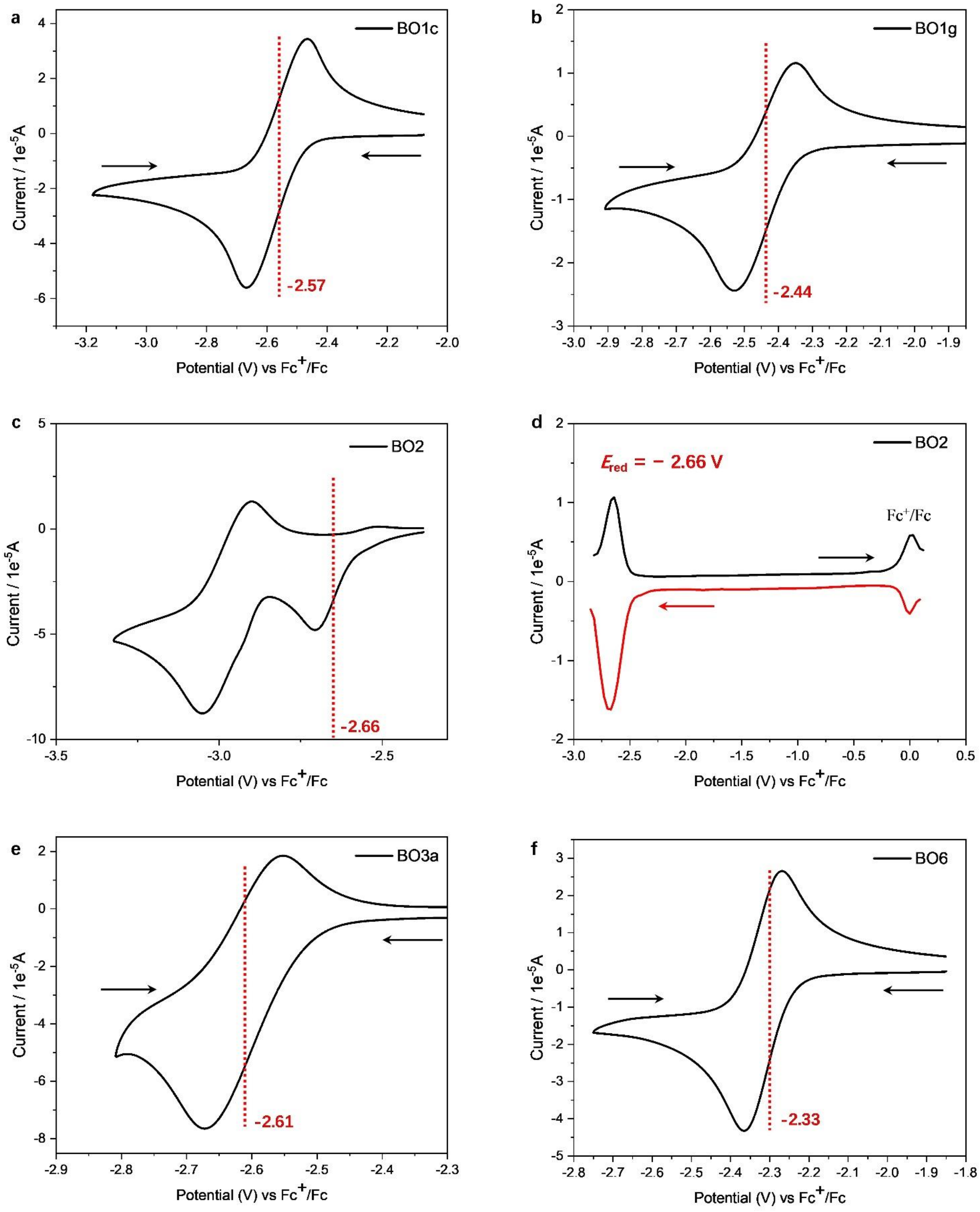

Supplementary Fig. 61. Electrochemical properties. Cyclic voltammogram (CV) of **a**, **BO1c**; **b**, **BO1g**; **c**, **BO2**; **e**, **BO3a**; **f**, **BO6**; **d**, differential pulse voltammetry (DPV) of **BO2** in anhydrous *N*,*N*-dimethylformamide (DMF).

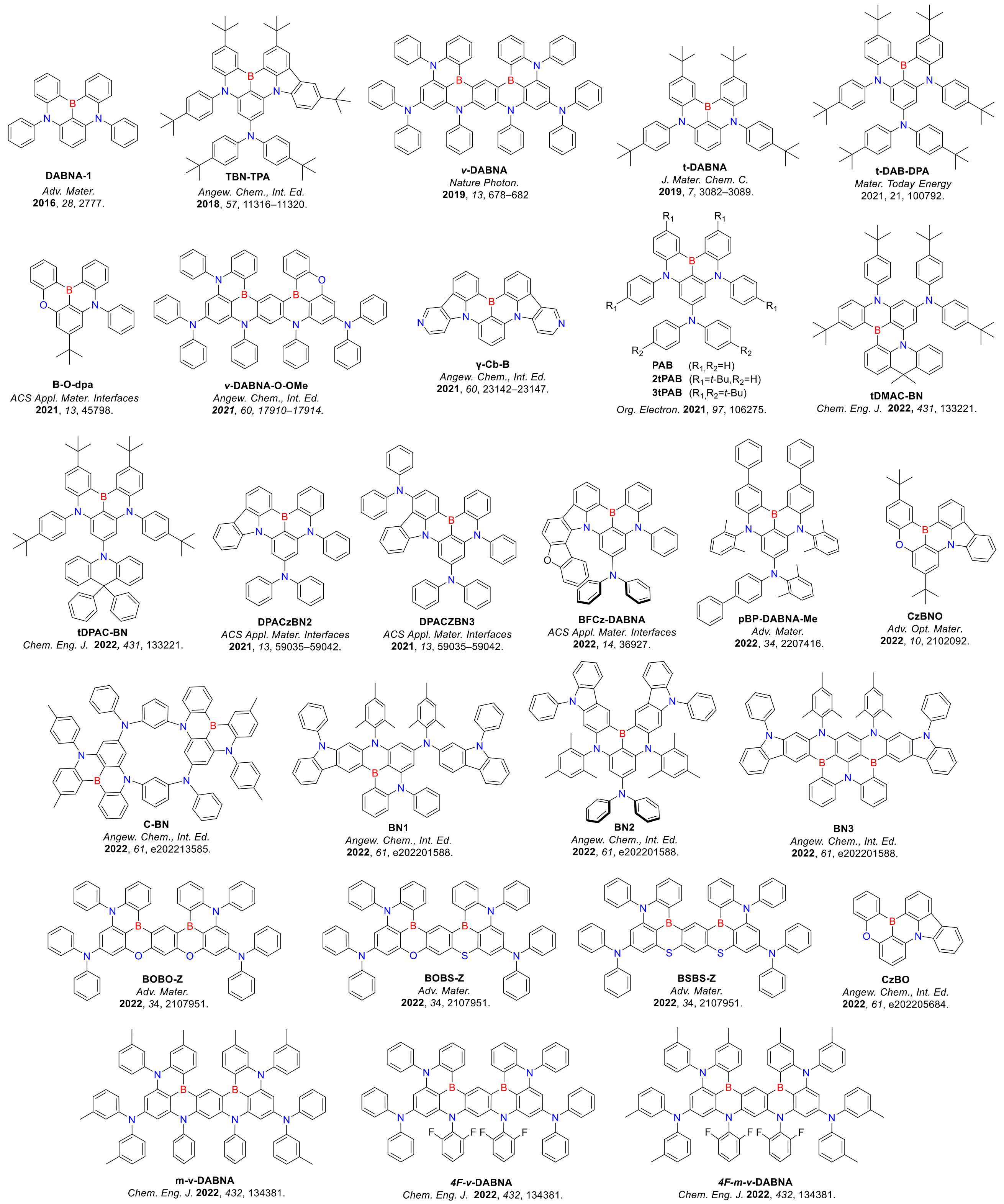

Supplementary Fig. 62. Deep-blue MR-BN-PAH emitters. Chemical structures of dopants for MR-BN-PAH-based deep-blue OLEDs discussed in this work.

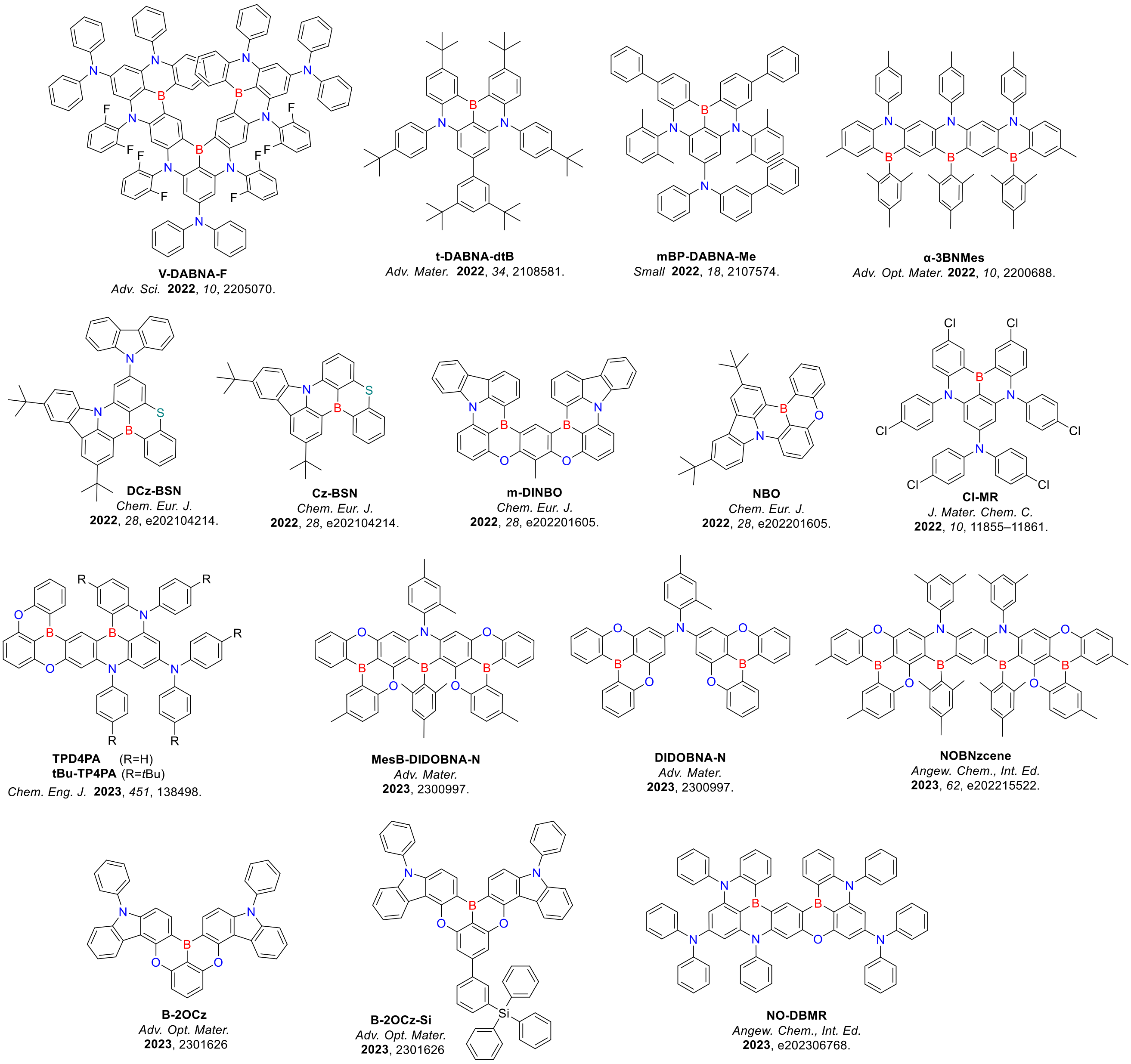

Supplementary Fig. 63. Deep-blue MR-BN-PAH emitters. Chemical structures of dopants for MR-BN-PAH-based deep-blue OLEDs discussed in this work.

Supplementary Table 9. Performance data for BN-based deep-blue OLEDs with CIE_y < 0.20

emitter/host	CIE (x, y)	λ_{EL} (nm)	L_{max} (cd/m^2)	EQE(%)[a] max/100/1000	Reference/year
PtON1/**BO1b** (device 4)	(0.138, 0.134)	**453**	**8007**	**22.8/21.5/16.3**	**This work**
PtON1/**BO1b**/26mCPy (device 5)	(0.138, 0.130)	**454**	**12982**	**24.8/24.4/20.9**	**This work**
PtON1/**BO1b**/mCP (device 6)	(0.138, 0.154)	**460**	**13098**	**22.3/20.0/19.2**	**This work**
PtON1/**BO1b**/mCBP (device 7)	(0.138, 0.142)	**456**	**15722**	**27.1/25.2/21.8**	**This work**
PtON7-dtb/**BO1b**/mCBP (device 8)	(0.138, **0.088**)	**453**	**5670**	**27.6/20.0/16.0**	**This work**
PtON-TBBI/**BO1b**/mCBP (device 9)	(0.134, **0.104**)	**458**	**9377**	**28.0/22.8/19.0**	**This work**
PtON1/**BO1c**/mCBP (device 10)	(0.141, 0.168)	**456**	**27219**	**27.8/24.3/20.0**	**This work**
PtON1/**BO1g**/mCBP (device 11)	(0.140, 0.170)	**458**	**22638**	**25.1/21.9/19.2**	**This work**
PtON-TBBI/**BO2**/mCBP (device 14)	(0.135, **0.103**)	**458**	**14481**	**22.2/18.0/14.9**	**This work**
PtON-TBBI/**BO3a**/mCBP(device15)	(0.134, **0.107**)	**459**	**15765**	**19.6/17.0/14.8**	**This work**
PtON-TBBI/**BO6**/mCBP (device 16)	(0.133, **0.111**)	**459**	**10071**	**21.7/16.2/13.4**	**This work**
DABNA-1/mCBP	(0.13, 0.09)	459	<1000	13.5/6.20/---	[43]/2016
TBN-TPA/2,6-DCzppy	(0.12, 0.19)	474	16593	32.1/27.4/13.9	[44]/2018
v-DABNA/DOBNA-OAr	(0.12, 0.11)	469	<3000	34.4/32.8/26.0	[45]/2019
t-DABNA/DMAC-DPS/DPEPO[b]	(0.13, 0.15)	466	<10000	31.4/27.2/19.8	[46]/2019
t-DAB-DPA/mCBP:mCBP-CN	(0.13, 0.08)	459	<10000	27.9/21.8/8.1	[47]/2021
B-O-dpa/DPEPO	(0.15, 0.05)	443	<1000	16.3/2.2/---	[48]/2021
v-DABNA-O-Me/DOBNA-Tol	(0.13, 0.10)	465	<13000	29.5/28.8/26.9	[49]/2021
γ-Cb-B/mCBP	(0.13, 0.13)	461	<3000	19.0/16.2/7.7	[50]/2021
3tPAB/mCP	(0.14, 0.08)	460	1100	19.3/<15/<5.0	[51]/2021
PAB/mCP	(0.14, 0.08)	456	782	14.7/~10/<5.0	[51]/2021
2tPAB/mCP	(0.14, 0.08)	456	1241	16.8/~10/<5.0	[51]/2021
tDMAC-BN/DMAC-DP/DPEPO[b]	(0.12, 0.19)	472	1536	22.3/19.0/10.4	[52]/2021
tDPAC-BN/DMAC-DPS/DPEPO[b]	(0.14, 0.09)	460	1126	21.6/15.3/5.4	[52]/2021
DPACzBN2/26DCzPPy	(0.13, 0.16)	469	15495	24.0/23.5/14.3	[53]/2021
DPACzBN3/26DCzPPy	(0.12, 0.18)	472	14629	27.7/19.3/6.7	[53]/2021
BFCz-DABNA/mCBP-CN	(0.13, 0.09)	463	<6000	28.0/13.4/5.1	[54]/2022
pBP-DABNA-Me/mCBP:DPEPO	(0.13, 0.09)	464	<1200	23.4/20.0/3.2	[55]/2022
C-BN/p4TzPhBN/mCBP[b]	(0.14, 0.07)	453	<10000	26.6/20.1/8.9	[56]/2022
BN1/3Cz2BN/DPFPO[b]	(0.14, 0.08)	457	8323	31.2/18.3/9.3	[57]/2022
BN2/3Cz2BN/DPFPO[b]	(0.13, 0.11)	467	14064	33.2/25.5/15.5	[57]/2022
BN3/3Cz2BN/DPFPO[b]	(0.14, 0.08)	458	18438	37.6/34.0/26.2	[57]/2022
CzBNO/26DCzPPy	(0.14, 0.08)	454	<8000	13.6/10.1/5.5	[58]/2022
CzBO/mCBP	(0.15, 0.05)	448	<2000	13.4/8.4/3.5	[59]/2022
BOBO-Z/mCBP	(0.15, 0.04)	445	<9000	13.6/9.8/3.3	[60]/2022
BOBS-Z/mCBP	(0.14, 0.06)	456	<9000	26.9/24.0/15.0	[60]/2022
BSBS-Z/mCBP	(0.13, 0.08)	463	<9000	26.8/24.0/15.9	[60]/2022
m-*v*-DABNA/ DBFPO	(0.12, 0.12)	471	~2000	36.2/---/---	[61]/2022
4F-*v*-DABNA/DBFPO	(0.13, 0.08)	464	~1000	35.8/26.4/10.2	[61]/2022
4F-m-*v*-DABNA/DBFPO	(0.13, 0.06)	461	~1000	33.7/25.8/---	[61]/2022
v-DABNA-F/DOBNA-Tol	(0.12, 0.10)	468	<10000	26.6/25.8/23.4	[62]/2022

t-DABNA-dtB/mCBP:mCBP-CN	(0.11, 0.16)	471	<10000	11.4/---/10.5	[63]/2022
mBP-DABNA-Me/mCP:DPEPO	(0.12, 0.14)	468	<1500	24.3/19.5/---	[64]/2022
α-3BNMes/DPEPO	(0.18, 0.08)	443	<900	---/1.70/---	[65]/2022
DCZ-BSN/mCBP	(0.11, 0.17)	473	<9000	22.0/---/---	[66]/2022
CzBSN/mCBP	(0.11, 0.16)	473	<9000	23.1/21.3/15.0	[66]/2022
m-DINBO/mCBP	(0.13, 0.10)	466	<1000	24.2/---/---	[67]/2022
NBO/mCBP	(0.14, 0.14)	459	<1000	16.8/---/---	[67]/2022
Cl-MR/DPEPO	(0.12, 0.19)	472	209	17.0/---/---	[68]/2022
TPD4PA/mCBP-CN	(0.14, 0.06)	455	<6000	30.7/30.6/17.8	[69]/2023
tBu-TPAD4PA/mCBP-CN	(0.14, 0.07)	460	<6000	32.5/30.9/20.5	[69]/2023
Mes-BDIDOBNA-N/CzSi:TSPO1	(0.17, 0.05)	402	<1000	16.2/3.5/---	[70]/2023
DIDOBNA-N/TSPO1	(0.15, 0.07)	429	<1000	15.2/3.8/---	[70]/2023
NOBNacene/TSPO1	(0.17, 0.06)	409	<100	8.5/---/---	[71]/2023
B-2OCz/DPEPO	(0.15, 0.06)	432	<1000	8.4/6.7/---	[72]/2023
B-2Ocz-Si/DPEPO	(0.16, 0.04)	423	<1000	15.2/8.1/---	[72]/2023
NO-DBMR/DBFPO	(0.12, 0.12)	469	<9000	33.7/---/---	[73]/2023

[a] EQE @ Max/100/1000 cd m^{-2}. [b] Hyperfluorescent OLED.

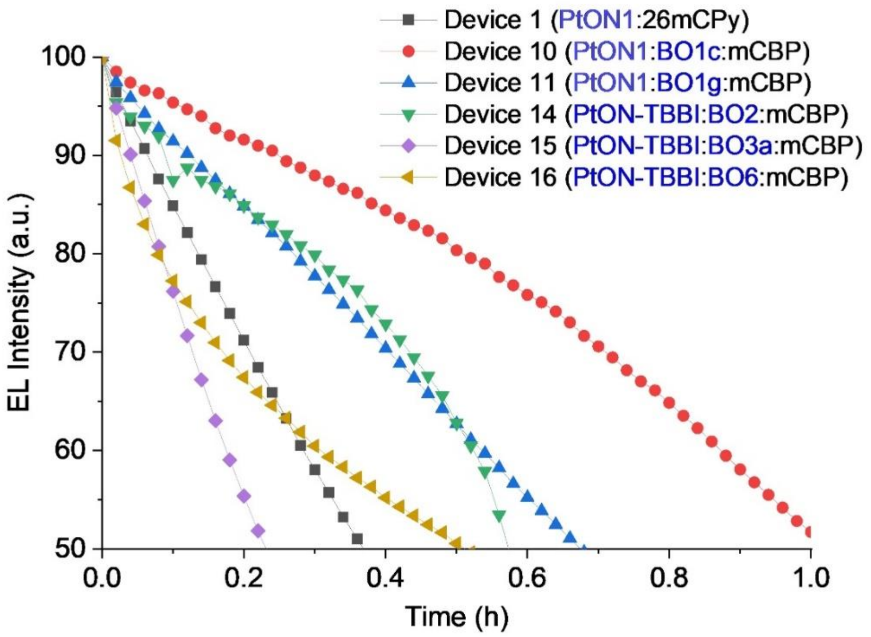

Supplementary Fig. 64. Operational lifetimes of the deep-blue OLEDs (L_0 = 500 cd/cm^2)

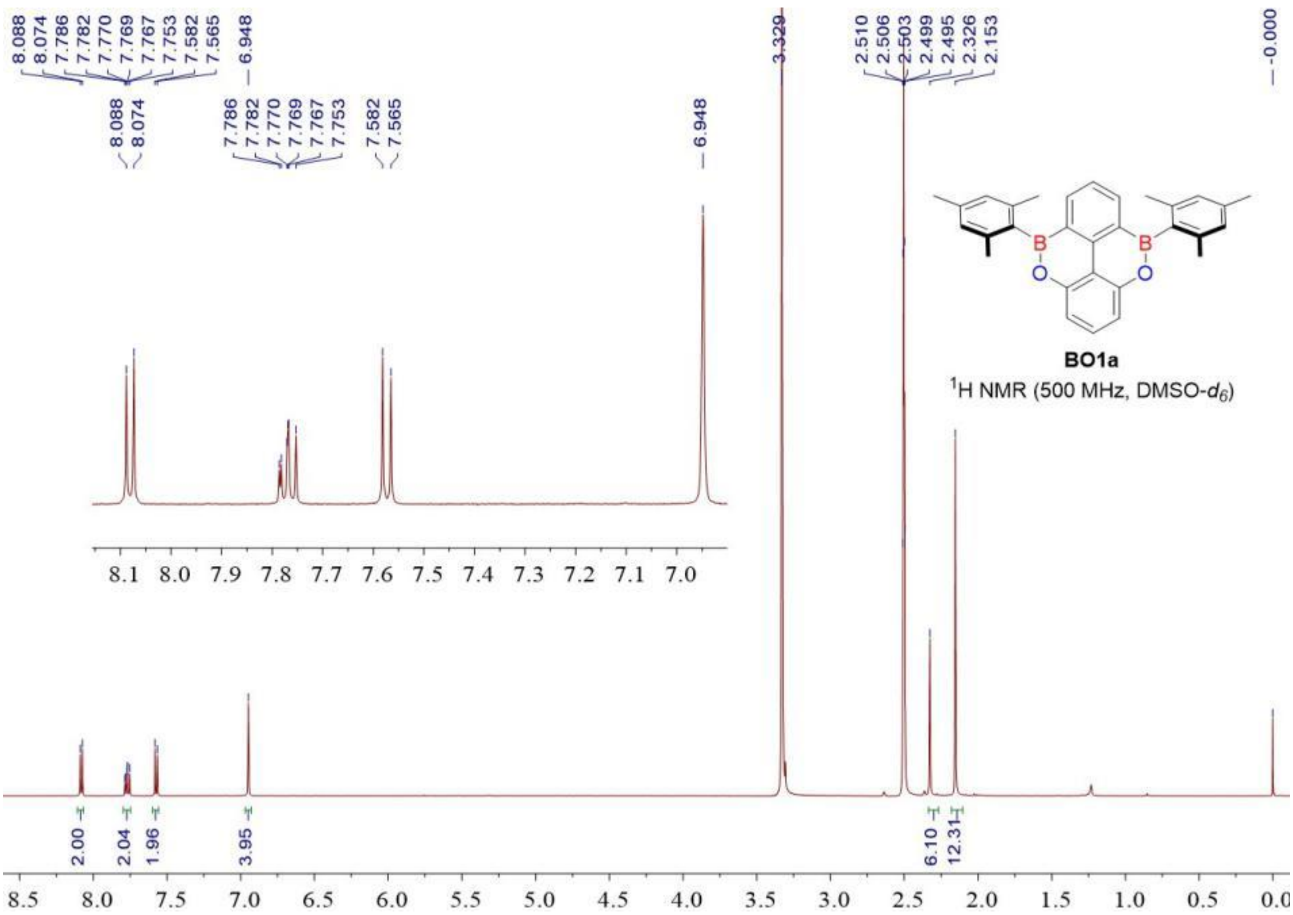

BO1a

^{1}H NMR (500 MHz, DMSO-d_6)

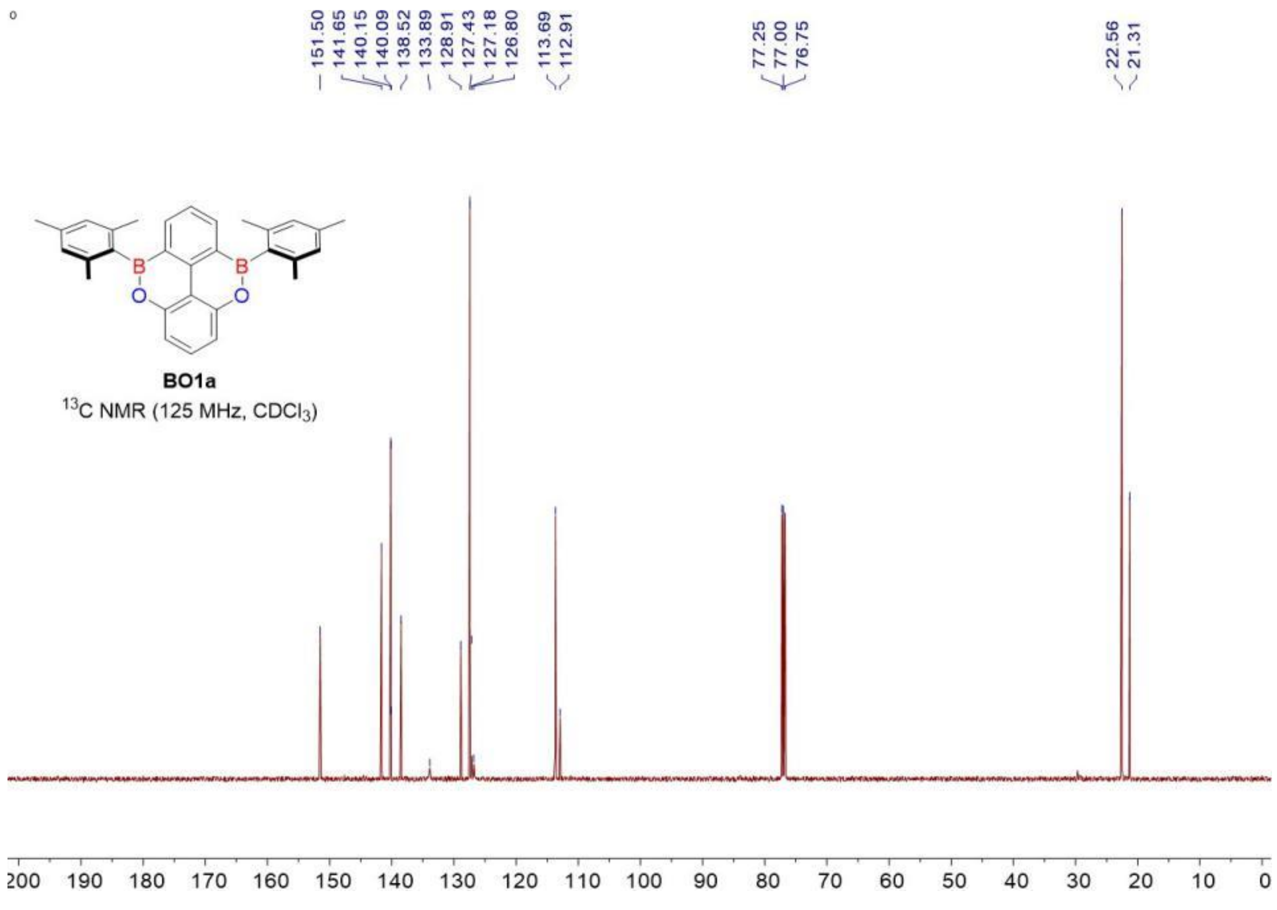

BO1a

^{13}C NMR (125 MHz, $CDCl_3$)

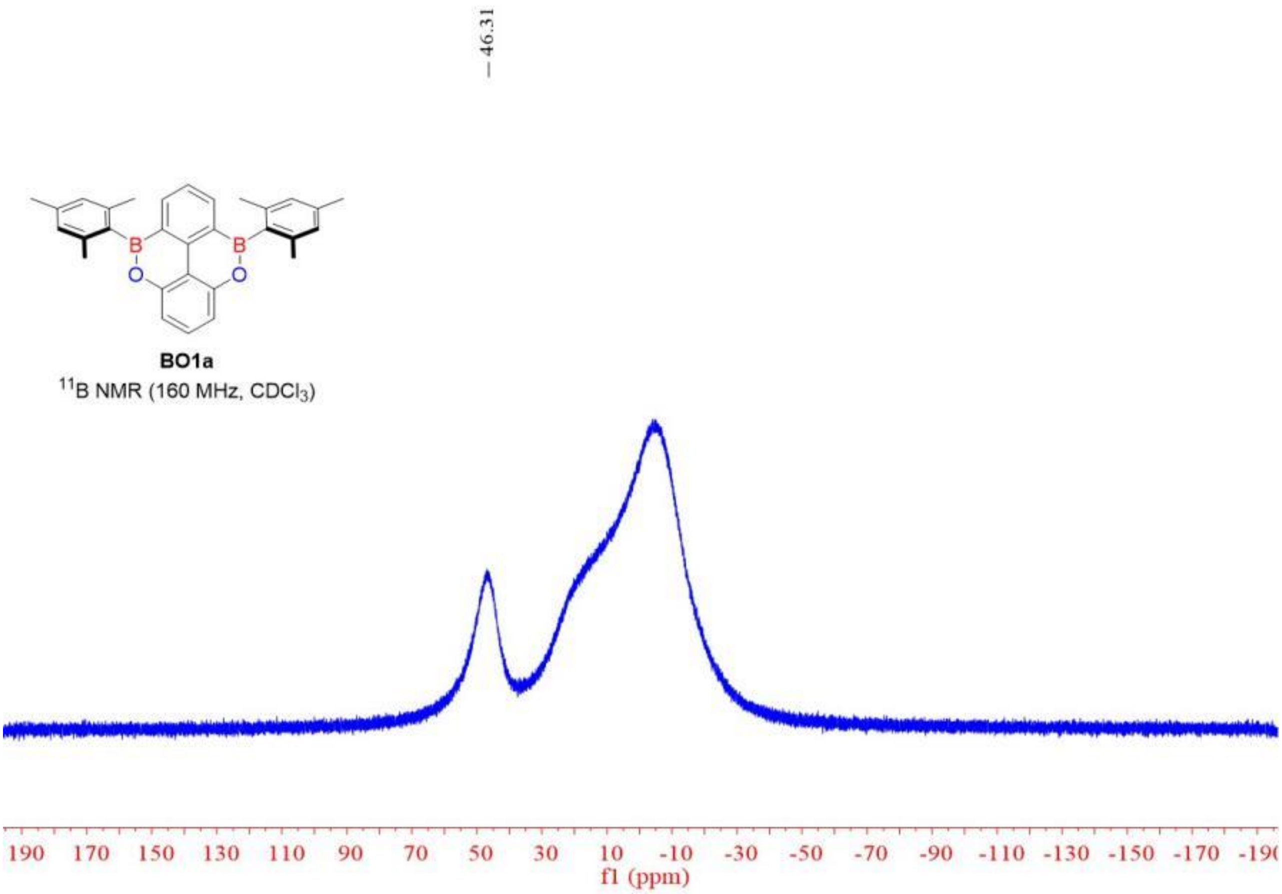

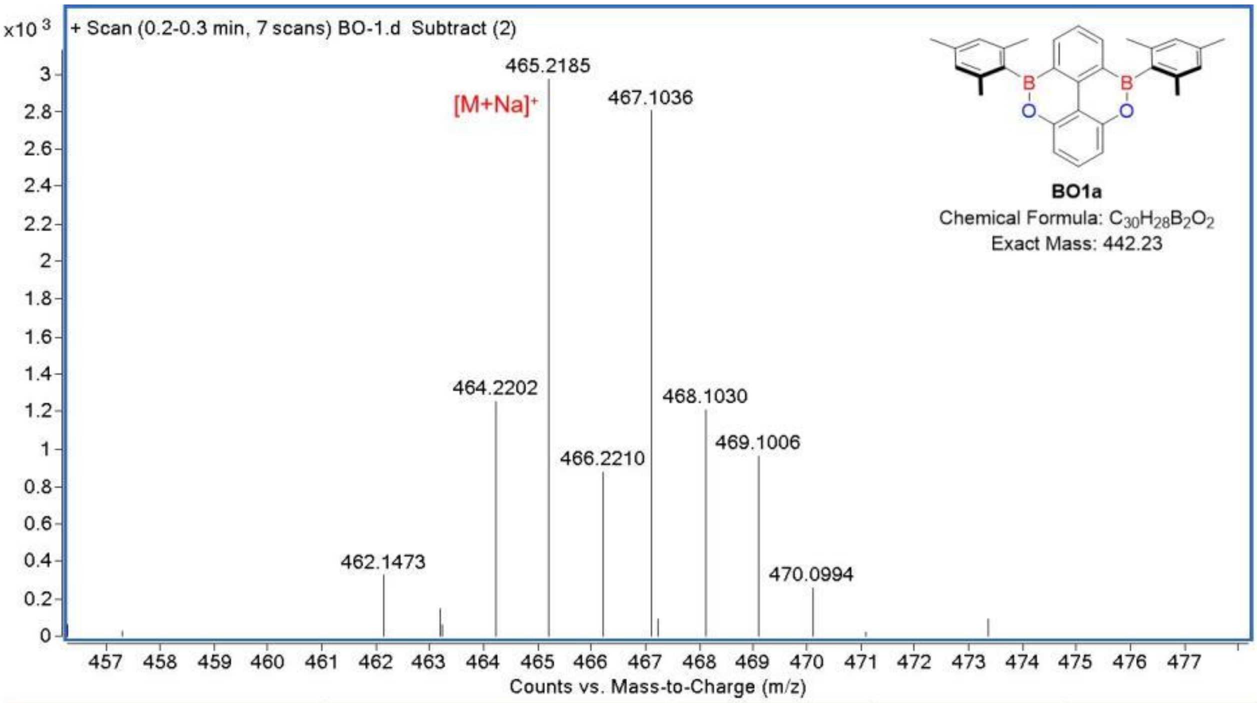

Formula (M)	Ion Formula	m/z	Calc m/z	Diff (ppm)
$C_{30}H_{28}[^{11}B]_2O_2$	$C_{30}H_{28}[^{11}B]_2NaO_2$	465.2185	465.2168	-3.93

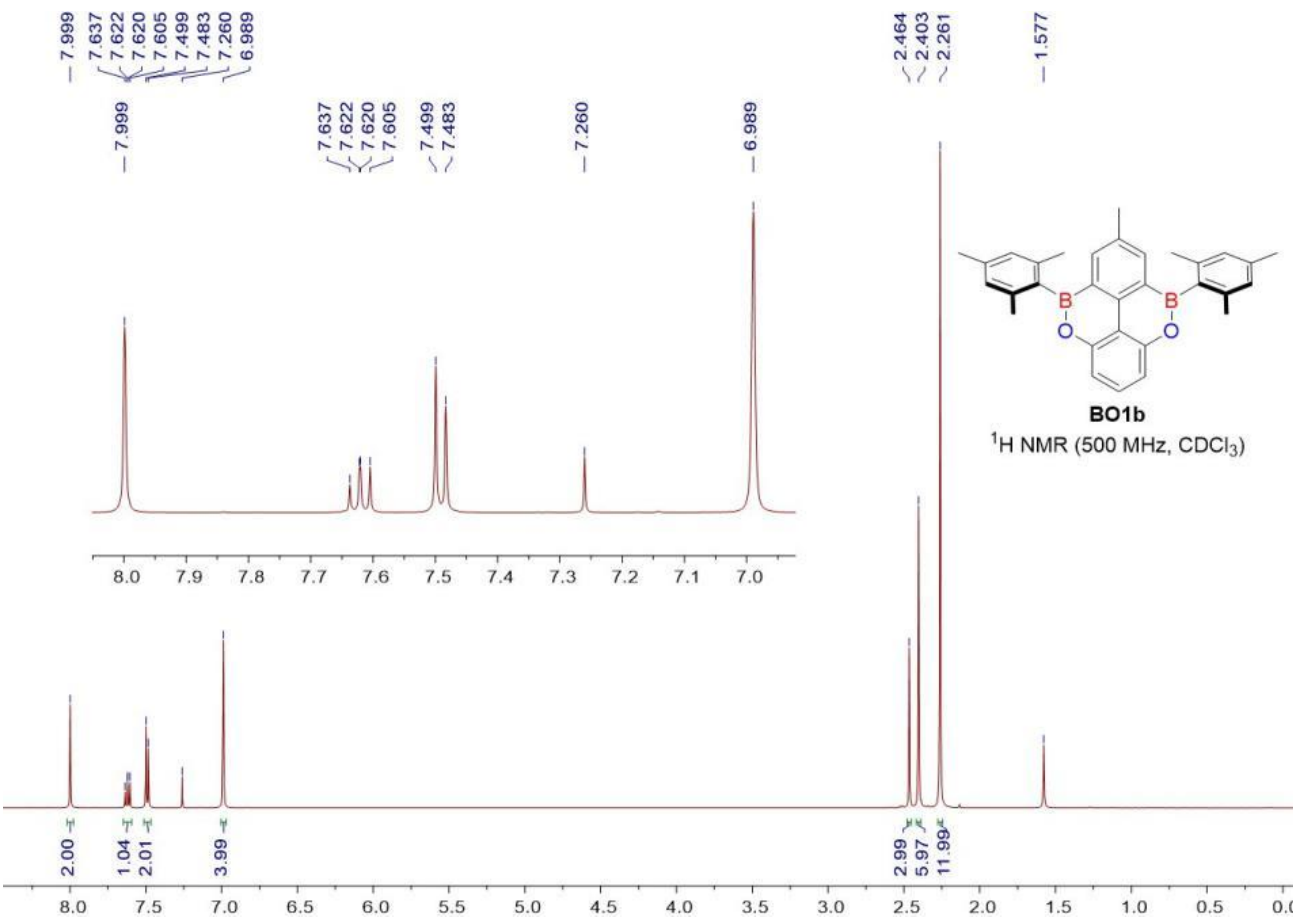
7.999
7.637
7.622
7.620
7.605
7.499
7.483
7.260
6.989
2.464
2.403
2.261
1.577
BO1b
^{1}H NMR (500 MHz, CDCl$_3$)
2.00
1.04
2.01
3.99
2.99
5.97
11.99

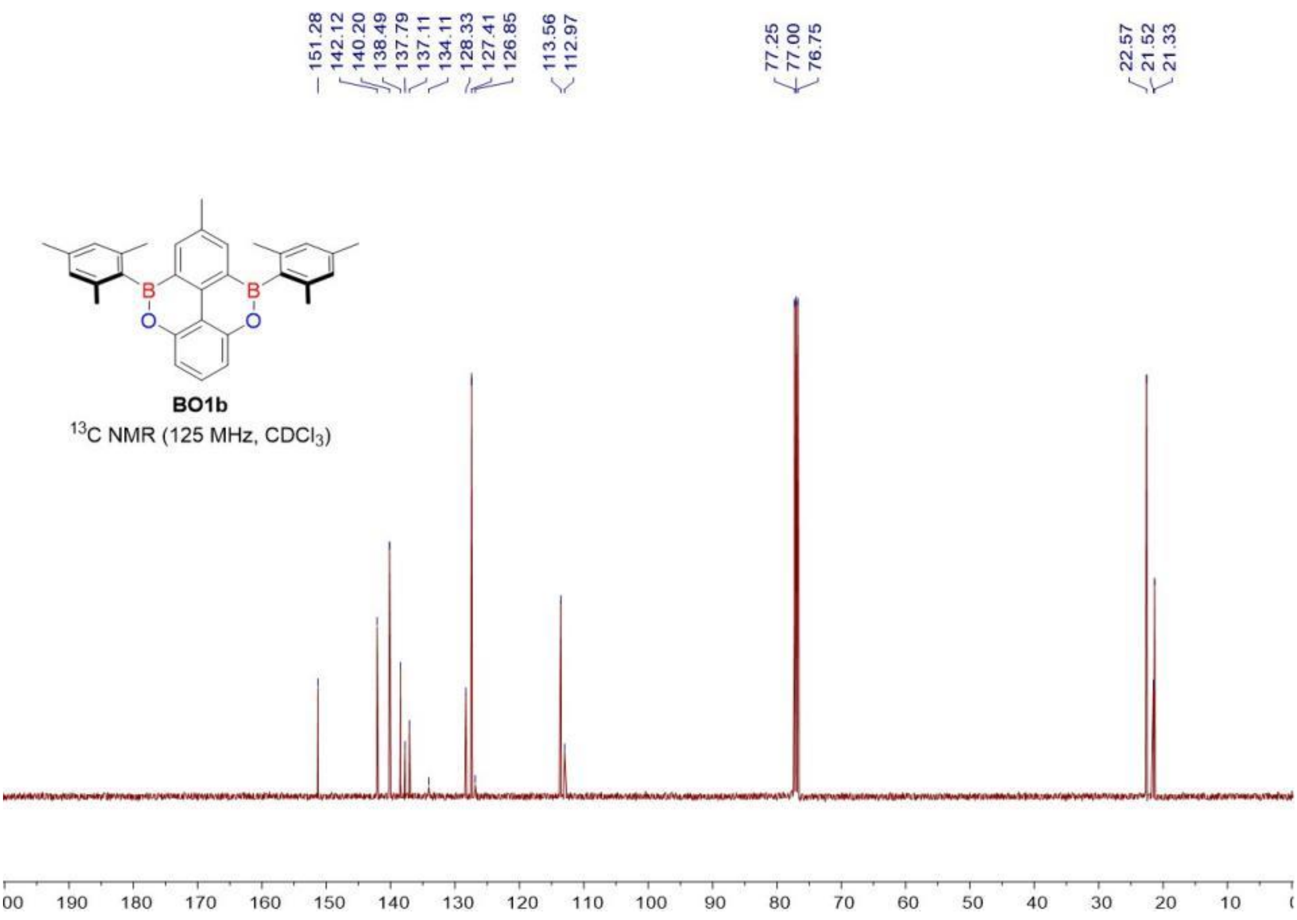
151.28
142.12
140.20
138.49
137.79
137.11
134.11
128.33
127.41
126.85
113.56
112.97
77.25
77.00
76.75
22.57
21.52
21.33
BO1b
^{13}C NMR (125 MHz, CDCl$_3$)

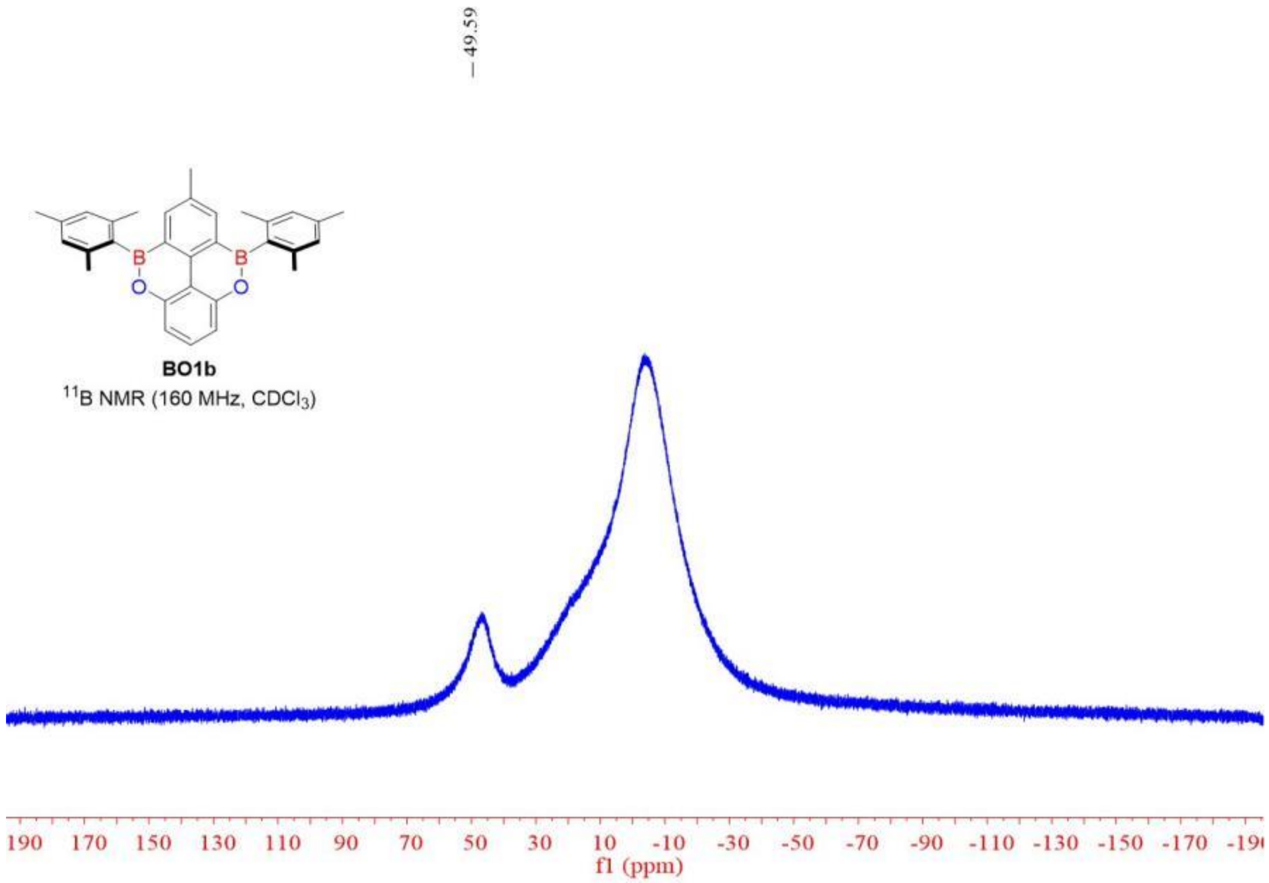

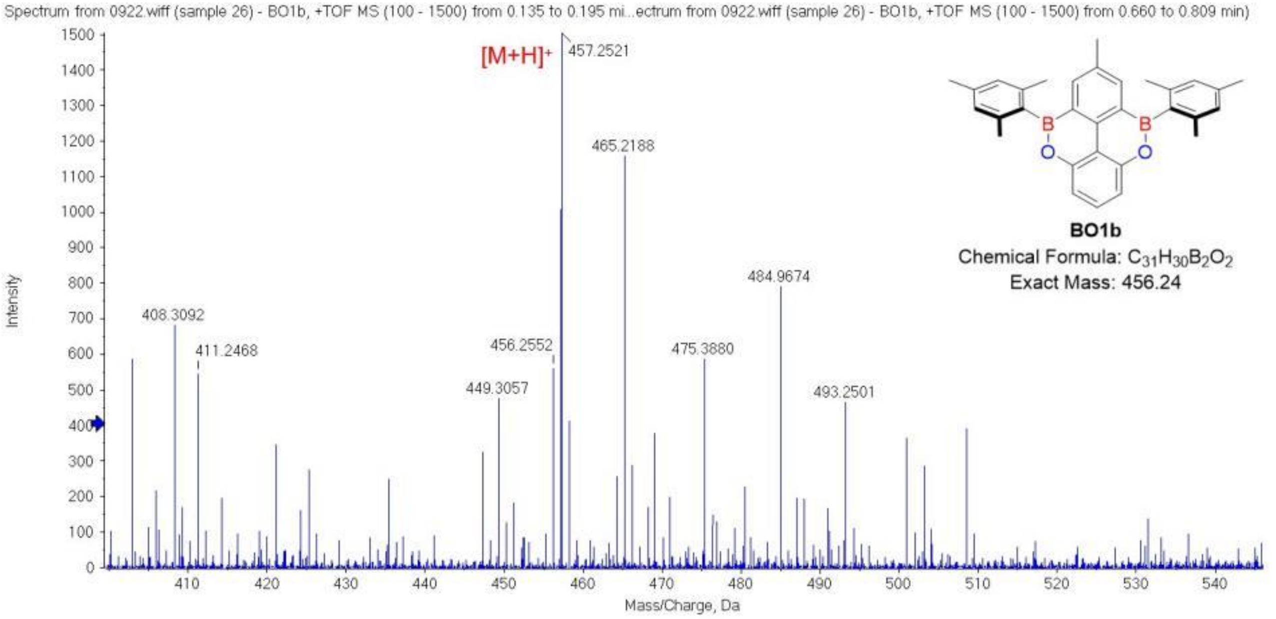

Formula (M)	Ion Formula	m/z	Calc m/z	Diff (ppm)
$C_{31}H_{30}[^{11}B]_2O_2$	$C_{31}H_{31}[^{11}B]_2O_2$	457.2521	457.2505	-3.70

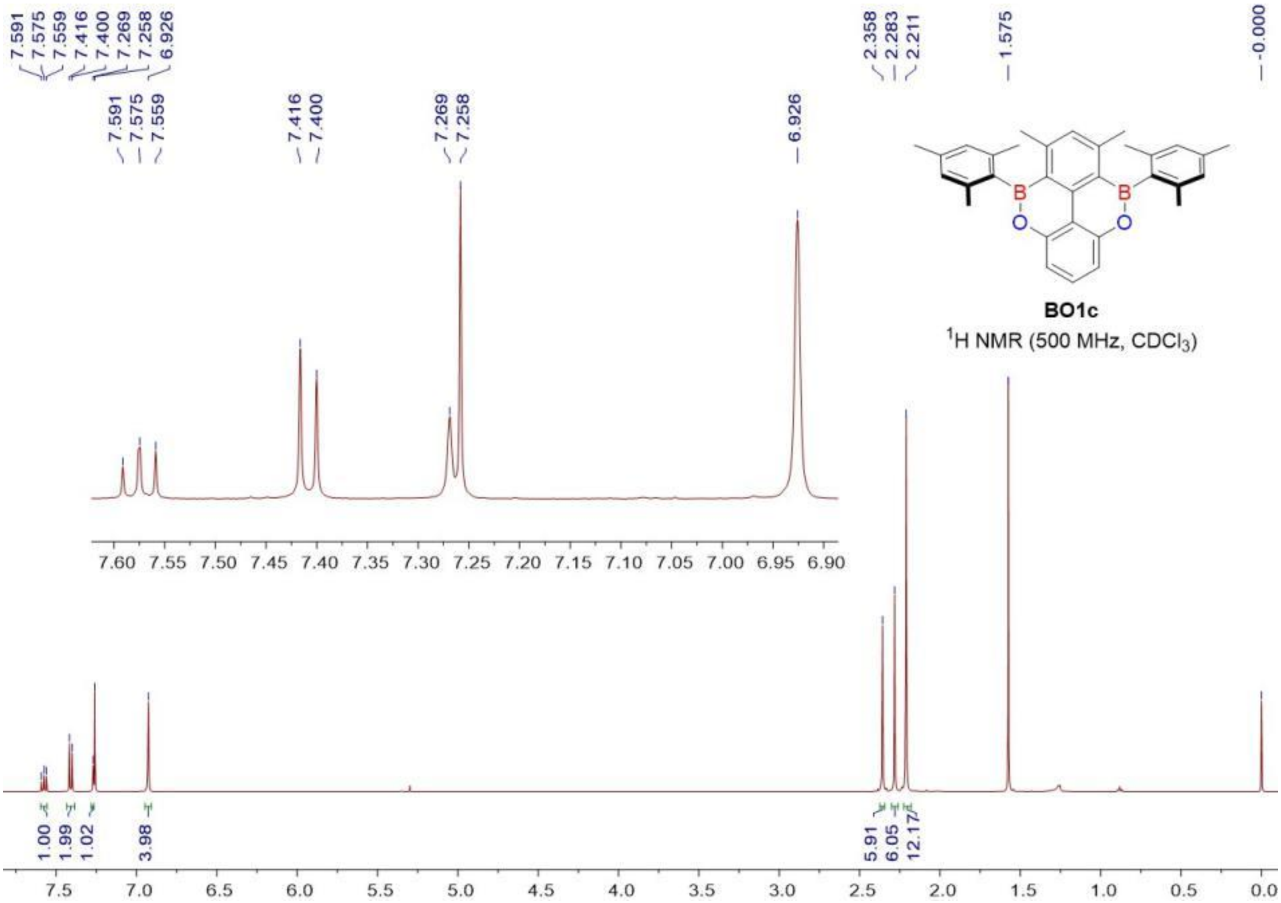
7.591
7.575
7.559
7.416
7.400
7.269
7.258
6.926
2.358
2.283
2.211
1.575
-0.000
BO1c
1H NMR (500 MHz, CDCl3)
1.00
1.99
1.02
3.98
5.91
6.05
12.17

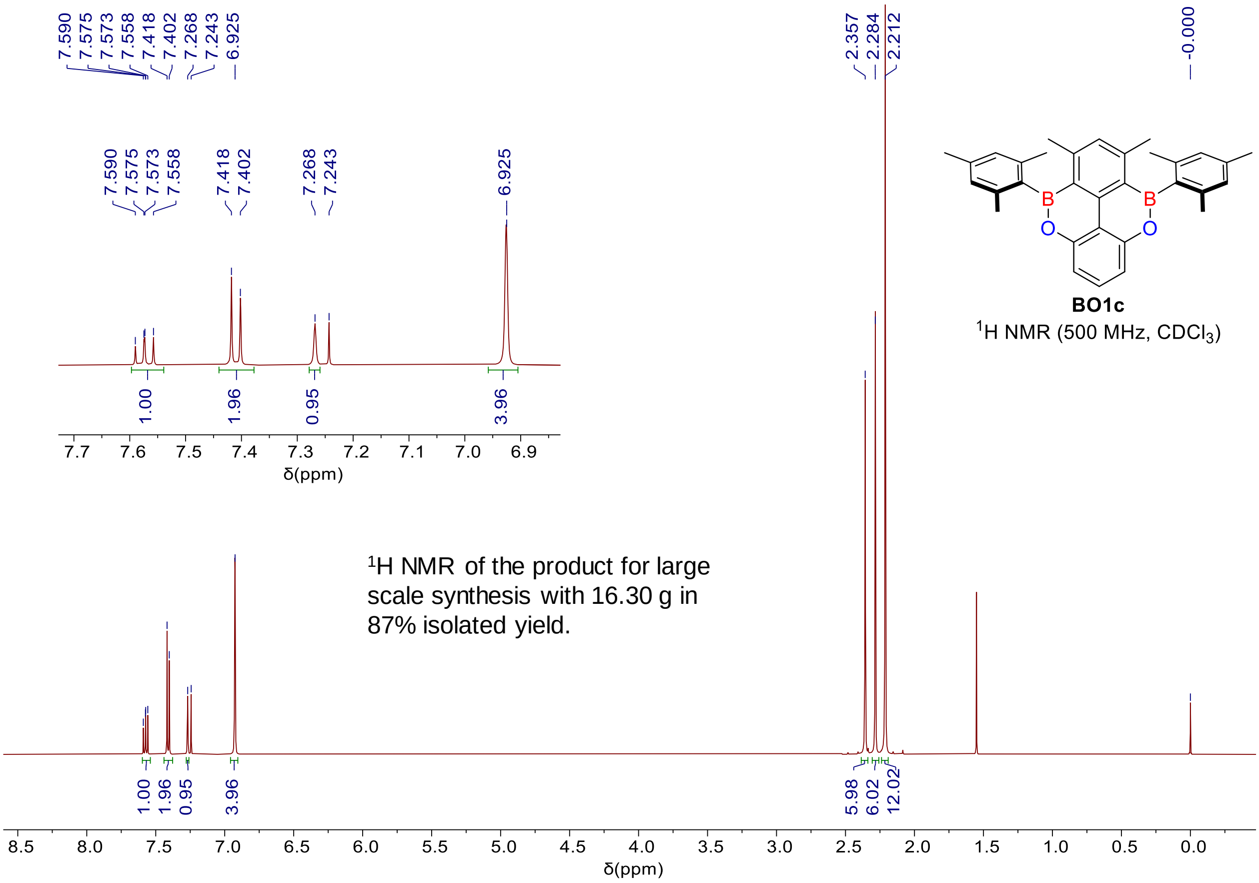
7.590
7.575
7.573
7.558
7.418
7.402
7.268
7.243
6.925
2.357
2.284
2.212
-0.000
BO1c
1H NMR (500 MHz, CDCl3)
1.00
1.96
0.95
3.96
δ(ppm)
1H NMR of the product for large scale synthesis with 16.30 g in 87% isolated yield.
5.98
6.02
12.02
δ(ppm)

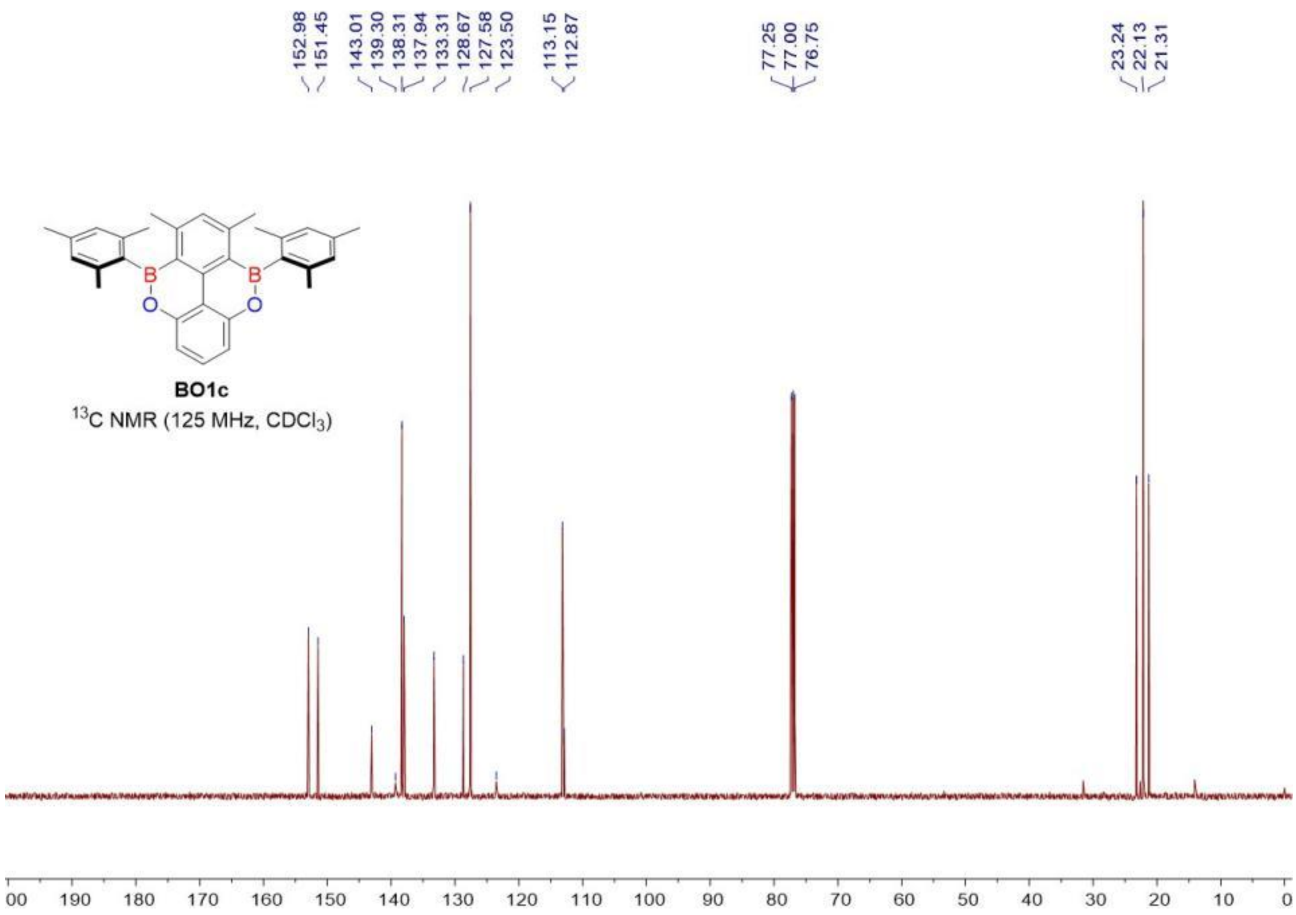
152.98
151.45
143.01
139.30
138.31
137.94
133.31
128.67
127.58
123.50
113.15
112.87
77.25
77.00
76.75
23.24
22.13
21.31
BO1c
^{13}C NMR (125 MHz, $CDCl_3$)
00 190 180 170 160 150 140 130 120 110 100 90 80 70 60 50 40 30 20 10 0

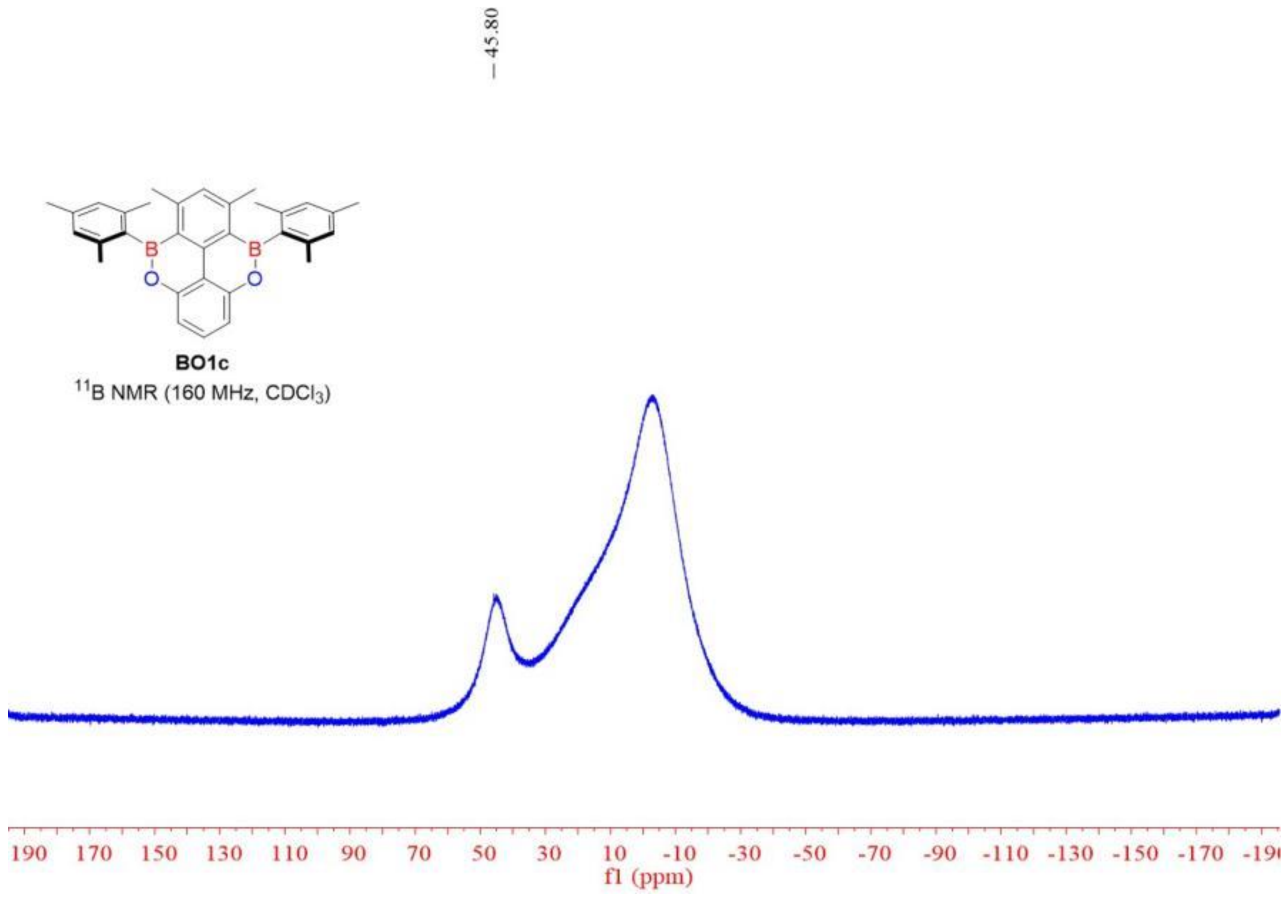
45.80
BO1c
^{11}B NMR (160 MHz, $CDCl_3$)
190 170 150 130 110 90 70 50 30 10 -10 -30 -50 -70 -90 -110 -130 -150 -170 -19
f1 (ppm)

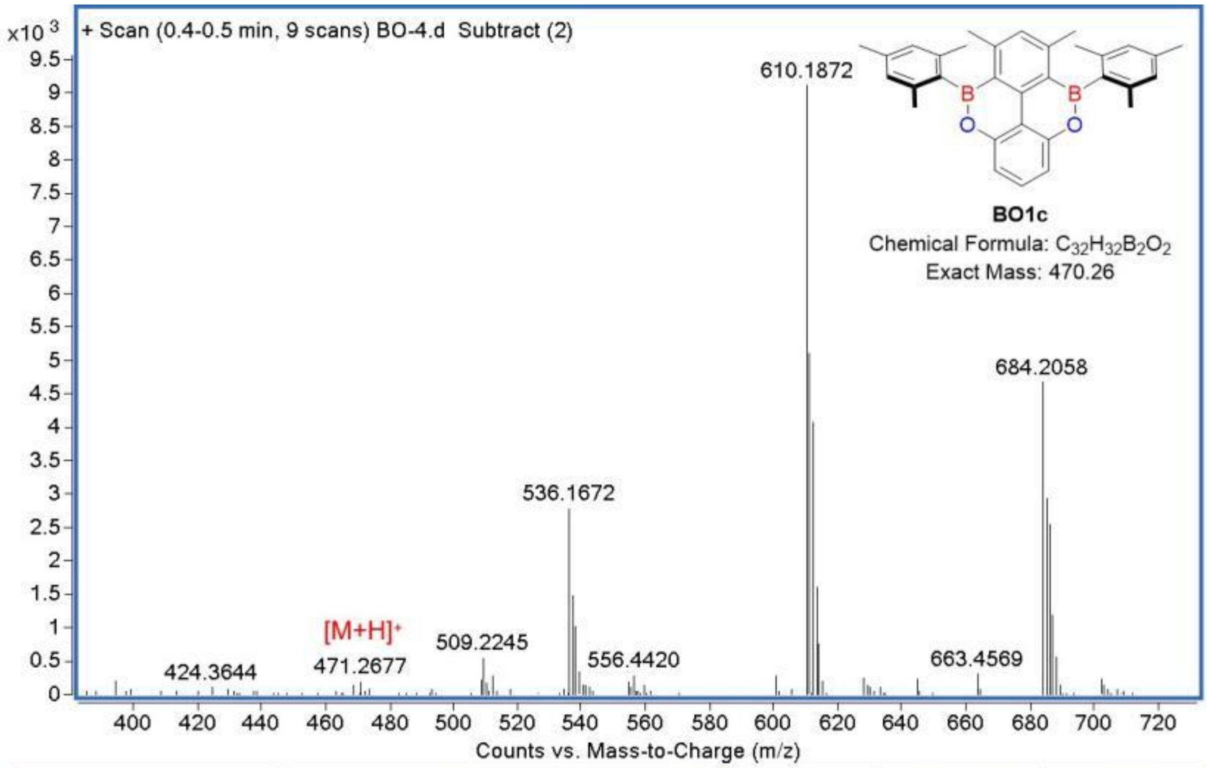

Formula (M)	Ion Formula	m/z	Calc m/z	Diff (ppm)	DBE
$C_{32}H_{32}[^{11}B]_2O_2$	$C_{32}H_{33}[^{11}B]_2O_2$	471.2677	471.2661	-3.36	18

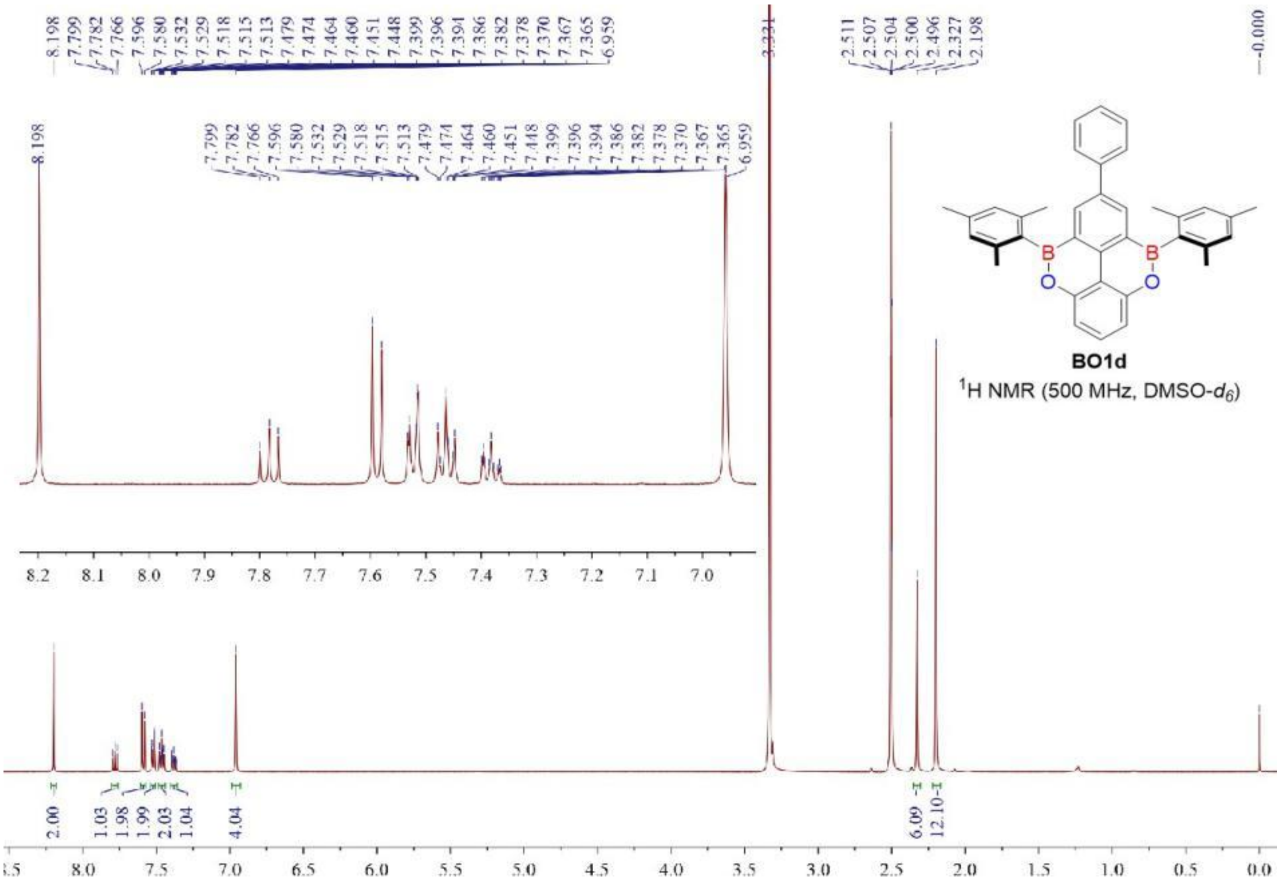

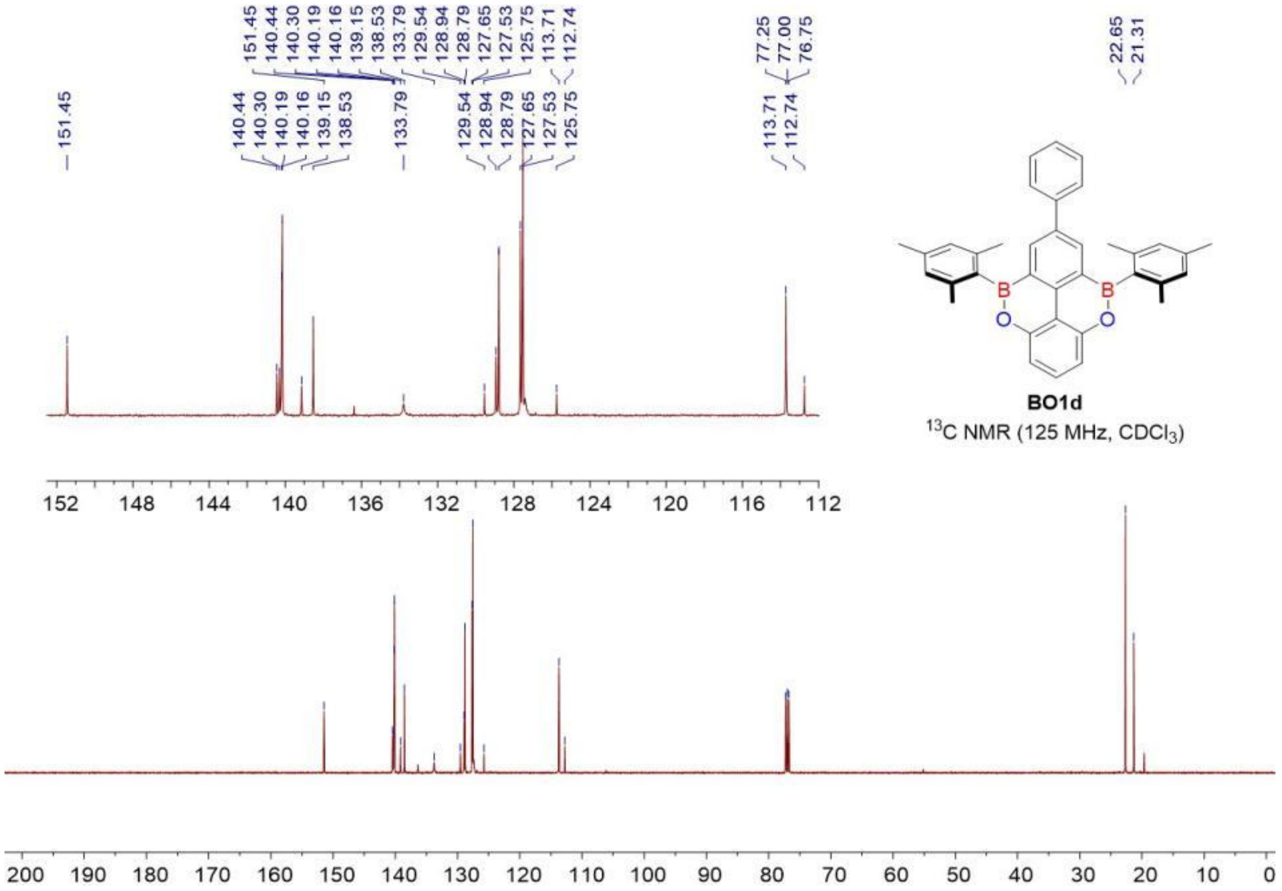
BO1d
^{13}C NMR (125 MHz, $CDCl_3$)

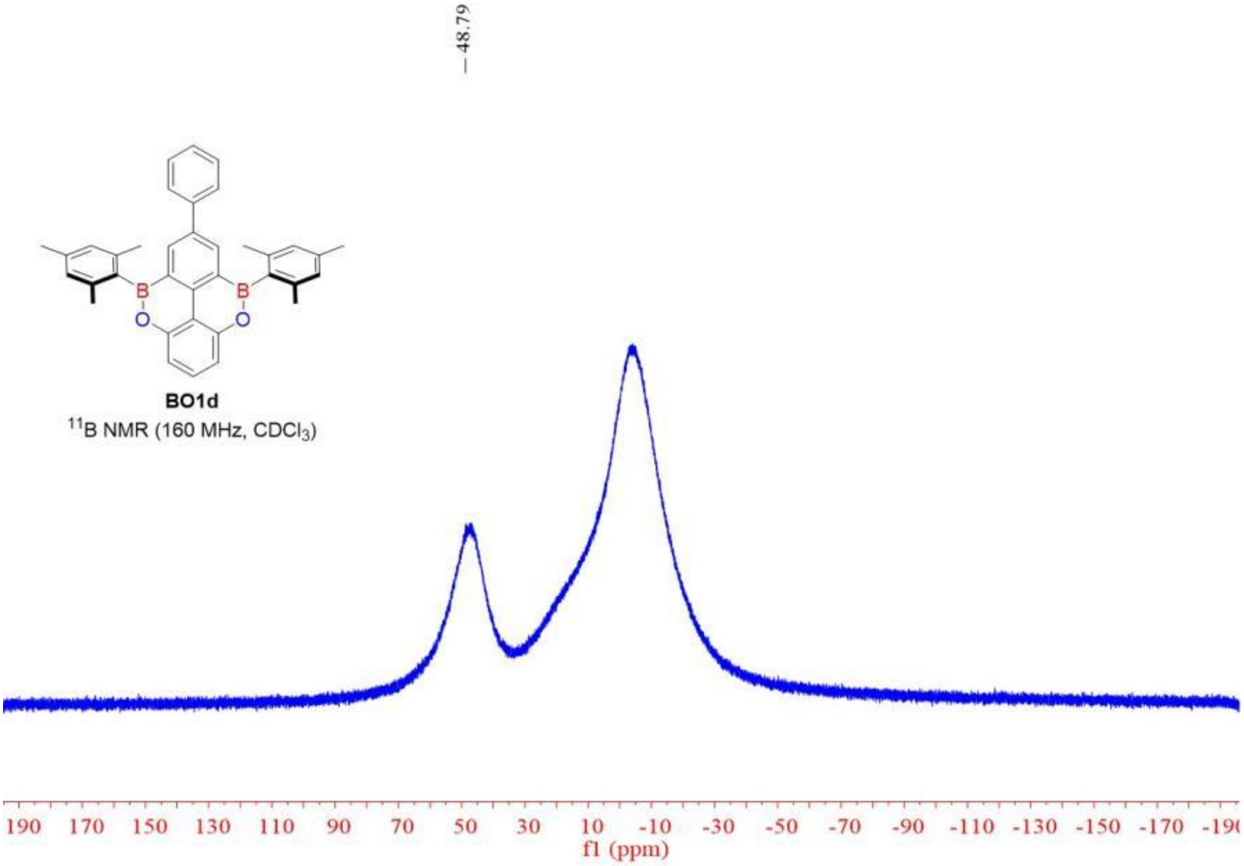
BO1d
^{11}B NMR (160 MHz, $CDCl_3$)
48.79
f1 (ppm)

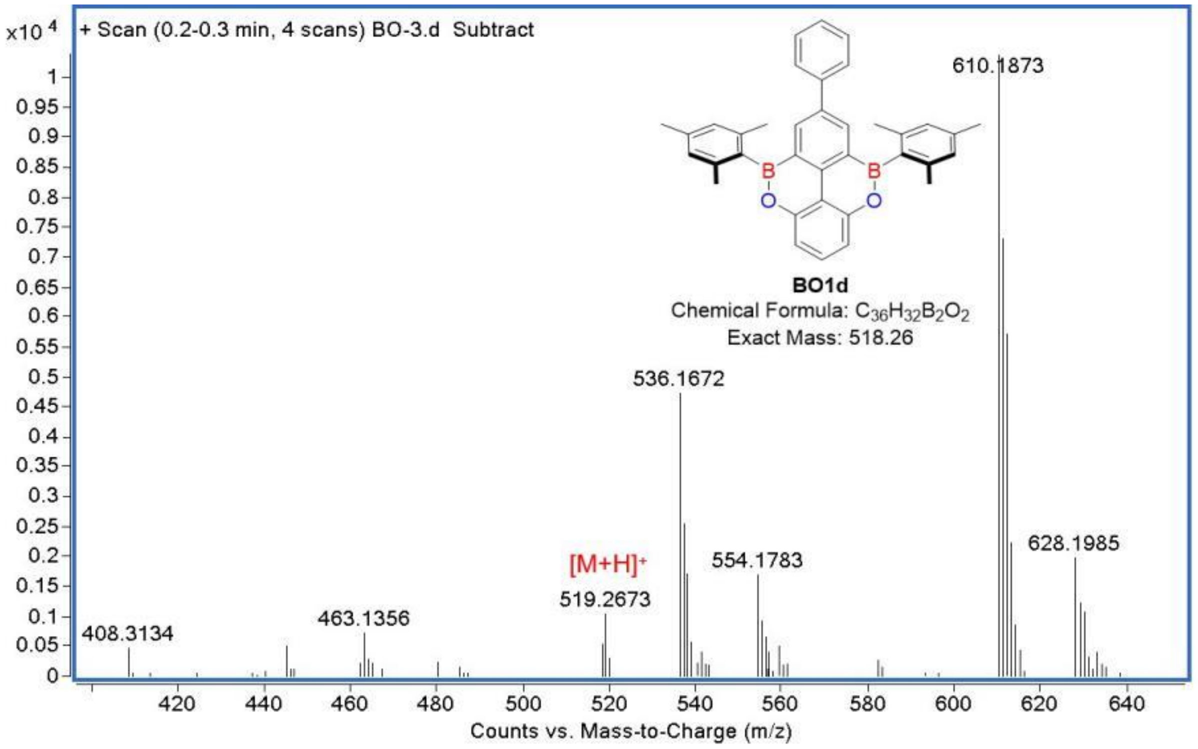

Formula (M)	Ion Formula	m/z	Calc m/z	Diff (ppm)	DBE
$C_{36}H_{32}[^{11}B]_2O_2$	$C_{36}H_{33}[^{11}B]_2O_2$	519.2673	519.2661	-2.28	22

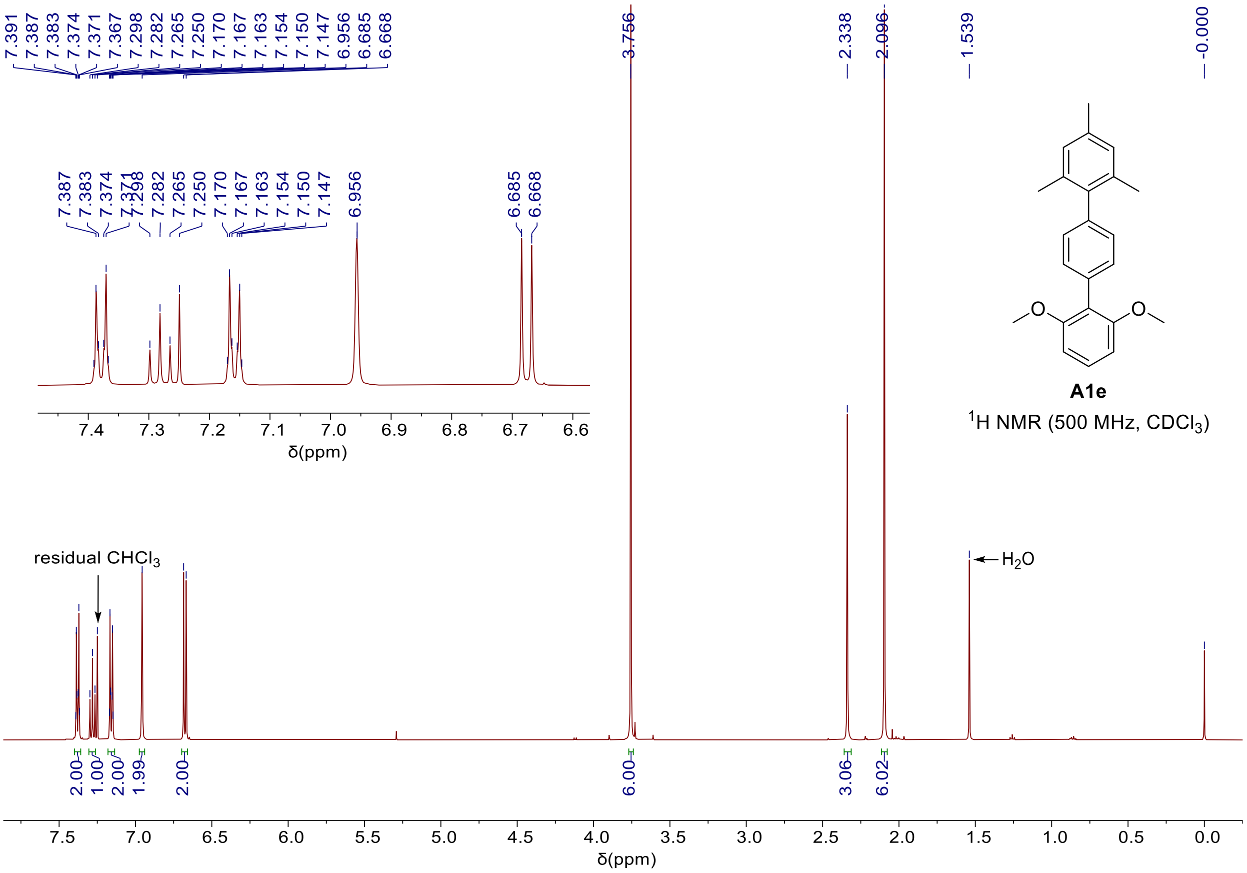

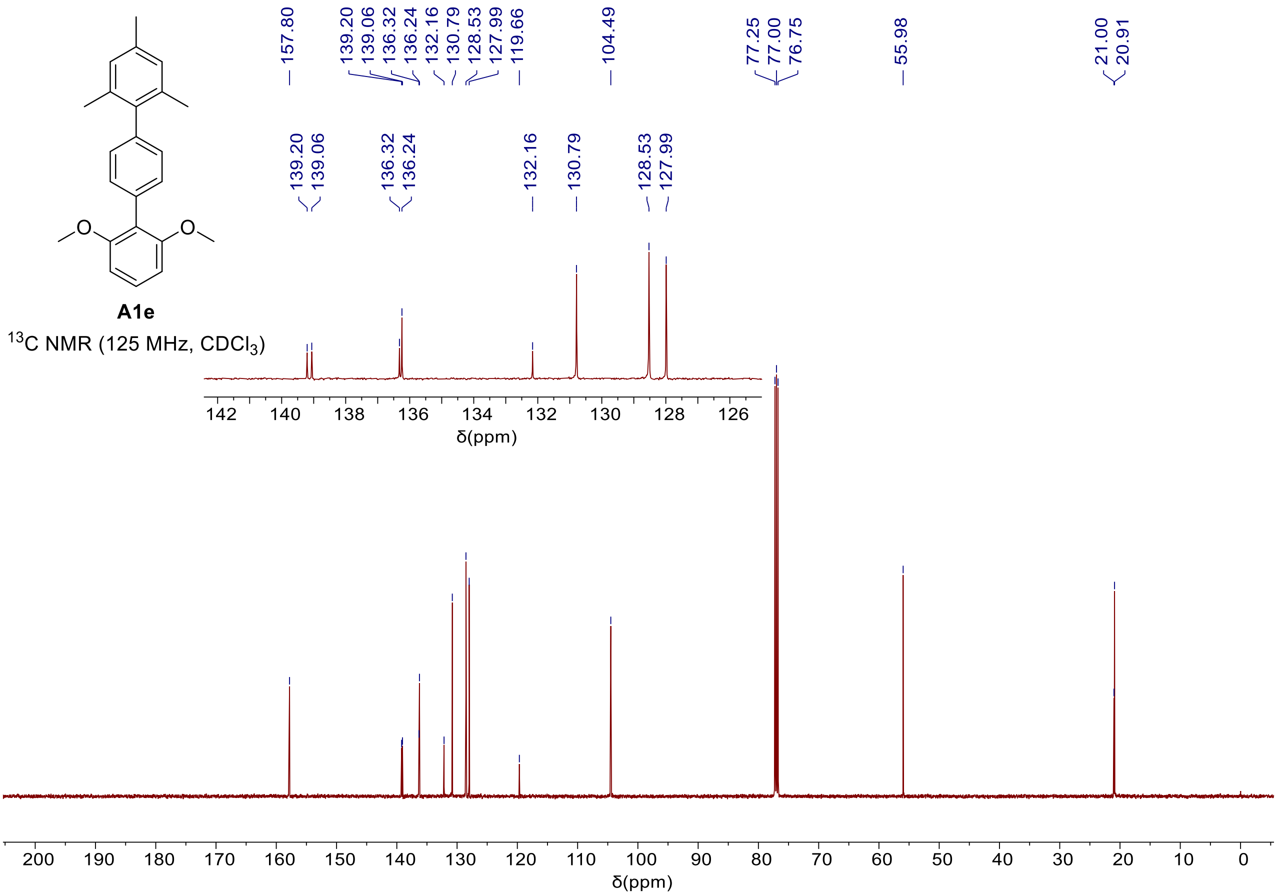
A1e
^{13}C NMR (125 MHz, CDCl$_3$)
157.80
139.20
139.06
136.32
136.24
132.16
130.79
128.53
127.99
119.66
104.49
77.25
77.00
76.75
55.98
21.00
20.91
δ(ppm)

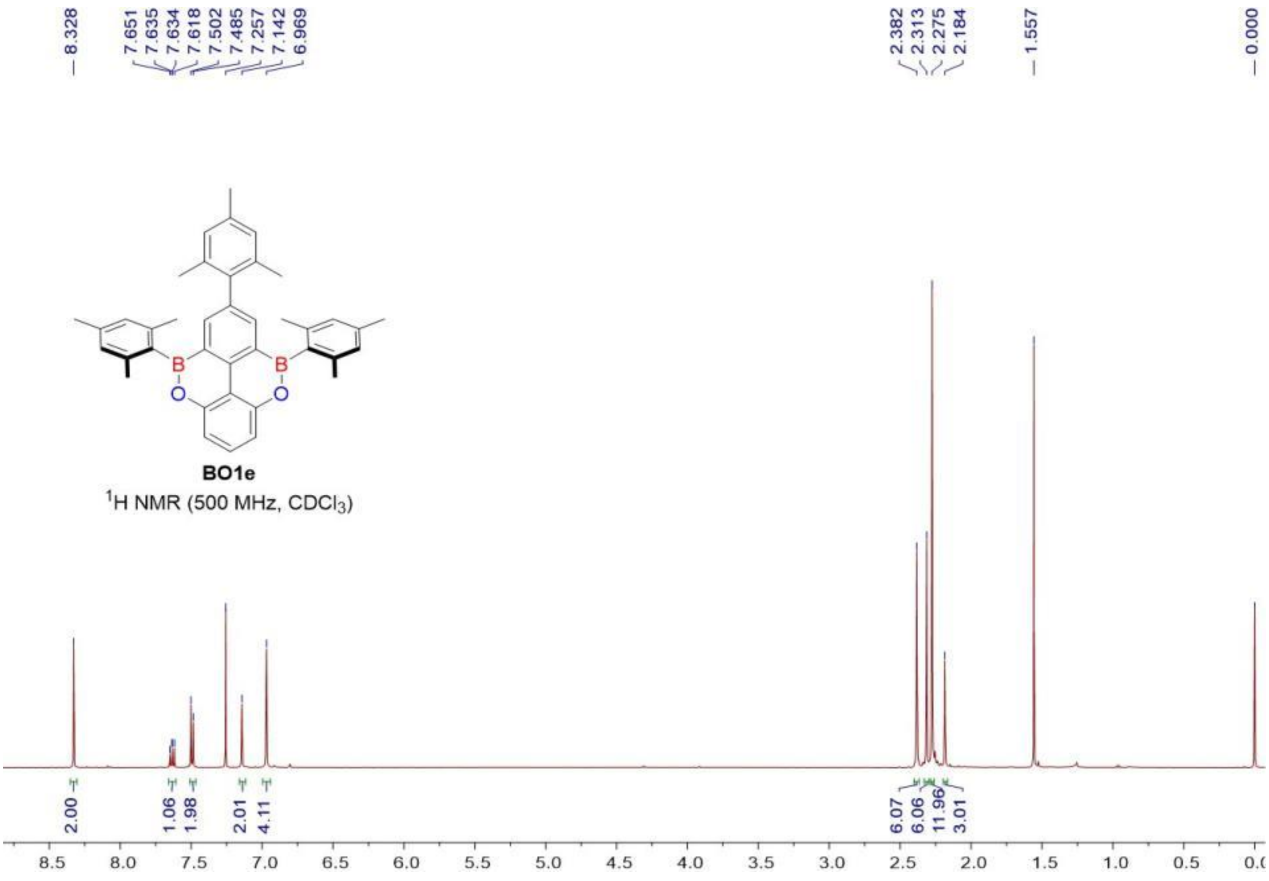
BO1e
^{1}H NMR (500 MHz, CDCl$_3$)
8.328
7.651
7.635
7.634
7.618
7.502
7.485
7.257
7.142
6.969
2.382
2.313
2.275
2.184
1.557
0.000
2.00
1.06
1.98
2.01
4.11
6.07
6.06
11.96
3.01

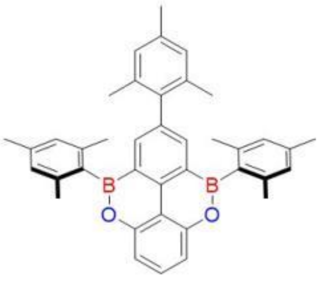

BO1e

^{13}C NMR (125 MHz, $CDCl_3$)

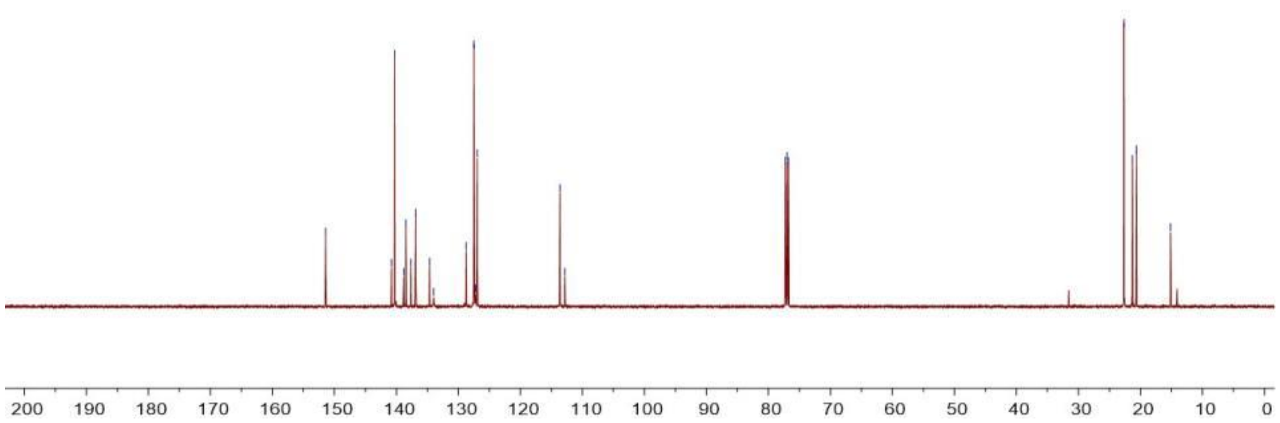

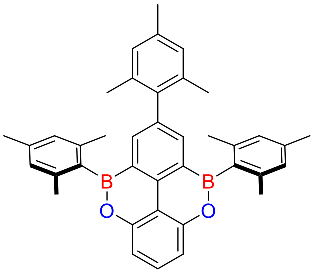

BO1e

^{11}B NMR (160 MHz, $CDCl_3$)

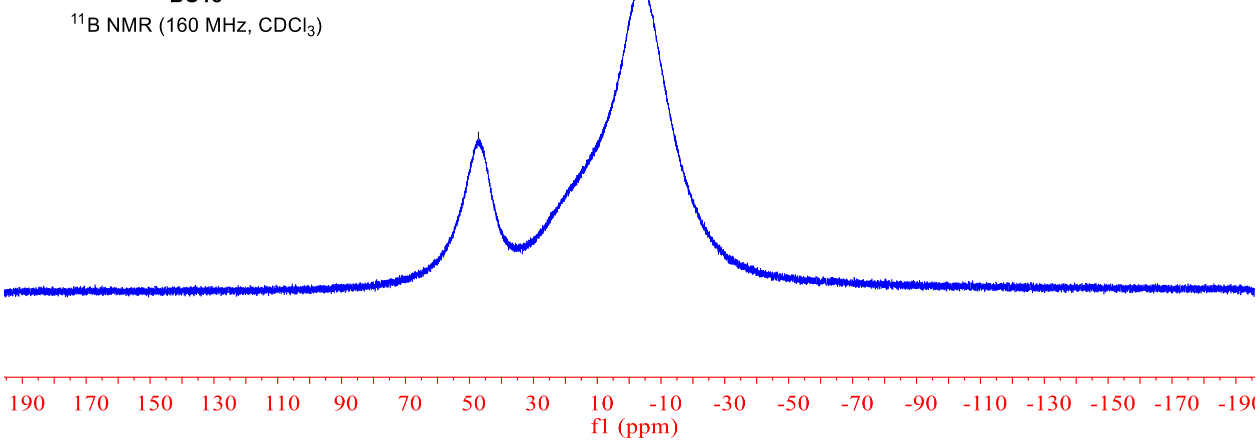

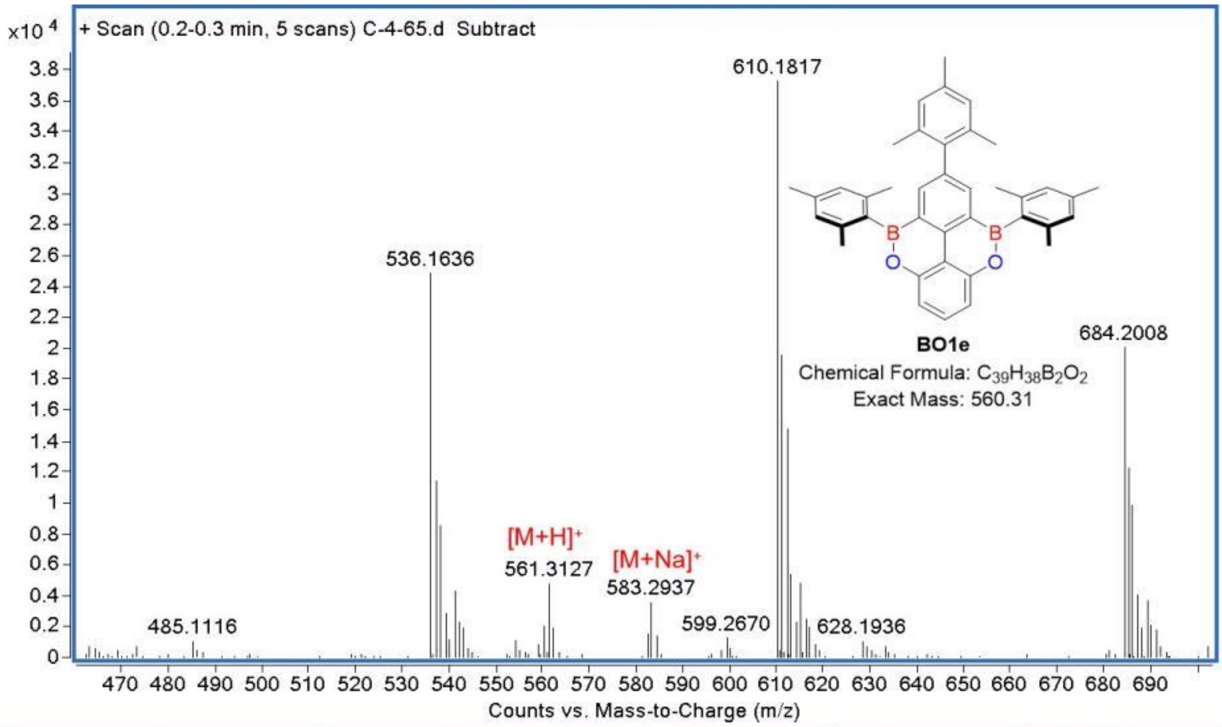

Formula (M)	Ion Formula	m/z	Calc m/z	Diff (ppm)
$C_{39}H_{38}[^{11}B]_2O_2$	$C_{39}H_{39}[^{11}B]_2O_2$	561.3127	561.3131	0.66

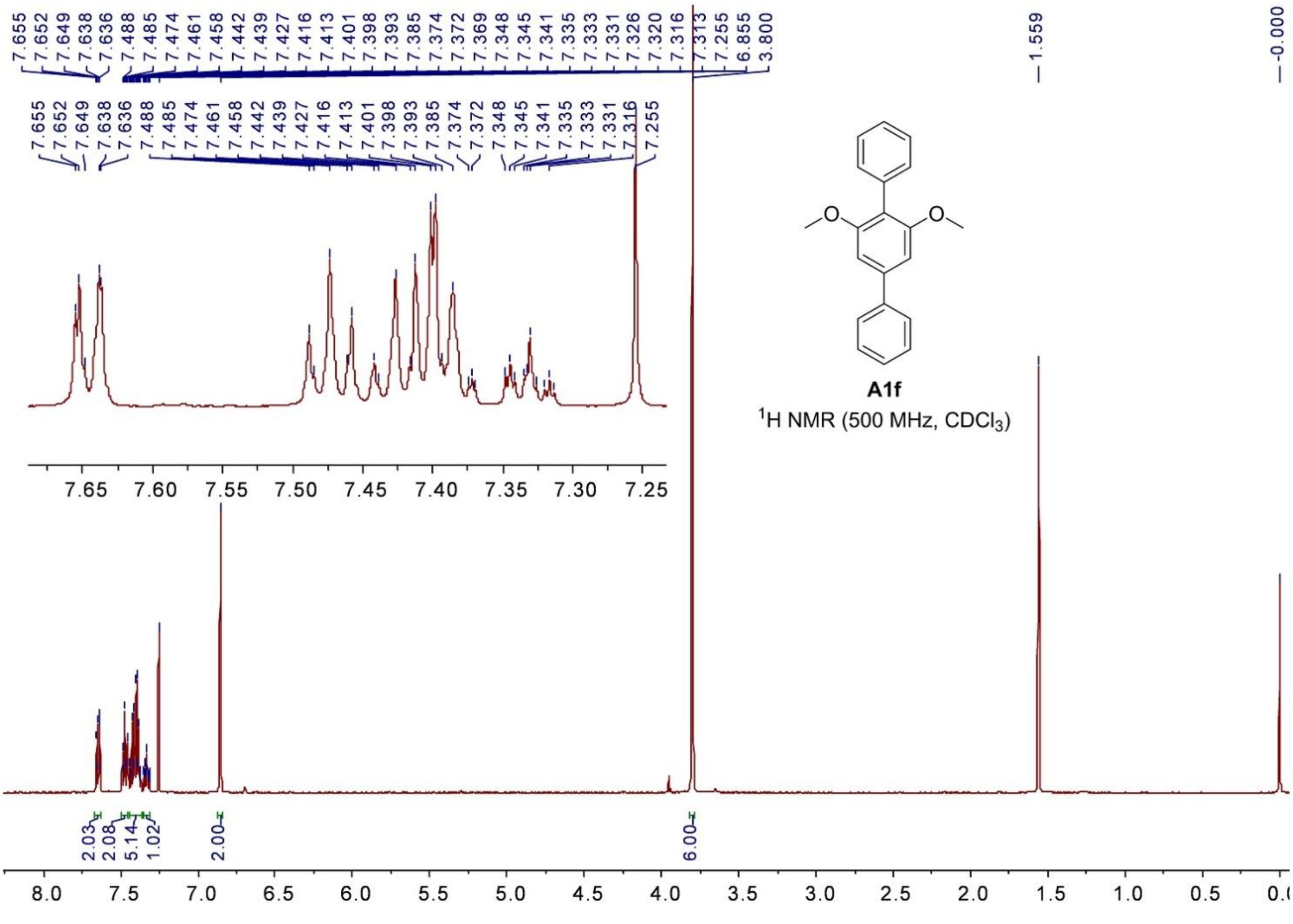

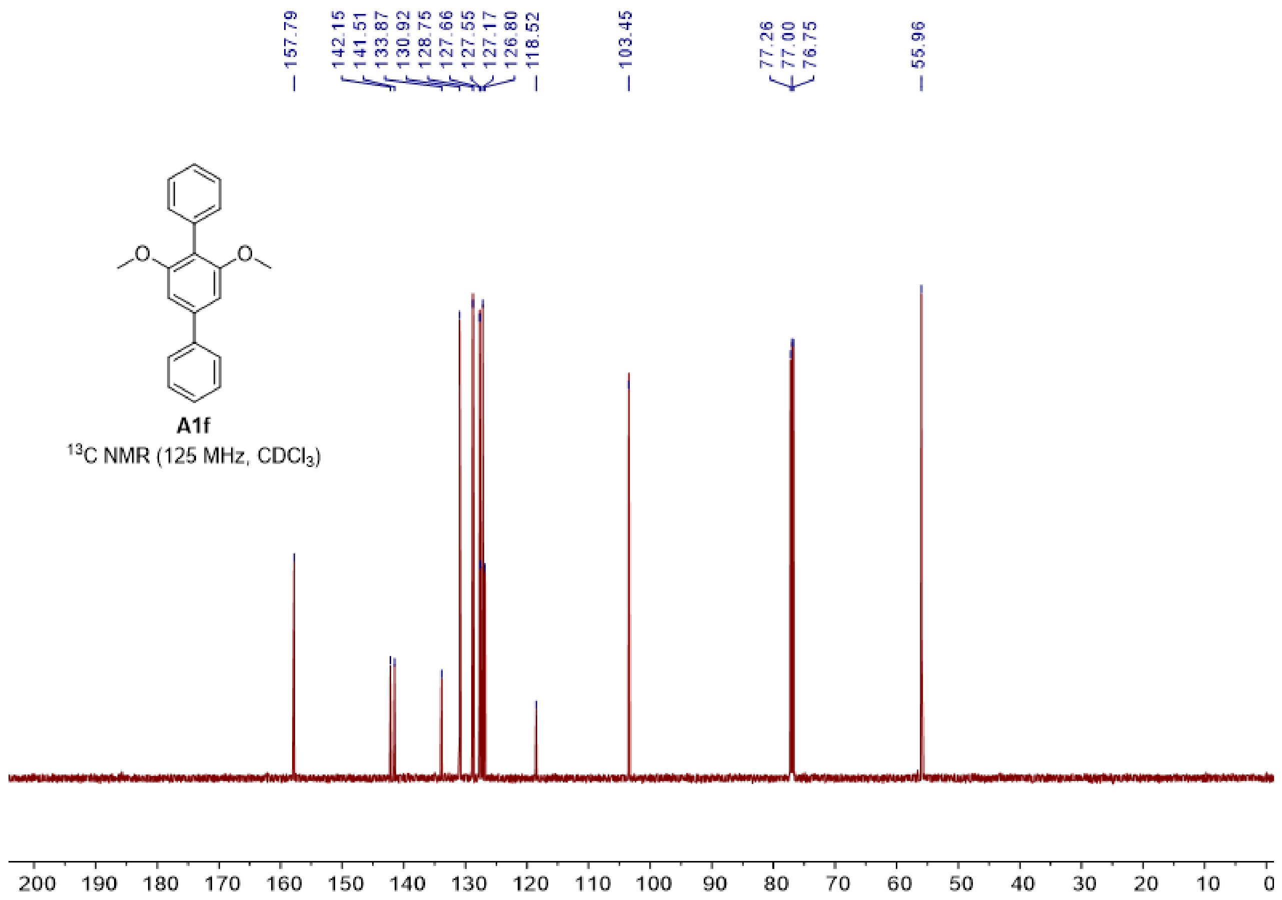

157.79
142.15
141.51
133.87
130.92
128.75
127.66
127.55
127.17
126.80
118.52
103.45
77.26
77.00
76.75
55.96
A1f
^{13}C NMR (125 MHz, CDCl$_3$)

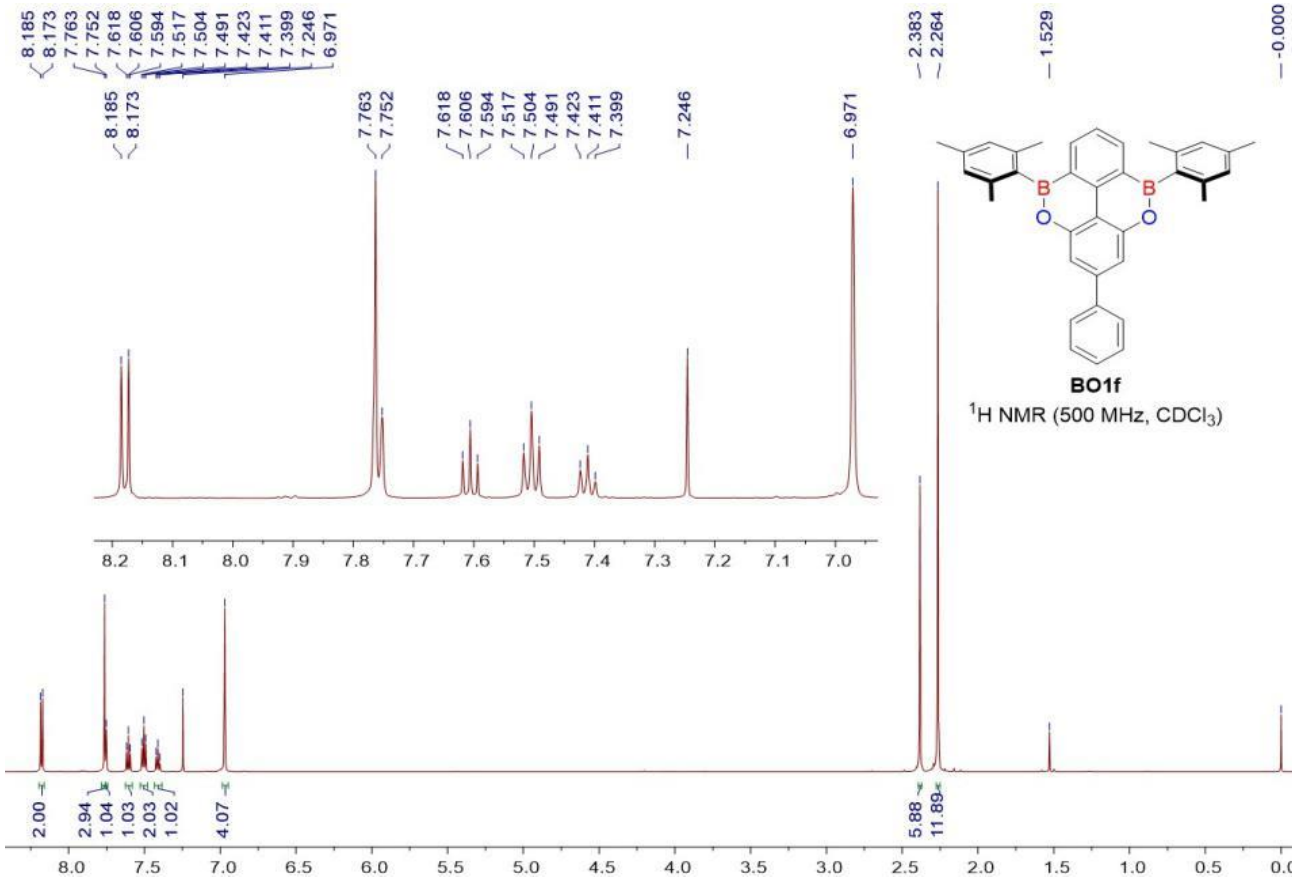

8.185
8.173
7.763
7.752
7.618
7.606
7.594
7.517
7.504
7.491
7.423
7.411
7.399
7.246
6.971
2.383
2.264
1.529
-0.000
B
O
BO1f
^{1}H NMR (500 MHz, CDCl$_3$)
2.00
2.94
1.04
1.03
2.03
1.02
4.07
5.88
11.89

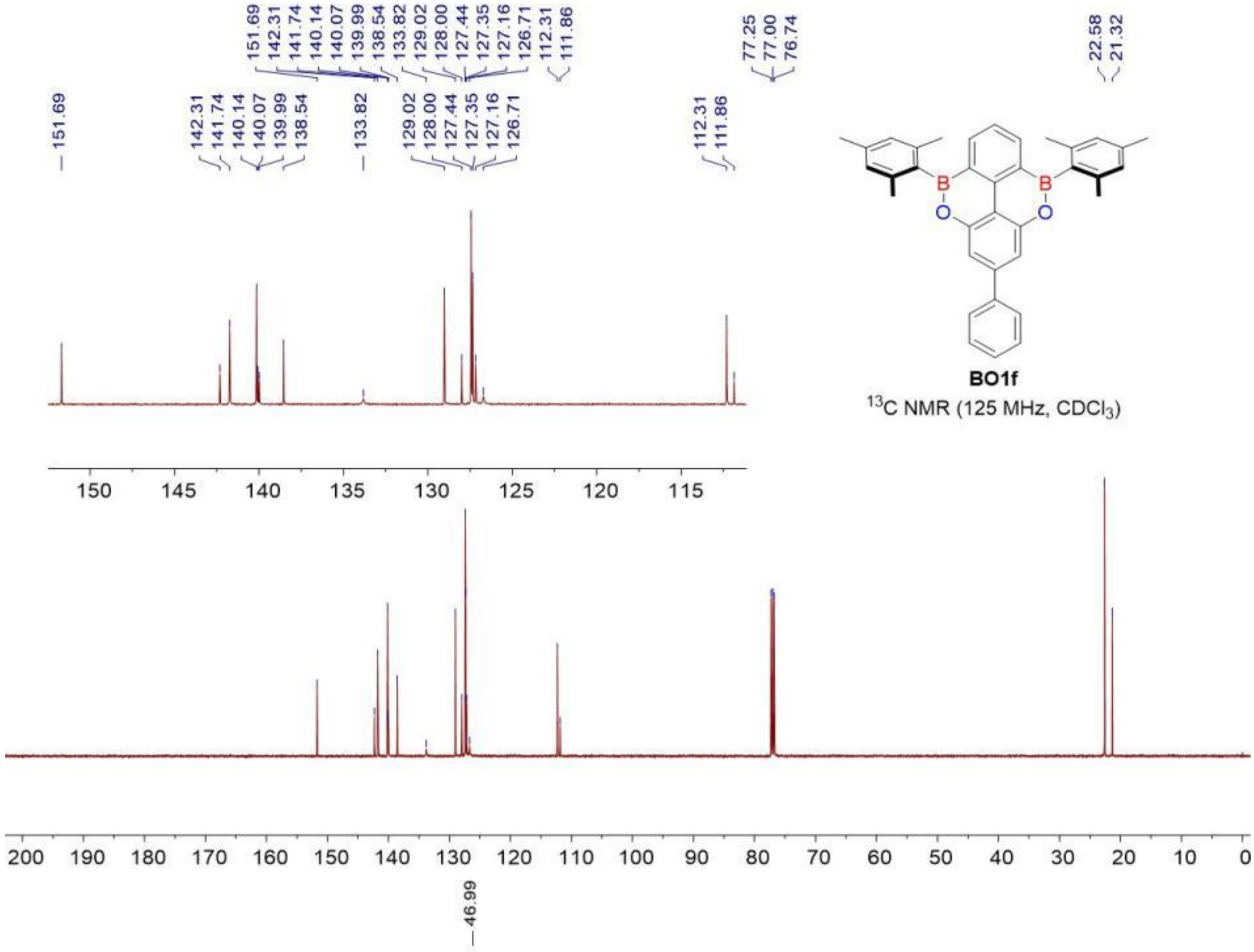
151.69
142.31
141.74
140.14
140.07
139.99
138.54
133.82
129.02
128.00
127.44
127.35
127.16
126.71
112.31
111.86
77.25
77.00
76.74
22.58
21.32
BO1f
^{13}C NMR (125 MHz, $CDCl_3$)
150 145 140 135 130 125 120 115
200 190 180 170 160 150 140 130 120 110 100 90 80 70 60 50 40 30 20 10 0

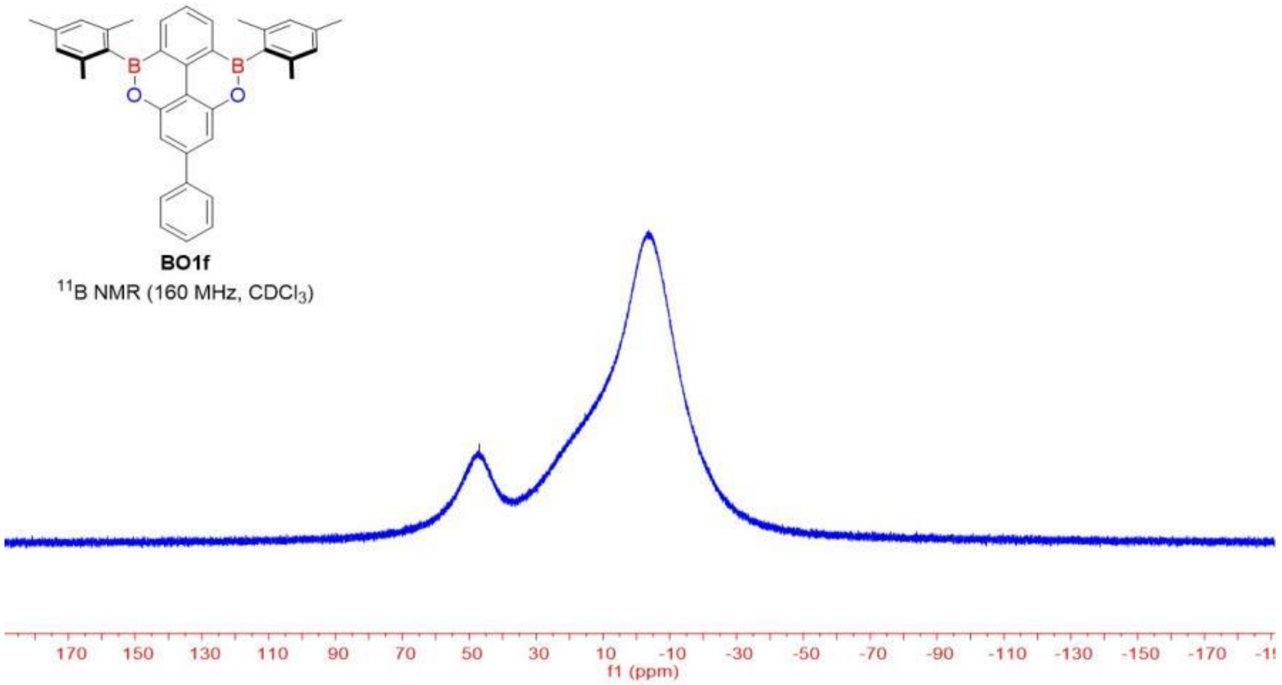
46.99
BO1f
^{11}B NMR (160 MHz, $CDCl_3$)
170 150 130 110 90 70 50 30 10 -10 -30 -50 -70 -90 -110 -130 -150 -170
f1 (ppm)

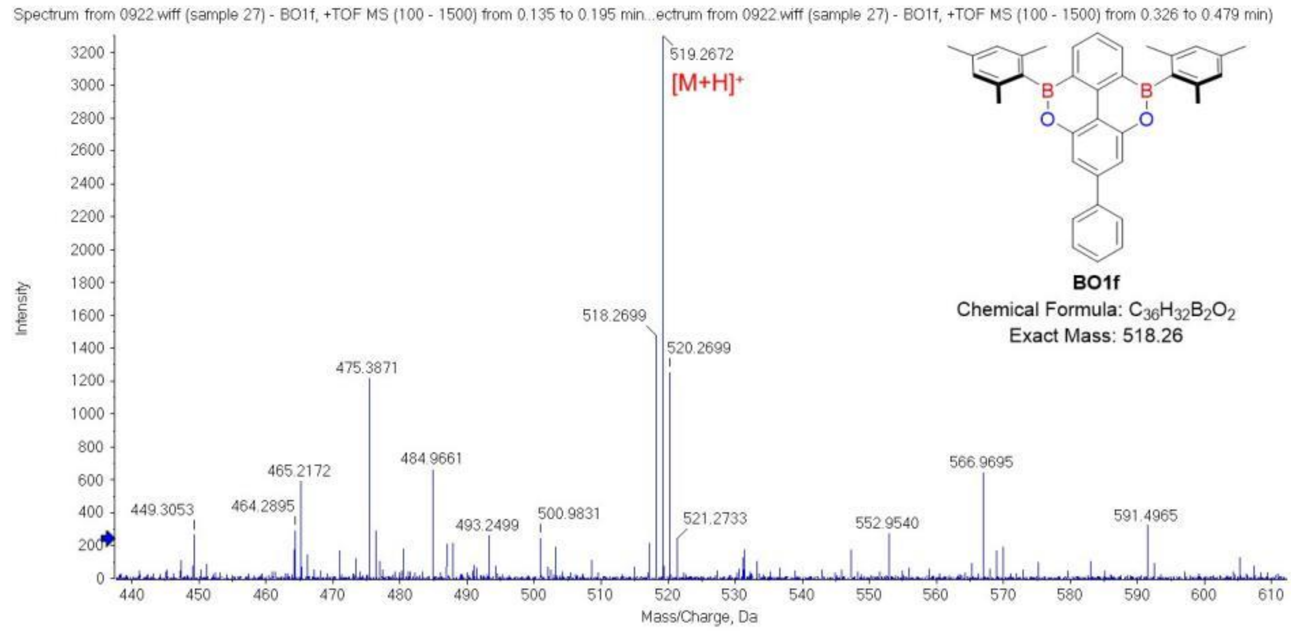

Formula (M)	Ion Formula	m/z	Calc m/z	Diff (ppm)
$C_{36}H_{32}[^{11}B]_2O_2$	$C_{36}H_{33}[^{11}B]_2O_2$	519.2672	519.2661	-2.2

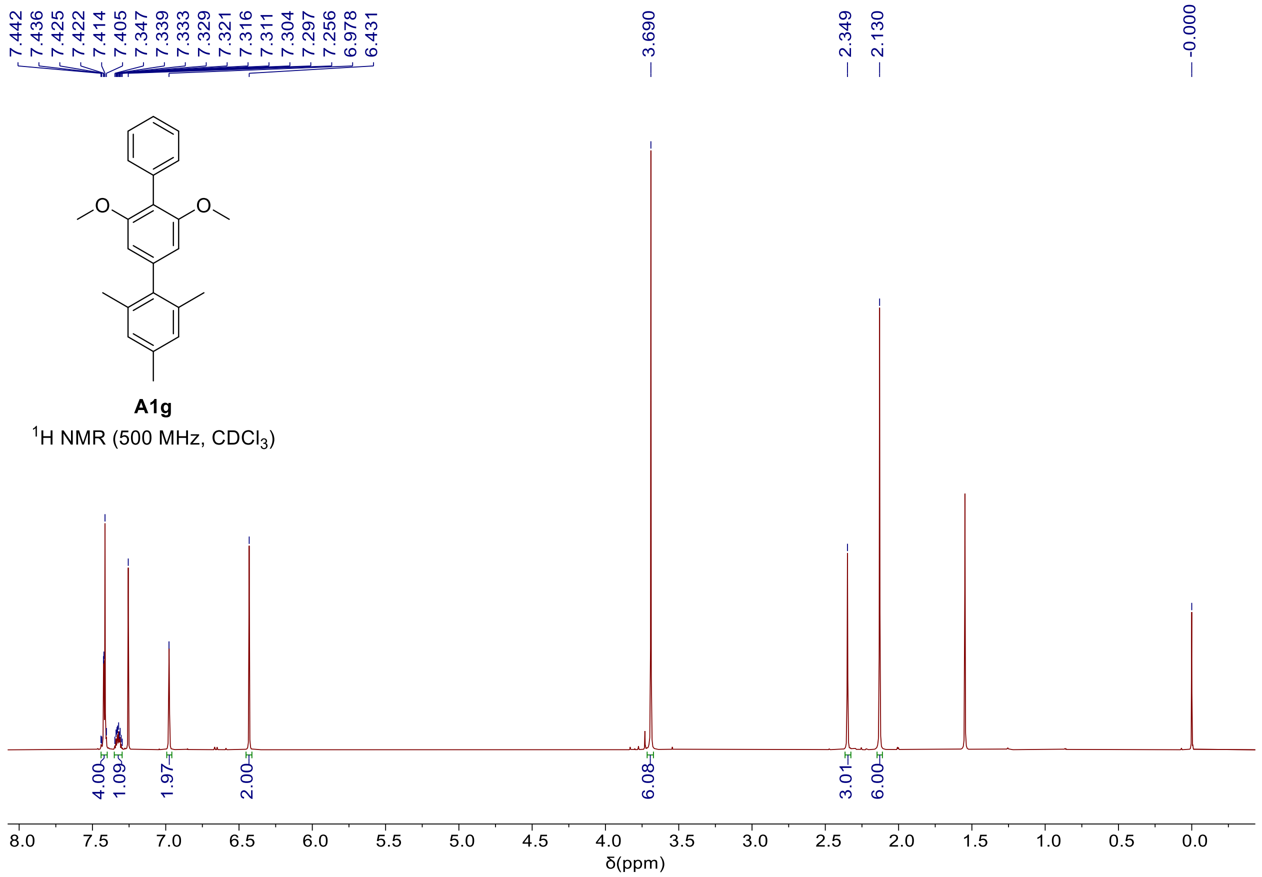

A1g

^{13}C NMR (125 MHz, $CDCl_3$)

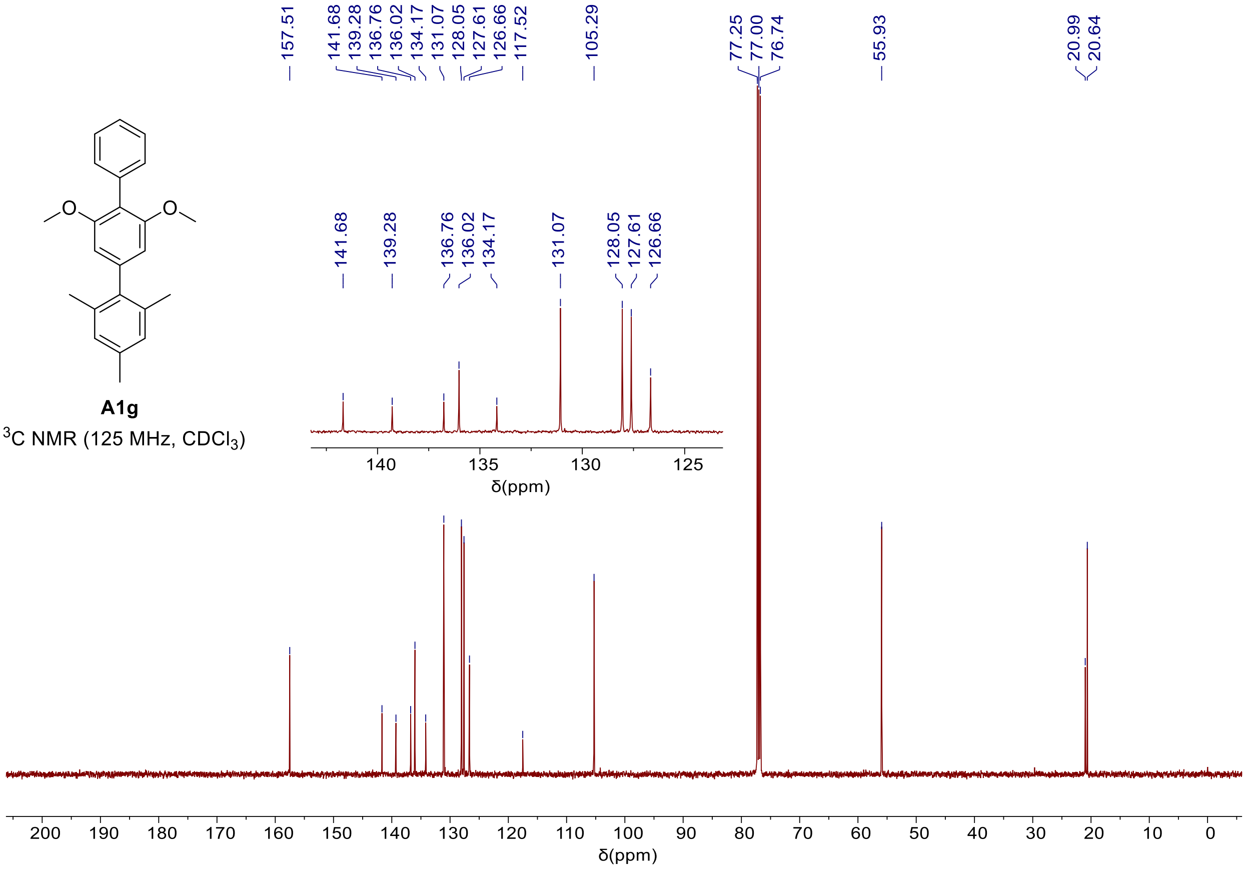

BO1g

^{1}H NMR (500 MHz, $CDCl_3$)

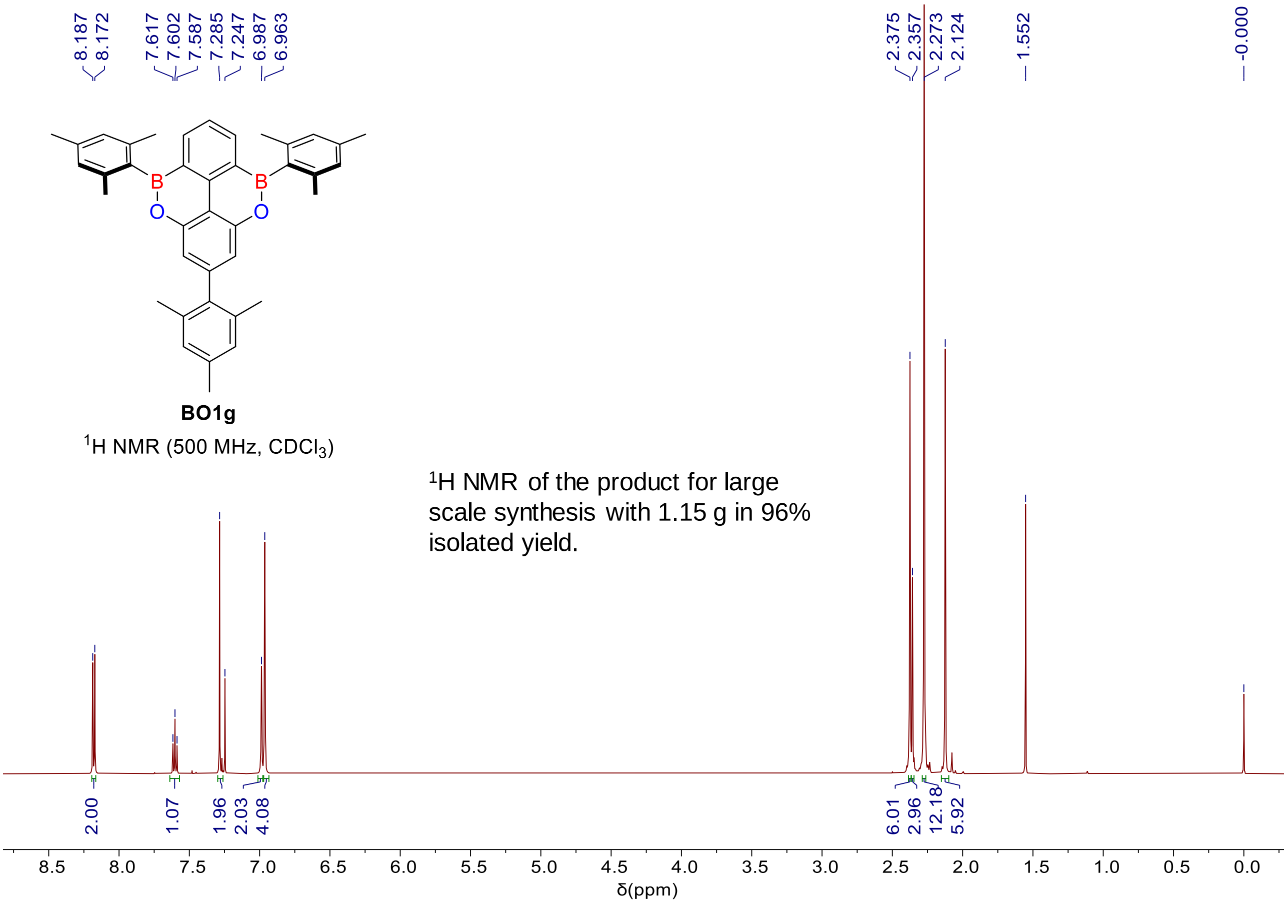

^{1}H NMR of the product for large scale synthesis with 1.15 g in 96% isolated yield.

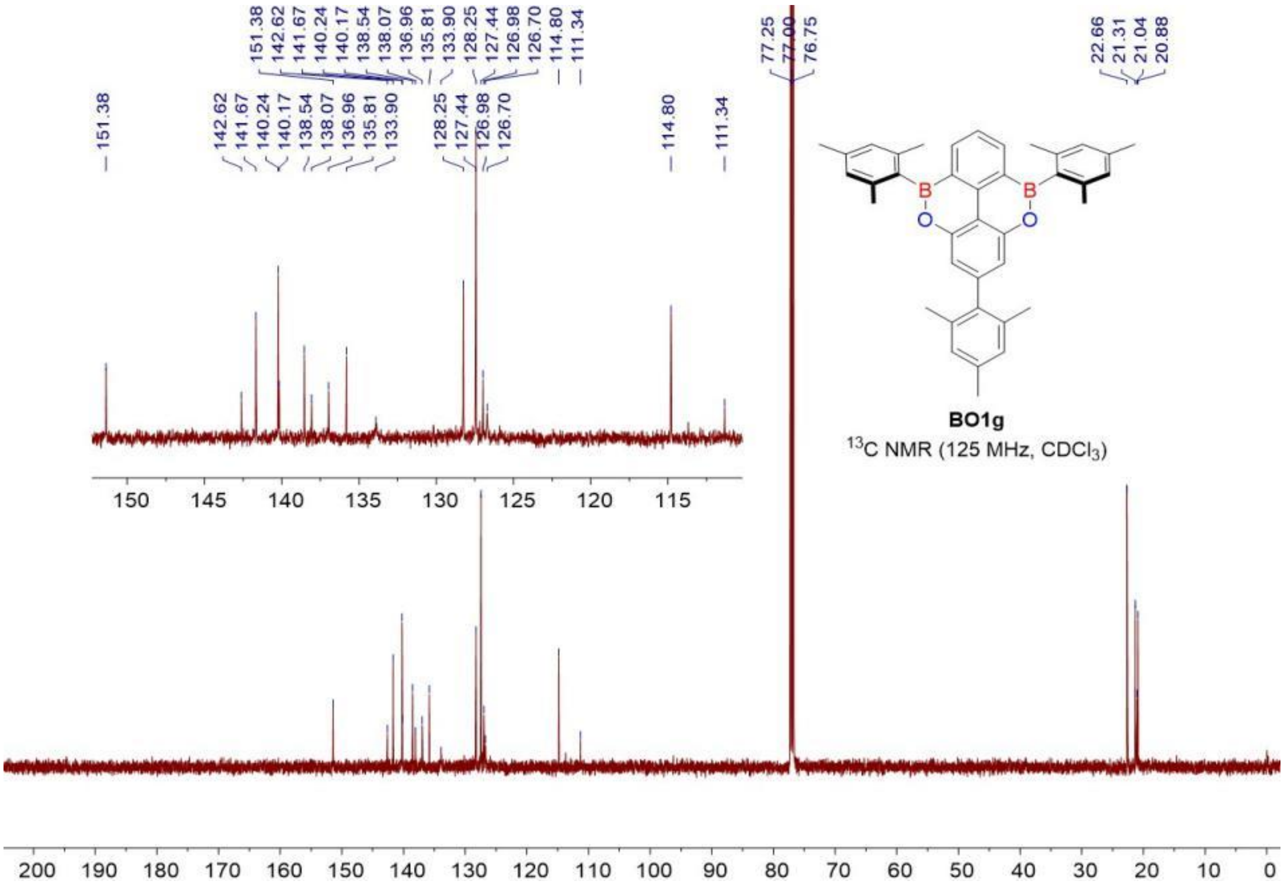
151.38
142.62
141.67
140.24
140.17
138.54
138.07
136.96
135.81
133.90
128.25
127.44
126.98
126.70
114.80
111.34
77.25
77.00
76.75
22.66
21.31
21.04
20.88
B
O
BO1g
13C NMR (125 MHz, CDCl3)

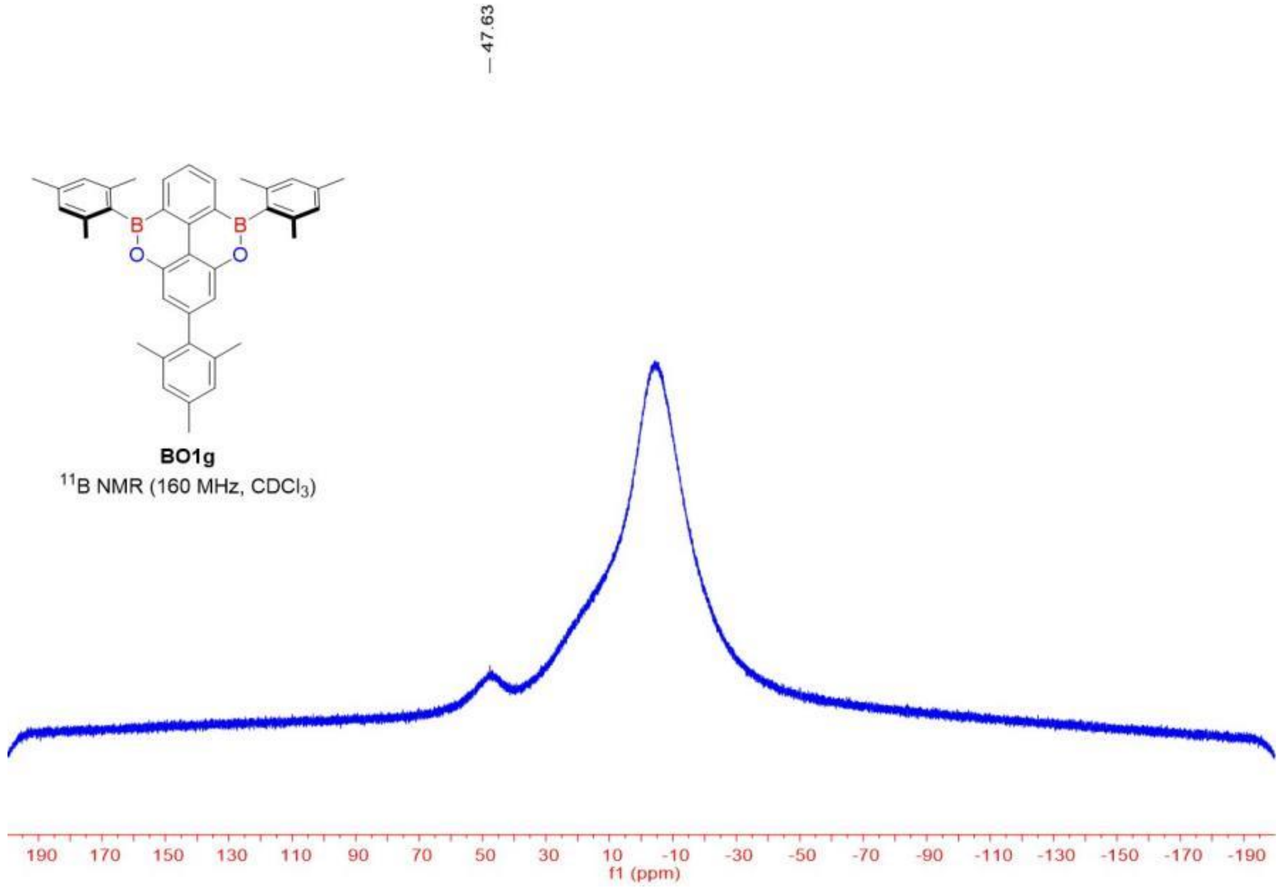
47.63
B
O
BO1g
11B NMR (160 MHz, CDCl3)
f1 (ppm)

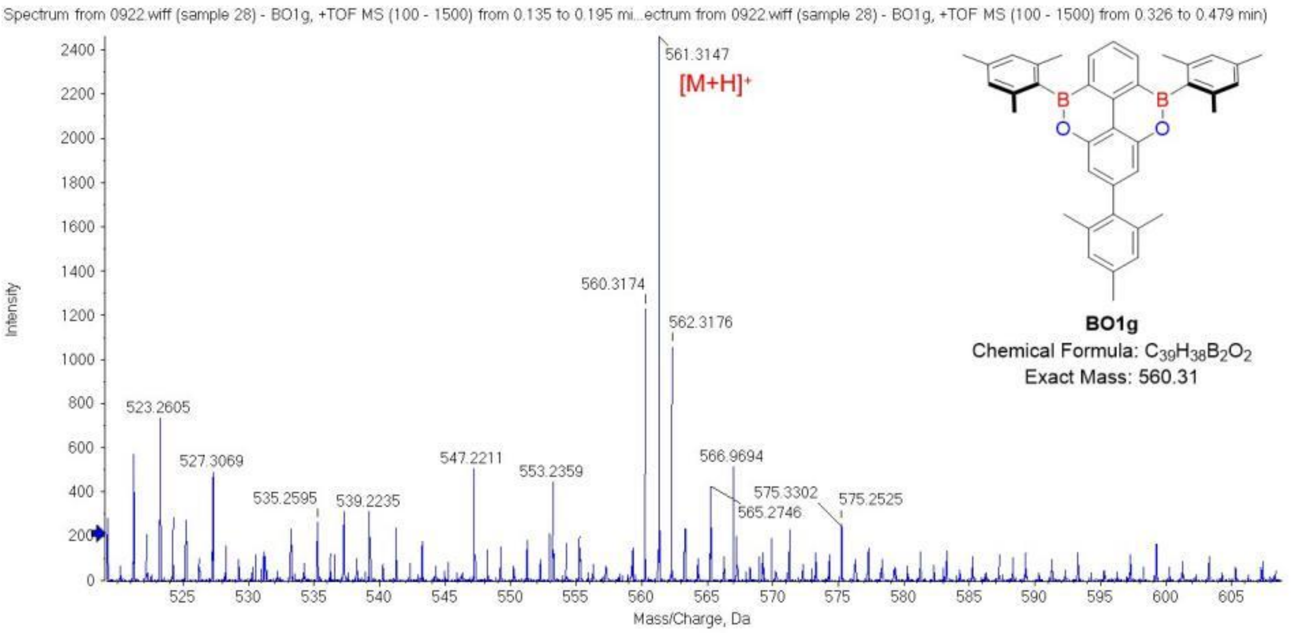

Formula (M)	Ion Formula	m/z	Calc m/z	Diff (ppm)
$C_{39}H_{38}[^{11}B]_2O_2$	$C_{39}H_{39}[^{11}B]_2O_2$	561.3147	561.3131	-2.9

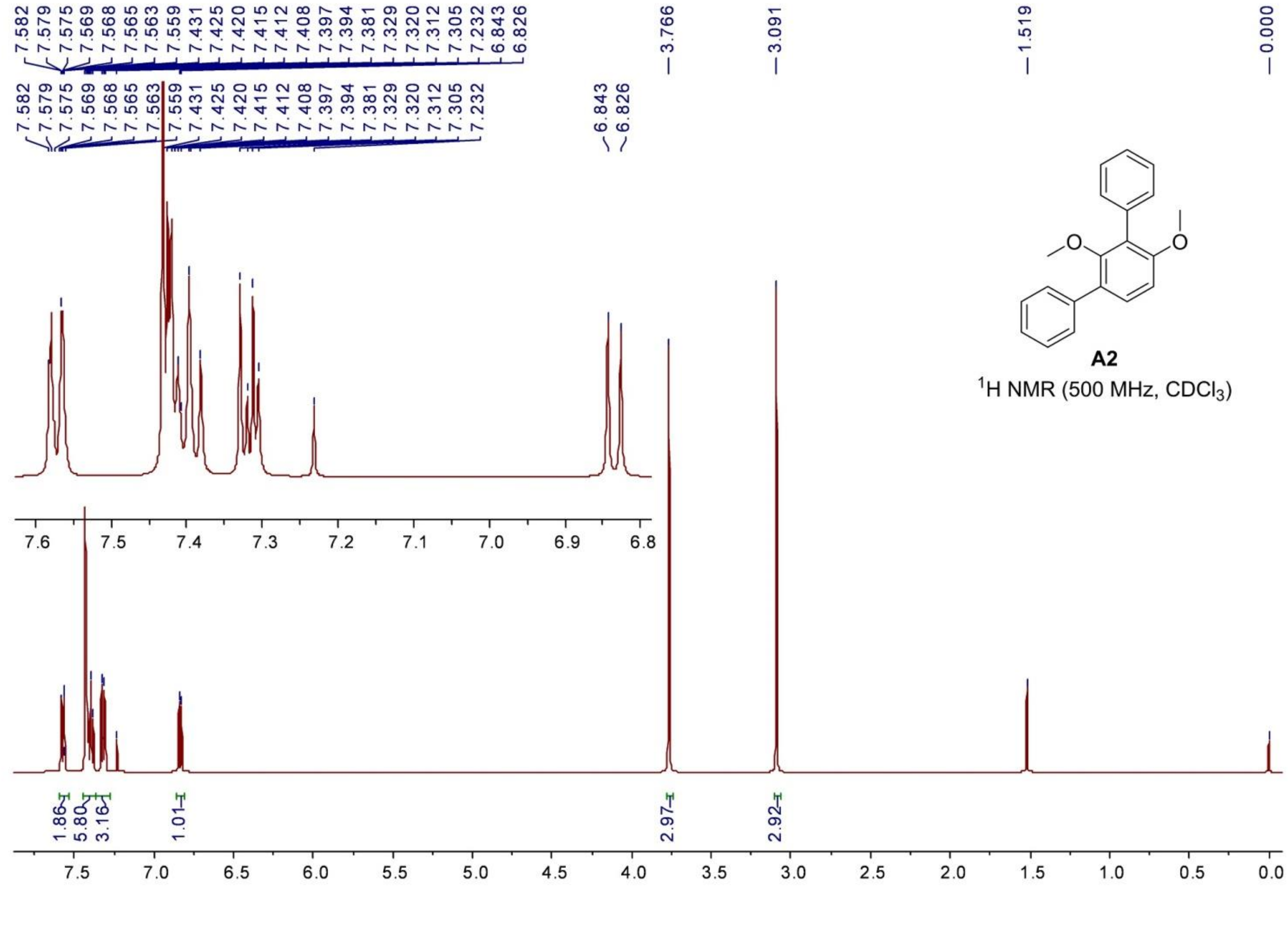

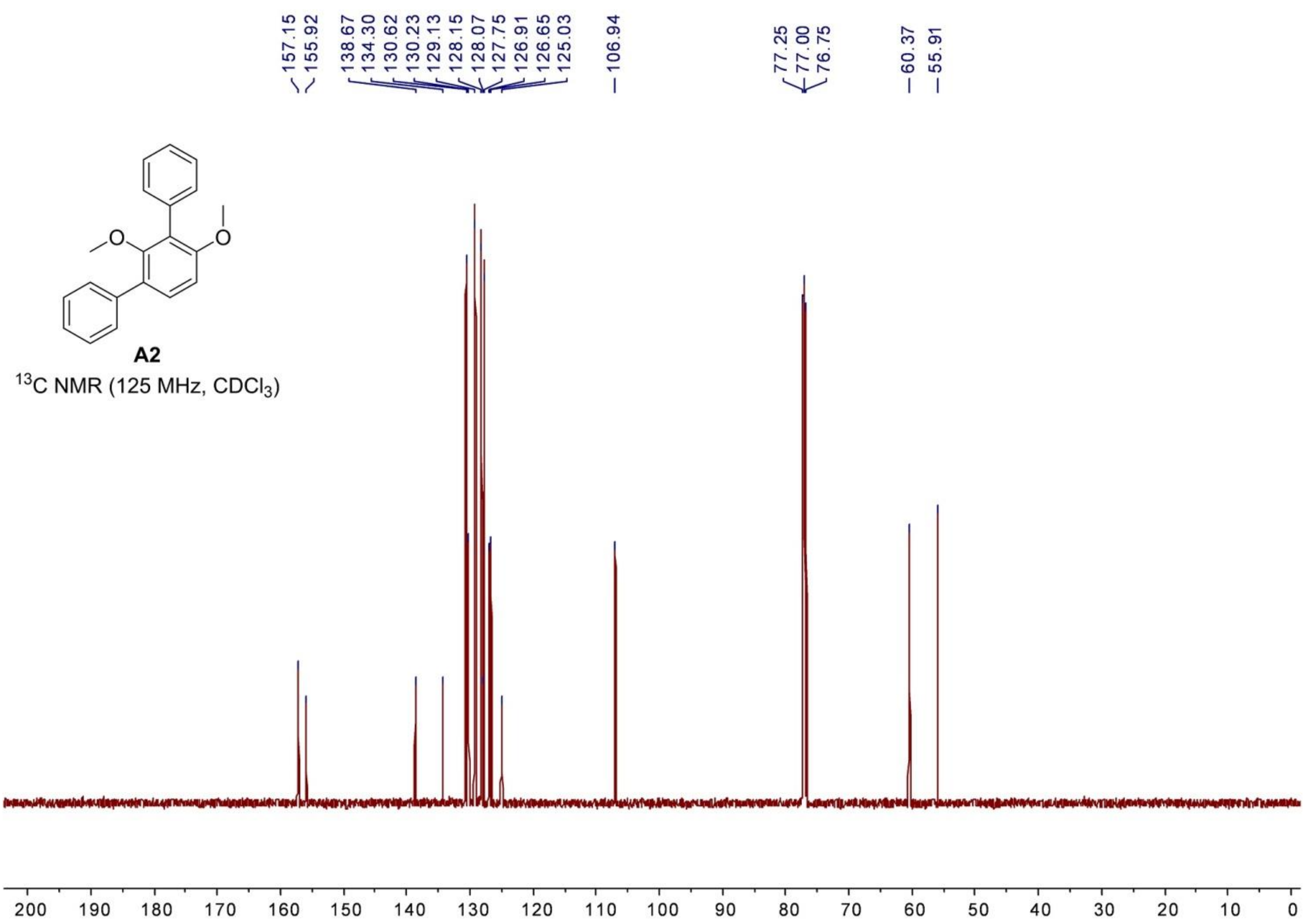
157.15
155.92
138.67
134.30
130.62
130.23
129.13
128.15
128.07
127.75
126.91
126.65
125.03
106.94
77.25
77.00
76.75
60.37
55.91
A2
13C NMR (125 MHz, CDCl3)

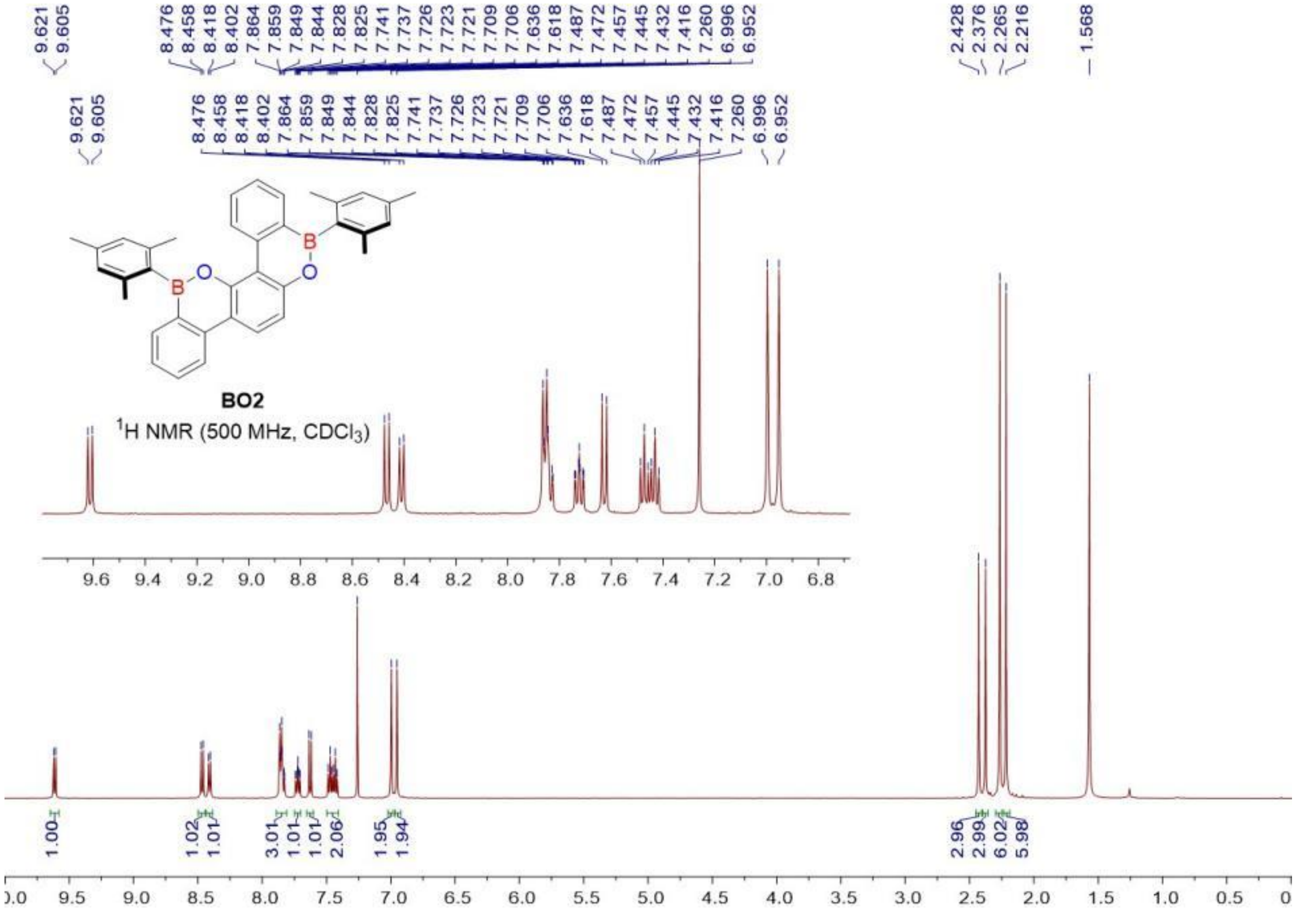
9.621
9.605
8.476
8.458
8.418
8.402
7.864
7.859
7.849
7.844
7.828
7.825
7.741
7.737
7.726
7.723
7.721
7.709
7.706
7.636
7.618
7.487
7.472
7.457
7.445
7.432
7.416
7.260
6.996
6.952
2.428
2.376
2.265
2.216
1.568
BO2
1H NMR (500 MHz, CDCl3)
1.00
1.02
1.01
3.01
1.01
1.01
2.06
1.95
1.94
2.96
2.99
6.02
5.98

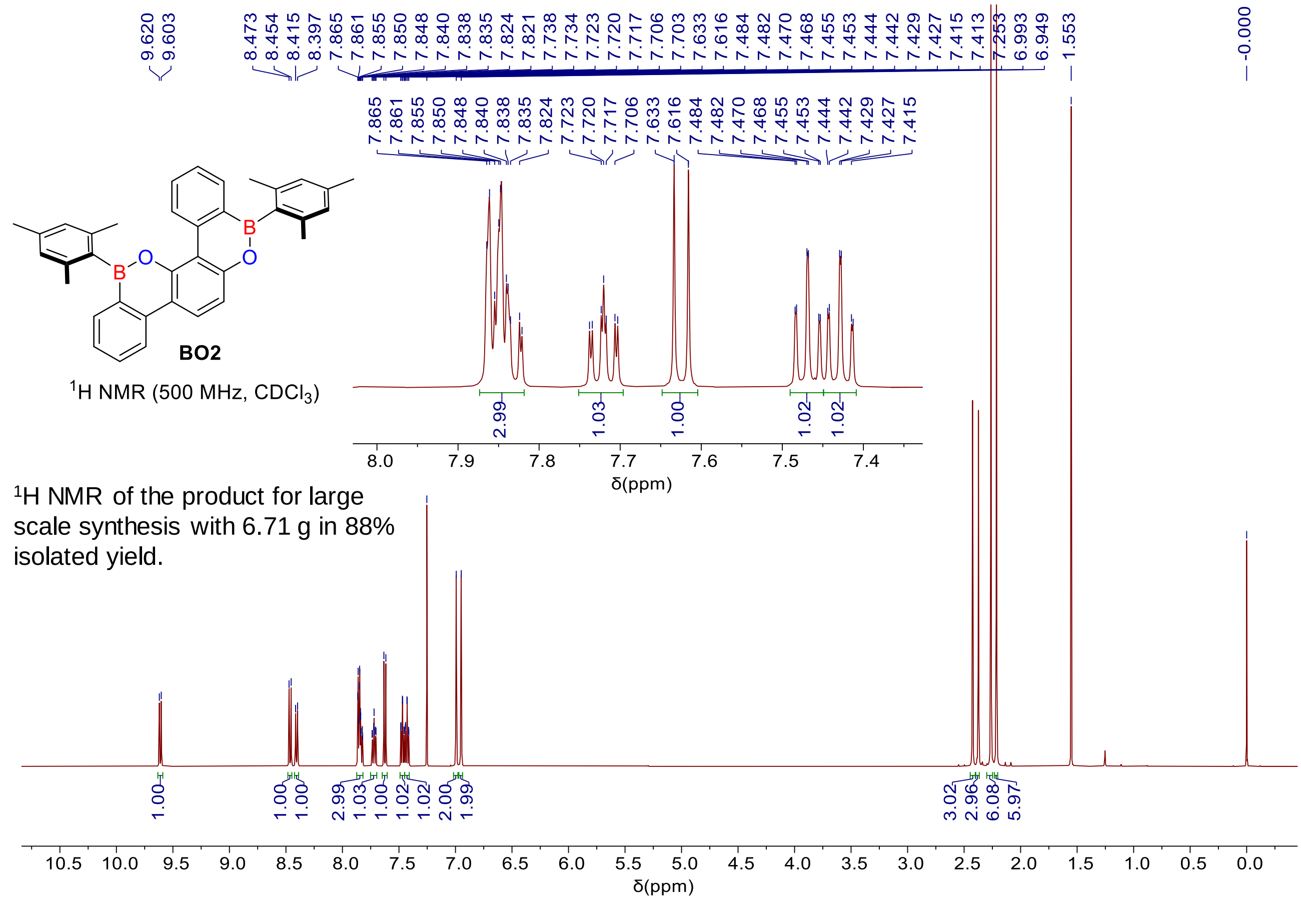
9.620
9.603
8.473
8.454
8.415
8.397
7.865 7.861 7.855 7.850 7.848 7.840 7.838 7.835 7.824 7.821 7.738 7.734 7.723 7.723 7.720 7.717 7.706 7.703 7.633 7.616 7.484 7.482 7.470 7.468 7.455 7.453 7.444 7.442 7.429 7.427 7.415 7.413 7.253
6.993
6.949
1.553
-0.000
BO2
^{1}H NMR (500 MHz, CDCl$_3$)
^{1}H NMR of the product for large scale synthesis with 6.71 g in 88% isolated yield.
δ(ppm)

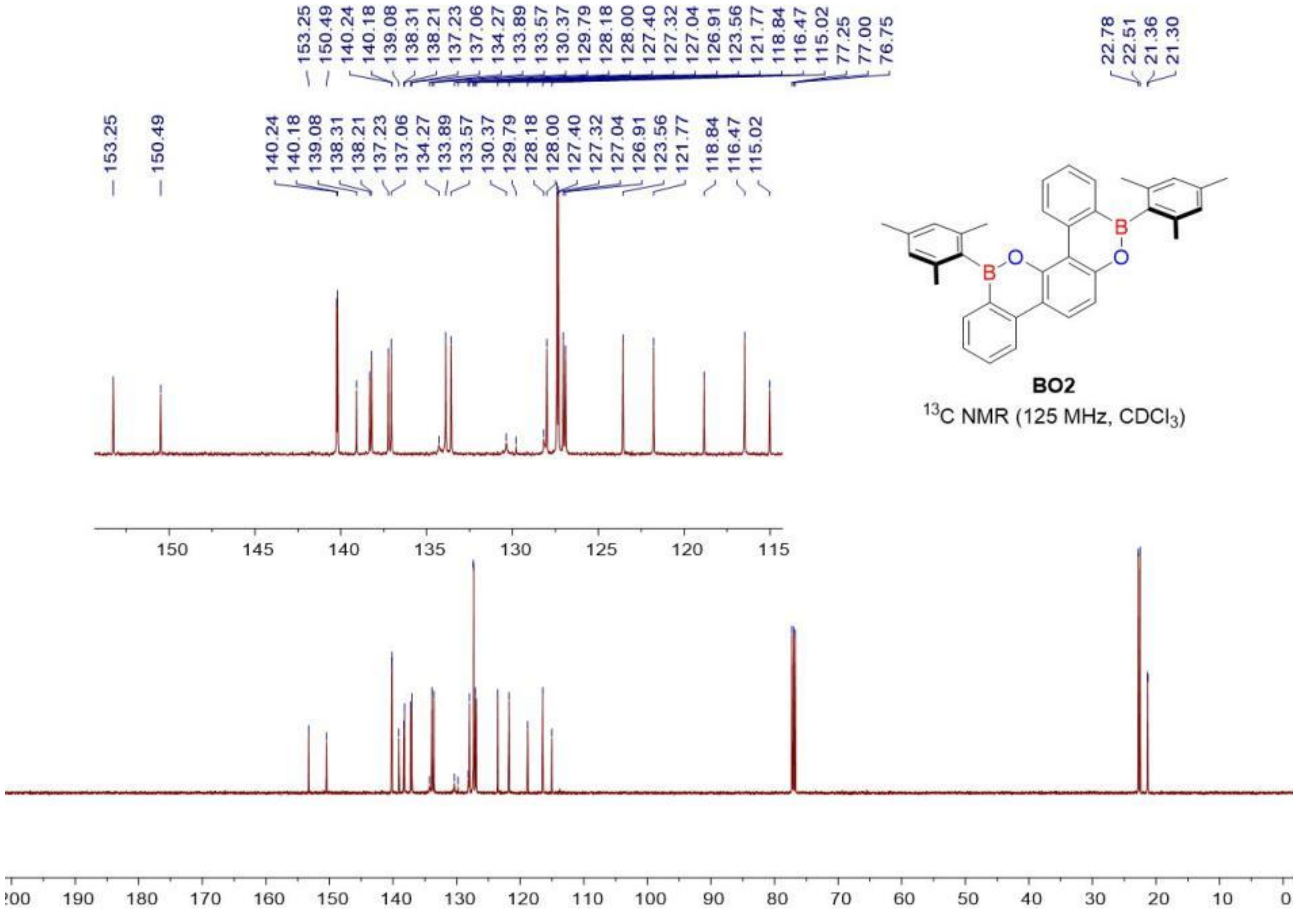
153.25 150.49 140.24 140.18 139.08 138.31 138.21 137.23 137.06 134.27 133.89 133.57 130.37 129.79 128.18 128.00 127.40 127.32 127.04 126.91 123.56 121.77 118.84 116.47 115.02 77.25 77.00 76.75
22.78 22.51 21.36 21.30
BO2
^{13}C NMR (125 MHz, CDCl$_3$)

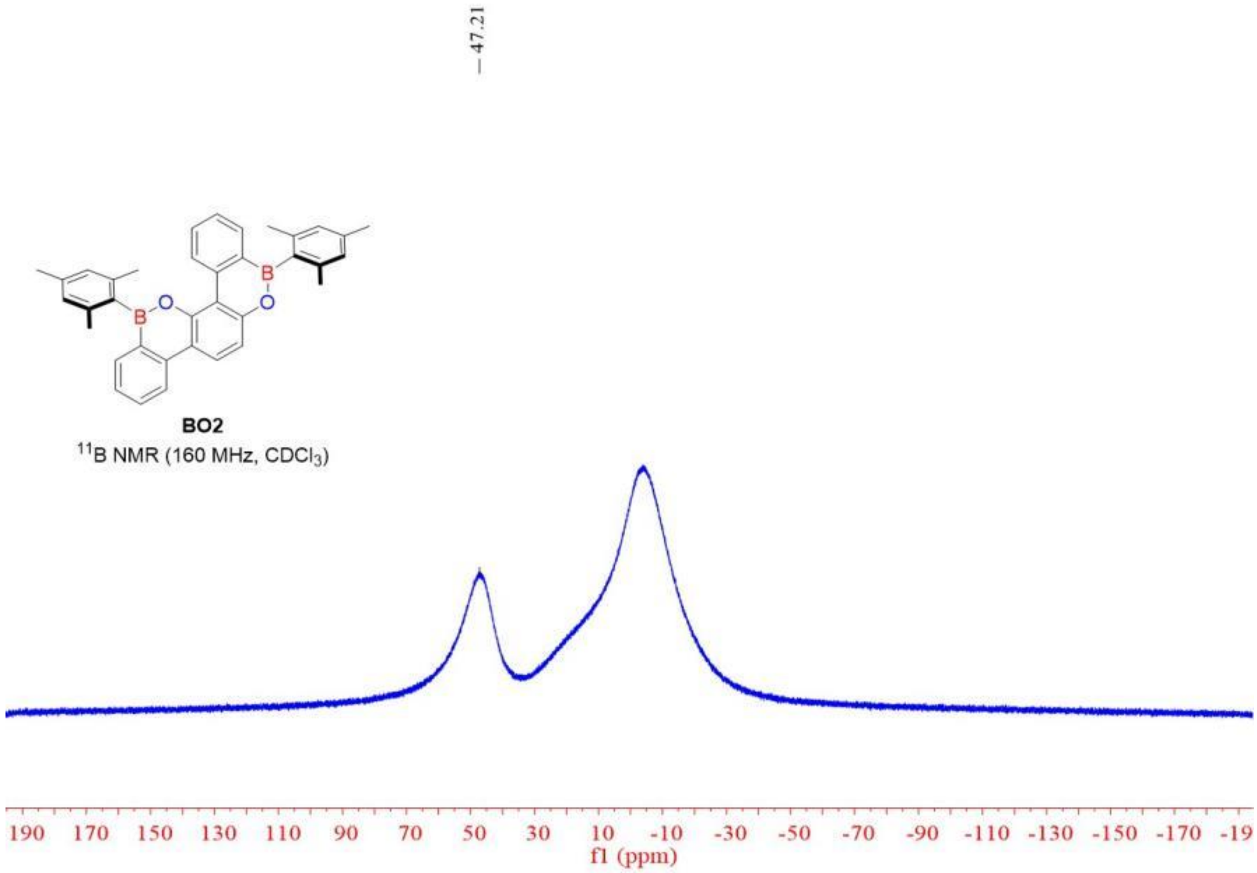

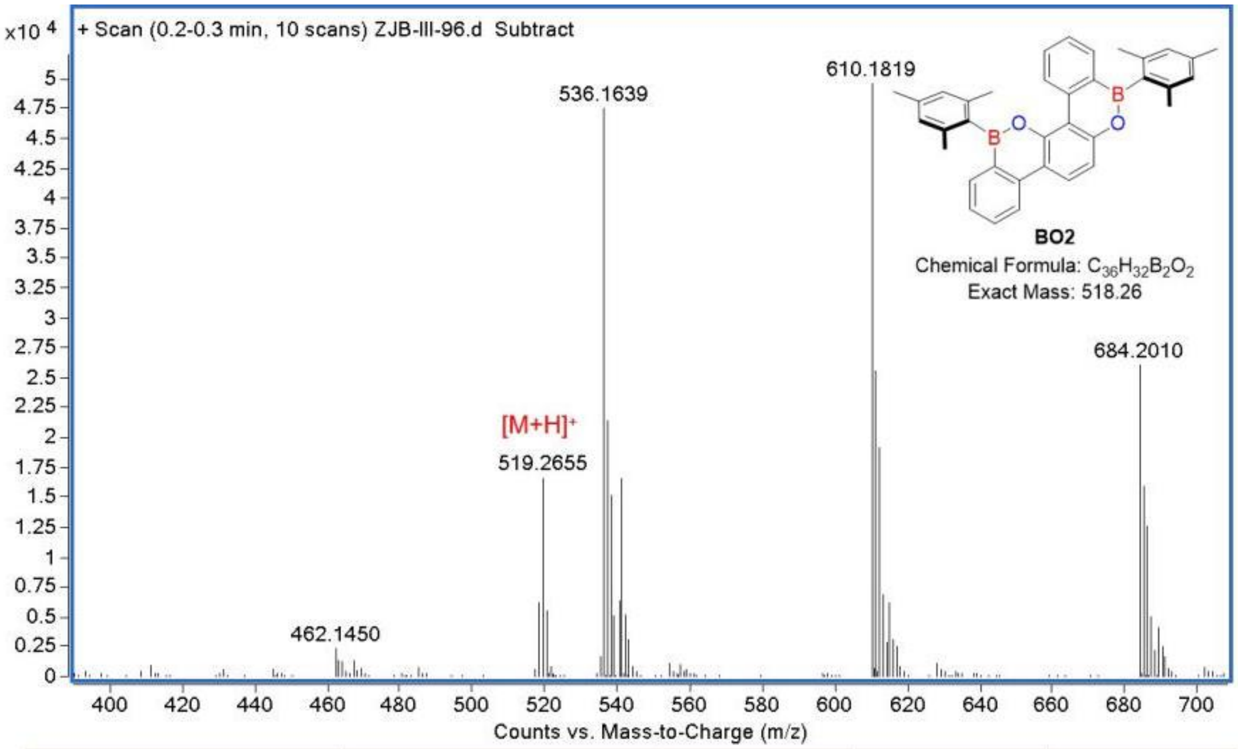

Formula (M)	Ion Formula	m/z	Calc m/z	Diff (ppm)
$C_{36}H_{32}[^{11}B]_2O_2$	$C_{36}H_{33}[^{11}B]_2O_2$	519.2655	519.2661	1.19

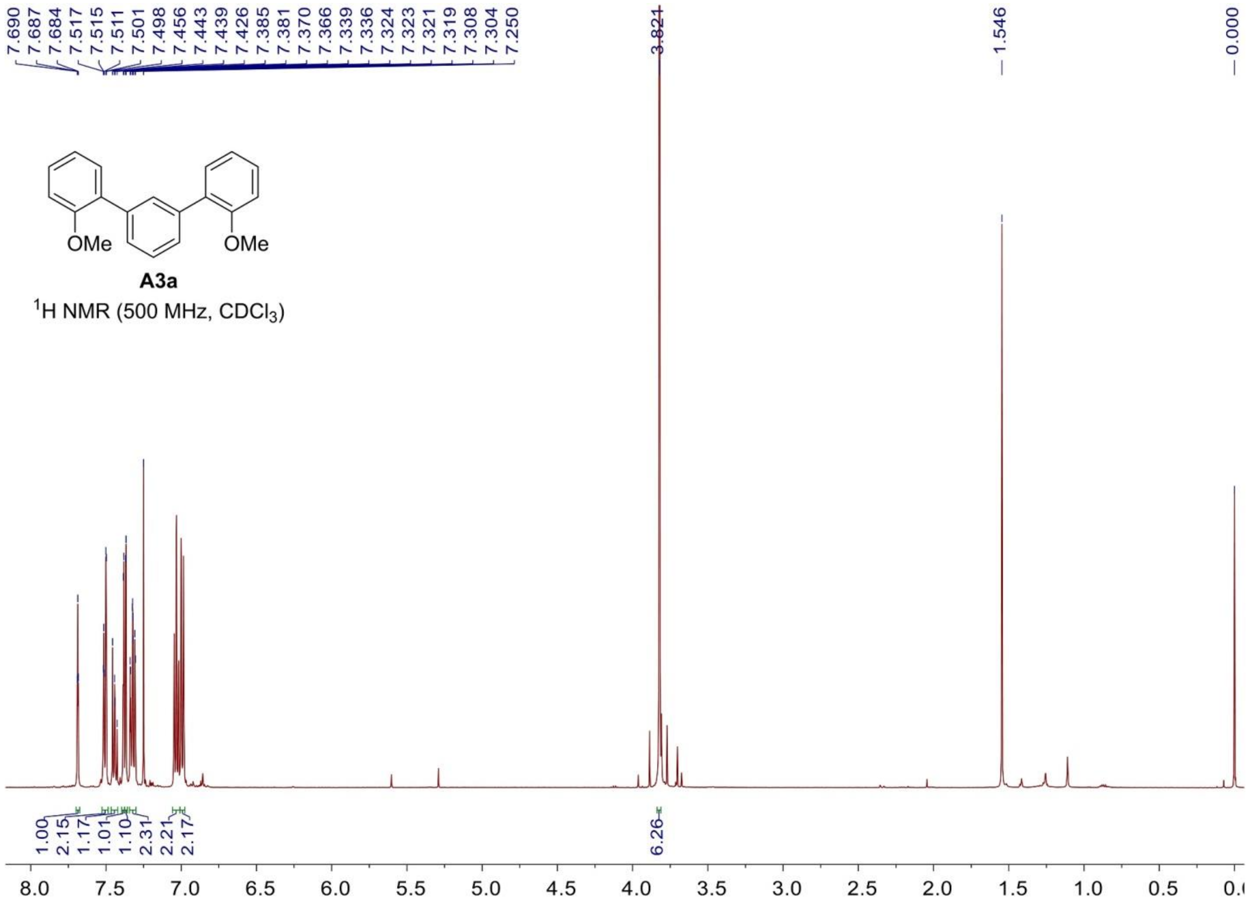
7.690
7.687
7.684
7.517
7.515
7.511
7.501
7.498
7.456
7.443
7.439
7.426
7.385
7.381
7.370
7.366
7.339
7.336
7.324
7.323
7.321
7.319
7.308
7.304
7.250
3.821
1.546
0.000
OMe
OMe
A3a
1H NMR (500 MHz, CDCl3)
1.00
2.15
1.17
1.01
1.10
2.31
2.21
2.17
6.26
8.0 7.5 7.0 6.5 6.0 5.5 5.0 4.5 4.0 3.5 3.0 2.5 2.0 1.5 1.0 0.5

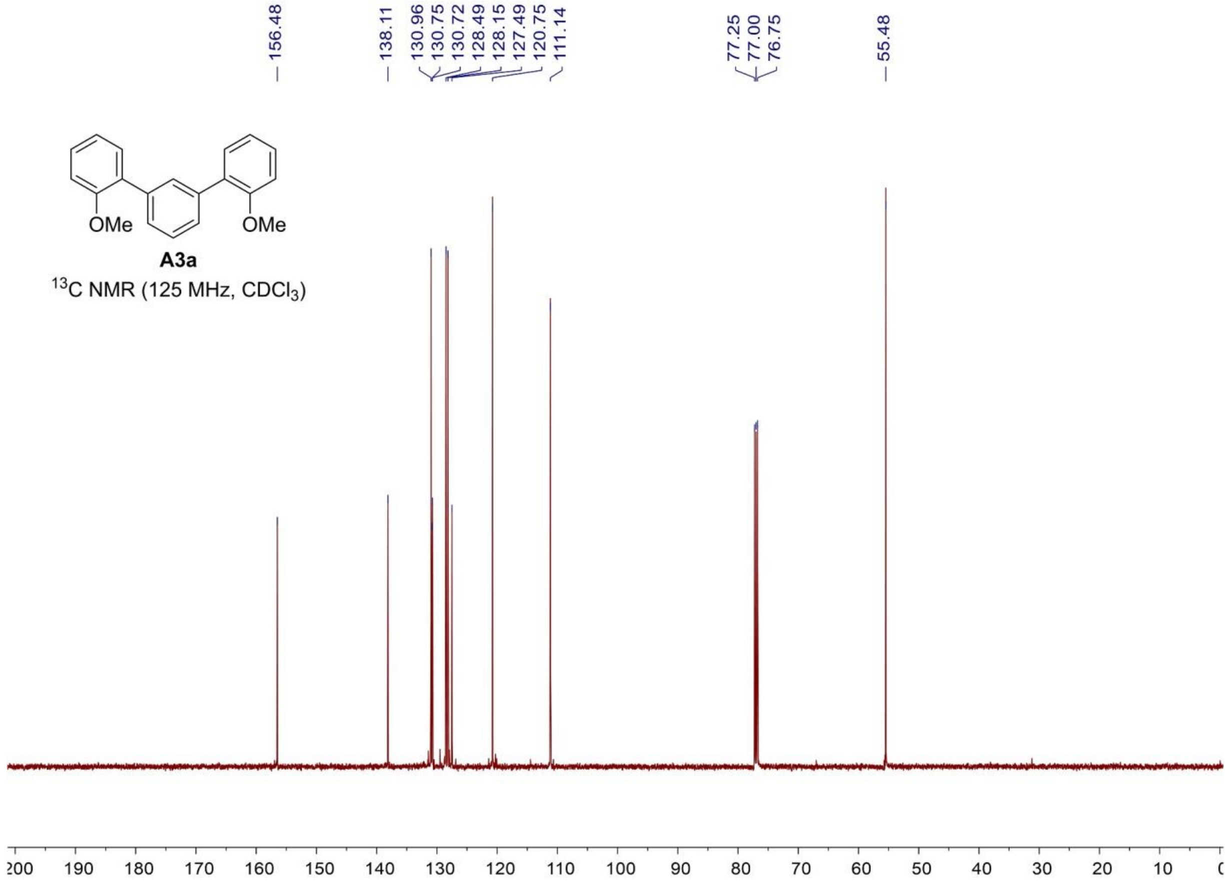
156.48
138.11
130.96
130.75
130.72
128.49
128.15
127.49
120.75
111.14
77.25
77.00
76.75
55.48
OMe
OMe
A3a
13C NMR (125 MHz, CDCl3)
190 180 170 160 150 140 130 120 110 100 90 80 70 60 50 40 30 20 10

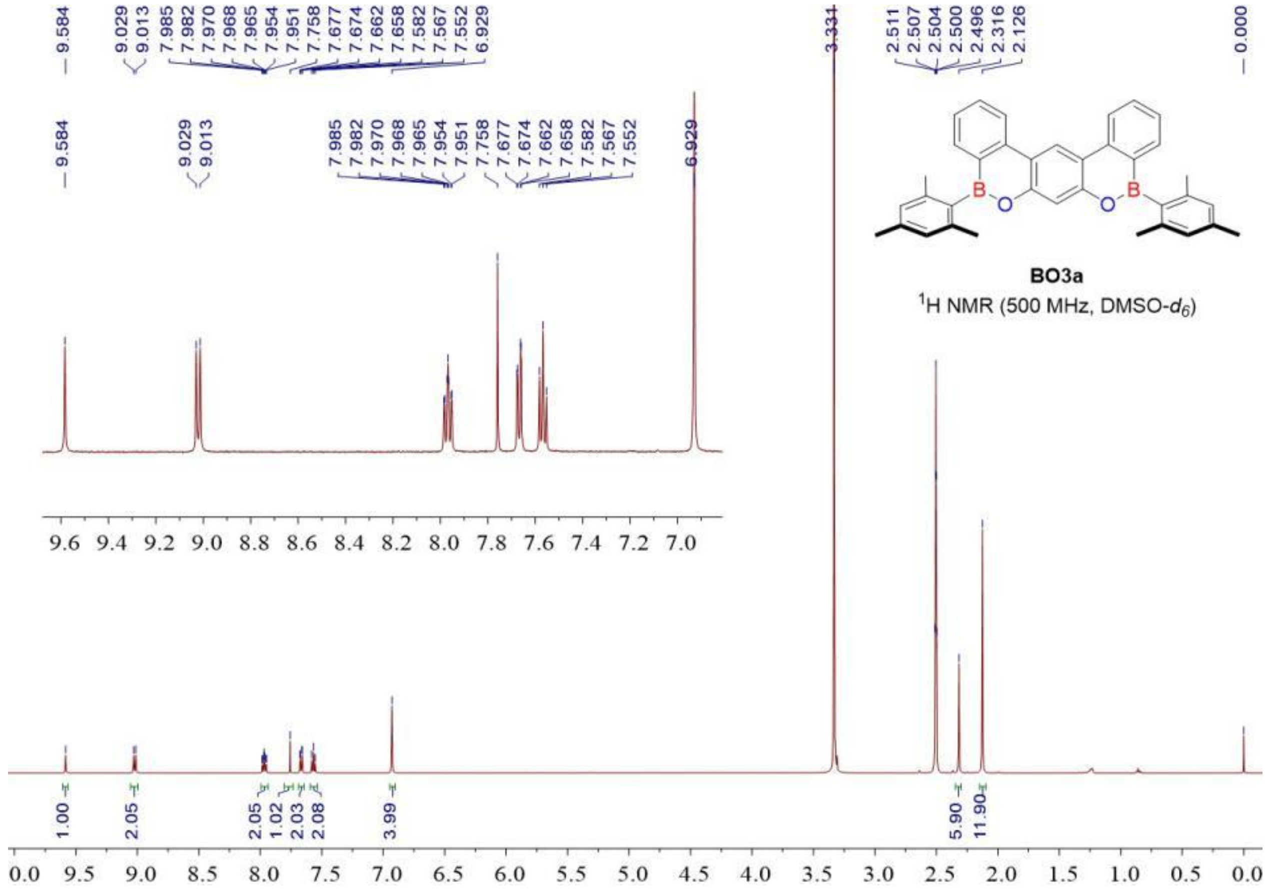
9.584
9.029
9.013
7.985
7.982
7.970
7.968
7.965
7.954
7.951
7.758
7.677
7.674
7.662
7.658
7.582
7.567
7.552
6.929
3.331
2.511
2.507
2.504
2.500
2.496
2.316
2.126
0.000
BO3a
¹H NMR (500 MHz, DMSO-d6)
1.00
2.05
2.05
1.02
2.03
2.08
3.99
5.90
11.90

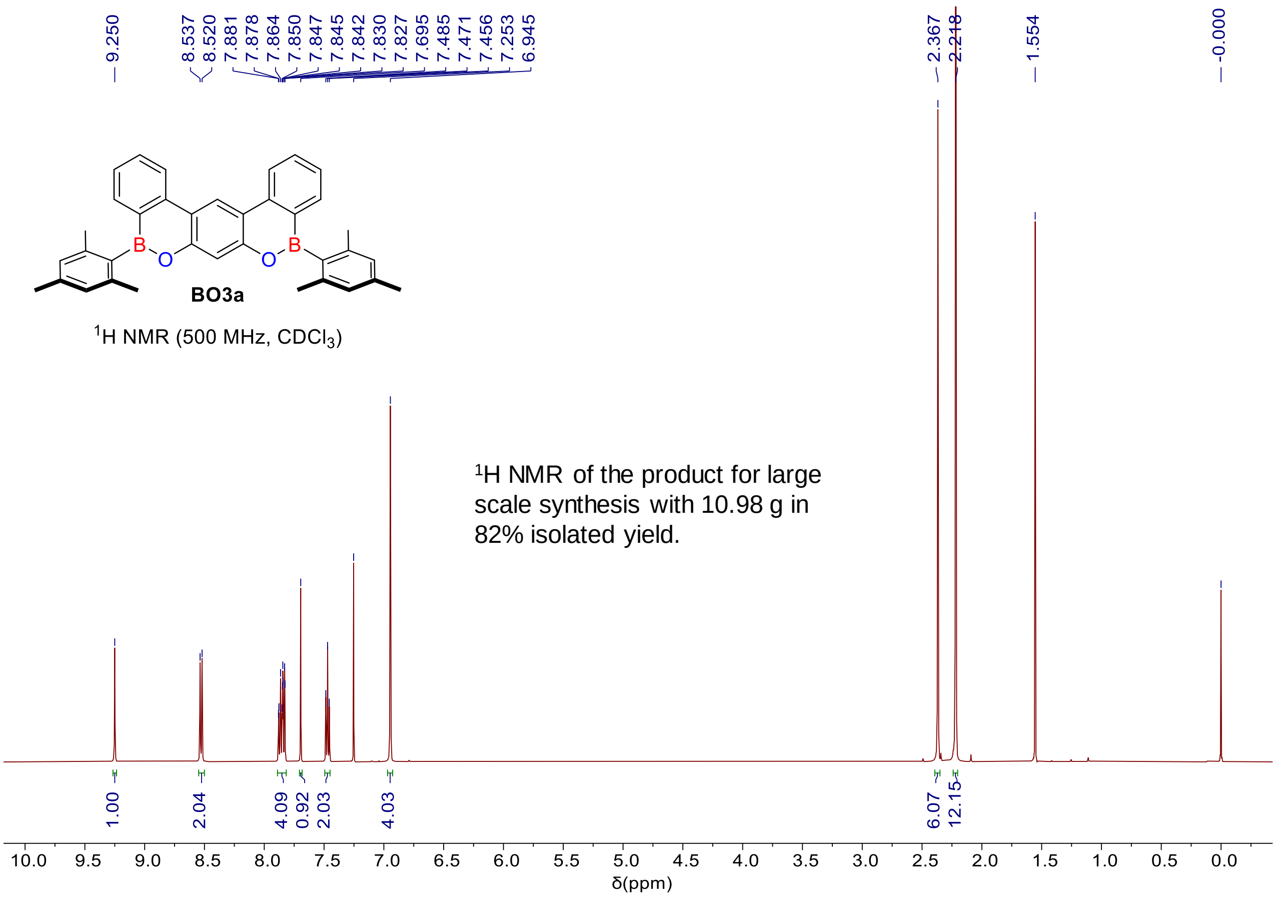
9.250
8.537
8.520
7.881
7.878
7.864
7.850
7.847
7.845
7.842
7.830
7.827
7.695
7.485
7.471
7.456
7.253
6.945
2.367
2.218
1.554
-0.000
BO3a
¹H NMR (500 MHz, CDCl3)
¹H NMR of the product for large scale synthesis with 10.98 g in 82% isolated yield.
1.00
2.04
4.09
0.92
2.03
4.03
6.07
12.15
δ(ppm)

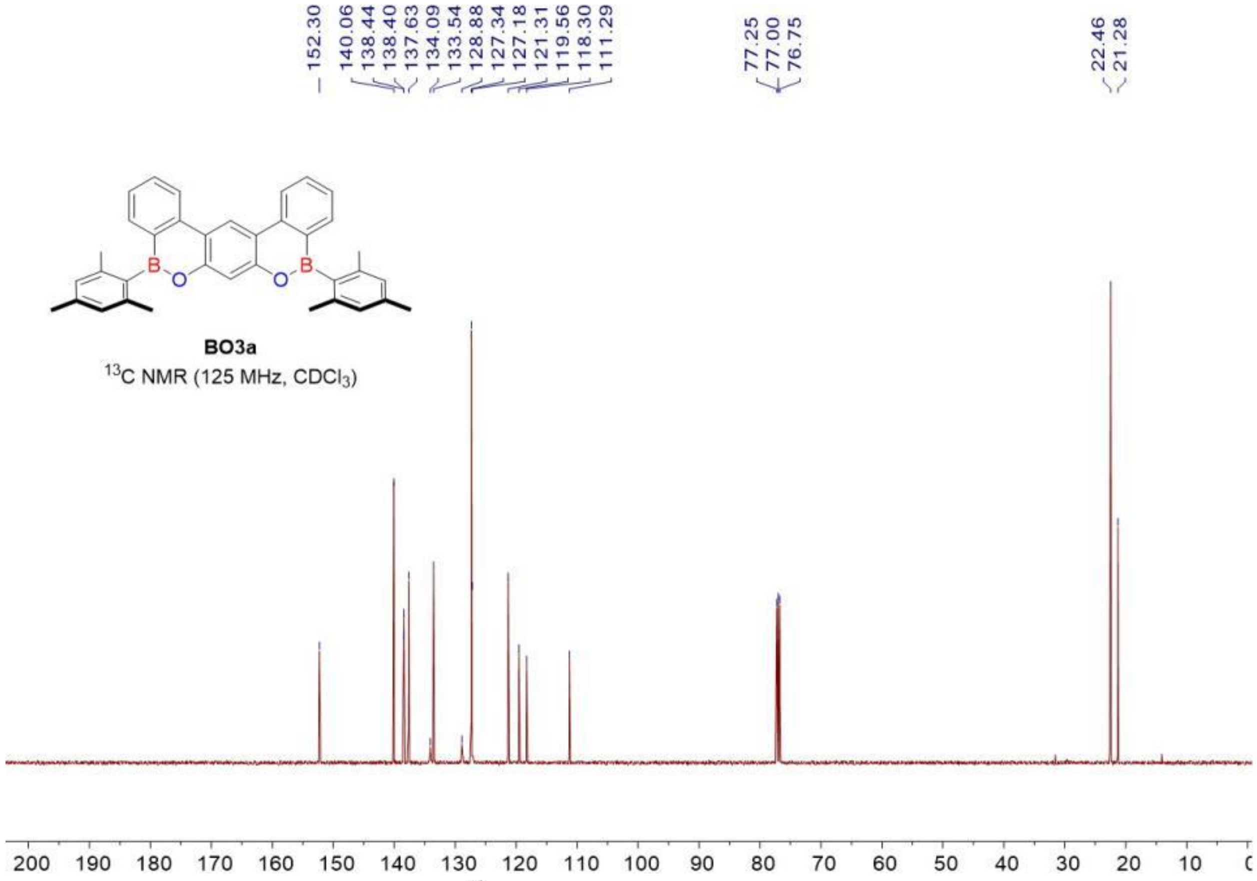
152.30
140.06
138.44
138.40
137.63
134.09
133.54
128.88
127.34
127.18
121.31
119.56
118.30
111.29
77.25
77.00
76.75
22.46
21.28
BO3a
^{13}C NMR (125 MHz, $CDCl_3$)
200 190 180 170 160 150 140 130 120 110 100 90 80 70 60 50 40 30 20 10

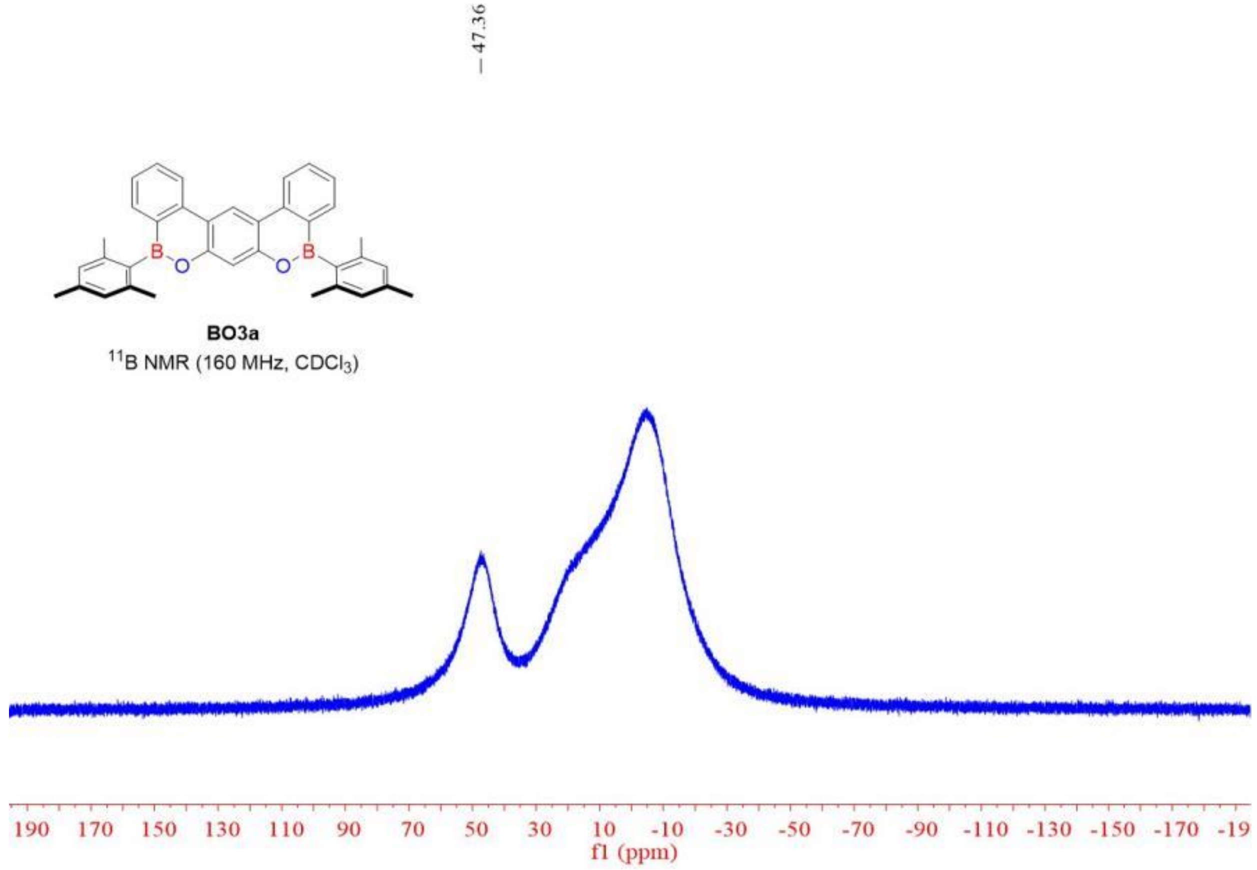
47.36
BO3a
^{11}B NMR (160 MHz, $CDCl_3$)
190 170 150 130 110 90 70 50 30 10 -10 -30 -50 -70 -90 -110 -130 -150 -170 -19
f1 (ppm)

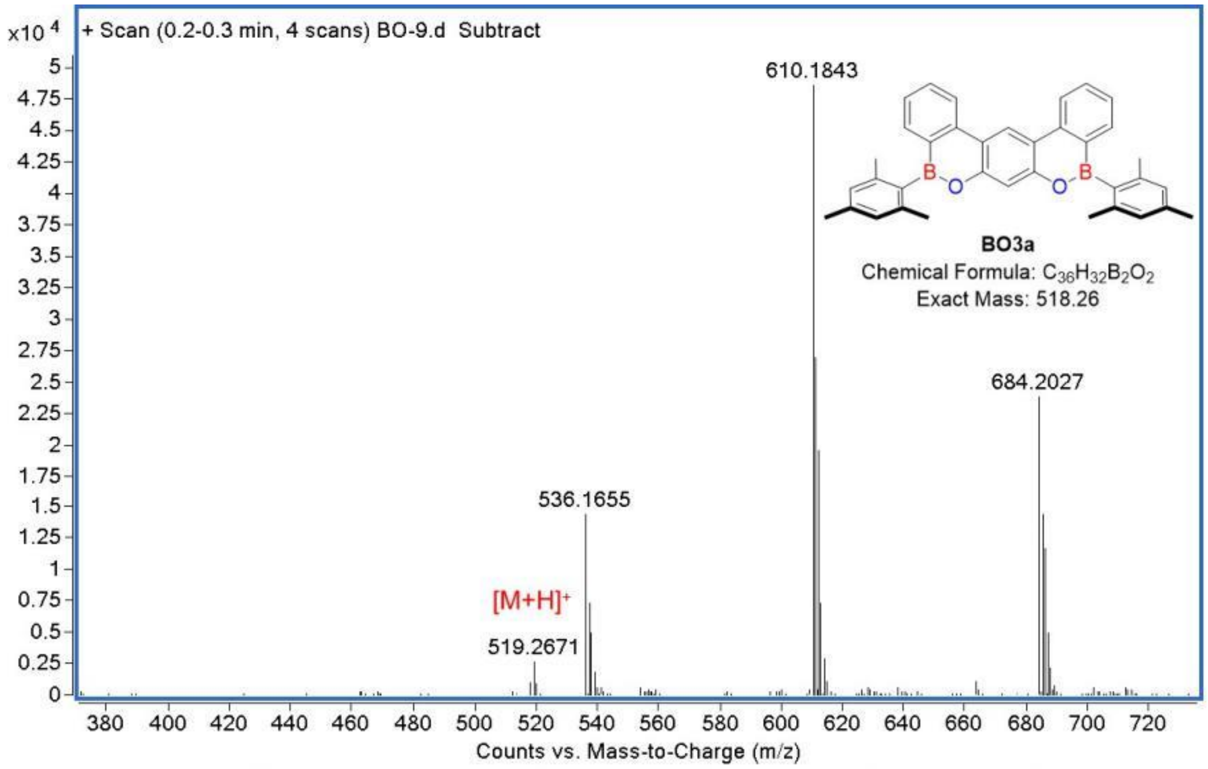

Formula (M)	Ion Formula	m/z	Calc m/z	Diff (ppm)	DBE
$C_{36}H_{32}[^{11}B]_2O_2$	$C_{36}H_{33}[^{11}B]_2O_2$	519.2671	519.2661	-1.9	22

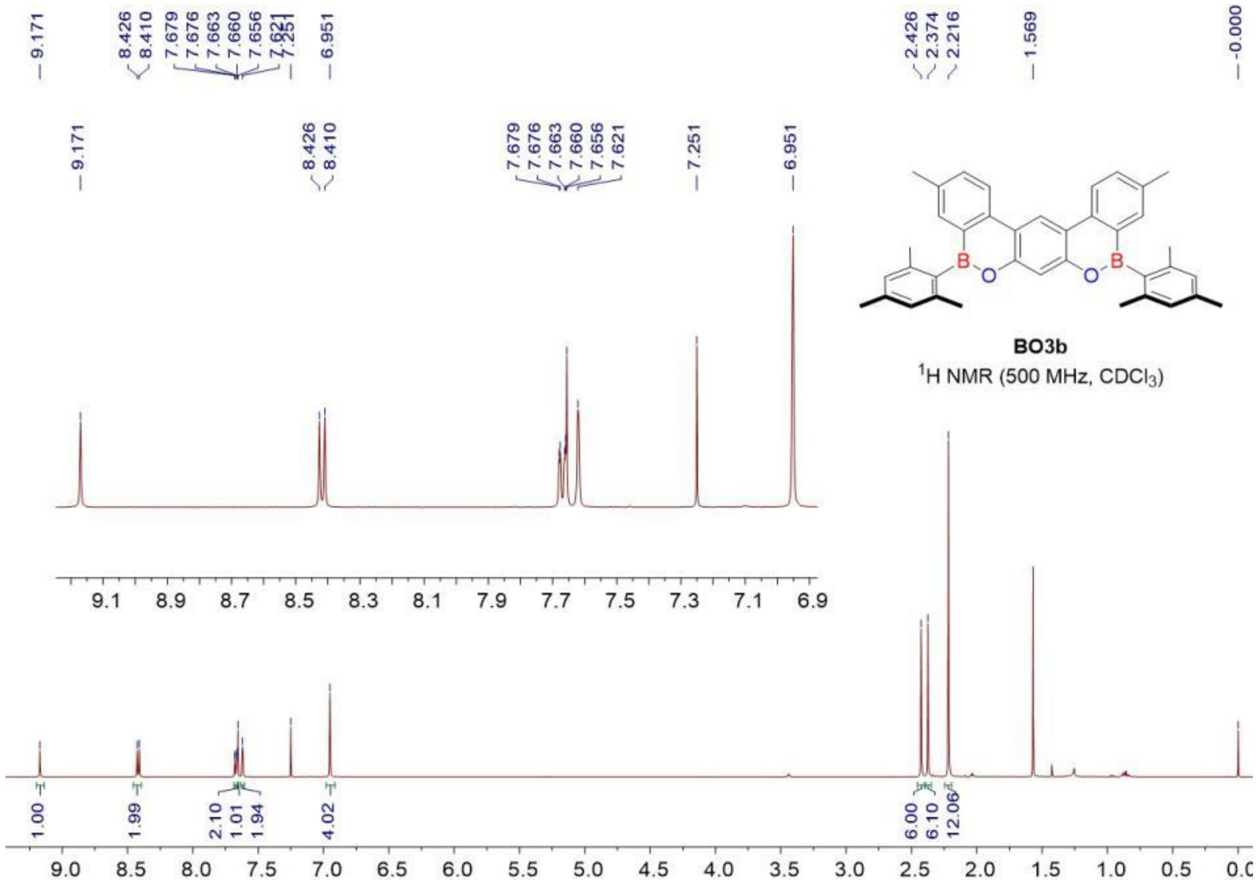

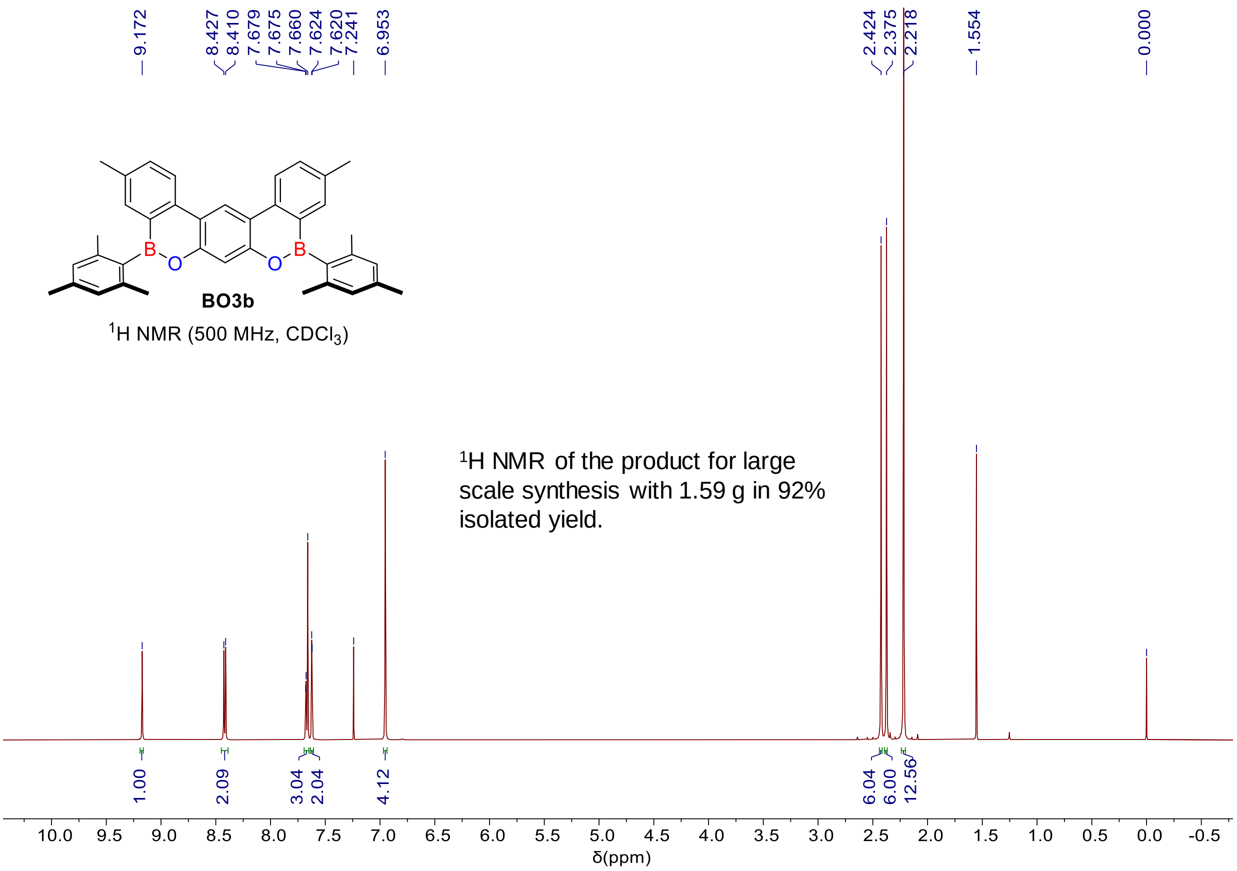
9.172
8.427
8.410
7.679
7.675
7.660
7.624
7.620
7.241
6.953
2.424
2.375
2.218
1.554
0.000
BO3b
^{1}H NMR (500 MHz, CDCl$_3$)
^{1}H NMR of the product for large scale synthesis with 1.59 g in 92% isolated yield.
1.00
2.09
3.04
2.04
4.12
6.04
6.00
12.56
10.0 9.5 9.0 8.5 8.0 7.5 7.0 6.5 6.0 5.5 5.0 4.5 4.0 3.5 3.0 2.5 2.0 1.5 1.0 0.5 0.0 -0.5
δ(ppm)

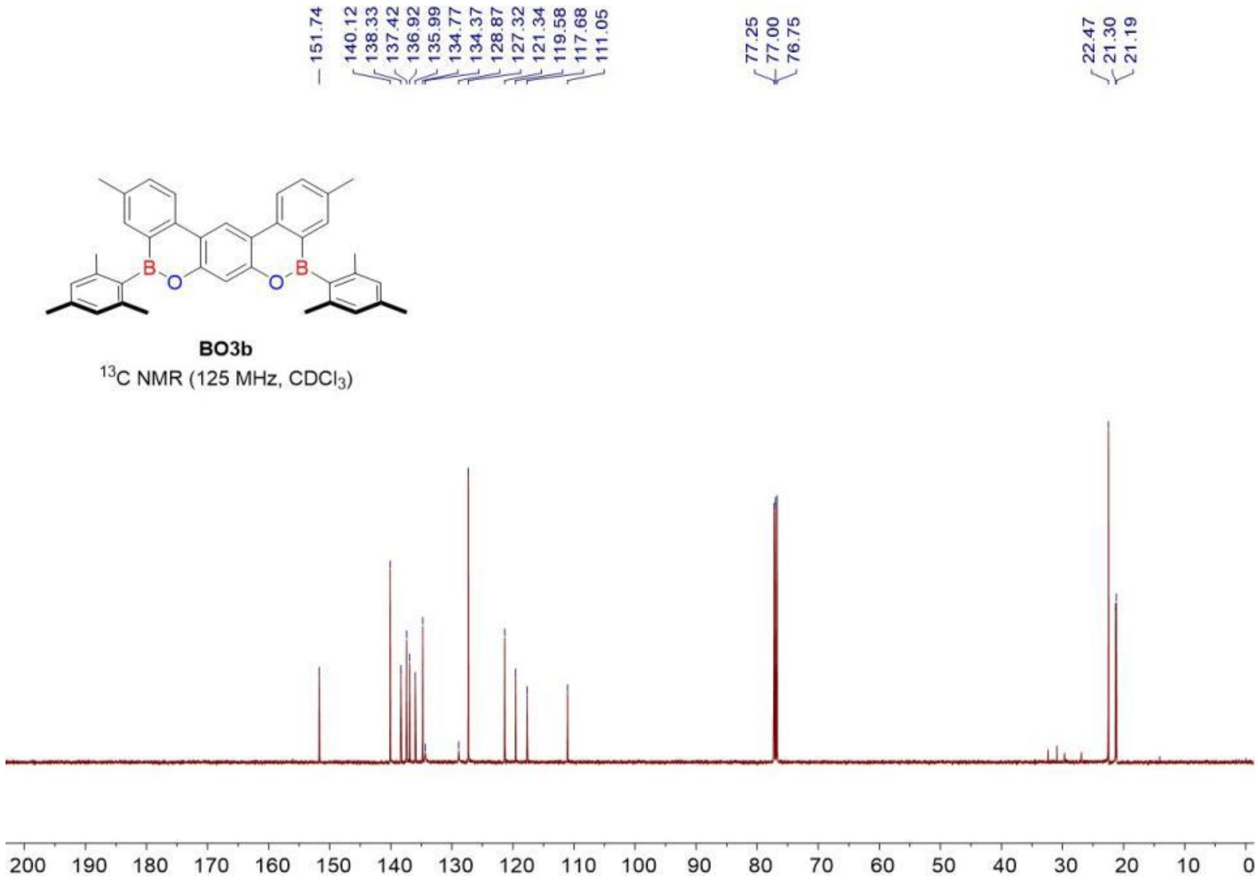
151.74
140.12
138.33
137.42
136.92
135.99
134.77
134.37
128.87
127.32
121.34
119.58
117.68
111.05
77.25
77.00
76.75
22.47
21.30
21.19
BO3b
^{13}C NMR (125 MHz, CDCl$_3$)
200 190 180 170 160 150 140 130 120 110 100 90 80 70 60 50 40 30 20 10 0

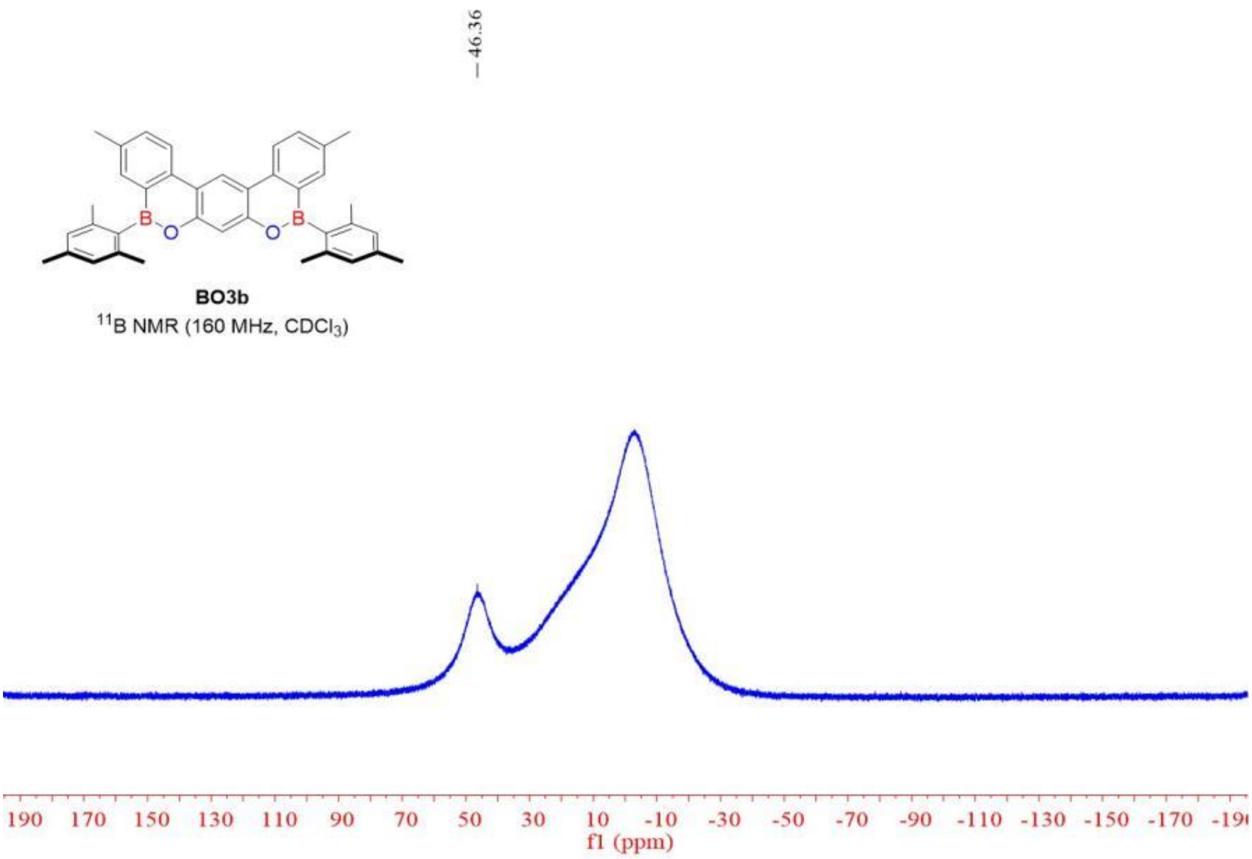

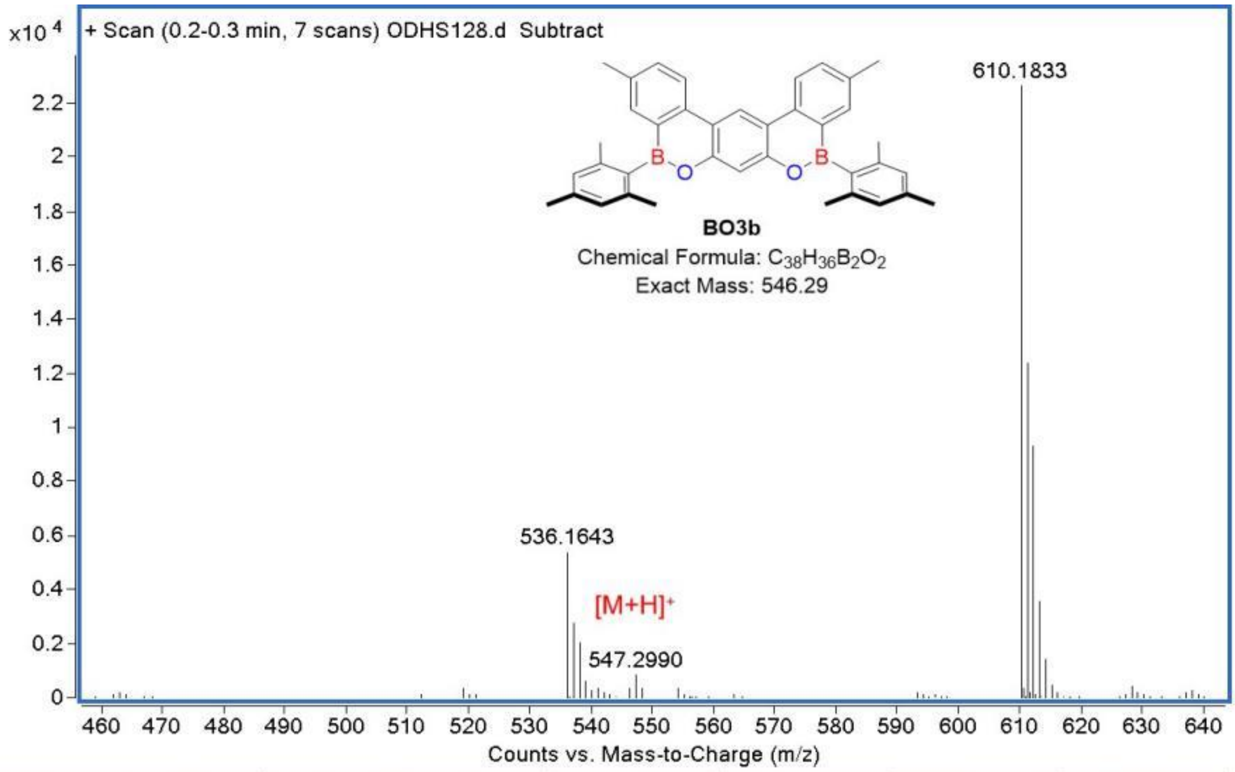

Formula (M)	Ion Formula	m/z	Calc m/z	Diff (ppm)	DBE
$C_{38}H_{36}[^{11}B]_2O_2$	$C_{38}H_{37}[^{11}B]_2O_2$	547.299	547.2974	-2.9	22

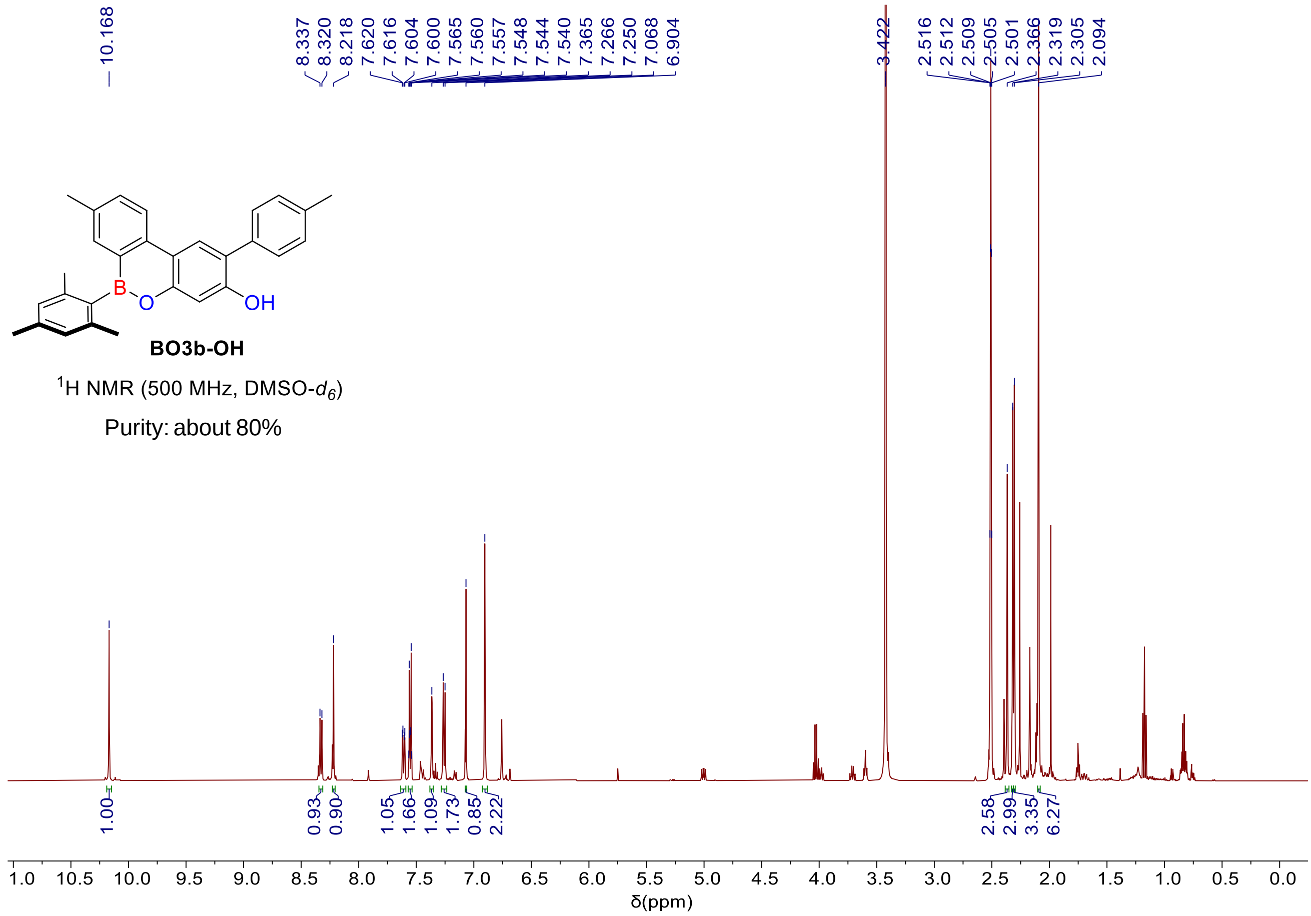

BO3b-OH
^{1}H NMR (500 MHz, DMSO-d_6)
Purity: about 80%

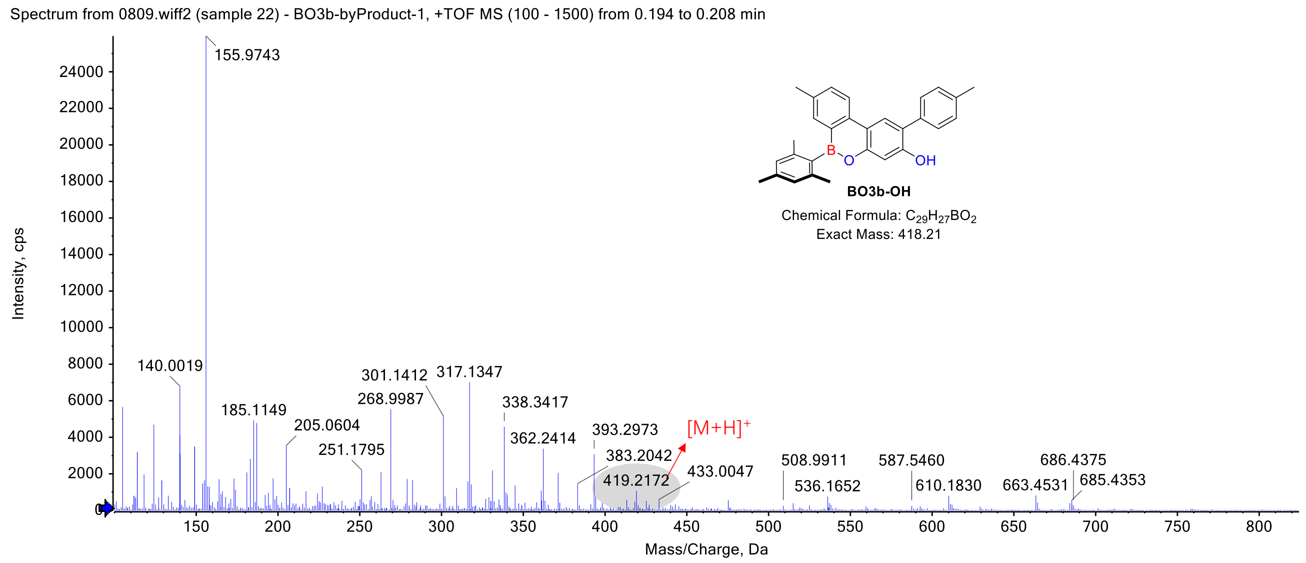

Spectrum from 0809.wiff2 (sample 22) - BO3b-byProduct-1, +TOF MS (100 - 1500) from 0.194 to 0.208 min
BO3b-OH
Chemical Formula: C29H27BO2
Exact Mass: 418.21
[M+H]+

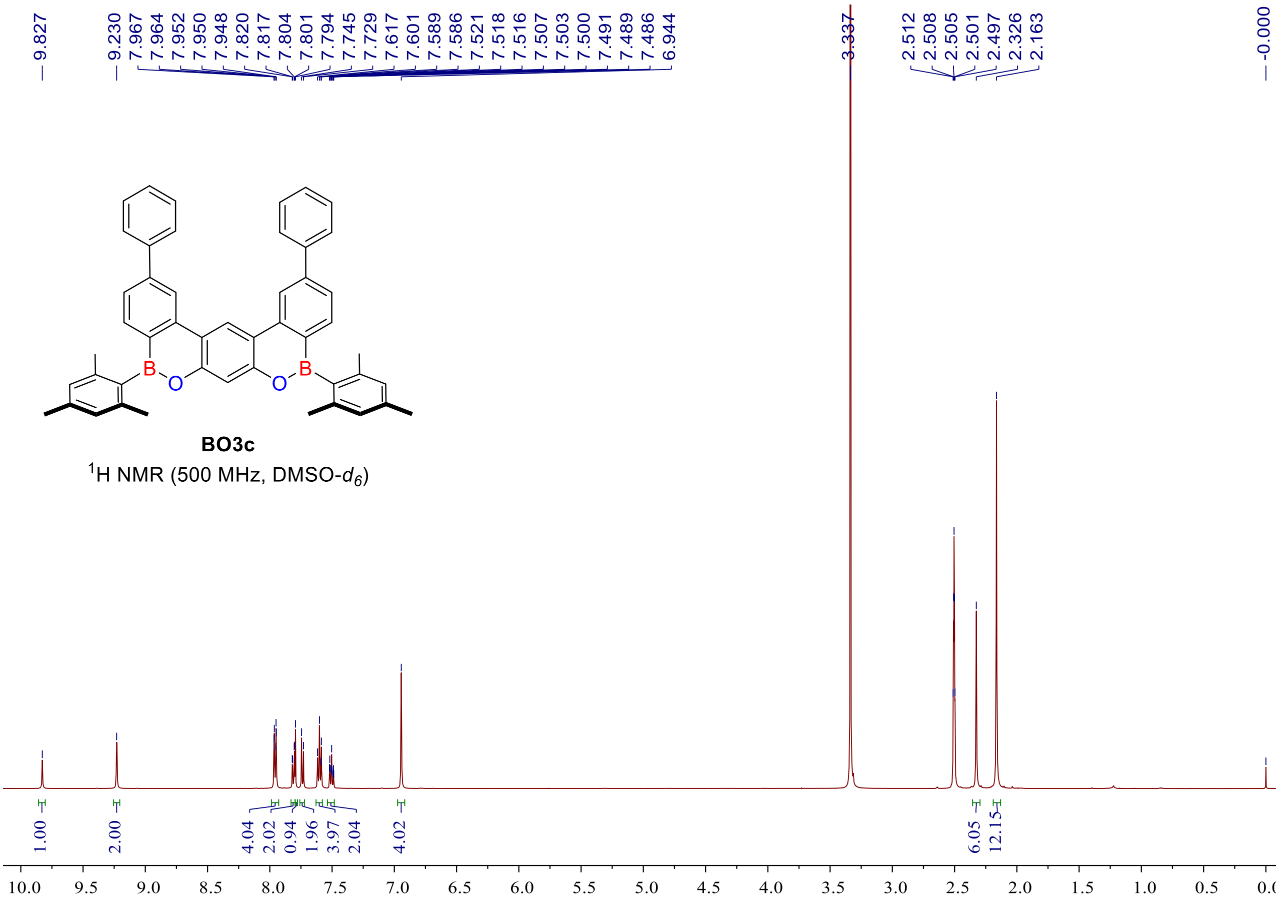
9.827
9.230
7.967
7.964
7.952
7.950
7.948
7.820
7.817
7.804
7.801
7.794
7.745
7.729
7.617
7.601
7.589
7.586
7.521
7.518
7.516
7.507
7.503
7.500
7.491
7.489
7.486
6.944
3.337
2.512
2.508
2.505
2.501
2.497
2.326
2.163
-0.000
B
O
O
B
BO3c
¹H NMR (500 MHz, DMSO-d6)
1.00
2.00
4.04
2.02
0.94
1.96
3.97
2.04
4.02
6.05
12.15
10.0 9.5 9.0 8.5 8.0 7.5 7.0 6.5 6.0 5.5 5.0 4.5 4.0 3.5 3.0 2.5 2.0 1.5 1.0 0.5 0.0

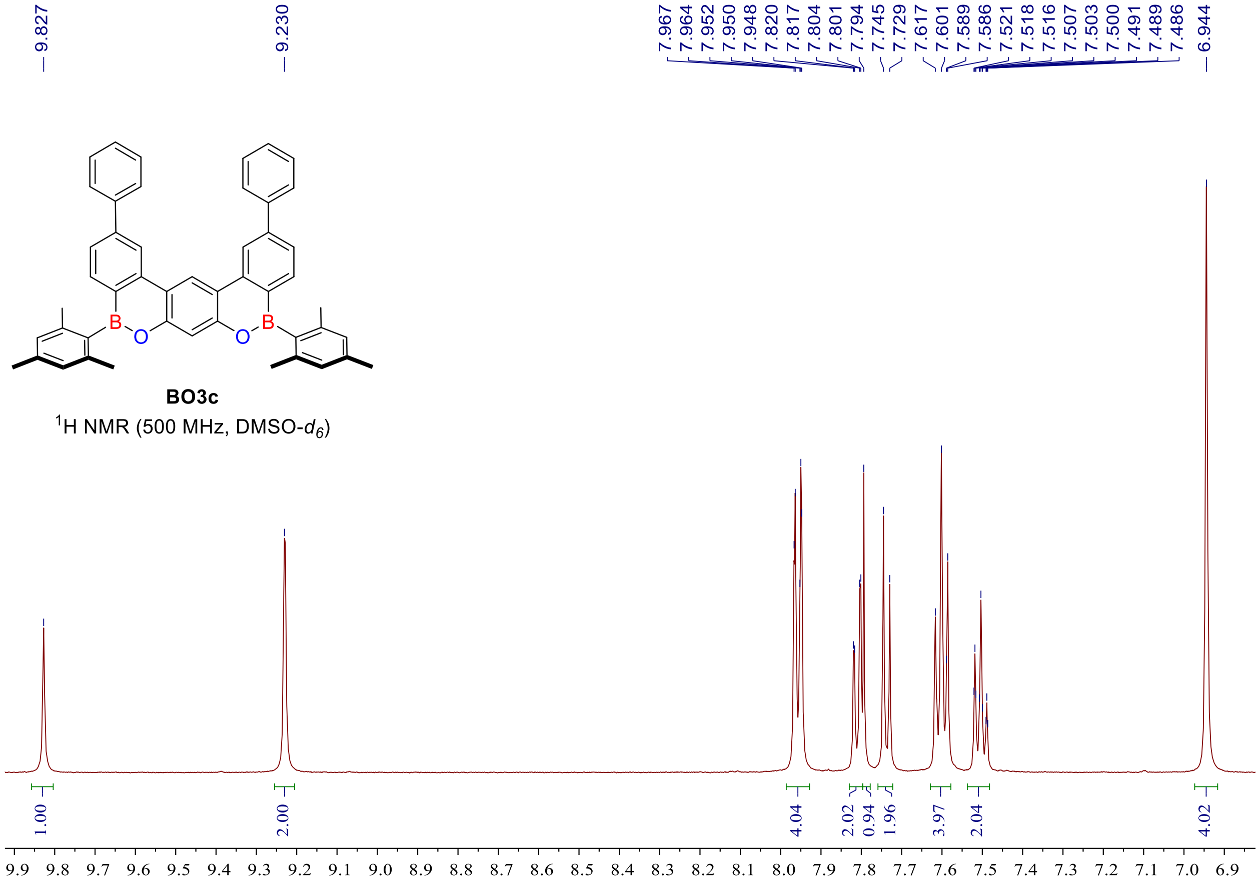
9.827
9.230
7.967
7.964
7.952
7.950
7.948
7.820
7.817
7.804
7.801
7.794
7.745
7.729
7.617
7.601
7.589
7.586
7.521
7.518
7.516
7.507
7.503
7.500
7.491
7.489
7.486
6.944
B
O
O
B
BO3c
¹H NMR (500 MHz, DMSO-d6)
1.00
2.00
4.04
2.02
0.94
1.96
3.97
2.04
4.02
9.9 9.8 9.7 9.6 9.5 9.4 9.3 9.2 9.1 9.0 8.9 8.8 8.7 8.6 8.5 8.4 8.3 8.2 8.1 8.0 7.9 7.8 7.7 7.6 7.5 7.4 7.3 7.2 7.1 7.0 6.9

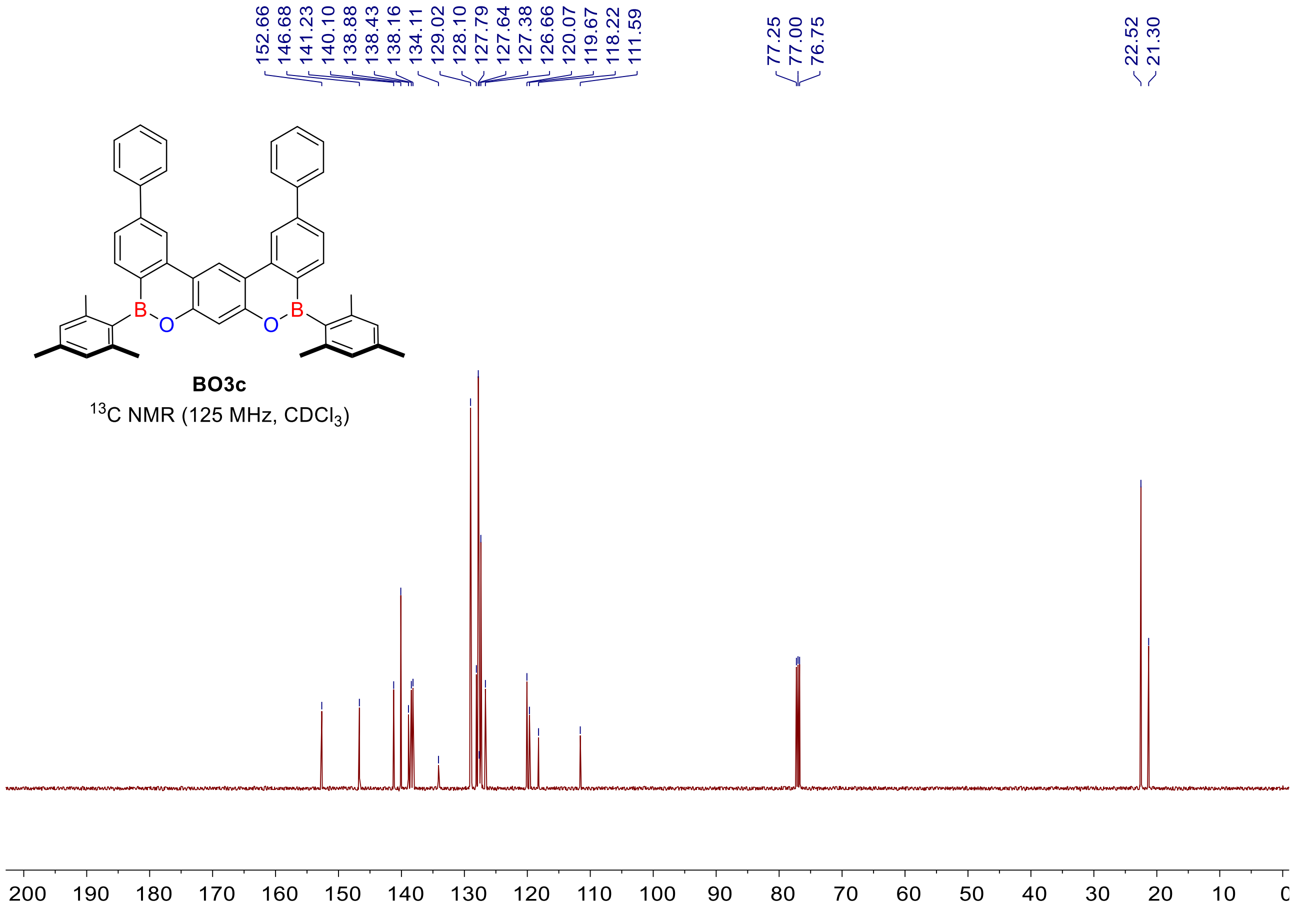
152.66
146.68
141.23
140.10
138.88
138.43
138.16
134.11
129.02
128.10
127.79
127.64
127.38
126.66
120.07
119.67
118.22
111.59
77.25
77.00
76.75
22.52
21.30
B
O
O
B
BO3c
13C NMR (125 MHz, CDCl3)
200 190 180 170 160 150 140 130 120 110 100 90 80 70 60 50 40 30 20 10 0

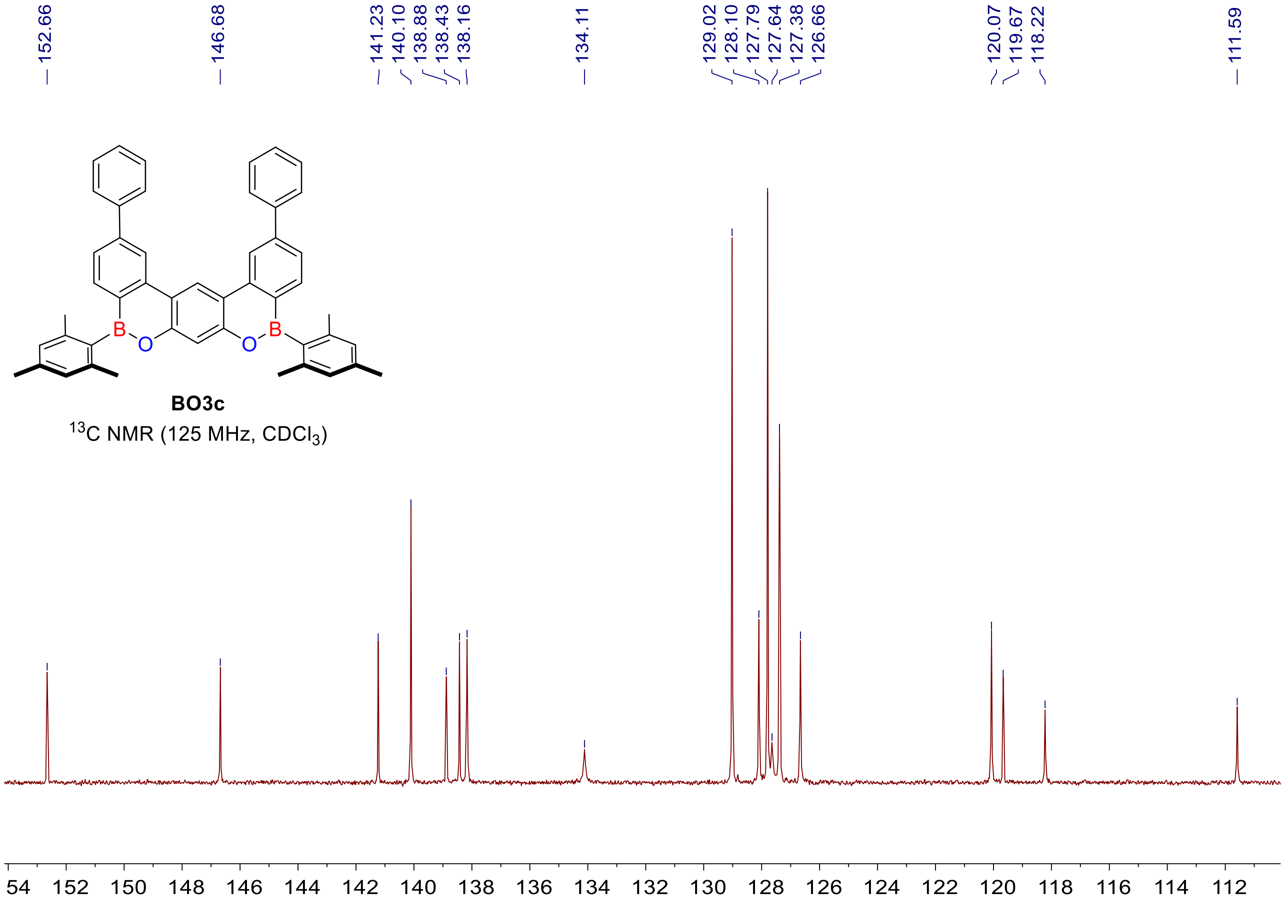
152.66
146.68
141.23
140.10
138.88
138.43
138.16
134.11
129.02
128.10
127.79
127.64
127.38
126.66
120.07
119.67
118.22
111.59
B
O
O
B
BO3c
13C NMR (125 MHz, CDCl3)
54 152 150 148 146 144 142 140 138 136 134 132 130 128 126 124 122 120 118 116 114 112

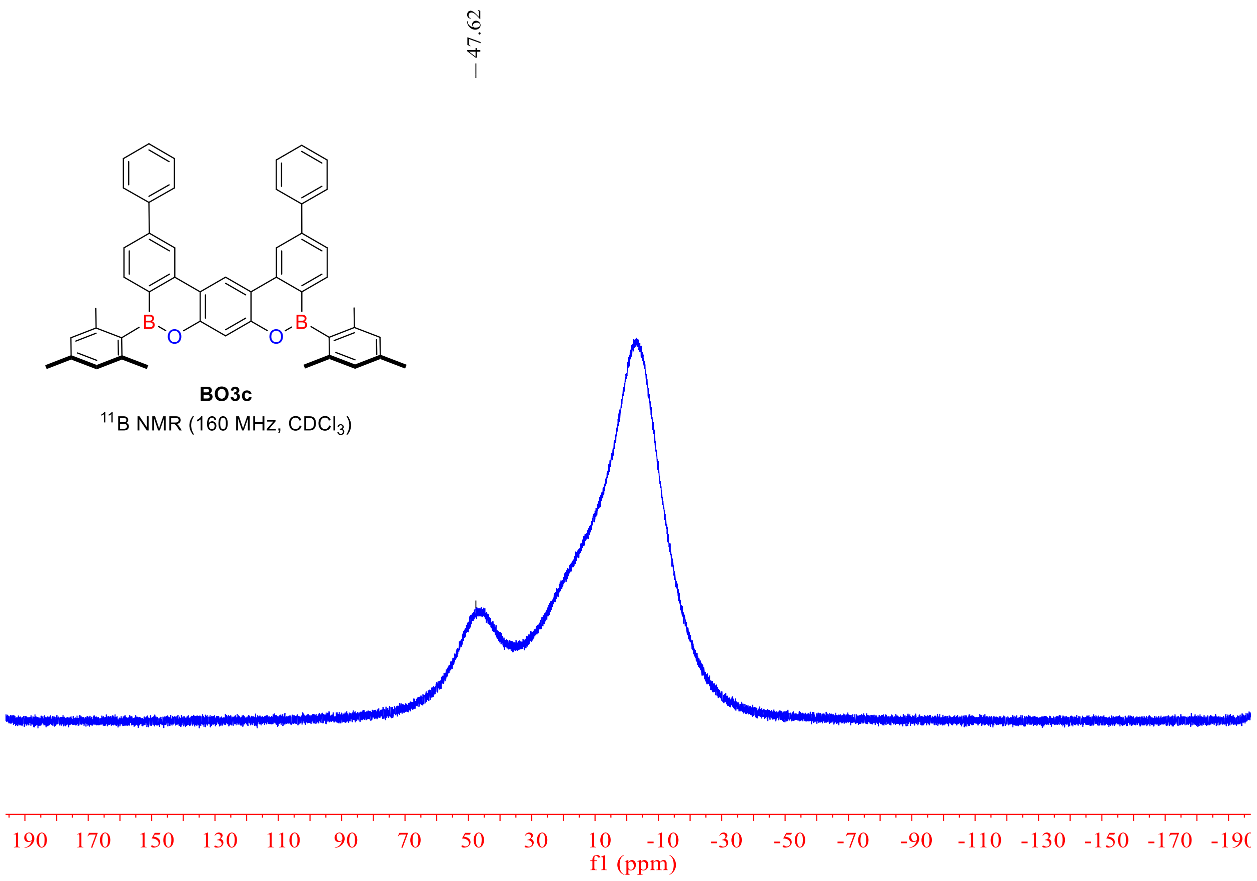

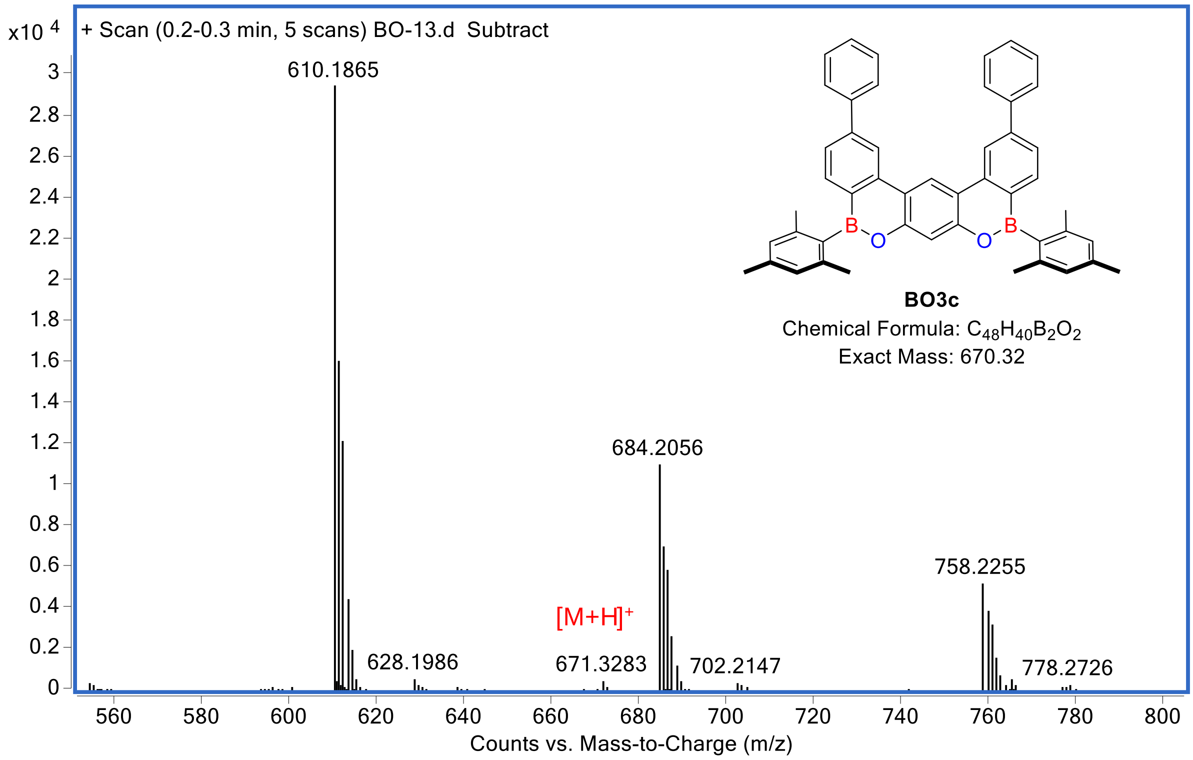

Formula (M)	Ion Formula	m/z	Calc m/z	Diff (ppm)	DBE
$C_{48}H_{40}[^{11}B]_2O_2$	$C_{48}H_{41}[^{11}B]_2O_2$	671.3283	671.3287	0.62	30

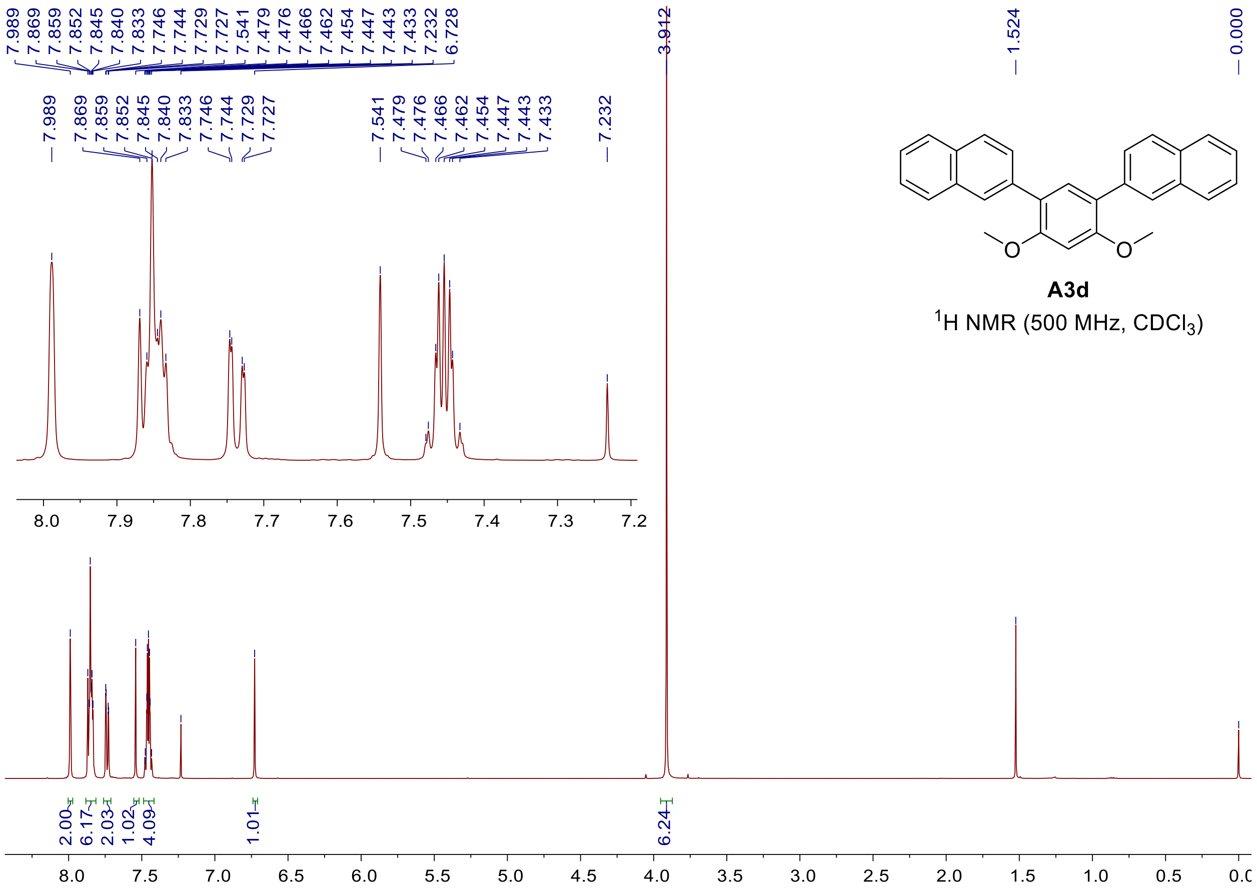
A3d
^{1}H NMR (500 MHz, CDCl$_3$)

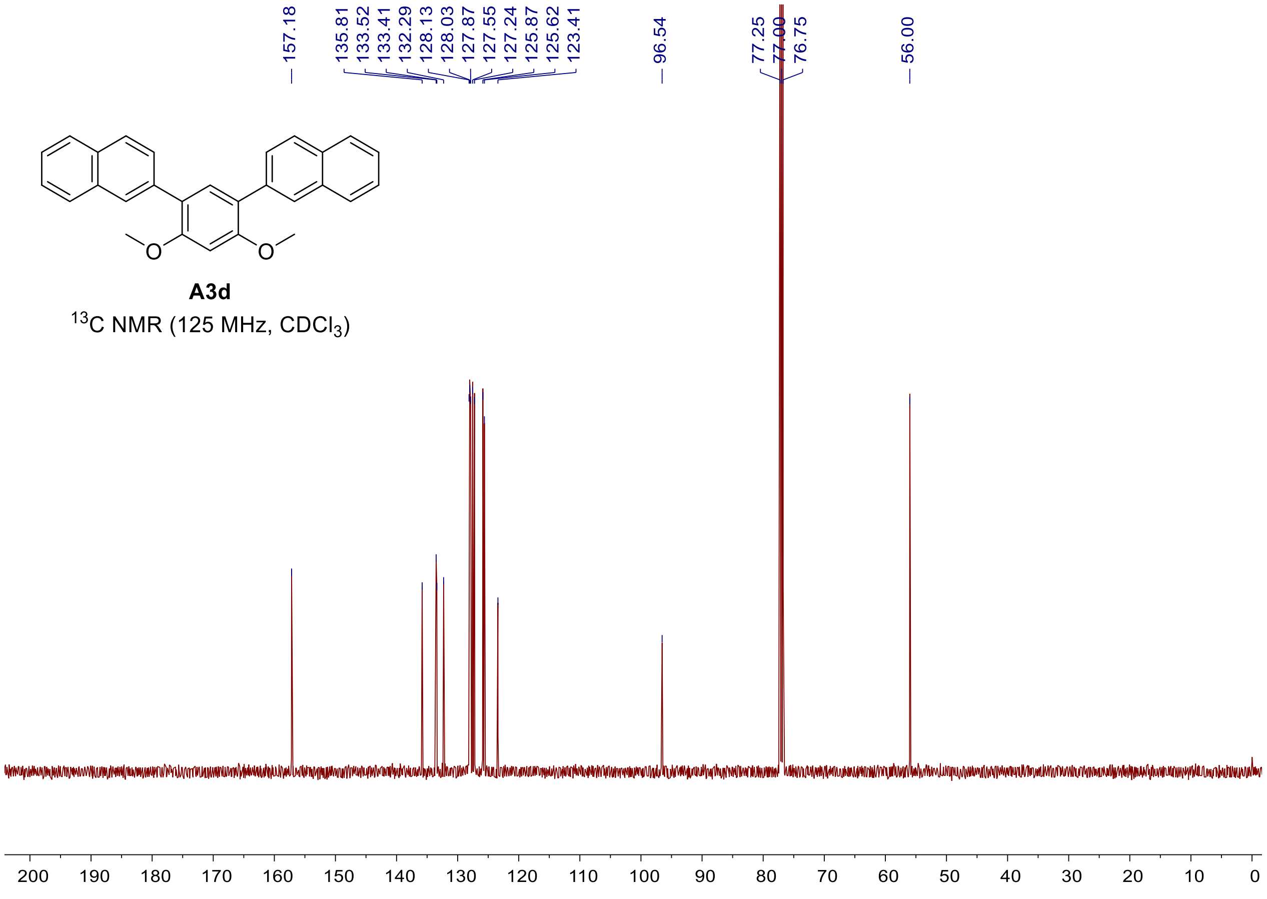
A3d
^{13}C NMR (125 MHz, CDCl$_3$)

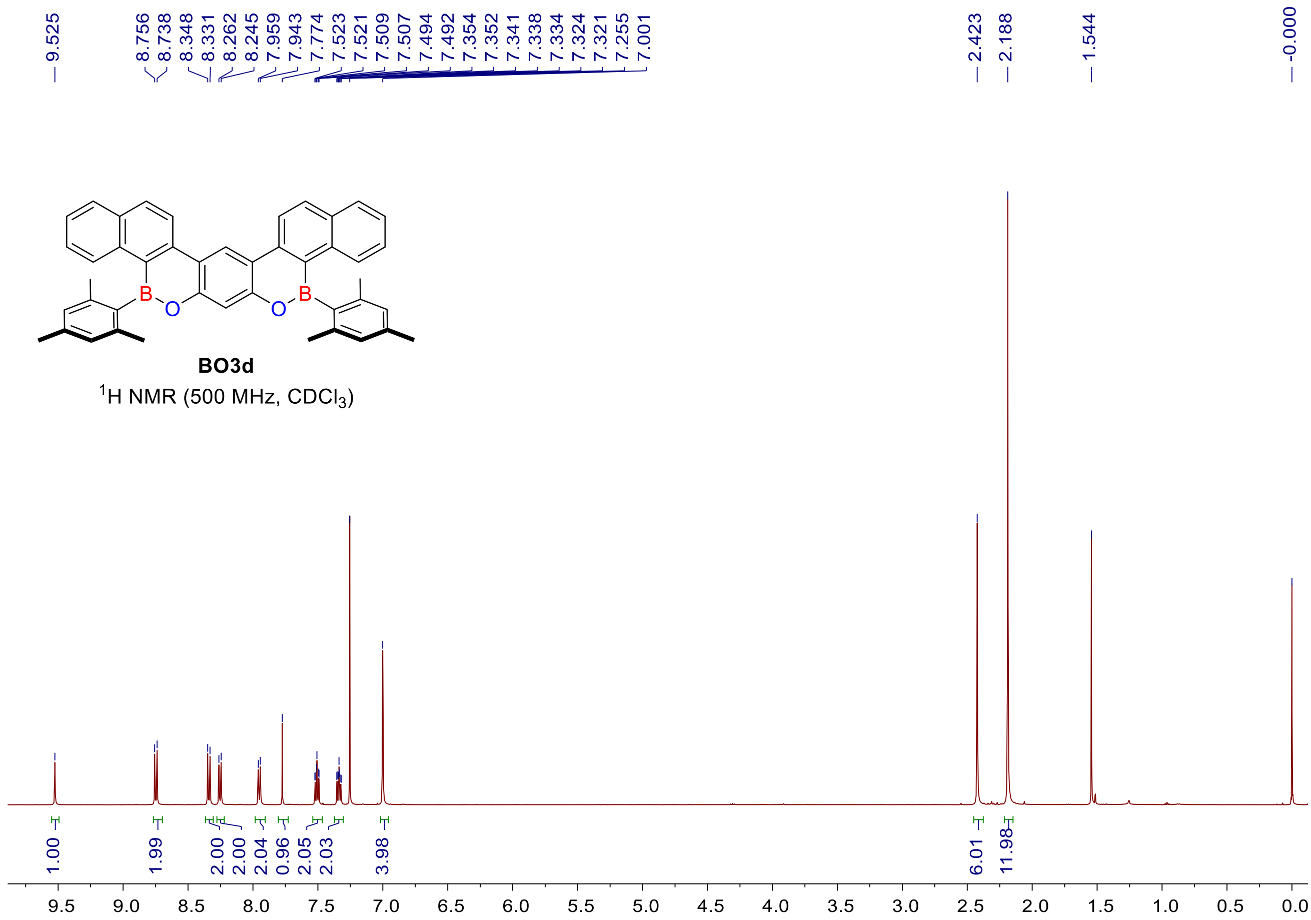
9.525
8.756
8.738
8.348
8.331
8.262
8.245
7.959
7.943
7.774
7.523
7.521
7.509
7.507
7.494
7.492
7.354
7.352
7.341
7.338
7.334
7.324
7.321
7.255
7.001
2.423
2.188
1.544
-0.000
B
O
BO3d
^{1}H NMR (500 MHz, $CDCl_3$)
1.00
1.99
2.00
2.00
2.04
0.96
2.05
2.03
3.98
6.01
11.98
9.5 9.0 8.5 8.0 7.5 7.0 6.5 6.0 5.5 5.0 4.5 4.0 3.5 3.0 2.5 2.0 1.5 1.0 0.5 0.0

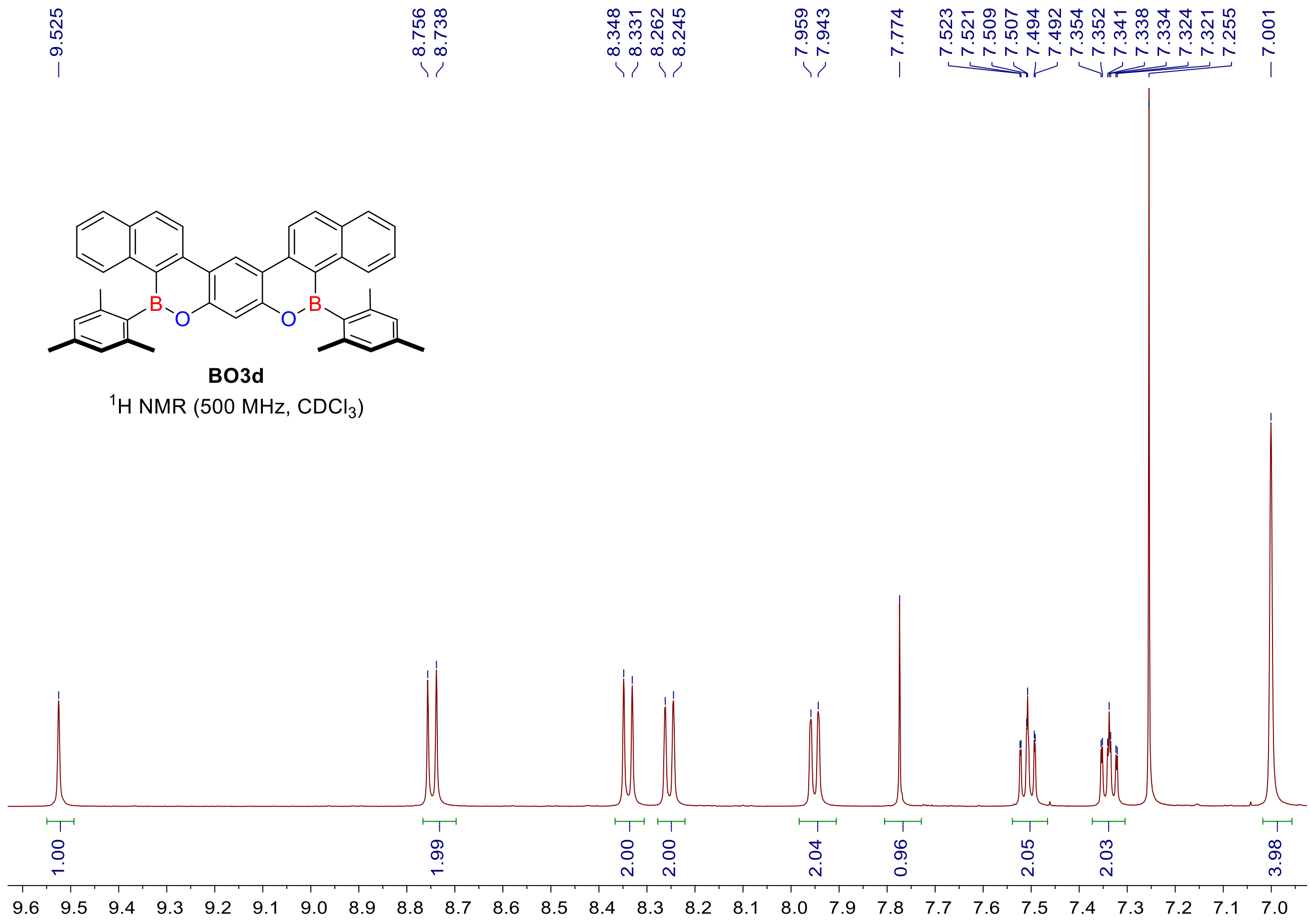
9.525
8.756
8.738
8.348
8.331
8.262
8.245
7.959
7.943
7.774
7.523
7.521
7.509
7.507
7.494
7.492
7.354
7.352
7.341
7.338
7.334
7.324
7.321
7.255
7.001
B
O
BO3d
^{1}H NMR (500 MHz, $CDCl_3$)
1.00
1.99
2.00
2.00
2.04
0.96
2.05
2.03
3.98
9.6 9.5 9.4 9.3 9.2 9.1 9.0 8.9 8.8 8.7 8.6 8.5 8.4 8.3 8.2 8.1 8.0 7.9 7.8 7.7 7.6 7.5 7.4 7.3 7.2 7.1 7.0

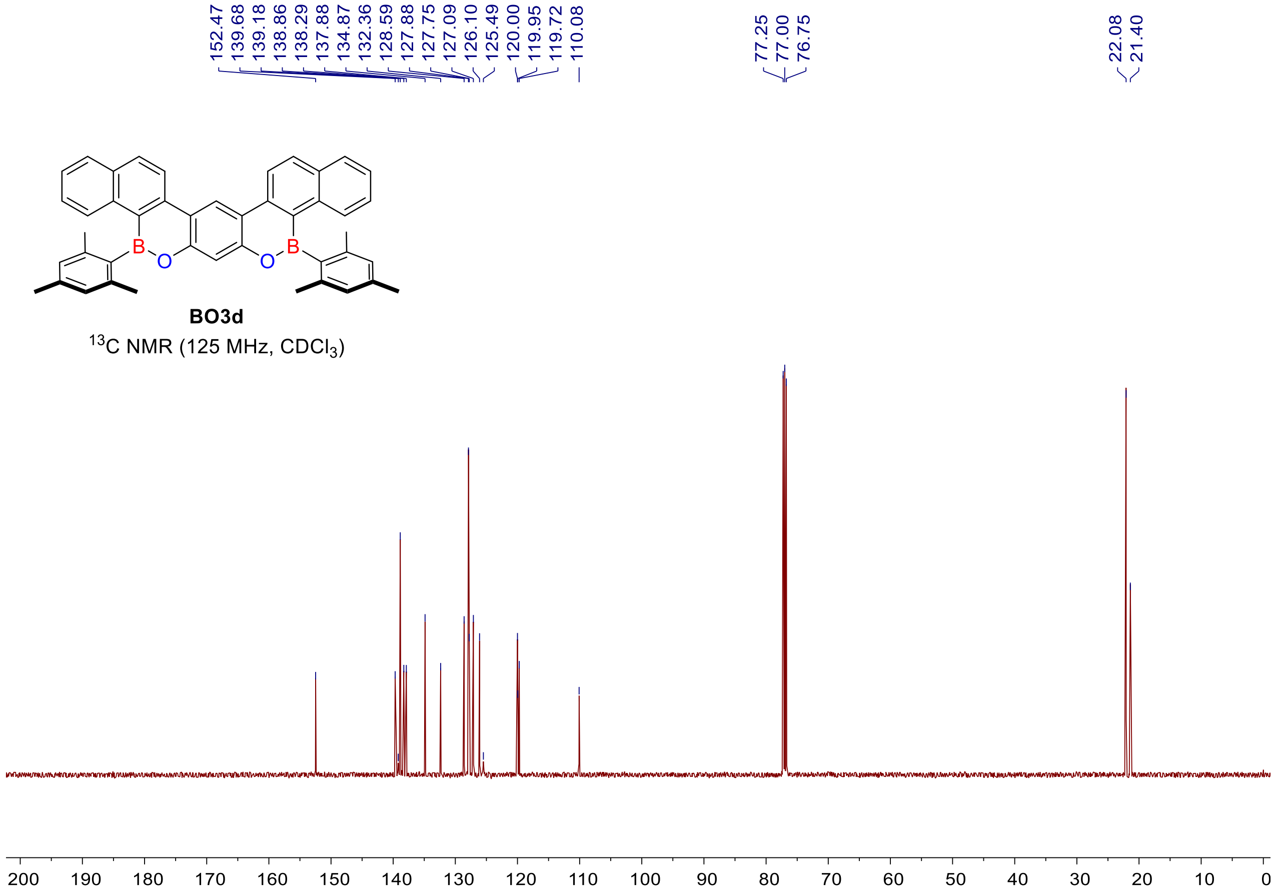

BO3d

^{13}C NMR (125 MHz, $CDCl_3$)

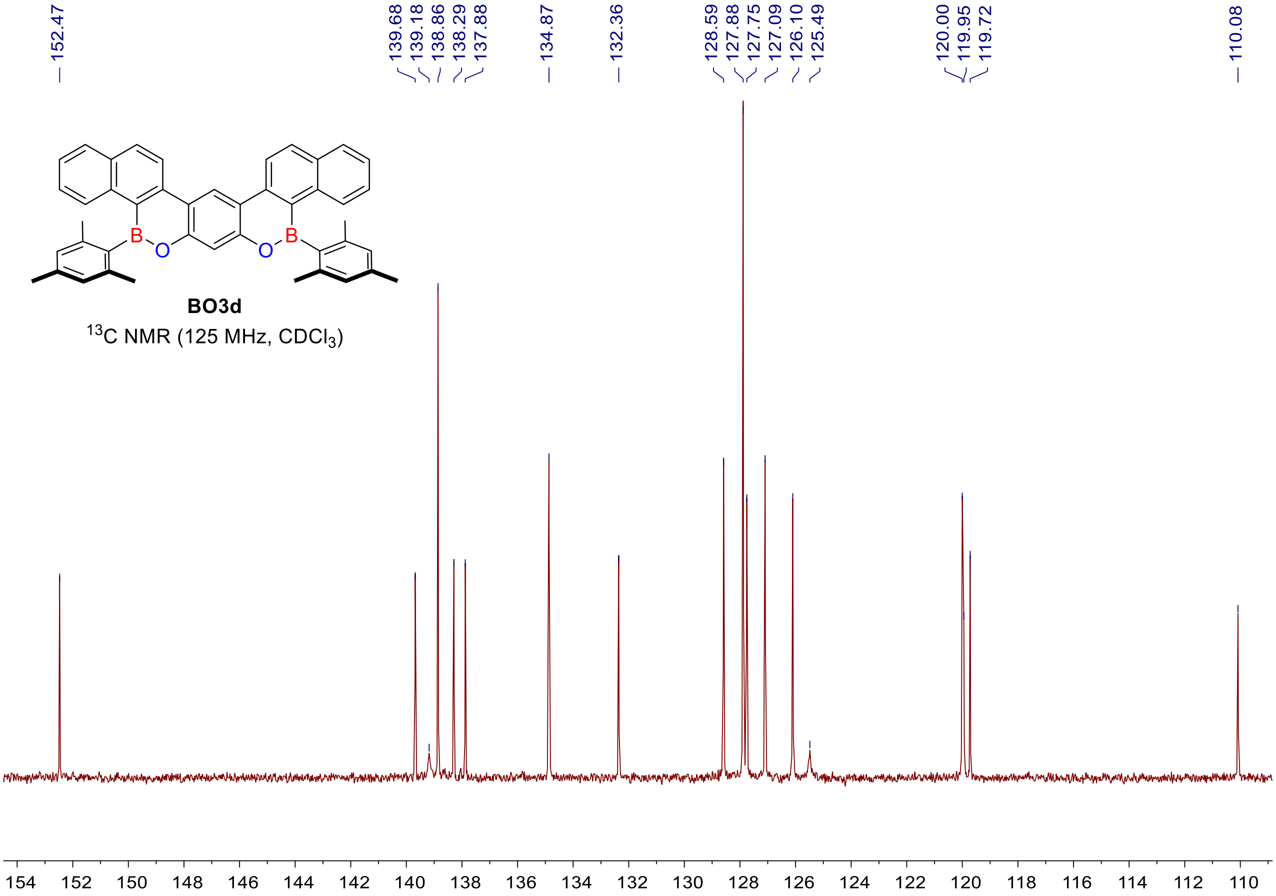

BO3d

^{13}C NMR (125 MHz, $CDCl_3$)

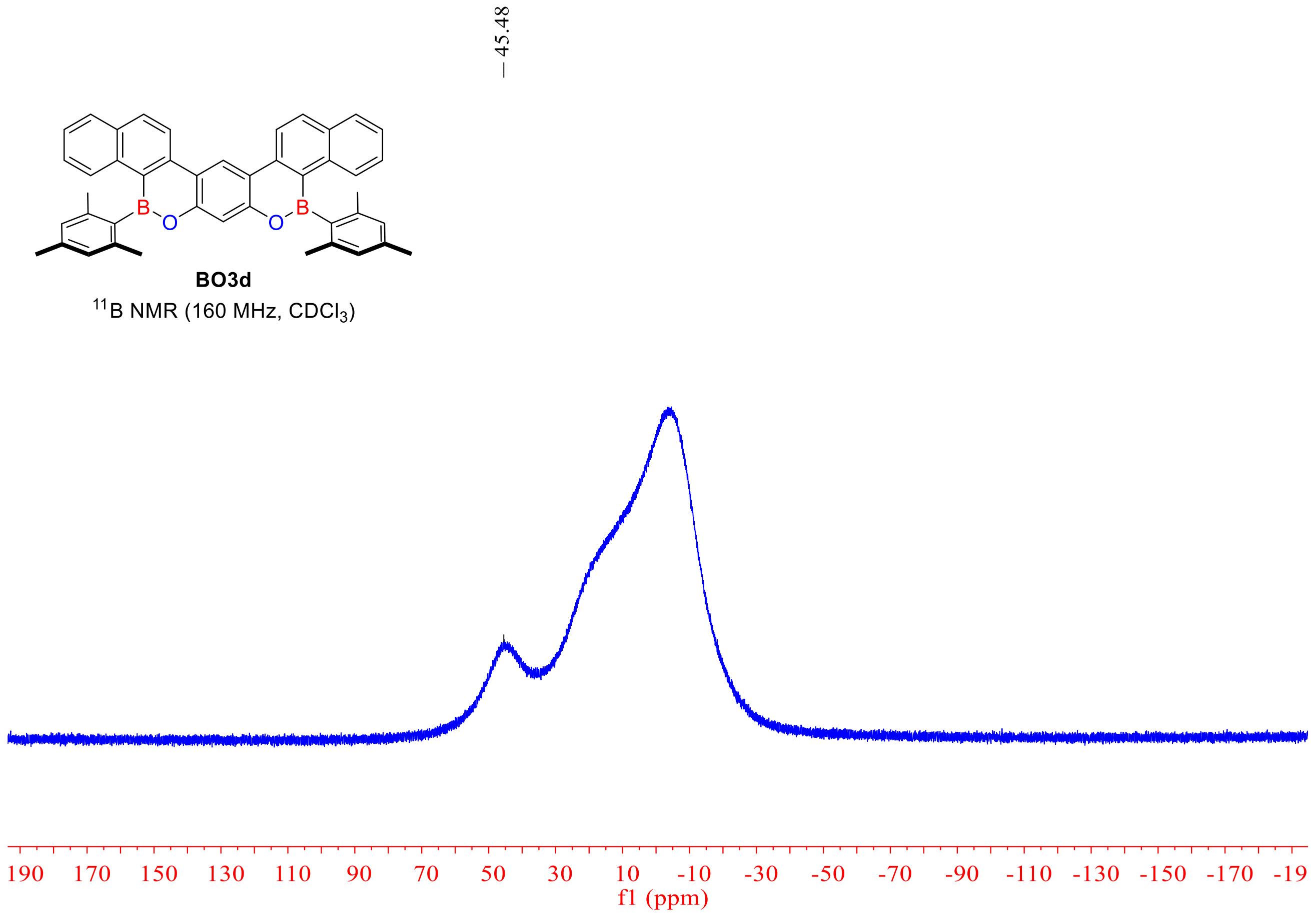

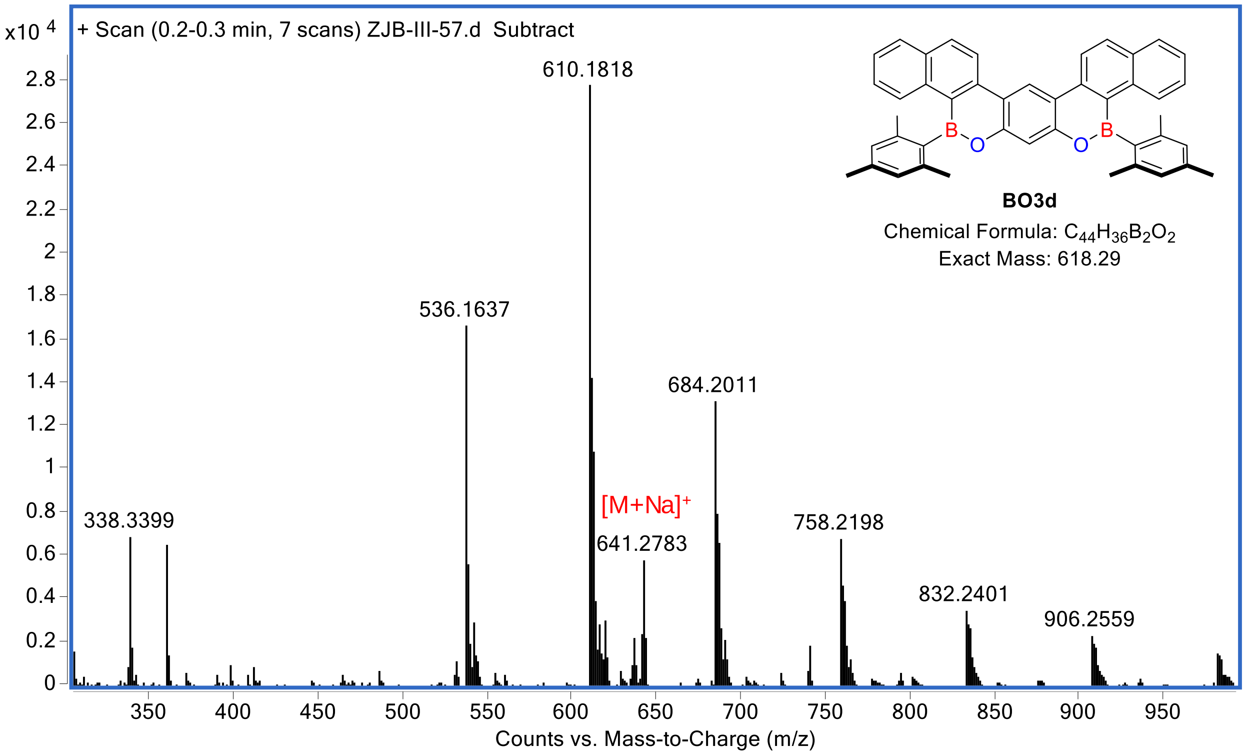

Formula (M)	Ion Formula	m/z	Calc m/z	Diff (ppm)
$C_{44}H_{36}[^{11}B]_2O_2$	$C_{44}H_{36}[^{11}B]_2NaO_2$	641.2783	641.2794	1.72

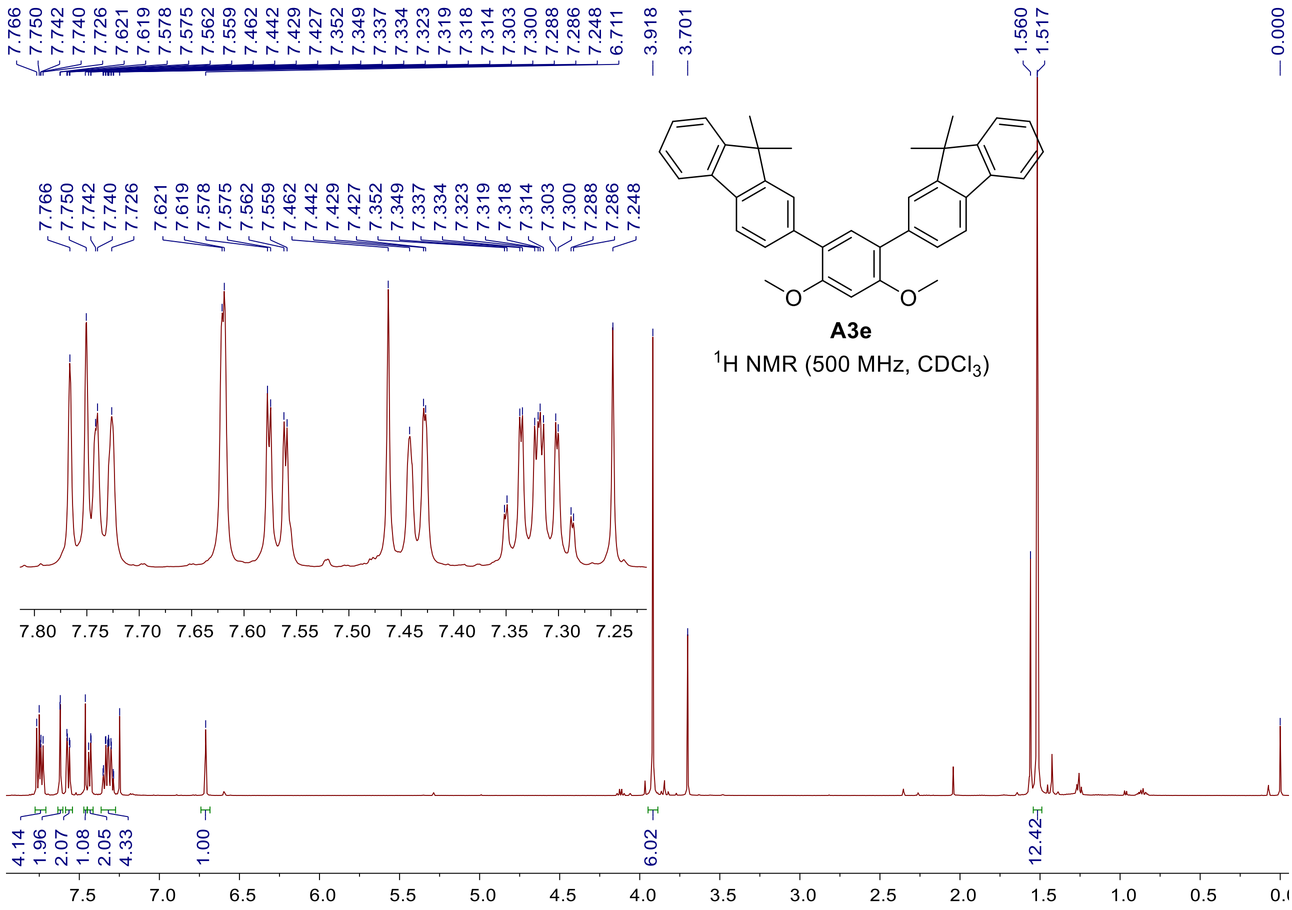

A3e

^{1}H NMR (500 MHz, $CDCl_3$)

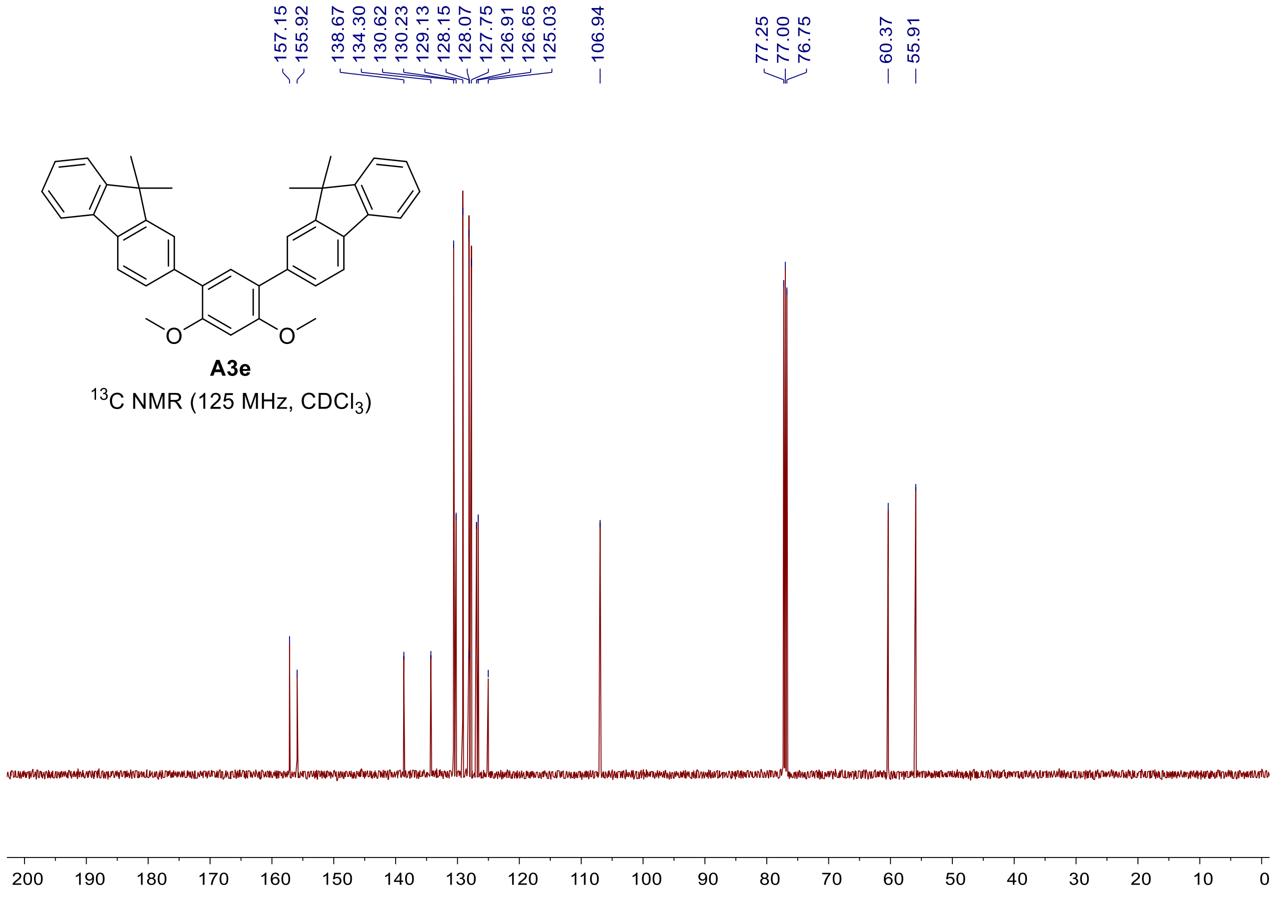

A3e

^{13}C NMR (125 MHz, $CDCl_3$)

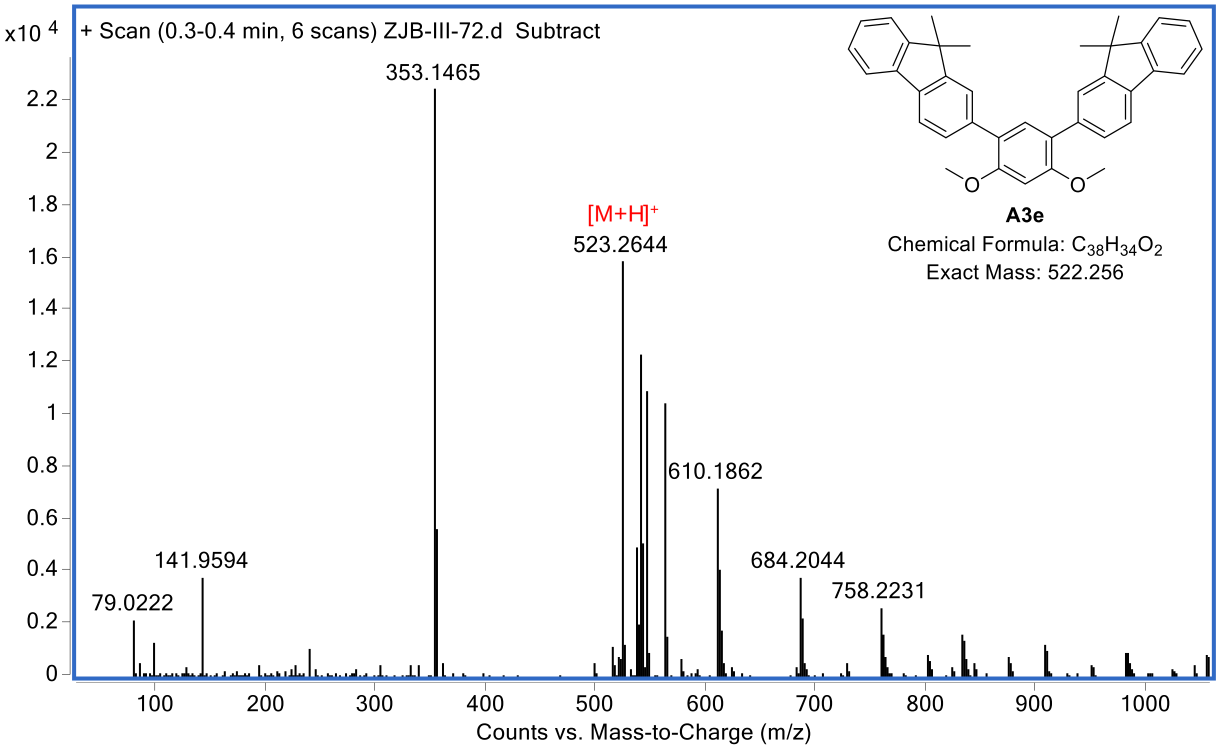

Formula (M)	Ion Formula	m/z	Calc m/z	Diff (ppm)	DBE
$C_{38}H_{34}O_2$	$C_{38}H_{35}O_2$	523.2644	523.2632	-2.38	22

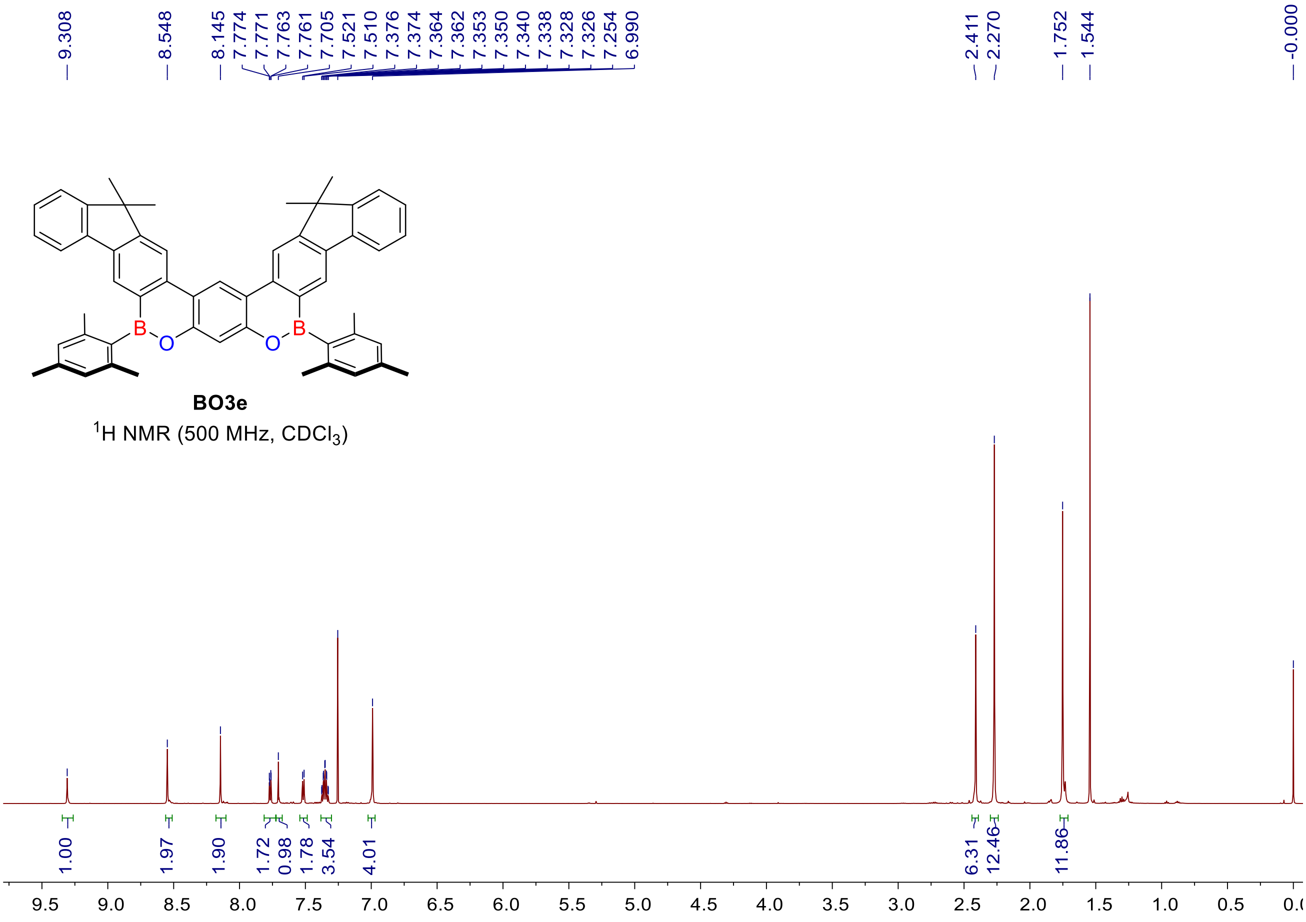

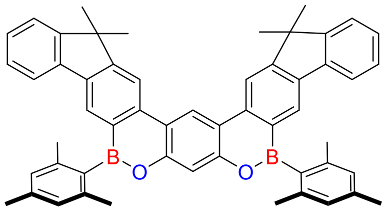

BO3e

^{1}H NMR (500 MHz, $CDCl_3$)

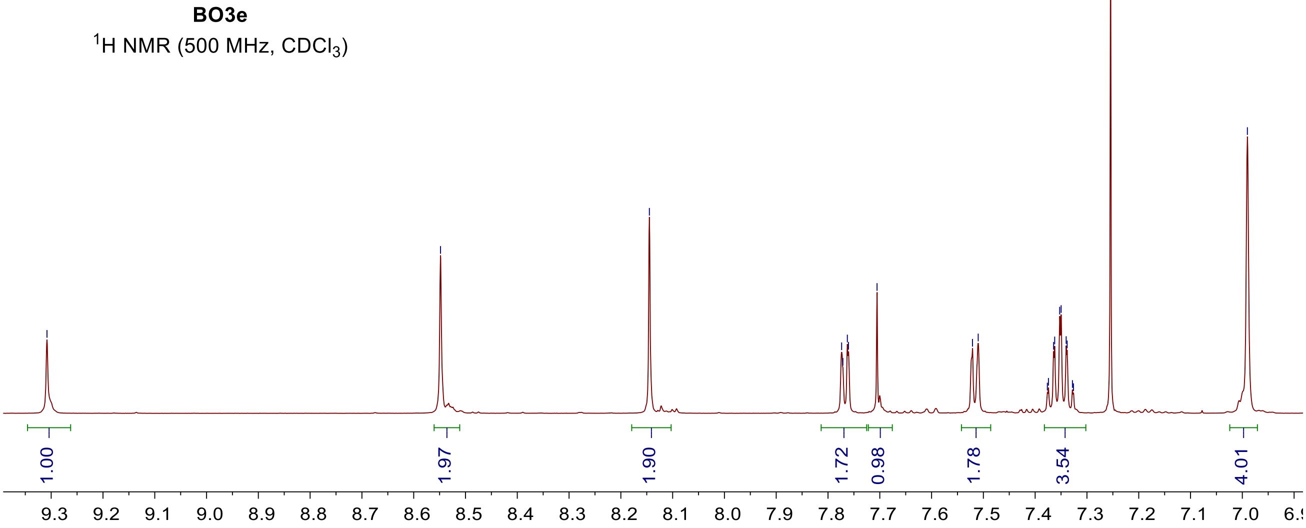

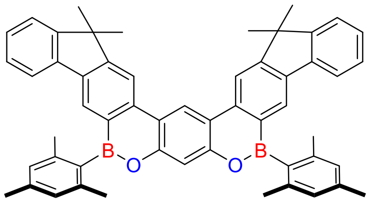

BO3e

^{13}C NMR (125 MHz, $CDCl_3$)

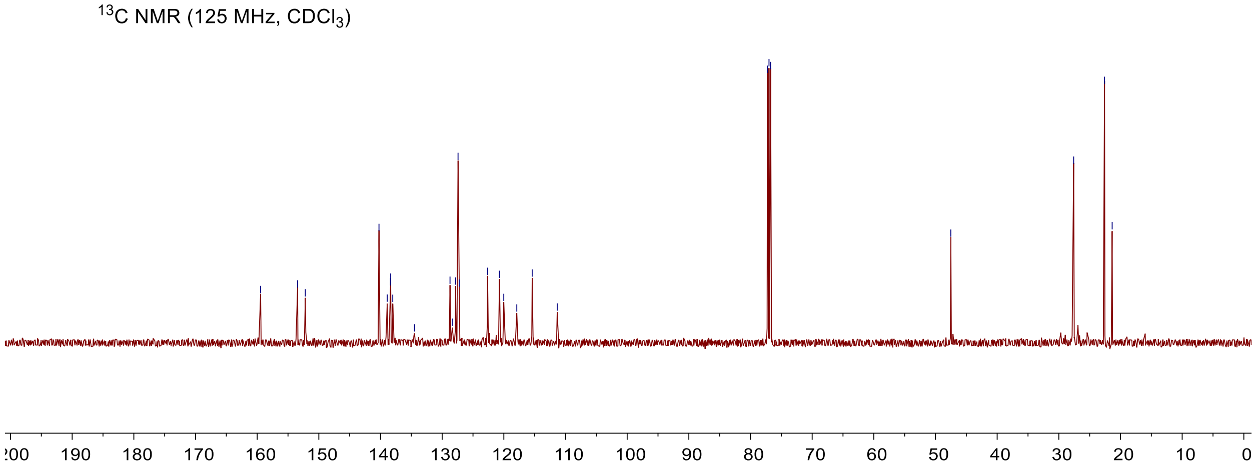

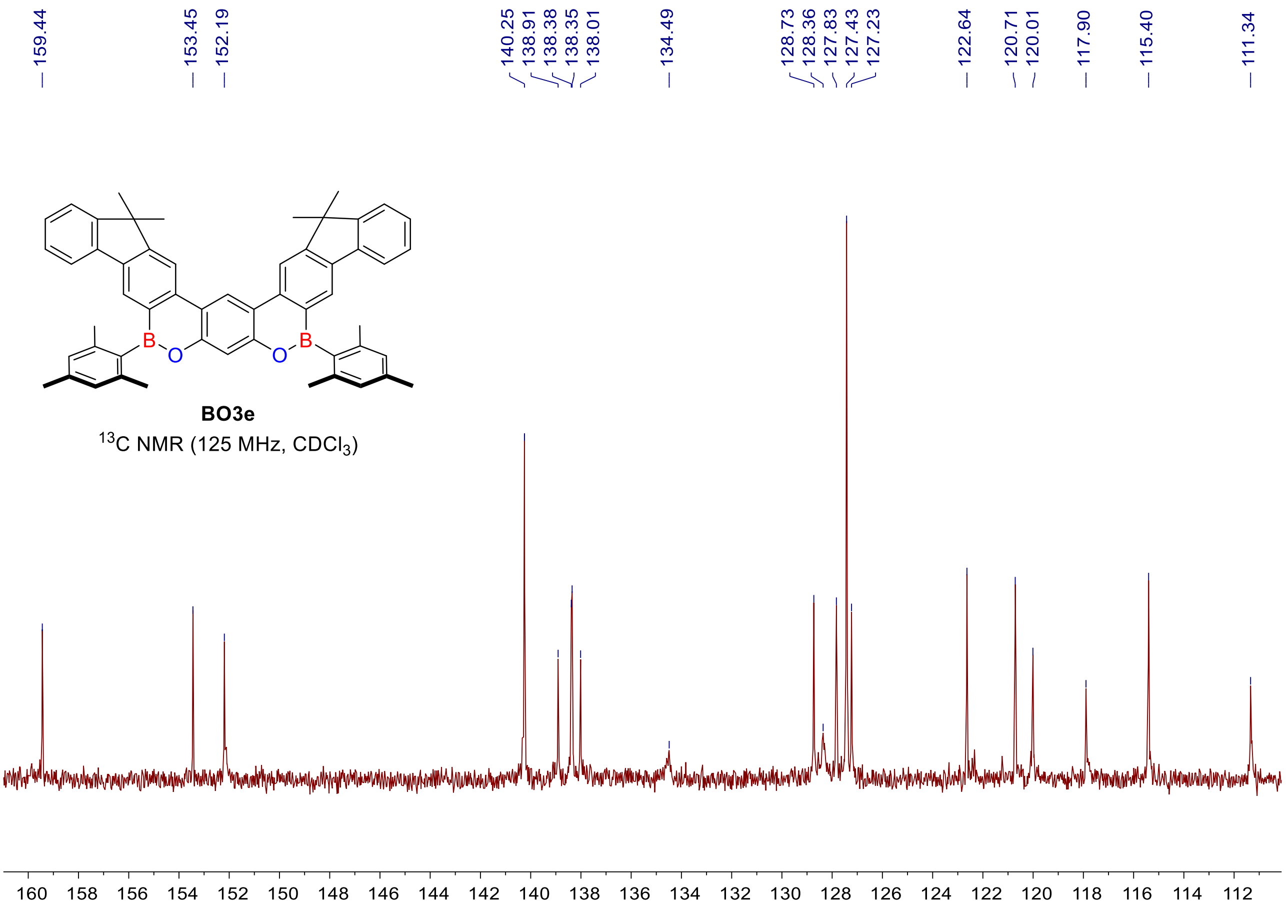
159.44
153.45
152.19
140.25
138.91
138.38
138.35
138.01
134.49
128.73
128.36
127.83
127.43
127.23
122.64
120.71
120.01
117.90
115.40
111.34
BO3e
^{13}C NMR (125 MHz, $CDCl_3$)
160 158 156 154 152 150 148 146 144 142 140 138 136 134 132 130 128 126 124 122 120 118 116 114 112

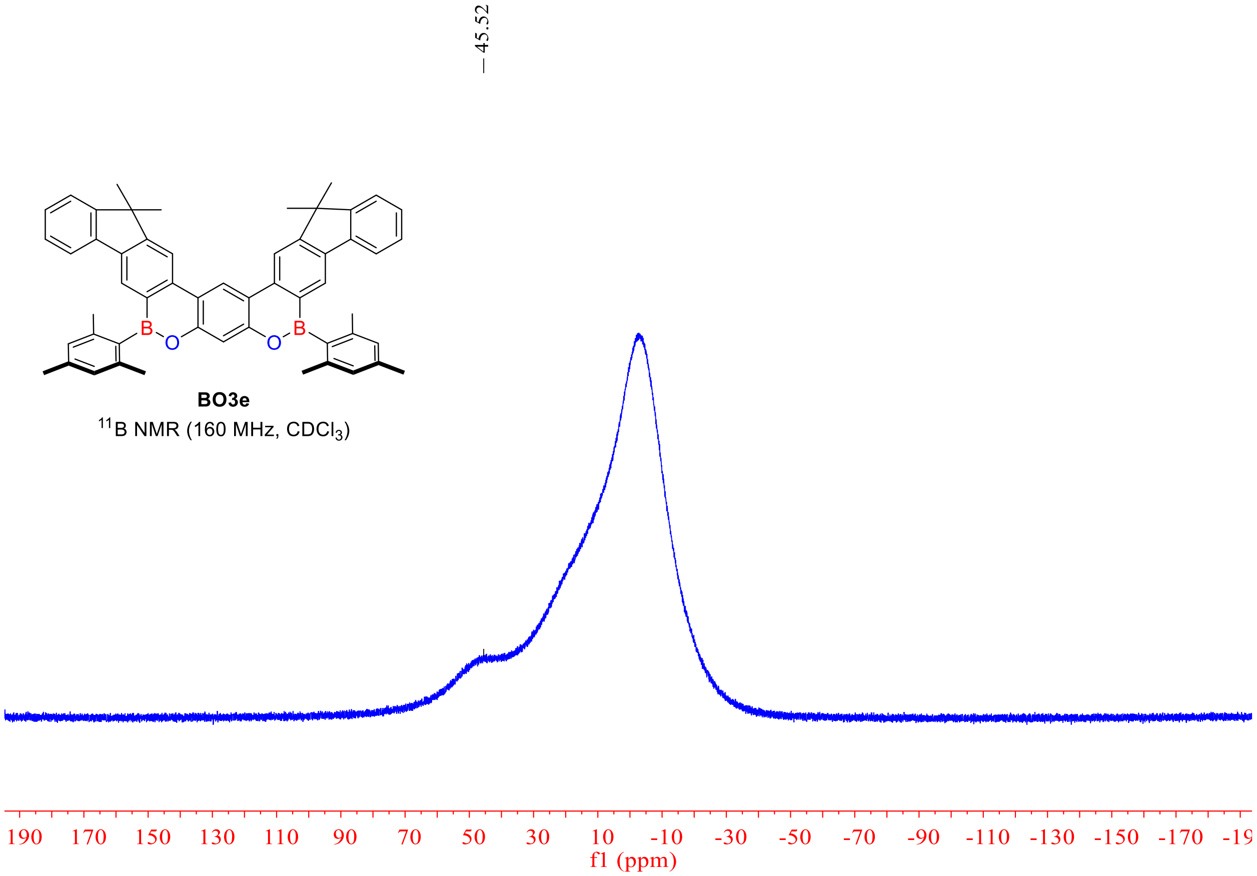
45.52
BO3e
^{11}B NMR (160 MHz, $CDCl_3$)
190 170 150 130 110 90 70 50 30 10 -10 -30 -50 -70 -90 -110 -130 -150 -170 -19
f1 (ppm)

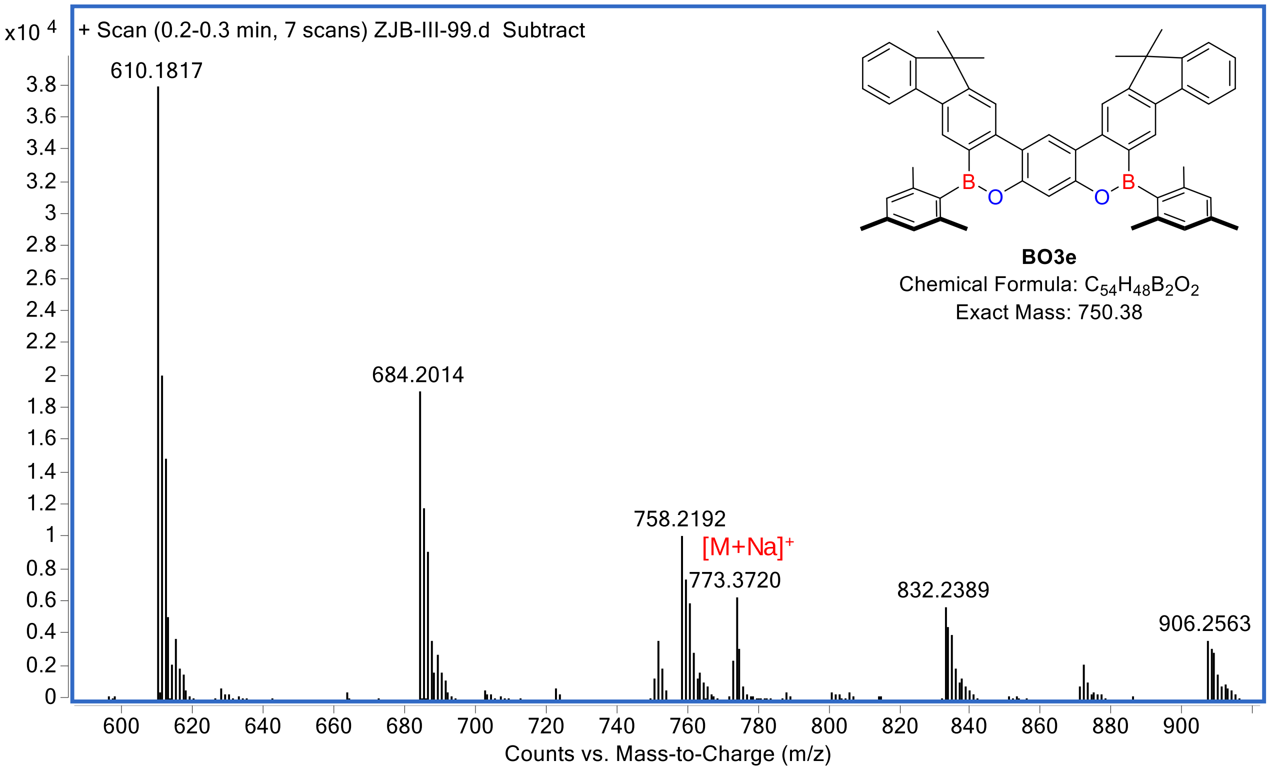

Formula (M)	Ion Formula	m/z	Calc m/z	Diff (ppm)
$C_{54}H_{48}[^{11}B]_2O_2$	$C_{54}H_{48}[^{11}B]_2NaO_2$	773.3720	773.3733	1.68

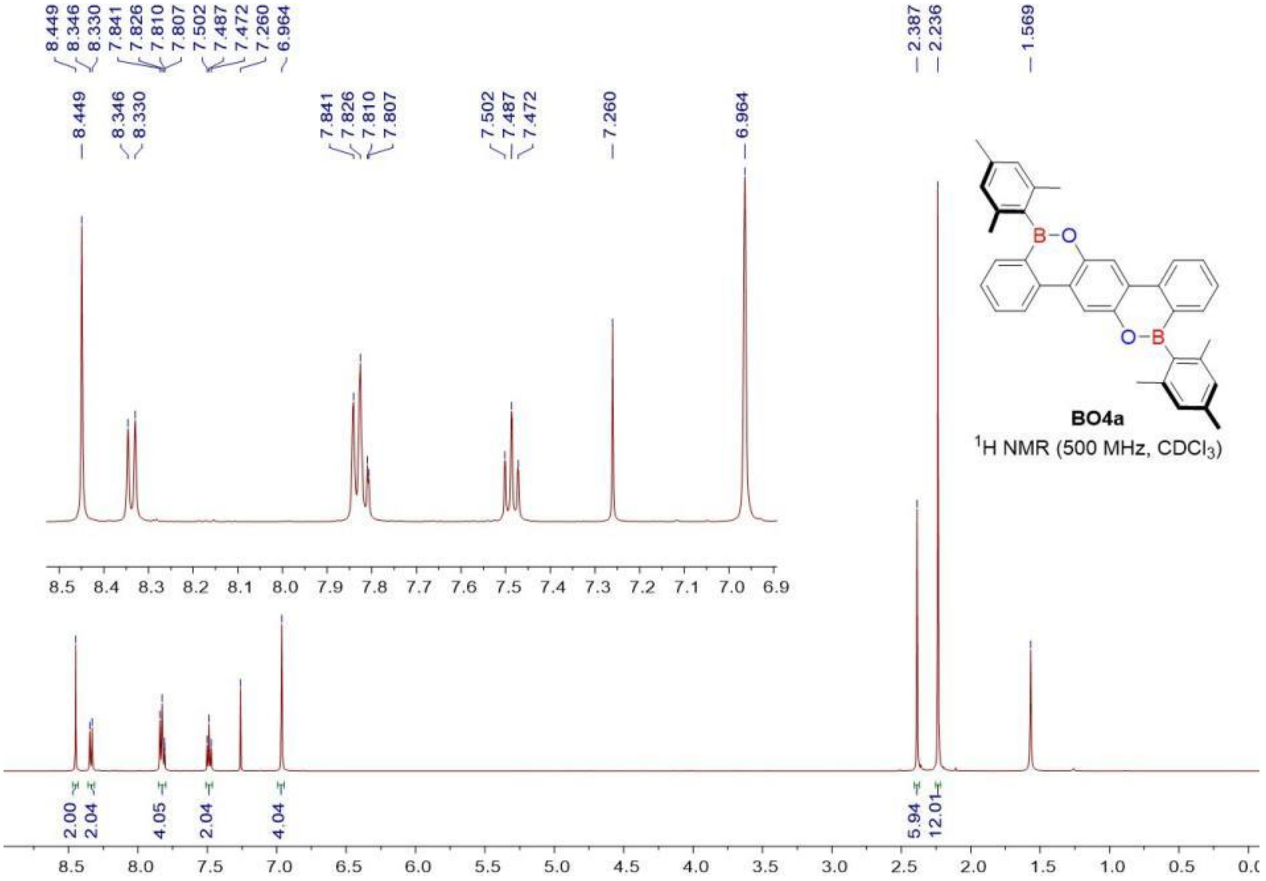

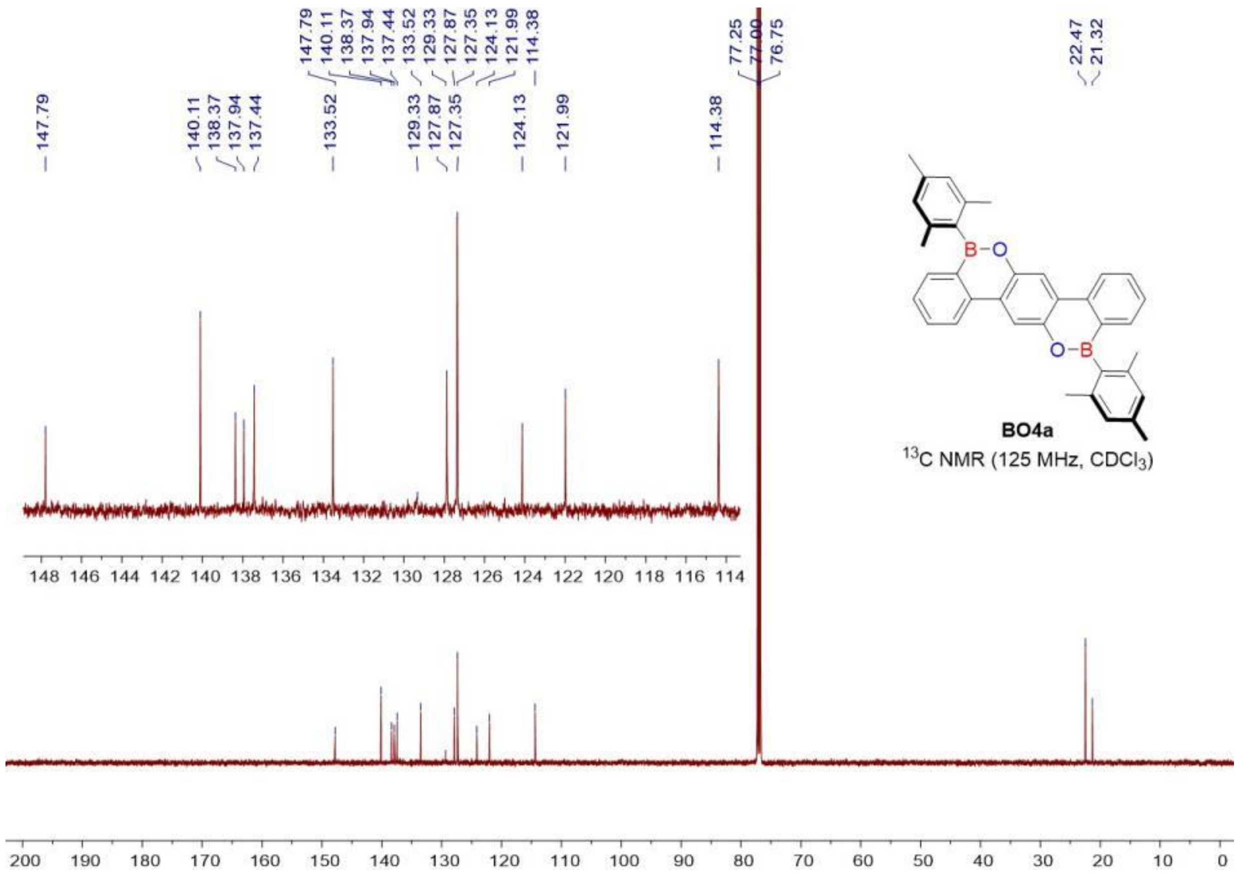
147.79
140.11
138.37
137.94
137.44
133.52
129.33
127.87
127.35
124.13
121.99
114.38
77.25
77.00
76.75
22.47
21.32
B
O
BO4a
^{13}C NMR (125 MHz, $CDCl_3$)
148 146 144 142 140 138 136 134 132 130 128 126 124 122 120 118 116 114
200 190 180 170 160 150 140 130 120 110 100 90 80 70 60 50 40 30 20 10 0

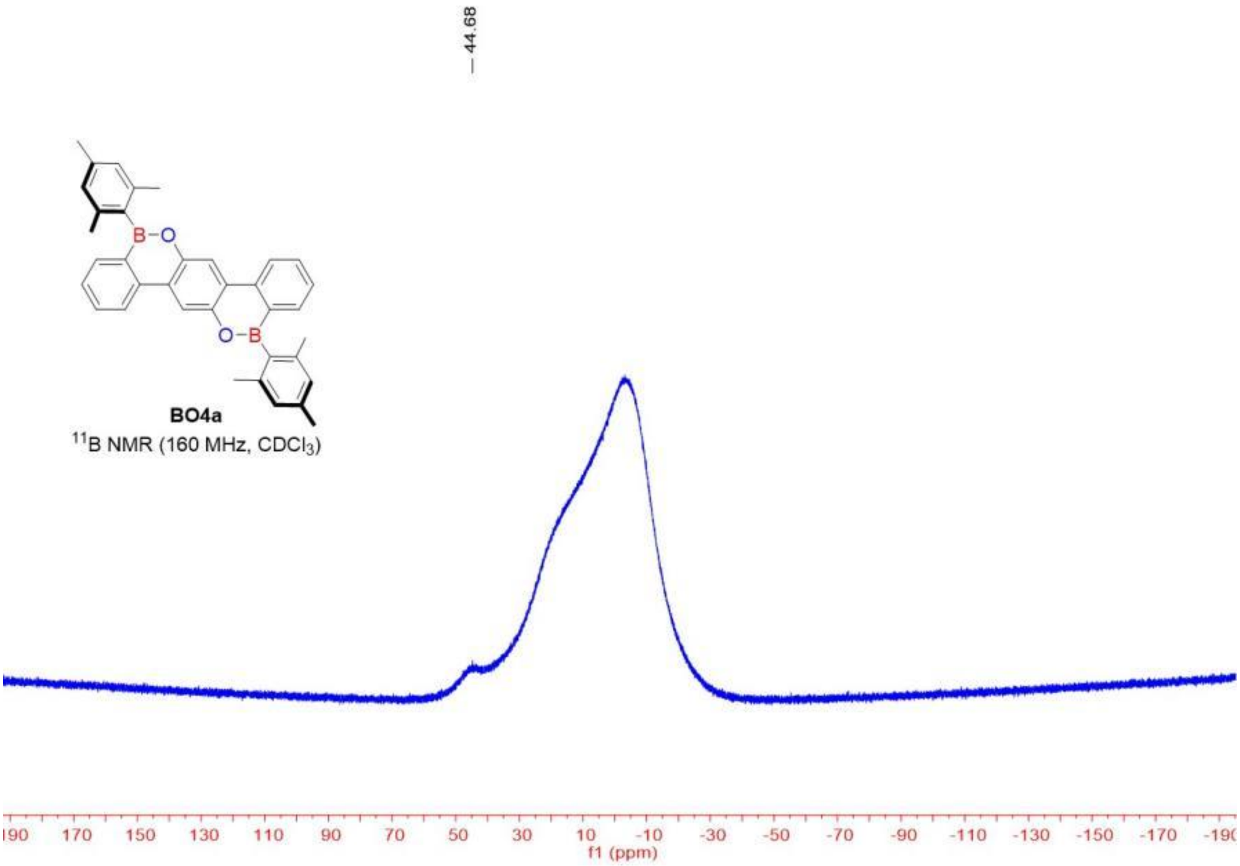
44.68
B
O
BO4a
^{11}B NMR (160 MHz, $CDCl_3$)
190 170 150 130 110 90 70 50 30 10 -10 -30 -50 -70 -90 -110 -130 -150 -170 -190
f1 (ppm)

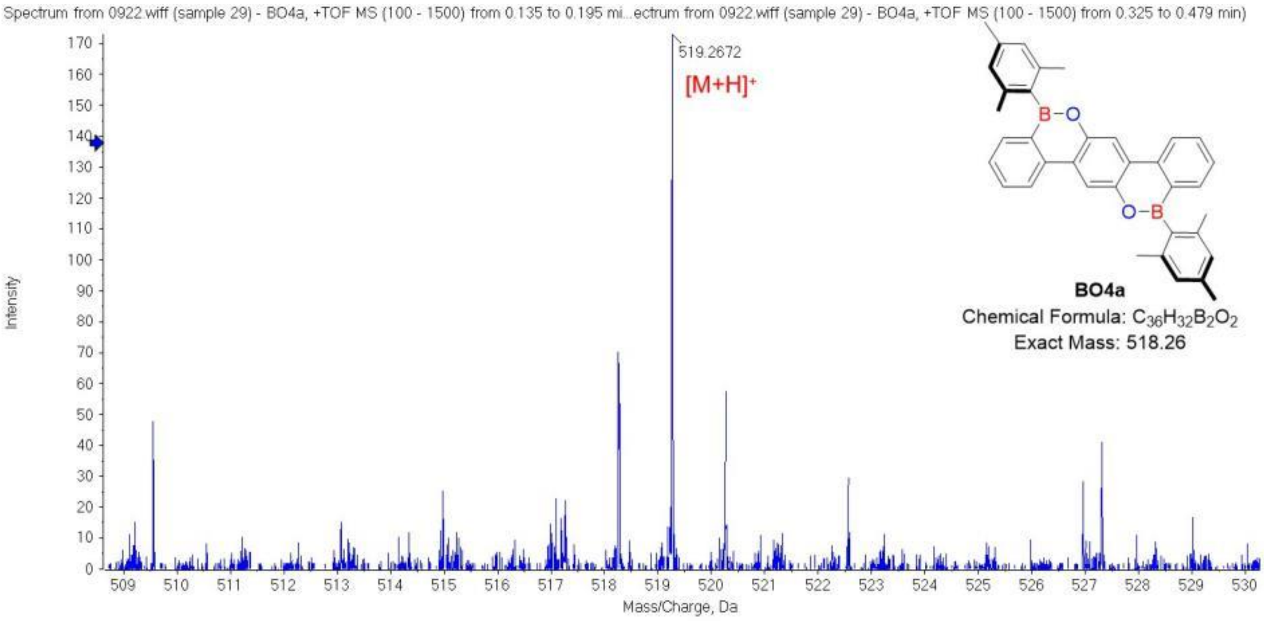

Formula (M)	Ion Formula	m/z	Calc m/z	Diff (ppm)
$C_{36}H_{32}[^{11}B]_2O_2$	$C_{36}H_{33}[^{11}B]_2O_2$	519.2672	519.2661	-2.1

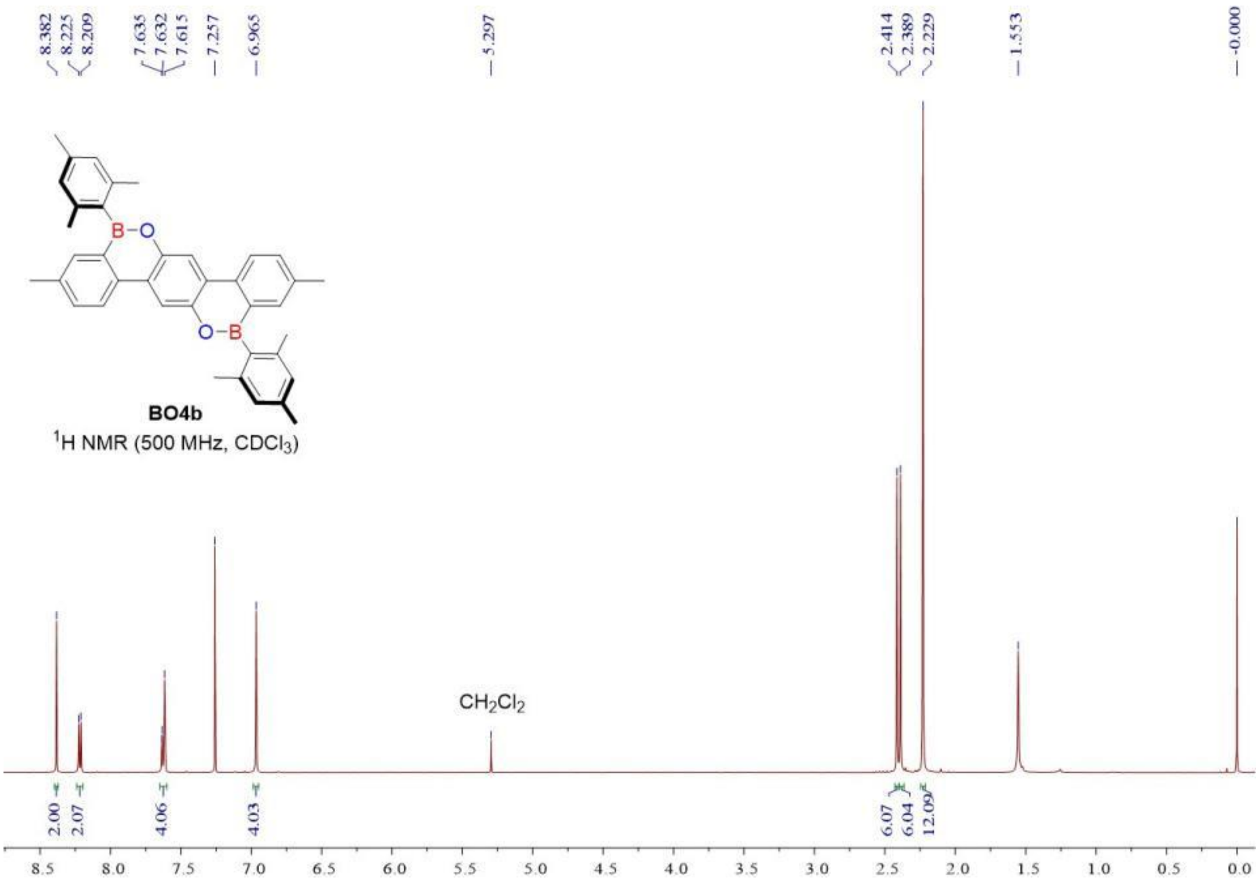

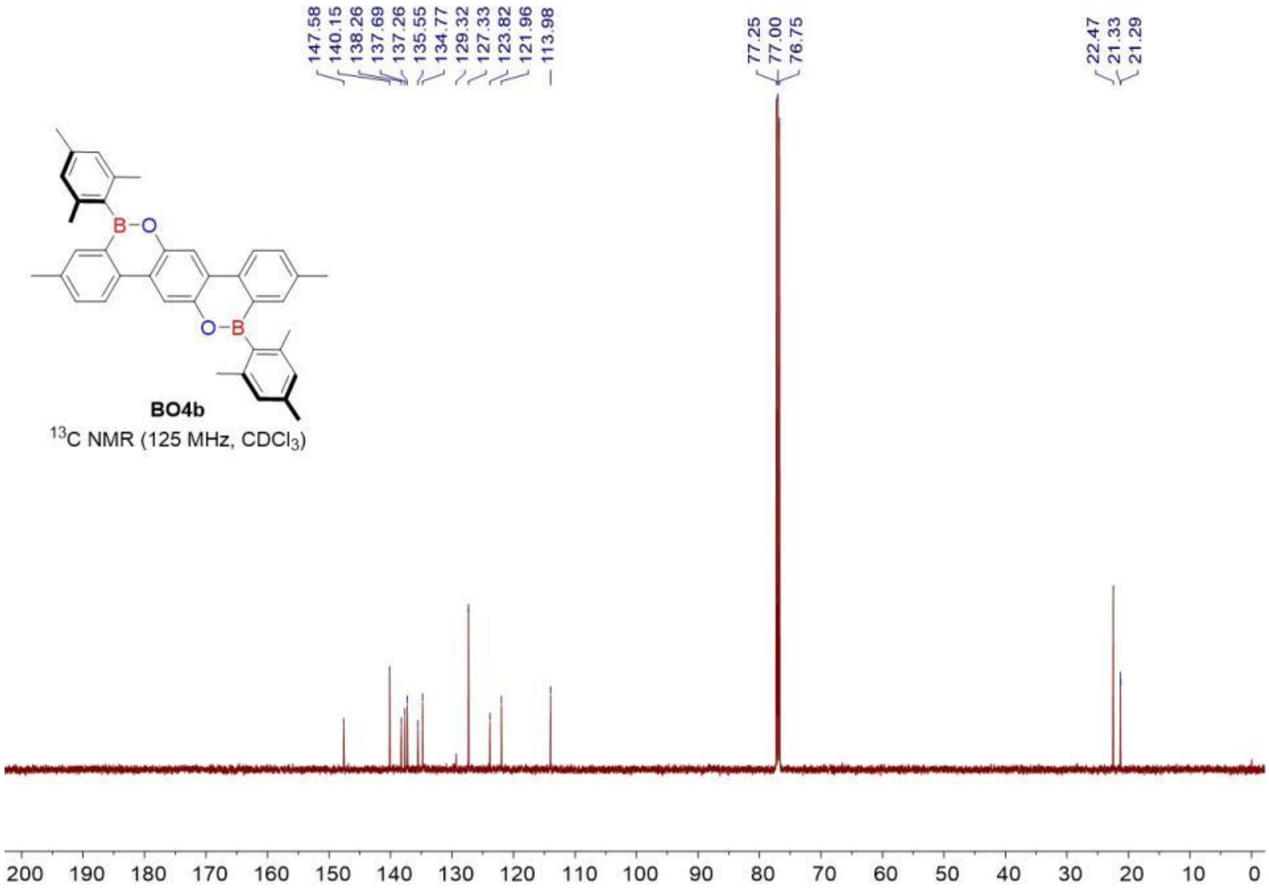
147.58
140.15
138.26
137.69
137.26
135.55
134.77
129.32
127.33
123.82
121.96
113.98
77.25
77.00
76.75
22.47
21.33
21.29
B
O
O
B
BO4b
^{13}C NMR (125 MHz, CDCl$_3$)
200 190 180 170 160 150 140 130 120 110 100 90 80 70 60 50 40 30 20 10 0

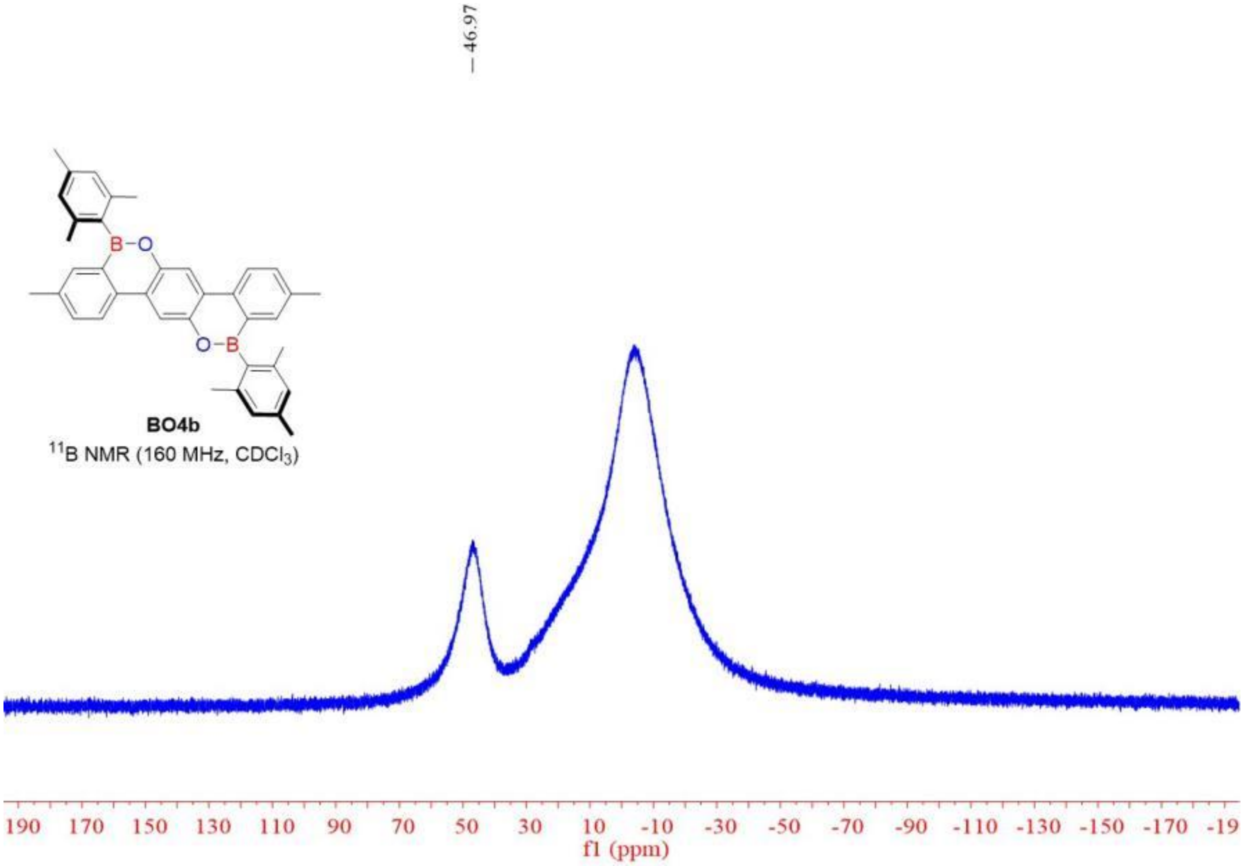
46.97
B
O
O
B
BO4b
^{11}B NMR (160 MHz, CDCl$_3$)
190 170 150 130 110 90 70 50 30 10 -10 -30 -50 -70 -90 -110 -130 -150 -170 -19
f1 (ppm)

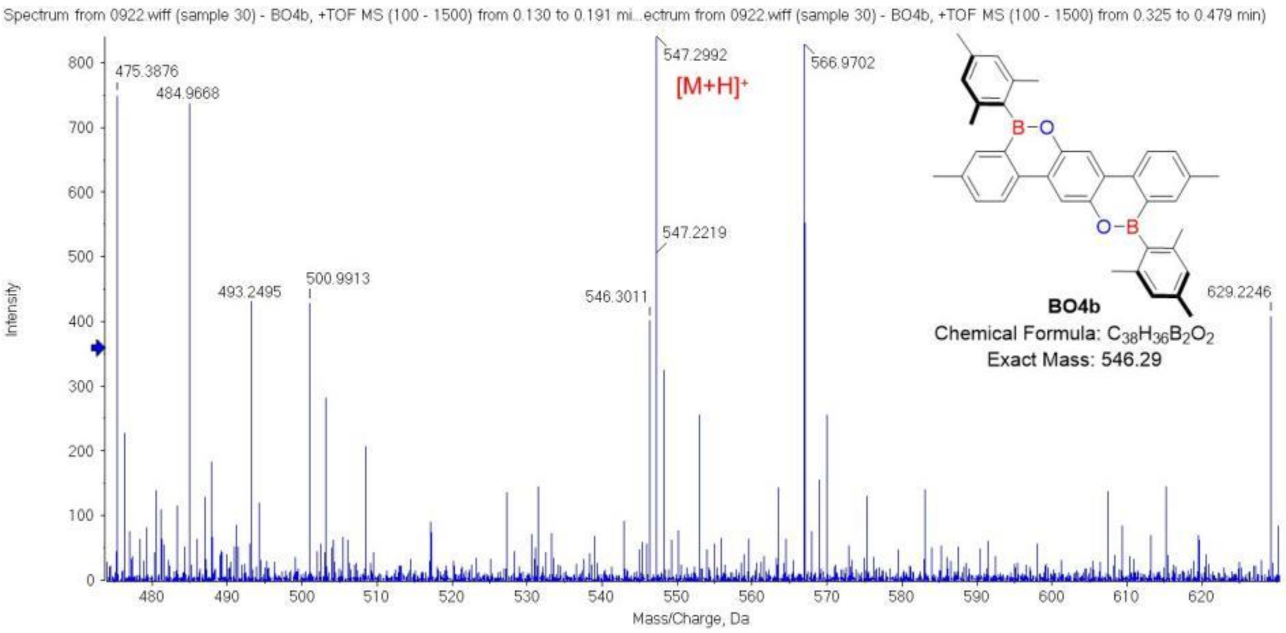

Formula (M)	Ion Formula	m/z	Calc m/z	Diff (ppm)
$C_{38}H_{36}[^{11}B]_2O_2$	$C_{38}H_{37}[^{11}B]_2O_2$	547.2992	547.2974	-3.3

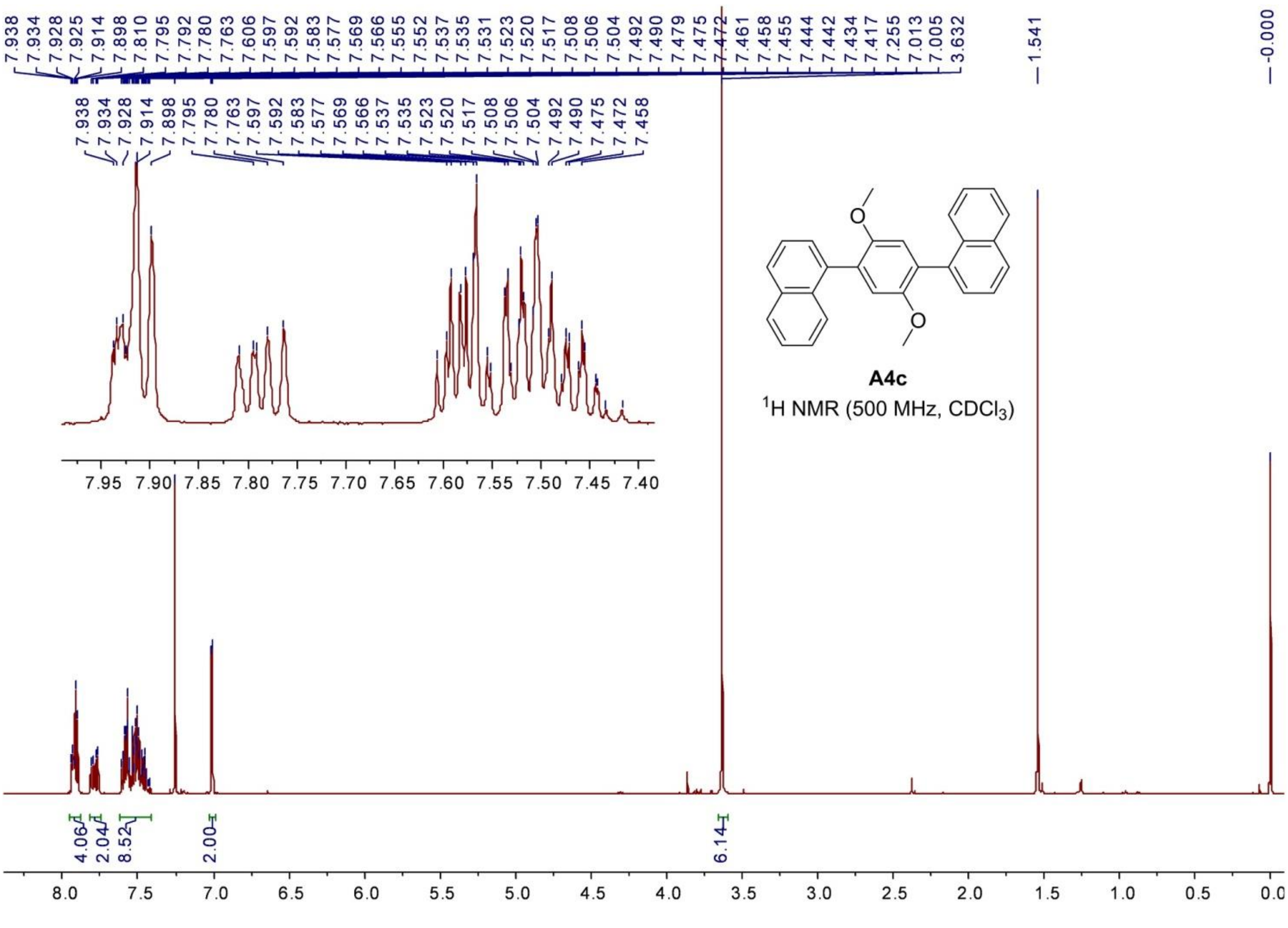

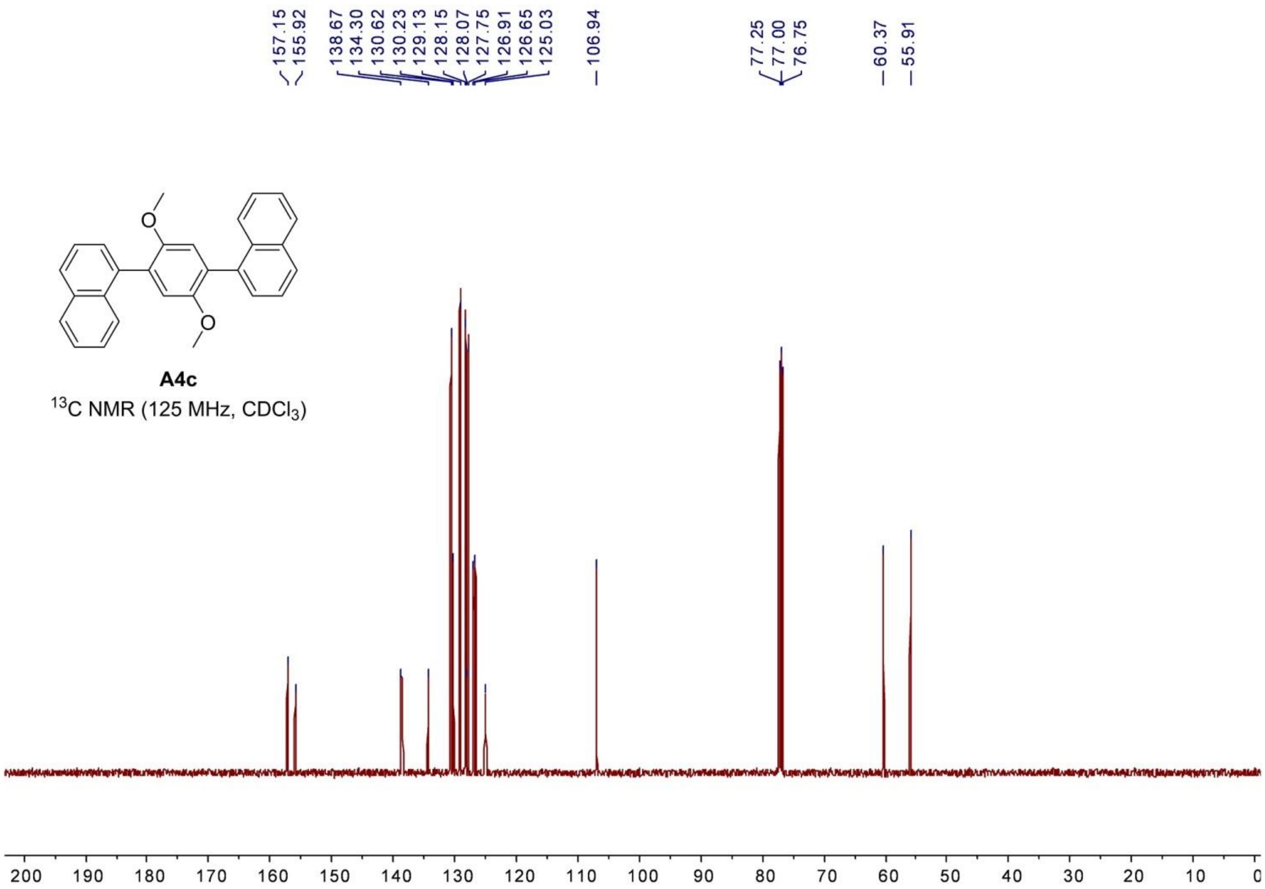

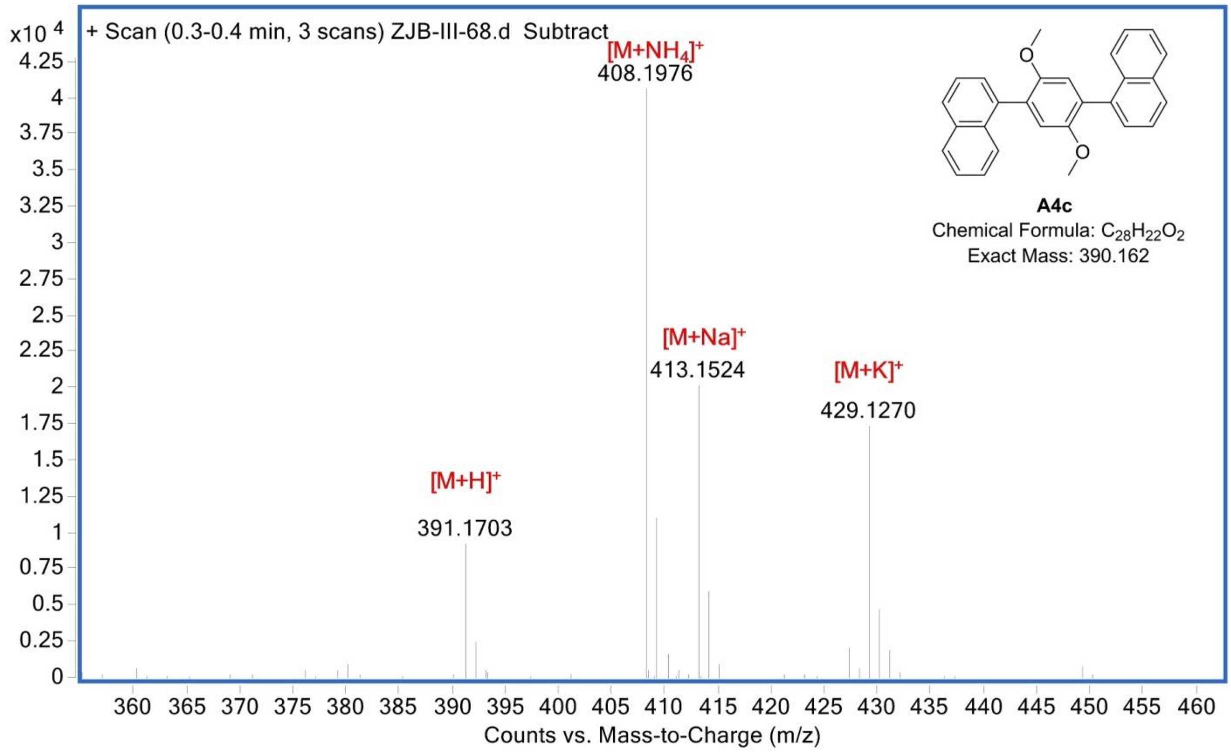

Formula (M)	Ion Formula	m/z	Calc m/z	Diff (ppm)	DBE
$C_{28}H_{22}O_2$	$C_{28}H_{26}NO_2$	408.1976	408.1958	-4.6	18

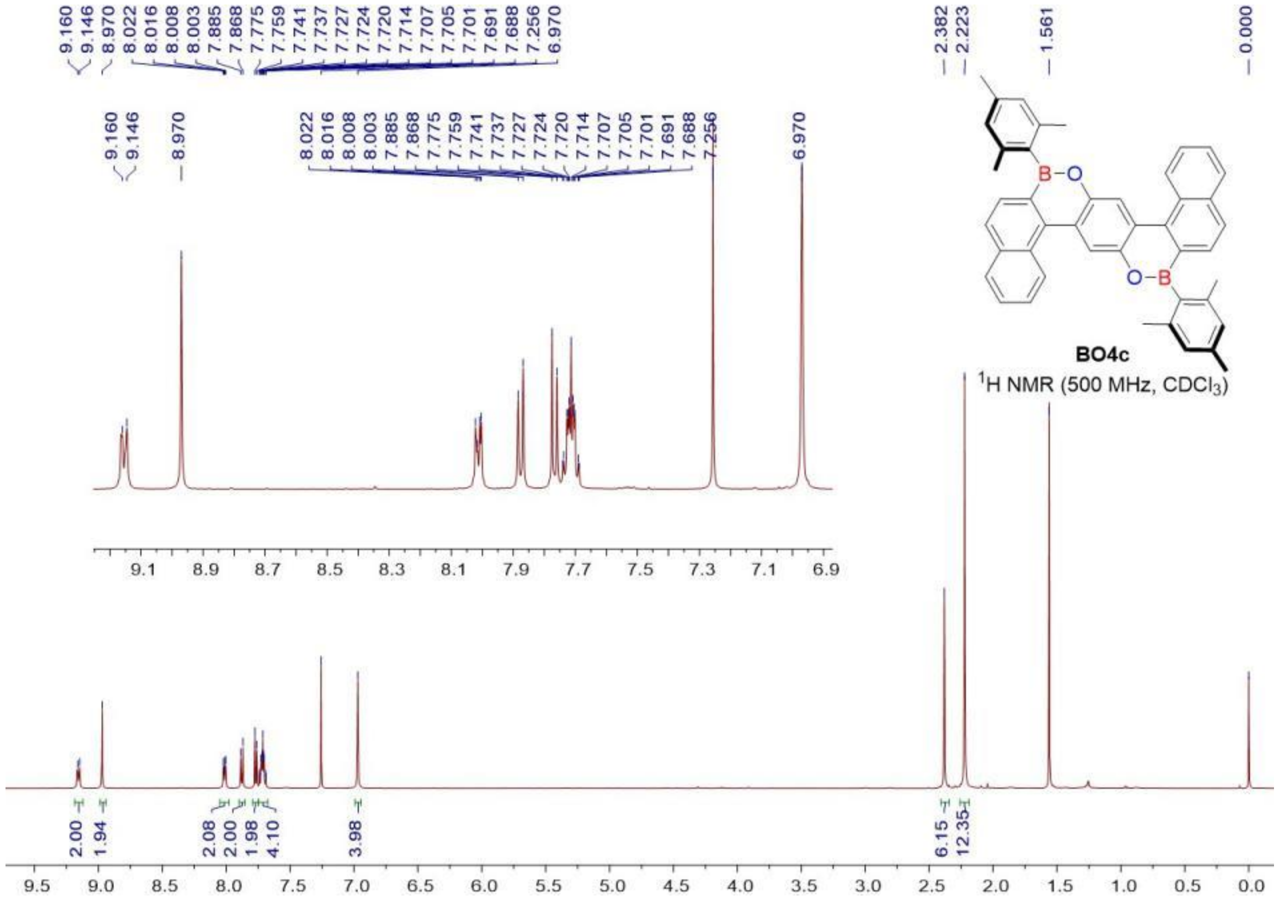
9.160
9.146
8.970
8.022
8.016
8.008
8.003
7.885
7.868
7.775
7.759
7.741
7.737
7.727
7.724
7.720
7.714
7.707
7.705
7.701
7.691
7.688
7.256
6.970
2.382
2.223
1.561
0.000
BO4c
1H NMR (500 MHz, CDCl3)
2.00
1.94
2.08
2.00
1.98
4.10
3.98
6.15
12.35

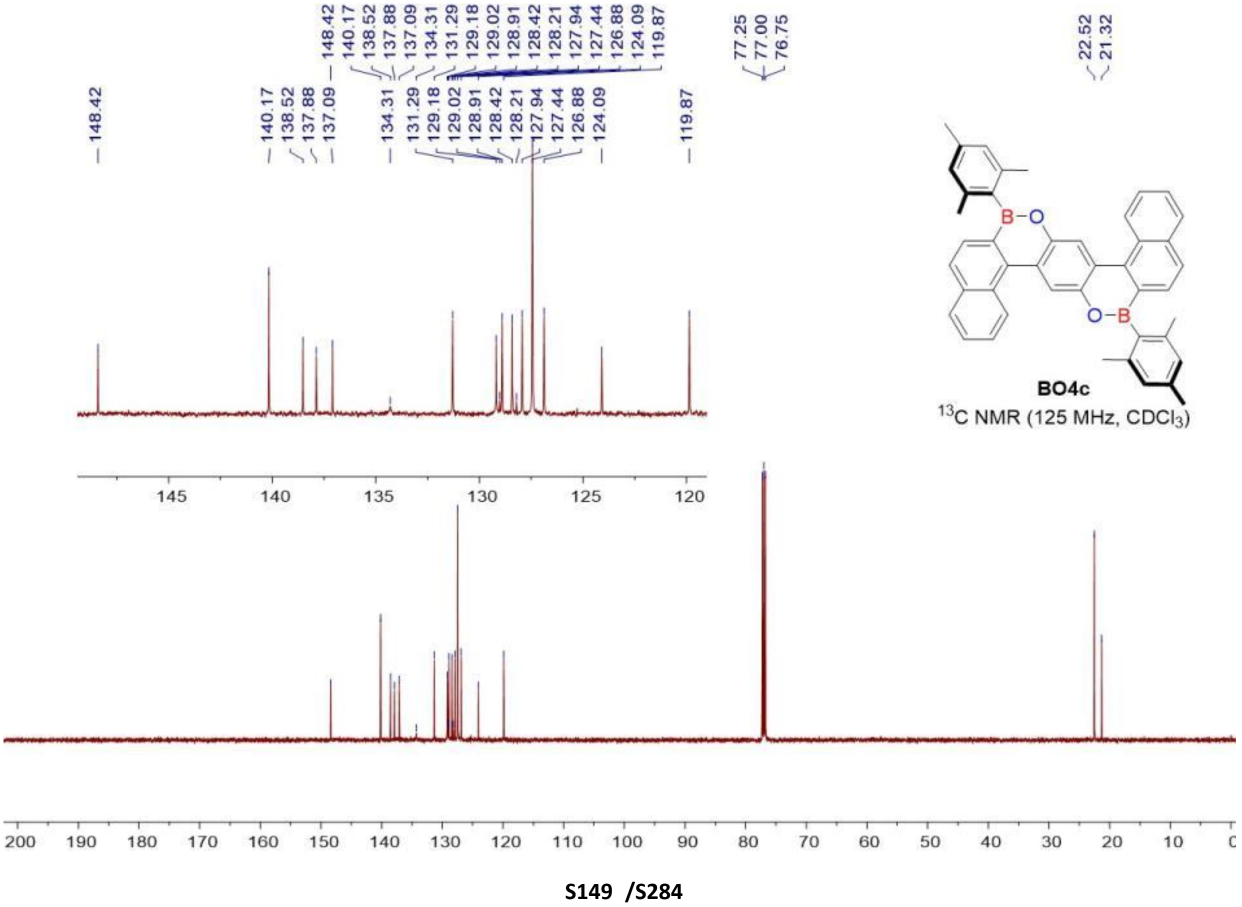
148.42
140.17
138.52
137.88
137.09
134.31
131.29
129.18
129.02
128.91
128.42
128.21
127.94
127.44
126.88
124.09
119.87
77.25
77.00
76.75
22.52
21.32
BO4c
13C NMR (125 MHz, CDCl3)

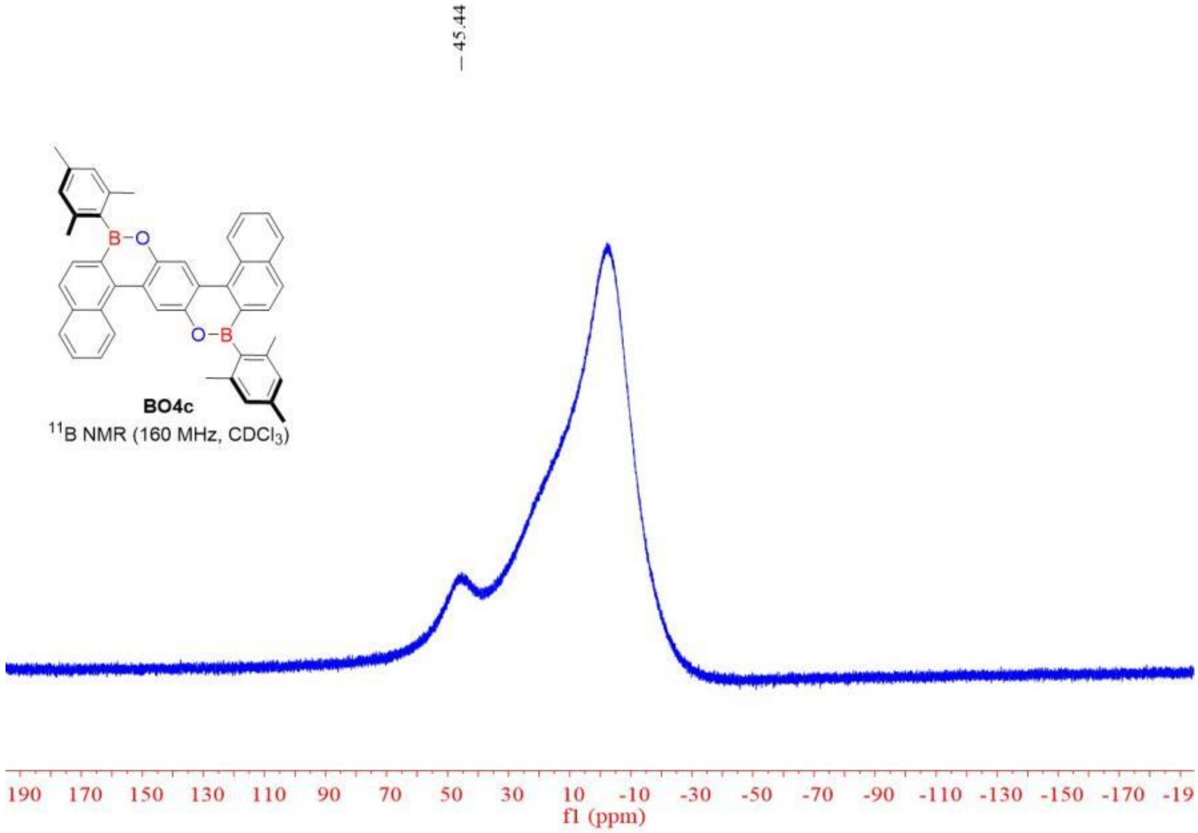

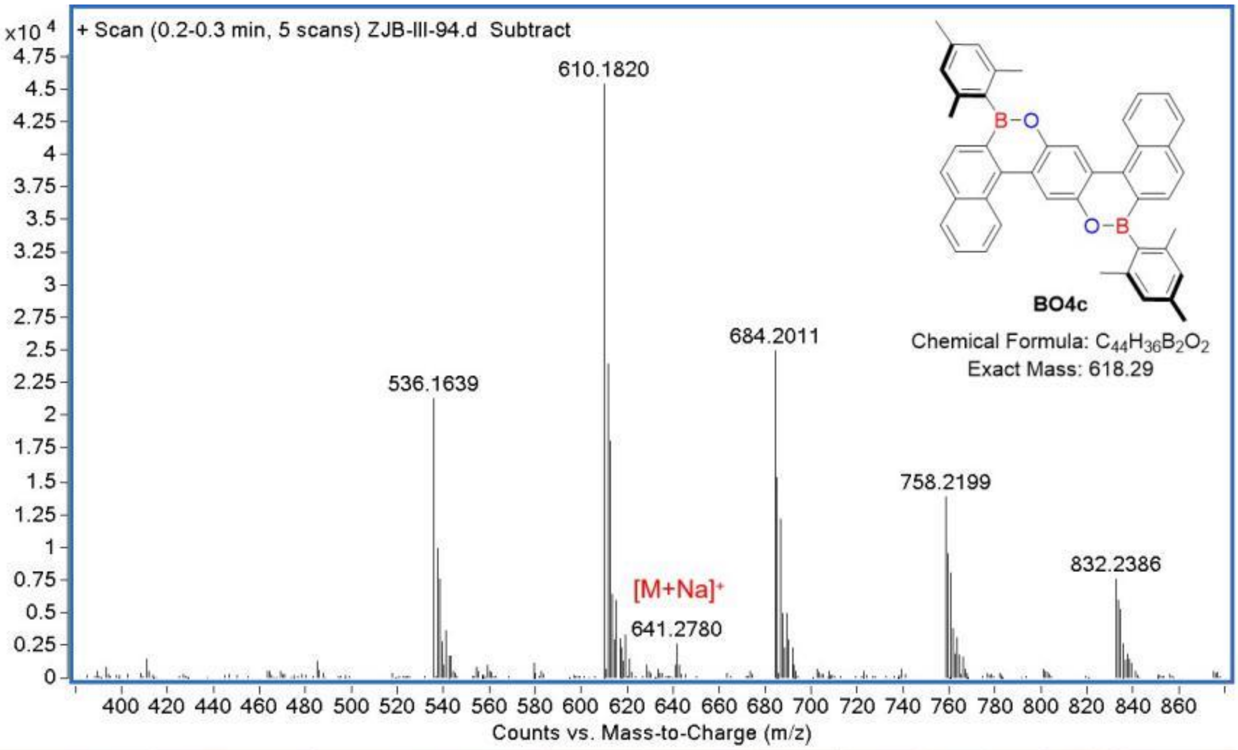

Formula (M)	Ion Formula	m/z	Calc m/z	Diff (ppm)
$C_{44}H_{36}[^{11}B]_2O_2$	$C_{44}H_{36}[^{11}B]_2NaO_2$	641.2780	641.2794	2.2

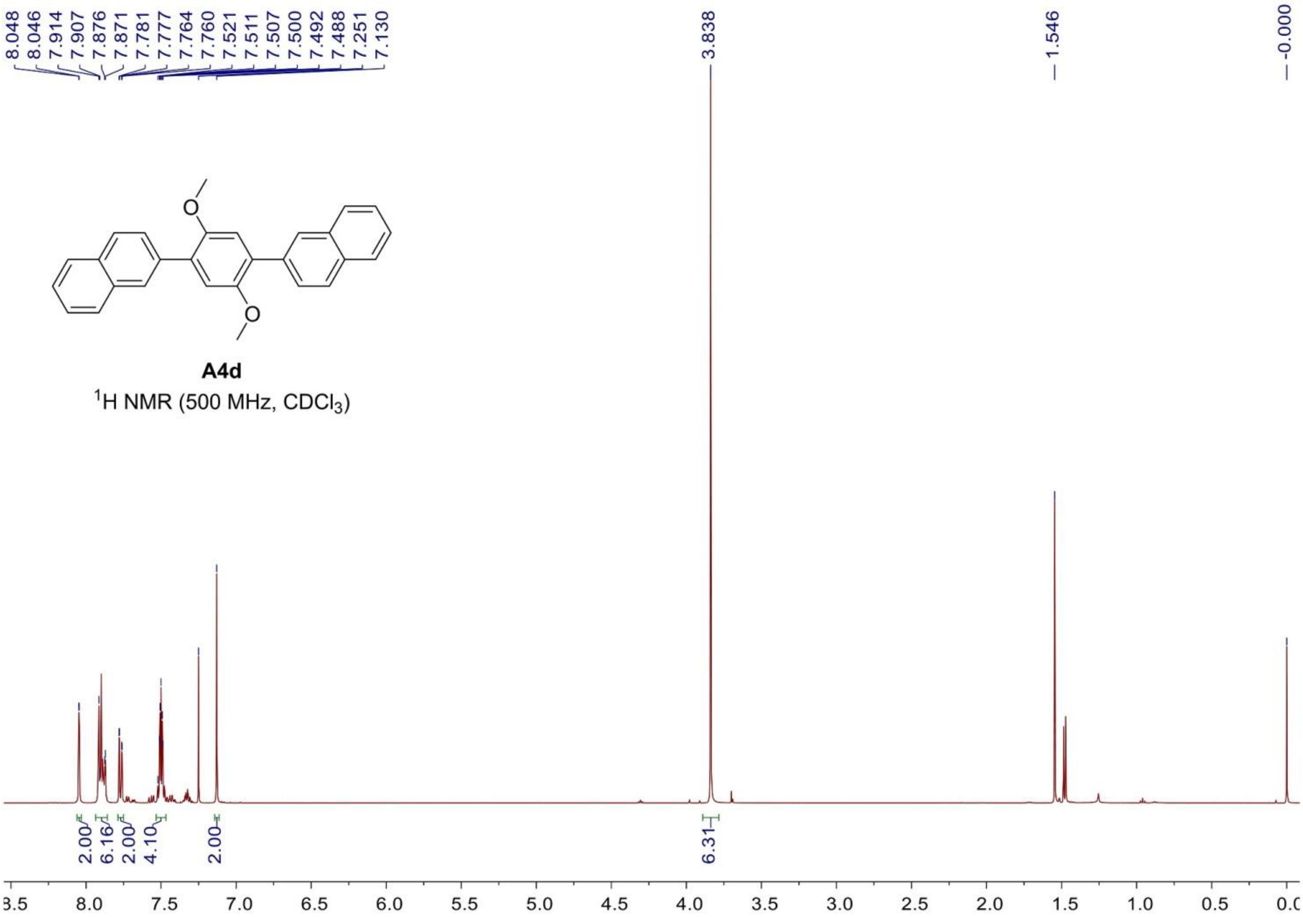
8.048
8.046
7.914
7.907
7.876
7.871
7.781
7.777
7.764
7.760
7.521
7.511
7.507
7.500
7.492
7.488
7.251
7.130
3.838
1.546
-0.000
A4d
^{1}H NMR (500 MHz, $CDCl_3$)
2.00
6.16
2.00
4.10
2.00
6.31
8.0
7.5
7.0
6.5
6.0
5.5
5.0
4.5
4.0
3.5
3.0
2.5
2.0
1.5
1.0
0.5

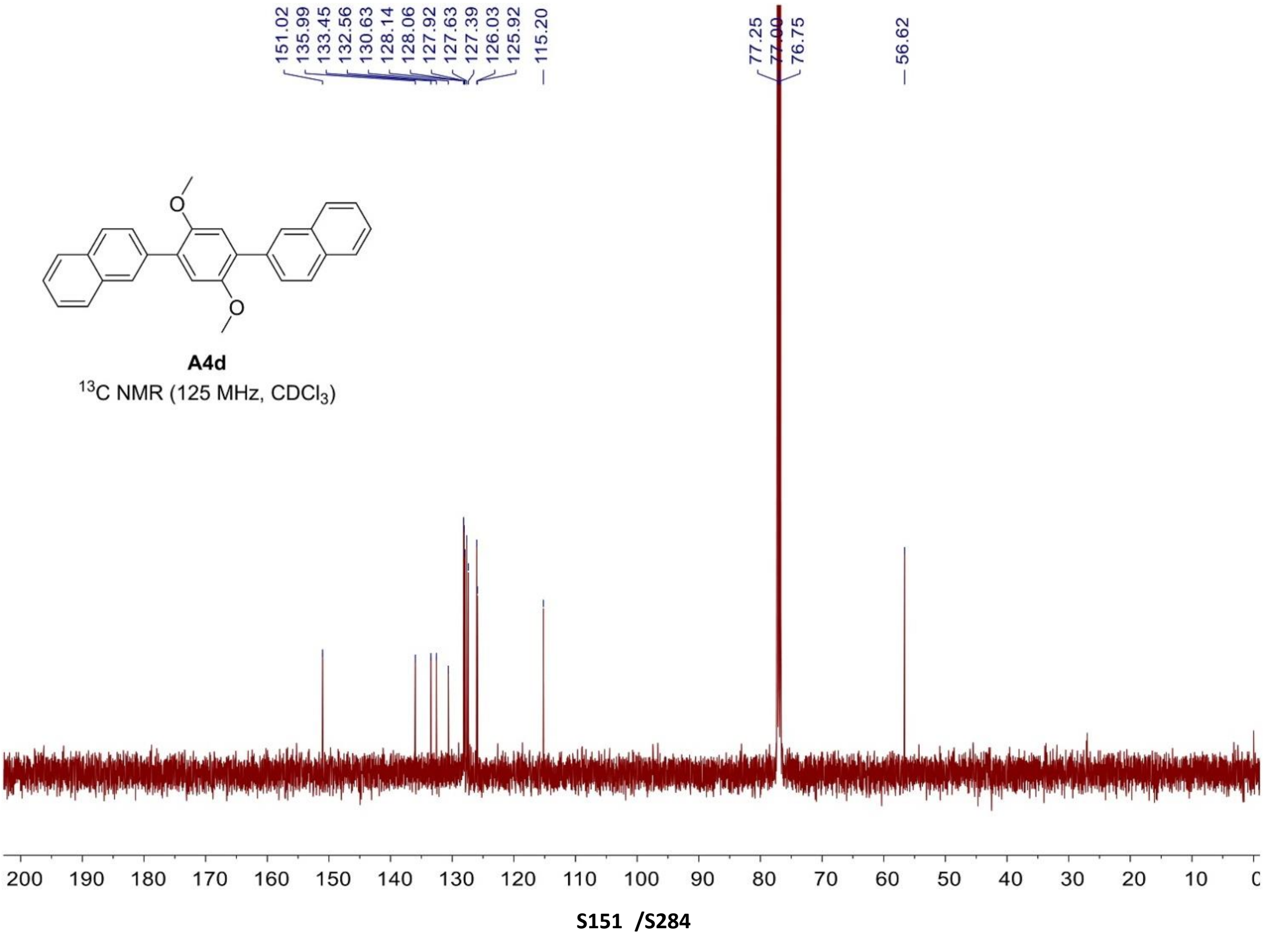
151.02
135.99
133.45
132.56
130.63
128.14
128.06
127.92
127.63
127.39
126.03
125.92
115.20
77.25
77.00
76.75
56.62
A4d
^{13}C NMR (125 MHz, $CDCl_3$)
200
190
180
170
160
150
140
130
120
110
100
90
80
70
60
50
40
30
20
10

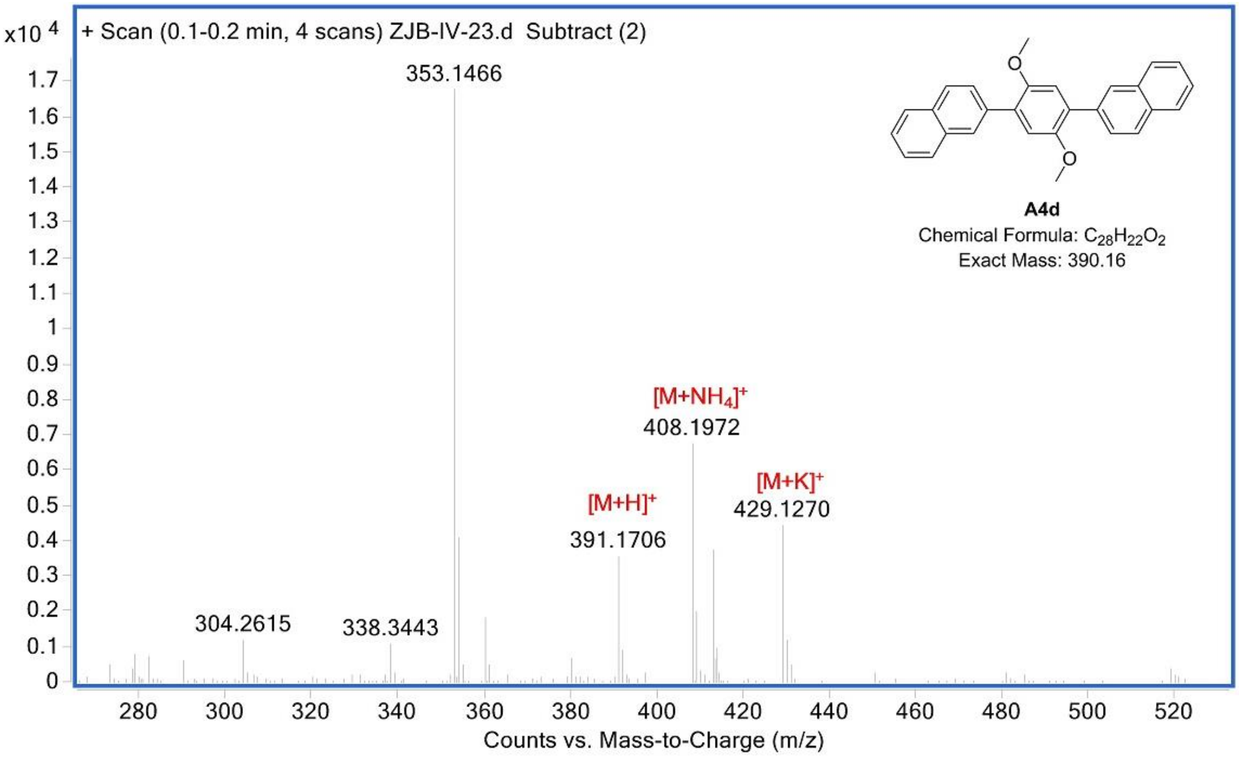

Formula (M)	Ion Formula	m/z	Calc m/z	Diff (ppm)	DBE
$C_{28}H_{22}O_2$	$C_{28}H_{26}NO_2$	408.1972	408.1958	-3.57	18

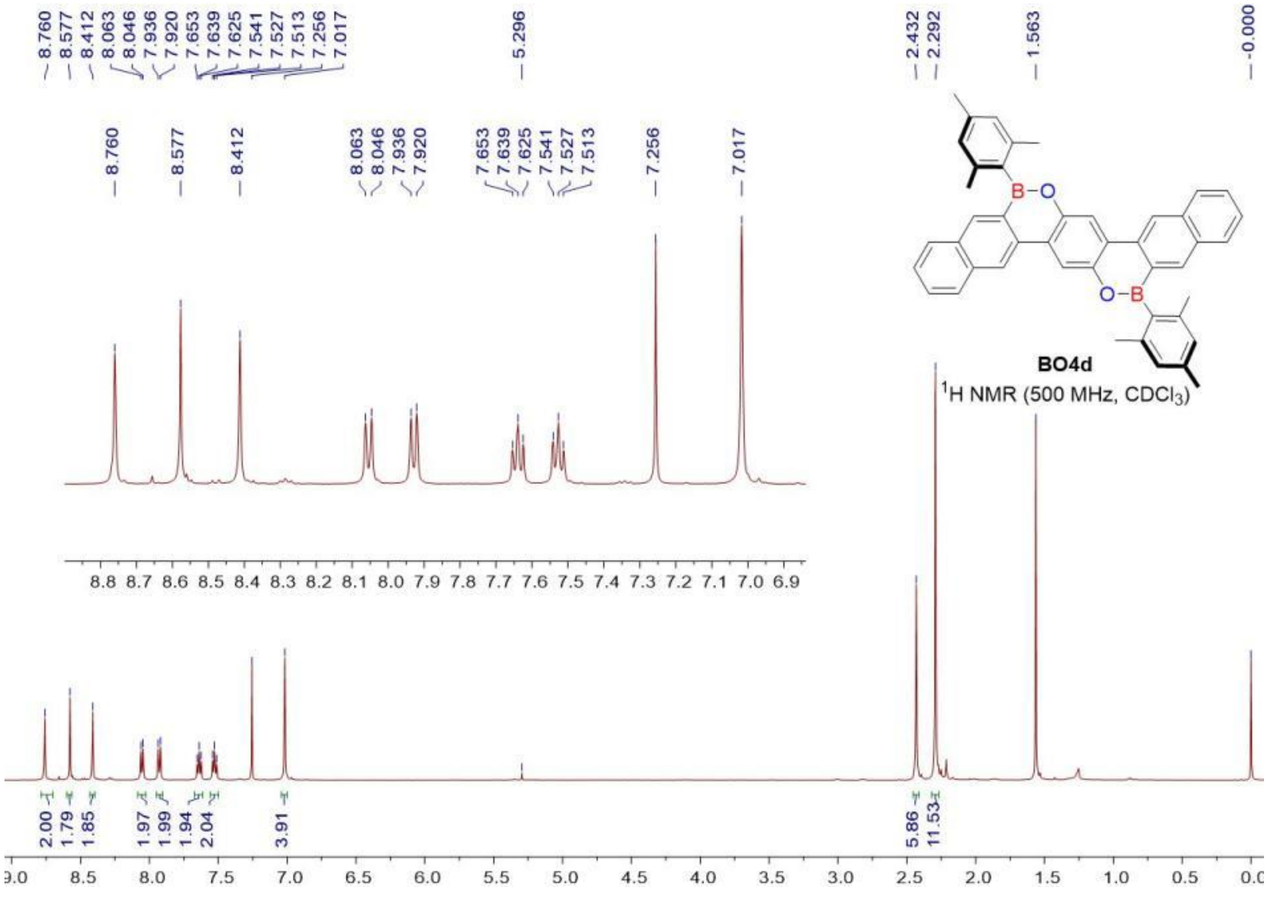

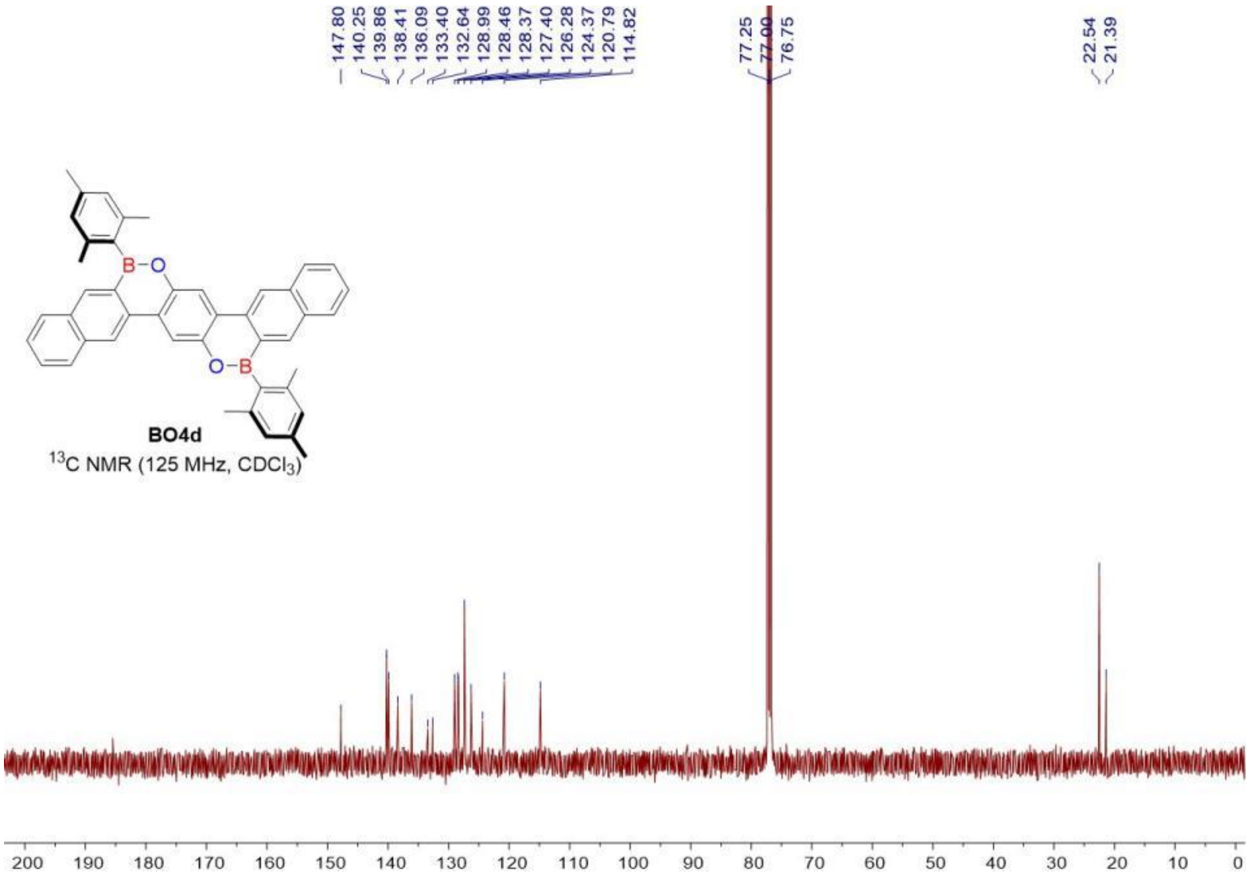
147.80
140.25
139.86
138.41
136.09
133.40
132.64
128.99
128.46
128.37
127.40
126.28
124.37
120.79
114.82
77.25
77.00
76.75
22.54
21.39
B
O
BO4d
^{13}C NMR (125 MHz, $CDCl_3$)
200 190 180 170 160 150 140 130 120 110 100 90 80 70 60 50 40 30 20 10 0

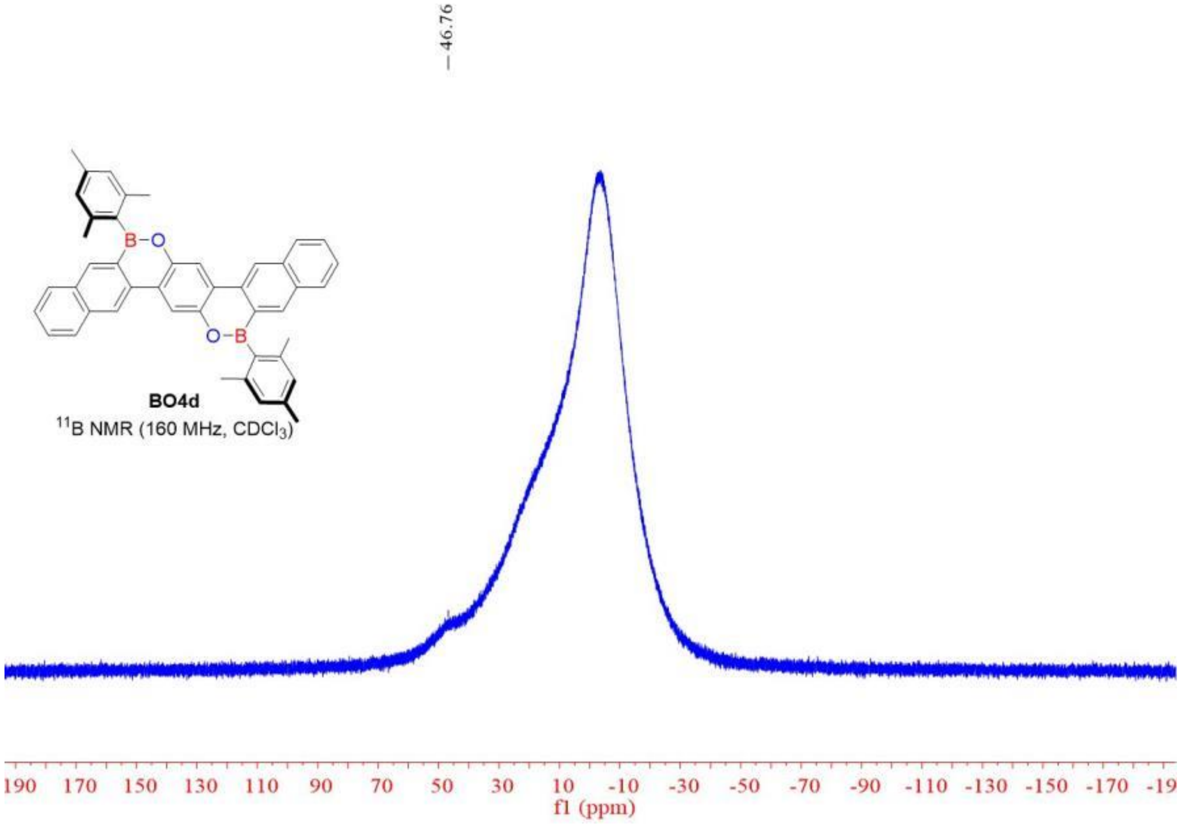
46.76
B
O
BO4d
^{11}B NMR (160 MHz, $CDCl_3$)
190 170 150 130 110 90 70 50 30 10 -10 -30 -50 -70 -90 -110 -130 -150 -170 -19
f1 (ppm)

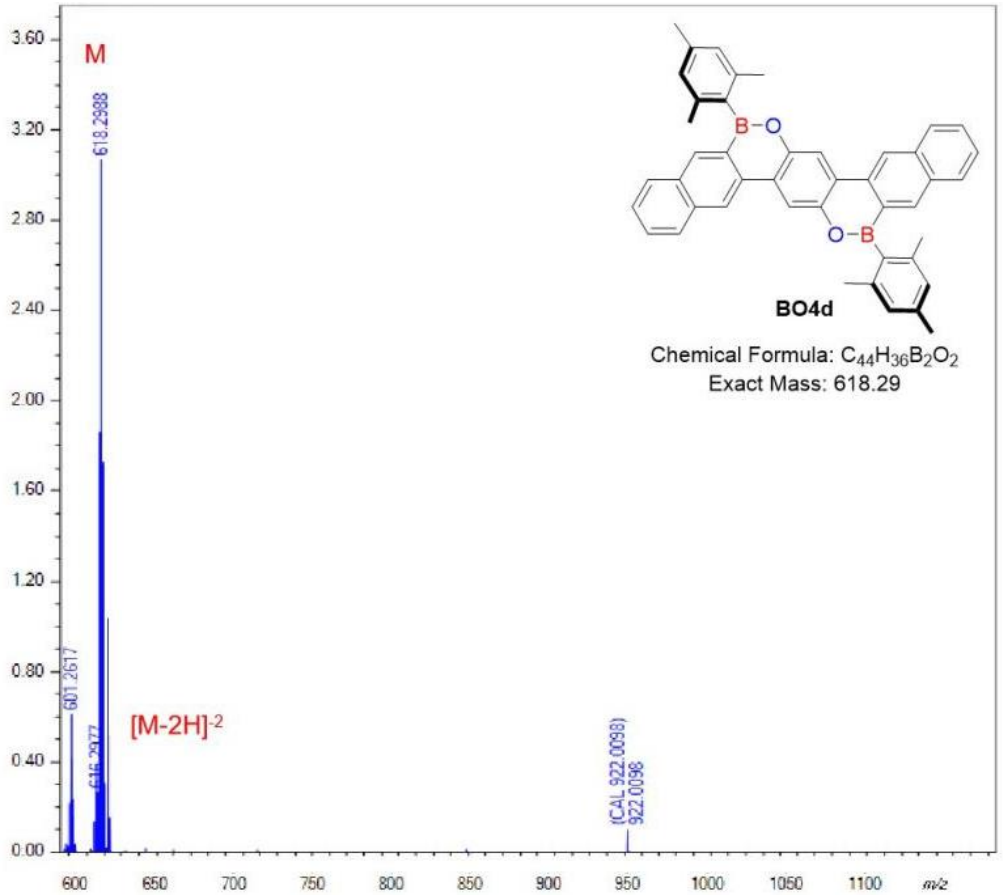

M
618.2988
601.2617
616.2977
[M-2H]-2
(CAL 922.0098)
922.0098
BO4d
Chemical Formula: C44H36B2O2
Exact Mass: 618.29
m/z

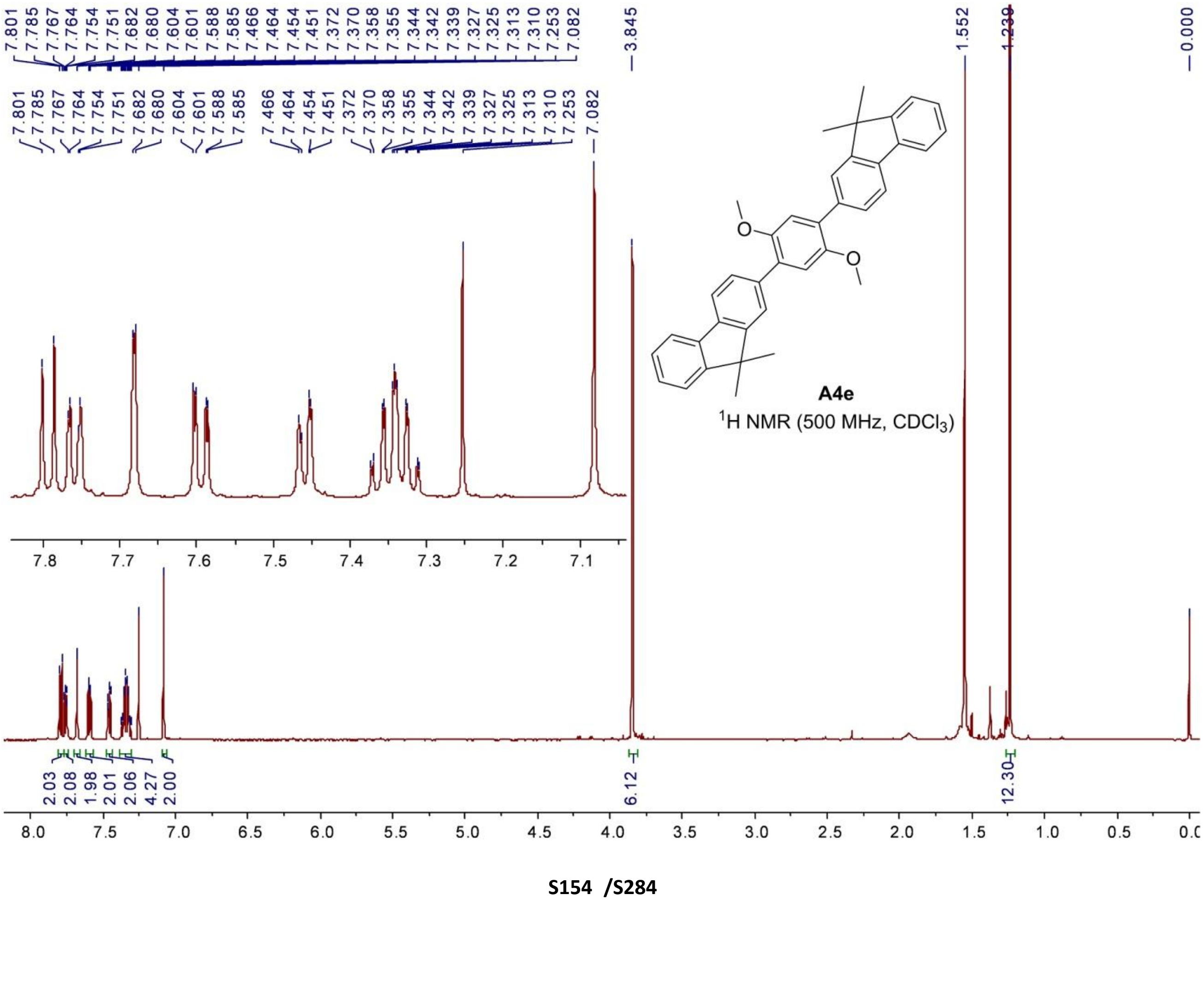

7.801 7.785 7.767 7.764 7.754 7.751 7.682 7.680 7.604 7.601 7.588 7.585 7.466 7.464 7.454 7.451 7.372 7.370 7.358 7.355 7.344 7.342 7.339 7.327 7.325 7.313 7.310 7.253 7.082
3.845
1.552
1.239
0.000
A4e
1H NMR (500 MHz, CDCl3)
2.03 2.08 1.98 2.01 2.06 4.27 2.00
6.12
12.30

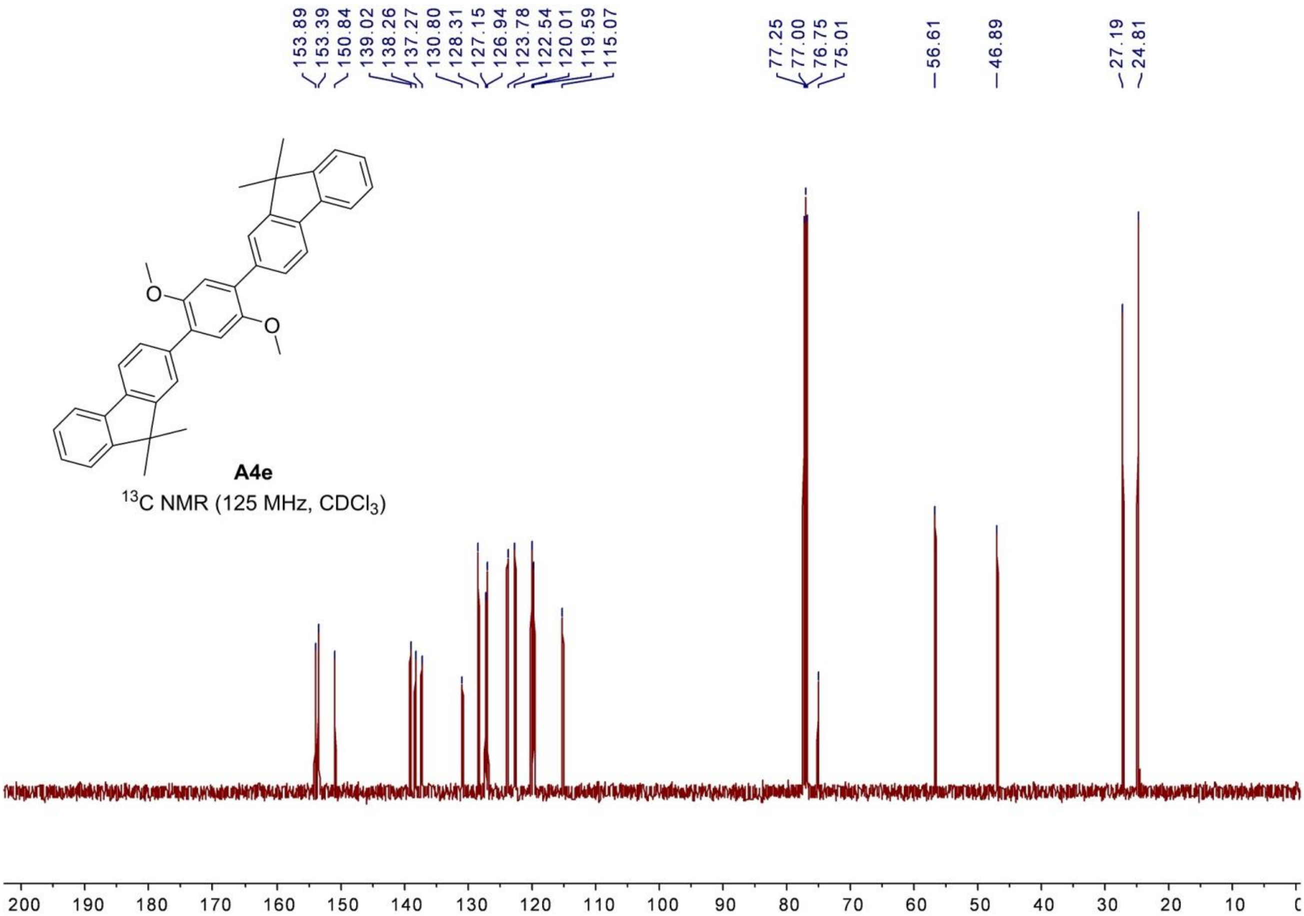

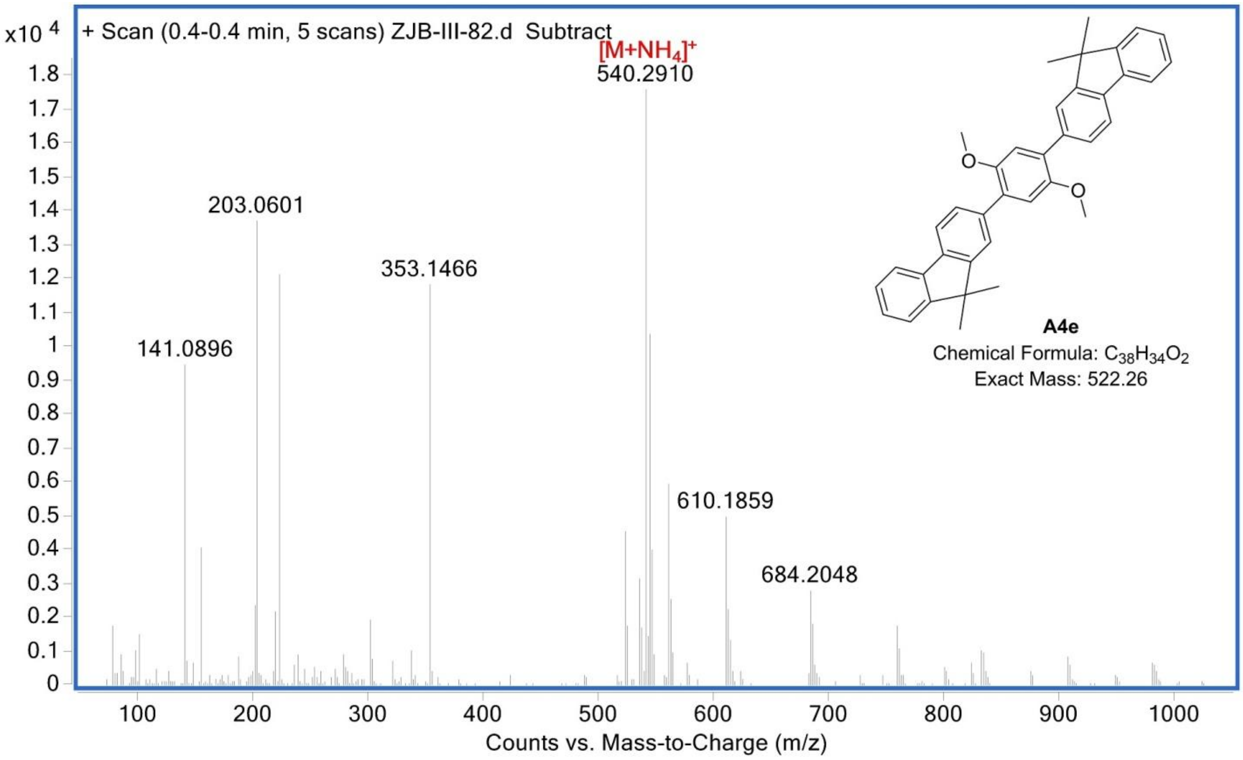

Formula (M)	Ion Formula	m/z	Calc m/z	Diff (ppm)	DBE
$C_{38}H_{34}O_2$	$C_{38}H_{38}NO_2$	540.2910	540.2897	-2.48	22

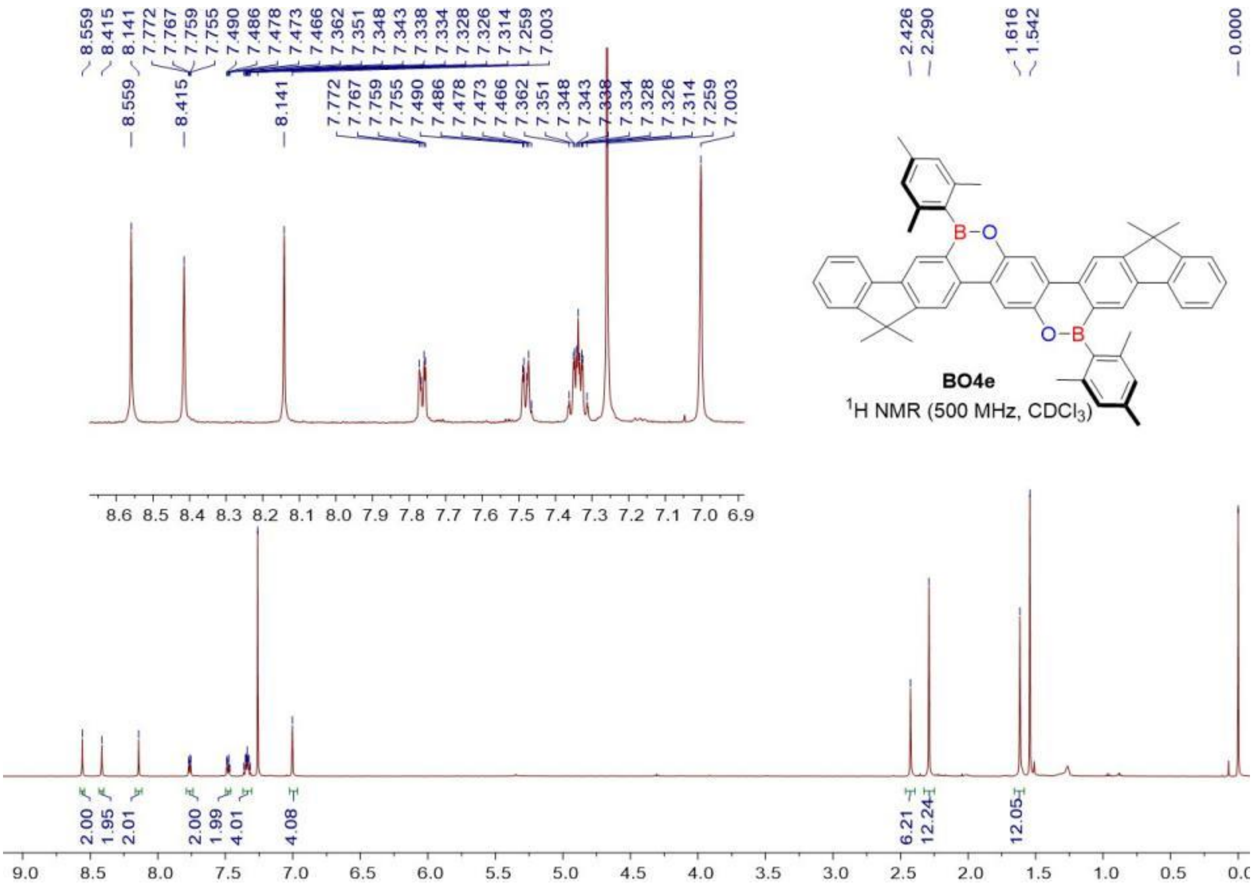
BO4e
^{1}H NMR (500 MHz, CDCl$_3$)

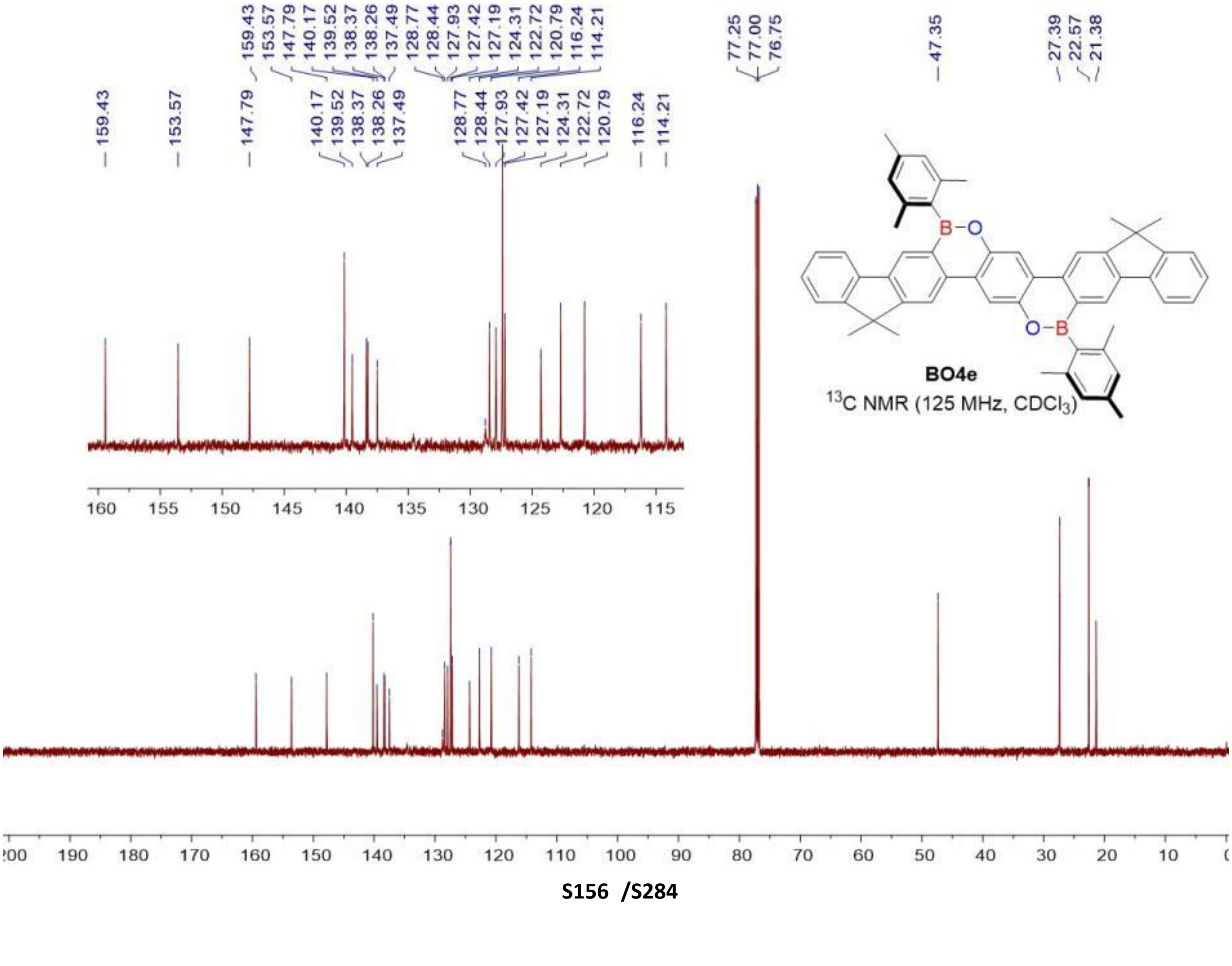
BO4e
^{13}C NMR (125 MHz, CDCl$_3$)

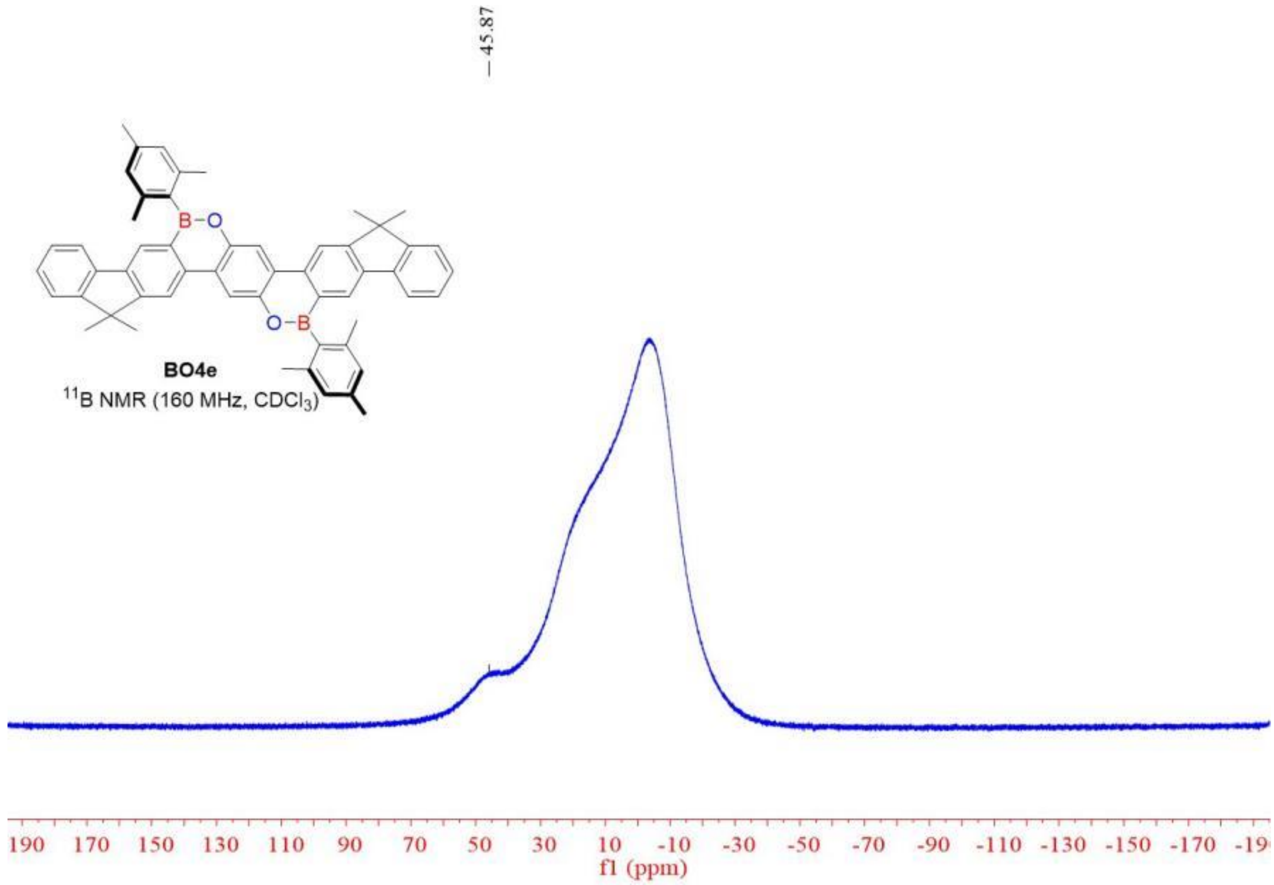

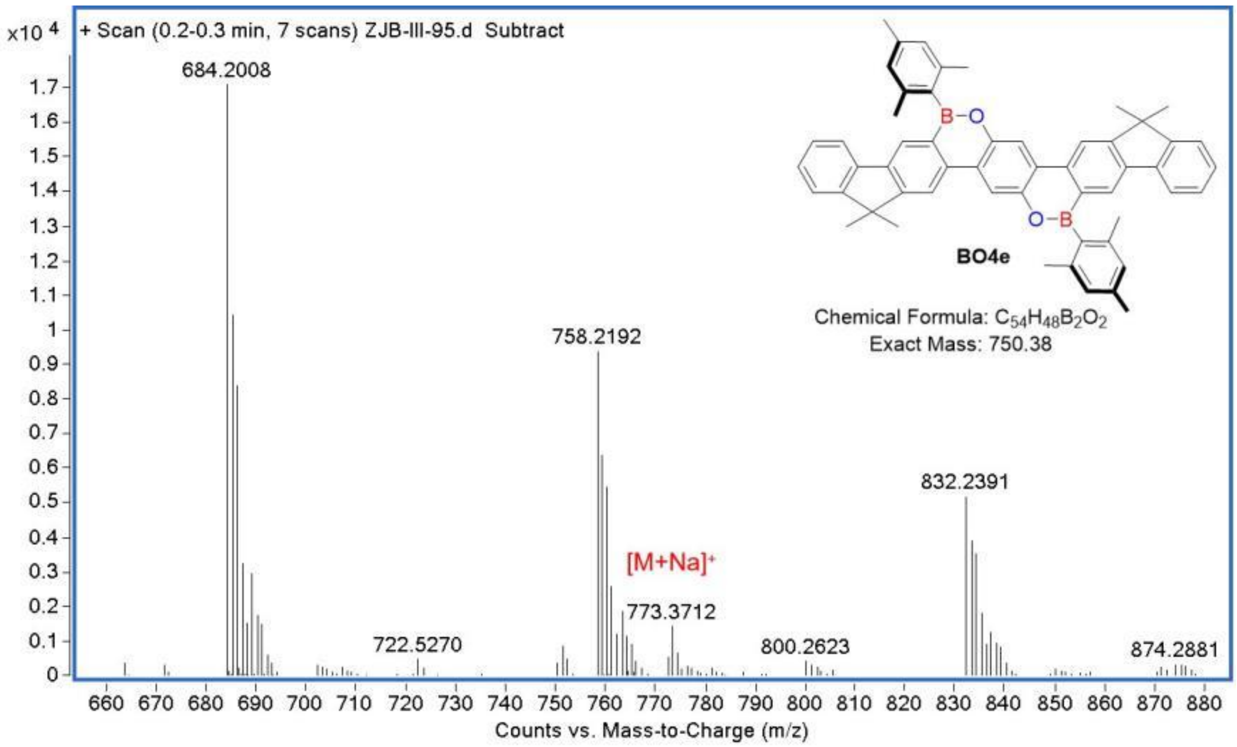

Formula (M)	Ion Formula	m/z	Calc m/z	Diff (ppm)
$C_{54}H_{48}[^{11}B]_2O_2$	$C_{54}H_{48}[^{11}B]_2NaO_2$	773.3712	773.3733	2.75

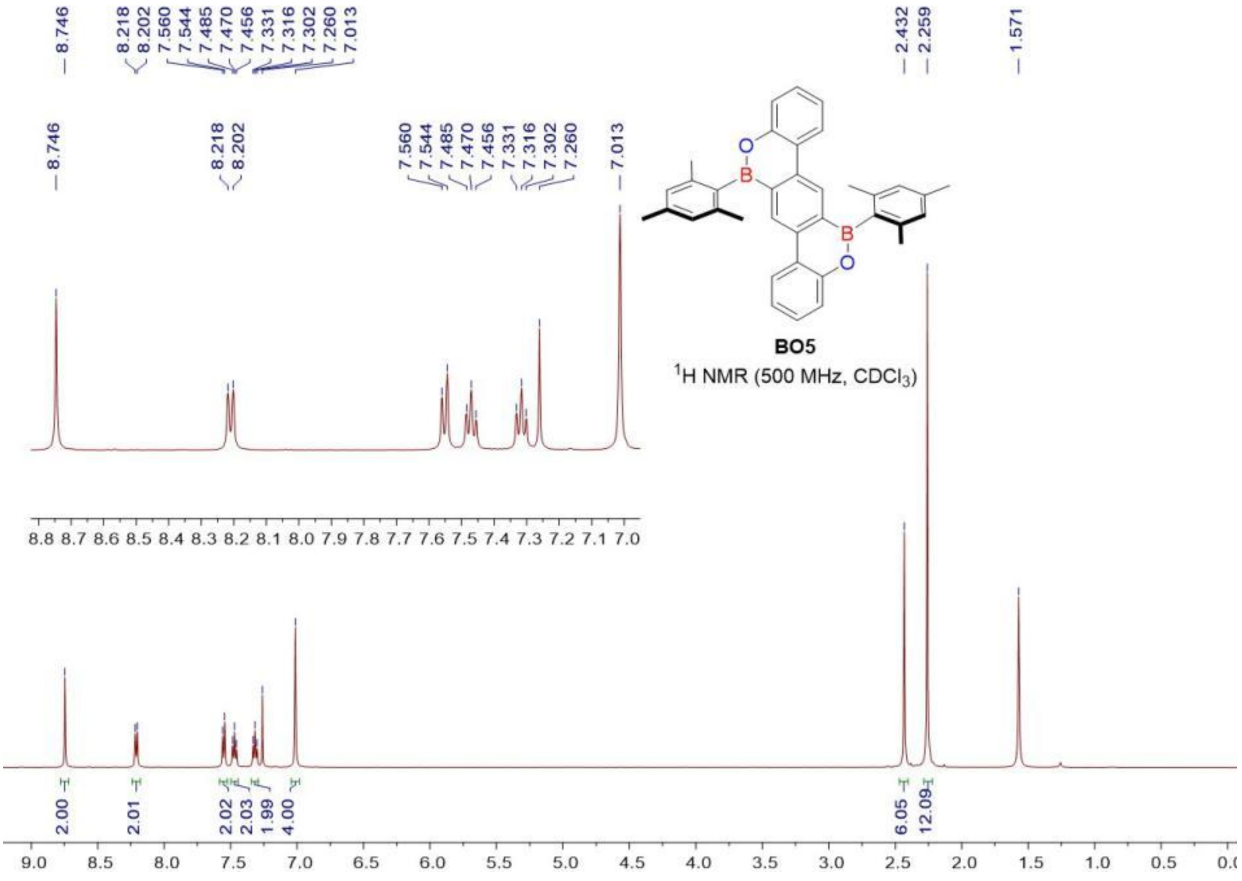
8.746
8.218
8.202
7.560
7.544
7.485
7.470
7.456
7.331
7.316
7.302
7.260
7.013
2.432
2.259
1.571
BO5
^{1}H NMR (500 MHz, CDCl$_3$)
8.8 8.7 8.6 8.5 8.4 8.3 8.2 8.1 8.0 7.9 7.8 7.7 7.6 7.5 7.4 7.3 7.2 7.1 7.0
2.00
2.01
2.02
2.03
1.99
4.00
6.05
12.09
9.0 8.5 8.0 7.5 7.0 6.5 6.0 5.5 5.0 4.5 4.0 3.5 3.0 2.5 2.0 1.5 1.0 0.5 0.0

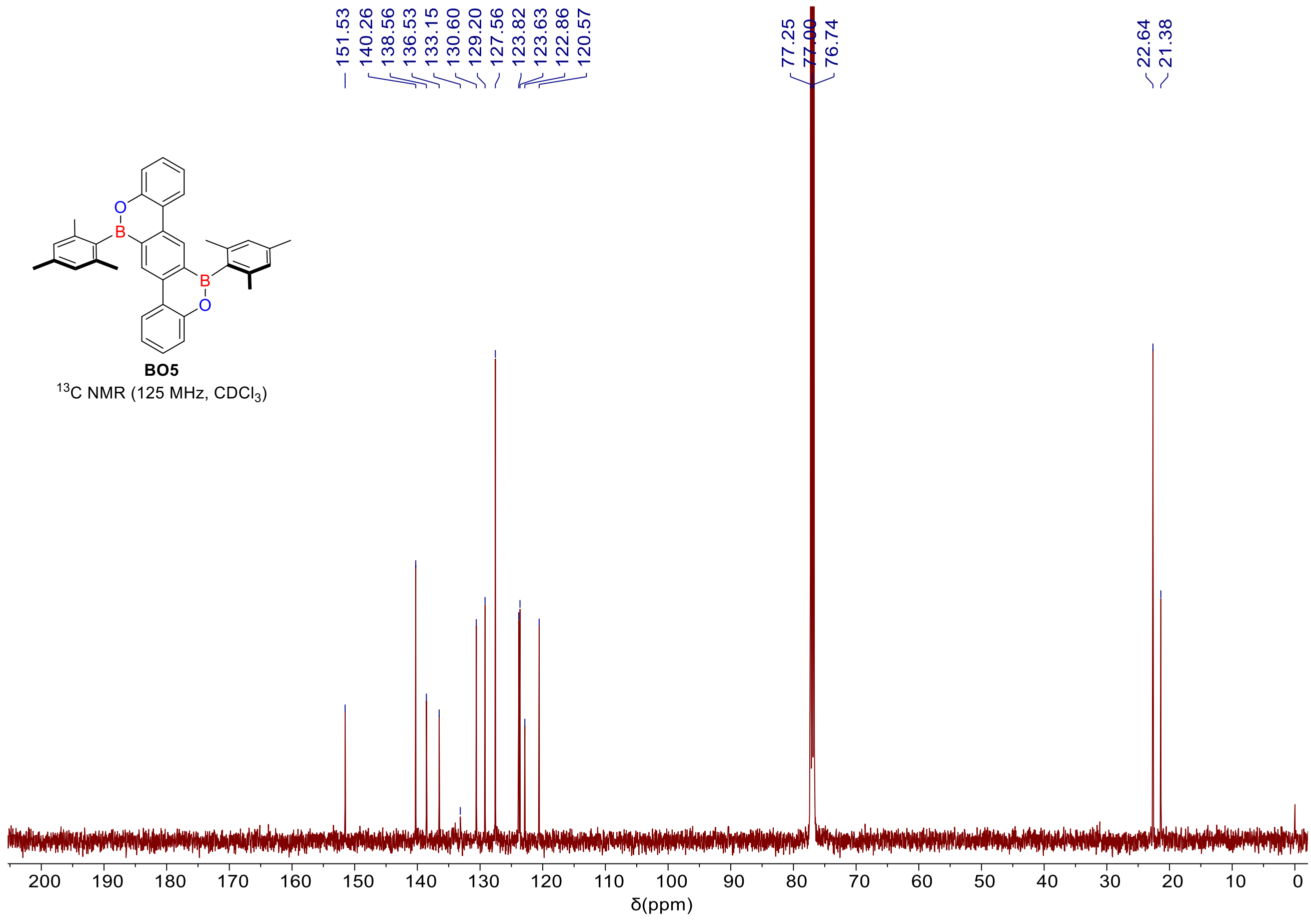
151.53
140.26
138.56
136.53
133.15
130.60
129.20
127.56
123.82
123.63
122.86
120.57
77.25
77.00
76.74
22.64
21.38
BO5
^{13}C NMR (125 MHz, CDCl$_3$)
200 190 180 170 160 150 140 130 120 110 100 90 80 70 60 50 40 30 20 10 0
δ(ppm)

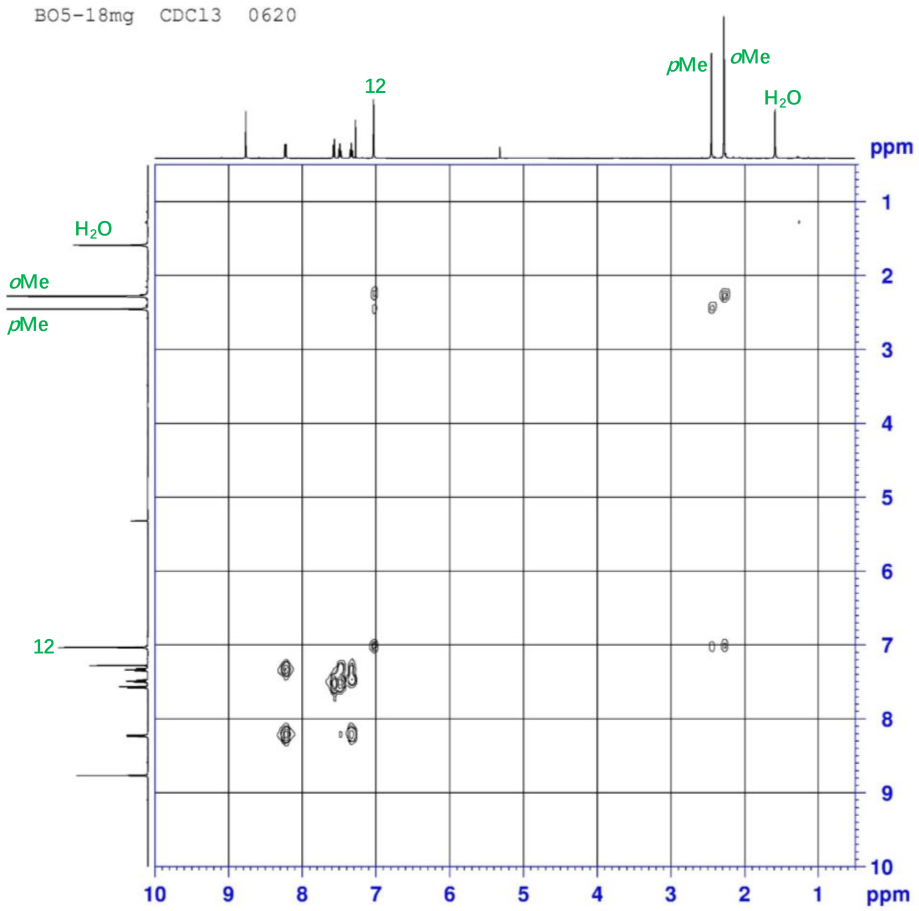

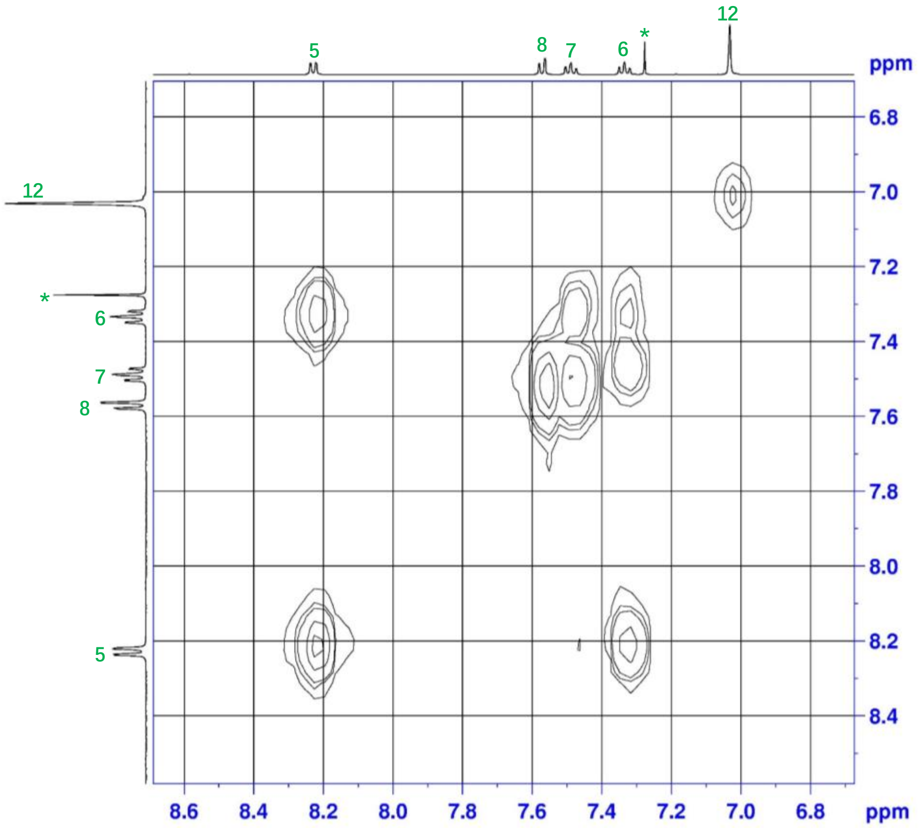

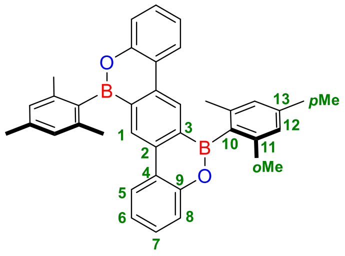

BO5

1H-^{1}H COSY (500 MHz, $CDCl_3$)

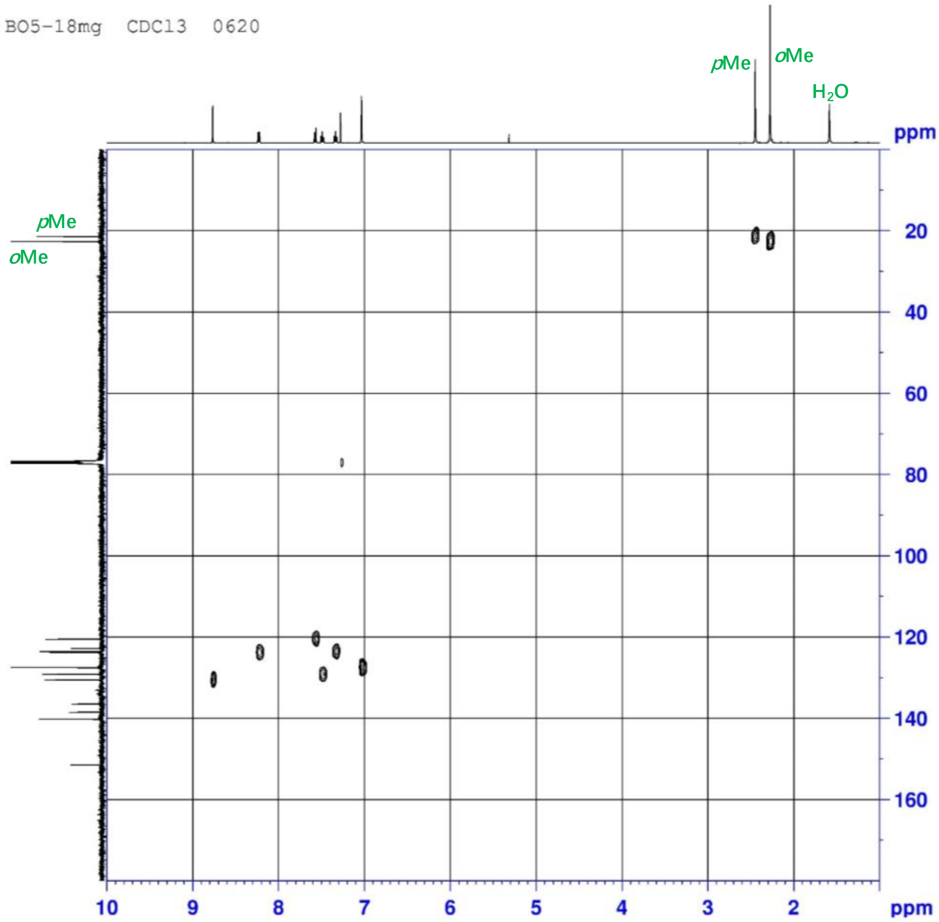

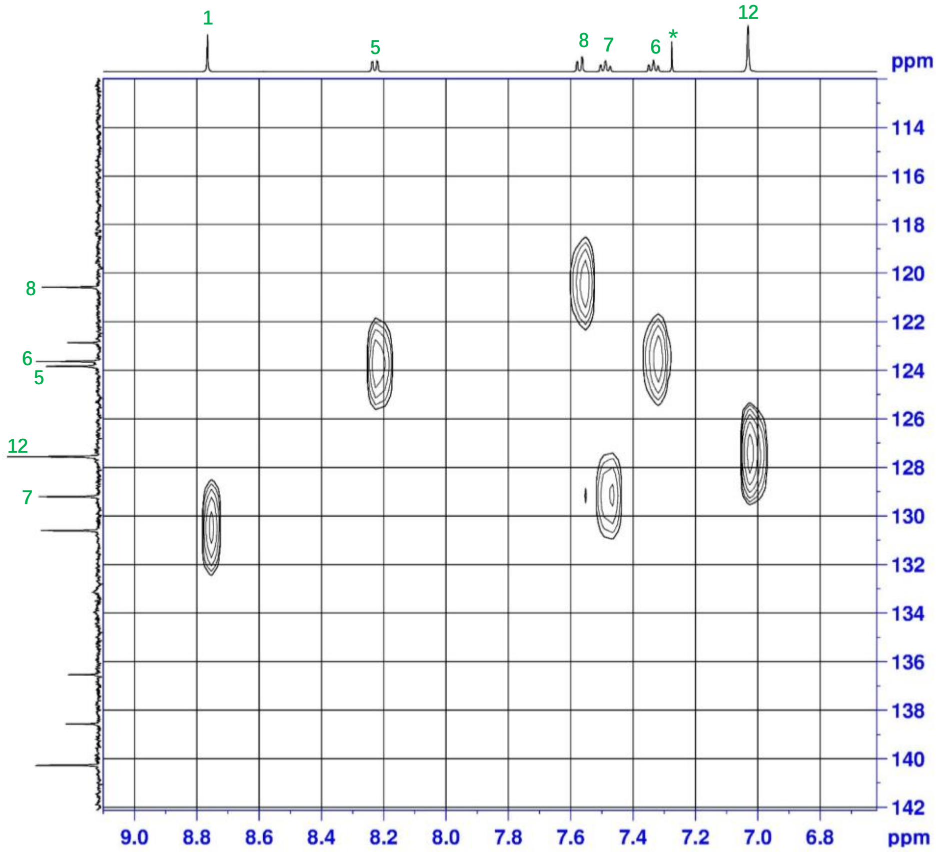

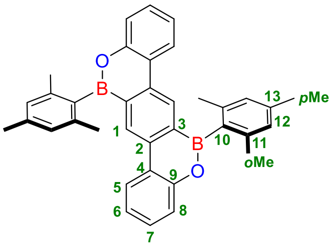

BO5

HSQC (500 MHz, $CDCl_3$)

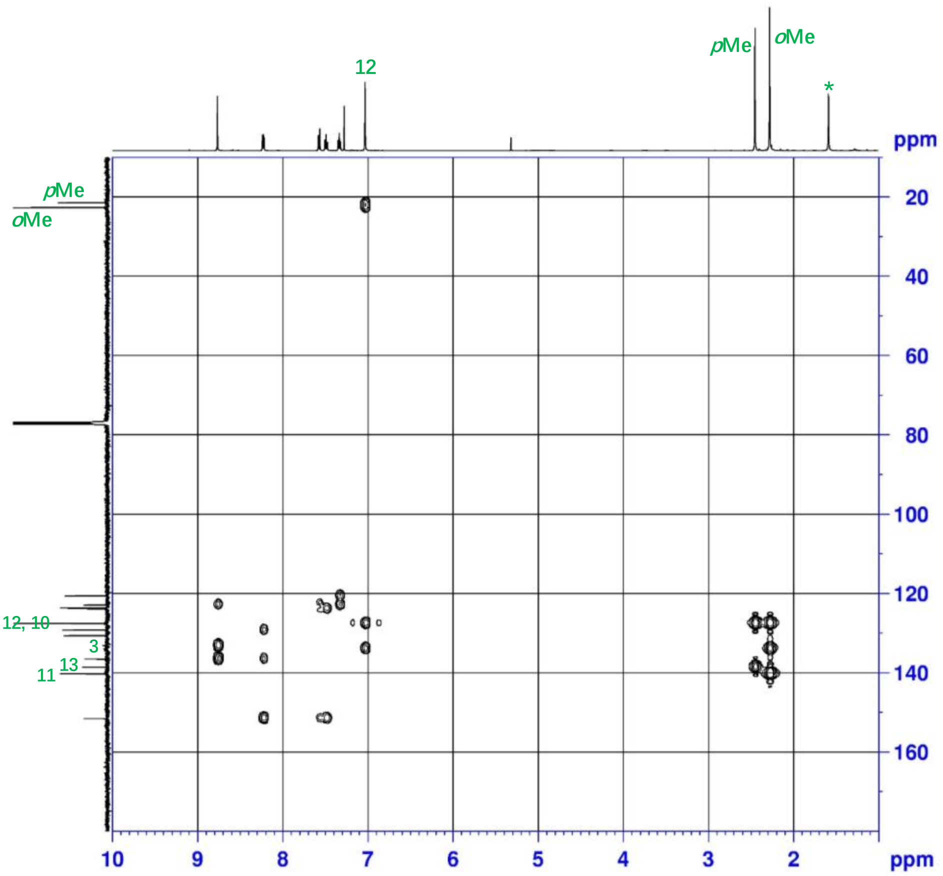

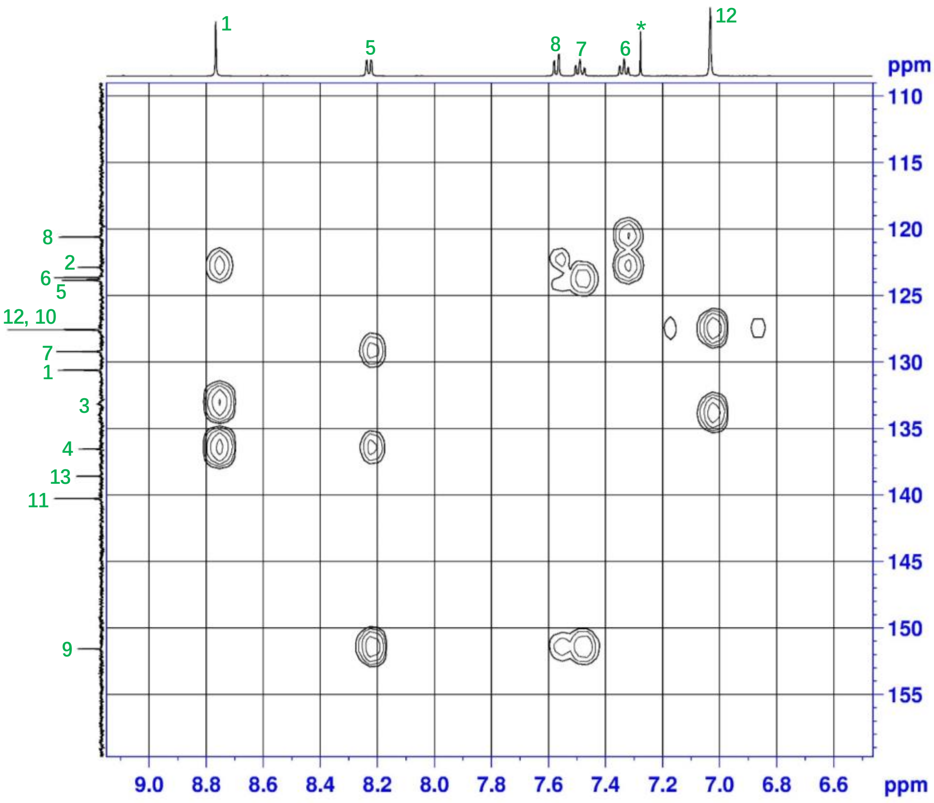

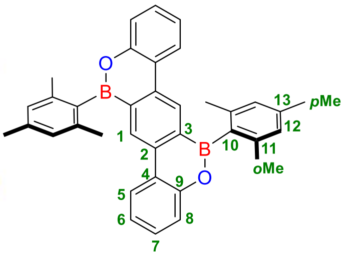

BO5

HMBC (500 MHz, $CDCl_3$)

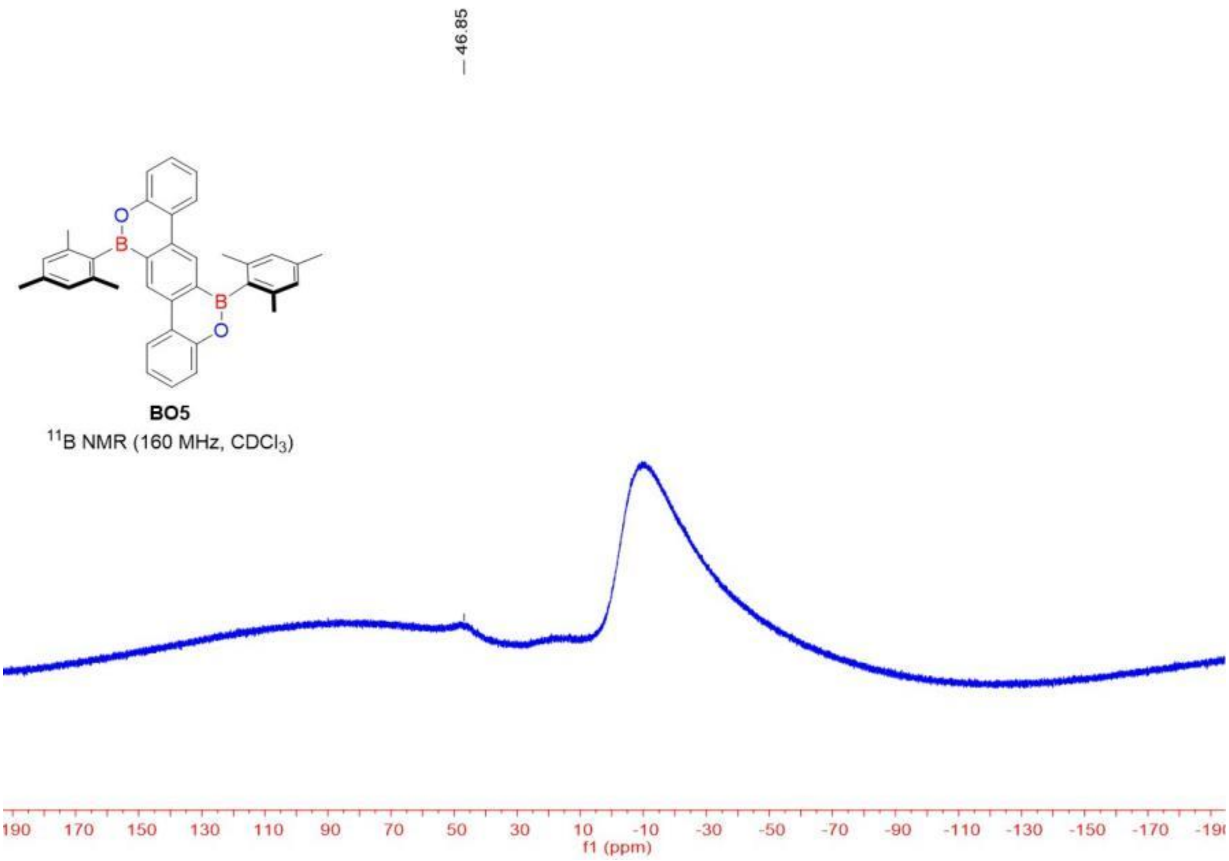

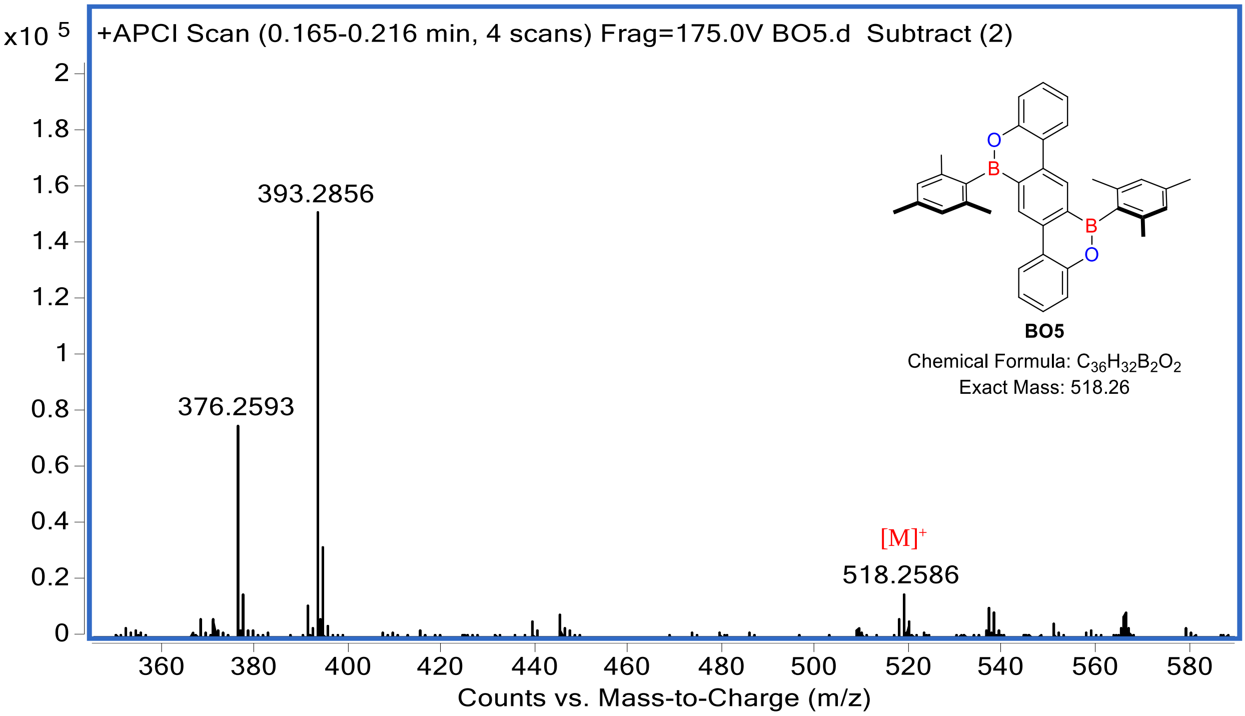

Formula (M)	Ion Formula	m/z	Calc m/z	Diff (ppm)
$C_{36}H_{32}[^{11}B]_2O_2$	$C_{36}H_{32}[^{11}B]_2O_2$	518.2586	518.2583	-0.59

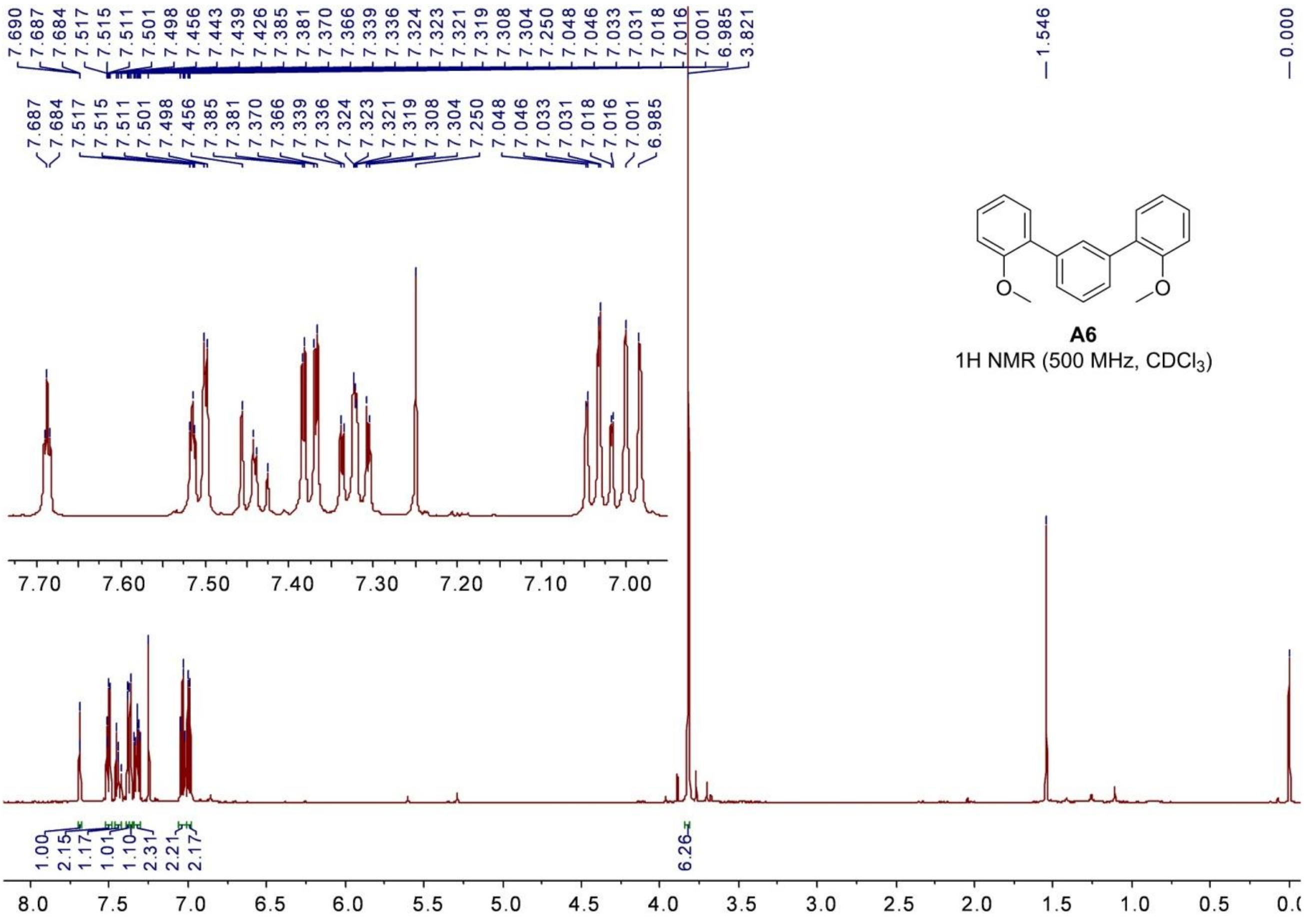
7.690 7.687 7.684 7.517 7.515 7.511 7.501 7.498 7.456 7.443 7.439 7.426 7.385 7.381 7.370 7.366 7.339 7.336 7.324 7.323 7.321 7.319 7.308 7.304 7.250 7.048 7.046 7.033 7.031 7.018 7.016 7.001 6.985 3.821
1.546
0.000
A6
1H NMR (500 MHz, CDCl3)
1.00 2.15 1.17 1.01 1.10 2.31 2.21 2.17
6.26

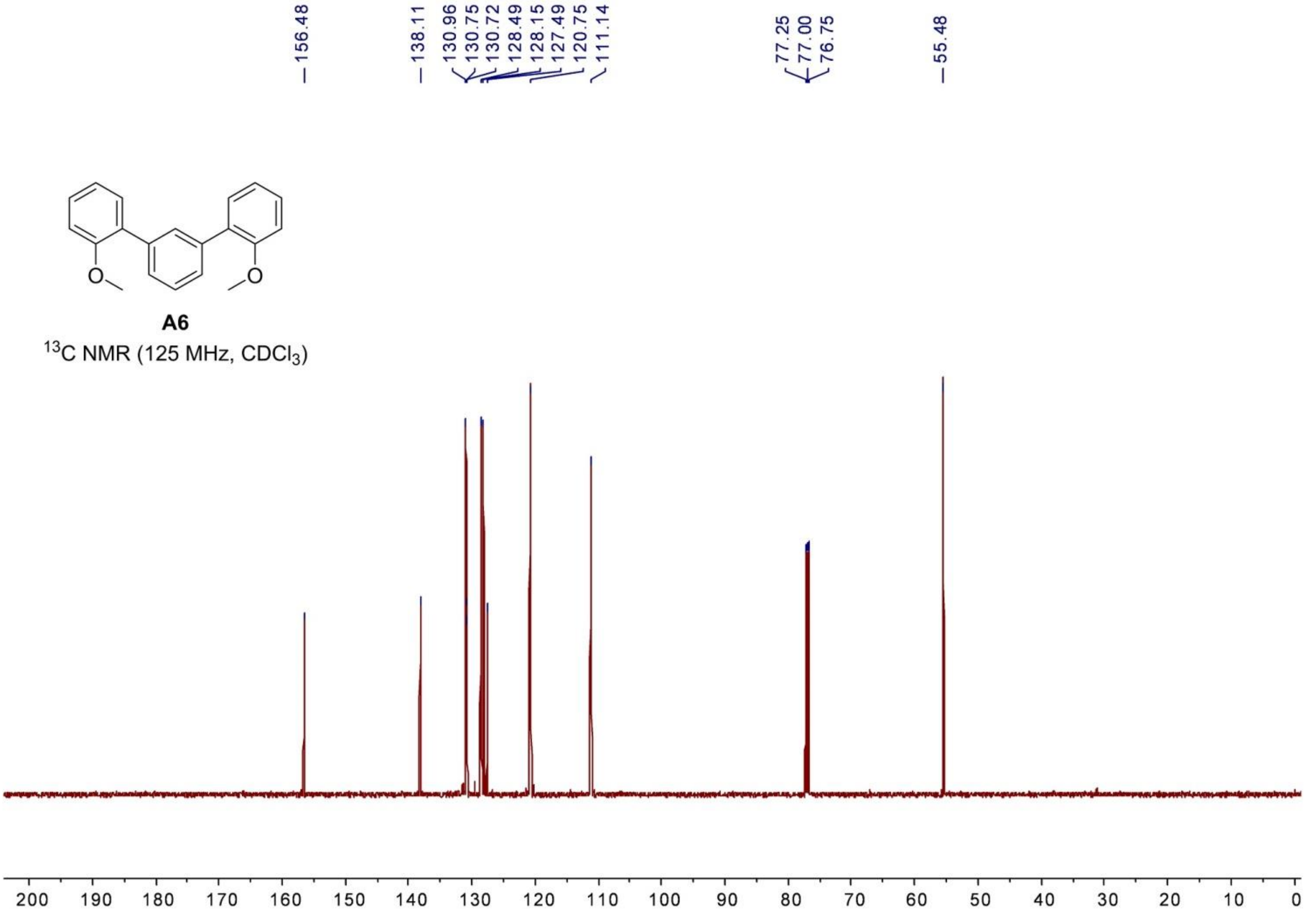
156.48
138.11 130.96 130.75 130.72 128.49 128.15 127.49 120.75 111.14
77.25 77.00 76.75
55.48
A6
13C NMR (125 MHz, CDCl3)

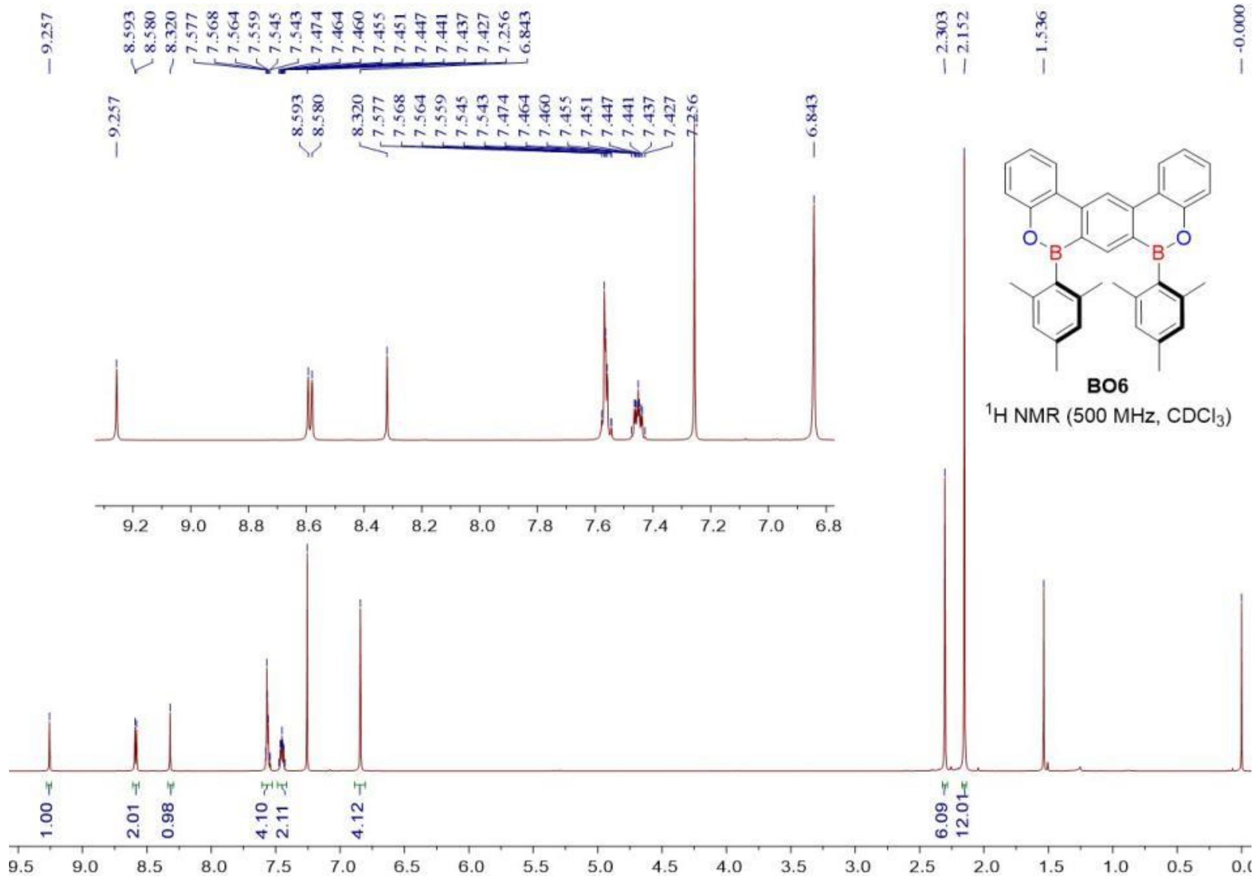
BO6
^{1}H NMR (500 MHz, $CDCl_3$)
9.257, 8.593, 8.580, 8.320, 7.577, 7.568, 7.564, 7.559, 7.545, 7.543, 7.474, 7.464, 7.460, 7.455, 7.451, 7.447, 7.441, 7.437, 7.427, 7.256, 6.843
2.303, 2.152, 1.536, -0.000
1.00, 2.01, 0.98, 4.10, 2.11, 4.12, 6.09, 12.01

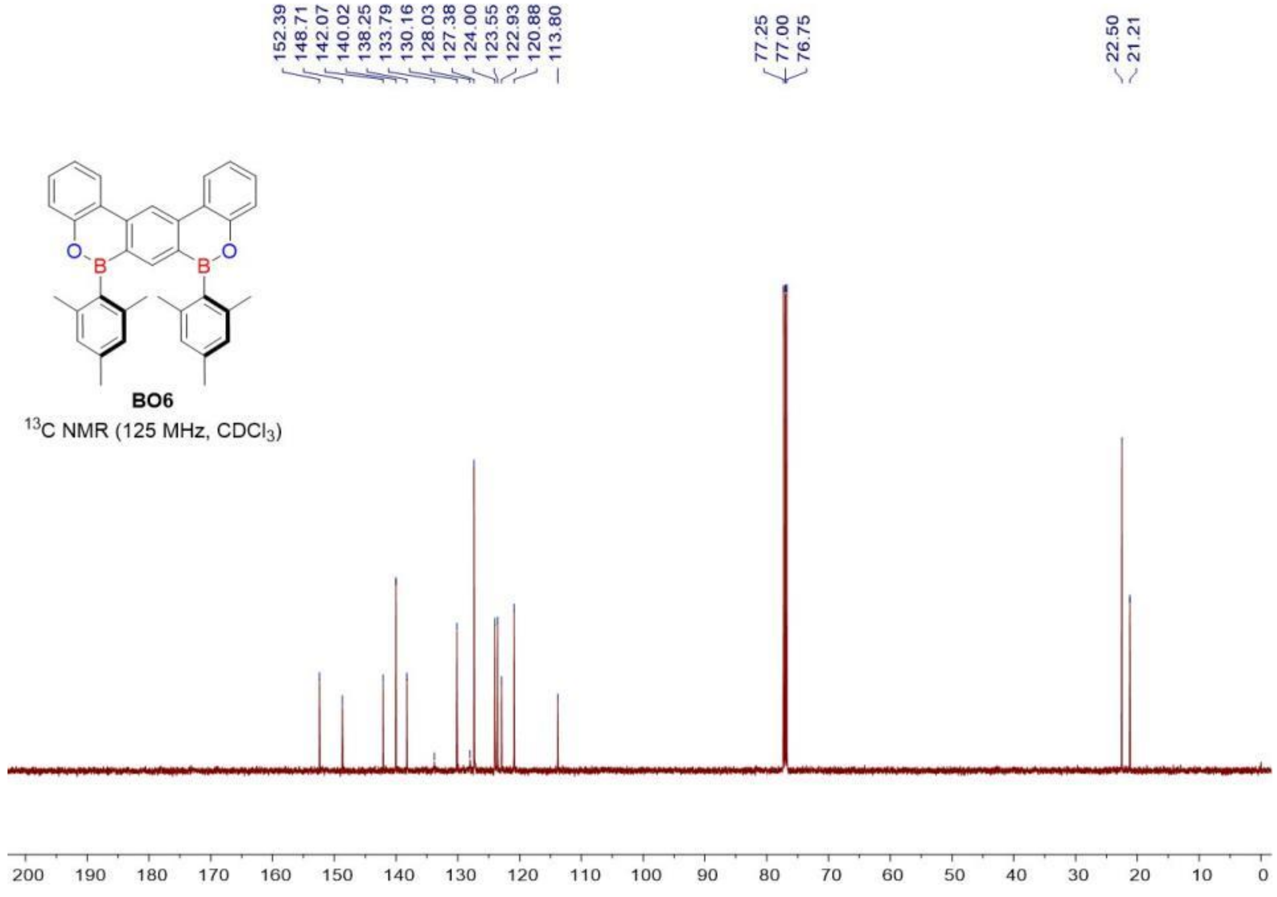
BO6
^{13}C NMR (125 MHz, $CDCl_3$)
152.39, 148.71, 142.07, 140.02, 138.25, 133.79, 130.16, 128.03, 127.38, 124.00, 123.55, 122.93, 120.88, 113.80
77.25, 77.00, 76.75
22.50, 21.21

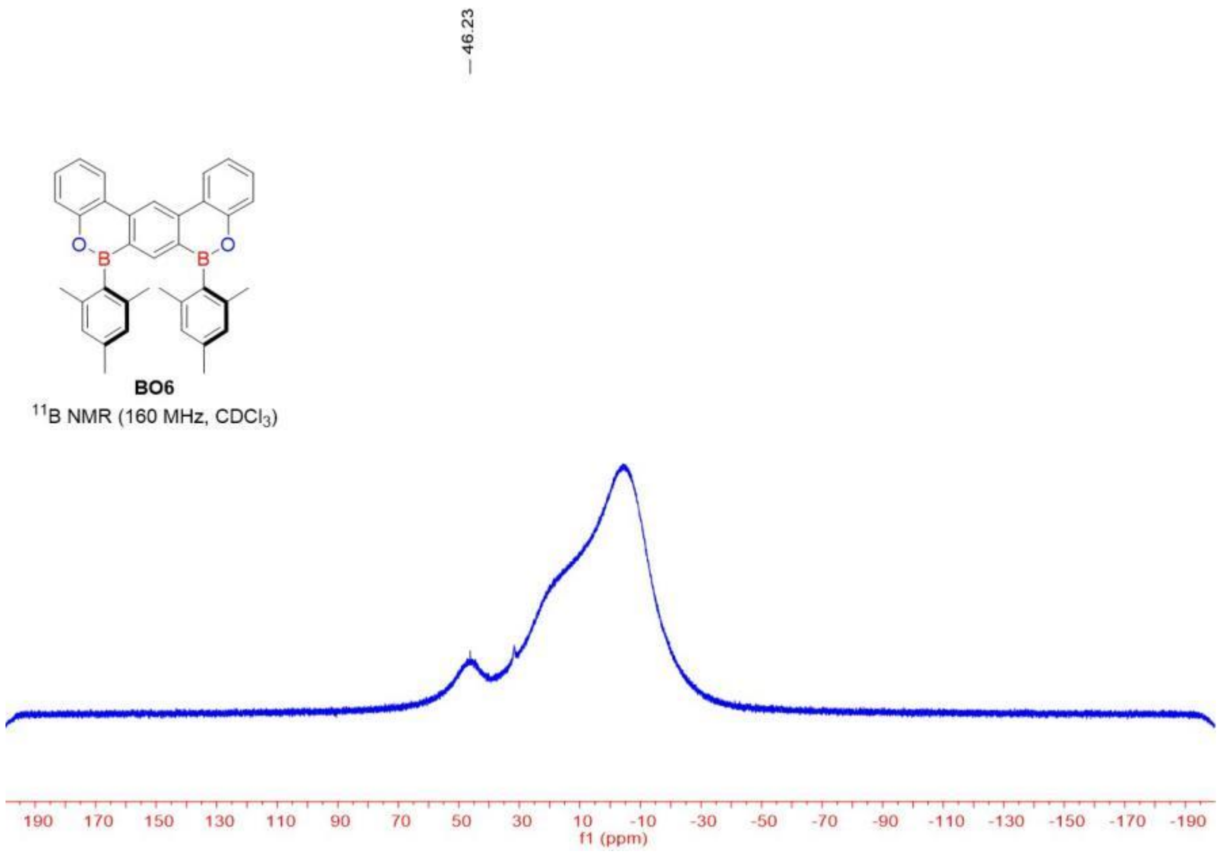

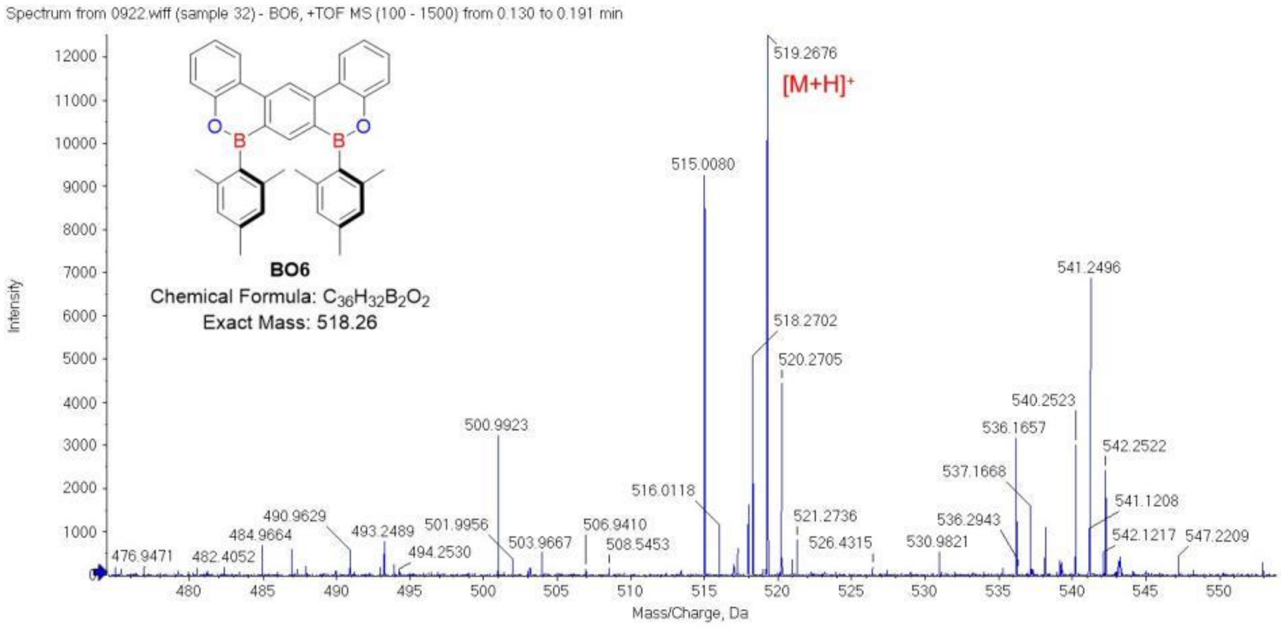

Formula (M)	Ion Formula	m/z	Calc m/z	Diff (ppm)
$C_{36}H_{32}[^{11}B]_2O_2$	$C_{36}H_{33}[^{11}B]_2O_2$	519.2676	519.2661	-3.0

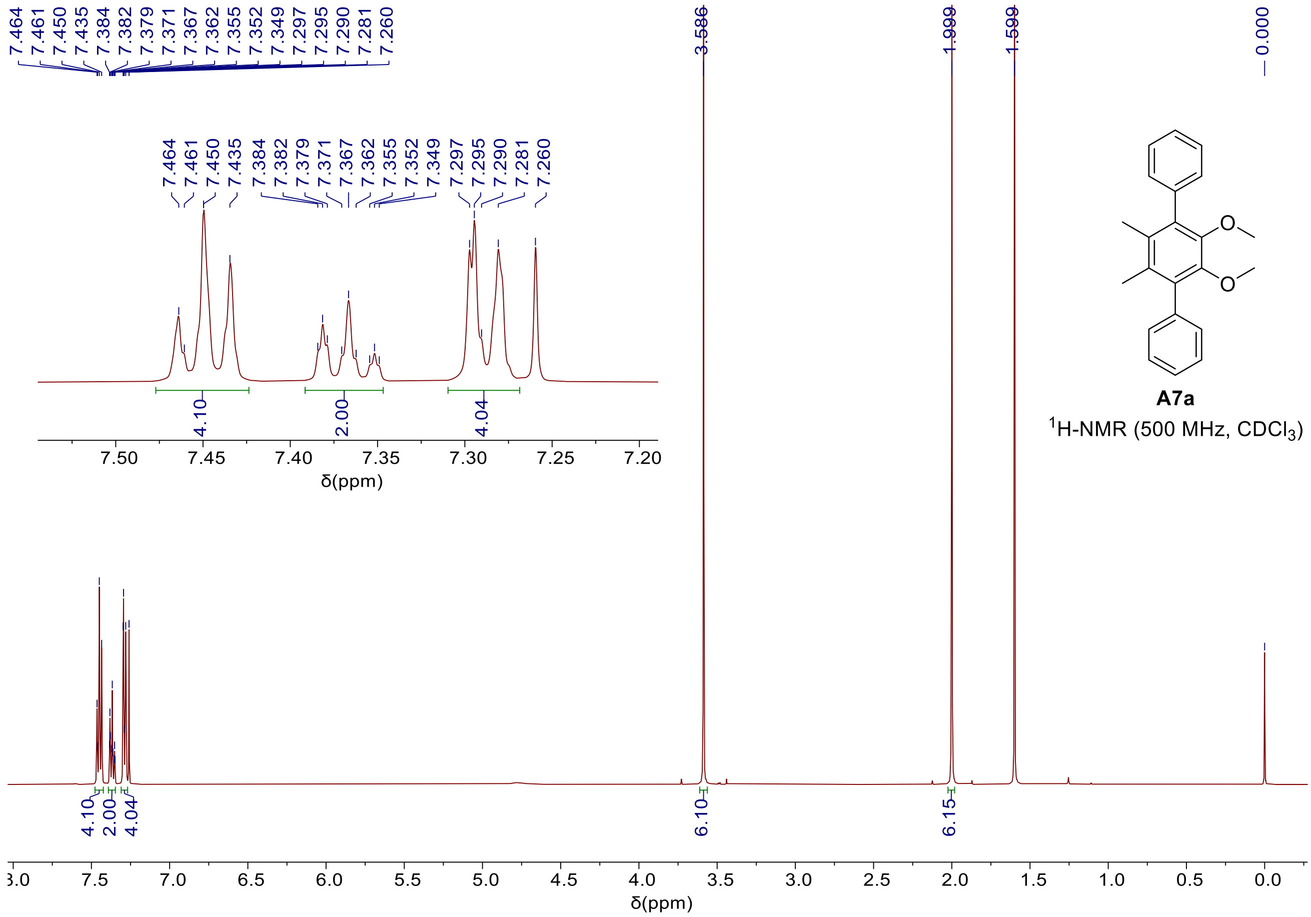
7.464
7.461
7.450
7.435
7.384
7.382
7.379
7.371
7.367
7.362
7.355
7.352
7.349
7.297
7.295
7.290
7.281
7.260
3.586
1.999
1.599
0.000
4.10
2.00
4.04
6.10
6.15
δ(ppm)
O
O
A7a
¹H-NMR (500 MHz, CDCl₃)

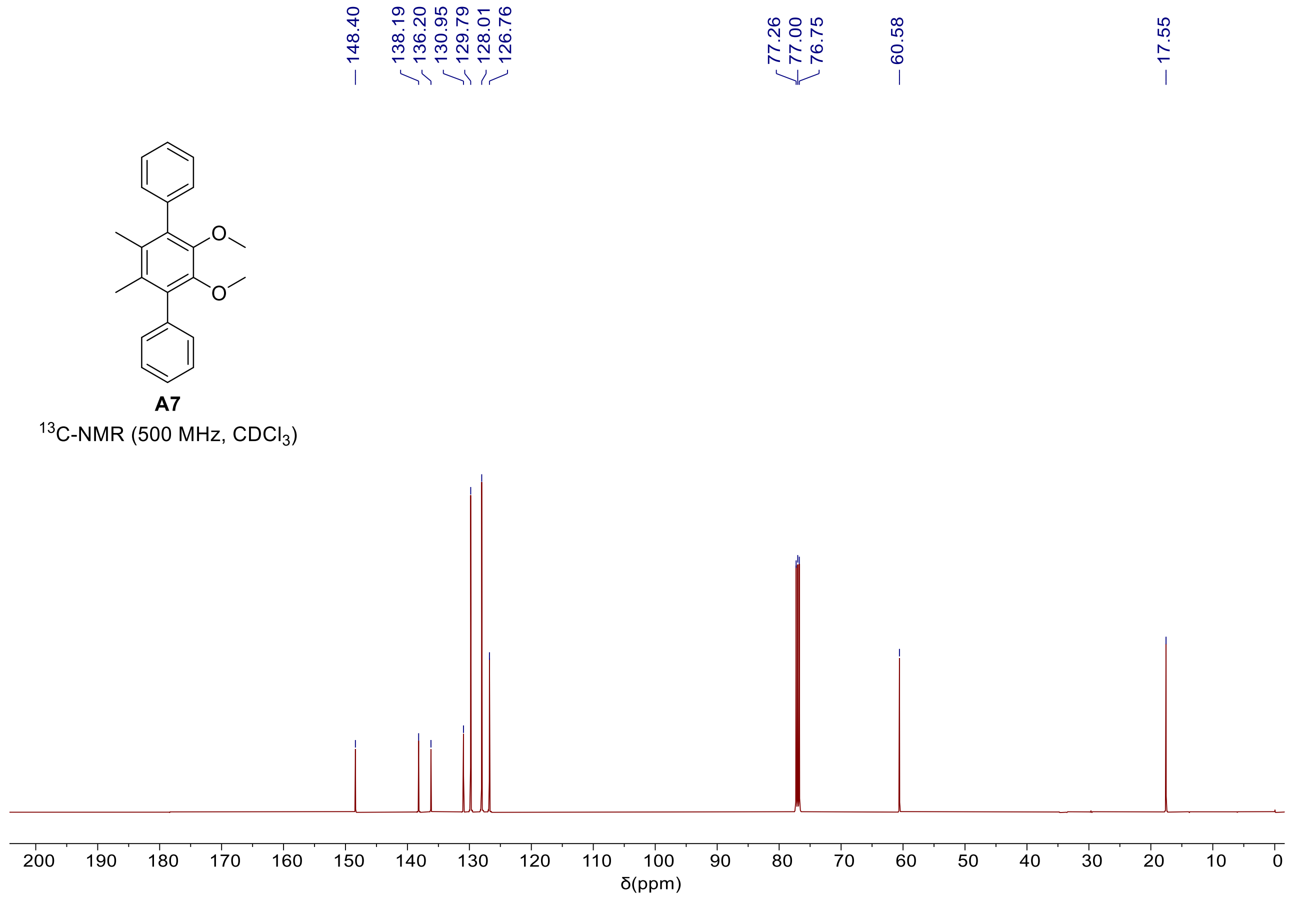
148.40
138.19
136.20
130.95
129.79
128.01
126.76
77.26
77.00
76.75
60.58
17.55
O
O
A7
¹³C-NMR (500 MHz, CDCl₃)
δ(ppm)

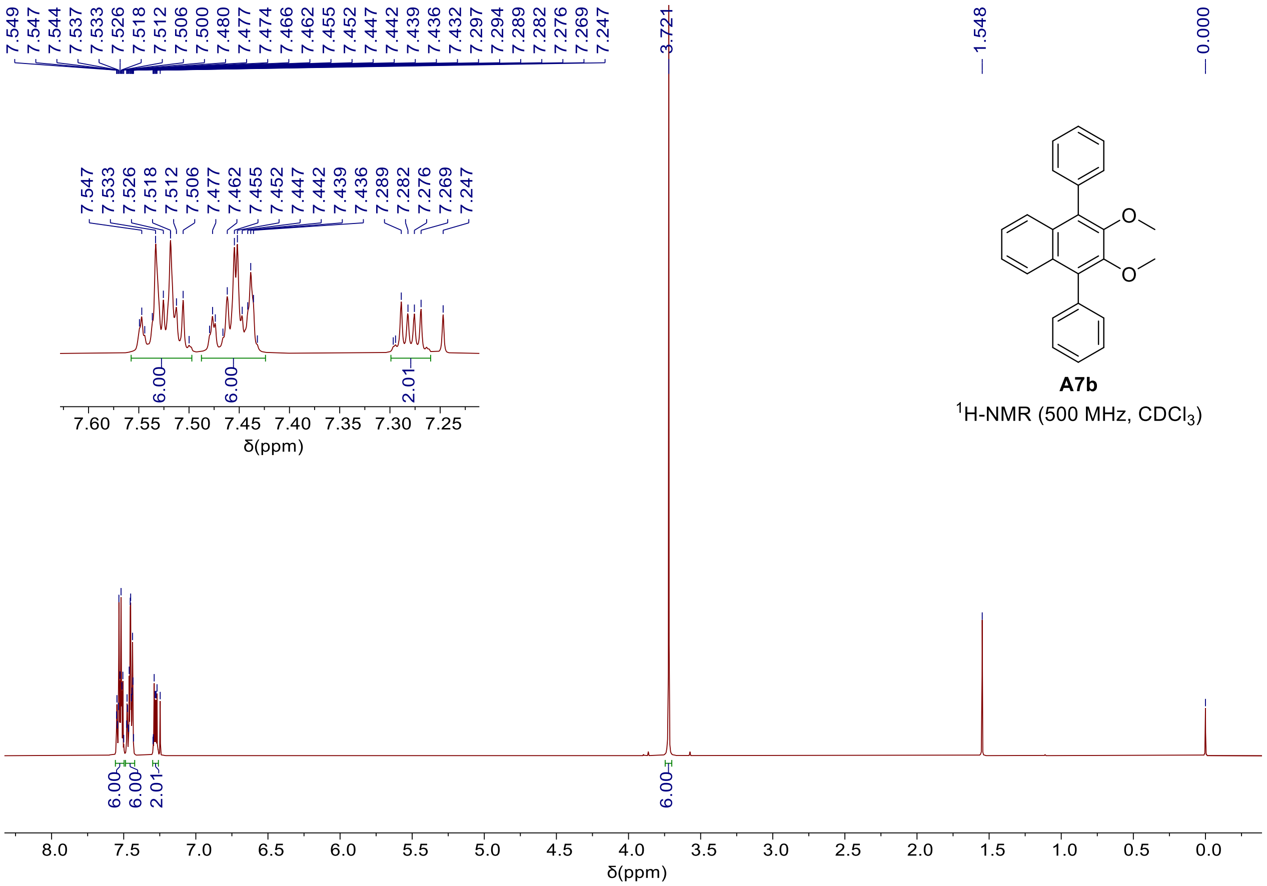
7.549 7.547 7.544 7.537 7.533 7.526 7.518 7.512 7.506 7.500 7.480 7.477 7.474 7.466 7.462 7.455 7.452 7.447 7.442 7.439 7.436 7.432 7.297 7.294 7.289 7.282 7.276 7.269 7.247
3.721
1.548
0.000
A7b
^{1}H-NMR (500 MHz, CDCl$_3$)
6.00 6.00 2.01 6.00
δ(ppm)

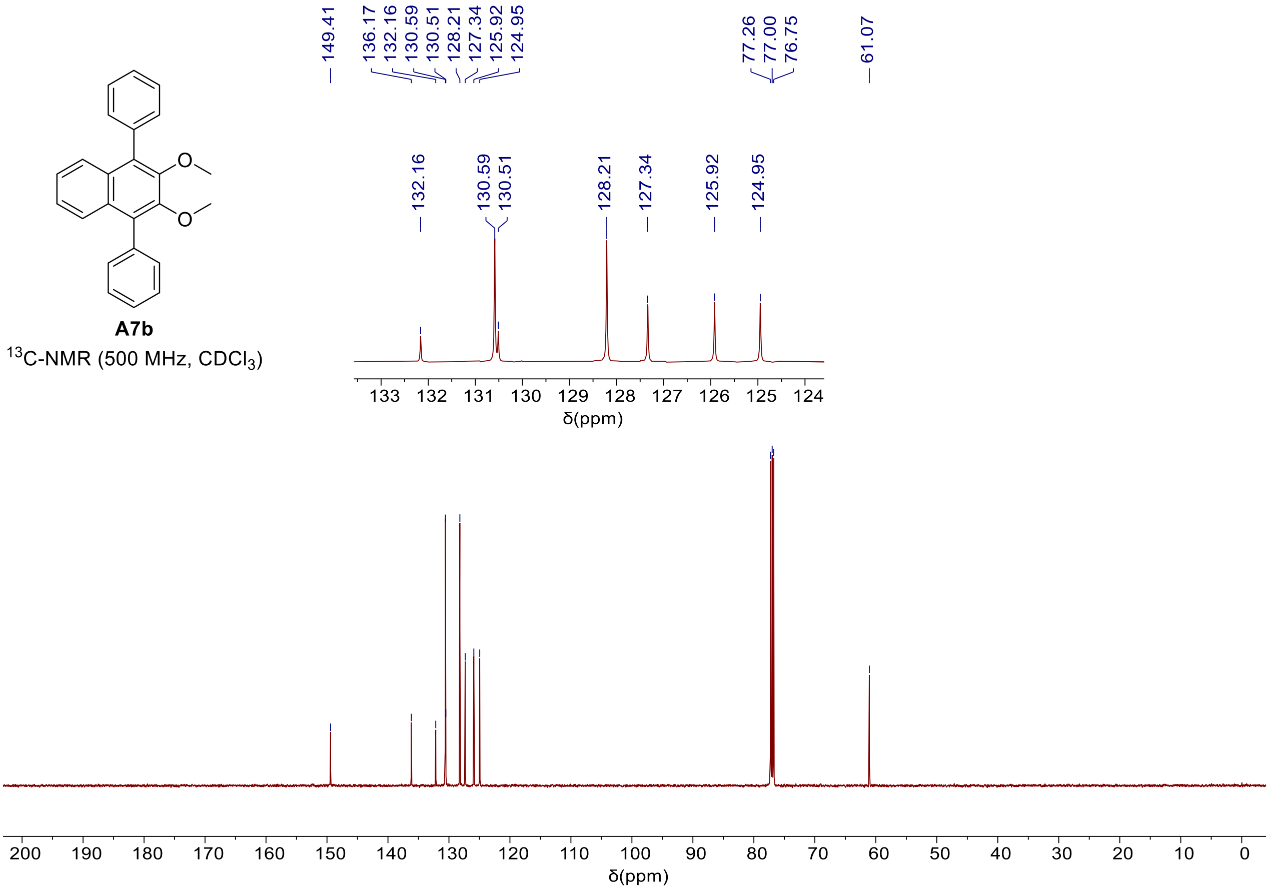
149.41
136.17 132.16 130.59 130.51 128.21 127.34 125.92 124.95
77.26 77.00 76.75
61.07
A7b
^{13}C-NMR (500 MHz, CDCl$_3$)
δ(ppm)

Cartesian coordinates of the optimized structures

CC1a_S_0

C	-0.00000100	4.67049000	0.00006800
C	1.21031700	3.97921100	0.00005600
C	1.23396100	2.57501400	0.00002500
C	0.00000000	1.86011400	0.00000300
C	-1.23396200	2.57501400	0.00002000
C	-1.21031900	3.97921000	0.00005300
C	2.45931000	1.82867500	0.00002100
C	0.00000000	0.43415800	-0.00003900
C	1.24042400	-0.28156800	-0.00005700
C	2.48872500	0.46215400	-0.00001200
C	1.21096800	-1.68644100	-0.00012200
H	2.14782100	-2.23441000	-0.00014000
C	0.00000200	-2.37445300	-0.00016000
C	-1.21096500	-1.68644300	-0.00012600
C	-1.24042300	-0.28156900	-0.00006400
C	-2.48872400	0.46215200	-0.00002900
C	-2.45931000	1.82867300	0.00001000
H	-3.39604200	2.38194500	0.00004100
H	3.39604100	2.38194800	0.00004900
H	-0.00000200	5.75723000	0.00009300
H	2.15091700	4.52471800	0.00007300
H	-2.15092000	4.52471700	0.00006600
H	0.00000300	-3.46126100	-0.00021500
H	-2.14781700	-2.23441300	-0.00015100
C	-3.80509600	-0.25706800	-0.00001000
C	-4.42858200	-0.59044700	-1.22078900
C	-4.42848800	-0.59053700	1.22080700
C	-5.66534500	-1.24369100	-1.19762900
C	-5.66524200	-1.24377800	1.19769700
C	-6.30415700	-1.57550900	0.00004400
H	-6.14046300	-1.50077000	-2.14266700
H	-6.14029100	-1.50093600	2.14274800
C	3.80509700	-0.25706400	-0.00000500
C	4.42855900	-0.59046800	-1.22079100
C	4.42851000	-0.59051500	1.22080500
C	5.66532100	-1.24371000	-1.19764300
C	5.66526400	-1.24375900	1.19768300
C	6.30415700	-1.57550600	0.00002500
H	6.14042000	-1.50081000	-2.14268500

H	6.14032600	-1.50090400	2.14273100
C	7.65550800	-2.25208000	0.00004900
H	8.47033000	-1.51539900	0.00001700
H	7.78897600	-2.88170200	0.88638600
H	7.78897500	-2.88177200	-0.88624000
C	3.78114100	-0.25519000	2.54640100
H	3.66272800	0.82692000	2.67657500
H	2.77909500	-0.69235500	2.62806200
H	4.38251500	-0.63088200	3.38002600
C	3.78124300	-0.25511100	-2.54640500
H	2.77926000	-0.69240700	-2.62817500
H	3.66268900	0.82699200	-2.67649500
H	4.38272400	-0.63065500	-3.38001900
C	-7.65551000	-2.25207700	0.00008600
H	-7.78896900	-2.88169700	0.88642500
H	-8.47033000	-1.51539300	0.00006200
H	-7.78899000	-2.88177000	-0.88620100
C	-3.78108800	-0.25523700	2.54639400
H	-2.77908200	-0.69249300	2.62806500
H	-3.66257400	0.82686500	2.67653800
H	-4.38249500	-0.63085200	3.38003000
C	-3.78129400	-0.25506800	-2.54641200
H	-3.66274800	0.82703800	-2.67649000
H	-2.77931100	-0.69235700	-2.62820800
H	-4.38279000	-0.63060300	-3.38001900

BO1a_S_0

C	0.00001600	4.44592300	-0.00019000
C	-1.21505300	3.75947600	0.00595400
C	-1.20828000	2.36681300	0.00641400
C	-0.00000100	1.63909100	-0.00006900
C	1.20829000	2.36679700	-0.00661400
C	1.21507700	3.75946200	-0.00627500
C	-0.00000500	0.19266900	-0.00000500
C	-1.23966300	-0.49745800	0.01569800
C	-1.20813100	-1.90141400	0.01145300
H	-2.14855800	-2.44767700	0.01804600
C	-0.00002600	-2.60140000	0.00012800
C	1.20808700	-1.90143300	-0.01126800
C	1.23964300	-0.49747600	-0.01564700
H	0.00002200	5.53205600	-0.00023800
H	-2.16627100	4.28088100	0.01140100
H	2.16630100	4.28085600	-0.01176300
H	-0.00003200	-3.68808900	0.00018500

H	2.14850600	-2.44771100	-0.01781200
C	4.00432800	-0.24143500	-0.03117000
C	4.73194600	-0.34625000	1.17525700
C	4.60561800	-0.68306500	-1.22977200
C	6.02042300	-0.88782500	1.16679200
C	5.89836000	-1.21740600	-1.20397400
C	6.62447600	-1.32756300	-0.01525200
H	6.56599200	-0.96993300	2.10557200
H	6.34929300	-1.55568100	-2.13549800
C	-4.00433000	-0.24141800	0.03123000
C	-4.60561700	-0.68302600	1.22983000
C	-4.73194200	-0.34626700	-1.17520600
C	-5.89835300	-1.21738900	1.20404200
C	-6.02040800	-0.88785900	-1.16673200
C	-6.62446200	-1.32758000	0.01532200
H	-6.34927500	-1.55565500	2.13557400
H	-6.56597900	-0.97000200	-2.10550900
C	-8.03043300	-1.88150500	0.00896700
H	-8.77590700	-1.07689700	0.06811100
H	-8.23633500	-2.44425400	-0.90836000
H	-8.20345500	-2.54740300	0.86111800
C	-4.12589500	0.12484700	-2.48142600
H	-3.94695600	1.20715800	-2.47173300
H	-3.15950300	-0.35758900	-2.67779300
H	-4.78331100	-0.09595500	-3.32837400
C	-3.87316200	-0.56601000	2.55124300
H	-2.89488900	-1.06135200	2.52125800
H	-3.69172100	0.48369400	2.81639500
H	-4.44870200	-1.01606300	3.36645300
C	8.03045400	-1.88147000	-0.00889900
H	8.20350100	-2.54731400	-0.86108600
H	8.77591900	-1.07684900	-0.06797700
H	8.23634600	-2.44427200	0.90839900
C	3.87321300	-0.56599600	-2.55120600
H	2.89462000	-1.06068700	-2.52105800
H	3.69245700	0.48374700	-2.81668200
H	4.44845200	-1.01667000	-3.36628600
C	4.12583900	0.12476000	2.48148800
H	3.94580300	1.20688100	2.47144500
H	3.15999300	-0.35858500	2.67833100
H	4.78373500	-0.09507300	3.32831400
B	-2.54101600	0.33964100	0.02608200
B	2.54100500	0.33959700	-0.02609900
O	-2.41304400	1.71818200	0.01492900

O	2.41303800	1.71815200	-0.01505600

CC1b_S_0

C	1.21328000	4.12151000	-0.00131900
C	-1.23323900	2.71823900	0.00036200
C	0.00264100	4.81367400	-0.00197100
C	1.23591600	2.71670600	0.00041700
C	0.00089600	2.00337500	0.00202000
C	-1.20885600	4.12301700	-0.00137900
H	0.00331800	5.90130200	-0.00332100
H	2.15499400	4.66689700	-0.00243500
H	-2.14989100	4.66957100	-0.00254500
C	0.00002000	0.57788200	0.00539100
C	-0.00174900	-2.25595900	0.01153400
C	-1.23722100	-0.14053300	0.00537800
C	1.23635300	-0.14208200	0.00539600
C	1.20328500	-1.54652300	0.00727800
C	-1.20588000	-1.54500900	0.00732800
H	2.14443100	-2.09127600	0.00415500
H	-2.14772700	-2.08853000	0.00428300
C	2.48681500	0.59929700	0.00074600
C	2.46024400	1.96700900	-0.00034800
C	-2.48676100	0.60239600	0.00064400
C	-2.45850200	1.97007300	-0.00046700
C	-3.80219100	-0.11934900	-0.00234500
C	-6.30305100	-1.43631300	-0.00877200
C	-4.43331600	-0.44454400	1.21708900
C	-4.42127100	-0.45552100	-1.22501500
C	-5.66035200	-1.10566400	-1.20538300
C	-5.67217800	-1.09488400	1.19108900
H	-6.13562300	-1.35859400	-2.15259600
H	-6.15672100	-1.33945800	2.13579800
C	3.80176400	-0.12333600	-0.00215600
C	6.29822400	-1.44856700	-0.00833500
C	4.42971900	-0.45429600	1.21736800
C	4.41911100	-0.46288900	-1.22475700
C	5.65502300	-1.11906000	-1.20498300
C	5.66539300	-1.11068000	1.19150500
H	6.12594000	-1.38055400	-2.15203100
H	6.14443300	-1.36568900	2.13624700
C	-7.62518900	-2.16741500	-0.01198500
H	-8.21533000	-1.92540100	-0.90289400
H	-8.22406500	-1.91734800	0.87083000
H	-7.47744900	-3.25635400	-0.00630300

C	-3.79453200	-0.09623000	2.54300200
H	-3.67678700	0.98767800	2.66204300
H	-2.79185000	-0.53187400	2.63177100
H	-4.39976400	-0.46478200	3.37738200
C	-3.76951700	-0.11878000	-2.54759500
H	-4.36875300	-0.49078700	-3.38476000
H	-2.76779500	-0.55885000	-2.62449900
H	-3.64659000	0.96384300	-2.67283400
C	-0.00268200	-3.76699900	0.04697500
H	-0.89052800	-4.17831900	-0.44549900
H	-0.00262300	-4.13798700	1.08120300
H	0.88438000	-4.17944300	-0.44596300
C	3.78727200	-0.11260300	2.54323200
H	3.66972200	0.97080200	2.66697300
H	4.39027300	-0.48517400	3.37743800
H	2.78418900	-0.54821000	2.62740400
C	7.64783600	-2.12768400	-0.01176700
H	7.77682000	-2.75930100	-0.89775100
H	7.78337000	-2.75522000	0.87614900
H	8.46334700	-1.39111700	-0.01643200
C	3.76518800	-0.13039900	-2.54734600
H	2.76288200	-0.56963600	-2.62124200
H	4.36276100	-0.50561100	-3.38426700
H	3.64324800	0.95195100	-2.67586400
H	3.39953100	2.51805200	-0.00261500
H	-3.39710100	2.52229300	-0.00282400

BO1b_ S_0

C	0.00000300	4.59305200	0.00691700
C	-1.21529200	3.90659600	0.01235100
C	-1.20829000	2.51396200	0.01199900
C	0.00005600	1.78711900	0.00589200
C	1.20837400	2.51400900	-0.00071700
C	1.21532000	3.90664600	0.00037600
C	0.00008900	0.34174400	0.00685700
C	-1.23591100	-0.35205800	0.02064500
C	-1.20107200	-1.75476800	0.01597200
H	-2.14438900	-2.29831200	0.01749200
C	0.00021900	-2.47641600	0.00816500
C	1.20138300	-1.75471800	-0.00660900
C	1.23607600	-0.35198600	-0.01009200
H	-0.00001700	5.67919000	0.00743500
H	-2.16639200	4.42828800	0.01753500
H	2.16639400	4.42838100	-0.00512300

H	2.14466200	-2.29819300	-0.01854300
C	4.00159900	-0.09804500	-0.03298200
C	4.73135100	-0.20793500	1.17165700
C	4.60024700	-0.53634900	-1.23406900
C	6.01918700	-0.75101400	1.15905300
C	5.89236300	-1.07244900	-1.21255900
C	6.62055900	-1.18747100	-0.02557200
H	6.56642700	-0.83689000	2.09654700
H	6.34116700	-1.40810300	-2.14608200
C	-4.00160600	-0.09809300	0.02775300
C	-4.60775000	-0.53721400	1.22477500
C	-4.72400500	-0.20680000	-1.18142100
C	-5.89984300	-1.07296400	1.19490000
C	-6.01203500	-0.74958600	-1.17715800
C	-6.62076600	-1.18689900	0.00337700
H	-6.35445700	-1.40923300	2.12538800
H	-6.55355000	-0.83447300	-2.11806100
C	-8.02621800	-1.74214100	-0.00758000
H	-8.77278100	-0.93816100	0.04626400
H	-8.22769300	-2.30748400	-0.92431600
H	-8.20230800	-2.40600200	0.84555300
C	-4.11276200	0.26174200	-2.48613600
H	-3.93482700	1.34422800	-2.47817100
H	-3.14498200	-0.22012700	-2.67692500
H	-4.76627700	0.03833900	-3.33544700
C	-3.88089800	-0.41597900	2.54886300
H	-2.90079200	-0.90788500	2.52282200
H	-3.70392400	0.63469100	2.81317400
H	-4.45802400	-0.86686500	3.36253100
C	8.02599100	-1.74287400	-0.02369300
H	8.19684000	-2.40595300	-0.87849800
H	8.77226400	-0.93888900	-0.08134300
H	8.23300900	-2.30907500	0.89127800
C	3.86524600	-0.41390900	-2.55355800
H	2.88528200	-0.90580200	-2.52202600
H	3.68671300	0.63701200	-2.81581800
H	4.43734800	-0.86403000	-3.37119500
C	4.12807400	0.25953000	2.48045100
H	3.94951700	1.34191800	2.47421200
H	3.16172100	-0.22294900	2.67689100
H	4.78695700	0.03592500	3.32555600
B	-2.53830900	0.48397400	0.02743100
B	2.53837200	0.48409900	-0.02353500
O	-2.41222100	1.86282300	0.01855400

O	2.41231300	1.86292900	-0.01065100
C	0.00031800	-3.98735600	0.03751200
H	0.00814300	-4.36504600	1.06877300
H	-0.89009600	-4.39732700	-0.45066400
H	0.88296600	-4.39781900	-0.46413000

CC1c_ S_0

H	3.46143800	-0.07596400	0.00000000
C	2.37327600	-0.05626300	0.00000000
C	-0.39056200	-0.01337000	0.00000000
C	1.73595700	-0.04498800	-1.23962400
C	1.73595700	-0.04498800	1.23962400
C	0.31575900	-0.02145700	1.26611000
C	0.31575900	-0.02145700	-1.26611000
C	-1.82710700	0.00204600	0.00000000
C	-4.66434800	0.02793700	0.00000000
C	-2.56131600	0.00959400	1.22223600
C	-2.56131600	0.00959400	-1.22223600
C	-3.96650900	0.02217400	-1.20456600
C	-3.96650900	0.02217400	1.20456600
H	-4.50296600	0.02771100	-2.15124200
H	-4.50296600	0.02771100	2.15124200
H	-5.75187600	0.03754100	0.00000000
C	-0.46707300	-0.00631900	2.50040700
C	-0.46707300	-0.00631900	-2.50040700
C	-1.83310500	0.00679100	2.44710400
H	-2.39596600	0.01799300	3.37898200
C	-1.83310500	0.00679100	-2.44710400
H	-2.39596600	0.01799300	-3.37898200
C	2.64943000	-0.06263000	2.44890100
H	3.69108000	-0.09324900	2.11362200
H	2.48237000	-0.93094000	3.09246600
H	2.53088900	0.82162700	3.08177200
C	2.64943000	-0.06263000	-2.44890100
H	2.48237000	-0.93094000	-3.09246600
H	3.69108000	-0.09324900	-2.11362200
H	2.53088900	0.82162700	-3.08177200
C	0.10843300	0.00215100	-3.89134400
C	1.04588800	0.02356300	-6.55841100
C	0.30796400	-1.21205000	-4.58065100
C	0.35309900	1.22770700	-4.54522000
C	0.82422200	1.21591900	-5.86283800
C	0.77985100	-1.17945000	-5.89769400
H	1.02246700	2.16577500	-6.35845600

H	0.94347400	-2.12116100	-6.42070500
C	0.10843300	0.00215100	3.89134400
C	1.04588800	0.02356300	6.55841100
C	0.30796400	-1.21205000	4.58065100
C	0.35309900	1.22770700	4.54522000
C	0.82422200	1.21591900	5.86283800
C	0.77985100	-1.17945000	5.89769400
H	1.02246700	2.16577500	6.35845600
H	0.94347400	-2.12116100	6.42070500
C	0.12648000	2.54622200	3.83956600
H	0.70455000	2.61235600	2.90935200
H	0.41476600	3.38711600	4.47841400
H	-0.92615300	2.67596100	3.56041200
C	0.03422200	-2.54147200	3.91297100
H	0.60608900	-2.65291900	2.98315000
H	-1.02314500	-2.64398100	3.64062400
H	0.29686500	-3.37339600	4.57424700
C	1.52691100	0.03561500	7.99059700
H	2.11181000	-0.86079400	8.22487100
H	0.68284800	0.06565400	8.69364500
H	2.15117000	0.91219200	8.19677300
C	0.12648000	2.54622200	-3.83956600
H	0.70455000	2.61235600	-2.90935200
H	-0.92615300	2.67596100	-3.56041200
H	0.41476600	3.38711600	-4.47841400
C	0.03422200	-2.54147200	-3.91297100
H	0.29686500	-3.37339600	-4.57424700
H	-1.02314500	-2.64398100	-3.64062400
H	0.60608900	-2.65291900	-2.98315000
C	1.52691100	0.03561500	-7.99059700
H	2.11181000	-0.86079400	-8.22487100
H	2.15117000	0.91219200	-8.19677300
H	0.68284800	0.06565400	-8.69364500

BO1c_ S_0

C	0.00001100	-4.47302500	-0.00001500
C	1.21047600	-3.78182400	0.00068200
C	1.19999700	-2.38875100	0.00066300
C	0.00000400	-1.64848600	-0.00001400
C	-1.19998500	-2.38875800	-0.00069200
C	-1.21045600	-3.78183100	-0.00071100
C	0.00000000	-0.19372000	-0.00001300
C	1.25566400	0.48859300	0.00127100
C	1.23085300	1.90597100	0.00243900

C	-0.00001500	2.56668800	-0.00001300
C	-1.23087500	1.90595700	-0.00246400
C	-1.25566900	0.48858100	-0.00129600
H	0.00001500	-5.55916100	-0.00001500
H	2.16557000	-4.29606900	0.00116500
H	-2.16554800	-4.29608200	-0.00119400
C	-4.05775700	0.08073400	-0.00037700
C	-4.75007000	0.26908800	1.21542000
C	-4.75467500	0.25399300	-1.21437800
C	-6.09580400	0.64744600	1.19941300
C	-6.10162600	0.63235200	-1.19698500
C	-6.79184800	0.83536200	0.00094400
H	-6.61470500	0.79741000	2.14506200
H	-6.62491600	0.77008100	-2.14187500
C	4.05776000	0.08074400	0.00037100
C	4.75465800	0.25397600	1.21438700
C	4.75009600	0.26910900	-1.21541000
C	6.10161000	0.63233400	1.19702400
C	6.09583100	0.64746400	-1.19937400
C	6.79185400	0.83536200	-0.00089000
H	6.62488400	0.77004900	2.14192500
H	6.61474700	0.79744400	-2.14501100
C	8.25365100	1.21881400	-0.00459300
H	8.89650900	0.34018900	-0.15070900
H	8.48269900	1.92229500	-0.81307600
H	8.54859100	1.68381600	0.94203100
C	4.04628200	0.05610000	-2.54036000
H	3.75995400	-0.99481600	-2.67480200
H	3.12474000	0.64818900	-2.61371400
H	4.68749000	0.33462100	-3.38279000
C	4.05694700	0.02627800	2.54016000
H	3.13608900	0.61804900	2.62431000
H	3.77078500	-1.02591600	2.66428100
H	4.70209800	0.29531200	3.38266100
C	-8.25364500	1.21881400	0.00467900
H	-8.54860200	1.68383300	-0.94193200
H	-8.89650100	0.34018600	0.15079000
H	-8.48268000	1.92228000	0.81317800
C	-4.05698500	0.02631200	-2.54016500
H	-3.13613100	0.61808600	-2.62432200
H	-3.77082300	-1.02588000	-2.66430400
H	-4.70215100	0.29535400	-3.38265300
C	-4.04623700	0.05605200	2.54035500
H	-3.75990800	-0.99486700	2.67477400

H	-3.12469300	0.64813900	2.61370900
H	-4.68743300	0.33455600	3.38280000
B	2.54586900	-0.37581400	0.00100800
B	-2.54586800	-0.37582900	-0.00103700
O	2.40379700	-1.75172200	0.00105300
O	-2.40379000	-1.75173600	-0.00108300
H	-0.00002100	3.65436200	-0.00001400
C	-2.47850300	2.76155000	-0.00628100
H	-3.10641800	2.56683100	-0.88098700
H	-3.10691200	2.57401900	0.86966900
H	-2.21090600	3.82268300	-0.01041100
C	2.47846200	2.76159000	0.00625400
H	3.10637400	2.56689500	0.88096700
H	3.10688100	2.57406700	-0.86969000
H	2.21083900	3.82271700	0.01037300

CC1d_S_0

C	0.00000000	0.00000000	1.60467800
C	0.00000000	0.00000000	-1.23248100
C	-0.00834900	-1.20684000	0.89027900
C	0.00834900	1.20684000	0.89027900
C	0.00726900	1.23656600	-0.51282200
C	-0.00726900	-1.23656600	-0.51282200
C	0.00000000	0.00000000	-2.65742700
C	0.00000000	0.00000000	-5.46714800
C	0.01072500	1.23475300	-3.37125000
C	-0.01072500	-1.23475300	-3.37125000
C	-0.01050800	-1.21118500	-4.77590600
C	0.01050800	1.21118500	-4.77590600
H	-0.01862600	-2.15239700	-5.32202200
H	0.01862600	2.15239700	-5.32202200
H	0.00000000	0.00000000	-6.55475200
C	0.02109800	2.48724300	-1.25466600
C	-0.02109800	-2.48724300	-1.25466600
C	0.02170900	2.45955500	-2.62216900
H	0.03022700	3.39841000	-3.17381000
C	-0.02170900	-2.45955500	-2.62216900
H	-0.03022700	-3.39841000	-3.17381000
C	-0.03052000	-3.80207500	-0.53205800
C	-0.04734200	-6.29745800	0.79447400
C	-1.25577000	-4.40915000	-0.18358400
C	1.18638500	-4.43964800	-0.20979100
C	1.15519400	-5.67462200	0.44762200
C	-1.24106000	-5.64488800	0.47299600

H	2.09780800	-6.16124800	0.69606600
H	-2.18992800	-6.10777800	0.74185600
C	0.03052000	3.80207500	-0.53205800
C	0.04734200	6.29745800	0.79447400
C	-1.18638500	4.43964800	-0.20979100
C	1.25577000	4.40915000	-0.18358400
C	1.24106000	5.64488800	0.47299600
C	-1.15519400	5.67462200	0.44762200
H	2.18992800	6.10777800	0.74185600
H	-2.09780800	6.16124800	0.69606600
C	2.57538300	3.74438600	-0.50599000
H	2.64299400	2.74695400	-0.05475600
H	3.41463700	4.34127500	-0.13516100
H	2.70555800	3.60969700	-1.58659800
C	-2.51519800	3.80926100	-0.56281600
H	-2.60847900	2.80223700	-0.13824900
H	-2.63594600	3.70375600	-1.64786300
H	-3.34676000	4.41421600	-0.18754600
C	0.05691500	7.64633400	1.47488100
H	-0.83431200	7.78847200	2.09621500
H	0.07335900	8.46236400	0.73906400
H	0.93938900	7.76739200	2.11287000
C	2.51519800	-3.80926100	-0.56281600
H	2.60847900	-2.80223700	-0.13824900
H	2.63594600	-3.70375600	-1.64786300
H	3.34676000	-4.41421600	-0.18754600
C	-2.57538300	-3.74438600	-0.50599000
H	-3.41463700	-4.34127500	-0.13516100
H	-2.70555800	-3.60969700	-1.58659800
H	-2.64299400	-2.74695400	-0.05475600
C	-0.05691500	-7.64633400	1.47488100
H	-0.93938900	-7.76739200	2.11287000
H	0.83431200	-7.78847200	2.09621500
H	-0.07335900	-8.46236400	0.73906400
H	0.04265500	2.14846600	1.43033400
H	-0.04265500	-2.14846600	1.43033400
C	0.00000000	0.00000000	3.09134200
C	0.00000000	0.00000000	5.91248000
C	-0.71943000	0.96758200	3.81400200
C	0.71943000	-0.96758200	3.81400200
C	0.71920100	-0.96806700	5.20846500
C	-0.71920100	0.96806700	5.20846500
H	-1.30289900	1.71095400	3.27653600
H	1.30289900	-1.71095400	3.27653600

H	1.28950600	-1.72226700	5.74614100
H	-1.28950600	1.72226700	5.74614100
H	0.00000000	0.00000000	6.99993000

BO1d_S$_0$

C	0.00001600	5.26478500	-0.00007900
C	-1.21534800	4.57856600	0.01805600
C	-1.20851100	3.18602700	0.01830300
C	0.00001000	2.45924000	-0.00002700
C	1.20853500	3.18602200	-0.01838700
C	1.21537700	4.57856100	-0.01819000
C	0.00000800	1.01470300	-0.00000200
C	-1.23575200	0.32023800	0.02517500
C	-1.20358500	-1.08089900	0.02189200
C	0.00000400	-1.80680100	0.00001900
C	1.20359100	-1.08090600	-0.02186100
C	1.23576200	0.32023500	-0.02516000
H	0.00001800	6.35090700	-0.00009800
H	-2.16626400	5.10033600	0.03293000
H	2.16629500	5.10032600	-0.03308200
C	4.00301200	0.57754100	-0.06414900
C	4.74993800	0.51362200	1.13351400
C	4.58887600	0.10355900	-1.25826400
C	6.04160400	-0.02030200	1.12123500
C	5.88538400	-0.42176100	-1.23630200
C	6.63035800	-0.49167500	-0.05624900
H	6.60217400	-0.07099600	2.05339000
H	6.32434700	-0.78473700	-2.16421000
C	-4.00300300	0.57756100	0.06416000
C	-4.58889800	0.10367800	1.25830200
C	-4.74990600	0.51355200	-1.13351500
C	-5.88540600	-0.42164200	1.23635100
C	-6.04157100	-0.02037400	-1.12122200
C	-6.63035100	-0.49165400	0.05628600
H	-6.32439400	-0.78453700	2.16427900
H	-6.60211800	-0.07114300	-2.05338600
C	-8.03990300	-1.03640100	0.05588500
H	-8.77799500	-0.23186000	0.17680600
H	-8.27252000	-1.54834700	-0.88443600
H	-8.19625700	-1.74562800	0.87574100
C	-4.16197600	1.01964900	-2.43511900
H	-3.97545200	2.09990700	-2.39632500
H	-3.20227000	0.53730500	-2.66222700

H	-4.83480700	0.82810700	-3.27706800
C	-3.83834200	0.17977800	2.57253700
H	-2.85734600	-0.30673700	2.51212300
H	-3.66112900	1.22108700	2.87184300
H	-4.39942100	-0.30264000	3.37921500
C	8.03990900	-1.03642400	-0.05583600
H	8.19626200	-1.74567200	-0.87567400
H	8.77800200	-0.23188700	-0.17677700
H	8.27252700	-1.54834800	0.88449800
C	3.83825400	0.17949800	-2.57247100
H	2.85739900	-0.30730400	-2.51203700
H	3.66072800	1.22076500	-2.87173200
H	4.39944100	-0.30273300	-3.37918600
C	4.16205100	1.01985800	2.43508400
H	3.97579500	2.10016300	2.39627700
H	3.20221300	0.53775400	2.66213400
H	4.83478500	0.82815500	3.27707400
B	-2.53914000	1.15613000	0.05087600
B	2.53915200	1.15611700	-0.05088500
O	-2.41193500	2.53499400	0.03866900
O	2.41195500	2.53498300	-0.03873100
H	-2.14664400	-1.62068300	0.05966600
H	2.14665200	-1.62068700	-0.05963300
C	-0.00000700	-3.29227600	0.00001800
C	0.97911200	-4.01453400	0.70352800
C	-0.97914700	-4.01450900	-0.70349500
C	0.97964700	-5.40847900	0.70297100
H	1.73167200	-3.47881800	1.27542500
C	-0.97971600	-5.40845100	-0.70294200
H	-1.73169200	-3.47876800	-1.27538900
C	-0.00004200	-6.11203700	0.00001400
H	1.74266400	-5.94551200	1.26030100
H	-1.74274500	-5.94546700	-1.26027300
H	-0.00005700	-7.19865100	0.00001200

CC1e_S_0

C	-0.00096200	1.23888300	-0.00000400
C	0.00193000	-1.59394700	0.00000000
C	-1.20735500	0.52818600	-0.00002700
C	1.20688700	0.53066400	0.00001900
C	1.23895200	-0.87451000	0.00002000
C	-1.23656100	-0.87704800	-0.00002500
C	0.00337300	-3.01988400	0.00000700
C	0.00623000	-5.83032300	0.00002200

C	1.23851300	-3.73286000	0.00003200
C	-1.23031100	-3.73537500	-0.00001100
C	-1.20546900	-5.14015600	-0.00000300
C	1.21652100	-5.13769000	0.00003900
H	-2.14632100	-5.68695800	-0.00001600
H	2.15847900	-5.68258500	0.00005800
H	0.00733900	-6.91793600	0.00002800
C	2.48968100	-1.61578300	0.00004300
C	-2.48577900	-1.62086200	-0.00004300
C	2.46281900	-2.98339200	0.00004900
H	3.40202500	-3.53451700	0.00006600
C	-2.45614500	-2.98841200	-0.00003500
H	-3.39422900	-3.54144500	-0.00004800
C	-3.80256400	-0.90168500	-0.00006500
C	-6.30138300	0.41860500	-0.00010400
C	-4.42633400	-0.56881600	-1.22113100
C	-4.42640500	-0.56887900	1.22098100
C	-5.66318100	0.08536300	1.19814400
C	-5.66311200	0.08542200	-1.19833200
H	-6.13854000	0.34294000	2.14400300
H	-6.13841800	0.34304500	-2.14420500
C	3.80501500	-0.89395200	0.00005400
C	6.30131800	0.43109200	0.00007300
C	4.42820600	-0.55995300	-1.22099600
C	4.42815000	-0.55988400	1.22111400
C	5.66369100	0.09668900	1.19830500
C	5.66374500	0.09662200	-1.19816800
H	6.13851000	0.35522300	2.14417300
H	6.13860800	0.35510100	-2.14402900
C	3.78232400	-0.90068700	2.54554200
H	2.77327000	-0.47758800	2.62208700
H	4.37613400	-0.51571100	3.38065600
H	3.67740200	-1.98476100	2.67577300
C	3.78244200	-0.90083400	-2.54543500
H	2.77338600	-0.47775300	-2.62204900
H	3.67753900	-1.98491700	-2.67561400
H	4.37628500	-0.51589600	-3.38054200
C	7.64987100	1.11218800	0.00008400
H	7.78091200	1.74201700	-0.88682300
H	8.46649400	0.37682800	0.00012400
H	7.78087200	1.74206800	0.88696200
C	-3.77997200	-0.90848500	2.54541900
H	-2.77180200	-0.48330400	2.62206000
H	-3.67281500	-1.99235100	2.67553600

H	-4.37463000	-0.52482400	3.38053600
C	-3.77982200	-0.90835200	-2.54554800
H	-4.37448300	-0.52473800	-3.38068400
H	-3.67255800	-1.99220600	-2.67566800
H	-2.77169000	-0.48307400	-2.62214500
C	-7.65126100	1.09708000	-0.00012500
H	-7.78350300	1.72668700	-0.88701500
H	-7.78354700	1.72665500	0.88678000
H	-8.46645400	0.36013700	-0.00015800
H	2.14503700	1.07943900	0.00004100
H	-2.14661100	1.07506200	-0.00004700
C	-0.00301100	2.73915700	0.00000300
C	-0.00328400	5.56521000	0.00002100
C	-0.00121600	3.44613300	-1.22046000
C	-0.00190700	3.44611800	1.22047500
C	-0.00094700	4.84515100	1.19800000
C	-0.00028100	4.84516700	-1.19796600
H	0.00333800	5.38599700	2.14383700
H	0.00452500	5.38602500	-2.14379300
C	0.00400900	2.71684800	-2.54542300
H	0.00359000	3.42388000	-3.38112800
H	-0.87311000	2.06663900	-2.65112200
H	0.88542800	2.07185500	-2.64757300
C	0.00257000	2.71681500	2.54543200
H	-0.87476700	2.06683700	2.65075700
H	0.00197900	3.42383700	3.38114500
H	0.88377300	2.07158800	2.64794100
C	-0.03571400	7.07576600	0.00002400
H	-1.06853000	7.45115500	-0.00025300
H	0.45896700	7.48761400	-0.88670800
H	0.45848300	7.48759700	0.88703500

BO1e_ S_0

C	-0.00822300	-5.65134300	-0.00398900
C	-1.22259200	-4.96340400	-0.01446700
C	-1.21386600	-3.57077600	-0.01346900
C	-0.00450100	-2.84550300	-0.00121600
C	1.20294000	-3.57400800	0.00964200
C	1.20796800	-4.96665500	0.00787200
C	-0.00259300	-1.39987900	-0.00000700
C	-1.23830600	-0.70522900	-0.02036100
C	-1.20342500	0.69792900	-0.01554900
C	0.00122800	1.41749400	0.00128000
C	1.20388600	0.69461000	0.01780000

C	1.23498400	-0.70863000	0.02130500
H	-0.00966700	-6.73747500	-0.00509200
H	-2.17432100	-5.48383900	-0.02442800
H	2.15831400	-5.48962800	0.01675700
C	4.00063500	-0.96755700	0.04825300
C	4.73231900	-0.86019000	-1.15538700
C	4.59875800	-0.53043700	1.25001500
C	6.02158400	-0.32068000	-1.14119100
C	5.89231400	0.00194800	1.23010500
C	6.62243500	0.11461700	0.04408000
H	6.57024400	-0.23636000	-2.07797100
H	6.34051400	0.33724000	2.16400300
C	-4.00471300	-0.95702200	-0.04719200
C	-4.60185100	-0.51565300	-1.24786000
C	-4.73613000	-0.85083400	1.15675200
C	-5.89418300	0.01971100	-1.22662100
C	-6.02411900	-0.30830600	1.14389000
C	-6.62398400	0.13127400	-0.04032600
H	-6.34163300	0.35825500	-2.15970000
H	-6.57255000	-0.22495800	2.08089400
C	-8.02933600	0.68675800	-0.03875200
H	-8.77561200	-0.11709700	-0.09826400
H	-8.23689800	1.25160300	0.87690600
H	-8.19923400	1.35127200	-0.89258500
C	-4.13442300	-1.32132500	2.46521400
H	-3.95556700	-2.40366400	2.45696300
H	-3.16835500	-0.83912300	2.66418500
H	-4.79434200	-1.09967100	3.30999800
C	-3.86492500	-0.63329500	-2.56665000
H	-2.88819200	-0.13472700	-2.53413200
H	-3.67896800	-1.68285600	-2.82921400
H	-4.43899000	-0.18641500	-3.38461600
C	8.02915600	0.66663500	0.04402900
H	8.19907500	1.33257300	0.89676600
H	8.77330500	-0.13894400	0.10684000
H	8.23991200	1.22894200	-0.87244300
C	3.86142700	-0.64948900	2.56844700
H	2.88571900	-0.14886000	2.53688500
H	3.67330100	-1.69925400	2.82863900
H	4.43628900	-0.20560200	3.38748300
C	4.12959700	-1.32622000	-2.46497600
H	3.94835400	-2.40818100	-2.45929500
H	3.16459100	-0.84144300	-2.66282300
H	4.79001400	-1.10403400	-3.30923300

B	-2.54210100	-1.53966300	-0.03687700
B	2.53655400	-1.54651200	0.03664500
O	-2.41691500	-2.91867800	-0.02631900
O	2.40774100	-2.92516300	0.02375400
H	-2.14316300	1.24618600	-0.02817100
H	2.14507700	1.24035300	0.03075400
C	0.00413300	2.91624200	0.00057000
C	0.12465400	3.62146300	-1.21499200
C	-0.11750200	3.62386500	1.21411800
C	0.12204400	5.01960700	-1.19368200
C	-0.11332300	5.02242300	1.18990600
C	0.00851000	5.74079100	-0.00205700
H	0.20999700	5.55895500	-2.13513300
H	-0.20879200	5.56388700	2.12930700
C	0.04285600	7.25160200	-0.00187800
H	1.07388000	7.62645700	0.05257500
H	-0.40259800	7.66305400	-0.91419500
H	-0.49841300	7.66535500	0.85564900
C	-0.25127500	2.89739100	2.53460700
H	-1.15991100	2.28442800	2.57223500
H	0.59162100	2.21846500	2.71078400
H	-0.29170000	3.60661300	3.36697400
C	0.24762600	2.89195500	-2.53484900
H	1.15193500	2.27279500	-2.57491800
H	-0.60049600	2.21844000	-2.70671400
H	0.28971400	3.59958500	-3.36848700

CC1f_S_0

C	0.00000000	0.00000000	-3.50164500
C	0.00000000	0.00000000	-0.69310600
C	0.00695500	1.21174300	-2.81335400
C	-0.00695500	-1.21174300	-2.81335400
C	-0.00727700	-1.24090900	-1.40791400
C	0.00727700	1.24090900	-1.40791400
C	0.00000000	0.00000000	0.73192000
C	0.00000000	0.00000000	3.56906100
C	-0.00214700	-1.23077300	1.45038200
C	0.00214700	1.23077300	1.45038200
C	0.00151300	1.20671400	2.85285100
C	-0.00151300	-1.20671400	2.85285100
H	0.03117100	2.15041000	3.39325200
H	-0.03117100	-2.15041000	3.39325200
C	-0.01563100	-2.48859600	-0.66210800
C	0.01563100	2.48859600	-0.66210800

C	-0.01339400	-2.45852900	0.70533600
H	-0.01949700	-3.39588500	1.25941700
C	0.01339400	2.45852900	0.70533600
H	0.01949700	3.39588500	1.25941700
C	0.02716300	3.80524900	-1.38163900
C	0.04940700	6.30398300	-2.70168700
C	-1.18845300	4.43976900	-1.71444900
C	1.25391400	4.41686800	-1.71657300
C	1.24193400	5.65414100	-2.37031200
C	-1.15452700	5.67661700	-2.36820600
H	2.19198600	6.12077200	-2.62844800
H	-2.09613600	6.16089100	-2.62479800
C	-0.02716300	-3.80524900	-1.38163900
C	-0.04940700	-6.30398300	-2.70168700
C	-1.25391400	-4.41686800	-1.71657300
C	1.18845300	-4.43976900	-1.71444900
C	1.15452700	-5.67661700	-2.36820600
C	-1.24193400	-5.65414100	-2.37031200
H	2.09613600	-6.16089100	-2.62479800
H	-2.19198600	-6.12077200	-2.62844800
C	2.51859600	-3.80335400	-1.37802000
H	2.60934300	-2.80400500	-1.82092700
H	3.34861400	-4.41425300	-1.74692900
H	2.64390800	-3.67902200	-0.29555700
C	-2.57243200	-3.75589700	-1.38180900
H	-2.64330400	-2.75437700	-1.82335200
H	-2.69739900	-3.63094000	-0.29937800
H	-3.41311600	-4.35046300	-1.75323600
C	-0.06149600	-7.65481700	-3.37818800
H	-0.94996600	-7.78056900	-4.00691700
H	-0.06804000	-8.46878500	-2.63994400
H	0.82389400	-7.79594200	-4.00802500
C	2.57243200	3.75589700	-1.38180900
H	2.64330400	2.75437700	-1.82335200
H	2.69739900	3.63094000	-0.29937800
H	3.41311600	4.35046300	-1.75323600
C	-2.51859600	3.80335400	-1.37802000
H	-3.34861400	4.41425300	-1.74692900
H	-2.64390800	3.67902200	-0.29555700
H	-2.60934300	2.80400500	-1.82092700
C	0.06149600	7.65481700	-3.37818800
H	-0.82389400	7.79594200	-4.00802500
H	0.94996600	7.78056900	-4.00691700
H	0.06804000	8.46878500	-2.63994400

H	-0.01222100	-2.15004100	-3.36085800
H	0.01222100	2.15004100	-3.36085800
H	0.00000000	0.00000000	-4.58931200
C	0.00000000	0.00000000	5.05543900
C	0.00000000	0.00000000	7.87616200
C	-0.75606300	0.93925900	5.77771200
C	0.75606300	-0.93925900	5.77771200
C	0.75604700	-0.93971800	7.17228500
C	-0.75604700	0.93971800	7.17228500
H	-1.36862800	1.65817600	5.23901400
H	1.36862800	-1.65817600	5.23901400
H	1.35540800	-1.67085100	7.71022200
H	-1.35540800	1.67085100	7.71022200
H	0.00000000	0.00000000	8.96357100

BO1f_S_0

C	-0.00008900	0.53373000	0.04588700
C	-0.00190500	3.36888000	0.09372800
C	1.20512000	1.26600700	0.05580900
C	-1.20622100	1.26443900	0.05720400
C	-1.21222600	2.65508800	0.08069500
C	1.20933900	2.65666600	0.08064200
H	-2.16815100	3.16862900	0.05906500
H	2.16462300	3.17041000	0.11924700
C	0.00075900	-0.91037300	0.01491100
C	0.00290200	-3.70259100	-0.08299800
C	-1.23945700	-1.60049900	-0.01022800
C	1.24195100	-1.59906000	0.00069200
C	1.21149300	-3.00256400	-0.05103900
C	-1.20672700	-3.00395600	-0.06270200
H	2.15320200	-3.54831100	-0.06895500
H	-2.14753700	-3.55084900	-0.09022500
H	0.00371800	-4.78924900	-0.12594400
C	-0.00298200	4.85397200	0.12026800
C	-0.00525000	7.67276300	0.17097000
C	0.95957400	5.58912800	-0.59299000
C	-0.96673300	5.56146000	0.85944000
C	-0.96761100	6.95554300	0.88481600
C	0.95826300	6.98322100	-0.56818200
H	1.69802100	5.06242000	-1.19226400
H	-1.70423400	5.01237300	1.43945200
H	-1.71735000	7.48241700	1.47063900
H	1.70721800	7.53203900	-1.13454600
H	-0.00609900	8.75995100	0.19053300

O	-2.41248500	0.61980700	0.03366900
O	2.41231000	0.62255800	0.05079900
B	2.54122800	-0.75797300	0.02878700
B	-2.53994900	-0.76079500	0.00495200
C	-4.00506400	-1.33575700	-0.03769900
C	-6.63150700	-2.40383900	-0.15737800
C	-4.72780400	-1.33464900	-1.25252700
C	-4.61468800	-1.87391300	1.11705800
C	-5.91072500	-2.39736900	1.04009100
C	-6.01930000	-1.86849000	-1.29569400
H	-6.36870400	-2.80969200	1.93893700
H	-6.56072300	-1.86883800	-2.24152100
C	4.00708700	-1.33252800	0.00862900
C	6.63465600	-2.40131700	-0.06610700
C	4.60458800	-1.85130800	1.17836400
C	4.74207400	-1.35159600	-1.19850200
C	6.03411600	-1.88543700	-1.21956600
C	5.90149700	-2.37529900	1.12359100
H	6.58529100	-1.90114400	-2.15962000
H	6.35023400	-2.77249800	2.03381900
C	3.86933200	-1.82032900	2.50264100
H	2.87230900	-2.27168300	2.42730400
H	3.72767800	-0.78975600	2.85524900
H	4.42270400	-2.35993500	3.27826800
C	4.13841300	-0.79887300	-2.47315400
H	3.18170600	-1.28324400	-2.71082300
H	4.80572200	-0.94766600	-3.32831000
H	3.93940000	0.27670000	-2.38579700
C	8.04365500	-2.94497600	-0.10153900
H	8.26142600	-3.43395100	-1.05792400
H	8.21326800	-3.67295000	0.69940200
H	8.78219000	-2.14135700	0.02641100
C	-8.03973100	-2.94743300	-0.21627500
H	-8.25160100	-3.40909000	-1.18739500
H	-8.77977300	-2.14842300	-0.06946100
H	-8.21316000	-3.69807200	0.56267700
C	-3.89559600	-1.86214700	2.45053500
H	-2.88982800	-2.29361300	2.37728200
H	-4.44870300	-2.42961500	3.20623800
H	-3.77696600	-0.83789800	2.82934100
C	-4.11178400	-0.76111900	-2.51203000
H	-3.15379600	-1.24305400	-2.74958100
H	-3.91193000	0.31240000	-2.40465500
H	-4.77167100	-0.89436800	-3.37544700

CC1g_S_0

C	-4.02489500	-0.03338600	0.00000600
C	-1.21607900	-0.01021700	0.00000200
C	-3.32656800	-1.23928900	0.00000300
C	-3.34655500	1.18387500	0.00000600
C	-1.94127300	1.22456300	0.00000400
C	-1.92078300	-1.25683700	0.00000100
C	0.20963500	0.00153600	0.00000100
C	3.04330700	0.02495800	-0.00000100
C	0.91667100	1.23897100	0.00000200
C	0.93705300	-1.22398500	-0.00000200
C	2.34119300	-1.18745700	-0.00000300
C	2.32118200	1.22561900	0.00000100
H	2.89541000	-2.12447900	-0.00000500
H	2.85992300	2.17162600	0.00000200
C	-1.20636500	2.47857100	0.00000400
C	-1.16518600	-2.49849600	-0.00000100
C	0.16143700	2.46005500	0.00000400
H	0.70766800	3.40204300	0.00000400
C	0.20213200	-2.45739400	-0.00000300
H	0.76381400	-3.39025300	-0.00000500
C	-1.87409000	-3.82094500	-0.00000300
C	-3.17457600	-6.33009400	-0.00000500
C	-2.20306500	-4.44672100	-1.22120000
C	-2.20305600	-4.44672800	1.22119400
C	-2.84715400	-5.68887700	1.19826900
C	-2.84716200	-5.68887100	-1.19827700
H	-3.10081600	-6.16633600	2.14414200
H	-3.10083100	-6.16632400	-2.14415100
C	-1.93709100	3.78911300	0.00000400
C	-3.27767900	6.27709000	0.00000300
C	-2.27618600	4.40945900	-1.22118800
C	-2.27618000	4.40946200	1.22119400
C	-2.94003300	5.64118400	1.19827100
C	-2.94003800	5.64117900	-1.19826500
H	-3.20128300	6.11452200	2.14415200
H	-3.20129100	6.11451700	-2.14414600
C	-1.93539800	3.76347200	2.54558700
H	-2.36936800	2.75936400	2.62628000
H	-2.31061500	4.36312300	3.38096700
H	-0.85195600	3.64781400	2.67077600
C	-1.93540700	3.76346400	-2.54557900
H	-2.36938100	2.75935700	-2.62626900

H	-0.85196500	3.64780200	-2.67076800
H	-2.31062200	4.36311400	-3.38096000
C	-3.96524000	7.62237900	0.00000000
H	-4.59562800	7.75062400	-0.88694800
H	-3.23369300	8.44239700	-0.00003900
H	-4.59557100	7.75065700	0.88698200
C	-1.87256200	-3.79538800	2.54557500
H	-2.32246100	-2.79832300	2.62624200
H	-0.79108900	-3.66247600	2.67069200
H	-2.23810900	-4.40094300	3.38098900
C	-1.87258000	-3.79537400	-2.54558100
H	-2.23812400	-4.40093000	-3.38099500
H	-0.79111000	-3.66245000	-2.67070000
H	-2.32249200	-2.79831400	-2.62624300
C	-3.84041900	-7.68627200	-0.00000500
H	-4.46860100	-7.82470800	-0.88698700
H	-4.46866300	-7.82467700	0.88693800
H	-3.09579200	-8.49443400	0.00003600
H	-3.90163100	2.11772200	0.00000800
H	-3.86627500	-2.18212000	0.00000300
H	-5.11253400	-0.04237200	0.00000800
C	4.54302900	0.03738400	-0.00000200
C	7.36821700	0.06630900	-0.00000300
C	5.24931400	0.04606100	-1.22086600
C	5.24931600	0.04602800	1.22086300
C	6.64831500	0.06146500	1.19822700
C	6.64831500	0.06149600	-1.19823000
H	7.18923200	0.07164200	2.14397200
H	7.18923000	0.07169700	-2.14397700
C	4.51858400	0.04307100	2.54522700
H	3.87735100	-0.84067100	2.65003900
H	3.86284200	0.91651800	2.64655300
H	5.22472400	0.05051000	3.38161700
C	4.51858300	0.04313800	-2.54523100
H	5.22472400	0.05057700	-3.38162000
H	3.86285900	0.91660100	-2.64654400
H	3.87733300	-0.84058900	-2.65005800
C	8.87903800	0.04980700	0.00000000
H	9.28567700	0.54839100	-0.88692900
H	9.26524500	-0.97900100	0.00007400
H	9.28567400	0.54851500	0.88686200

BO1g_S_0

C	-0.00510400	2.86438600	-0.00069700

C	-1.21437400	2.15426000	0.00497100
C	-1.20647700	0.76220800	0.00566200
C	0.00012400	0.03393400	-0.00052700
C	1.20402800	0.76666900	-0.00681200
C	1.20679500	2.15873900	-0.00630100
C	0.00280800	-1.41142600	-0.00030500
C	-1.23597200	-2.10299700	0.01567700
C	-1.20175200	-3.50687500	0.01159500
C	0.00791500	-4.20444200	0.00046100
C	1.21501000	-3.50244700	-0.01107100
C	1.24412400	-2.09844500	-0.01596700
H	-2.16679500	2.67425000	0.00995500
H	2.15729800	2.68224100	-0.01135400
C	4.00802200	-1.83314700	-0.03156900
C	4.73841900	-1.92822100	1.17400300
C	4.60855400	-2.28002200	-1.22863500
C	6.02867300	-2.46553700	1.16635300
C	5.90316200	-2.80979700	-1.20209300
C	6.63197900	-2.91030100	-0.01417700
H	6.57629500	-2.54011900	2.10456200
H	6.35351300	-3.15201500	-2.13246500
C	-4.00070000	-1.84822400	0.03171000
C	-4.60001800	-2.29365800	1.22989700
C	-4.72995600	-1.95037700	-1.17396400
C	-5.89234100	-2.82904200	1.20440700
C	-6.01791000	-2.49315700	-1.16525500
C	-6.61998500	-2.93663300	0.01640000
H	-6.34175100	-3.17012300	2.13564500
H	-6.56468300	-2.57317000	-2.10351600
C	-8.02550800	-3.49168400	0.01046300
H	-8.77154000	-2.68778000	0.07204400
H	-8.23197600	-4.05274100	-0.90777800
H	-8.19705400	-4.15944600	0.86145300
C	-4.12614500	-1.47516500	-2.47973300
H	-3.94834600	-0.39270800	-2.46721400
H	-3.15955300	-1.95617600	-2.67866800
H	-4.78451100	-1.69433500	-3.32636900
C	-3.86620300	-2.17932400	2.55079800
H	-2.88718200	-2.67299400	2.51833000
H	-3.68621200	-1.13006400	2.81873700
H	-4.44016200	-2.63257000	3.36535900
C	8.03993200	-3.45913500	-0.00737100
H	8.21290500	-4.13133600	-0.85457900
H	8.78226400	-2.65232100	-0.07531000

H	8.25053300	-4.01361200	0.91390800
C	3.87384400	-2.17278100	-2.54963200
H	2.89576200	-2.66809300	-2.51464400
H	3.69179500	-1.12499900	-2.82197400
H	4.44831200	-2.62838900	-3.36251600
C	4.13340600	-1.45131700	2.47860600
H	3.95202800	-0.36950000	2.46308900
H	3.16847400	-1.93496000	2.67921900
H	4.79275000	-1.66593600	3.32564400
B	-2.53777600	-1.26613500	0.02601700
B	2.54284100	-1.25678100	-0.02659600
O	-2.41124300	0.11295300	0.01418200
O	2.41115400	0.12184500	-0.01519300
H	-2.14100800	-4.05518200	0.01821600
H	2.15625300	-4.04734500	-0.01733000
H	0.00991500	-5.29113000	0.00079200
C	-0.00829400	4.36342500	-0.00073900
C	-0.00750200	5.06834500	1.22037500
C	-0.00738300	5.06833700	-1.22177000
C	-0.00745800	6.46670300	1.19702200
C	-0.00747000	6.46676000	-1.19836000
C	-0.01111000	7.18637100	-0.00069300
H	-0.00317700	7.00714000	2.14188100
H	-0.00333100	7.00723100	-2.14319100
C	-0.00224300	4.33904700	-2.54731900
H	0.88050200	3.69743600	-2.65358500
H	-0.87733200	3.68715700	-2.65405400
H	-0.00609800	5.04713100	-3.38162500
C	-0.00232900	4.33895600	2.54586800
H	-0.88078500	3.69206300	2.65546600
H	0.87706000	3.69229900	2.64917200
H	0.00040700	5.04699700	3.38021400
C	-0.04434700	8.69725300	-0.00051500
H	-1.07647300	9.07297300	0.00743900
H	0.45673600	9.10952000	0.88196800
H	0.44328500	9.10938000	-0.89050900

CC2_S_0

C	-5.44156000	3.28017000	-0.02234400
C	-5.00334600	1.97163800	-0.00117900
C	-3.62112400	1.65977200	-0.02345100
C	-2.66391200	2.71919300	-0.05502900
C	-3.15016000	4.05032600	-0.07782800
C	-4.50332900	4.32824800	-0.06613100

C	-1.24322000	2.41079600	-0.06613400
C	-0.81649700	1.05997600	-0.20225700
C	0.59597900	0.75849900	-0.32734900
C	1.52257400	1.78021400	-0.00198400
C	1.06532500	3.10904400	0.20775600
C	-0.26164000	3.42499100	0.11495100
H	-6.50565500	3.49995600	-0.00989000
H	-5.71836400	1.15566400	0.03091000
H	-2.45342500	4.87931900	-0.13035000
H	-4.84154600	5.36058900	-0.09511800
H	1.79828000	3.88125000	0.42808100
H	-0.56534900	4.45591800	0.25405400
C	1.14373400	-0.51972400	-0.76512500
C	2.53711900	-0.79989800	-0.56387800
C	0.39041400	-1.48382000	-1.48541900
C	3.05324100	-2.06471900	-0.93957300
C	0.92890900	-2.69673900	-1.87251600
H	-0.62068900	-1.24748100	-1.79004000
C	2.26429200	-3.00897500	-1.56522000
H	0.31824400	-3.40073300	-2.43142700
H	2.68442200	-3.96868200	-1.85354000
H	4.10284100	-2.26849600	-0.75326300
C	-4.12042700	-0.84217600	0.18744100
C	-4.45641200	-1.29386500	1.48161800
C	-4.68034200	-1.47366600	-0.94255500
C	-5.33936300	-2.36930000	1.61997900
C	-5.56076100	-2.54564700	-0.75834600
C	-5.90567600	-3.00878700	0.51357300
H	-5.59048300	-2.71614300	2.62081300
H	-5.98735600	-3.03073000	-1.63447700
C	4.87351200	-0.04333700	0.19087800
C	5.82781800	0.21950900	-0.81387800
C	5.29091400	-0.54851000	1.44074100
C	7.18033800	-0.02171500	-0.54796800
C	6.65263600	-0.77470600	1.66369600
C	7.61565000	-0.51403200	0.68467800
H	7.91199300	0.17952700	-1.32835600
H	6.96819500	-1.16553600	2.62946300
C	-1.81538300	0.03585100	-0.08572200
C	-3.15649400	0.29490900	0.01617700
H	-1.49501300	-0.99468200	-0.00320700
C	3.41956000	0.22727400	-0.05707600
C	2.91310000	1.48070800	0.14022200
H	3.57674800	2.28487300	0.45043100

C	-4.34236300	-1.01481100	-2.34418700
H	-4.54656700	0.05340000	-2.48084700
H	-3.28044300	-1.16262000	-2.57513900
H	-4.92581600	-1.56809800	-3.08663900
C	-3.87211300	-0.63828200	2.71331600
H	-2.78119800	-0.74190700	2.74771500
H	-4.08647900	0.43654400	2.73745500
H	-4.28180400	-1.08527400	3.62439600
C	-6.88256500	-4.14816200	0.69055600
H	-7.90418000	-3.77700300	0.84903400
H	-6.90601200	-4.79690600	-0.19147000
H	-6.62602000	-4.76586700	1.55833700
C	4.28973600	-0.84787400	2.53440700
H	3.72293300	0.04650300	2.81824800
H	3.55498000	-1.59659900	2.21505600
H	4.79127900	-1.22878300	3.42939600
C	5.41355900	0.74925200	-2.16927200
H	4.66715200	0.10218700	-2.64438700
H	4.96318700	1.74606600	-2.09551100
H	6.27581400	0.81974400	-2.83958100
C	9.08462300	-0.73699900	0.96094400
H	9.53536100	0.13664800	1.45096600
H	9.24279800	-1.59522700	1.62332800
H	9.64409600	-0.91516500	0.03648100

BO2_S_0

C	5.31203700	3.65090900	-0.17777000
C	4.95332500	2.31235800	-0.11912400
C	3.60114900	1.91783100	-0.07512800
C	2.58173900	2.90616700	-0.09923600
C	2.96344200	4.26311200	-0.15943600
C	4.30345000	4.62458000	-0.19615300
C	1.17004000	2.48784700	-0.06701400
C	0.83102300	1.11865200	-0.01706300
C	-0.51341400	0.63722400	0.00756800
C	-1.50856700	1.63864300	-0.02548600
C	-1.20184800	3.00384000	-0.07029700
C	0.11024900	3.41793800	-0.08999900
H	6.35748800	3.94476900	-0.21063400
H	5.72034200	1.54199600	-0.10738500
H	2.21753100	5.04998100	-0.17857200
H	4.56885500	5.67786700	-0.24207900
H	-2.02495100	3.70990800	-0.09107100
H	0.31957900	4.48032600	-0.12632400

C	-0.92877300	-0.78610800	0.06169500
C	-2.32766300	-1.07206700	0.05489300
C	-0.03683500	-1.88123000	0.12482400
C	-2.77149800	-2.40729200	0.10702700
C	-0.50929600	-3.18860700	0.17357700
H	1.02815000	-1.71967100	0.13955700
C	-1.87902600	-3.46785500	0.16401600
H	0.21027500	-4.00234500	0.22175700
H	-2.23502800	-4.49379700	0.20357200
H	-3.84207500	-2.59519600	0.10435300
B	3.17822200	0.44314000	-0.01050000
O	1.82148000	0.18174200	0.00846100
O	-2.84308500	1.35927800	-0.01880900
B	-3.34825600	0.07724600	0.00854600
C	4.13189700	-0.81078300	0.01628300
C	4.36537100	-1.54869000	-1.16577700
C	4.77351500	-1.21431700	1.20913400
C	5.22587800	-2.65087400	-1.14035700
C	5.62551700	-2.32310000	1.19979500
C	5.86436600	-3.05753800	0.03445500
H	5.40418700	-3.20400000	-2.06108600
H	6.11474400	-2.62149900	2.12570700
C	-4.91763800	-0.06586100	0.00725000
C	-5.64281900	0.02534000	1.21653700
C	-5.62307600	-0.29219800	-1.19382500
C	-7.03310500	-0.11601200	1.20716700
C	-7.01580400	-0.42703200	-1.16903200
C	-7.74081000	-0.34085900	0.02183500
H	-7.57807500	-0.05057100	2.14778100
H	-7.54674500	-0.60364000	-2.10303200
C	6.76737600	-4.26908300	0.05180600
H	7.59426100	-4.14362500	0.75960300
H	6.21813900	-5.17117300	0.35389800
H	7.19457700	-4.46545200	-0.93733000
C	3.69343000	-1.15658900	-2.46647000
H	2.60559300	-1.28821700	-2.41294600
H	3.87646400	-0.10368300	-2.71646700
H	4.05974400	-1.76263500	-3.30116800
C	4.53146100	-0.47262700	2.50821400
H	4.72420800	0.60204200	2.40723300
H	3.49131300	-0.58033800	2.84273400
H	5.17417300	-0.85329400	3.30842300
C	-4.88821500	-0.37569900	-2.51626900
H	-4.40993300	0.57944900	-2.76943700

H	-4.09589300	-1.13395700	-2.49388000
H	-5.56921800	-0.62962200	-3.33493600
C	-4.92359700	0.27584800	2.52628000
H	-4.15778800	-0.48623000	2.72006300
H	-4.41438300	1.24771000	2.52370600
H	-5.61970900	0.26796500	3.37110700
C	-9.24700700	-0.46400800	0.03049900
H	-9.72810600	0.52232600	0.07758200
H	-9.61383000	-0.96264500	-0.87283600
H	-9.59853100	-1.03401600	0.89814300

CC3a_S_0

C	1.22377600	0.08052300	-0.00004400
C	1.23125500	-1.35112900	-0.00000300
C	2.51907400	-2.04318600	0.00003600
C	3.72819200	-1.28249400	0.00001700
C	-0.00000100	0.75706400	-0.00006400
C	0.00000000	-2.01931600	0.00001400
C	-1.23125500	-1.35113000	-0.00001000
C	-1.22377800	0.08052200	-0.00005100
C	-3.72819200	-1.28249700	-0.00001800
C	-2.51907500	-2.04318800	0.00000700
H	-0.00000100	1.84526500	-0.00009400
H	0.00000000	-3.10107900	0.00005000
C	4.96858900	-1.96170700	0.00007900
C	2.62124200	-3.45227300	0.00010300
C	3.84772200	-4.09405800	0.00015100
C	5.03376000	-3.34337300	0.00014000
H	5.88152300	-1.37482800	0.00007300
H	1.72193900	-4.05845100	0.00012300
H	3.88818000	-5.17999100	0.00020400
H	5.99848800	-3.84336500	0.00018200
C	-4.96859000	-1.96171000	-0.00003400
C	-5.03376000	-3.34337700	-0.00000200
C	-3.84772100	-4.09406100	0.00003400
C	-2.62124100	-3.45227500	0.00003500
H	-5.88152300	-1.37483300	-0.00007200
H	-5.99848700	-3.84336900	-0.00001100
H	-3.88817800	-5.17999400	0.00005400
H	-1.72193700	-4.05845200	0.00005100
C	-4.93973200	0.98283700	-0.00003900
C	-5.53273600	1.36795400	-1.22082600
C	-5.53274100	1.36787000	1.22077500
C	-6.70240800	2.13482900	-1.19764300

C	-6.70241300	2.13474500	1.19764200
C	-7.30191000	2.53343700	0.00001100
H	-7.15745900	2.42598500	-2.14269500
H	-7.15746800	2.42583100	2.14271300
C	4.93973100	0.98283900	-0.00002500
C	5.53285600	1.36776500	-1.22081700
C	5.53262000	1.36806200	1.22078400
C	6.70252800	2.13463800	-1.19763900
C	6.70229500	2.13493300	1.19764600
C	7.30191200	2.53343400	0.00001400
H	7.15767400	2.42563900	-2.14269200
H	7.15725500	2.42617300	2.14271500
C	-4.92672800	0.96274200	-2.54641700
H	-3.92583800	1.38955800	-2.68097400
H	-4.81690000	-0.12523500	-2.62469100
H	-5.55103600	1.29993500	-3.37961300
C	-4.92674900	0.96253700	2.54633600
H	-4.81733600	-0.12547900	2.62468400
H	-3.92568000	1.38897000	2.68075100
H	-5.55084500	1.30003000	3.37957000
C	-8.54825700	3.38821300	0.00004200
H	-8.29822400	4.45781400	0.00024800
H	-9.16362500	3.20013800	-0.88636500
H	-9.16378800	3.19984000	0.88627400
C	8.54826000	3.38820700	0.00002700
H	8.29823000	4.45780900	-0.00029500
H	9.16354400	3.20020800	0.88650800
H	9.16387400	3.19975700	-0.88613000
C	4.92647800	0.96297000	2.54635100
H	3.92556200	1.38977300	2.68075400
H	4.81667100	-0.12500100	2.62472500
H	5.55068700	1.30026500	3.37958000
C	4.92699800	0.96230900	-2.54640200
H	4.81762200	-0.12571700	-2.62467200
H	3.92593000	1.38870300	-2.68094700
H	5.55116300	1.29975000	-3.37960400
C	-3.67771500	0.17197000	-0.00006900
C	-2.46718300	0.79860800	-0.00007600
C	3.67771400	0.17197200	-0.00004200
C	2.46718000	0.79861000	-0.00006000
H	-2.42691400	1.88571600	-0.00009300
H	2.42691100	1.88571800	-0.00008700

BO3a_S_0

C	1.20347900	0.15668400	0.00607000
C	1.23611500	1.57222200	0.00897900
C	2.53316900	2.26872300	0.02392200
C	3.72148200	1.48939700	0.02301900
C	0.00002500	-0.53875800	-0.00009800
C	0.00001700	2.23253400	0.00017500
C	-1.23607700	1.57221800	-0.00875600
C	-1.20342900	0.15668000	-0.00612500
C	-3.72144400	1.48938300	-0.02263600
C	-2.53313600	2.26871600	-0.02348900
H	0.00002700	-1.62270500	-0.00020900
H	0.00001500	3.31480200	0.00030700
C	4.97012300	2.14120800	0.04706100
C	2.64208800	3.67283400	0.04442500
C	3.88729500	4.28859100	0.06405500
C	5.06292100	3.52639100	0.06629000
H	5.87286500	1.53550100	0.05212300
H	1.75469600	4.29721900	0.04671700
H	3.94507200	5.37410800	0.07980900
H	6.03251100	4.01631100	0.08463400
C	-4.97009500	2.14118800	-0.04629300
C	-5.06290200	3.52637700	-0.06516500
C	-3.88728200	4.28858300	-0.06298000
C	-2.64206600	3.67283000	-0.04368800
H	-5.87283300	1.53547600	-0.05132000
H	-6.03249900	4.01629600	-0.08319700
H	-3.94506800	5.37410200	-0.07849600
H	-1.75467800	4.29722100	-0.04600100
B	-3.61006800	-0.05128800	-0.00852700
O	-2.34122700	-0.59823700	-0.00836100
O	2.34127800	-0.59822200	0.00815300
B	3.61012700	-0.05126700	0.00845300
C	-4.81536000	-1.06496100	-0.01157000
C	-5.49594300	-1.38753200	1.18318500
C	-5.23516800	-1.66624200	-1.21842600
C	-6.56747200	-2.28572600	1.15280700
C	-6.31238000	-2.55767500	-1.21466100
C	-6.99141000	-2.88403700	-0.03724100
H	-7.08358100	-2.52466600	2.08134800
H	-6.62991500	-3.00797200	-2.15381400
C	4.81538700	-1.06497000	0.01122400
C	5.23419400	-1.66765400	1.21777000
C	5.49688800	-1.38625800	-1.18332300
C	6.31131700	-2.55915000	1.21387500

C	6.56830500	-2.28463300	-1.15308800
C	6.99124800	-2.88428700	0.03660600
H	6.62806800	-3.01051600	2.15278400
H	7.08506500	-2.52266400	-2.08149600
C	-5.06503300	-0.78381300	2.50460000
H	-4.04348900	-1.08656100	2.76881400
H	-5.07549400	0.31269100	2.47448300
H	-5.72304900	-1.10048800	3.32012600
C	-4.52543300	-1.35474500	-2.52026300
H	-4.49788800	-0.27551900	-2.71903000
H	-3.48548800	-1.70363300	-2.50175400
H	-5.02194600	-1.83475300	-3.36959700
C	-8.13274600	-3.87453300	-0.04572800
H	-7.77448800	-4.89685300	0.13666400
H	-8.86824700	-3.64647100	0.73350700
H	-8.65165400	-3.88085400	-1.01037600
C	8.13251600	-3.87486700	0.04500700
H	7.77380400	-4.89757200	-0.13426800
H	8.86644300	-3.64859200	-0.73621500
H	8.65339900	-3.87905200	1.00861900
C	5.06724700	-0.78093700	-2.50442700
H	4.04517400	-1.08156700	-2.76898600
H	5.07979400	0.31552900	-2.47364600
H	5.72478300	-1.09835700	-3.32004900
C	4.52344300	-1.35754800	2.51938500
H	4.49560800	-0.27852300	2.71920400
H	3.48356200	-1.70655100	2.49974900
H	5.01940000	-1.83833000	3.36860700

CC3b_S_0

C	-0.31396600	0.00003500	-1.22424100
C	1.11838800	-0.00011300	1.23144900
C	1.11838800	-0.00011300	-1.23144900
C	-0.99134900	0.00009000	0.00000000
C	-0.31396600	0.00003500	1.22424100
C	1.78772400	-0.00021400	0.00000000
H	-2.08052700	0.00018900	0.00000000
H	2.87078900	-0.00037300	0.00000000
C	1.80904200	-0.00009700	-2.51862000
C	3.11818700	0.00005300	-5.05671600
C	1.05318700	-0.00003000	-3.72819500
C	3.21864400	-0.00007700	-2.62697500
C	3.85564600	-0.00000700	-3.85378400
C	1.73406000	0.00004700	-4.96885400

H	3.82960200	-0.00006500	-1.72950600
H	4.94379500	-0.00000800	-3.89207000
H	1.14125200	0.00011900	-5.88019600
C	1.80904200	-0.00009700	2.51862000
C	3.11818700	0.00005300	5.05671600
C	3.21864400	-0.00007700	2.62697500
C	1.05318700	-0.00003000	3.72819500
C	1.73406000	0.00004700	4.96885400
C	3.85564600	-0.00000700	3.85378400
H	3.82960200	-0.00006500	1.72950600
H	1.14125200	0.00011900	5.88019600
H	4.94379500	-0.00000800	3.89207000
C	-0.40149700	0.00000500	3.67996700
C	-1.03041200	0.00008200	2.46910700
H	-2.11867800	0.00016800	2.43123600
C	-0.40149700	0.00000500	-3.67996700
C	-1.03041200	0.00008200	-2.46910700
H	-2.11867800	0.00016800	-2.43123600
C	-1.21088000	-0.00000400	4.94341800
C	-2.75899600	-0.00002100	7.30840700
C	-1.59515000	-1.22104500	5.53724500
C	-1.59521100	1.22102800	5.53722600
C	-2.36127200	1.19821800	6.70812900
C	-2.36125000	-1.19824700	6.70813100
H	-2.65236800	2.14415100	7.16374100
H	-2.65235700	-2.14418400	7.16372600
C	-1.21088000	-0.00000400	-4.94341800
C	-2.75899600	-0.00002100	-7.30840700
C	-1.59521100	1.22102800	-5.53722600
C	-1.59515000	-1.22104500	-5.53724500
C	-2.36125000	-1.19824700	-6.70813100
C	-2.36127200	1.19821800	-6.70812900
H	-2.65235700	-2.14418400	-7.16372600
H	-2.65236800	2.14415100	-7.16374100
C	3.82615900	0.00016600	6.38989700
H	4.47157200	0.88169800	6.49807500
H	4.47035600	-0.88215200	6.49894300
H	3.11427600	0.00105500	7.22132100
C	3.82615900	0.00016600	-6.38989700
H	4.47157200	0.88169800	-6.49807500
H	3.11427600	0.00105500	-7.22132100
H	4.47035600	-0.88215200	-6.49894300
C	-1.18920200	2.54523200	4.92983900
H	-0.10033900	2.62096800	4.82192200

H	-1.52761900	3.38042900	5.55130500
H	-1.61337400	2.67549200	3.92678200
C	-1.18913600	-2.54525200	4.92986700
H	-0.10022400	-2.62124500	4.82259600
H	-1.61272500	-2.67523800	3.92653200
H	-1.52812500	-3.38046500	5.55099900
C	-3.61270300	-0.00001400	8.55500100
H	-3.42392300	0.88670900	9.17030900
H	-3.42464800	-0.88722400	9.16982300
H	-4.68255500	0.00050200	8.30403900
C	-1.18913600	-2.54525200	-4.92986700
H	-0.10022400	-2.62124500	-4.82259600
H	-1.52812500	-3.38046500	-5.55099900
H	-1.61272500	-2.67523800	-3.92653200
C	-1.18920200	2.54523200	-4.92983900
H	-0.10033900	2.62096800	-4.82192200
H	-1.61337400	2.67549200	-3.92678200
H	-1.52761900	3.38042900	-5.55130500
C	-3.61270300	-0.00001400	-8.55500100
H	-3.42392300	0.88670900	-9.17030900
H	-4.68255500	0.00050200	-8.30403900
H	-3.42464800	-0.88722400	-9.16982300

BO3b_S_0

C	-1.20330400	-0.08192600	-0.00416700
C	-1.23591900	1.33357800	-0.00661900
C	-2.53290800	2.02771100	-0.01867800
C	-3.72147000	1.25279200	-0.01619300
C	0.00001000	-0.77768300	-0.00014600
C	0.00000600	1.99448800	-0.00000900
C	1.23593300	1.33358100	0.00653400
C	1.20332200	-0.08192200	0.00394400
C	3.72148600	1.25280100	0.01622600
C	2.53292100	2.02771500	0.01869600
H	0.00001100	-1.86166300	-0.00019600
H	0.00000200	3.07695800	0.00006600
C	-4.96965300	1.90582400	-0.03691700
C	-2.64926100	3.43161900	-0.03792400
C	-3.89464400	4.04178300	-0.05463100
C	-5.08632800	3.29253200	-0.05535800
H	-5.87043500	1.29555000	-0.04027500
H	-1.76538600	4.06134000	-0.04139900
H	-3.95027900	5.12871400	-0.06952400
C	4.96966200	1.90583900	0.03713500

C	5.08633100	3.29254500	0.05571700
C	3.89464300	4.04179200	0.05492200
C	2.64926600	3.43162400	0.03805300
H	5.87044800	1.29557200	0.04053200
H	3.95027200	5.12872300	0.06989300
H	1.76538800	4.06134000	0.04147400
B	3.61075400	-0.28770700	0.00279800
O	2.34240000	-0.83564700	0.00441900
O	-2.34238100	-0.83565700	-0.00469700
B	-3.61073000	-0.28771600	-0.00294100
C	4.81624200	-1.30197100	0.00429600
C	5.49228100	-1.62808700	-1.19197900
C	5.24076900	-1.90002000	1.21108900
C	6.56388100	-2.52636500	-1.16333700
C	6.31776200	-2.79174200	1.20576200
C	6.99225500	-3.12152400	0.02666200
H	7.07635400	-2.76798900	-2.09323300
H	6.63868100	-3.23963300	2.14495700
C	-4.81623500	-1.30196300	-0.00445300
C	-5.24123200	-1.89953100	-1.21131700
C	-5.49183000	-1.62851600	1.19195100
C	-6.31823700	-2.79123500	-1.20592900
C	-6.56346600	-2.52675800	1.16336600
C	-6.99229500	-3.12145300	-0.02669800
H	-6.63951200	-3.23876100	-2.14517600
H	-7.07560400	-2.76872100	2.09335800
C	5.05624200	-1.02780500	-2.51324300
H	4.03490300	-1.33402700	-2.77418800
H	5.06322700	0.06874600	-2.48429400
H	5.71311300	-1.34370400	-3.33005300
C	4.53590600	-1.58474900	2.51465100
H	4.50977000	-0.50496600	2.71044700
H	3.49559900	-1.93273600	2.50053700
H	5.03499200	-2.06302900	3.36350100
C	8.13334900	-4.11237700	0.03359100
H	7.77368300	-5.13578900	-0.13972000
H	8.86356000	-3.88950800	-0.75211500
H	8.65895400	-4.11275000	0.99467400
C	-8.13340500	-4.11229000	-0.03359800
H	-7.77355300	-5.13591900	0.13803200
H	-8.86274500	-3.89038400	0.75318400
H	-8.66006900	-4.11147500	-0.99410400
C	-5.05531000	-1.02868100	2.51325900
H	-4.03381900	-1.33485800	2.77365200

H	-5.06246300	0.06788300	2.48471300
H	-5.71179400	-1.34496700	3.33023000
C	-4.53680200	-1.58380800	-2.51500200
H	-4.51051000	-0.50394300	-2.71031600
H	-3.49655900	-1.93202100	-2.50143900
H	-5.03629700	-2.06160200	-3.36388500
C	6.43051000	3.98019900	0.07779900
H	7.24875000	3.25358500	0.07783000
H	6.56197000	4.63210700	-0.79515000
H	6.54361300	4.61131700	0.96839000
C	-6.43051300	3.98018200	-0.07719300
H	-7.24874700	3.25356200	-0.07749400
H	-6.56198300	4.63175900	0.79600400
H	-6.54361700	4.61163700	-0.96754300

CC3c_S_0

C	-1.22445300	-1.69573300	0.00465800
C	1.23158900	-0.26356200	0.00722400
C	-1.23159000	-0.26356000	0.00721200
C	-0.00000200	-2.37269100	0.00412900
C	1.22445100	-1.69573500	0.00466000
C	0.00000000	0.40513200	0.01012800
H	-0.00000200	-3.46177700	0.00317800
H	0.00000000	1.48794600	0.01671300
C	-2.52046100	0.42877900	0.00782300
C	-5.02529300	1.73219400	-0.00518900
C	-3.72724200	-0.33417300	0.00318100
C	-2.61997500	1.83603600	0.00493400
C	-3.84235200	2.50545900	0.00016600
C	-4.96358800	0.35246700	-0.00316700
H	-1.71562600	2.43494600	-0.02380500
H	-5.88105200	-0.22925300	0.00278200
C	2.52046000	0.42877700	0.00785900
C	5.02529400	1.73219000	-0.00509200
C	2.61997600	1.83603400	0.00501600
C	3.72724100	-0.33417600	0.00320200
C	4.96358800	0.35246200	-0.00311200
C	3.84235300	2.50545500	0.00027800
H	1.71562800	2.43494600	-0.02370400
H	5.88105100	-0.22925900	0.00283400
C	3.67927200	-1.78717100	0.00363200
C	2.46785300	-2.41503400	0.00394700
H	2.42808700	-3.50310600	0.00282600
C	-3.67927500	-1.78716800	0.00364600

C	-2.46785700	-2.41503200	0.00396000
H	-2.42809100	-3.50310400	0.00285900
C	4.94384500	-2.59467200	0.00172300
C	7.31251200	-4.13632500	-0.00168100
C	5.53530300	-2.97921100	-1.22038500
C	5.54123400	-2.97552300	1.22210300
C	6.71406600	-3.73851600	1.19744900
C	6.70823400	-3.74216500	-1.19908500
H	7.17277700	-4.02692100	2.14265800
H	7.16236100	-4.03345500	-2.14563000
C	-4.94384900	-2.59466700	0.00177000
C	-7.31251800	-4.13631800	-0.00156700
C	-5.54130100	-2.97536800	1.22216700
C	-5.53524400	-2.97935500	-1.22032000
C	-6.70817700	-3.74230500	-1.19898800
C	-6.71413300	-3.73836200	1.19754500
H	-7.16225700	-4.03370700	-2.14552100
H	-7.17289100	-4.02665400	2.14276600
H	-5.99178300	2.22932100	0.01654800
H	5.99178400	2.22931500	0.01666900
C	-3.90244000	3.98918200	-0.00151200
C	-4.01946600	6.80797300	-0.01036200
C	-4.87236100	4.66998800	-0.75798700
C	-2.99463300	4.75271200	0.75298600
C	-3.05148800	6.14599100	0.74788900
C	-4.92998200	6.06324500	-0.76301300
H	-5.56985600	4.10077200	-1.36768500
H	-2.25647900	4.24620800	1.37021000
H	-2.34397200	6.71583100	1.34610800
H	-5.68337900	6.56851200	-1.36319100
H	-4.06470700	7.89441000	-0.01364500
C	3.90244400	3.98917800	-0.00135100
C	4.01947800	6.80796900	-0.01010100
C	2.99464200	4.75268400	0.75317900
C	4.87236400	4.67000700	-0.75780500
C	4.92998900	6.06326400	-0.76278200
C	3.05150100	6.14596200	0.74813100
H	2.25649000	4.24615900	1.37038800
H	5.56985500	4.10081100	-1.36752500
H	5.68338500	6.56855100	-1.36294600
H	2.34399000	6.71578300	1.34637400
H	4.06472200	7.89440600	-0.01334500
C	-4.92353400	-2.57764600	-2.54388600
H	-4.81356300	-1.48919000	-2.62268400

H	-5.54337300	-2.91710300	-3.37979700
H	-3.92093000	-3.00396800	-2.67062700
C	-4.93618300	-2.56910700	2.54735700
H	-4.83093600	-1.48005700	2.62477600
H	-3.93238400	-2.99126700	2.67844600
H	-5.55786000	-2.90952000	3.38150000
C	-8.56153000	-4.98638600	-0.00325500
H	-9.17794800	-4.79505500	0.88210300
H	-8.31345000	-6.05689100	-0.00135300
H	-9.17403000	-4.79733300	-0.89182500
C	4.93604600	-2.56942900	2.54731300
H	4.83058800	-1.48040400	2.62477700
H	5.55779100	-2.90976000	3.38143800
H	3.93232900	-2.99179000	2.67839300
C	4.92366500	-2.57733200	-2.54393200
H	4.81391200	-1.48885000	-2.62268700
H	3.92097500	-3.00344600	-2.67068000
H	5.54343300	-2.91687700	-3.37986000
C	8.56152300	-4.98639600	-0.00340800
H	9.17792200	-4.79513300	0.88197900
H	9.17404400	-4.79727500	-0.89194900
H	8.31344200	-6.05690000	-0.00159200

BO3c_S_0

C	-1.20377500	-1.49931700	-0.00919900
C	-1.23606100	-0.08363900	-0.01235800
C	-2.53391400	0.61266900	-0.03143200
C	-3.72003200	-0.16860800	-0.02677900
C	-0.00003600	-2.19440200	-0.00023700
C	-0.00002400	0.57638600	0.00029100
C	1.23600700	-0.08365500	0.01263500
C	1.20371200	-1.49933200	0.00898300
C	3.71997600	-0.16865300	0.02747100
C	2.53386300	0.61264600	0.03183200
H	-0.00003500	-3.27834900	-0.00037500
H	-0.00002100	1.65856800	0.00066600
C	-4.96319600	0.49275400	-0.04593100
C	-2.63938400	2.01426800	-0.05205700
C	-3.87944500	2.66012100	-0.06957300
C	-5.05120100	1.87547000	-0.06521200
H	-5.87136500	-0.10475100	-0.05612900
H	-1.74709500	2.63025800	-0.02880400
H	-6.02174000	2.36120000	-0.10740000
C	4.96315000	0.49267200	0.04663100

C	5.05118000	1.87540100	0.06573100
C	3.87944200	2.66005200	0.06976100
C	2.63935300	2.01424200	0.05214700
H	5.87130900	-0.10484700	0.05698200
H	6.02172100	2.36112100	0.10795300
H	1.74709500	2.63025500	0.02843700
B	3.61035900	-1.70782300	0.01568000
O	2.34093400	-2.25497800	0.01417000
O	-2.34100800	-2.25494700	-0.01448200
B	-3.61043300	-1.70778700	-0.01523600
C	4.81620100	-2.72087700	0.02349800
C	5.50160200	-3.04378400	-1.16840500
C	5.23221200	-3.32083300	1.23237600
C	6.57402200	-3.94078900	-1.13332800
C	6.31039900	-4.21106700	1.23336600
C	6.99421900	-4.53761500	0.05877000
H	7.09377900	-4.18001700	-2.05977000
H	6.62493000	-4.66024300	2.17407100
C	-4.81626000	-2.72085400	-0.02314600
C	-5.23124700	-3.32174500	-1.23194700
C	-5.50263800	-3.04291900	1.16841100
C	-6.30941300	-4.21197700	-1.23319000
C	-6.57499100	-3.94002200	1.13308700
C	-6.99419300	-4.53770800	-0.05890800
H	-6.62317400	-4.66181800	-2.17383600
H	-7.09545800	-4.17865800	2.05928000
C	5.07469400	-2.44224000	-2.49212400
H	4.05522500	-2.74822400	-2.76057800
H	5.08150300	-1.34570000	-2.46272200
H	5.73724200	-2.75752100	-3.30453700
C	4.51732900	-3.00911400	2.53133000
H	4.48813200	-1.92978400	2.72922600
H	3.47774400	-3.35882100	2.50904300
H	5.01104300	-3.48816000	3.38284000
C	8.13672200	-5.52672000	0.07245900
H	7.78040700	-6.54971700	-0.10993600
H	8.87473100	-5.29868100	-0.70442200
H	8.65223300	-5.53120400	1.03894500
C	-8.13661600	-5.52689900	-0.07290000
H	-7.77990100	-6.55024800	0.10672500
H	-8.87342900	-5.30056900	0.70559800
H	-8.65363300	-5.52941600	-1.03860000
C	-5.07691800	-2.44045500	2.49210400
H	-4.05712600	-2.74498200	2.76097500

H	-5.08520200	-1.34393700	2.46244600
H	-5.73929500	-2.75643700	3.30438500
C	-4.51535000	-3.01094000	-2.53056100
H	-4.48575500	-1.93173000	-2.72906100
H	-3.47587100	-3.36087700	-2.50731700
H	-5.00858700	-3.49034900	-3.38214300
C	3.96004000	4.14324000	0.09294800
C	3.06096600	4.90516000	0.85832400
C	4.94092400	4.82154300	-0.65025100
C	3.13884600	6.29687400	0.87869800
H	2.31416300	4.39988600	1.46446800
C	5.01764800	6.21330300	-0.63197600
H	5.63130300	4.25313900	-1.26715100
C	4.11712800	6.95707100	0.13287300
H	2.43950500	6.86562300	1.48584300
H	5.77843300	6.71761900	-1.22193700
H	4.17819400	8.04185200	0.14879700
C	-3.96001600	4.14331200	-0.09312800
C	-4.94057000	4.82192300	0.65025500
C	-3.06116800	4.90495600	-0.85903100
C	-5.01720300	6.21366300	0.63159100
H	-5.63074000	4.25373800	1.26759100
C	-3.13895100	6.29668100	-0.87979200
H	-2.31459800	4.39947300	-1.46528700
C	-4.11691100	6.95716100	-0.13382200
H	-5.77773200	6.71823100	1.22166600
H	-2.43976000	6.86518200	-1.48734300
H	-4.17794000	8.04193800	-0.15002000

CC3d_S_0

C	-1.22039100	-0.45039800	-0.00865400
C	1.23404000	0.97875800	0.03444000
C	-1.23404100	0.97876900	0.03438600
C	0.00000200	-1.13234100	-0.02689900
C	1.22039600	-0.45040600	-0.00862300
C	0.00000400	1.64833900	0.04557800
H	-0.00000700	-2.22073000	-0.06140500
H	0.00002400	2.72873600	0.02837900
C	-2.52293800	1.66477700	0.04984000
C	-4.96490100	3.13117700	0.01151000
C	-3.74874000	0.94431500	-0.04853100
C	-2.55228400	3.08724400	0.18637900
C	-3.71812500	3.79063600	0.19118600
C	-4.99695500	1.70434700	-0.13178500

H	-1.62281100	3.62877100	0.31796200
C	2.52293700	1.66476400	0.04995800
C	4.96489200	3.13117500	0.01174100
C	2.55227900	3.08721700	0.18664400
C	3.74873900	0.94431800	-0.04847900
C	4.99694300	1.70436700	-0.13173300
C	3.71811800	3.79061100	0.19153500
H	1.62280000	3.62872300	0.31829600
C	3.68424500	-0.51657600	-0.04236400
C	2.46543800	-1.14339900	-0.02270400
H	2.44075500	-2.23153800	-0.00325400
C	-3.68423800	-0.51657600	-0.04239800
C	-2.46542800	-1.14339500	-0.02273500
H	-2.44074800	-2.23153400	-0.00326100
C	4.85732300	-1.45934700	0.01806000
C	6.94403300	-3.36114200	0.16651200
C	5.28640300	-2.13122800	-1.14665200
C	5.46323100	-1.74892700	1.25977600
C	6.49641800	-2.69067700	1.30896000
C	6.32591900	-3.06377100	-1.05164100
H	6.96379700	-2.90586000	2.26928900
H	6.66161400	-3.56936300	-1.95645400
C	-4.85730700	-1.45936100	0.01805800
C	-6.94400500	-3.36115200	0.16661800
C	-5.46315400	-1.74895800	1.25983900
C	-5.28644300	-2.13121400	-1.14661000
C	-6.32597800	-3.06377800	-1.05153800
C	-6.49630900	-2.69069100	1.30907700
H	-6.66173300	-3.56936100	-1.95632900
H	-6.96364500	-2.90587900	2.26943100
H	-3.70945800	4.87163100	0.31559600
H	3.70944800	4.87158800	0.31609100
C	-6.15982600	3.89082300	-0.04347300
H	-6.08878100	4.96978000	0.08065400
C	-7.37918900	3.28883700	-0.26158000
H	-8.28998800	3.88117500	-0.30618500
C	-7.42514900	1.89335900	-0.44315700
H	-8.37576300	1.40333300	-0.64090600
C	-6.27485200	1.12935900	-0.38084300
H	-6.36615700	0.06639500	-0.54066900
C	6.15981700	3.89082400	-0.04320700
H	6.08879600	4.96976500	0.08107300
C	7.37916200	3.28886100	-0.26147600
H	8.28995900	3.88120400	-0.30606000

C	7.42511100	1.89340600	-0.44326000
H	8.37571500	1.40342200	-0.64115600
C	6.27481800	1.12940400	-0.38097000
H	6.36609300	0.06646200	-0.54096500
C	-4.66450800	-1.83621000	-2.49359000
H	-4.65505300	-0.75989300	-2.70442100
H	-5.21784500	-2.33500000	-3.29575000
H	-3.62197400	-2.17359300	-2.54337700
C	-5.02455300	-1.04790500	2.52487700
H	-5.22399800	0.02981900	2.47353600
H	-3.94771100	-1.16317500	2.69568100
H	-5.55368000	-1.44655500	3.39619700
C	-8.04199400	-4.39532400	0.25195600
H	-8.75061000	-4.16205300	1.05443500
H	-7.63237200	-5.39345400	0.46058500
H	-8.60208800	-4.46446500	-0.68716100
C	5.02474900	-1.04787300	2.52485700
H	5.22474700	0.02976400	2.47374700
H	5.55351900	-1.44693200	3.39620700
H	3.94781900	-1.16262300	2.69544100
C	4.66434900	-1.83625200	-2.49358100
H	4.65488600	-0.75994600	-2.70446100
H	3.62180300	-2.17362200	-2.54323900
H	5.21759400	-2.33508600	-3.29577700
C	8.04196500	-4.39538800	0.25165200
H	8.74713000	-4.16565300	1.05815300
H	8.60602000	-4.46002100	-0.68543600
H	7.63175800	-5.39461400	0.45379300

BO3d_S_0

C	-1.20377500	-1.49931700	-0.00919900
C	-1.23606100	-0.08363900	-0.01235800
C	-2.53391400	0.61266900	-0.03143200
C	-3.72003200	-0.16860800	-0.02677900
C	-0.00003600	-2.19440200	-0.00023700
C	-0.00002400	0.57638600	0.00029100
C	1.23600700	-0.08365500	0.01263500
C	1.20371200	-1.49933200	0.00898300
C	3.71997600	-0.16865300	0.02747100
C	2.53386300	0.61264600	0.03183200
H	-0.00003500	-3.27834900	-0.00037500
H	-0.00002100	1.65856800	0.00066600
C	-4.96319600	0.49275400	-0.04593100
C	-2.63938400	2.01426800	-0.05205700

C	-3.87944500	2.66012100	-0.06957300
C	-5.05120100	1.87547000	-0.06521200
H	-5.87136500	-0.10475100	-0.05612900
H	-1.74709500	2.63025800	-0.02880400
H	-6.02174000	2.36120000	-0.10740000
C	4.96315000	0.49267200	0.04663100
C	5.05118000	1.87540100	0.06573100
C	3.87944200	2.66005200	0.06976100
C	2.63935300	2.01424200	0.05214700
H	5.87130900	-0.10484700	0.05698200
H	6.02172100	2.36112100	0.10795300
H	1.74709500	2.63025500	0.02843700
B	3.61035900	-1.70782300	0.01568000
O	2.34093400	-2.25497800	0.01417000
O	-2.34100800	-2.25494700	-0.01448200
B	-3.61043300	-1.70778700	-0.01523600
C	4.81620100	-2.72087700	0.02349800
C	5.50160200	-3.04378400	-1.16840500
C	5.23221200	-3.32083300	1.23237600
C	6.57402200	-3.94078900	-1.13332800
C	6.31039900	-4.21106700	1.23336600
C	6.99421900	-4.53761500	0.05877000
H	7.09377900	-4.18001700	-2.05977000
H	6.62493000	-4.66024300	2.17407100
C	-4.81626000	-2.72085400	-0.02314600
C	-5.23124700	-3.32174500	-1.23194700
C	-5.50263800	-3.04291900	1.16841100
C	-6.30941300	-4.21197700	-1.23319000
C	-6.57499100	-3.94002200	1.13308700
C	-6.99419300	-4.53770800	-0.05890800
H	-6.62317400	-4.66181800	-2.17383600
H	-7.09545800	-4.17865800	2.05928000
C	5.07469400	-2.44224000	-2.49212400
H	4.05522500	-2.74822400	-2.76057800
H	5.08150300	-1.34570000	-2.46272200
H	5.73724200	-2.75752100	-3.30453700
C	4.51732900	-3.00911400	2.53133000
H	4.48813200	-1.92978400	2.72922600
H	3.47774400	-3.35882100	2.50904300
H	5.01104300	-3.48816000	3.38284000
C	8.13672200	-5.52672000	0.07245900
H	7.78040700	-6.54971700	-0.10993600
H	8.87473100	-5.29868100	-0.70442200
H	8.65223300	-5.53120400	1.03894500

C	-8.13661600	-5.52689900	-0.07290000
H	-7.77990100	-6.55024800	0.10672500
H	-8.87342900	-5.30056900	0.70559800
H	-8.65363300	-5.52941600	-1.03860000
C	-5.07691800	-2.44045500	2.49210400
H	-4.05712600	-2.74498200	2.76097500
H	-5.08520200	-1.34393700	2.46244600
H	-5.73929500	-2.75643700	3.30438500
C	-4.51535000	-3.01094000	-2.53056100
H	-4.48575500	-1.93173000	-2.72906100
H	-3.47587100	-3.36087700	-2.50731700
H	-5.00858700	-3.49034900	-3.38214300
C	3.96004000	4.14324000	0.09294800
C	3.06096600	4.90516000	0.85832400
C	4.94092400	4.82154300	-0.65025100
C	3.13884600	6.29687400	0.87869800
H	2.31416300	4.39988600	1.46446800
C	5.01764800	6.21330300	-0.63197600
H	5.63130300	4.25313900	-1.26715100
C	4.11712800	6.95707100	0.13287300
H	2.43950500	6.86562300	1.48584300
H	5.77843300	6.71761900	-1.22193700
H	4.17819400	8.04185200	0.14879700
C	-3.96001600	4.14331200	-0.09312800
C	-4.94057000	4.82192300	0.65025500
C	-3.06116800	4.90495600	-0.85903100
C	-5.01720300	6.21366300	0.63159100
H	-5.63074000	4.25373800	1.26759100
C	-3.13895100	6.29668100	-0.87979200
H	-2.31459800	4.39947300	-1.46528700
C	-4.11691100	6.95716100	-0.13382200
H	-5.77773200	6.71823100	1.22166600
H	-2.43976000	6.86518200	-1.48734300
H	-4.17794000	8.04193800	-0.15002000

CC3e_S_0

C	-0.00010000	-1.22450400	1.85808100
C	0.00000000	1.23310100	0.42530500
C	0.00000000	-1.23310100	0.42530500
C	0.00000000	0.00000000	2.53435400
C	0.00010000	1.22450400	1.85808100
C	0.00000000	0.00000000	-0.24233200
H	0.00000000	0.00000000	3.62350500
H	0.00000000	0.00000000	-1.32497300

C	0.00007700	-2.51914700	-0.26869000
C	0.00014400	-5.03144600	-1.55682400
C	-0.00022600	-3.73147000	0.49382800
C	0.00049000	-2.61075500	-1.68375700
C	0.00051100	-3.83587800	-2.31596700
C	-0.00019800	-4.97904400	-0.17375400
H	0.00084900	-1.70493000	-2.28333700
H	-0.00043300	-5.88978300	0.41887800
C	-0.00007700	2.51914700	-0.26869000
C	-0.00014400	5.03144600	-1.55682400
C	-0.00049000	2.61075500	-1.68375700
C	0.00022600	3.73147000	0.49382800
C	0.00019800	4.97904400	-0.17375400
C	-0.00051100	3.83587800	-2.31596700
H	-0.00084900	1.70493000	-2.28333700
H	0.00043300	5.88978300	0.41887800
C	0.00048600	3.67807200	1.94871900
C	0.00035200	2.46731600	2.57673200
H	0.00049500	2.42813300	3.66486000
C	-0.00048600	-3.67807200	1.94871900
C	-0.00035200	-2.46731600	2.57673200
H	-0.00049500	-2.42813300	3.66486000
C	0.00084400	4.94046800	2.75998400
C	0.00157100	7.30332500	4.31121000
C	1.22218600	5.53345000	3.14486800
C	-1.22010100	5.53380600	3.14541800
C	-1.19685700	6.70374100	3.91303000
C	1.19966900	6.70335100	3.91247100
H	-2.14263900	7.15904900	4.20502500
H	2.14573400	7.15837800	4.20400900
C	-0.00084400	-4.94046800	2.75998400
C	-0.00157100	-7.30332500	4.31121000
C	-1.22218600	-5.53345000	3.14486800
C	1.22010100	-5.53380600	3.14541800
C	1.19685700	-6.70374100	3.91303000
C	-1.19966900	-6.70335100	3.91247100
H	2.14263900	-7.15904900	4.20502500
H	-2.14573400	-7.15837800	4.20400900
C	-2.54459300	4.92716400	2.73924300
H	-2.62171800	4.82243300	1.65016500
H	-3.37953100	5.54748800	3.08028700
H	-2.67402200	3.92291600	3.16080800
C	2.54627900	4.92627000	2.73819900
H	2.62246600	4.82028000	1.64919000

H	2.67598200	3.92249400	3.16083200
H	3.38155900	5.54690000	3.07784900
C	0.00206400	8.54879900	5.16653400
H	-0.88706400	9.16202200	4.98247700
H	0.88690100	9.16613400	4.97550100
H	0.00694800	8.29634000	6.23601500
C	2.54459300	-4.92716400	2.73924300
H	2.62171800	-4.82243300	1.65016500
H	3.37953100	-5.54748800	3.08028700
H	2.67402200	-3.92291600	3.16080800
C	-2.54627900	-4.92627000	2.73819900
H	-2.62246600	-4.82028000	1.64919000
H	-2.67598200	-3.92249400	3.16083200
H	-3.38155900	-5.54690000	3.07784900
C	-0.00206400	-8.54879900	5.16653400
H	-0.88690100	-9.16613400	4.97550100
H	-0.00694800	-8.29634000	6.23601500
H	0.88706400	-9.16202200	4.98247700
C	-0.00026000	6.16971500	-2.48305600
C	-0.00061400	7.93122800	-4.63727600
C	-0.00069900	5.67294100	-3.80221800
C	0.00000000	7.54600200	-2.23961300
C	-0.00018100	8.42263100	-3.32678700
C	-0.00087400	6.55230600	-4.88069000
H	0.00033400	7.93084900	-1.22214200
H	0.00001700	9.49647200	-3.15430200
H	-0.00120600	6.18097800	-5.90390300
H	-0.00074600	8.62650900	-5.47363200
C	0.00026000	-6.16971500	-2.48305600
C	0.00061400	-7.93122800	-4.63727600
C	0.00000000	-7.54600200	-2.23961300
C	0.00069900	-5.67294100	-3.80221800
C	0.00087400	-6.55230600	-4.88069000
C	0.00018100	-8.42263100	-3.32678700
H	-0.00033400	-7.93084900	-1.22214200
H	0.00120600	-6.18097800	-5.90390300
H	-0.00001700	-9.49647200	-3.15430200
H	0.00074600	-8.62650900	-5.47363200
C	-0.00090000	4.14320700	-3.81420800
C	0.00090000	-4.14320700	-3.81420800
C	-1.26422600	-3.58713800	-4.50563300
H	-1.28473800	-3.87301300	-5.56420600
H	-1.28617400	-2.49187700	-4.45379200
H	-2.17394800	-3.96949100	-4.03096600

C	1.26656500	-3.58747600	-4.50492200
H	1.28762300	-3.87342500	-5.56346300
H	2.17591700	-3.97001900	-4.02970000
H	1.28874700	-2.49221700	-4.45314200
C	-1.26656500	3.58747600	-4.50492200
H	-2.17591700	3.97001900	-4.02970000
H	-1.28874700	2.49221700	-4.45314200
H	-1.28762300	3.87342500	-5.56346300
C	1.26422600	3.58713800	-4.50563300
H	1.28473800	3.87301300	-5.56420600
H	1.28617400	2.49187700	-4.45379200
H	2.17394800	3.96949100	-4.03096600

BO3e_S_0

C	-1.20366800	-1.67021300	0.00103800
C	-1.23759100	-0.25404500	0.00040500
C	-2.53352700	0.44322700	-0.00117100
C	-3.72328300	-0.33936400	0.00353500
C	0.00000400	-2.36472400	-0.00008400
C	0.00000500	0.40517200	-0.00005300
C	1.23760100	-0.25404200	-0.00052800
C	1.20367800	-1.67021200	-0.00119200
C	3.72329300	-0.33936800	-0.00362100
C	2.53353700	0.44322600	0.00108000
H	0.00000900	-3.44868800	-0.00009600
H	-0.00000300	1.48725300	-0.00003200
C	-4.97934000	0.29894600	-0.00274000
C	-2.63466600	1.85237300	-0.00978400
C	-3.87787100	2.45794000	-0.01375700
C	-5.06178000	1.68454400	-0.01062000
H	-5.87795700	-0.31337900	-0.00213900
H	-1.74199000	2.47046200	-0.01398800
C	4.97934600	0.29894500	0.00271600
C	5.06178000	1.68454500	0.01064300
C	3.87787600	2.45794000	0.01376900
C	2.63467300	1.85237300	0.00973100
H	5.87797200	-0.31336600	0.00213100
H	1.74198700	2.47044600	0.01391400
B	3.60814400	-1.87882500	-0.00880400
O	2.34024300	-2.42662500	-0.00355700
O	-2.34023600	-2.42662700	0.00338700
B	-3.60813000	-1.87882400	0.00865700
C	4.81402400	-2.89310300	-0.00910600
C	5.43462100	-3.27797500	-1.21723500

C	5.29457900	-3.43077200	1.20529700
C	6.50811400	-4.17464600	-1.19378000
C	6.37041500	-4.32339100	1.19438600
C	6.99063000	-4.71150100	0.00282500
H	6.97873700	-4.46100700	-2.13299600
H	6.73413600	-4.72538100	2.13879900
C	-4.81402400	-2.89308700	0.00899100
C	-5.29490600	-3.43044100	-1.20540400
C	-5.43431100	-3.27825200	1.21720100
C	-6.37075400	-4.32306600	-1.19442200
C	-6.50780600	-4.17489900	1.19381700
C	-6.99064000	-4.71147300	-0.00280300
H	-6.73473500	-4.72481800	-2.13883500
H	-6.97819300	-4.46147100	2.13308900
C	4.93710700	-2.74115600	-2.54395300
H	3.91666200	-3.08529100	-2.75674200
H	4.91362800	-1.64440100	-2.55560200
H	5.57450100	-3.06938500	-3.37125400
C	4.64886800	-3.05088600	2.52220400
H	4.63648100	-1.96318600	2.66876400
H	3.60709000	-3.39218200	2.56975800
H	5.18173200	-3.49327700	3.36998000
C	8.13232300	-5.70171700	0.00808900
H	7.76307900	-6.73608600	-0.01367800
H	8.78106800	-5.57073700	-0.86465100
H	8.74913500	-5.59874900	0.90774600
C	-8.13230500	-5.70172200	-0.00793200
H	-7.76307000	-6.73603800	0.01645600
H	-8.78241400	-5.56908200	0.86354900
H	-8.74770900	-5.60050900	-0.90874200
C	-4.93644200	-2.74173200	2.54390600
H	-3.91601900	-3.08609000	2.75644300
H	-4.91275700	-1.64498200	2.55571500
H	-5.57373800	-3.06996600	3.37128000
C	-4.64954600	-3.05025100	-2.52239600
H	-4.63703300	-1.96251000	-2.66863100
H	-3.60783400	-3.39169600	-2.57038000
H	-5.18273600	-3.49230700	-3.37014200
C	6.21318800	2.59325000	0.01795900
C	5.73595500	3.91904600	0.02497700
C	7.58479300	2.32957700	0.01863200
C	6.62987400	4.98420200	0.03240700
C	8.47680200	3.40369100	0.02623500
H	7.95507500	1.30763400	0.01342000

C	8.00479300	4.72075700	0.03301100
H	6.27417400	6.01204700	0.03772000
H	9.54703800	3.21508400	0.02690200
H	8.71132700	5.54645700	0.03882000
C	-6.21318900	2.59324600	-0.01786900
C	-5.73595700	3.91903600	-0.02486500
C	-7.58480000	2.32956400	-0.01850400
C	-6.62989100	4.98420100	-0.03222600
C	-8.47681000	3.40366600	-0.02604300
H	-7.95505400	1.30761000	-0.01331100
C	-8.00480000	4.72074100	-0.03279200
H	-6.27419900	6.01204700	-0.03751800
H	-9.54704900	3.21507800	-0.02667900
H	-8.71134800	5.54643000	-0.03855000
C	4.20708900	3.95148300	0.02298500
C	-4.20708900	3.95147600	-0.02291800
C	-3.65700000	4.64116400	-1.29176600
H	-3.95944400	5.69435000	-1.31893700
H	-2.56147400	4.60565400	-1.31020500
H	-4.03038200	4.15529500	-2.19873900
C	-3.66040700	4.65565400	1.23944400
H	-2.56497500	4.61925000	1.26177200
H	-3.96187600	5.70939600	1.25323000
H	-4.03712200	4.18088100	2.15090500
C	3.66044700	4.65569600	-1.23937200
H	2.56501500	4.61928000	-1.26174300
H	3.96190100	5.70944300	-1.25311400
H	4.03720100	4.18095900	-2.15083500
C	3.65696700	4.64112900	1.29184000
H	3.95941100	5.69431300	1.31905900
H	2.56143900	4.60562200	1.31024700
H	4.03032100	4.15522700	2.19880600

CC4a_S_0

C	-1.41210100	3.51561200	-0.00027900
C	-4.18961600	3.05398500	-0.00073200
C	-1.90955200	2.17683900	-0.00040400
C	-2.33175100	4.59077600	-0.00036600
C	-3.69783500	4.36948900	-0.00058800
C	-3.30940600	1.98489800	-0.00064300
H	-1.94416900	5.60564200	-0.00025300
H	-4.38634600	5.21120200	-0.00065800
H	-3.71450900	0.97773600	-0.00078300
H	-5.26164300	2.87127900	-0.00091700

C	-0.96942000	1.05810000	-0.00030000
C	0.96942000	-1.05810000	-0.00030000
C	0.43498400	1.33829400	-0.00021300
C	-1.36025900	-0.28472500	-0.00023700
C	-0.43498400	-1.33829400	-0.00021300
C	1.36025900	0.28472500	-0.00023700
H	-2.41407000	-0.54886300	-0.00017500
H	2.41407000	0.54886300	-0.00017500
C	1.90955200	-2.17683900	-0.00040400
C	3.69783500	-4.36948900	-0.00058800
C	3.30940600	-1.98489800	-0.00064300
C	1.41210100	-3.51561200	-0.00027900
C	2.33175100	-4.59077600	-0.00036600
C	4.18961600	-3.05398500	-0.00073200
H	3.71450900	-0.97773600	-0.00078300
H	1.94416900	-5.60564200	-0.00025300
H	5.26164300	-2.87127900	-0.00091700
H	4.38634600	-5.21120200	-0.00065800
C	0.02230800	3.76187300	-0.00005700
C	0.88283900	2.70304300	-0.00006000
H	1.95606500	2.88725200	0.00007900
C	-0.88283900	-2.70304300	-0.00006000
H	-1.95606500	-2.88725200	0.00007900
C	-0.02230800	-3.76187300	-0.00005700
C	0.56152100	5.16224000	0.00023300
C	1.63161300	7.77800300	0.00077700
C	0.82861600	5.81726800	-1.22088600
C	0.82732700	5.81718500	1.22148900
C	1.35901200	7.11145200	1.19889100
C	1.36025900	7.11137100	-1.19776600
H	1.56670500	7.61041100	2.14487100
H	1.56899400	7.61028800	-2.14356300
C	-0.56152100	-5.16224000	0.00023300
C	-1.63161300	-7.77800300	0.00077700
C	-0.82732700	-5.81718500	1.22148900
C	-0.82861600	-5.81726800	-1.22088600
C	-1.36025900	-7.11137100	-1.19776600
C	-1.35901200	-7.11145200	1.19889100
H	-1.56899400	-7.61028800	-2.14356300
H	-1.56670500	-7.61041100	2.14487100
C	0.55644300	5.13922100	-2.54512900
H	-0.48923700	4.81804400	-2.62438600
H	1.17111300	4.23959400	-2.67126200
H	0.76845600	5.81386100	-3.38070000

C	0.55380900	5.13925800	2.54551800
H	-0.49205600	4.81847000	2.62392800
H	0.76536300	5.81383600	3.38125700
H	1.16805400	4.23941800	2.67218100
C	2.17640600	9.18725500	0.00064400
H	1.36453500	9.92761000	-0.01801600
H	2.80494700	9.37419000	-0.87721500
H	2.77549200	9.38571200	0.89625800
C	-0.55644300	-5.13922100	-2.54512900
H	-1.17111300	-4.23959400	-2.67126200
H	-0.76845600	-5.81386100	-3.38070000
H	0.48923700	-4.81804400	-2.62438600
C	-0.55380900	-5.13925800	2.54551800
H	-1.16805400	-4.23941800	2.67218100
H	0.49205600	-4.81847000	2.62392800
H	-0.76536300	-5.81383600	3.38125700
C	-2.17640600	-9.18725500	0.00064400
H	-2.77549200	-9.38571200	0.89625800
H	-1.36453500	-9.92761000	-0.01801600
H	-2.80494700	-9.37419000	-0.87721500

BO4a_S_0

C	3.91402400	4.17890000	-0.00136400
C	4.27916500	2.83886700	0.00579300
C	3.31171300	1.81555300	0.01192900
C	1.93513500	2.16745800	0.00650600
C	1.57769900	3.52959300	-0.00149900
C	2.55428400	4.51759400	-0.00449800
C	0.93574600	1.08977100	0.00665000
C	1.35768200	-0.26076100	0.00632000
C	0.44888600	-1.31131800	0.00634500
H	0.85962000	-2.31412100	0.00739100
C	-0.93574300	-1.08975900	0.00668600
C	-1.35767800	0.26077300	0.00632000
C	-0.44888300	1.31133000	0.00631000
H	4.67112900	4.95821200	-0.00562300
H	5.33029800	2.56156700	0.00617500
H	0.53399200	3.82630900	-0.00571500
H	2.25586600	5.56290800	-0.01056200
H	-0.85961700	2.31413300	0.00732700
C	-1.93513200	-2.16744700	0.00658000
C	-1.57769500	-3.52958200	-0.00141000
C	-2.55428100	-4.51758300	-0.00436800
C	-3.91402000	-4.17888800	-0.00120700

C	-4.27916100	-2.83885500	0.00593200
C	-3.31171000	-1.81554100	0.01202300
H	-0.53398900	-3.82629800	-0.00564900
H	-2.25586300	-5.56289700	-0.01042100
H	-4.67112600	-4.95820000	-0.00543200
H	-5.33029400	-2.56155500	0.00633300
B	3.70459300	0.31988900	0.01540200
O	2.68367300	-0.60686700	0.00769900
B	-3.70458900	-0.31987800	0.01546200
O	-2.68367000	0.60687900	0.00770200
C	5.17637100	-0.24254900	0.01028900
C	5.91367800	-0.36000200	1.20840200
C	5.78405000	-0.63069100	-1.20440000
C	7.22320600	-0.85130500	1.17477100
C	7.09499300	-1.11562900	-1.20387400
C	7.83256800	-1.23712300	-0.02207400
H	7.78102000	-0.93400700	2.10622700
H	7.55294600	-1.40401100	-2.14880400
C	-5.17636900	0.24255500	0.01036800
C	-5.78414500	0.63052500	-1.20432800
C	-5.91358400	0.36016900	1.20852200
C	-7.09508900	1.11546200	-1.20376600
C	-7.22311500	0.85146700	1.17492600
C	-7.83257200	1.23711700	-0.02192400
H	-7.55311200	1.40372300	-2.14869900
H	-7.78085000	0.93430800	2.10641600
C	-5.29396600	-0.02231100	2.53739000
H	-4.89100600	-1.04237000	2.52087400
H	-4.46251600	0.64552100	2.79815000
H	-6.02664700	0.03296100	3.34894800
C	-5.02432700	0.52593300	-2.51096500
H	-4.15603300	1.19653800	-2.52377100
H	-4.64579600	-0.49055200	-2.67935600
H	-5.65917000	0.78787700	-3.36347100
C	-9.23824400	1.79164700	-0.03713000
H	-9.23466900	2.88903400	0.01251600
H	-9.77057300	1.51246400	-0.95320500
H	-9.82126000	1.43040100	0.81683200
C	5.02412800	-0.52629200	-2.51099200
H	4.15599200	-1.19710400	-2.52373500
H	4.64534500	0.49010000	-2.67937700
H	5.65898000	-0.78810100	-3.36353300
C	5.29415100	0.02259900	2.53727700
H	4.89066700	1.04244400	2.52052400

H	4.46309700	-0.64557800	2.79842300
H	6.02702900	-0.03204900	3.34870000
C	9.23823800	-1.79165800	-0.03730700
H	9.23467200	-2.88902400	0.01281600
H	9.77039300	-1.51287000	-0.95360300
H	9.82141600	-1.43004700	0.81638900

CC4b_S_0

C	-4.45243900	3.63111600	-0.00149700
C	-4.62719100	2.25565300	-0.00155000
C	-3.53740200	1.35381300	-0.00102800
C	-2.20818800	1.87003300	-0.00060500
C	-2.04794500	3.27367700	-0.00056600
C	-3.13282300	4.12959300	-0.00096400
C	-1.07375000	0.95169200	-0.00025000
C	-1.33011700	-0.45737400	-0.00030600
C	-0.26186400	-1.36455300	0.00001500
H	-0.50804200	-2.42183600	-0.00003200
C	1.07378900	-0.95179100	0.00039400
C	1.33015500	0.45727500	0.00042400
C	0.26190100	1.36445300	0.00010800
H	-5.63364500	1.84715100	-0.00204700
H	-1.05114600	3.70179900	-0.00020400
H	-2.96628200	5.20469300	-0.00089300
H	0.50808100	2.42173500	0.00012600
C	2.20822600	-1.87013000	0.00079500
C	2.04798700	-3.27377400	0.00087400
C	3.13286700	-4.12968700	0.00133600
C	4.45248400	-3.63120900	0.00183000
C	4.62723300	-2.25574700	0.00177100
C	3.53744100	-1.35390800	0.00117000
H	1.05118900	-3.70189800	0.00056300
H	2.96632800	-5.20478800	0.00135200
H	5.63368400	-1.84723800	0.00225500
C	-5.14896700	-0.65040600	-0.00093000
C	-5.79923400	-0.92864100	-1.22095100
C	-5.80093200	-0.92475400	1.22034100
C	-7.08438300	-1.48184900	-1.19756500
C	-7.08516700	-1.47742500	1.19780000
C	-7.74413900	-1.76862100	-0.00019600
H	-7.58171000	-1.69299300	-2.14253100
H	-7.58342800	-1.68517100	2.14318100
C	5.14899200	0.65034400	0.00085200
C	5.79923700	0.92886000	1.22076500

C	5.80090500	0.92458200	-1.22052500
C	7.08434300	1.48227100	1.19724900
C	7.08502700	1.47741400	-1.19811500
C	7.74401700	1.76890400	-0.00014900
H	7.58163700	1.69368100	2.14216800
H	7.58323500	1.68509800	-2.14354200
C	-9.11888000	-2.39620500	0.00288800
H	-9.05962300	-3.48567600	0.12955200
H	-9.73505700	-2.01022300	0.82267200
H	-9.64849600	-2.20663400	-0.93671200
C	-5.13018500	-0.63932700	-2.54658800
H	-4.82488500	0.41068300	-2.62355300
H	-4.22305800	-1.24027700	-2.68094200
H	-5.80512800	-0.85757100	-3.38007200
C	-5.13221000	-0.63098300	2.54514000
H	-4.22375500	-1.22968400	2.68049700
H	-4.82907600	0.41984600	2.61958700
H	-5.80649600	-0.84872700	3.37928100
C	-5.62961500	4.57677600	-0.00195800
H	-5.61664300	5.23057700	-0.88335700
H	-6.57917800	4.03297100	-0.00268000
H	-5.61771000	5.23008700	0.87982400
C	5.62966000	-4.57686900	0.00239300
H	5.61775900	-5.23026900	-0.87932400
H	6.57922200	-4.03306300	0.00306600
H	5.61668300	-5.23058000	0.88385700
C	5.13210700	0.63060300	-2.54523600
H	4.82870800	-0.42016300	-2.61940500
H	4.22379900	1.22950200	-2.68074100
H	5.80643600	0.84797000	-3.37944100
C	5.13025800	0.63979900	2.54649800
H	4.22347100	1.24122300	2.68105800
H	4.82442200	-0.41005000	2.62345000
H	5.80545500	0.85764900	3.37988000
C	9.11877100	2.39645100	-0.00356300
H	9.05994800	3.48493800	-0.13852300
H	9.64546500	2.21374000	0.93901800
H	9.73752400	2.00444000	-0.81856700
C	3.75981300	0.08401000	0.00095300
C	2.68689800	0.92623900	0.00067700
C	-2.68686200	-0.92633700	-0.00062000
C	-3.75977500	-0.08410900	-0.00091600
H	-2.85285700	-2.00156100	-0.00051400
H	2.85289200	2.00146200	0.00051700

BO4b_S_0

C	-4.23179800	-3.89026500	-0.00006600
C	-4.47505100	-2.51936100	0.00829000
C	-3.43613000	-1.56852000	0.01650400
C	-2.08998400	-2.01644300	0.01146100
C	-1.84052400	-3.40302300	0.00246500
C	-2.88830400	-4.31133200	-0.00207600
C	-1.01346100	-1.01803100	0.01206200
C	-1.33446100	0.36009900	0.01146500
C	-0.35115800	1.34107200	0.01164100
H	-0.68701800	2.37155100	0.01262000
C	1.01346900	1.01805600	0.01200500
C	1.33447000	-0.36007500	0.01149900
C	0.35116700	-1.34104700	0.01172900
H	-5.50260000	-2.16147100	0.00720100
H	-0.82327900	-3.78106400	-0.00126600
H	-2.66503000	-5.37652100	-0.00894200
H	0.68702600	-2.37152600	0.01277100
C	2.08999300	2.01646500	0.01134800
C	1.84053100	3.40304700	0.00220600
C	2.88830800	4.31135500	-0.00239100
C	4.23180700	3.89028900	-0.00028300
C	4.47505900	2.51938800	0.00817900
C	3.43613700	1.56854400	0.01645600
H	0.82328400	3.78108200	-0.00162200
H	2.66503800	5.37654400	-0.00939200
H	5.50260600	2.16149300	0.00712300
B	-3.71838100	-0.04814000	0.02001000
O	-2.63184600	0.80122600	0.01218400
B	3.71839200	0.04816500	0.02006100
O	2.63185300	-0.80119900	0.01226100
C	-5.14195900	0.62797900	0.01128900
C	-5.88445600	0.77969600	1.20268300
C	-5.69753600	1.09412200	-1.20078300
C	-7.14705700	1.38094600	1.16461300
C	-6.96304700	1.68833000	-1.20493900
C	-7.70422600	1.84531700	-0.02996600
H	-7.70854800	1.49084500	2.09109500
H	-7.38092600	2.03673100	-2.14819300
C	5.14196000	-0.62796900	0.01135600
C	5.69738900	-1.09439100	-1.20069900
C	5.88459300	-0.77944600	1.20267700
C	6.96287600	-1.68860900	-1.20488600

C	7.14720300	-1.38071400	1.16457300
C	7.70421300	-1.84534600	-0.02995900
H	7.38064000	-2.03720600	-2.14812100
H	7.70881500	-1.49039800	2.09100500
C	-9.05562900	2.52137000	-0.04719900
H	-8.95969600	3.60752100	0.08661700
H	-9.57475100	2.35809600	-0.99802400
H	-9.69963700	2.15221700	0.75840800
C	-5.31911200	0.31595500	2.52986700
H	-5.02219000	-0.73947200	2.49956900
H	-4.42604500	0.89087900	2.80755600
H	-6.04981300	0.43398200	3.33655500
C	-4.93211300	0.95495800	-2.50100800
H	-3.99815500	1.52991100	-2.47912300
H	-4.65989800	-0.08954900	-2.70095200
H	-5.52262800	1.31109200	-3.35122700
C	-5.35592200	-4.89826900	-0.00861900
H	-5.31018900	-5.55718600	0.86775100
H	-6.33257600	-4.40472100	-0.00496500
H	-5.30970700	-5.54262900	-0.89572200
C	5.35592600	4.89829800	-0.00871400
H	5.31103900	5.55614400	0.86851400
H	6.33258000	4.40474100	-0.00655800
H	5.30886100	5.54374200	-0.89497400
C	5.31939000	-0.31556700	2.52987200
H	5.02127900	0.73950600	2.49914000
H	4.42710000	-0.89134100	2.80831200
H	6.05060700	-0.43235300	3.33627400
C	4.93181800	-0.95546200	-2.50086100
H	3.99783300	-1.53036200	-2.47874500
H	4.65963400	0.08901800	-2.70099300
H	5.52221700	-1.31180300	-3.35107500
C	9.05561400	-2.52139900	-0.04729800
H	8.95949000	-3.60788400	0.08363700
H	9.57586400	-2.35576900	-0.99710800
H	9.69868200	-2.15428100	0.75997700

CC4c_S_0

C	-1.22419300	3.54665700	-0.02225000
C	-4.00383700	3.39669700	-0.57807000
C	-1.77574600	2.29693600	-0.40404000
C	-2.08419000	4.67339700	0.16913400
C	-3.42977300	4.58568500	-0.04187900
C	-3.16773900	2.25733500	-0.82004300

H	-1.64192200	5.60514900	0.50886100
H	-4.07608700	5.44083000	0.14591700
C	-0.91370600	1.12091000	-0.35241700
C	0.91370600	-1.12091000	-0.35241700
C	0.50725900	1.30581900	-0.23378900
C	-1.37212700	-0.20215000	-0.26753900
C	-0.50725900	-1.30581900	-0.23378900
C	1.37212700	0.20215000	-0.26753900
H	-2.43141300	-0.39906500	-0.14522900
H	2.43141300	0.39906500	-0.14522900
C	1.77574600	-2.29693600	-0.40404000
C	3.42977300	-4.58568500	-0.04187900
C	3.16773900	-2.25733500	-0.82004300
C	1.22419300	-3.54665700	-0.02225000
C	2.08419000	-4.67339700	0.16913400
C	4.00383700	-3.39669700	-0.57807000
H	1.64192200	-5.60514900	0.50886100
H	4.07608700	-5.44083000	0.14591700
C	0.20672100	3.69908900	0.17168700
C	1.02521400	2.61859100	0.00252900
H	2.10279900	2.73824700	0.10109900
C	-1.02521400	-2.61859100	0.00252900
H	-2.10279900	-2.73824700	0.10109900
C	-0.20672100	-3.69908900	0.17168700
C	0.80085400	5.03240500	0.52151200
C	1.97572400	7.51512900	1.18778200
C	1.19331900	5.92535100	-0.49856100
C	0.99383300	5.38319000	1.87452400
C	1.57799000	6.61718000	2.18234000
C	1.77574600	7.14785200	-0.14639200
H	1.72770100	6.88089400	3.22873300
H	2.08225000	7.83083800	-0.93801400
C	-0.80085400	-5.03240500	0.52151200
C	-1.97572400	-7.51512900	1.18778200
C	-0.99383300	-5.38319000	1.87452400
C	-1.19331900	-5.92535100	-0.49856100
C	-1.77574600	-7.14785200	-0.14639200
C	-1.57799000	-6.61718000	2.18234000
H	-2.08225000	-7.83083800	-0.93801400
H	-1.72770100	-6.88089400	3.22873300
C	0.99696900	5.57863200	-1.95752000
H	-0.05841500	5.38450900	-2.18513300
H	1.54920000	4.67287400	-2.23575800
H	1.33735500	6.39500800	-2.60239900

C	0.58350500	4.44623600	2.98855500
H	-0.47901200	4.18192400	2.92107700
H	0.75734400	4.90320500	3.96793300
H	1.14324500	3.50382800	2.94960300
C	2.57777100	8.85462000	1.54281800
H	1.80558000	9.63410200	1.60365700
H	3.30562700	9.17891800	0.79057200
H	3.08386800	8.82227800	2.51389000
C	-0.99696900	-5.57863200	-1.95752000
H	-1.54920000	-4.67287400	-2.23575800
H	-1.33735500	-6.39500800	-2.60239900
H	0.05841500	-5.38450900	-2.18513300
C	-0.58350500	-4.44623600	2.98855500
H	-1.14324500	-3.50382800	2.94960300
H	0.47901200	-4.18192400	2.92107700
H	-0.75734400	-4.90320500	3.96793300
C	-2.57777100	-8.85462000	1.54281800
H	-3.08386800	-8.82227800	2.51389000
H	-1.80558000	-9.63410200	1.60365700
H	-3.30562700	-9.17891800	0.79057200
C	-5.37673300	3.35624100	-0.92864900
H	-5.99146000	4.22693600	-0.70813500
C	-5.91926600	2.26013100	-1.56425400
H	-6.97198400	2.24490900	-1.83596900
C	-3.74821700	1.17918300	-1.54471900
H	-3.12170300	0.35890700	-1.87218200
C	-5.08232000	1.17753000	-1.90380500
H	-5.48304600	0.34003300	-2.47024100
C	3.74821700	-1.17918300	-1.54471900
H	3.12170300	-0.35890700	-1.87218200
C	5.08232000	-1.17753000	-1.90380500
H	5.48304600	-0.34003300	-2.47024100
C	5.91926600	-2.26013100	-1.56425400
H	6.97198400	-2.24490900	-1.83596900
C	5.37673300	-3.35624100	-0.92864900
H	5.99146000	-4.22693600	-0.70813500

BO4c_S_0

C	-1.01076800	-1.04457800	-0.02899000
C	-1.32525300	0.32010500	0.19527400
C	-0.35175200	1.30960300	0.26045300
H	-0.69611600	2.30094100	0.51774700
C	1.00989600	1.04248900	0.03523100
C	1.32468000	-0.32224900	-0.18677600

C	0.35118800	-1.31131700	-0.25415600
H	0.69635900	-2.30249500	-0.51022900
B	-3.69038400	-0.05817700	0.04308800
O	-2.61536100	0.75251100	0.33493400
B	3.68740400	0.05234600	-0.00497600
O	2.61571600	-0.75570200	-0.31644800
C	-5.11582100	0.61309500	0.08160500
C	-5.97531000	0.45313700	1.19054500
C	-5.55519700	1.39111500	-1.01285000
C	-7.23636400	1.05869400	1.18677200
C	-6.82353000	1.97875000	-0.98685300
C	-7.68025500	1.82730000	0.10737600
H	-7.88806000	0.92721800	2.04906500
H	-7.15131100	2.56876000	-1.84137200
C	5.11351200	-0.61733400	-0.03363000
C	5.77112800	-0.85379000	-1.26200100
C	5.75791700	-0.99048800	1.16576400
C	7.03947000	-1.44034300	-1.27103300
C	7.02751600	-1.57773300	1.12142900
C	7.68580900	-1.81299100	-0.08786000
H	7.53735800	-1.60908800	-2.22469300
H	7.51262500	-1.85941400	2.05460600
C	-9.03483000	2.49676300	0.13356300
H	-8.96893100	3.51446800	0.54187900
H	-9.46149800	2.57948400	-0.87209000
H	-9.74403800	1.94294800	0.75821400
C	-5.54453000	-0.35899900	2.39528000
H	-5.34347400	-1.40452400	2.13078000
H	-4.62518000	0.04034300	2.84197900
H	-6.31638600	-0.35576500	3.17153700
C	-4.66281600	1.59349900	-2.22078900
H	-3.74607600	2.13428800	-1.95533900
H	-4.35322100	0.63716600	-2.66222400
H	-5.17346000	2.16663700	-3.00122100
C	5.07932900	-0.78606700	2.50543000
H	4.75236800	0.25144500	2.64534000
H	4.18632500	-1.41748000	2.60223600
H	5.74986900	-1.03793000	3.33337300
C	5.11337000	-0.47374400	-2.57276200
H	4.19000300	-1.04247100	-2.73674500
H	4.84267300	0.58978500	-2.59608900
H	5.77799100	-0.66449800	-3.42142300
C	9.04525300	-2.47204300	-0.12181600
H	8.96298600	-3.54474300	-0.34335600

H	9.68565900	-2.03275700	-0.89511200
H	9.56133200	-2.37663000	0.83933500
C	-1.59697200	-6.30635600	0.00066200
C	-2.78140500	-5.74414200	-0.41783500
C	-2.97169000	-4.33766200	-0.40849400
C	-1.88681400	-3.47519000	-0.02908000
C	-0.70786500	-4.10009300	0.46631800
C	-0.56528300	-5.47215500	0.48117300
H	-5.05077400	-4.45478900	-1.00616600
H	-1.46892200	-7.38530400	-0.00197000
H	-3.60850600	-6.37388100	-0.73676400
C	-4.24654000	-3.78427700	-0.71276600
C	-2.09281400	-2.04199600	-0.08997000
H	0.07707500	-3.49512500	0.89949500
H	0.34382700	-5.91190100	0.88193000
C	-3.40370200	-1.54921100	-0.24542000
C	-4.46584200	-2.44129800	-0.56570800
H	-5.46015900	-2.02798600	-0.71118500
C	3.40186000	1.54900600	0.25791700
C	2.09210600	2.04026000	0.08899500
C	1.88664100	3.47174800	-0.00088600
C	2.97313800	4.34142400	0.35683000
C	4.24941800	3.79403000	0.66509300
C	4.46702200	2.44791100	0.54762700
H	-0.08177300	3.47336500	-0.91976800
C	0.70558300	4.08695200	-0.50350100
C	2.78337100	5.74795400	0.33809100
H	5.05679000	4.47067900	0.93482300
H	5.46358900	2.03884600	0.68929800
C	1.59726500	6.30189100	-0.08639300
C	0.56314100	5.45837800	-0.54516300
H	3.61205600	6.38374400	0.64046500
H	1.46939900	7.38070300	-0.10499600
H	-0.34790100	5.89035600	-0.94998900

CC4d_S_0

C	-1.21060900	-0.76838900	-0.00010000
C	-1.24159900	0.65927400	-0.00002200
C	-0.04323200	1.38561100	0.00003800
H	-0.11985900	2.46843900	0.00009400
C	1.21061000	0.76839100	0.00003400
C	1.24160000	-0.65927100	0.00000200
C	0.04323300	-1.38560900	-0.00006900
H	0.11985900	-2.46843600	-0.00008700

C	-4.98349000	1.47147900	0.00001200
C	-5.58549200	1.84204700	1.22096000
C	-5.58551700	1.84223800	-1.22086700
C	-6.77350700	2.58025500	1.19779500
C	-6.77353300	2.58043600	-1.19756300
C	-7.38247100	2.96434100	0.00015300
H	-7.23534800	2.86038400	2.14288200
H	-7.23540300	2.86069900	-2.14259500
C	4.98349100	-1.47147800	0.00008700
C	5.58546200	-1.84216700	-1.22083900
C	5.58554500	-1.84211800	1.22098700
C	6.77347800	-2.58037100	-1.19763200
C	6.77355600	-2.58032900	1.19772600
C	7.38246700	-2.96434700	0.00003300
H	7.23530400	-2.86058200	-2.14270100
H	7.23543900	-2.86051300	2.14277600
C	-8.64865900	3.78940500	0.00022600
H	-8.42381400	4.86458200	0.00030100
H	-9.25956000	3.58697000	-0.88611800
H	-9.25954100	3.58684300	0.88655400
C	-4.96920000	1.45277300	2.54655600
H	-4.82644200	0.36854500	2.62237800
H	-3.98208600	1.90991600	2.68341200
H	-5.60455300	1.76904700	3.37959400
C	-4.96927100	1.45314300	-2.54653800
H	-3.98225300	1.91047100	-2.68346800
H	-4.82632600	0.36894500	-2.62242900
H	-5.60475800	1.76934900	-3.37950100
C	4.96931600	-1.45292100	2.54663500
H	4.82654700	-0.36869900	2.62251900
H	3.98221500	-1.91008500	2.68352000
H	5.60471700	-1.76922900	3.37962300
C	4.96915400	-1.45299700	-2.54646000
H	3.98212000	-1.91030000	-2.68335800
H	4.82622400	-0.36879300	-2.62229000
H	5.60459000	-1.76917300	-3.37947200
C	8.64865200	-3.78941700	0.00000600
H	8.42380200	-4.86459200	0.00018600
H	9.25944200	-3.58704500	-0.88642800
H	9.25964600	-3.58679600	0.88624500
C	-6.22159200	-4.96680900	-0.00041900
C	-6.21215500	-3.59327300	-0.00033000
C	-4.98230000	-2.87320000	-0.00027900
C	-3.74894700	-3.60712900	-0.00032200

C	-3.79675900	-5.03145500	-0.00041300
C	-4.99995300	-5.69416100	-0.00046000
H	-5.86022100	-0.90865200	-0.00015700
H	-7.16513800	-5.50583400	-0.00045600
H	-7.14412600	-3.03292900	-0.00029700
C	-4.92866500	-1.46709400	-0.00018600
C	-2.53412400	-2.89452100	-0.00026900
H	-2.86095600	-5.58572700	-0.00044300
H	-5.02401200	-6.78054800	-0.00052900
C	-2.47895700	-1.50377400	-0.00017800
C	-3.72299200	-0.76756100	-0.00014200
C	2.47895900	1.50377600	0.00007100
C	2.53412500	2.89452300	0.00007800
C	3.74894900	3.60713100	0.00013300
C	4.98230200	2.87320200	0.00018600
C	4.92866600	1.46709600	0.00018000
C	3.72299300	0.76756400	0.00011600
H	2.86095900	5.58573000	0.00010500
C	3.79676200	5.03145800	0.00014200
C	6.21215700	3.59327400	0.00024600
H	5.86022300	0.90865400	0.00022300
C	6.22159400	4.96681100	0.00025100
C	4.99995600	5.69416300	0.00019900
H	7.14412800	3.03293000	0.00028700
H	7.16514100	5.50583500	0.00029800
H	5.02401500	6.78055000	0.00020700
H	1.61694300	3.47530500	0.00004100
H	-1.61694100	-3.47530200	-0.00030200
C	-3.70276900	0.69101300	-0.00005800
C	-2.50852400	1.34378700	0.00000500
C	2.50852400	-1.34378500	0.00005000
C	3.70277000	-0.69101100	0.00011700
H	-2.49600300	2.43166200	0.00009000
H	2.49600300	-2.43165900	0.00003000

BO4d_S_0

C	-1.16995400	-0.83477600	-0.00610300
C	-1.25665500	0.57647500	-0.00240100
C	-0.12271600	1.37828400	-0.00413900
H	-0.28180700	2.45034900	-0.00135400
C	1.16998600	0.83482400	-0.00605600
C	1.25668400	-0.57642700	-0.00245200
C	0.12274500	-1.37823500	-0.00423500
H	0.28183800	-2.45030100	-0.00154300

B	-3.67590600	0.58743900	0.01854400
O	-2.46128100	1.23696500	0.00618700
B	3.67593700	-0.58739500	0.01850700
O	2.46130900	-1.23691800	0.00605900
C	-4.96774600	1.48928700	0.03542000
C	-5.63715300	1.77287600	1.24559300
C	-5.48152300	2.02104600	-1.16819800
C	-6.79193200	2.56268300	1.23380500
C	-6.63877500	2.80478200	-1.14561200
C	-7.30942100	3.09003100	0.04780800
H	-7.29964400	2.77113400	2.17421300
H	-7.02782400	3.20113800	-2.08230400
C	4.96774600	-1.48929000	0.03539900
C	5.48176000	-2.02067400	-1.16828000
C	5.63685100	-1.77333300	1.24563100
C	6.63896200	-2.80449200	-1.14569100
C	6.79158300	-2.56320300	1.23384400
C	7.30931400	-3.09018300	0.04778500
H	7.02819500	-3.20057400	-2.08242200
H	7.29905900	-2.77200600	2.17430100
C	-8.54277400	3.96344200	0.05581500
H	-8.27705400	5.02867300	0.09282100
H	-9.14686500	3.81353000	-0.84592400
H	-9.17510300	3.75634900	0.92576300
C	-5.10684000	1.24531200	2.56340800
H	-4.98420000	0.15545800	2.54641000
H	-4.12390300	1.67373900	2.79869500
H	-5.77972400	1.49183600	3.39096700
C	-4.78561000	1.74974600	-2.48641400
H	-3.77811100	2.18374300	-2.50293100
H	-4.67351700	0.67369300	-2.67184100
H	-5.34410700	2.17387800	-3.32695500
C	5.10632800	-1.24605400	2.56347700
H	4.98484100	-0.15605500	2.54707400
H	4.12281700	-1.67359200	2.79795000
H	5.77850200	-1.49376800	3.39125800
C	4.78613000	-1.74891700	-2.48655300
H	3.77861400	-2.18286700	-2.50341600
H	4.67411600	-0.67280100	-2.67165600
H	5.34477500	-2.17280300	-3.32711900
C	8.54260100	-3.96368600	0.05579100
H	8.27680200	-5.02889600	0.09283000
H	9.14668900	-3.81383700	-0.84595900
H	9.17496000	-3.75662400	0.92572700

C	-5.99085700	-5.25346400	-0.07153500
C	-6.03474000	-3.87919700	-0.04883600
C	-4.83601200	-3.11394000	-0.03576100
C	-3.57249500	-3.79386500	-0.04701000
C	-3.56395300	-5.21724200	-0.07079900
C	-4.74163200	-5.92737300	-0.08253200
H	-5.78909500	-1.17791600	-0.00622000
H	-6.91161500	-5.83010700	-0.08123300
H	-6.98768200	-3.35548100	-0.04036000
C	-4.83487600	-1.70077500	-0.01262900
C	-2.37913700	-3.03558100	-0.03608500
H	-2.60787000	-5.73524600	-0.07948500
H	-4.72039000	-7.01372800	-0.10049300
C	-2.39638600	-1.64802600	-0.01258800
C	-3.66097500	-0.96033000	0.00099700
C	2.39642200	1.64807500	-0.01250000
C	2.37917700	3.03563000	-0.03594700
C	3.57253700	3.79391200	-0.04683700
C	4.83605200	3.11398200	-0.03561400
C	4.83491100	1.70081500	-0.01254600
C	3.66100700	0.96037300	0.00105100
H	2.60791900	5.73529700	-0.07923600
C	3.56400100	5.21728900	-0.07056900
C	6.03478300	3.87923600	-0.04865300
H	5.78912800	1.17795700	-0.00616700
C	5.99090400	5.25350500	-0.07129400
C	4.74168200	5.92741700	-0.08226800
H	6.98772400	3.35551700	-0.04019700
H	6.91166400	5.83014500	-0.08096400
H	4.72044300	7.01377300	-0.10018400
H	1.43955100	3.58011400	-0.04862100
H	-1.43951000	-3.58006200	-0.04876100

CC4e_S_0

C	-0.81810400	3.70201500	-0.00011300
C	-3.63016600	3.68606400	0.00160200
C	-1.52938600	2.45945200	0.00058000
C	-1.53792500	4.92020900	-0.00005900
C	-2.92217000	4.91285000	0.00074000
C	-2.94663800	2.48864500	0.00154500
H	-0.98461600	5.85541800	-0.00058600
C	-0.78378400	1.20422700	0.00037500
C	0.78378400	-1.20422700	0.00037500
C	0.64826100	1.24909000	0.00016400

C	-1.38780100	-0.05827200	0.00020400
C	-0.64826100	-1.24909000	0.00016400
C	1.38780100	0.05827200	0.00020400
H	-2.47049000	-0.14812400	-0.00009600
H	2.47049000	0.14812400	-0.00009600
C	1.52938600	-2.45945200	0.00058000
C	2.92217000	-4.91285000	0.00074000
C	2.94663800	-2.48864500	0.00154500
C	0.81810400	-3.70201500	-0.00011300
C	1.53792500	-4.92020900	-0.00005900
C	3.63016600	-3.68606400	0.00160200
H	0.98461600	-5.85541800	-0.00058600
C	0.63766000	3.70660000	-0.00067000
C	1.31388000	2.52155300	-0.00036000
H	2.40285600	2.52789800	-0.00061500
C	-1.31388000	-2.52155300	-0.00036000
H	-2.40285600	-2.52789800	-0.00061500
C	-0.63766000	-3.70660000	-0.00067000
C	1.39792900	5.00050300	-0.00147900
C	2.86000600	7.41961300	-0.00316900
C	1.76352500	5.60523900	-1.22301500
C	1.76360500	5.60680900	1.21910000
C	2.48894000	6.80339000	1.19548200
C	2.48880800	6.80173700	-1.20107300
H	2.77184300	7.26472900	2.14109700
H	2.77165900	7.26180400	-2.14734100
C	-1.39792900	-5.00050300	-0.00147900
C	-2.86000600	-7.41961300	-0.00316900
C	-1.76360500	-5.60680900	1.21910000
C	-1.76352500	-5.60523900	-1.22301500
C	-2.48880800	-6.80173700	-1.20107300
C	-2.48894000	-6.80339000	1.19548200
H	-2.77165900	-7.26180400	-2.14734100
H	-2.77184300	-7.26472900	2.14109700
C	1.38780100	4.97714400	-2.54643000
H	0.30387800	4.83033800	-2.62744300
H	1.84801600	3.98909100	-2.66858600
H	1.70854400	5.60608000	-3.38298700
C	1.38780100	4.98065800	2.54340800
H	0.30367300	4.83575800	2.62532400
H	1.71014200	5.60989700	3.37912000
H	1.84641900	3.99195300	2.66619300
C	3.61543500	8.72811600	-0.00446500
H	2.92722500	9.58475400	-0.01893000

H	4.26157800	8.81712800	-0.88489400
H	4.24161100	8.83048800	0.88875100
C	-1.38780100	-4.97714400	-2.54643000
H	-1.84801600	-3.98909100	-2.66858600
H	-1.70854400	-5.60608000	-3.38298700
H	-0.30387800	-4.83033800	-2.62744300
C	-1.38780100	-4.98065800	2.54340800
H	-1.84641900	-3.99195300	2.66619300
H	-0.30367300	-4.83575800	2.62532400
H	-1.71014200	-5.60989700	3.37912000
C	-3.61543500	-8.72811600	-0.00446500
H	-4.24161100	-8.83048800	0.88875100
H	-2.92722500	-9.58475400	-0.01893000
H	-4.26157800	-8.81712800	-0.88489400
H	-3.50472100	1.55650400	0.00240200
H	3.50472100	-1.55650400	0.00240200
C	-3.89599500	6.01064300	0.00102600
C	-6.12330000	7.67925400	0.00196100
C	-5.19296300	5.45839100	0.00213800
C	-3.71146000	7.39605500	0.00036700
C	-4.83484800	8.22576200	0.00084000
C	-6.30776600	6.29117200	0.00261100
H	-2.71135400	7.82410700	-0.00050100
H	-4.70797200	9.30595900	0.00033800
H	-7.31421800	5.87659600	0.00346100
H	-6.98843800	8.33837000	0.00232500
C	3.89599500	-6.01064300	0.00102600
C	6.12330000	-7.67925400	0.00196100
C	5.19296300	-5.45839100	0.00213800
C	3.71146000	-7.39605500	0.00036700
C	4.83484800	-8.22576200	0.00084000
C	6.30776600	-6.29117200	0.00261100
H	2.71135400	-7.82410700	-0.00050100
H	4.70797200	-9.30595900	0.00033800
H	7.31421800	-5.87659600	0.00346100
H	6.98843800	-8.33837000	0.00232500
C	-5.14013400	3.92943400	0.00260900
C	-5.80512600	3.34436900	1.26869200
H	-5.34550500	3.74679700	2.17751000
H	-5.70643000	2.25236200	1.29022100
H	-6.87483700	3.58494500	1.29100000
C	-5.80688900	3.34357600	-1.26219400
H	-6.87666400	3.58400400	-1.28311600
H	-5.70807800	2.25157100	-1.28324400

H	-5.34862600	3.74554600	-2.17190100
C	5.14013400	-3.92943400	0.00260900
C	5.80688900	-3.34357600	-1.26219400
H	5.70807800	-2.25157100	-1.28324400
H	5.34862600	-3.74554600	-2.17190100
H	6.87666400	-3.58400400	-1.28311600
C	5.80512600	-3.34436900	1.26869200
H	5.70643000	-2.25236200	1.29022100
H	6.87483700	-3.58494500	1.29100000
H	5.34550500	-3.74679700	2.17751000

BO4e_S_0

C	5.58046300	1.24109900	0.01997400
C	5.13988700	-0.07589900	0.00699500
C	3.76125600	-0.36455000	-0.00485300
C	2.81958600	0.70366200	0.00197000
C	3.28230800	2.03834600	0.01558500
C	4.64019900	2.29794300	0.02334700
C	1.38888900	0.37434100	-0.00073400
C	0.97535000	-0.97945000	-0.00342300
C	-0.36681100	-1.33562200	-0.00236500
H	-0.58989900	-2.39605100	-0.00499100
C	-1.38884600	-0.37431100	0.00074600
C	-0.97530100	0.97948100	0.00342200
C	0.36685200	1.33565700	0.00236500
H	5.84828800	-0.90098400	0.00721100
H	2.57820800	2.86487600	0.02091900
H	0.58994400	2.39608300	0.00497000
C	-2.81954600	-0.70362900	-0.00194600
C	-3.28226700	-2.03831700	-0.01541100
C	-4.64015700	-2.29791000	-0.02315800
C	-5.58042000	-1.24106500	-0.01990000
C	-5.13984300	0.07593500	-0.00706100
C	-3.76121200	0.36458300	0.00475200
H	-2.57816400	-2.86484400	-0.02062900
B	3.24081700	-1.82012700	-0.01609100
O	1.87541300	-2.01232500	-0.00883200
B	-3.24076400	1.82016000	0.01592200
O	-1.87537300	2.01236500	0.00881200
C	4.13774700	-3.11626100	-0.01811700
C	4.40402800	-3.79766000	1.19022900
C	4.69243000	-3.61523300	-1.21686700
C	5.20937900	-4.94041700	1.18339100
C	5.49253200	-4.76251900	-1.18958500

C	5.76195200	-5.44238700	0.00125200
H	5.41178000	-5.45090400	2.12373200
H	5.91487800	-5.13501500	-2.12158000
C	-4.13775100	3.11626100	0.01785400
C	-4.40476100	3.79714400	-1.19062100
C	-4.69177900	3.61568400	1.21671100
C	-5.21015700	4.93986700	-1.18378900
C	-5.49195900	4.76291800	1.18942100
C	-5.76208800	5.44228700	-0.00153600
H	-5.41309200	5.44997800	-2.12422000
H	-5.91378500	5.13578000	2.12150500
C	6.92916200	1.81728500	0.03300800
C	8.18426600	1.20425000	0.03551200
C	6.81506500	3.22189400	0.04371300
C	9.32592200	2.00780200	0.04865300
H	8.27466600	0.12105100	0.02749300
C	7.95634400	4.01623100	0.05666900
C	9.21452800	3.40251400	0.05911000
H	10.30966400	1.54602500	0.05082300
H	7.88150400	5.10124700	0.06485300
H	10.11233400	4.01477800	0.06923300
C	-6.92911900	-1.81724500	-0.03286800
C	-8.18421400	-1.20420900	-0.03544000
C	-6.81502500	-3.22186300	-0.04341800
C	-9.32588100	-2.00776100	-0.04849100
C	-7.95630200	-4.01619100	-0.05628300
C	-9.21449200	-3.40246700	-0.05879000
H	-10.30961500	-1.54596600	-0.05071000
H	-7.88148200	-5.10120800	-0.06434300
H	-10.11229000	-4.01474400	-0.06884300
C	6.60417200	-6.69719200	0.00807800
H	5.98138700	-7.59630000	-0.09476100
H	7.32104300	-6.70374600	-0.82012300
H	7.16567000	-6.79923000	0.94329000
C	3.82311200	-3.29936900	2.49797700
H	4.10559800	-2.25725300	2.69587800
H	2.72676800	-3.33711600	2.48908400
H	4.17003700	-3.90206100	3.34342800
C	4.41410900	-2.93110600	-2.53999000
H	3.34921400	-2.98423600	-2.80132200
H	4.68350500	-1.86821600	-2.51374200
H	4.97654800	-3.39739800	-3.35529200
C	-4.41273200	2.93202100	2.53992100
H	-4.68279200	1.86927600	2.51438600

H	-3.34758600	2.98462900	2.80032200
H	-4.97421400	3.39901200	3.35548200
C	-3.82459500	3.29830700	-2.49849200
H	-2.72822300	3.33530100	-2.48995600
H	-4.10786600	2.25635900	-2.69617100
H	-4.17137500	3.90112900	-3.34391000
C	-6.60436500	6.69705300	-0.00841700
H	-5.98144700	7.59630100	0.09237300
H	-7.16738600	6.79801700	-0.94283400
H	-7.31989200	6.70451200	0.82093400
C	5.34803900	3.65326800	0.03864700
C	5.00714100	4.47919200	-1.22227200
H	3.94041000	4.73024400	-1.24575100
H	5.57362800	5.41747200	-1.23216000
H	5.24877600	3.92487200	-2.13469800
C	4.99238800	4.45854500	1.30879200
H	3.92567600	4.71038100	1.32347100
H	5.22240400	3.88888300	2.21473200
H	5.55959200	5.39584400	1.34097800
H	-8.27461800	-0.12100900	-0.02754100
H	-5.84824200	0.90102300	-0.00734600
C	-5.34800100	-3.65323700	-0.03832200
C	-5.00708700	-4.47906200	1.22265100
H	-5.57357500	-5.41734000	1.23262200
H	-3.94035700	-4.73011700	1.24613600
H	-5.24870700	-3.92467000	2.13503700
C	-4.99237100	-4.45860400	-1.30842000
H	-3.92565700	-4.71043000	-1.32310400
H	-5.55956900	-5.39591000	-1.34052700
H	-5.22241300	-3.88900600	-2.21439200

CC5_S_0

C	-0.20047600	5.71816300	-0.00002300
C	-1.35566100	4.95873500	-0.00017000
C	-1.29891500	3.54714200	-0.00010900
C	-0.03520600	2.89253900	0.00005000
C	1.12822700	3.69376000	0.00027800
C	1.05042600	5.07648700	0.00023300
C	-0.00391000	1.43227000	-0.00002300
C	-1.23523100	0.69564500	0.00004500
C	-1.19111900	-0.70539900	0.00001900
H	-2.13918100	-1.22941500	0.00017900
C	0.00391700	-1.43228500	-0.00010500
C	1.23523800	-0.69566000	-0.00009600

C	1.19112600	0.70538300	-0.00012600
H	-0.25777500	6.80323700	-0.00008200
H	-2.33048000	5.44108900	-0.00031100
H	2.10803500	3.22835300	0.00056600
H	1.96313800	5.66619500	0.00042100
H	2.13918900	1.22939900	-0.00024100
C	0.03521500	-2.89255500	-0.00022900
C	1.29892400	-3.54715600	0.00005200
H	-2.10802600	-3.22837200	-0.00111500
C	-1.12821700	-3.69377800	-0.00067200
C	1.35567200	-4.95874900	0.00004500
C	0.20048900	-5.71817900	-0.00028600
C	-1.05041400	-5.07650500	-0.00068700
H	2.33049200	-5.44110100	0.00028600
H	0.25778900	-6.80325300	-0.00027500
H	-1.96312500	-5.66621400	-0.00104700
C	-3.80408000	0.65308200	0.00013000
C	-4.42012900	0.30568000	-1.22074700
C	-4.41954200	0.30481500	1.22104500
C	-5.64124900	-0.37650200	-1.19751400
C	-5.64068700	-0.37736300	1.19787800
C	-6.27183700	-0.72378200	0.00021400
H	-6.11048200	-0.64409300	-2.14257700
H	-6.10952100	-0.64558800	2.14295500
C	3.80408500	-0.65309200	0.00004900
C	4.41987000	-0.30534700	-1.22088100
C	4.41979700	-0.30514300	1.22091100
C	5.64098200	0.37683800	-1.19770700
C	5.64092700	0.37707100	1.19768600
C	6.27181900	0.72381300	-0.00000800
H	6.11005500	0.64465400	-2.14278500
H	6.10991000	0.64509200	2.14274600
C	3.78081900	-0.65570800	2.54659400
H	2.76814700	-0.24374900	2.62805900
H	3.68964000	-1.74036900	2.67717500
H	4.37244300	-0.26456100	3.38005700
C	3.78087500	-0.65599900	-2.54652400
H	3.68805300	-1.74060800	-2.67628800
H	2.76885300	-0.24254200	-2.62867800
H	4.37337700	-0.26636200	-3.38006700
C	7.60663900	1.43246300	-0.00012000
H	7.72634000	2.06280800	0.88767000
H	8.43872900	0.71538900	-0.00317200
H	7.72382000	2.06731000	-0.88505300

C	-7.60667000	-1.43240800	0.00023800
H	-8.43874800	-0.71531600	-0.00177900
H	-7.72433800	-2.06653100	-0.88514800
H	-7.72590700	-2.06347500	0.88757800
C	-3.78024400	0.65490200	2.54669200
H	-2.76818500	0.24145600	2.62841800
H	-3.68744600	1.73945900	2.67691500
H	-4.37252900	0.26486800	3.38020300
C	-3.78145800	0.65681500	-2.54642600
H	-3.69026400	1.74153000	-2.67654800
H	-2.76882000	0.24485200	-2.62831800
H	-4.37329600	0.26606200	-3.37992100
C	-2.50589300	2.76701700	-0.00010600
C	-2.50515600	1.40342400	0.00009800
C	2.50516300	-1.40343700	0.00006300
C	2.50590200	-2.76703000	0.00023100
H	-3.45449800	3.29974200	-0.00023600
H	3.45450700	-3.29975300	0.00046200

BO5_S_0

C	0.85072600	-5.66203700	0.12341400
C	1.91341800	-4.76832900	0.10688900
C	1.66602400	-3.39340100	0.08025000
C	0.35376600	-2.87380700	0.06828900
C	-0.70099600	-3.80992500	0.08641000
C	-0.46324000	-5.17739800	0.11346600
C	0.14987100	-1.41796100	0.03582300
C	1.29085900	-0.56563900	0.02683700
C	1.11944600	0.82356700	-0.00986400
H	2.01512400	1.43691100	-0.01790900
C	-0.14986100	1.41795100	-0.03606600
C	-1.29085700	0.56562600	-0.02698800
C	-1.11944300	-0.82357400	0.00973400
H	1.04188500	-6.73116000	0.14452200
H	2.94555100	-5.10408700	0.11495200
H	-1.72719800	-3.45836100	0.07971600
H	-1.30065800	-5.86901100	0.12707100
H	-2.01511500	-1.43692400	0.01790300
C	-0.35375800	2.87379300	-0.06872700
C	-1.66601000	3.39339600	-0.08054300
H	1.72721200	3.45832800	-0.08074700
C	0.70101000	3.80990000	-0.08722100
C	-1.91340400	4.76832200	-0.10733600
C	-0.85070700	5.66201800	-0.12418800

C	0.46325700	5.17736900	-0.11443300
H	-2.94553600	5.10408100	-0.11526800
H	-1.04185500	6.73114000	-0.14541700
H	1.30067700	5.86897500	-0.12832400
O	2.77088400	-2.58141500	0.06807700
B	2.70196800	-1.20639000	0.04927400
B	-2.70196600	1.20639300	-0.04929800
O	-2.77087400	2.58141600	-0.06811600
C	4.06853700	-0.42317500	0.03783000
C	4.76354500	-0.22090600	-1.17553500
C	4.61995800	0.08846300	1.23230400
C	5.97229900	0.48080500	-1.17662900
C	5.83330600	0.78450000	1.19680800
C	6.52731700	0.99009500	0.00173700
H	6.49390300	0.63460100	-2.12006700
H	6.24691000	1.17383100	2.12566800
C	-4.06854100	0.42319200	-0.03757700
C	-4.62016100	-0.08859300	-1.23195200
C	-4.76332400	0.22100700	1.17587700
C	-5.83338600	-0.78474100	-1.19619000
C	-5.97206200	-0.48081900	1.17723500
C	-6.52721900	-0.99025800	-0.00094800
H	-6.24704100	-1.17431000	-2.12493500
H	-6.49346300	-0.63462300	2.12077800
C	-4.20835800	0.76075000	2.47829400
H	-3.19617100	0.38391400	2.67481100
H	-4.14492700	1.85583900	2.46277700
H	-4.83723100	0.47613300	3.32786100
C	-3.91995000	0.12478700	-2.55876300
H	-3.86578400	1.19062900	-2.81570200
H	-2.88996000	-0.25248200	-2.54123000
H	-4.44601700	-0.38348900	-3.37314600
C	-7.85120700	-1.71862400	0.01733500
H	-7.96442400	-2.32388000	0.92375300
H	-8.69372100	-1.01424100	-0.00708700
H	-7.95664200	-2.38139500	-0.84811200
C	7.85128800	1.71850100	-0.01651100
H	8.69382900	1.01388900	-0.00228700
H	7.96030000	2.33156800	-0.91822300
H	7.96082700	2.37380600	0.85406900
C	3.91932400	-0.12466000	2.55894200
H	2.89043500	0.25567000	2.54200500
H	3.86204800	-1.19065000	2.81453800
H	4.44695200	0.38106300	3.37390100

C	4.20865900	-0.76039900	-2.47808700
H	4.14422800	-1.85542200	-2.46242800
H	3.19689000	-0.38266800	-2.67506900
H	4.83813200	-0.47647300	-3.32744100

CC6_S0

C	-0.84024100	1.22976000	0.00015200
C	-2.27554600	1.23348500	0.00017900
C	-2.99474200	2.50582300	0.00037700
C	-2.24173500	3.71357200	0.00055200
C	-0.16909000	0.00000300	-0.00001200
C	-2.93756400	0.00005900	-0.00000100
C	-2.27559800	-1.23339600	-0.00018600
C	-0.84029400	-1.22973000	-0.00017300
C	-2.24188800	-3.71348400	-0.00055900
C	-2.99484700	-2.50570600	-0.00037400
H	0.91554000	-0.00002100	-0.00001900
H	-4.01932000	0.00008200	0.00000200
C	-2.91165700	4.95746500	0.00077600
C	-4.40407000	2.60533800	0.00041500
C	-5.04184900	3.83463300	0.00062700
C	-4.29234600	5.02381200	0.00081500
H	-2.31762600	5.86851500	0.00091500
H	-5.01309700	1.70779000	0.00027000
H	-6.12767700	3.87700900	0.00064900
H	-4.79548100	5.98684700	0.00099300
C	-2.91186000	-4.95735100	-0.00078700
C	-4.29255100	-5.02364400	-0.00081100
C	-5.04200800	-3.83443500	-0.00060400
C	-4.40418000	-2.60516600	-0.00039200
H	-2.31786400	-5.86842500	-0.00093600
H	-4.79572500	-5.98665800	-0.00098900
H	-6.12783700	-3.87676900	-0.00061200
H	-5.01317100	-1.70759400	-0.00023100
C	1.38984900	-2.51233100	-0.00009500
C	2.09654700	-2.53679400	-1.22072500
C	2.09607500	-2.53810500	1.22075000
C	3.49376400	-2.59408100	-1.19732900
C	3.49332600	-2.59535900	1.19781200
C	4.21231200	-2.62857800	0.00037100
H	4.03427800	-2.61000700	-2.14208800
H	4.03349000	-2.61230500	2.14275000
C	1.38995900	2.51228200	0.00008000
C	2.09664900	2.53665000	1.22072000

C	2.09619500	2.53809400	-1.22075500
C	3.49386400	2.59389800	1.19734000
C	3.49345100	2.59529800	-1.19780000
C	4.21242600	2.62844900	-0.00035500
H	4.03437000	2.60975200	2.14210600
H	4.03362600	2.61226100	-2.14273200
C	1.36751300	-2.50485900	2.54607300
H	0.72151600	-3.38116400	2.67537900
H	2.07596000	-2.48270900	3.37992300
H	0.72085100	-1.62335000	2.62857300
C	1.36845000	-2.50204700	-2.54626000
H	2.07718100	-2.47994500	-3.37987000
H	0.72171800	-3.37768800	-2.67633900
H	0.72260200	-1.61990000	-2.62837200
C	5.72058100	-2.72344500	0.00055700
H	6.15200900	-2.24902900	0.88859000
H	6.05484100	-3.76987700	-0.00298600
H	6.15265100	-2.24290500	-0.88388200
C	1.36853400	2.50181600	2.54624300
H	2.07725300	2.47963600	3.37985900
H	0.72180000	3.37744200	2.67638100
H	0.72268500	1.61965800	2.62827500
C	1.36765200	2.50492000	-2.54609200
H	0.72188900	3.38138200	-2.67549900
H	2.07611500	2.48251200	-3.37992300
H	0.72076700	1.62357100	-2.62853000
C	5.72070000	2.72324200	-0.00051000
H	6.15277200	2.24202900	0.88356500
H	6.15207800	2.24945900	-0.88890200
H	6.05501100	3.76965400	0.00380700
C	-0.10985000	-2.48666900	-0.00031200
C	-0.80626500	-3.65895000	-0.00051200
C	-0.80611400	3.65898000	0.00049600
C	-0.10974400	2.48667400	0.00028300
H	-0.26312700	-4.60160800	-0.00054600
H	-0.26294000	4.60161800	0.00057700

BO6_S_0

C	-0.95280200	1.17594300	0.01210900
C	-2.37531800	1.08456600	0.01111500
C	-3.15566000	2.33358200	0.01743300
C	-2.48309100	3.57552600	0.02316800
C	-0.21041900	-0.01212800	0.00243200
C	-2.98288600	-0.17822400	0.00287500

C	-2.22894700	-1.35935400	-0.00451700
C	-0.80576400	-1.28021100	-0.00520500
C	-2.03769000	-3.84539400	-0.02058900
C	-2.85413400	-2.69291300	-0.01224200
H	0.87538600	0.05274600	0.00062100
H	-4.06348200	-0.24294900	0.00231000
C	-3.18018200	4.78757700	0.02856300
C	-4.56558600	2.37517600	0.01723900
C	-5.26398500	3.57440600	0.02254200
C	-4.56817900	4.78986700	0.02826600
H	-2.60528200	5.70810900	0.03312500
H	-5.12827300	1.44786600	0.01262600
H	-6.35008100	3.56589100	0.02221200
H	-5.10857100	5.73221000	0.03259300
C	-2.58449200	-5.13224400	-0.02835400
C	-3.96219200	-5.30092500	-0.02768700
C	-4.79856100	-4.17756900	-0.01919300
C	-4.24887600	-2.90328700	-0.01164600
H	-1.90328300	-5.97713800	-0.03469800
H	-4.38569600	-6.30125000	-0.03370600
H	-5.87785700	-4.29917800	-0.01843800
H	-4.91854000	-2.05004800	-0.00495600
B	0.02435100	-2.58217600	-0.01448500
O	-0.67111300	-3.77498700	-0.02172000
O	-1.11798100	3.66914600	0.02438400
B	-0.28453300	2.56825400	0.02080900
C	1.59640500	-2.67808700	-0.01551100
C	2.31293600	-2.71065100	-1.23148800
C	2.31306100	-2.73185800	1.19962400
C	3.70869800	-2.79202800	-1.21500900
C	3.70883100	-2.81282000	1.18157700
C	4.42681900	-2.84658300	-0.01714100
H	4.24903700	-2.81182600	-2.16015200
H	4.24928600	-2.84918000	2.12617500
C	1.26465900	2.85137800	0.01847200
C	1.99435400	2.88377500	1.22681600
C	1.94826200	3.07435200	-1.19657800
C	3.37085700	3.12849100	1.20211500
C	3.32548400	3.31557500	-1.18674600
C	4.05631300	3.34897100	0.00417400
H	3.92207300	3.14579900	2.14099700
H	3.84104700	3.47853300	-2.13185800
C	1.58081400	-2.70698300	2.52580200
H	0.91484900	-3.57270600	2.63284900

H	2.28022400	-2.72148700	3.36778100
H	0.95789400	-1.80904800	2.62765300
C	1.58041100	-2.66269700	-2.55686200
H	2.27971800	-2.65961000	-3.39903600
H	0.91671900	-3.52805400	-2.68007200
H	0.95527900	-1.76459900	-2.64152400
C	5.93356600	-2.96336500	-0.01824000
H	6.37212900	-2.50181100	0.87317000
H	6.25280800	-4.01448900	-0.02964000
H	6.37237800	-2.48253500	-0.89928900
C	1.29707700	2.66519300	2.55410200
H	2.01098600	2.66594200	3.38396300
H	0.55775500	3.45177800	2.75334300
H	0.76069900	1.70832200	2.57814800
C	1.19972900	3.05632400	-2.51372800
H	0.45116900	3.85733400	-2.56024600
H	1.88012200	3.18862000	-3.36099200
H	0.66514200	2.10924700	-2.66245900
C	5.53961700	3.63825200	-0.00083800
H	6.04768200	3.14176000	0.83309500
H	6.01032000	3.30356400	-0.93161800
H	5.73725100	4.71474500	0.09398500

BO1a_T_1

C	-0.02920400	4.41066600	-0.16656200
C	-1.24207300	3.73388100	-0.12697300
C	-1.23938500	2.34018300	-0.06803100
C	-0.01377300	1.58691300	-0.04672400
C	1.21708500	2.33204000	-0.09068600
C	1.20140900	3.70929200	-0.14982200
C	-0.03192200	0.18011600	0.00620500
C	-1.26642600	-0.52523100	0.05865700
C	-1.24025400	-1.91931100	0.10847100
H	-2.16506400	-2.48774500	0.13068700
C	0.04517800	-2.62997900	0.14064400
C	1.23542800	-1.95658800	0.09755300
C	1.28464900	-0.52685400	-0.00124700
H	-0.01914800	5.49643700	-0.21051500
H	-2.19420000	4.25493500	-0.13925600
H	2.14857000	4.23869800	-0.18304700
H	0.03781800	-3.71606900	0.20859800
H	2.17303700	-2.50632000	0.14054900
C	4.03222900	-0.25194700	-0.05897800
C	4.88055500	-0.00033500	1.04834600

C	4.53978200	-1.01565900	-1.13832900
C	6.17668500	-0.52186800	1.06903500
C	5.84638900	-1.51466100	-1.08747000
C	6.68184200	-1.28228900	0.00846700
H	6.80964400	-0.33340800	1.93604000
H	6.22406800	-2.09504800	-1.92893300
C	-4.03341700	-0.24220000	0.06864300
C	-4.59836600	-0.77540800	1.24904700
C	-4.81169700	-0.22795400	-1.11232600
C	-5.90367500	-1.28072300	1.22886000
C	-6.10994200	-0.74598700	-1.09869500
C	-6.67739800	-1.27597300	0.06503800
H	-6.32690800	-1.68754800	2.14699600
H	-6.69260400	-0.73841300	-2.01962400
C	-8.09344700	-1.80188200	0.06810800
H	-8.81712800	-0.99151400	0.23318000
H	-8.34897800	-2.27261400	-0.88807900
H	-8.24549400	-2.54054100	0.86274100
C	-4.24680700	0.33591400	-2.39995800
H	-4.02634600	1.40626500	-2.30358000
H	-3.30870100	-0.16100000	-2.68142900
H	-4.94886300	0.21150100	-3.23085100
C	-3.82387800	-0.77629500	2.55116300
H	-2.83386200	-1.23316500	2.43553400
H	-3.66504500	0.24580900	2.92101800
H	-4.35961700	-1.32719400	3.33127000
C	8.09736500	-1.80781600	0.03566700
H	8.21322900	-2.68335400	-0.61254200
H	8.80978600	-1.04703900	-0.31300000
H	8.40055900	-2.09176700	1.04997700
C	3.70887200	-1.26975000	-2.38018300
H	2.79393900	-1.83270300	-2.15919500
H	3.39622400	-0.32915700	-2.85150400
H	4.27763600	-1.83851300	-3.12341300
C	4.39478400	0.81693300	2.22748000
H	4.17399800	1.84897600	1.93105600
H	3.47043100	0.40350300	2.65215000
H	5.14373600	0.84457100	3.02572500
B	-2.56292200	0.31402900	0.04387000
B	2.56192400	0.30466900	-0.06578000
O	-2.42967300	1.70196500	-0.03074000
O	2.42976600	1.70872800	-0.07968400

BO1b_T_1

C	0.02372800	4.55536600	0.08173500
C	1.23778000	3.87945700	0.05689200
C	1.23586900	2.48421300	0.02583300
C	0.01359100	1.73422200	0.01876000
C	-1.21674600	2.47547700	0.04740200
C	-1.20373800	3.85630600	0.07809100
C	0.03287400	0.32204600	-0.00818400
C	1.26402800	-0.38103500	-0.04666900
C	1.23489300	-1.77997500	-0.07122100
H	2.15950300	-2.35074800	-0.07688800
C	-0.05286500	-2.50982700	-0.09434100
C	-1.23632500	-1.80987200	-0.06198500
C	-1.27974900	-0.38492400	0.00881300
H	0.01403600	5.64199900	0.10428800
H	2.18923300	4.40182100	0.05957100
H	-2.15178600	4.38463300	0.09996000
H	-2.17783000	-2.35556300	-0.09511900
C	-4.02871900	-0.10971100	0.06715400
C	-4.87160000	0.09858500	-1.05209500
C	-4.54179000	-0.82769400	1.17391200
C	-6.17034400	-0.41786000	-1.05625600
C	-5.85042900	-1.32336800	1.13938500
C	-6.68199300	-1.13189400	0.03260800
H	-6.79981900	-0.26135900	-1.93216200
H	-6.23246100	-1.86853500	2.00228700
C	4.03057300	-0.09977100	-0.05610700
C	4.59475600	-0.66657000	-1.22126900
C	4.81102100	-0.05094200	1.12247700
C	5.90066200	-1.16977900	-1.18877300
C	6.10995200	-0.56787400	1.12181500
C	6.67633200	-1.13053900	-0.02687300
H	6.32306800	-1.60212200	-2.09561000
H	6.69419800	-0.53286800	2.04114900
C	8.09311800	-1.65450900	-0.01722600
H	8.81532000	-0.84960500	-0.21278600
H	8.35284000	-2.09190000	0.95356400
H	8.24325900	-2.41985100	-0.78663300
C	4.24883000	0.55058500	2.39422000
H	4.03714400	1.61975800	2.26959200
H	3.30599800	0.06936400	2.68673300
H	4.94870200	0.44205600	3.22922500
C	3.81768700	-0.70795500	-2.52125300
H	2.83640500	-1.18045400	-2.39343500
H	3.63856400	0.30307100	-2.91145500

H	4.36187400	-1.26481600	-3.29127400
C	-8.10016300	-1.65149400	0.02323400
H	-8.21752500	-2.51083700	0.69264200
H	-8.80818400	-0.87977100	0.35648900
H	-8.40843600	-1.95853000	-0.98276100
C	-3.71130900	-1.04169500	2.42375300
H	-2.81389500	-1.64051200	2.22575400
H	-3.36942100	-0.08815500	2.84605100
H	-4.29090200	-1.55690200	3.19716000
C	-4.37790100	0.86754800	-2.26020100
H	-4.16711900	1.91315100	-2.00622000
H	-3.44596900	0.44271100	-2.65609800
H	-5.11785400	0.85647600	-3.06728600
B	2.55922800	0.45693200	-0.04537200
B	-2.55698700	0.44633700	0.05800700
O	2.42683400	1.84662700	0.00206200
O	-2.42754700	1.85131100	0.04910900
C	-0.00501000	-4.00221100	-0.16381200
H	0.54357300	-4.32749600	-1.06085900
H	0.55239900	-4.41015900	0.69291900
H	-1.00250600	-4.45054600	-0.18024400

BO1c_T_1

C	0.00006200	4.54062100	0.00002400
C	-1.21171100	3.85163900	0.04527900
C	-1.20485400	2.45364800	0.04151000
C	0.00001100	1.72333500	-0.00005300
C	1.20490900	2.45359900	-0.04153800
C	1.21182100	3.85157100	-0.04524800
C	-0.00004100	0.26981500	-0.00011600
C	-1.23741700	-0.41522000	0.14712100
C	-1.16649300	-1.90430500	0.37020300
C	-0.00001100	-2.56754200	-0.00062900
C	1.16644400	-1.90428700	-0.37104800
C	1.23739700	-0.41527400	-0.14745400
H	0.00009000	5.62758200	0.00003800
H	-2.16590300	4.36864700	0.07315300
H	2.16603400	4.36854400	-0.07307600
C	4.02230900	-0.08025500	0.02927500
C	4.46057000	-0.83553300	1.14346100
C	4.96331200	0.24935200	-0.97563700
C	5.79474500	-1.25222900	1.22625100
C	6.28549200	-0.19108600	-0.86534000
C	6.72474700	-0.94140100	0.23107900

H	6.11524000	-1.83245600	2.09113900
H	6.99247200	0.05468500	-1.65761000
C	-4.02232100	-0.08013500	-0.02916800
C	-4.96306400	0.24915400	0.97608400
C	-4.46086000	-0.83509300	-1.14346600
C	-6.28525800	-0.19132000	0.86602000
C	-5.79502900	-1.25183800	-1.22601100
C	-6.72477200	-0.94134900	-0.23048500
H	-6.99204000	0.05420100	1.65854400
H	-6.11572900	-1.83184000	-2.09097600
C	-8.16631500	-1.37748100	-0.34373600
H	-8.79353100	-0.57208500	-0.75061800
H	-8.27349900	-2.24166500	-1.00827400
H	-8.58211600	-1.64605700	0.63427400
C	-3.51144000	-1.18318200	-2.27139500
H	-3.13279700	-0.28205000	-2.77099800
H	-2.63509300	-1.73968200	-1.91312500
H	-4.00725000	-1.79777100	-3.03016500
C	-4.55842900	1.06241400	2.18868900
H	-3.68516400	0.63065500	2.69516300
H	-4.28674900	2.08670700	1.90792900
H	-5.37307000	1.11514600	2.91833500
C	8.16628100	-1.37750300	0.34455800
H	8.58192800	-1.64705900	-0.63325300
H	8.79360100	-0.57173700	0.75054100
H	8.27351000	-2.24104800	1.00991400
C	4.55896000	1.06289000	-2.18815300
H	3.68574600	0.63130100	-2.69485700
H	4.28732600	2.08715300	-1.90724300
H	5.37373500	1.11568400	-2.91764600
C	3.51085600	-1.18407900	2.27100500
H	3.13165100	-0.28315000	2.77054400
H	2.63487300	-1.74091200	1.91235900
H	4.00663400	-1.79853100	3.02990400
B	-2.53074000	0.41843800	0.06575900
B	2.53075800	0.41834900	-0.06584900
O	-2.39639800	1.81173100	0.04954800
O	2.39645200	1.81163300	-0.04953900
H	-0.00010100	-3.65866100	-0.00094000
C	2.30530700	-2.63043800	-1.01510900
H	2.58071400	-2.15689700	-1.97077200
H	3.22107800	-2.61626700	-0.40931200
H	2.03777200	-3.67296000	-1.21687200
C	-2.30527500	-2.63069200	1.01411100

H	-2.58089800	-2.15726800	1.96975800
H	-3.22100300	-2.61664500	0.40821600
H	-2.03759600	-3.67319000	1.21580400

BO1d_T1

C	-0.00000800	5.22814100	-0.00001400
C	-1.22105700	4.54895800	0.01128500
C	-1.22967800	3.15620500	0.01171100
C	-0.00000400	2.41497400	0.00000900
C	1.22966800	3.15621000	-0.01170500
C	1.22104200	4.54896300	-0.01130200
C	-0.00000100	0.99037100	0.00001500
C	-1.26403200	0.30008900	0.01898700
C	-1.23198400	-1.12077300	-0.01678600
C	0.00000400	-1.83357700	0.00001400
C	1.23198900	-1.12076800	0.01682000
C	1.26403200	0.30009300	-0.01895200
H	-0.00001100	6.31580300	-0.00002300
H	-2.16927200	5.07642900	0.02157600
H	2.16925600	5.07643700	-0.02160100
C	4.01845800	0.57337100	-0.08423000
C	4.86260000	0.75643300	1.03820400
C	4.53245700	-0.11743100	-1.20781400
C	6.16364100	0.24481200	1.02888800
C	5.84238000	-0.61059100	-1.18627000
C	6.67570600	-0.44203600	-0.07675200
H	6.79391700	0.38338200	1.90732700
H	6.22416200	-1.13539500	-2.06194700
C	-4.01846000	0.57335900	0.08424000
C	-4.53249700	-0.11741900	1.20782100
C	-4.86256700	0.75640300	-1.03822300
C	-5.84242100	-0.61057700	1.18624500
C	-6.16361000	0.24478600	-1.02893800
C	-6.67571200	-0.44204100	0.07669800
H	-6.22423000	-1.13536400	2.06192000
H	-6.79385800	0.38334200	-1.90740000
C	-8.09566400	-0.95726000	0.08249900
H	-8.79751600	-0.18550900	0.42866400
H	-8.41746800	-1.25871500	-0.92093200
H	-8.20753100	-1.81946400	0.74941400
C	-4.36834700	1.49621400	-2.26422700
H	-4.17802800	2.55301400	-2.04065400
H	-3.42476400	1.07609500	-2.63658800
H	-5.09944900	1.44963300	-3.07825900

C	-3.69835600	-0.30848600	2.45861100
H	-2.80316200	-0.91275100	2.26648000
H	-3.35196200	0.65262000	2.85958700
H	-4.27590400	-0.80675200	3.24458600
C	8.09565700	-0.95725900	-0.08258800
H	8.20750700	-1.81946100	-0.74950600
H	8.79750300	-0.18550800	-0.42876800
H	8.41748400	-1.25871500	0.92083600
C	3.69826800	-0.30853100	-2.45856600
H	2.80311400	-0.91284300	-2.26639700
H	3.35180700	0.65255900	-2.85952200
H	4.27580500	-0.80676400	-3.24457100
C	4.36841800	1.49626500	2.26421100
H	4.17809100	2.55306000	2.04062400
H	3.42484700	1.07615100	2.63660700
H	5.09954400	1.44970000	3.07822000
B	-2.54366400	1.12275200	0.05907300
B	2.54366100	1.12276100	-0.05902700
O	-2.41956900	2.53474700	0.02890000
O	2.41956100	2.53475500	-0.02888500
H	-2.17029700	-1.66340200	0.00363100
H	2.17030300	-1.66339500	-0.00359300
C	0.00000900	-3.29731800	0.00001600
C	1.09482500	-4.02783600	0.52181700
C	-1.09480100	-4.02784500	-0.52178700
C	1.08893900	-5.41740900	0.52937100
H	1.93361200	-3.49340200	0.95599100
C	-1.08890200	-5.41741900	-0.52934200
H	-1.93359200	-3.49341800	-0.95596200
C	0.00002100	-6.11907200	0.00001500
H	1.93296300	-5.95824500	0.94990000
H	-1.93292100	-5.95826200	-0.94987200
H	0.00002600	-7.20644400	0.00001400

BO1e_T_1

C	-0.01602400	-5.63727200	0.05500100
C	-1.23269200	-4.95678700	0.00886400
C	-1.23367900	-3.56652000	-0.00686600
C	-0.01414200	-2.81138600	0.02334600
C	1.21687300	-3.55381500	0.06887800
C	1.20907800	-4.93312600	0.08534900
C	-0.02903200	-1.40756300	0.01121200
C	-1.26704100	-0.69301900	-0.04094900
C	-1.24817500	0.68841800	-0.03770400

C	0.03475200	1.44269900	-0.01815100
C	1.22931000	0.70835200	-0.01500300
C	1.27238600	-0.68939400	0.04244100
H	-0.00640500	-6.72369600	0.06699200
H	-2.18273200	-5.48178800	-0.01708100
H	2.15855100	-5.45850700	0.12268400
C	4.02489600	-0.95382900	0.14473000
C	4.85419800	-1.08196300	-0.99438500
C	4.54495100	-0.30764800	1.29059600
C	6.15136900	-0.55969600	-0.97870400
C	5.85047400	0.19666800	1.27529600
C	6.67125400	0.08290400	0.14932400
H	6.77223900	-0.65657700	-1.86922400
H	6.23812100	0.68767600	2.16779100
C	-4.03236000	-0.97381800	-0.13683000
C	-4.58144800	-0.45757200	-1.33120300
C	-4.81840000	-0.95596200	1.03822500
C	-5.88122300	0.06248800	-1.33167600
C	-6.11129400	-0.42530900	1.00438700
C	-6.66407100	0.08754800	-0.17422900
H	-6.29220300	0.45742100	-2.26054300
H	-6.70153400	-0.40972700	1.92046200
C	-8.07492900	0.62709000	-0.19869700
H	-8.80599600	-0.18037500	-0.34424600
H	-8.33022700	1.12701400	0.74272000
H	-8.21618000	1.34465800	-1.01437600
C	-4.26657000	-1.50338100	2.33849200
H	-4.05611300	-2.57735300	2.26094300
H	-3.32437400	-1.01152000	2.61548300
H	-4.97117500	-1.35813800	3.16391800
C	-3.79209600	-0.48922300	-2.62380300
H	-2.78909400	-0.06432000	-2.49693300
H	-3.66217600	-1.51832200	-2.98549600
H	-4.29881700	0.07472600	-3.41397000
C	8.08689400	0.60996900	0.16204200
H	8.18458600	1.48292700	0.81732600
H	8.79093400	-0.15043400	0.52813300
H	8.41652500	0.89935300	-0.84212900
C	3.72067100	-0.17742500	2.55609400
H	2.80965000	0.41123000	2.39451500
H	3.40183000	-1.15965400	2.92839300
H	4.29478400	0.30767900	3.35264000
C	4.34805600	-1.77714700	-2.24151000
H	4.14061400	-2.83646000	-2.04780900

H	3.41126100	-1.33043500	-2.60042500
H	5.07857600	-1.71793900	-3.05511400
B	-2.56540000	-1.54168000	-0.09091400
B	2.55493700	-1.51884100	0.11262800
O	-2.43342100	-2.92293900	-0.05774800
O	2.43003900	-2.91777700	0.10272100
H	-2.17499600	1.24402700	-0.13372300
H	2.17217300	1.25005700	0.00768000
C	0.02691800	2.90825800	-0.02052500
C	0.79661300	3.64804800	-0.96898100
C	-0.76831900	3.62543400	0.92788400
C	0.73624700	5.04277300	-0.95712300
C	-0.77415500	5.01771100	0.90311000
C	-0.03563700	5.75344400	-0.03304500
H	1.30097500	5.59446800	-1.70752400
H	-1.36263300	5.55065300	1.64904000
C	-0.05244900	7.26127400	-0.02148300
H	0.63616300	7.65719600	0.73822600
H	0.25531600	7.67385800	-0.98801400
H	-1.04968400	7.64959500	0.21544800
C	-1.53159900	2.92426400	2.03083700
H	-2.45901900	2.46134800	1.67260000
H	-0.93609900	2.12750500	2.48926700
H	-1.80412000	3.63817400	2.81475800
C	1.62964900	2.98615800	-2.04617400
H	2.63040400	2.71552600	-1.68511500
H	1.16681100	2.06812000	-2.42025100
H	1.76790000	3.67048500	-2.88979100

BO1f_T1

C	0.00000000	0.51683800	-0.05156300
C	-0.00001000	3.41648800	-0.10153100
C	-1.23069900	1.28287400	-0.06122300
C	1.23069300	1.28288300	-0.06121000
C	1.23860100	2.64100600	-0.08576900
C	-1.23861500	2.64099800	-0.08576000
H	2.20554400	3.12858400	-0.09217600
H	-2.20556100	3.12856900	-0.09213200
C	0.00000500	-0.88797100	-0.02482900
C	0.00000800	-3.69167200	0.08388600
C	1.25249200	-1.59488500	-0.00401400
C	-1.25247900	-1.59488900	-0.00398600
C	-1.21574000	-2.98888500	0.05429000
C	1.21575300	-2.98888000	0.05428600

H	-2.15471300	-3.53921600	0.07958600
H	2.15472400	-3.53921400	0.07959700
H	0.00001100	-4.77775000	0.13126700
C	-0.00001300	4.82899600	-0.12691000
C	-0.00002200	7.69327800	-0.17711500
C	-1.22399300	5.59861200	-0.14061100
C	1.22396200	5.59861700	-0.14067600
C	1.21331400	6.97689000	-0.16481500
C	-1.21335400	6.97688400	-0.16474000
H	-2.18030400	5.08812300	-0.13216100
H	2.18027700	5.08813500	-0.13224500
H	2.15760800	7.51686000	-0.17453500
H	-2.15765200	7.51685000	-0.17438200
H	-0.00002500	8.77950000	-0.19604000
O	2.43651400	0.62367100	-0.04491800
O	-2.43651500	0.62365500	-0.04496800
B	-2.55576900	-0.75593500	-0.02313500
B	2.55577900	-0.75591500	-0.02315300
C	4.01760100	-1.33845400	0.01329200
C	6.63984800	-2.41750600	0.12517100
C	4.74835600	-1.33011700	1.22355100
C	4.61759600	-1.88824400	-1.14098700
C	5.91172700	-2.41717400	-1.06772200
C	6.03729300	-1.86991100	1.26300300
H	6.36217200	-2.83865300	-1.96613300
H	6.58438700	-1.86577400	2.20555500
C	-4.01758900	-1.33848100	0.01329800
C	-6.63986900	-2.41745200	0.12507500
C	-4.61771100	-1.88778200	-1.14107100
C	-4.74822800	-1.33057700	1.22369600
C	-6.03713300	-1.87030100	1.26309900
C	-5.91192100	-2.41667900	-1.06785400
H	-6.58412600	-1.86647600	2.20572000
H	-6.36250700	-2.83775800	-1.96637500
C	-3.89316000	-1.87837600	-2.47152000
H	-2.87262800	-2.27038200	-2.38457000
H	-3.81095400	-0.85845400	-2.87129900
H	-4.42169300	-2.48123000	-3.21740100
C	-4.14178700	-0.74572100	2.48262500
H	-3.18564900	-1.22585700	2.73113000
H	-4.80792400	-0.87158000	3.34233000
H	-3.94084600	0.32661700	2.36673800
C	-8.04559200	-2.96780900	0.18021100
H	-8.25516700	-3.43764500	1.14796200

H	-8.21575600	-3.71340300	-0.60421800
H	-8.78926500	-2.17103600	0.03979500
C	8.04561900	-2.96776800	0.18000400
H	8.25973400	-3.42616700	1.15218800
H	8.78888600	-2.17305800	0.02629300
H	8.21172300	-3.72266500	-0.59640500
C	3.89284400	-1.87932000	-2.47133200
H	2.87265900	-2.27220900	-2.38428300
H	4.42179200	-2.48164300	-3.21734400
H	3.80969500	-0.85942900	-2.87099100
C	4.14216200	-0.74475800	2.48236900
H	3.18575200	-1.22432800	2.73090700
H	3.94181300	0.32767400	2.36632100
H	4.80819200	-0.87085800	3.34212100

BO1g_T$_1$

C	-0.01195000	2.88514700	0.00362500
C	-1.24277000	2.12011700	0.02892700
C	-1.24019200	0.76106900	0.00989200
C	0.00127700	-0.00942700	0.00205800
C	1.23562100	0.77259900	-0.00465200
C	1.22577400	2.13168000	-0.02213100
C	0.00772800	-1.39487700	0.00089200
C	-1.25293400	-2.11878200	0.01086800
C	-1.20580300	-3.50627500	0.00088400
C	0.02072000	-4.20690100	-0.00168000
C	1.24072800	-3.49491700	-0.00303800
C	1.27503500	-2.10704200	-0.01048500
H	-2.20005700	2.62762200	-0.00739400
H	2.17847500	2.64761100	0.01484900
C	4.03809700	-1.83432000	0.03175400
C	4.76741600	-1.83570900	1.24375600
C	4.64440700	-2.36551000	-1.12764300
C	6.05880800	-2.36839200	1.27958600
C	5.94193200	-2.88798000	-1.05772900
C	6.66706300	-2.89927800	0.13625500
H	6.60375200	-2.37278100	2.22345400
H	6.39698800	-3.29522500	-1.96037000
C	-4.01821500	-1.87198800	-0.03250600
C	-4.62037000	-2.41124800	1.12523200
C	-4.74630000	-1.87844400	-1.24533600
C	-5.91255600	-2.94668300	1.05298800
C	-6.03213300	-2.42406500	-1.28351400
C	-6.63616700	-2.96331400	-0.14176700

H	-6.36444000	-3.36005400	1.95442600
H	-6.57603700	-2.43218800	-2.22796600
C	-8.03779400	-3.52382900	-0.19884300
H	-8.78818400	-2.72219300	-0.15714400
H	-8.21094000	-4.07784800	-1.12890000
H	-8.23418900	-4.19934400	0.64073600
C	-4.13805500	-1.30433500	-2.50823600
H	-3.93466000	-0.23183100	-2.39960900
H	-3.18278300	-1.78868200	-2.75189600
H	-4.80397400	-1.43554000	-3.36734300
C	-3.90532800	-2.38015300	2.46051400
H	-2.86744200	-2.72331700	2.37717600
H	-3.87363300	-1.36056900	2.86868000
H	-4.41100800	-3.01221700	3.19817700
C	8.07472300	-3.44476600	0.19021200
H	8.27212000	-4.12874100	-0.64229900
H	8.81643900	-2.63604000	0.13261400
H	8.26026200	-3.98508200	1.12585200
C	3.92760100	-2.33924900	-2.46208200
H	2.89393700	-2.69489600	-2.37862600
H	3.88337900	-1.31900400	-2.86741800
H	4.43994000	-2.96326700	-3.20199000
C	4.15481700	-1.27010100	2.50839200
H	3.94160100	-0.19923100	2.40243000
H	3.20416800	-1.76367800	2.75160700
H	4.82250200	-1.39719100	3.36674700
B	-2.55848300	-1.28387700	0.00025700
B	2.57279200	-1.26010800	0.00125900
O	-2.44622900	0.09817100	-0.01575800
O	2.44769900	0.12083400	0.02006300
H	-2.13957500	-4.06623200	-0.00301100
H	2.17961500	-4.04627700	-0.00028200
H	0.02576200	-5.29372500	-0.00266500
C	-0.02018200	4.34123500	0.00383300
C	-0.85918200	5.07521100	0.90827500
C	0.81183800	5.08616900	-0.89832200
C	-0.84228100	6.46800100	0.88673600
C	0.77568900	6.47829100	-0.87760800
C	-0.04089700	7.19979900	0.00210000
H	-1.46078100	7.00603400	1.60440600
H	1.39298500	7.02422400	-1.59039500
C	1.67363200	4.42422100	-1.95299400
H	2.65300400	4.11709600	-1.56315000
H	1.19991000	3.52937100	-2.36771000

H	1.86307200	5.12279600	-2.77465700
C	-1.70295100	4.40177400	1.97024200
H	-2.68124800	4.08117800	1.58864300
H	-1.21366600	3.51357700	2.38113000
H	-1.89517700	5.09799900	2.79328100
C	-0.07665300	8.70669800	-0.02309500
H	-0.81177400	9.07371900	-0.75379600
H	-0.35800100	9.11768700	0.95273500
H	0.89520500	9.12757600	-0.30449800

BO2_T_1

C	5.32241100	3.63559600	-0.30827800
C	4.96732900	2.29646000	-0.24369400
C	3.61864800	1.89833600	-0.14199300
C	2.59491000	2.89262700	-0.11699100
C	2.97772300	4.25486000	-0.18495500
C	4.31347000	4.61364400	-0.27568200
C	1.19568200	2.48610600	-0.02690500
C	0.86703300	1.14078600	0.04299100
C	-0.51789700	0.64413600	0.15117900
C	-1.55262900	1.70627200	-0.03255600
C	-1.22773500	3.03109200	-0.08879400
C	0.11669000	3.45542200	-0.04175400
H	6.36629100	3.92982000	-0.38514400
H	5.73525100	1.52600700	-0.27273700
H	2.23054900	5.04109500	-0.16767800
H	4.58073200	5.66712000	-0.32585200
H	-2.03481600	3.75175100	-0.18383100
H	0.33459500	4.51495300	-0.06313300
C	-0.91311000	-0.67424300	0.43165900
C	-2.34169100	-0.99519700	0.39818600
C	-0.01319500	-1.76088400	0.77918700
C	-2.76805600	-2.28444000	0.68193600
C	-0.49062300	-3.02451600	1.05564500
H	1.04883600	-1.57761100	0.83387000
C	-1.86791900	-3.31716100	1.01211800
H	0.21769000	-3.80711600	1.31940500
H	-2.23011200	-4.31739100	1.23333400
H	-3.83493600	-2.49675200	0.64684000
B	3.19608900	0.42968700	-0.06990300
O	1.82943900	0.18695100	0.00329900
O	-2.88143200	1.39011900	-0.13993000
B	-3.36243700	0.10950500	0.04437200
C	4.13232900	-0.83762400	-0.08469200

C	4.25465600	-1.61358000	-1.26086300
C	4.86960500	-1.21766600	1.06001300
C	5.10161800	-2.72605700	-1.27817600
C	5.70492900	-2.33982500	1.00959100
C	5.83568000	-3.10843600	-0.15052100
H	5.19350400	-3.30752300	-2.19536400
H	6.26789400	-2.62050500	1.89948200
C	-4.91921800	-0.08213400	-0.08951300
C	-5.76800300	0.32499300	0.96610800
C	-5.49458400	-0.65847600	-1.24292200
C	-7.14922000	0.14068400	0.85950700
C	-6.88297600	-0.82399600	-1.31922900
C	-7.72870200	-0.43136300	-0.27871000
H	-7.78988600	0.44844000	1.68575800
H	-7.31357300	-1.27106500	-2.21491900
C	6.72219600	-4.33139200	-0.17857800
H	7.53373000	-4.25533800	0.55347400
H	6.15257400	-5.24048400	0.05981600
H	7.16885500	-4.48072700	-1.16813400
C	3.47734700	-1.24735100	-2.50850000
H	2.39619300	-1.34352700	-2.34766900
H	3.66441400	-0.20835200	-2.81060600
H	3.74896900	-1.89209200	-3.35077400
C	4.75092700	-0.44032800	2.35556300
H	4.97819500	0.62336700	2.21633400
H	3.73323900	-0.49680500	2.76479900
H	5.43319300	-0.83201600	3.11721800
C	-4.63144700	-1.06823200	-2.41848900
H	-4.22227300	-0.18948100	-2.93548800
H	-3.77793100	-1.68025100	-2.10352200
H	-5.20513200	-1.64287000	-3.15321100
C	-5.18936200	0.95402300	2.21666200
H	-4.44159100	0.30171800	2.68741200
H	-4.68825100	1.90307700	1.98883300
H	-5.96799900	1.15513700	2.95969300
C	-9.22664900	-0.59520100	-0.38584400
H	-9.70826000	0.34499800	-0.68842500
H	-9.49510100	-1.35361000	-1.12921400
H	-9.66817900	-0.88807900	0.57388700

BO3a_T_1

C	1.20862400	-0.19715700	-0.02437700
C	1.24791700	-1.64896900	-0.03198800
C	2.52643100	-2.30168400	-0.08103000

C	3.73501700	-1.50831200	-0.08080500
C	0.00001100	0.49741000	0.00006400
C	-0.00000300	-2.31837800	-0.00000400
C	-1.24791700	-1.64896100	0.03202300
C	-1.20861500	-0.19714200	0.02446900
C	-3.73501300	-1.50828200	0.08083800
C	-2.52642400	-2.30166400	0.08107500
H	0.00001700	1.58022900	0.00008400
H	-0.00001300	-3.39794100	-0.00004800
C	4.97188900	-2.17263000	-0.16074000
C	2.64468800	-3.72155900	-0.14505700
C	3.87887900	-4.33452000	-0.21114500
C	5.06062400	-3.55881600	-0.22142700
H	5.88025900	-1.57542600	-0.18221900
H	1.75457200	-4.34117400	-0.15105900
H	3.94188700	-5.41805700	-0.26171600
H	6.03013600	-4.04529100	-0.28172800
C	-4.97188100	-2.17261000	0.16079200
C	-5.06061000	-3.55879400	0.22149800
C	-3.87886200	-4.33450300	0.21123100
C	-2.64467600	-3.72153900	0.14513300
H	-5.88025500	-1.57541300	0.18228200
H	-6.03012100	-4.04526800	0.28181300
H	-3.94186900	-5.41803900	0.26183200
H	-1.75455300	-4.34114500	0.15117300
B	-3.63166300	0.01210300	0.03643200
O	-2.32009100	0.54432000	0.04047600
O	2.32011200	0.54430200	-0.04036100
B	3.63166600	0.01208100	-0.03639700
C	-4.79643200	1.06452800	0.02361200
C	-5.64082700	1.22005100	-1.10106600
C	-5.02003100	1.88582700	1.15565300
C	-6.67410800	2.16301900	-1.07192700
C	-6.06687500	2.81082100	1.15293700
C	-6.90644300	2.96819900	0.04582100
H	-7.31128100	2.27528800	-1.94762000
H	-6.23318900	3.42421300	2.03712200
C	4.79644500	1.06450100	-0.02362600
C	5.02013400	1.88561700	-1.15577800
C	5.64074100	1.22020700	1.10110000
C	6.06696100	2.81063000	-1.15311500
C	6.67401200	2.16318400	1.07190200
C	6.90642700	2.96819700	-0.04595000
H	6.23334600	3.42388000	-2.03738500

H	7.31111900	2.27558500	1.94762600
C	-5.43090100	0.40434100	-2.36196200
H	-4.40687800	0.50626600	-2.74214800
H	-5.60143700	-0.66561900	-2.19595300
H	-6.11102800	0.72891400	-3.15614100
C	-4.14144700	1.76741500	2.38431500
H	-4.08993500	0.73321400	2.74804100
H	-3.11300300	2.08328600	2.17267600
H	-4.51960400	2.38842100	3.20269100
C	-8.01139500	3.99856000	0.04929600
H	-7.63226300	4.99022300	-0.23291100
H	-8.80390000	3.73959900	-0.66083100
H	-8.46457000	4.09813900	1.04198400
C	8.01137300	3.99856500	-0.04949700
H	7.63218300	4.99029900	0.23238600
H	8.80375500	3.73979300	0.66083600
H	8.46472200	4.09789100	-1.04213100
C	5.43064100	0.40475000	2.36212800
H	4.40689700	0.50772100	2.74280000
H	5.60002500	-0.66538200	2.19608500
H	6.11145700	0.72869600	3.15597100
C	4.14165900	1.76698600	-2.38449700
H	4.09021000	0.73272500	-2.74806500
H	3.11318700	2.08285900	-2.17299500
H	4.51986400	2.38787400	-3.20293900

BO3b_T_1

C	1.21023100	-0.04738200	0.01843400
C	1.24721000	1.40435900	0.02674900
C	2.52543300	2.05807500	0.07120600
C	3.73520100	1.26836200	0.06477100
C	-0.00000100	-0.74060400	0.00000400
C	-0.00000600	2.07243200	0.00000500
C	-1.24721600	1.40436000	-0.02673700
C	-1.21023500	-0.04737600	-0.01842400
C	-3.73520700	1.26836700	-0.06477100
C	-2.52544500	2.05807800	-0.07119700
H	-0.00000100	-1.82450900	0.00000200
H	-0.00000700	3.15351900	0.00000200
C	4.97237800	1.93543900	0.14174700
C	2.64906300	3.47703900	0.13754300
C	3.88193700	4.08727600	0.19990600
C	5.08252600	3.32328800	0.20592000
H	5.88027100	1.33395700	0.15991100

H	1.76001100	4.10040300	0.14810500
H	3.94276500	5.17283100	0.25240800
C	-4.97238800	1.93544000	-0.14175200
C	-5.08253900	3.32328700	-0.20592400
C	-3.88195300	4.08727200	-0.19989800
C	-2.64907600	3.47703700	-0.13753100
H	-5.88027800	1.33395400	-0.15992300
H	-3.94278000	5.17282800	-0.25239600
H	-1.76002400	4.10040300	-0.14808300
B	-3.63331400	-0.25163200	-0.01723100
O	-2.32041700	-0.78940300	-0.02831200
O	2.32041400	-0.78941000	0.02831600
B	3.63331100	-0.25163500	0.01722900
C	-4.79969000	-1.30353200	0.00273300
C	-5.65049400	-1.44601100	1.12486900
C	-5.01942000	-2.13671300	-1.12192900
C	-6.68651700	-2.38711000	1.10007000
C	-6.06873700	-3.05981300	-1.11531000
C	-6.91549400	-3.20316200	-0.01111000
H	-7.32854600	-2.48933400	1.97471800
H	-6.23140700	-3.68294400	-1.99455700
C	4.79969000	-1.30353200	-0.00273700
C	5.01942700	-2.13671100	1.12192500
C	5.65049800	-1.44600500	-1.12487300
C	6.06875300	-3.05980200	1.11530800
C	6.68652800	-2.38709600	-1.10007300
C	6.91551000	-3.20314500	0.01110900
H	6.23143200	-3.68292600	1.99455800
H	7.32855900	-2.48931400	-1.97471900
C	-5.44430200	-0.61928000	2.37887100
H	-4.41762200	-0.70981700	2.75578700
H	-5.62323300	0.44837100	2.20363700
H	-6.11995400	-0.94296600	3.17768500
C	-4.13391700	-2.03193700	-2.34638600
H	-4.09026700	-1.00268700	-2.72643200
H	-3.10358900	-2.33199000	-2.11966700
H	-4.49978600	-2.67103800	-3.15675500
C	-8.02215800	-4.23112200	-0.00946100
H	-7.64720200	-5.21780500	0.29661000
H	-8.82328300	-3.95750400	0.68589700
H	-8.46201700	-4.34887000	-1.00650400
C	8.02218500	-4.23109400	0.00946200
H	7.64724500	-5.21777600	-0.29663200
H	8.82331900	-3.95745700	-0.68587900

H	8.46203000	-4.34885300	1.00650900
C	5.44429900	-0.61927800	-2.37887700
H	4.41763800	-0.70988000	-2.75583000
H	5.62315400	0.44838300	-2.20363100
H	6.12000000	-0.94291700	-3.17766900
C	4.13393400	-2.03193200	2.34638900
H	4.09028200	-1.00268000	2.72642900
H	3.10360500	-2.33199100	2.11967800
H	4.49981100	-2.67102700	3.15675900
C	-6.41817400	4.01254500	-0.28648900
H	-7.24336300	3.29373800	-0.28169900
H	-6.56227000	4.70051200	0.55803700
H	-6.49971600	4.61627100	-1.20092800
C	6.41816000	4.01254700	0.28646700
H	7.24334800	3.29373800	0.28175100
H	6.56227800	4.70044900	-0.55811000
H	6.49967900	4.61634600	1.20086000

BO3c_T_1

C	-1.21047700	-1.47854200	-0.02001700
C	-1.24618400	-0.02485500	-0.02467900
C	-2.52653700	0.63485200	-0.06524900
C	-3.73239200	-0.16178200	-0.05484100
C	-0.00005000	-2.17016500	0.00005700
C	-0.00000100	0.64005200	0.00012100
C	1.24615200	-0.02488200	0.02485600
C	1.21040600	-1.47856300	0.02015900
C	3.73236400	-0.16188300	0.05499000
C	2.52654600	0.63479000	0.06538600
H	-0.00006100	-3.25411900	0.00003900
H	0.00000700	1.72085500	0.00018100
C	-4.96438000	0.51694100	-0.12180900
C	-2.63652300	2.04901800	-0.12055500
C	-3.86426300	2.70003900	-0.17442700
C	-5.04444300	1.90012800	-0.17631700
H	-5.88010200	-0.07038800	-0.14851800
H	-1.73860500	2.65789600	-0.09725800
H	-6.01482300	2.38300400	-0.25657200
C	4.96437700	0.51679600	0.12200000
C	5.04449000	1.89997800	0.17649300
C	3.86434500	2.69992800	0.17452400
C	2.63658000	2.04894800	0.12064100
H	5.88007600	-0.07056500	0.14876400
H	6.01488300	2.38282100	0.25677900

H	1.73868800	2.65786200	0.09727300
B	3.63306200	-1.68001000	0.01596600
O	2.31835000	-2.22044500	0.03484800
O	-2.31845000	-2.22039600	-0.03474300
B	-3.63313800	-1.67991600	-0.01586200
C	4.79889900	-2.73060500	-0.00392300
C	5.65598600	-2.86591600	-1.12254400
C	5.01300300	-3.57003000	1.11768700
C	6.69238300	-3.80635700	-1.09696800
C	6.06366100	-4.49125500	1.11186800
C	6.91655200	-4.62766900	0.01138800
H	7.33885500	-3.90390100	-1.96878700
H	6.22275600	-5.11848600	1.98877000
C	-4.79901700	-2.73047100	0.00391600
C	-5.01322800	-3.56962300	-1.11787000
C	-5.65599600	-2.86604600	1.12259000
C	-6.06388500	-4.49085500	-1.11217100
C	-6.69240900	-3.80646400	1.09687800
C	-6.91667800	-4.62752000	-0.01165100
H	-6.22305800	-5.11787700	-1.98920800
H	-7.33881800	-3.90419400	1.96872200
C	5.45661600	-2.03383200	-2.37420400
H	4.42786700	-2.10936200	-2.74844400
H	5.65129900	-0.96906800	-2.19823000
H	6.12548400	-2.36521400	-3.17547600
C	4.12181000	-3.47280700	2.33862500
H	4.07686800	-2.44622400	2.72558600
H	3.09254000	-3.77152500	2.10546700
H	4.48408500	-4.11715600	3.14634300
C	8.02460000	-5.65390900	0.01057500
H	7.65140000	-6.64040900	-0.29814600
H	8.82694100	-5.37790300	-0.68232700
H	8.46199100	-5.77286700	1.00851800
C	-8.02474000	-5.65374600	-0.01098200
H	-7.65152300	-6.64032900	0.29745600
H	-8.82700800	-5.37790100	0.68206900
H	-8.46223200	-5.77244900	-1.00891100
C	-5.45637100	-2.03437700	2.37448300
H	-4.42807300	-2.11170900	2.74962600
H	-5.64906800	-0.96927500	2.19842900
H	-6.12648400	-2.36471200	3.17514600
C	-4.12212800	-3.47211100	-2.33885400
H	-4.07727500	-2.44544700	-2.72561100
H	-3.09282200	-3.77081600	-2.10583100

H	-4.48442200	-4.11631900	-3.14667500
C	3.94921300	4.17787400	0.22645900
C	2.99553900	4.93899800	0.92804700
C	4.99038200	4.86368000	-0.42663400
C	3.07480600	6.32989600	0.96731900
H	2.20325900	4.43199100	1.47343200
C	5.06994000	6.25482300	-0.38623000
H	5.72691900	4.30171200	-0.99571800
C	4.11244500	6.99566800	0.31017800
H	2.33053900	6.89499600	1.52393300
H	5.87877500	6.76222400	-0.90725500
H	4.17607400	8.08058900	0.34368700
C	-3.94908100	4.17798500	-0.22642800
C	-4.99020500	4.86385900	0.42666800
C	-2.99540800	4.93904300	-0.92808900
C	-5.06971400	6.25500300	0.38620100
H	-5.72674100	4.30194500	0.99580400
C	-3.07462700	6.32994200	-0.96742500
H	-2.20316700	4.43198300	-1.47348300
C	-4.11221900	6.99578200	-0.31027700
H	-5.87851300	6.76245600	0.90723000
H	-2.33036300	6.89498900	-1.52409600
H	-4.17581200	8.08070300	-0.34383700

BO3d_T_1

C	-1.19037100	-0.24024600	0.12321500
C	-1.22165900	1.18347100	0.14034600
C	0.00776400	-0.92791400	0.09978900
C	0.02039100	1.84602800	0.09535600
C	1.26022500	1.18450600	0.07663500
C	1.21783600	-0.22420200	0.08104900
H	0.01555800	-2.01183400	0.10277000
H	0.00357000	2.92639800	0.04946300
B	3.63244000	-0.42668500	0.02408200
O	2.35115200	-0.96151300	0.05765300
O	-2.32973800	-0.99115600	0.15801200
B	-3.61447800	-0.45632200	0.06285000
C	4.73123400	-1.56353700	0.00558600
C	5.17716100	-2.10985200	-1.21723000
C	5.23188800	-2.09700100	1.21363800
C	6.11253600	-3.14987300	-1.21509100
C	6.16606000	-3.13662700	1.18093800
C	6.61888200	-3.68019600	-0.02524900
H	6.45349000	-3.55627400	-2.16597400

H	6.54940500	-3.53266100	2.12008400
C	-4.74848000	-1.55244300	0.10522600
C	-4.90639800	-2.45429800	-0.97364300
C	-5.59166500	-1.69006700	1.23269500
C	-5.89934400	-3.43720000	-0.92229000
C	-6.56497900	-2.69404000	1.25608600
C	-6.73801900	-3.57793700	0.18749100
H	-6.01921900	-4.11271100	-1.76783700
H	-7.20487800	-2.78890500	2.13187400
C	4.65511400	-1.57179000	-2.53331800
H	3.57361700	-1.73073800	-2.63056600
H	4.83108600	-0.49233600	-2.62643000
H	5.13906000	-2.06113900	-3.38463300
C	4.76852700	-1.54457500	2.54560000
H	4.95219100	-0.46501900	2.62073800
H	3.69163000	-1.69836100	2.69030400
H	5.28668800	-2.02783700	3.38007400
C	7.60555700	-4.82475400	-0.03961300
H	7.09530100	-5.79225100	0.06181000
H	8.17282300	-4.85397100	-0.97609600
H	8.32054800	-4.74991600	0.78735900
C	-7.77951100	-4.67118600	0.24150500
H	-7.35736500	-5.60336500	0.64098500
H	-8.61990500	-4.39375800	0.88690800
H	-8.17644200	-4.89790200	-0.75417100
C	-5.44661300	-0.77529000	2.43160500
H	-4.44225200	-0.83939200	2.86863800
H	-5.61415300	0.27441200	2.16390900
H	-6.16383600	-1.03753600	3.21606500
C	-4.01644500	-2.36420800	-2.19702600
H	-4.00262100	-1.35108000	-2.61928800
H	-2.97909300	-2.62047800	-1.95273900
H	-4.35487700	-3.04579100	-2.98394800
C	7.47218800	1.90586200	-0.08617600
C	6.29279200	1.18937600	-0.05383600
C	5.02623300	1.83640200	-0.02187500
C	5.03586700	3.26980400	-0.02493900
C	6.26037800	3.98268300	-0.05851900
C	7.46490100	3.31656600	-0.08882100
H	8.41879000	1.37229500	-0.11001700
H	6.33669300	0.10922100	-0.05273700
C	3.79665000	3.96333100	0.00842800
H	6.22780800	5.06990500	-0.05971800
H	8.40044400	3.86856500	-0.11455100

C	2.60748200	3.29246500	0.04114200
H	3.80811600	5.05119500	0.00872300
H	1.68964700	3.86732800	0.07069200
C	-4.96222900	1.80367200	-0.29823400
C	-5.01828800	3.22192300	-0.04271700
C	-3.82730200	3.90011700	0.39860500
C	-2.60376100	3.23553400	0.47138700
H	-6.10848600	0.12814300	-1.02495700
C	-6.13226400	1.18353000	-0.78518400
C	-6.21811700	3.90557400	-0.22439500
H	-3.88538300	4.96228400	0.61847700
H	-1.72310500	3.80724500	0.74889400
C	-7.38564800	3.24653900	-0.68875000
C	-7.33690000	1.89819000	-0.97232600
H	-6.25727300	4.96898600	-0.00049900
H	-8.30424700	3.81130400	-0.82088400
H	-8.21430400	1.37495200	-1.34073100
C	2.56193600	1.86587000	0.04148100
C	3.76397100	1.12014300	0.01250400
C	-2.49273400	1.87760000	0.19696200
C	-3.74153800	1.07595400	-0.04170200

BO3e_T_1

C	-1.20777700	1.62409000	-0.00493600
C	-1.25019500	0.18124200	-0.00238900
C	-2.52672800	-0.47298500	0.00504100
C	-3.73630000	0.32542300	-0.01202100
C	0.00000200	2.31765800	0.00005900
C	0.00000200	-0.48602600	0.00006000
C	1.25019900	0.18123700	0.00251600
C	1.20778000	1.62408400	0.00505800
C	3.73630700	0.32541400	0.01212300
C	2.52673700	-0.47299200	-0.00492800
H	0.00000200	3.40073900	0.00005700
H	0.00000100	-1.56595700	0.00004900
C	-4.98174000	-0.30993300	0.02039400
C	-2.64643200	-1.90203900	0.03995300
C	-3.88004900	-2.49610100	0.06267700
C	-5.07329400	-1.70453600	0.05506600
H	-5.88190300	0.30071300	0.02181200
H	-1.75391800	-2.51956200	0.05326100
C	4.98174900	-0.30994100	-0.02034400
C	5.07330500	-1.70454100	-0.05504000
C	3.88006300	-2.49610400	-0.06262800

C	2.64644400	-1.90204200	-0.03986500
H	5.88191200	0.30070400	-0.02177500
H	1.75393100	-2.51956600	-0.05316200
B	3.62120800	1.85060600	0.03655800
O	2.32164900	2.37930000	0.01304700
O	-2.32164400	2.37930900	-0.01292400
B	-3.62120400	1.85061500	-0.03640400
C	4.79371000	2.89868500	0.05386700
C	5.55276000	3.13642700	1.22249400
C	5.10512200	3.62878500	-1.11700100
C	6.59214900	4.07290200	1.19961900
C	6.15355000	4.55279900	-1.10625300
C	6.90955600	4.79290100	0.04508600
H	7.16565900	4.24762000	2.10852500
H	6.38719500	5.09864600	-2.01904500
C	-4.79371000	2.89868800	-0.05382600
C	-5.10514000	3.62889300	1.11696100
C	-5.55276500	3.13629600	-1.22248500
C	-6.15359200	4.55289200	1.10611100
C	-6.59216700	4.07274900	-1.19971500
C	-6.90959000	4.79286500	-0.04525100
H	-6.38725600	5.09882400	2.01884500
H	-7.16568000	4.24735500	-2.10864200
C	5.24166800	2.41196100	2.51733200
H	4.21354400	2.60632700	2.84864800
H	5.34162300	1.32517000	2.41522300
H	5.91211400	2.73446800	3.32051200
C	4.31575900	3.41399700	-2.39217400
H	4.31495400	2.35883900	-2.69469100
H	3.26770300	3.71201300	-2.26850100
H	4.73267700	3.99594800	-3.22036600
C	8.01715700	5.82078900	0.04742800
H	7.62880000	6.82459700	0.26728100
H	8.77443100	5.59322900	0.80538600
H	8.51708800	5.87491700	-0.92602400
C	-8.01720100	5.82074200	-0.04774100
H	-7.62910000	6.82425000	-0.26941500
H	-8.77532700	5.59216000	-0.80454500
H	-8.51603300	5.87618200	0.92619400
C	-5.24166000	2.41167400	-2.51723100
H	-4.21353600	2.60600900	-2.84856800
H	-5.34160200	1.32489400	-2.41498300
H	-5.91210700	2.73406900	-3.32045500
C	-4.31579200	3.41424600	2.39216700

H	-4.31497300	2.35911800	2.69478900
H	-3.26774000	3.71226900	2.26848000
H	-4.73273300	3.99627200	3.22029500
C	6.21968200	-2.58961600	-0.08904400
C	5.75900500	-3.92897500	-0.11559100
C	7.59532500	-2.31069600	-0.09718500
C	6.66671300	-4.97889300	-0.14866100
C	8.49649800	-3.37186400	-0.13100800
H	7.95322000	-1.28471800	-0.07765800
C	8.03979700	-4.69723900	-0.15632900
H	6.32525200	-6.01127200	-0.16853500
H	9.56461600	-3.17186000	-0.13781100
H	8.75684200	-5.51322100	-0.18223600
C	-6.21966800	-2.58961000	0.08902600
C	-5.75899100	-3.92897000	0.11556500
C	-7.59531200	-2.31069000	0.09713400
C	-6.66669900	-4.97888800	0.14859400
C	-8.49648500	-3.37185700	0.13091600
H	-7.95320700	-1.28471200	0.07761200
C	-8.03978500	-4.69723400	0.15622800
H	-6.32523900	-6.01126700	0.16846100
H	-9.56460300	-3.17185400	0.13769100
H	-8.75683000	-5.51321600	0.18210200
C	4.23059900	-3.98495500	-0.10185100
C	-4.23058400	-3.98495400	0.10186700
C	-3.68250200	-4.66238600	1.37835800
H	-4.00244900	-5.70980000	1.42820300
H	-2.58650500	-4.64433800	1.38617900
H	-4.03868000	-4.15308400	2.27936700
C	-3.70651800	-4.72199400	-1.15150100
H	-2.61100800	-4.69978300	-1.18345900
H	-4.02215300	-5.77192800	-1.14352200
H	-4.08398000	-4.25859200	-2.06848200
C	3.70656800	-4.72201400	1.15152100
H	2.61105900	-4.69980400	1.18350900
H	4.02220400	-5.77194700	1.14351700
H	4.08405500	-4.25862500	2.06849900
C	3.68248100	-4.66236800	-1.37833600
H	4.00242300	-5.70978200	-1.42820400
H	2.58648300	-4.64431700	-1.38612700
H	4.03863500	-4.15305500	-2.27934800

BO4a_T_1

C	3.88557600	4.18028500	-0.02569200

C	4.26403300	2.83132400	-0.00937900
C	3.32003100	1.80379000	0.01128100
C	1.91139000	2.13891100	0.00674200
C	1.54732500	3.52773800	-0.00782000
C	2.51561500	4.51190900	-0.02303300
C	0.95638700	1.09959200	0.01054500
C	1.37549600	-0.29494800	0.01122600
C	0.48364300	-1.32214200	0.01082100
H	0.88512000	-2.32839300	0.01214300
C	-0.95638600	-1.09958900	0.01063400
C	-1.37549500	0.29495100	0.01121800
C	-0.48364200	1.32214600	0.01073000
H	4.63819400	4.96416200	-0.04046200
H	5.32016900	2.56848000	-0.01273300
H	0.50154300	3.81716000	-0.00910500
H	2.21511800	5.55744400	-0.03486800
H	-0.88511900	2.32839700	0.01198000
C	-1.91138900	-2.13890800	0.00692700
C	-1.54732500	-3.52773600	-0.00752100
C	-2.51561500	-4.51190800	-0.02263100
C	-3.88557700	-4.18028400	-0.02529000
C	-4.26403300	-2.83132100	-0.00908700
C	-3.32003000	-1.80378600	0.01145900
H	-0.50154300	-3.81715900	-0.00880000
H	-2.21511900	-5.55744400	-0.03438000
H	-4.63819500	-4.96416300	-0.03997600
H	-5.32016900	-2.56847700	-0.01244300
B	3.72203800	0.31329500	0.02333500
O	2.70549200	-0.62604500	0.01333600
B	-3.72203700	-0.31329100	0.02337800
O	-2.70549100	0.62604800	0.01331300
C	5.19613100	-0.24117900	0.02018700
C	5.96248600	-0.28956300	1.20517000
C	5.77816200	-0.69088600	-1.18715800
C	7.27632100	-0.77183700	1.16549700
C	7.09339700	-1.16410900	-1.19273000
C	7.86108500	-1.21486100	-0.02388300
H	7.85682700	-0.80157800	2.08732400
H	7.53188000	-1.49868200	-2.13277900
C	-5.19613000	0.24118200	0.02025700
C	-5.77820400	0.69083900	-1.18708500
C	-5.96244300	0.28960900	1.20526600
C	-7.09343900	1.16406300	-1.19262800
C	-7.27628000	0.77188100	1.16562100

C	-7.86108500	1.21486100	-0.02375500
H	-7.53195500	1.49860000	-2.13267300
H	-7.85675400	0.80165500	2.08746800
C	-5.36847700	-0.14430800	2.52965800
H	-4.89679300	-1.13202200	2.46098600
H	-4.59312300	0.55748100	2.86589500
H	-6.13185400	-0.19047200	3.31343000
C	-4.98675000	0.65798700	-2.47818800
H	-4.10345400	1.30644100	-2.42058900
H	-4.62631500	-0.35462400	-2.70411100
H	-5.59266900	0.99110500	-3.32714500
C	-9.27135500	1.75600400	-0.04538600
H	-9.27689800	2.85316000	0.01645200
H	-9.79202700	1.48308500	-0.97056200
H	-9.85863700	1.37913100	0.79906900
C	4.98666100	-0.65809000	-2.47823400
H	4.10338400	-1.30656600	-2.42058600
H	4.62619000	0.35450400	-2.70417300
H	5.59255700	-0.99121600	-3.32720300
C	5.36856500	0.14439200	2.52957200
H	4.89686600	1.13209600	2.46088600
H	4.59323400	-0.55739700	2.86586300
H	6.13197300	0.19059000	3.31331200
C	9.27135400	-1.75600200	-0.04554400
H	9.27690000	-2.85315900	0.01630600
H	9.79200200	-1.48309400	-0.97073700
H	9.85865800	-1.37912000	0.79889100

BO4b_T_1

C	-4.19568200	-3.90025400	0.00422600
C	-4.45428400	-2.52068400	0.01085200
C	-3.44119000	-1.56147500	0.02065600
C	-2.05981100	-1.99324000	0.01714500
C	-1.80362400	-3.40614600	0.01194600
C	-2.83872300	-4.31277100	0.00628000
C	-1.03252200	-1.02830400	0.01460300
C	-1.34906400	0.39015300	0.01217800
C	-0.38717000	1.35196300	0.01212300
H	-0.71535400	2.38354000	0.01092800
C	1.03252400	1.02831200	0.01454900
C	1.34906500	-0.39014500	0.01218000
C	0.38717200	-1.35195500	0.01217400
H	-5.48823100	-2.18062600	0.00778600
H	-0.78374000	-3.77451000	0.01165600

H	-2.61196700	-5.37716900	0.00190000
H	0.71535500	-2.38353300	0.01101600
C	2.05981300	1.99324800	0.01703700
C	1.80362500	3.40615300	0.01177000
C	2.83872400	4.31277800	0.00604700
C	4.19568300	3.90026100	0.00400000
C	4.45428600	2.52069200	0.01068600
C	3.44119300	1.56148200	0.02055200
H	0.78374200	3.77451700	0.01146600
H	2.61196800	5.37717500	0.00161000
H	5.48823200	2.18063300	0.00761400
B	-3.73574200	-0.04734900	0.02113300
O	-2.65383900	0.81355800	0.01052900
B	3.73574500	0.04735600	0.02108600
O	2.65384000	-0.81355000	0.01053300
C	-5.16381200	0.61786700	0.01042200
C	-5.90988700	0.76584600	1.20009000
C	-5.71940000	1.08065100	-1.20318400
C	-7.17668000	1.35822600	1.15848300
C	-6.98884400	1.66601300	-1.21071400
C	-7.73411300	1.81818000	-0.03756200
H	-7.74115000	1.46467900	2.08356700
H	-7.40689700	2.01089200	-2.15519400
C	5.16381200	-0.61786400	0.01040400
C	5.71934400	-1.08080800	-1.20318100
C	5.90993900	-0.76569800	1.20004400
C	6.98877500	-1.66616900	-1.21070300
C	7.17674100	-1.35808900	1.15844500
C	7.73411000	-1.81818900	-0.03756000
H	7.40677900	-2.01116900	-2.15516200
H	7.74125400	-1.46442900	2.08351300
C	-9.09028500	2.48448100	-0.05857500
H	-9.00220000	3.57174700	0.07154200
H	-9.60723100	2.31429100	-1.00938900
H	-9.73251100	2.11353400	0.74762600
C	-5.34286400	0.31250700	2.53011400
H	-5.00300100	-0.72943600	2.49431600
H	-4.47693700	0.92060300	2.82315100
H	-6.08708900	0.39477500	3.32889800
C	-4.94962900	0.94577100	-2.50120200
H	-4.02000800	1.52760400	-2.47707000
H	-4.66904300	-0.09702900	-2.69836300
H	-5.54038500	1.29640500	-3.35354900
C	-5.30659600	-4.91647000	-0.00571500

H	-5.25480900	-5.57762400	0.86994200
H	-6.28918100	-4.43485800	-0.00355300
H	-5.25201500	-5.56324200	-0.89190300
C	5.30659700	4.91647700	-0.00595600
H	5.25507200	5.57735600	0.86992700
H	6.28918100	4.43486200	-0.00422300
H	5.25175500	5.56352800	-0.89192100
C	5.34298500	-0.31223200	2.53005500
H	5.00293100	0.72964200	2.49412100
H	4.47720100	-0.92043600	2.82329100
H	6.08731300	-0.39423700	3.32876900
C	4.94950900	-0.94607400	-2.50117700
H	4.01984600	-1.52783300	-2.47690200
H	4.66899000	0.09671700	-2.69848300
H	5.54019200	-1.29688200	-3.35350200
C	9.09028100	-2.48449000	-0.05861800
H	9.00203900	-3.57201400	0.06921700
H	9.60813400	-2.31243100	-1.00861000
H	9.73175300	-2.11516300	0.74891700

BO4c_T_1

C	-1.01647600	-1.06641800	-0.08735100
C	-1.34099900	0.33068500	0.12184600
C	-0.38071100	1.30392700	0.19519400
H	-0.72214800	2.29634900	0.45278700
C	1.01463500	1.04123700	0.01035900
C	1.34273500	-0.34315600	-0.15692800
C	0.38577500	-1.32826700	-0.24123800
H	0.73430100	-2.32592800	-0.46810500
B	-3.72157300	-0.07182200	0.07452900
O	-2.63624000	0.74991600	0.31287100
B	3.70583200	0.07454900	-0.00003500
O	2.64080800	-0.76225100	-0.26098200
C	-5.14981100	0.58430400	0.16224000
C	-6.01896500	0.31387700	1.24269500
C	-5.58512400	1.46344100	-0.85663800
C	-7.28450900	0.90950600	1.28422800
C	-6.85893200	2.03402200	-0.78850400
C	-7.72481800	1.77273700	0.27810900
H	-7.94270000	0.69436200	2.12436300
H	-7.18471300	2.69873400	-1.58717600
C	5.13723900	-0.58463800	0.00999800
C	6.03218200	-0.42463800	-1.07100600
C	5.54604800	-1.35535600	1.12185800

C	7.29643700	-1.02161500	-1.02272400
C	6.81870600	-1.93383600	1.14062800
C	7.70991800	-1.78190000	0.07445400
H	7.97494700	-0.89014900	-1.86413700
H	7.12248200	-2.51673200	2.00878400
C	-9.08578500	2.42544400	0.35015800
H	-9.01818200	3.44441200	0.75479700
H	-9.54720400	2.50323200	-0.64078200
H	-9.76658600	1.86282600	0.99764600
C	-5.58962700	-0.59064600	2.38016200
H	-5.27524600	-1.57691000	2.02019200
H	-4.73980700	-0.16554800	2.92989200
H	-6.40323700	-0.73871900	3.09750900
C	-4.68558000	1.79087300	-2.03146300
H	-3.79102800	2.33676600	-1.70872200
H	-4.33859300	0.88385900	-2.54306400
H	-5.20680900	2.40809700	-2.77015800
C	4.61791700	-1.55798800	2.30257800
H	4.28979400	-0.60150700	2.73000500
H	3.71276900	-2.10427400	2.01090100
H	5.10781200	-2.12487900	3.10072300
C	5.63539600	0.37287000	-2.29713100
H	4.75185700	-0.05609600	-2.78688800
H	5.38816600	1.41127800	-2.04571500
H	6.44380900	0.39214300	-3.03505400
C	9.06901100	-2.44227800	0.09567500
H	9.02606000	-3.45659700	-0.32401800
H	9.79842000	-1.87837400	-0.49590300
H	9.45653500	-2.53134100	1.11648600
C	-1.48780700	-6.30987700	-0.30458500
C	-2.64045200	-5.70903500	-0.81582200
C	-2.84715300	-4.32014800	-0.71413800
C	-1.82453500	-3.49174600	-0.13941800
C	-0.71914500	-4.13166000	0.43329400
C	-0.54206800	-5.52338200	0.34360600
H	-4.86730000	-4.37897500	-1.51380300
H	-1.34770000	-7.38347500	-0.39221000
H	-3.41092600	-6.31752100	-1.28321400
C	-4.09890400	-3.73751400	-1.09084000
C	-2.03672400	-2.03515100	-0.13508500
H	0.00828400	-3.55201400	0.98890000
H	0.33978800	-5.97684200	0.78722400
C	-3.42106100	-1.54212300	-0.27243700
C	-4.38442400	-2.39552800	-0.78357900

H	-5.39004900	-2.01584500	-0.94808000
C	3.39491500	1.56690200	0.23178200
C	2.06029900	2.03388100	0.05332500
C	1.83639800	3.47227200	-0.03378800
C	2.89390600	4.35470800	0.37162400
C	4.17066700	3.82417100	0.71456800
C	4.42104500	2.48007400	0.57418100
H	-0.08940500	3.44376000	-1.02880100
C	0.67075800	4.06835700	-0.57838500
C	2.68190400	5.75634400	0.35769100
H	4.95586600	4.50976300	1.02351800
H	5.42125300	2.09120200	0.74593600
C	1.50204400	6.29615400	-0.10968200
C	0.50346700	5.44171000	-0.61388500
H	3.48739400	6.40349300	0.69649000
H	1.35792700	7.37298100	-0.12357600
H	-0.40039300	5.85992300	-1.04808900

BO4d_T_1

C	-1.18612200	-0.84315600	-0.00004400
C	-1.25707900	0.60805700	0.00480600
C	-0.14434000	1.39556100	0.00271700
H	-0.29710600	2.46743400	0.00667600
C	1.18613100	0.84316100	0.00009900
C	1.25708700	-0.60805500	0.00488100
C	0.14434800	-1.39555600	0.00265700
H	0.29711300	-2.46742900	0.00658700
B	-3.67890600	0.59017100	0.02039700
O	-2.46809700	1.24990100	0.01327700
B	3.67891100	-0.59016500	0.02075500
O	2.46810400	-1.24989700	0.01343400
C	-4.97667700	1.48189700	0.03721700
C	-5.65054800	1.75757400	1.24668000
C	-5.49147700	2.01245500	-1.16649600
C	-6.81140300	2.53837100	1.23391000
C	-6.65482200	2.78700700	-1.14472700
C	-7.33024700	3.06435700	0.04790800
H	-7.32290800	2.74058600	2.17360800
H	-7.04501700	3.18215200	-2.08143400
C	4.97668300	-1.48189300	0.03740600
C	5.49148200	-2.01222400	-1.16640700
C	5.65054700	-1.75781300	1.24682000
C	6.65482500	-2.78678600	-1.14478600
C	6.81139400	-2.53861600	1.23390000

C	7.33024200	-3.06437200	0.04779500
H	7.04502100	-3.18175100	-2.08156900
H	7.32289000	-2.74102300	2.17356100
C	-8.57047500	3.92794100	0.05502000
H	-8.31316700	4.99511900	0.09491300
H	-9.17132700	3.77523600	-0.84840500
H	-9.20308200	3.71402000	0.92310500
C	-5.11819300	1.23203800	2.56440100
H	-4.97204300	0.14532100	2.54004900
H	-4.14640500	1.68002800	2.80962800
H	-5.80174700	1.45817200	3.38902300
C	-4.79045100	1.74863400	-2.48348500
H	-3.78708100	2.19226300	-2.49782600
H	-4.66808700	0.67373600	-2.66908500
H	-5.35119000	2.16795400	-3.32492300
C	5.11818500	-1.23254900	2.56464700
H	4.97208600	-0.14582200	2.54053800
H	4.14636900	-1.68054900	2.80974900
H	5.80170900	-1.45890400	3.38923300
C	4.79046100	-1.74814300	-2.48334600
H	3.78708800	-2.19176400	-2.49777600
H	4.66810300	-0.67320800	-2.66873900
H	5.35119900	-2.16730200	-3.32486400
C	8.57046000	-3.92797100	0.05475200
H	8.31314500	-4.99514400	0.09472900
H	9.17117600	-3.77530800	-0.84877000
H	9.20320200	-3.71402700	0.92273400
C	-5.94870000	-5.28422700	-0.08411500
C	-5.98931200	-3.89365100	-0.06264400
C	-4.80555800	-3.13671700	-0.04393000
C	-3.53565300	-3.81019800	-0.04723800
C	-3.52562700	-5.23258900	-0.06942600
C	-4.70812600	-5.95058200	-0.08734100
H	-5.76830200	-1.19263400	-0.01817800
H	-6.87208700	-5.85594200	-0.09841100
H	-6.94483200	-3.37433400	-0.06002400
C	-4.80776700	-1.70402000	-0.02064600
C	-2.34805400	-3.05120000	-0.03022700
H	-2.56927900	-5.74991700	-0.07216700
H	-4.68027700	-7.03680900	-0.10414300
C	-2.36473900	-1.62729400	-0.00913900
C	-3.65188800	-0.95749400	-0.00133900
C	2.36474200	1.62729500	-0.00888900
C	2.34805300	3.05120900	-0.02996600

C	3.53565000	3.81020900	-0.04691300
C	4.80555700	3.13672900	-0.04355100
C	4.80776700	1.70402900	-0.02027700
C	3.65189200	0.95749900	-0.00101800
H	2.56927500	5.74992500	-0.07187700
C	3.52562400	5.23259900	-0.06909400
C	5.98930700	3.89366300	-0.06220800
H	5.76830300	1.19264600	-0.01776100
C	5.94869600	5.28424300	-0.08367400
C	4.70812400	5.95059400	-0.08695200
H	6.94482900	3.37434800	-0.05954800
H	6.87208300	5.85595700	-0.09792600
H	4.68027300	7.03682100	-0.10375000
H	1.40554000	3.58829400	-0.03631600
H	-1.40554300	-3.58828900	-0.03652700

BO4e_T_1

C	5.55465100	-1.27259200	-0.01606500
C	5.11812200	0.06295100	-0.00535400
C	3.76059100	0.36619200	0.00612300
C	2.78001100	-0.70577900	0.00137200
C	3.25146800	-2.06762400	-0.00806400
C	4.59535100	-2.33057400	-0.01623000
C	1.40777000	-0.37761000	0.00189100
C	0.97391600	1.00469600	0.00379700
C	-0.34272800	1.35800700	0.00195800
H	-0.56894900	2.41669300	0.00352700
C	-1.40775300	0.37759900	-0.00176000
C	-0.97390000	-1.00470800	-0.00365500
C	0.34274400	-1.35801800	-0.00181600
H	5.83937700	0.87716600	-0.00633800
H	2.54040900	-2.88724500	-0.01011300
H	0.56896400	-2.41670400	-0.00337800
C	-2.77999600	0.70577000	-0.00127500
C	-3.25145400	2.06761400	0.00811400
C	-4.59533900	2.33056300	0.01621100
C	-5.55463900	1.27257900	0.01601800
C	-5.11810700	-0.06296400	0.00536100
C	-3.76057400	-0.36620200	-0.00603400
H	-2.54039700	2.88723700	0.01017500
B	3.25302700	1.82477100	0.01487800
O	1.88809300	2.02842300	0.00813500
B	-3.25301000	-1.82478000	-0.01473100
O	-1.88807700	-2.02843700	-0.00798000

C	4.16184200	3.11217300	0.01617800
C	4.46311700	3.77101800	-1.19637000
C	4.69115900	3.62644300	1.21982500
C	5.27843100	4.90666900	-1.18873100
C	5.50269700	4.76565000	1.19313000
C	5.80704900	5.42317100	-0.00184800
H	5.50786000	5.39979400	-2.13213800
H	5.90609000	5.14971100	2.12883900
C	-4.16184000	-3.11217400	-0.01605200
C	-4.46349900	-3.77081400	1.19651600
C	-4.69079800	-3.62662600	-1.21977100
C	-5.27881800	-4.90645500	1.18881300
C	-5.50236100	-4.76582300	-1.19314000
C	-5.80708400	-5.42314300	0.00184900
H	-5.50853100	-5.39942900	2.13223100
H	-5.90547200	-5.15003200	-2.12890800
C	6.88434700	-1.84699800	-0.02772900
C	8.14791000	-1.23669900	-0.03224900
C	6.76983900	-3.25864200	-0.03476500
C	9.28410400	-2.04308400	-0.04360800
H	8.24107900	-0.15392700	-0.02709500
C	7.90850700	-4.05216200	-0.04598700
C	9.16969600	-3.43977600	-0.05040800
H	10.26958200	-1.58503100	-0.04728800
H	7.83275600	-5.13706600	-0.05135200
H	10.06632100	-4.05350600	-0.05922300
C	-6.88433700	1.84698400	0.02759900
C	-8.14790000	1.23668500	0.03206500
C	-6.76983000	3.25862900	0.03461200
C	-9.28409600	2.04306800	0.04334900
C	-7.90850000	4.05214900	0.04576000
C	-9.16968900	3.43976100	0.05012800
H	-10.26957300	1.58501500	0.04698600
H	-7.83275000	5.13705200	0.05110800
H	-10.06631500	4.05349100	0.05888400
C	6.66075100	6.67021000	-0.00886100
H	6.04381000	7.57641000	0.06196700
H	7.35606700	6.68524000	0.83742600
H	7.24709600	6.75075000	-0.93084900
C	3.90801000	3.25583500	-2.50865500
H	4.18681900	2.20851500	-2.68255100
H	2.81196900	3.30167400	-2.52457300
H	4.27790300	3.84210600	-3.35592100
C	4.37253400	2.96718100	2.54636100

H	3.30444100	3.04922900	2.78631100
H	4.61636900	1.89789000	2.53803400
H	4.93075000	3.43068900	3.36616100
C	-4.37180400	-2.96753600	-2.54630400
H	-4.61599300	-1.89832000	-2.53829500
H	-3.30358200	-3.04928300	-2.78577300
H	-4.92952000	-3.43138300	-3.36625200
C	-3.90878600	-3.25540800	2.50888000
H	-2.81273900	-3.30101800	2.52504400
H	-4.18785200	-2.20813000	2.68262400
H	-4.27874300	-3.84168400	3.35611500
C	-6.66080200	-6.67017200	0.00882900
H	-6.04377700	-7.57643300	-0.06046100
H	-7.24830200	-6.74994900	0.93015200
H	-7.35505900	-6.68589300	-0.83831100
C	5.30226100	-3.68801800	-0.02814800
C	4.96193200	-4.51040400	1.23495900
H	3.89477700	-4.75923300	1.25925300
H	5.52741500	-5.44937100	1.24759100
H	5.20404000	-3.95359200	2.14574100
C	4.94647400	-4.49632100	-1.29607400
H	3.87924600	-4.74567400	-1.30980300
H	5.17678000	-3.92902600	-2.20342900
H	5.51236000	-5.43460500	-1.32632500
H	-8.24106800	0.15391200	0.02692700
H	-5.83936000	-0.87718200	0.00632400
C	-5.30225200	3.68800600	0.02806300
C	-4.96185900	4.51036400	-1.23504500
H	-5.52734400	5.44933000	-1.24772800
H	-3.89470200	4.75919500	-1.25928800
H	-5.20391800	3.95353000	-2.14582700
C	-4.94653100	4.49633700	1.29598900
H	-3.87930400	4.74569100	1.30976800
H	-5.51241900	5.43462200	1.32619000
H	-5.17688400	3.92906300	2.20334500

BO5_T_1

C	-0.83267300	5.64603100	0.09441400
C	-1.90775000	4.74691900	0.08308900
C	-1.67878800	3.38228200	0.06365000
C	-0.34558400	2.83611900	0.05424200
C	0.72277100	3.79316300	0.06677900
C	0.48303900	5.15362400	0.08623400
C	-0.15282600	1.43401400	0.03229500

C	-1.32665800	0.55014700	0.02263600
C	-1.15417800	-0.80746000	-0.01112900
H	-2.04366600	-1.43004300	-0.02084500
C	0.15283600	-1.43402600	-0.03219500
C	1.32666700	-0.55015900	-0.02249900
C	1.15418800	0.80744800	0.01124900
H	-1.02100400	6.71510200	0.10957900
H	-2.93627700	5.09354500	0.08928500
H	1.74761200	3.44021800	0.06142900
H	1.32062000	5.84546200	0.09542800
H	2.04367700	1.43002900	0.02095200
C	0.34559500	-2.83613100	-0.05422200
C	1.67879900	-3.38229200	-0.06368600
H	-1.74760000	-3.44023100	-0.06141400
C	-0.72275900	-3.79317400	-0.06679800
C	1.90776300	-4.74692800	-0.08320700
C	0.83268600	-5.64604000	-0.09456500
C	-0.48302600	-5.15363500	-0.08633400
H	2.93629100	-5.09355200	-0.08944000
H	1.02101900	-6.71511000	-0.10979400
H	-1.32060700	-5.84547300	-0.09555400
O	-2.79138800	2.57618900	0.05432800
B	-2.72928400	1.19494900	0.04049400
B	2.72929200	-1.19495900	-0.04039800
O	2.79140100	-2.57619800	-0.05434000
C	-4.10077500	0.42116100	0.03114200
C	-4.78020200	0.19122700	-1.18624800
C	-4.67427100	-0.05182800	1.23117600
C	-5.99424800	-0.50125900	-1.18584500
C	-5.89179000	-0.74069400	1.19674700
C	-6.56982300	-0.97441800	-0.00226300
H	-6.50339300	-0.67697500	-2.13226700
H	-6.32142800	-1.10185100	2.12971500
C	4.10077900	-0.42116200	-0.03104500
C	4.67440800	0.05149500	-1.23117000
C	4.78004200	-0.19085700	1.18633700
C	5.89189700	0.74037200	-1.19681400
C	5.99410700	0.50164500	1.18586100
C	6.56981300	0.97443700	0.00222300
H	6.32161700	1.10129100	-2.12984000
H	6.50314300	0.67764500	2.13228600
C	4.20239300	-0.69108200	2.49453000
H	3.19169300	-0.29898300	2.66637100
H	4.12730400	-1.78560200	2.50541200

H	4.82306400	-0.39196300	3.34514700
C	3.99499900	-0.19860500	-2.56224500
H	3.97386800	-1.26924300	-2.80417100
H	2.95471700	0.14863800	-2.55905700
H	4.51556300	0.31275800	-3.37826500
C	7.89842800	1.69437000	0.01984500
H	8.00248500	2.32406600	0.91058400
H	8.73632100	0.98411200	0.02766300
H	8.02118200	2.33188700	-0.86214500
C	-7.89837600	-1.69446400	-0.02006700
H	-8.73627400	-0.98428700	-0.03249700
H	-8.00045600	-2.32756700	-0.90863800
H	-8.02302300	-2.32863900	0.86405300
C	-3.99481100	0.19796000	2.56228700
H	-2.95442800	-0.14896400	2.55888200
H	-3.97398600	1.26851200	2.80462600
H	-4.51515700	-0.31386400	3.37815600
C	-4.20267600	0.69182500	-2.49435300
H	-4.12763900	1.78634900	-2.50495100
H	-3.19196900	0.29981600	-2.66636800
H	-4.82339400	0.39289100	-3.34500100

BO6_T_1

C	-1.24789600	-0.85338900	0.06293900
C	-1.25111700	-2.30549500	0.04882900
C	-2.49992900	-3.01334700	0.07763000
C	-3.72834400	-2.25811900	0.11415900
C	-0.00042100	-0.20559200	0.00008300
C	-0.00449700	-2.98438400	-0.00017100
C	1.24411500	-2.30914500	-0.04893800
C	1.24521300	-0.85700800	-0.06289400
C	3.72147900	-2.26901700	-0.11445700
C	2.49081000	-3.02066600	-0.07771100
H	0.00119800	0.88470300	0.00020400
H	-0.00609400	-4.06923500	-0.00048300
C	-4.97138800	-2.89299600	0.13902300
C	-2.63325600	-4.43246800	0.06989300
C	-3.86992000	-5.04813300	0.09547100
C	-5.05282900	-4.28209500	0.12999700
H	-5.85867700	-2.26727100	0.16685000
H	-1.74347800	-5.05337400	0.04272600
H	-3.92938300	-6.13370200	0.08876400
H	-6.02243100	-4.77190200	0.14996600
C	4.96263800	-2.90763500	-0.13936500

C	5.03994500	-4.29694800	-0.13010500
C	3.85476100	-5.05950300	-0.09525800
C	2.61994200	-4.44019200	-0.06964200
H	5.85178400	-2.28456100	-0.16739600
H	6.00808200	-4.78964500	-0.15011300
H	3.91104300	-6.14523900	-0.08829900
H	1.72833300	-5.05844500	-0.04207800
B	2.56752100	-0.12137500	-0.11985100
O	3.73775400	-0.91590900	-0.12828200
O	-3.74058900	-0.90487600	0.12782200
B	-2.56816900	-0.11388800	0.11968700
C	2.80563400	1.43492700	-0.14256700
C	2.51968000	2.20840600	-1.29025200
C	3.31757500	2.08713000	1.00410900
C	2.73971300	3.59116400	-1.27190200
C	3.51884800	3.47022000	0.99110700
C	3.23759600	4.24280800	-0.14076300
H	2.51680400	4.17367200	-2.16546600
H	3.90400700	3.95802900	1.88652500
C	-2.80147900	1.44314900	0.14257100
C	-2.51384900	2.21546400	1.29057400
C	-3.31063800	2.09722300	-1.00431800
C	-2.72949700	3.59894300	1.27237500
C	-3.50756900	3.48091100	-0.99114400
C	-3.22457100	4.25239200	0.14108000
H	-2.50533500	4.18053600	2.16621800
H	-3.89064300	3.97011100	-1.88670100
C	3.64708300	1.29906300	2.25502300
H	4.46450400	0.58993600	2.07450500
H	3.94906400	1.95986600	3.07429600
H	2.78583800	0.71150600	2.59991800
C	1.98969100	1.56520300	-2.55635500
H	1.94942600	2.28803900	-3.37806600
H	2.62193100	0.72856700	-2.87992700
H	0.97820100	1.16357000	-2.41817000
C	3.49234100	5.73181800	-0.14598100
H	3.25679500	6.18221100	0.82529400
H	4.54761900	5.95426200	-0.35736800
H	2.89263800	6.23870500	-0.90978500
C	-1.98674000	1.57036100	2.55691800
H	-1.94469600	2.29293900	3.37876500
H	-2.62186200	0.73570400	2.87995700
H	-0.97645600	1.16551400	2.41930700
C	-3.64171400	1.31046400	-2.25564400

H	-4.46078800	0.60311400	-2.07565900
H	-3.94200300	1.97232900	-3.07468300
H	-2.78172500	0.72111500	-2.60062800
C	-3.47445700	5.74222500	0.14627900
H	-2.87759400	6.24637600	0.91409100
H	-3.23166000	6.19278700	-0.82315300
H	-4.53023900	5.96794900	0.35153500

Supplementary References

Supplementary references for Pt(II)-based deep-blue OLEDs:

[1] Li, K., Guan, X., Ma, C.-W., Lu, W., Chen, Y. & Che, C.-M. Blue electrophosphorescent organoplatinum(II) complexes with dianionic tetradentate bis(carbene) ligands. *Chem. Commun.* **47**, 9075–9077 (2011).

[2] Fleetham, T., Wang, Z. & Li, J. Efficient deep blue electrophosphorescent devices based on platinum(II) bis(n-methyl-imidazolyl)benzene chloride. *Org. Electron.* **13**, 1430−1435 (2012).

[3] Hang, X.-C., Fleetham, T., Turner, E., Brooks, J. & Li, J. Highly efficient blue-emitting cyclometalated platinum(II) complexes by judicious molecular design. *Angew. Chem. Int. Ed.* **52**, 6753−6756 (2013).

[4] Fleetham, T., Li, G., Wen, L. & Li, J. Efficient "pure" blue OLEDs employing tetradentate Pt complexes with a narrow spectral bandwidth. *Adv. Mater.* **26**, 7116−7121 (2014).

[5] Wang, X. et al. Highly Efficient Deep-Blue Electrophosphorescent Pt(II) Compounds with Non-Distorted Flat Geometry: Tetradentate versus Macrocyclic Chelate Ligands. *Adv. Funct. Mater.* **27**, 1604318 (2017).

[6] Ma, H. et al. High-color-purity and efficient solution-processable blue phosphorescent light-emitting diodes with Pt(II) complexes featuring $^3\pi\pi^*$ transitions. *Mater. Chem. Front.* **3**, 2448−2454 (2019).

[7] Zhu, L. et al. Tetradentate Pt(II) complexes for spectrum-stable deep-blue and white electroluminescence. *Adv. Optical Mater.* **8**, 2000406 (2020).

[8] Chen, Y. et al. Ultrapure blue phosphorescent organic light-emitting diodes employing a twisted Pt(II) complex. *ACS Appl. Mater. Interfaces* **13**, 52833−52839 (2021).

[9] Huh, J.-S., Sung, M.-J., Kwon, S.-K., Kim, Y.-H. & Kim, J.-J. Highly efficient deep blue phosphorescent OLEDs based on tetradentate Pt(II) complexes containing adamantyl spacer groups. Adv. *Funct. Mater.* **31**, 2100967 (2021).

[10] Park, H.-J., Jang, J.-H., Lee, J.-H. & Hwang, D.-H. Highly efficient deep-blue phosphorescent OLEDs based on a trimethylsilyl-substituted tetradentate Pt(II) complex. *ACS Appl. Mater. Interfaces* **14**, 34901–34908 (2022).

[11] Huh, J.-S. et al. Control of the horizontal dipole ratio and emission color of deep blue tetradentate Pt(II) complexes using aliphatic spacer groups. *Chem. Eng. J.* **450**, 137836 (2022).

[12] Sun, J. et al. Exceptionally stable blue phosphorescent organic light-emitting diodes. *Nat. Photonics*. **16,** 212−218 (2022).

Supplementary reference for Ir(III)-based deep-blue OLEDs:

[13] Chang, C.-F. et al. Highly Efficient Blue-Emitting Iridium(III) Carbene Complexes and Phosphorescent OLEDs. *Angew. Chem. Int. Ed.* **47**, 4542–4545 (2008).

[14] Chiu B.-Y.-C. et al. En Route to High External Quantum Efficiency (~12%), Organic True-Blue-Light-Emitting Diodes Employing Novel Design of Iridium (III) Phosphors. *Adv. Mater.* **21**, 2221–2225 (2009).

[15] Jeon, S. O., Yook, K. S., Joo, C. & W. J. Y. Lee, Phenylcarbazole-Based Phosphine Oxide Host Materials for High Efficiency in Deep Blue Phosphorescent Organic Light-Emitting Diodes. *Adv. Funct. Mater*. **19**, 3644−3649 (2009).

[16] Sasabe, B. H. et al. High-Efficiency Blue and White Organic Light-Emitting Devices Incorporating a Blue Iridium Carbene Complex. *Adv. Mater*. **22**, 5003−5007 (2010).

[17] Hsieh, C.-H. et al. Design and Synthesis of Iridium Bis (carbene) Complexes for Efficient Blue Electrophosphorescence. Cheng, *Chem. Eur. J.* **17**, 9180–9187 (2011).

[18] Lu, K.-Y. et al. Wide-Range Color Tuning of Iridium Biscarbene Complexes from Blue to Red by Different N^N Ligands: an Alternative Route for Adjusting the Emission Colors. *Adv. Mater.* **23**, 4933–4937 (2011).

[19] Jeon, S. O., Jang, S. E., Son, H. S. & Lee, J. Y. External Quantum Efficiency Above 20% in Deep Blue Phosphorescent Organic Light-Emitting Diodes. *Adv. Mater*. **23**, 1436−1441 (2011).

[20] Park, M. S. & Lee, J. Y. Indolo Acridine-Based Hole-Transport Materials for Phosphorescent OLEDs with Over 20% External Quantum Efficiency in Deep Blue and Green. *Chem. Mater*. **23**, 4338−4343 (2011).

[21] Cho, Y. J. & Lee, J. Y. Modified N’N’-Dicarbazolyl-3,5-benzene as a High Triplet Energy Host Material for Deep-Blue Phosphorescent Organic Light-Emitting Diodes. *Chem. Eur. J.* **17**, 11415−11418 (2011).

[22] Jeong, S. H. et al. Comparison of Bipolar Hosts and Mixed-Hosts as Host Structures for Deep-Blue Phosphorescent Organic Light Emitting Diodes. *Chem. Asian J.* **6**, 2895−2898 (2011).

[23] Park, M. S., Choi, D. H., Lee, B. S. & Lee, J. Y. Fused indole derivatives as high triplet energy hole transport materials for deep blue phosphorescent organic light-emitting diodes. *J. Mater. Chem.* **22**, 3099–3104 (2012).

[24] Yang, C.-H. et al. Deep-Blue-Emitting Heteroleptic Iridium(III) Complexes Suited for Highly Efficient Phosphorescent OLEDs. *Chem. Mater*. **24**, 3684−369 (2012).

[25] Lee, S. et al. Deep-Blue Phosphorescence from Perfluoro Carbonyl-Substituted Iridium Complexes. *J. Am. Chem. Soc*. **135**, 14321−14328 (2013).

[26] Kang, Y. et al. Highly efficient blue phosphorescent and electroluminescent Ir(III) compounds. *J. Mater. Chem. C*. **1**, 441−450 (2013).

[27] Kim, J.-B. et al. Highly efficient deep-blue phosphorescence from heptafluoropropyl-substituted iridium complexes. *Chem. Commun.* 51, 58−61(2015).

[28] Lee, J. et al. Deep blue phosphorescent organic light-emitting diodes with very high brightness and efficiency. *Nat. Mater.* **15**, 92–98 (2016).

[29] Feng, Y., Zhuang, X. Zhu, D., Liu, Y., Wang, Y. & Bryce, M. R. Rational design and characterization of heteroleptic phosphorescent iridium(III) complexes for highly efficient deep-blue OLEDs. *J. Mater. Chem. C*. **4**, 10246–10252 (2016).

[30] Cho, Y.-J. et al. Probing photophysical properties of isomeric N-heterocyclic carbene Ir(III) complexes and their applications to deep-blue phosphorescent organic light-emitting diodes. *J. Mater. Chem. C*. **5**, 1651−1659 (2017).

[31] Pal, A. K. et al. High-Efficiency Deep-Blue-Emitting Organic Light-Emitting Diodes Based on Iridium(III) Carbene Complexes. *Adv. Mater.* **30**, 1804231 (2018).

[32] Li, X. et al. Deep Blue Phosphorescent Organic Light-Emitting Diodes with CIEy Value of 0.11 and External Quantum Efficiency up to 22.5%. *Adv. Mater.* **30**, 1705005 (2018).

[33] Shin, H. et al. Controlling Horizontal Dipole Orientation and Emission Spectrum of Ir Complexes by Chemical Design of Ancillary Ligands for Efficient Deep-Blue Organic Light-Emitting Diodes. *Adv. Mater*. **31**, 1808102 (2019).

[34] Park, H. Y. et al. External Quantum Efficiency Exceeding 24% with CIE_y Value of 0.08 using a Novel Carbene-Based Iridium Complex in Deep-Blue Phosphorescent Organic Light-Emitting Diodes. *Adv. Mater.* **32**, 2002120 (2020).

[35] Jung, M., Lee, K. H., Lee, J. Y. & Kim, T. A bipolar host based high triplet energy electroplex for an over 10 000 h lifetime in pure blue phosphorescent organic light-emitting diodes. *Mater. Horiz.* **7**, 559–565 (2020).

[36] You, C. et al. Homoleptic Ir(III) Phosphors with 2-Phenyl-1,2,4-triazol-3-ylidene Chelates for Efficient Blue Organic Light-Emitting Diodes. *ACS Appl. Mater. Interfaces* **13**, 59023–59034 (2021).

[37] Lan, Y., Liu, D., Li, J., Meia, Y. & Tian, H. Blue heteroleptic iridium(III) complexes for OLEDs: simultaneous optimization of color purity and efficiency. *J. Mater. Chem. C*, **10**, 17965–17973 (2022).

[38] Zhang, M. et al. Saturated-blue-emitting [3+2+1] coordinated iridium(III) complexes for vacuum-deposited organic light-emitting devices. *J. Mater. Chem. C*. **10**, 14616–14625 (2022).

[39] Qin, Y. et al. Facially Coordinated, Tris-bidentate Purin-8-ylidene Ir(III) Complexes for Blue Electrophosphorescence and Hyperluminescence. *Adv. Optical Mater*. **10**, 2201633 (2022).

[40] Kumaresan, R. et al. High Performance Solution-Processed Deep-Blue Phosphorescence Organic Light-Emitting Diodes with EQE Over 24% by Employing New Carbenic Ir(III) Complexes. *Adv. Optical Mater.* **10**, 2101686 (2022).

[41] Wu, C. et al. Blue Iridium (III) Phosphorescent OLEDs with High Brightness Over 10 000 cd m^{-2} and Ultralow Efficiency Roll-Off. *Adv. Optical Mater.* **11**, 2201998 (2023).

[42] Mackenzie, C. F. R., Zhang, L., Cordes, D. B. Slawin, A. M. Z., Samuel, I. D.W. & Zysman-Colman, E. Bulky Iridium NHC Complexes for Bright, Efficient Deep-Blue OLEDs. *Adv. Optical Mater.* **11**, 2201495 (2023).

Supplementary reference for MR-BN-PAHs-based deep-blue OLEDs:

[43] Hatakeyama T. et al. Ultrapure Blue Thermally Activated Delayed Fluorescence Molecules: Efficient HOMO–LUMO Separation by the Multiple Resonance Effect. *Adv. Mater.* **28**, 2777 (2016)

[44] Liang, X. et al. Peripheral Amplification of Multi-Resonance Induced Thermally Activated Delayed Fluorescence for Highly Efficient OLEDs. *Angew. Chem., Int. Ed.* **57**, 11316–11320 (2018).

[45] Kondo, Y. et al. Narrowband deep-blue organic light-emitting diode featuring an organoboron-based emitter. *Nature Photon.* **13**, 678–682 (2019).

[46] Han, S. H., Jeong, J. H., Yoo, J. W. & Lee, J. Y. Ideal blue thermally activated delayed fluorescence emission assisted by a thermally activated delayed fluorescence assistant dopant through a fast reverse intersystem crossing mediated cascade energy transfer process. *J. Mater. Chem. C.* **7**, 3082–3089 (2019).

[47] Kim, J. H., Chung, W. J., Kim, J. & Lee, J. Y. Concentration quenchingeresistant multiresonance thermally activated delayed fluorescence emitters. *Mater. Today Energy* **21**, 100792 (2021).

[48] Park, J. et al. Asymmetric Blue Multiresonance TADF Emitters with a Narrow Emission Band. *ACS Appl. Mater. Interfaces* **13**, 45798 (2021).

[49] Tanaka, H. et al. Hypsochromic Shift of Multiple-Resonance-Induced Thermally Activated Delayed Fluorescence by Oxygen Atom Incorporation. *Angew. Chem., Int. Ed.* **60**, 17910–17914 (2021).

[50] Yang, M. et al. Wide-Range Color Tuning of Narrowband Emission in Multi-resonance Organoboron Delayed Fluorescence Materials through Rational Imine/Amine Functionalization. *Angew. Chem., Int. Ed.* **60**, 23142–23147 (2021).

[51] Wang, Y. et al. A periphery cladding strategy to improve the performance of narrowband emitters, achieving deep-blue OLEDs with CIEy < 0.08 and external quantum efficiency approaching 20%. *Org. Electron.* **97**, 106275 (2021).

[52] Wang, Y. et al. The selective regulation of borylation site based on one-shot electrophilic C–H borylation reaction, achieving highly efficient narrowband organic light-emitting diodes. *Chem. Eng.*

J. **431**, 133221 (2022).

[53] Qiu, Y. et al. Narrowing the Electroluminescence Spectra of Multiresonance Emitters for High-Performance Blue OLEDs by a Peripheral Decoration Strategy. *ACS Appl. Mater. Interfaces* **13**, 59035–59042 (2021).

[54] Lee, H. et al. Manipulating Spectral Width and Emission Wavelength towards Highly Efficient Blue Asymmetric Carbazole Fused Multi-Resonance Emitters. *ACS Appl. Mater. Interfaces* **14**, 36927 (2022).

[55] Cheon, H. J. Woo, S. J. Baek, S. H., Lee, J. H. & Kim, Y. H. Dense Local Triplet States and Steric Shielding of a Multi-Resonance TADF Emitter Enable High-Performance Deep-Blue OLEDs. *Adv. Mater.* **34**, 2207416 (2022).

[56] Fan, T. et al. One-Shot Synthesis of B/N-Doped Calix[4]arene Exhibiting Narrowband Multiple Resonance Fluorescence. *Angew. Chem., Int. Ed.* **61**, e202213585 (2022).

[57] Lv, X. et al. Extending the π-Skeleton of Multi-Resonance TADF Materials towards High-Efficiency Narrowband Deep-Blue Emission. *Angew. Chem., Int. Ed.* **61**, e202201588 (2022).

[58] Han, J. M. et al. Simple Molecular Design Strategy for Multiresonance Induced TADF Emitter: Highly Efficient Deep Blue to Blue Electroluminescence with High Color Purity. *Adv. Opt. Mater.* **10**, 2102092 (2022).

[59] Park, I. S., Min, H. & Yasuda, T. Ultrafast Triplet–Singlet Exciton Interconversion in Narrowband Blue Organoboron Emitters Doped with Heavy Chalcogens. *Angew. Chem., Int. Ed.* **61**, e202205684 (2022).

[60] Park, I. S., Yang, M., Shibata, H., Amanokura, N. & Yasuda, T. Achieving Ultimate Narrowband and Ultrapure Blue Organic Light-Emitting Diodes Based on Polycyclo-Heteraborin Multi-Resonance Delayed-Fluorescence Emitters. *Adv. Mater.* **34**, 2107951 (2022).

[61] Naveen, K. R. et al. Deep blue diboron embedded multi-resonance thermally activated delayed fluorescence emitters for narrowband organic light emitting diodes. *Chem. Eng. J.* **432**, 134381(2022).

[62] Oda, S. et al. Ultra-Narrowband Blue Multi-Resonance Thermally Activated Delayed Fluorescence Materials. *Adv. Sci.* **10**, 2205070 (2022).

[63] Park, J., Kim, K. J. Lim, J., Kim, T., & Lee, J. Y. High Efficiency of over 25% and Long Device Lifetime of over 500 h at 1000 nit in Blue Fluorescent Organic Light-Emitting Diodes. *Adv. Mater.* **34**, 2108581 (2022).

[64] Cheon, H. J., Shin, Y.-S. Park, N.-H., Lee, J.-H. & Kim, Y.-H. Boron-Based Multi-Resonance TADF Emitter with Suppressed Intermolecular Interaction and Isomer Formation for Efficient Pure

Blue OLEDs. *Small* **18**, 2107574 (2022).

[65] Stavrou, K. et al. Emission and Absorption Tuning in TADF B,N-Doped Heptacenes: Toward Ideal-Blue Hyperfluorescent OLEDs. *Adv. Opt. Mater.* **10**, 2200688 (2022).

[66] Li, Q. et al. Boron-, Sulfur- and Nitrogen-Doped Polycyclic Aromatic Hydrocarbon Multiple Resonance Emitters for Narrow-Band Blue Emission. *Chem. Eur. J.* **28**, e202104214 (2022).

[67] Liu, G., Sasabe, H., Kumada, K., Arai, H. & Kido, J. Nonbonding/Bonding Molecular Orbital Regulation of Nitrogen-Boron-Oxygen-embedded Blue/Green Multiresonant TADF Emitters with High Efficiency and Color Purity. *Chem. Eur. J.* **28**, e202201605 (2022).

[68] Lee, Y. & Hong, J.-I. Multiple resonance thermally activated delayed fluorescence enhanced by halogen atoms. *J. Mater. Chem. C.* **10,** 11855–11861 (2022).

[69] Naveen, K. R. et al. Modular design for constructing narrowband deep-blue multiresonant thermally activated delayed fluorescent emitters for efficient organic light emitting diodes. *Chem. Eng. J.* **451**, 138498 (2023).

[70] Suresh, S. M. et al. Judicious Heteroatom Doping Produces High-Performance Deep-Blue/Near-UV Multiresonant Thermally Activated Delayed Fluorescence OLEDs. *Adv. Mater.* **2023**, 2300997 (2023).

[71] Suresh, S. M. et al. A Deep-Blue-Emitting Heteroatom-Doped MR-TADF Nonacene for High-Performance Organic Light-Emitting Diodes. *Angew. Chem., Int. Ed.* **62**, e202215522 (2023).

[72] Park, J. et al. A Design Strategy for Multiple Resonance-Induced Pure Violet Thermally Activated Delayed Fluorescence Emitters with a Narrow Emission Band. *Adv. Opt. Mater.* 2301626 (2023).

[73] Naveen, K. R., Oh, J. H., Lee, H. & Kwon, J. H. Tailoring Extremely Narrow FWHM in Hypsochromic and Bathochromic Shift of Polycyclo-Heteraborin MR-TADF Materials for High-Performance OLEDs. *Angew. Chem., Int. Ed.* e202306768 (2023).
