# Supplementary material for: Double boron–oxygen-fused polycyclic aromatic hydrocarbons: skeletal editing and applications as organic optoelectronic materials
Source: Nat Commun. 2023 Nov 4;14:7089. doi: 10.1038/s41467-023-42973-1 (PMC10625603; doi:10.1038/s41467-023-42973-1)
Supplement: Supplementary file 3 — Description of Additional Supplementary Files [file 41467_2023_42973_MOESM3_ESM.pdf]

Description of Additional Supplementary Files

File name: Supplementary Data 1

Description: CheckCIF report of BO1a (CCDC1954663)

File name: Supplementary Data 2

Description: CheckCIF report of BO2 (CCDC1954662)

File name: Supplementary Data 3

Description: CheckCIF report of BO3a (CCDC1954665)

File name: Supplementary Data 4

Description: CheckCIF report of BO3b-OH (CCDC2290997)

File name: Supplementary Data 5

Description: CheckCIF report of BO4b (CCDC1954661)

File name: Supplementary Data 6

Description: CheckCIF report of BO4d (CCDC1966566)

File name: Supplementary Data 7

Description: CheckCIF report of BO4e (CCDC1968760)

File name: Supplementary Data 8

Description: CheckCIF report of BO3d (CCDC1954664)

File name: Supplementary Data 9

Description: CheckCIF report of BO5 (CCDC2280610)

File name: Supplementary Data 10

Description: CheckCIF report of BO6 (CCDC2029823)
