# Supplementary material for: Double boron–oxygen-fused polycyclic aromatic hydrocarbons: skeletal editing and applications as organic optoelectronic materials
Source: Nat Commun. 2023 Nov 4;14:7089. doi: 10.1038/s41467-023-42973-1 (PMC10625603; doi:10.1038/s41467-023-42973-1)
Supplement: Supplementary file 5 — Supplementary Data 2 [file 41467_2023_42973_MOESM5_ESM.pdf]

checkCIF (basic structural check) running

Checking for embedded fcf data in CIF ...
Found embedded fcf data in CIF. Extracting fcf data from uploaded CIF, please wait . . . .

checkCIF/PLATON (basic structural check)

Structure factors have been supplied for datablock(s) mo_d8v19820_0m

THIS REPORT IS FOR GUIDANCE ONLY. IF USED AS PART OF A REVIEW PROCEDURE FOR PUBLICATION, IT SHOULD NOT REPLACE THE EXPERTISE OF AN EXPERIENCED CRYSTALLOGRAPHIC REFEREE.

No syntax errors found.
Please wait while processing ....

CIF dictionary
Interpreting this report

Structure factor report

Datablock: mo_d8v19820_0m

Bond precision:	C-C = 0.0041 A		Wavelength=0.71073
Cell:	a=8.0150(3)	b=14.7515(6)	c=24.6982(8)
	alpha=90	beta=90	gamma=90
Temperature: 193 K			

	Calculated	Reported
Volume	2920.15(19)	2920.15(19)
Space group	P 21 21 21	P 21 21 21
Hall group	P 2ac 2ab	P 2ac 2ab
Moiety formula	C36 H32 B2 O2	?
Sum formula	C36 H32 B2 O2	C36 H32 B2 O2
Mr	518.24	518.23
Dx,g cm-3	1.179	1.179
Z	4	4
Mu (mm-1)	0.070	0.070
F000	1096.0	1096.0
F000'	1096.43	
h,k,lmax	9,18,30	9,18,30
Nref	5734[3258]	5706
Tmin,Tmax	0.987,0.991	0.495,0.746
Tmin'	0.986	

Correction method= # Reported T Limits: Tmin=0.495 Tmax=0.746 AbsCorr = MULTI-SCAN

Data completeness= 1.75/1.00 Theta(max)= 25.985

R(reflections)= 0.0483(4893) wR2(reflections)= 0.1313(5706)

S = 1.032 Npar= 368

The following ALERTS were generated. Each ALERT has the format
test-name_ALERT_alert-type_alert-level.
Click on the hyperlinks for more details of the test.

Alert level C

STRVA01_ALERT_2_C Chirality of atom sites is inverted?
From the CIF: _refine_ls_abs_structure_Flack 2.200
From the CIF: _refine_ls_abs_structure_Flack_su 0.900

PLAT340_ALERT_3_C Low Bond Precision on C-C Bonds .... 0.00413 Ang.
PLAT907_ALERT_2_C Flack x > 0.5, Structure Needs to be Inverted? . 2.20 Check
PLAT911_ALERT_3_C Missing FCF Refl Between Thmin & STh/L= 0.600 15 Report
PLAT913_ALERT_3_C Missing # of Very Strong Reflections in FCF .... 6 Note

Alert level G

PLAT032_ALERT_4_G Std. Uncertainty on Flack Parameter Value High . 0.900 Report
PLAT883_ALERT_1_G No Info/Value for _atom_sites_solution_primary . Please Do !
PLAT910_ALERT_3_G Missing # of FCF Reflection(s) Below Theta(Min). 3 Note
PLAT916_ALERT_2_G Hooft y and Flack x Parameter Values Differ by . 0.25 Check
PLAT933_ALERT_2_G Number of OMIT Records in Embedded .res File ... 1 Note
PLAT978_ALERT_2_G Number C-C Bonds with Positive Residual Density. 6 Info

0 **ALERT level A** = Most likely a serious problem - resolve or explain
0 **ALERT level B** = A potentially serious problem, consider carefully
5 **ALERT level C** = Check. Ensure it is not caused by an omission or oversight
6 **ALERT level G** = General information/check it is not something unexpected

1 ALERT type 1 CIF construction/syntax error, inconsistent or missing data
5 ALERT type 2 Indicator that the structure model may be wrong or deficient
4 ALERT type 3 Indicator that the structure quality may be low
1 ALERT type 4 Improvement, methodology, query or suggestion
0 ALERT type 5 Informative message, check

It is advisable to attempt to resolve as many as possible of the alerts in all categories. Often the minor alerts point to easily fixed oversights, errors and omissions in your CIF or refinement strategy, so attention to these fine details can be worthwhile. In order to resolve some of the more serious problems it may be necessary to carry out additional measurements or structure refinements. However, the purpose of your study may justify the reported deviations and the more serious of these should normally be commented upon in the discussion or experimental section of a paper or in the "special_details" fields of the CIF. checkCIF was carefully designed to identify outliers and unusual parameters, but every test has its limitations and alerts that are not important in a particular case may appear. Conversely, the absence of alerts does not guarantee there are no aspects of the results needing attention. It is up to the individual to critically assess their own results and, if necessary, seek expert advice.

Publication of your CIF in IUCr journals

A basic structural check has been run on your CIF. These basic checks will be run on all CIFs submitted for publication in IUCr journals (*Acta Crystallographica*, *Journal of Applied Crystallography*, *Journal of Synchrotron Radiation*); however, if you intend to submit to *Acta Crystallographica Section C* or *E* or *IUCrData*, you should make sure that full publication checks are run on the final version of your CIF prior to submission.

Publication of your CIF in other journals

Please refer to the *Notes for Authors* of the relevant journal for any special instructions relating to CIF submission.

PLATON version of 07/08/2019; check.def file version of 30/07/2019

Datablock mo_d8v19820_0m - ellipsoid plot

Download CIF editor (publCIF) from the IUCr
Download CIF editor (enCIFer) from the CCDC
Test a new CIF entry
