# Supplementary material for: Double boron–oxygen-fused polycyclic aromatic hydrocarbons: skeletal editing and applications as organic optoelectronic materials
Source: Nat Commun. 2023 Nov 4;14:7089. doi: 10.1038/s41467-023-42973-1 (PMC10625603; doi:10.1038/s41467-023-42973-1)

checkCIF/PLATON report

Structure factors have been supplied for datablock(s) mo_d8v19628_0m

THIS REPORT IS FOR GUIDANCE ONLY. IF USED AS PART OF A REVIEW PROCEDURE FOR PUBLICATION, IT SHOULD NOT REPLACE THE EXPERTISE OF AN EXPERIENCED CRYSTALLOGRAPHIC REFEREE.

No syntax errors found. CIF dictionary Interpreting this report

Datablock: mo_d8v19628_0m

Bond precision:	C-C = 0.0050 A		Wavelength=0.71073
Cell:	a=30.8251(8)	b=7.5638(2)	c=17.0835(4)
	alpha=90	beta=92.974(1)	gamma=90
Temperature:	293 K		

	Calculated	Reported
Volume	3977.74(17)	3977.74(17)
Space group	C 2	C 1 2 1
Hall group	C 2y	C 2y
Moiety formula	C44 H36 B2 O2 [+ solvent]	C44 H36 B2 O2
Sum formula	C44 H36 B2 O2 [+ solvent]	C44 H36 B2 O2
Mr	618.35	618.35
Dx,g cm-3	1.033	1.033
Z	4	4
Mu (mm-1)	0.061	0.061
F000	1304.0	1304.0
F000’	1304.51	
h,k,lmax	40,9,22	40,9,22
Nref	9120[4908]	9027
Tmin,Tmax	0.991,0.994	0.643,0.746
Tmin’	0.988	

Correction method= # Reported T Limits: Tmin=0.643 Tmax=0.746
AbsCorr = MULTI-SCAN

Data completeness= 1.84/0.99 Theta(max)= 27.487

R(reflections)= 0.0607(5984) wR2(reflections)= 0.1863(9027)

S = 1.034 Npar= 439

The following ALERTS were generated. Each ALERT has the format
      test-name_ALERT_alert-type_alert-level.
Click on the hyperlinks for more details of the test.

Alert level C

```
STRVA01_ALERT_4_C          Flack test results are meaningless.
           From the CIF: _refine_ls_abs_structure_Flack   -0.200
           From the CIF: _refine_ls_abs_structure_Flack_su    0.600
PLAT220_ALERT_2_C NonSolvent   Resd 1  C   Ueq(max)/Ueq(min) Range        3.7 Ratio
PLAT222_ALERT_3_C NonSolvent Resd 1  H   Uiso(max)/Uiso(min) Range        4.1 Ratio
PLAT340_ALERT_3_C Low Bond Precision on  C-C Bonds ...............    0.00502 Ang.
PLAT911_ALERT_3_C Missing FCF Refl Between Thmin & STh/L=    0.600         31 Report
PLAT913_ALERT_3_C Missing # of Very Strong Reflections in FCF ....          7 Note
PLAT934_ALERT_3_C Number of (Iobs-Icalc)/Sigma(W) > 10 Outliers ..          1 Check
```

Alert level G

```
PLAT032_ALERT_4_G Std. Uncertainty on Flack Parameter Value High .      0.600 Report
PLAT072_ALERT_2_G SHELXL First  Parameter in WGHT  Unusually Large       0.11 Report
PLAT199_ALERT_1_G Reported _cell_measurement_temperature ..... (K)        293 Check
PLAT200_ALERT_1_G Reported   _diffrn_ambient_temperature ..... (K)        293 Check
PLAT605_ALERT_4_G Largest Solvent Accessible VOID in the Structure        346 A**3
PLAT868_ALERT_4_G ALERTS Due to the Use of _smtbx_masks Suppressed          ! Info
PLAT883_ALERT_1_G No Info/Value for _atom_sites_solution_primary .     Please Do !
PLAT910_ALERT_3_G Missing # of FCF Reflection(s) Below Theta(Min).          4 Note
PLAT912_ALERT_4_G Missing # of FCF Reflections Above STh/L=  0.600          2 Note
PLAT916_ALERT_2_G Hooft y and Flack x Parameter Values Differ by .       0.40 Check
PLAT933_ALERT_2_G Number of HKL-OMIT Records in Embedded .res File         25 Note
PLAT941_ALERT_3_G Average HKL Measurement Multiplicity ...........        4.6 Low
PLAT978_ALERT_2_G Number C-C Bonds with Positive Residual Density.          0 Info
```

   0 **ALERT level A** = Most likely a serious problem - resolve or explain
   0 **ALERT level B** = A potentially serious problem, consider carefully
   7 **ALERT level C** = Check. Ensure it is not caused by an omission or oversight
  13 **ALERT level G** = General information/check it is not something unexpected

   3 ALERT type 1 CIF construction/syntax error, inconsistent or missing data
   5 ALERT type 2 Indicator that the structure model may be wrong or deficient
   7 ALERT type 3 Indicator that the structure quality may be low
   5 ALERT type 4 Improvement, methodology, query or suggestion
   0 ALERT type 5 Informative message, check

It is advisable to attempt to resolve as many as possible of the alerts in all categories. Often the minor alerts point to easily fixed oversights, errors and omissions in your CIF or refinement strategy, so attention to these fine details can be worthwhile. In order to resolve some of the more serious problems it may be necessary to carry out additional measurements or structure refinements. However, the purpose of your study may justify the reported deviations and the more serious of these should normally be commented upon in the discussion or experimental section of a paper or in the "special_details" fields of the CIF. checkCIF was carefully designed to identify outliers and unusual parameters, but every test has its limitations and alerts that are not important in a particular case may appear. Conversely, the absence of alerts does not guarantee there are no aspects of the results needing attention. It is up to the individual to critically assess their own results and, if necessary, seek expert advice.

Publication of your CIF in IUCr journals

A basic structural check has been run on your CIF. These basic checks will be run on all CIFs submitted for publication in IUCr journals (*Acta Crystallographica*, *Journal of Applied Crystallography*, *Journal of Synchrotron Radiation*); however, if you intend to submit to *Acta Crystallographica Section C* or *E* or *IUCrData*, you should make sure that full publication checks are run on the final version of your CIF prior to submission.

Publication of your CIF in other journals

Please refer to the *Notes for Authors* of the relevant journal for any special instructions relating to CIF submission.

PLATON version of 06/07/2023; check.def file version of 30/06/2023

Datablock mo_d8v19628_0m - ellipsoid plot

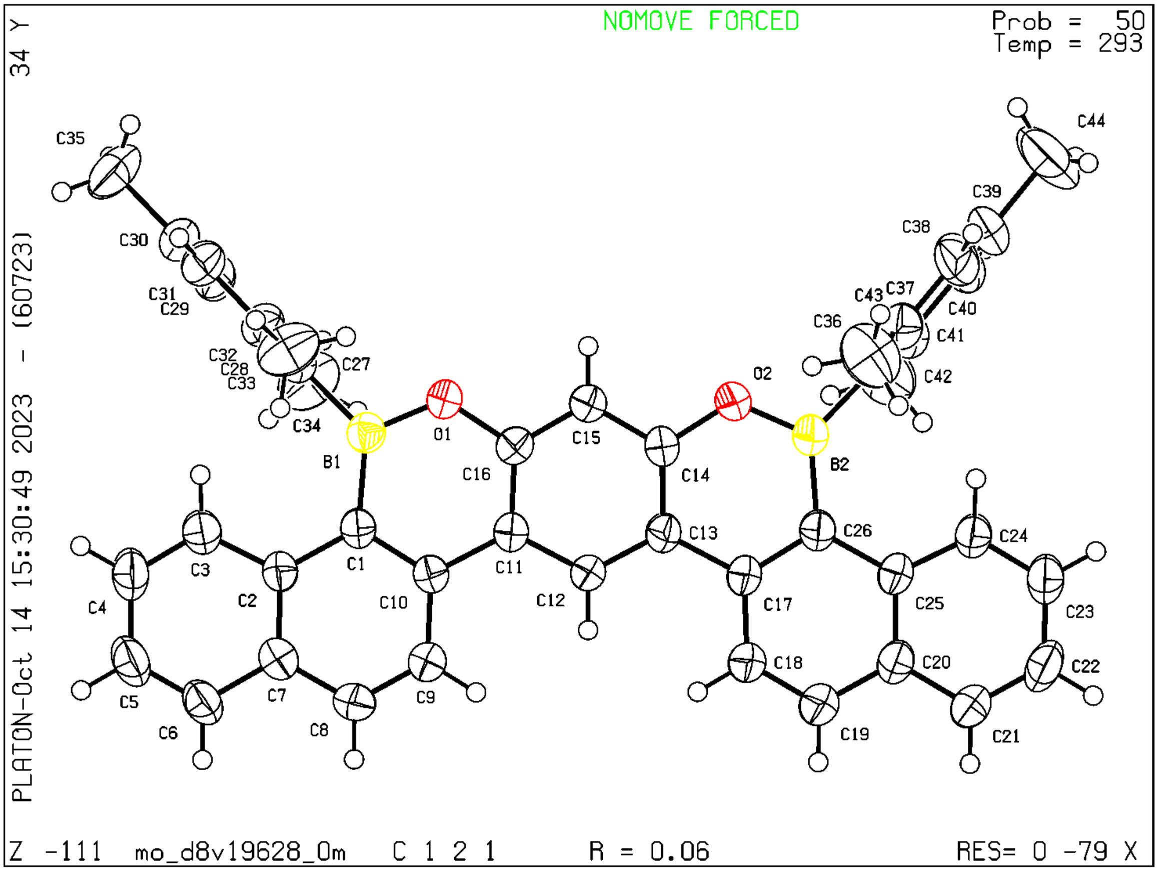

Supplement: Supplementary file 8 — Supplementary Data 5 [file 41467_2023_42973_MOESM8_ESM.pdf]
