# Supplementary material for: Double boron–oxygen-fused polycyclic aromatic hydrocarbons: skeletal editing and applications as organic optoelectronic materials
Source: Nat Commun. 2023 Nov 4;14:7089. doi: 10.1038/s41467-023-42973-1 (PMC10625603; doi:10.1038/s41467-023-42973-1)

checkCIF/PLATON report

Structure factors have been supplied for datablock(s) 191126_bo5e_0m

THIS REPORT IS FOR GUIDANCE ONLY. IF USED AS PART OF A REVIEW PROCEDURE FOR PUBLICATION, IT SHOULD NOT REPLACE THE EXPERTISE OF AN EXPERIENCED CRYSTALLOGRAPHIC REFEREE.

No syntax errors found.   CIF dictionary   Interpreting this report

Datablock: 191126_bo5e_0m

```
Bond precision:  C-C = 0.0041 A          Wavelength=0.71073

Cell:             a=14.345(6)     b=15.450(6)       c=20.591(9)
                  alpha=90        beta=99.88(2)     gamma=90
Temperature:      170 K

                Calculated                Reported
Volume          4496(3)                   4496(3)
Space group     P 21/n                    P 1 21/n 1
Hall group      -P 2yn                    -P 2yn
Moiety formula  C54 H48 B2 O2, C H2 Cl2   C54 H48 B2 O2, C H2 Cl2
Sum formula     C55 H50 B2 Cl2 O2         C55 H50 B2 Cl2 O2
Mr              835.47                    835.47
Dx,g cm-3       1.234                     1.234
Z               4                         4
Mu (mm-1)       0.187                     0.187
F000            1760.0                    1760.0
F000'           1761.81
h,k,lmax        17,19,25                  17,19,25
Nref            9240                      9215
Tmin,Tmax       0.950,0.965               0.690,0.745
Tmin'           0.930

Correction method= # Reported T Limits: Tmin=0.690 Tmax=0.745
AbsCorr = MULTI-SCAN

Data completeness= 0.997          Theta(max)= 26.405

R(reflections)= 0.0735( 6606)     wR2(reflections)= 0.2315( 9215)

S = 1.024                   Npar= 588
```

The following ALERTS were generated. Each ALERT has the format
test-name_ALERT_alert-type_alert-level.
Click on the hyperlinks for more details of the test.

Alert level C

```
PLAT220_ALERT_2_C Non-Solvent  Resd 1  C   Ueq(max)/Ueq(min) Range        3.5 Ratio
PLAT222_ALERT_3_C Non-Solv.  Resd 1  H   Uiso(max)/Uiso(min) Range        4.3 Ratio
PLAT260_ALERT_2_C Large Average Ueq of Residue Including       Cl1      0.113 Check
PLAT260_ALERT_2_C Large Average Ueq of Residue Including      Cl1A      0.175 Check
PLAT340_ALERT_3_C Low Bond Precision on  C-C Bonds ..............     0.0041 Ang.
PLAT906_ALERT_3_C Large K Value in the Analysis of Variance ......      3.758 Check
PLAT910_ALERT_3_C Missing # of FCF Reflection(s) Below Theta(Min).          6 Note
PLAT911_ALERT_3_C Missing FCF Refl Between Thmin & STh/L=    0.600         11 Report
```

Alert level G

```
PLAT002_ALERT_2_G Number of Distance or Angle Restraints on AtSite          6 Note
PLAT003_ALERT_2_G Number of Uiso or Uij Restrained non-H Atoms ...          6 Report
PLAT072_ALERT_2_G SHELXL First  Parameter in WGHT  Unusually Large       0.12 Report
PLAT176_ALERT_4_G The CIF-Embedded .res File Contains SADI Records          3 Report
PLAT177_ALERT_4_G The CIF-Embedded .res File Contains DELU Records          1 Report
PLAT178_ALERT_4_G The CIF-Embedded .res File Contains SIMU Records          1 Report
PLAT302_ALERT_4_G Anion/Solvent/Minor-Residue Disorder (Resd  2  )       100% Note
PLAT302_ALERT_4_G Anion/Solvent/Minor-Residue Disorder (Resd  3  )       100% Note
PLAT304_ALERT_4_G Non-Integer Number of Atoms in ...... Resd  2          3.70 Check
PLAT304_ALERT_4_G Non-Integer Number of Atoms in ...... Resd  3          1.31 Check
PLAT860_ALERT_3_G Number of Least-Squares Restraints ............          99 Note
PLAT912_ALERT_4_G Missing # of FCF Reflections Above STh/L=  0.600          7 Note
PLAT933_ALERT_2_G Number of OMIT Records in Embedded .res File ...          3 Note
PLAT978_ALERT_2_G Number C-C Bonds with Positive Residual Density.          8 Info
PLAT992_ALERT_5_G Repd & Actual _reflns_number_gt Values Differ by          1 Check
```

---

  0 **ALERT level A** = Most likely a serious problem - resolve or explain
  0 **ALERT level B** = A potentially serious problem, consider carefully
  8 **ALERT level C** = Check. Ensure it is not caused by an omission or oversight
 15 **ALERT level G** = General information/check it is not something unexpected

  0 ALERT type 1 CIF construction/syntax error, inconsistent or missing data
  8 ALERT type 2 Indicator that the structure model may be wrong or deficient
  6 ALERT type 3 Indicator that the structure quality may be low
  8 ALERT type 4 Improvement, methodology, query or suggestion
  1 ALERT type 5 Informative message, check

It is advisable to attempt to resolve as many as possible of the alerts in all categories. Often the minor alerts point to easily fixed oversights, errors and omissions in your CIF or refinement strategy, so attention to these fine details can be worthwhile. In order to resolve some of the more serious problems it may be necessary to carry out additional measurements or structure refinements. However, the purpose of your study may justify the reported deviations and the more serious of these should normally be commented upon in the discussion or experimental section of a paper or in the "special_details" fields of the CIF. checkCIF was carefully designed to identify outliers and unusual parameters, but every test has its limitations and alerts that are not important in a particular case may appear. Conversely, the absence of alerts does not guarantee there are no aspects of the results needing attention. It is up to the individual to critically assess their own results and, if necessary, seek expert advice.

Publication of your CIF in IUCr journals

A basic structural check has been run on your CIF. These basic checks will be run on all CIFs submitted for publication in IUCr journals (*Acta Crystallographica*, *Journal of Applied Crystallography*, *Journal of Synchrotron Radiation*); however, if you intend to submit to *Acta Crystallographica Section C* or *E* or *IUCrData*, you should make sure that full publication checks are run on the final version of your CIF prior to submission.

Publication of your CIF in other journals

Please refer to the *Notes for Authors* of the relevant journal for any special instructions relating to CIF submission.

PLATON version of 07/08/2019; check.def file version of 30/07/2019

Datablock 191126_bo5e_0m - ellipsoid plot

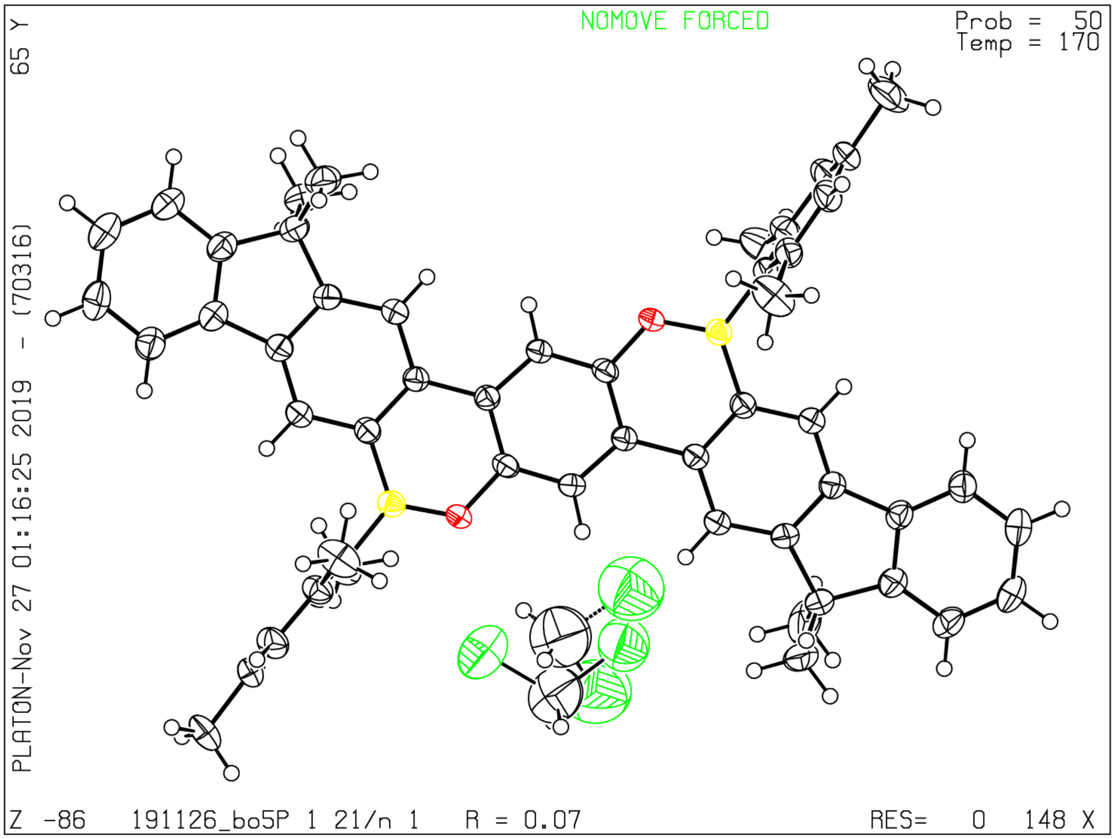

Supplement: Supplementary file 11 — Supplementary Data 8 [file 41467_2023_42973_MOESM11_ESM.pdf]
