# Supplementary material for: Double boron–oxygen-fused polycyclic aromatic hydrocarbons: skeletal editing and applications as organic optoelectronic materials
Source: Nat Commun. 2023 Nov 4;14:7089. doi: 10.1038/s41467-023-42973-1 (PMC10625603; doi:10.1038/s41467-023-42973-1)

checkCIF/PLATON report

Structure factors have been supplied for datablock(s) 230705_bo5_0m

THIS REPORT IS FOR GUIDANCE ONLY. IF USED AS PART OF A REVIEW PROCEDURE FOR PUBLICATION, IT SHOULD NOT REPLACE THE EXPERTISE OF AN EXPERIENCED CRYSTALLOGRAPHIC REFEREE.

No syntax errors found. CIF dictionary Interpreting this report

Datablock: 230705_bo5_0m

Bond precision:	C-C = 0.0022 A		Wavelength=1.34139
Cell:	a=9.1072(6)	b=10.3791(7)	c=15.0015(10)
	alpha=90	beta=105.825(2)	gamma=90
Temperature:	170 K		

	Calculated	Reported
Volume	1364.27(16)	1364.27(16)
Space group	P 21/c	P 1 21/c 1
Hall group	-P 2ybc	-P 2ybc
Moiety formula	C36 H32 B2 O2	C36 H32 B2 O2
Sum formula	C36 H32 B2 O2	C36 H32 B2 O2
Mr	518.24	518.23
Dx,g cm-3	1.262	1.262
Z	2	2
Mu (mm-1)	0.369	0.369
F000	548.0	548.0
F000’	549.10	
h,k,lmax	11,13,19	11,13,19
Nref	3122	3107
Tmin,Tmax	0.982,0.989	0.680,0.752
Tmin’	0.978	

Correction method= # Reported T Limits: Tmin=0.680 Tmax=0.752
AbsCorr = MULTI-SCAN

Data completeness= 0.995 Theta(max)= 60.561

R(reflections)= 0.0528(2223) wR2(reflections)= 0.1453(3107)

S = 1.072 Npar= 184

The following ALERTS were generated. Each ALERT has the format
test-name_ALERT_alert-type_alert-level.
Click on the hyperlinks for more details of the test.

Alert level C

```
PLAT911_ALERT_3_C Missing FCF Refl Between Thmin & STh/L=    0.600          4 Report
```

Alert level G

```
ABSMU01_ALERT_1_G  Calculation of _exptl_absorpt_correction_mu
                not performed for this radiation type.
PLAT912_ALERT_4_G Missing # of FCF Reflections Above STh/L=  0.600         11 Note
PLAT941_ALERT_3_G Average HKL Measurement Multiplicity ..........        3.7 Low
PLAT978_ALERT_2_G Number C-C Bonds with Positive Residual Density.          8 Info
```

0 **ALERT level A** = Most likely a serious problem - resolve or explain
0 **ALERT level B** = A potentially serious problem, consider carefully
1 **ALERT level C** = Check. Ensure it is not caused by an omission or oversight
4 **ALERT level G** = General information/check it is not something unexpected

1 ALERT type 1 CIF construction/syntax error, inconsistent or missing data
1 ALERT type 2 Indicator that the structure model may be wrong or deficient
2 ALERT type 3 Indicator that the structure quality may be low
1 ALERT type 4 Improvement, methodology, query or suggestion
0 ALERT type 5 Informative message, check

It is advisable to attempt to resolve as many as possible of the alerts in all categories. Often the minor alerts point to easily fixed oversights, errors and omissions in your CIF or refinement strategy, so attention to these fine details can be worthwhile. In order to resolve some of the more serious problems it may be necessary to carry out additional measurements or structure refinements. However, the purpose of your study may justify the reported deviations and the more serious of these should normally be commented upon in the discussion or experimental section of a paper or in the "special_details" fields of the CIF. checkCIF was carefully designed to identify outliers and unusual parameters, but every test has its limitations and alerts that are not important in a particular case may appear. Conversely, the absence of alerts does not guarantee there are no aspects of the results needing attention. It is up to the individual to critically assess their own results and, if necessary, seek expert advice.

Publication of your CIF in IUCr journals

A basic structural check has been run on your CIF. These basic checks will be run on all CIFs submitted for publication in IUCr journals (*Acta Crystallographica*, *Journal of Applied Crystallography*, *Journal of Synchrotron Radiation*); however, if you intend to submit to *Acta Crystallographica Section C* or *E* or *IUCrData*, you should make sure that full publication checks are run on the final version of your CIF prior to submission.

Publication of your CIF in other journals

Please refer to the *Notes for Authors* of the relevant journal for any special instructions relating to CIF submission.

PLATON version of 10/05/2023; check.def file version of 10/05/2023

Datablock 230705_bo5_0m - ellipsoid plot

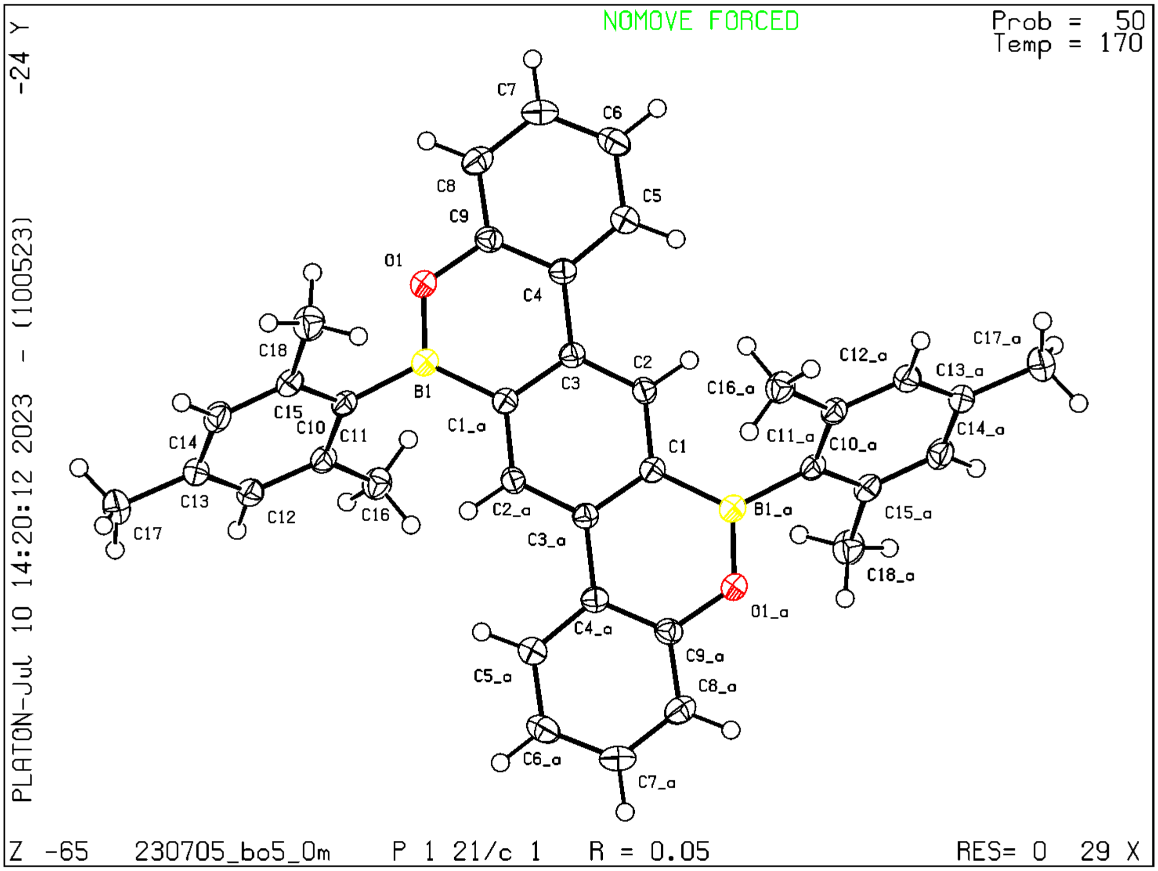

Supplement: Supplementary file 12 — Supplementary Data 9 [file 41467_2023_42973_MOESM12_ESM.pdf]
