# Supplementary material for: Double boron–oxygen-fused polycyclic aromatic hydrocarbons: skeletal editing and applications as organic optoelectronic materials
Source: Nat Commun. 2023 Nov 4;14:7089. doi: 10.1038/s41467-023-42973-1 (PMC10625603; doi:10.1038/s41467-023-42973-1)

checkCIF/PLATON report

Structure factors have been supplied for datablock(s) d8v20176

THIS REPORT IS FOR GUIDANCE ONLY. IF USED AS PART OF A REVIEW PROCEDURE FOR PUBLICATION, IT SHOULD NOT REPLACE THE EXPERTISE OF AN EXPERIENCED CRYSTALLOGRAPHIC REFEREE.

No syntax errors found. CIF dictionary Interpreting this report

Datablock: d8v20176

Bond precision:	C-C = 0.0124 A		Wavelength=0.71073
Cell:	a=19.776(2)	b=18.2753(15)	c=16.4263(15)
	alpha=90	beta=90.339(3)	gamma=90
Temperature:	193 K		

	Calculated	Reported
Volume	5936.6(9)	5936.7(9)
Space group	P c	P 1 c 1
Hall group	P -2yc	P -2yc
Moiety formula	C36 H32 B2 O2	C36 H32 B2 O2
Sum formula	C36 H32 B2 O2	C36 H32 B2 O2
Mr	518.24	518.23
Dx,g cm-3	1.160	1.160
Z	8	8
Mu (mm-1)	0.069	0.069
F000	2192.0	2192.0
F000'	2192.87	
h,k,lmax	23,21,19	23,21,19
Nref	20911[10466]	19509
Tmin,Tmax	0.988,0.993	0.524,0.746
Tmin'	0.988	

Correction method= # Reported T Limits: Tmin=0.524 Tmax=0.746
AbsCorr = MULTI-SCAN

Data completeness= 1.86/0.93 Theta(max)= 24.999

R(reflections)= 0.0731(12554) wR2(reflections)= 0.2127(19509)

S = 1.016 Npar= 1467

The following ALERTS were generated. Each ALERT has the format
test-name_ALERT_alert-type_alert-level.
Click on the hyperlinks for more details of the test.

Alert level B

```
PLAT340_ALERT_3_B Low Bond Precision on  C-C Bonds ..............    0.01237 Ang.
```

Author Response: This alert is due to the poor quality and weak diffraction of the crystal. We made various attempt to grow crystals of better quality without success

Alert level C

```
STRVA01_ALERT_4_C           Flack parameter is too small
           From the CIF: _refine_ls_abs_structure_Flack   -0.500
           From the CIF: _refine_ls_abs_structure_Flack_su    1.000
PLAT089_ALERT_3_C Poor Data / Parameter Ratio (Zmax < 18) ........       7.12 Note
PLAT193_ALERT_1_C Cell and Diffraction Temperatures Differ by ....          1 Degree
PLAT234_ALERT_4_C Large Hirshfeld Difference C31      --C32      .       0.16 Ang.
PLAT234_ALERT_4_C Large Hirshfeld Difference C3B      --C4B      .       0.17 Ang.
PLAT234_ALERT_4_C Large Hirshfeld Difference C22B     --C23B     .       0.17 Ang.
PLAT601_ALERT_2_C Unit Cell Contains Solvent Accessible VOIDS of .         90 Ang**3
PLAT910_ALERT_3_C Missing # of FCF Reflection(s) Below Theta(Min).          7 Note
PLAT911_ALERT_3_C Missing FCF Refl Between Thmin & STh/L=    0.595          8 Report
PLAT913_ALERT_3_C Missing # of Very Strong Reflections in FCF ....          5 Note
```

Alert level G

```
PLAT032_ALERT_4_G Std. Uncertainty on Flack Parameter Value High .      1.000 Report
PLAT870_ALERT_4_G ALERTS Related to Twinning Effects Suppressed ..          ! Info
PLAT916_ALERT_2_G Hooft y and Flack x Parameter Values Differ by .       0.30 Check
PLAT931_ALERT_5_G CIFcalcFCF  Twin Law ( 0 0 1)        Est.d BASF       0.11 Check
PLAT933_ALERT_2_G Number of HKL-OMIT Records in Embedded .res File          8 Note
PLAT967_ALERT_5_G Note: Two-Theta Cutoff Value in Embedded .res ..       50.0 Degree
```

 0 **ALERT level A** = Most likely a serious problem - resolve or explain
 1 **ALERT level B** = A potentially serious problem, consider carefully
10 **ALERT level C** = Check. Ensure it is not caused by an omission or oversight
 6 **ALERT level G** = General information/check it is not something unexpected

```
 1 ALERT type 1 CIF construction/syntax error, inconsistent or missing data
 3 ALERT type 2 Indicator that the structure model may be wrong or deficient
 5 ALERT type 3 Indicator that the structure quality may be low
 6 ALERT type 4 Improvement, methodology, query or suggestion
 2 ALERT type 5 Informative message, check
```

It is advisable to attempt to resolve as many as possible of the alerts in all categories. Often the minor alerts point to easily fixed oversights, errors and omissions in your CIF or refinement strategy, so attention to these fine details can be worthwhile. In order to resolve some of the more serious problems it may be necessary to carry out additional measurements or structure refinements. However, the purpose of your study may justify the reported deviations and the more serious of these should normally be commented upon in the discussion or experimental section of a paper or in the "special_details" fields of the CIF. checkCIF was carefully designed to identify outliers and unusual parameters, but every test has its limitations and alerts that are not important in a particular case may appear. Conversely, the absence of alerts does not guarantee there are no aspects of the results needing attention. It is up to the individual to critically assess their own results and, if necessary, seek expert advice.

Publication of your CIF in IUCr journals

A basic structural check has been run on your CIF. These basic checks will be run on all CIFs submitted for publication in IUCr journals (*Acta Crystallographica*, *Journal of Applied Crystallography*, *Journal of Synchrotron Radiation*); however, if you intend to submit to *Acta Crystallographica Section C* or *E* or *IUCrData*, you should make sure that full publication checks are run on the final version of your CIF prior to submission.

Publication of your CIF in other journals

Please refer to the *Notes for Authors* of the relevant journal for any special instructions relating to CIF submission.

PLATON version of 06/07/2023; check.def file version of 30/06/2023

Datablock d8v20176 - ellipsoid plot

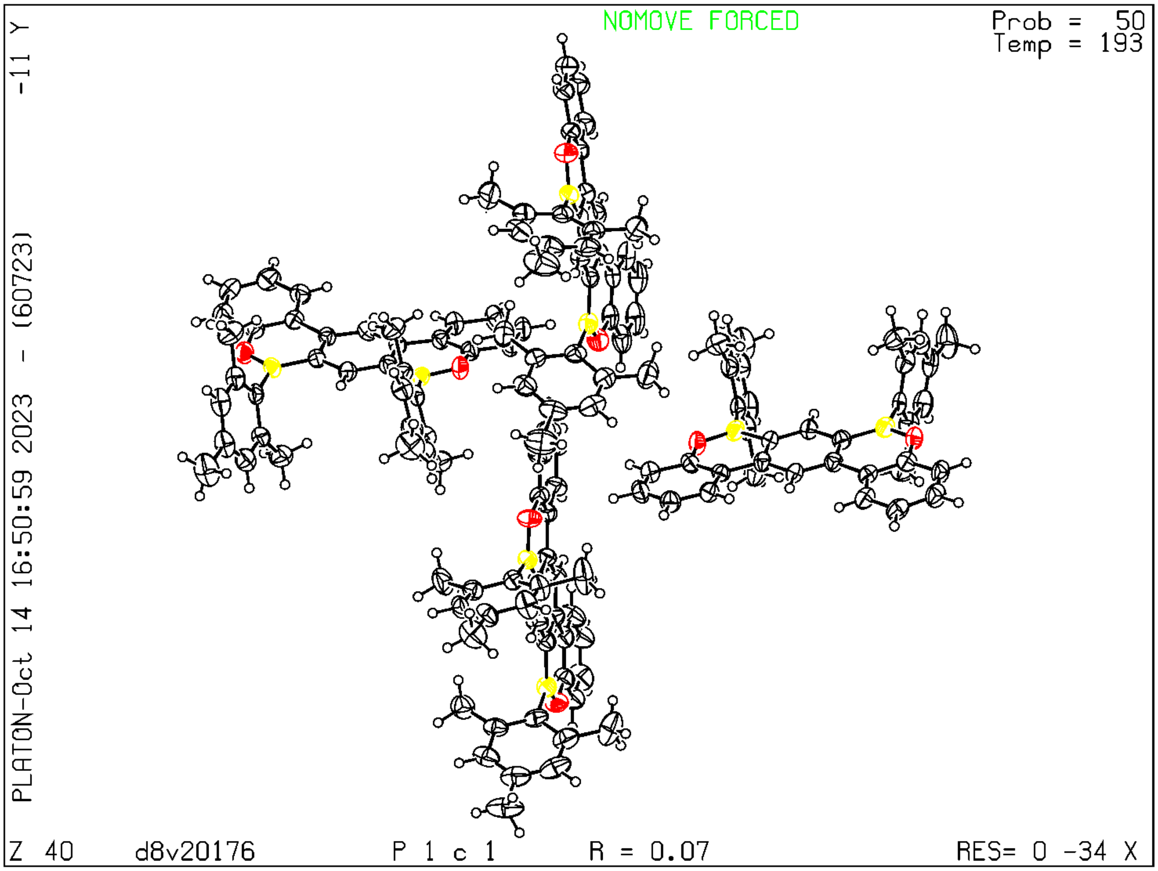

Supplement: Supplementary file 13 — Supplementary Data 10 [file 41467_2023_42973_MOESM13_ESM.pdf]
