# Supplementary material for: Double boron–oxygen-fused polycyclic aromatic hydrocarbons: skeletal editing and applications as organic optoelectronic materials
Source: Nat Commun. 2023 Nov 4;14:7089. doi: 10.1038/s41467-023-42973-1 (PMC10625603; doi:10.1038/s41467-023-42973-1)

checkCIF/PLATON report

Structure factors have been supplied for datablock(s) cu_d8v23441_0m

THIS REPORT IS FOR GUIDANCE ONLY. IF USED AS PART OF A REVIEW PROCEDURE FOR PUBLICATION, IT SHOULD NOT REPLACE THE EXPERTISE OF AN EXPERIENCED CRYSTALLOGRAPHIC REFEREE.

No syntax errors found. CIF dictionary Interpreting this report

Datablock: cu_d8v23441_0m

```
Bond precision:   C-C = 0.0043 A          Wavelength=1.54178

Cell:             a=17.5960(5)     b=7.5552(2)         c=19.2892(5)
                  alpha=90         beta=114.590(1)     gamma=90
Temperature:      293 K

                 Calculated                  Reported
Volume           2331.77(11)                 2331.77(11)
Space group      P 21/n                      P 1 21/n 1
Hall group       -P 2yn                      -P 2yn
Moiety formula   C29 H27 B O2                C29 H27 B O2
Sum formula      C29 H27 B O2                C29 H27 B O2
Mr               418.32                      418.31
Dx,g cm-3        1.192                       1.192
Z                4                           4
Mu (mm-1)        0.562                       0.562
F000             888.0                       888.0
F000'            890.42
h,k,lmax         21,9,23                     21,8,23
Nref             4139                        4083
Tmin,Tmax        0.910,0.945                 0.573,0.753
Tmin'            0.894

Correction method= # Reported T Limits: Tmin=0.573 Tmax=0.753
AbsCorr = NONE

Data completeness= 0.986           Theta(max)= 66.996

R(reflections)= 0.0718( 2977)                          wR2(reflections)=
                                                       0.2241( 4083)
S = 1.031                    Npar= 297
```

The following ALERTS were generated. Each ALERT has the format
test-name_ALERT_alert-type_alert-level.
Click on the hyperlinks for more details of the test.

Alert level B

```
PLAT420_ALERT_2_B D-H Bond Without Acceptor  O1       --H1       .     Please Check
```

Author Response: H1 is located from electron density map and DFIX command is used to fix its coordinate. There seems to be a weak interaction between O1 and the benzene ring of the neighboring molecule.

Alert level C

```
PLAT230_ALERT_2_C Hirshfeld Test Diff for    C25      --C28      .        5.3 s.u.
PLAT242_ALERT_2_C Low    'MainMol' Ueq as Compared to Neighbors of       C25 Check
PLAT340_ALERT_3_C Low Bond Precision on  C-C Bonds ...............    0.00429 Ang.
PLAT906_ALERT_3_C Large K Value in the Analysis of Variance ......      5.553 Check
PLAT911_ALERT_3_C Missing FCF Refl Between Thmin & STh/L=    0.597         52 Report
PLAT913_ALERT_3_C Missing # of Very Strong Reflections in FCF ....         18 Note
```

Alert level G

```
PLAT002_ALERT_2_G Number of Distance or Angle Restraints on AtSite          2 Note
PLAT072_ALERT_2_G SHELXL First  Parameter in WGHT  Unusually Large       0.12 Report
PLAT172_ALERT_4_G The CIF-Embedded .res File Contains DFIX Records          1 Report
PLAT199_ALERT_1_G Reported _cell_measurement_temperature ..... (K)        293 Check
PLAT200_ALERT_1_G Reported   _diffrn_ambient_temperature ..... (K)        293 Check
PLAT380_ALERT_4_G Incorrectly? Oriented X(sp2)-Methyl Moiety .....        C21 Check
PLAT860_ALERT_3_G Number of Least-Squares Restraints ............           1 Note
PLAT883_ALERT_1_G No Info/Value for _atom_sites_solution_primary .     Please Do !
PLAT909_ALERT_3_G Percentage of I>2sig(I) Data at Theta(Max) Still        47% Note
PLAT910_ALERT_3_G Missing # of FCF Reflection(s) Below Theta(Min).          1 Note
PLAT933_ALERT_2_G Number of HKL-OMIT Records in Embedded .res File          3 Note
PLAT941_ALERT_3_G Average HKL Measurement Multiplicity ..........         4.7 Low
PLAT967_ALERT_5_G Note: Two-Theta Cutoff Value in Embedded .res ..      134.0 Degree
PLAT978_ALERT_2_G Number C-C Bonds with Positive Residual Density.          3 Info
```

```
   0 ALERT level A = Most likely a serious problem - resolve or explain
   1 ALERT level B = A potentially serious problem, consider carefully
   6 ALERT level C = Check. Ensure it is not caused by an omission or oversight
  14 ALERT level G = General information/check it is not something unexpected

   3 ALERT type 1 CIF construction/syntax error, inconsistent or missing data
   7 ALERT type 2 Indicator that the structure model may be wrong or deficient
   8 ALERT type 3 Indicator that the structure quality may be low
   2 ALERT type 4 Improvement, methodology, query or suggestion
   1 ALERT type 5 Informative message, check
```

It is advisable to attempt to resolve as many as possible of the alerts in all categories. Often the minor alerts point to easily fixed oversights, errors and omissions in your CIF or refinement strategy, so attention to these fine details can be worthwhile. In order to resolve some of the more serious problems it may be necessary to carry out additional measurements or structure refinements. However, the purpose of your study may justify the reported deviations and the more serious of these should normally be commented upon in the discussion or experimental section of a paper or in the "special_details" fields of the CIF. checkCIF was carefully designed to identify outliers and unusual parameters, but every test has its limitations and alerts that are not important in a particular case may appear. Conversely, the absence of alerts does not guarantee there are no aspects of the results needing attention. It is up to the individual to critically assess their own results and, if necessary, seek expert advice.

Publication of your CIF in IUCr journals

A basic structural check has been run on your CIF. These basic checks will be run on all CIFs submitted for publication in IUCr journals (*Acta Crystallographica*, *Journal of Applied Crystallography*, *Journal of Synchrotron Radiation*); however, if you intend to submit to *Acta Crystallographica Section C* or *E* or *IUCrData*, you should make sure that full publication checks are run on the final version of your CIF prior to submission.

Publication of your CIF in other journals

Please refer to the *Notes for Authors* of the relevant journal for any special instructions relating to CIF submission.

PLATON version of 06/07/2023; check.def file version of 30/06/2023

Datablock cu_d8v23441_0m - ellipsoid plot

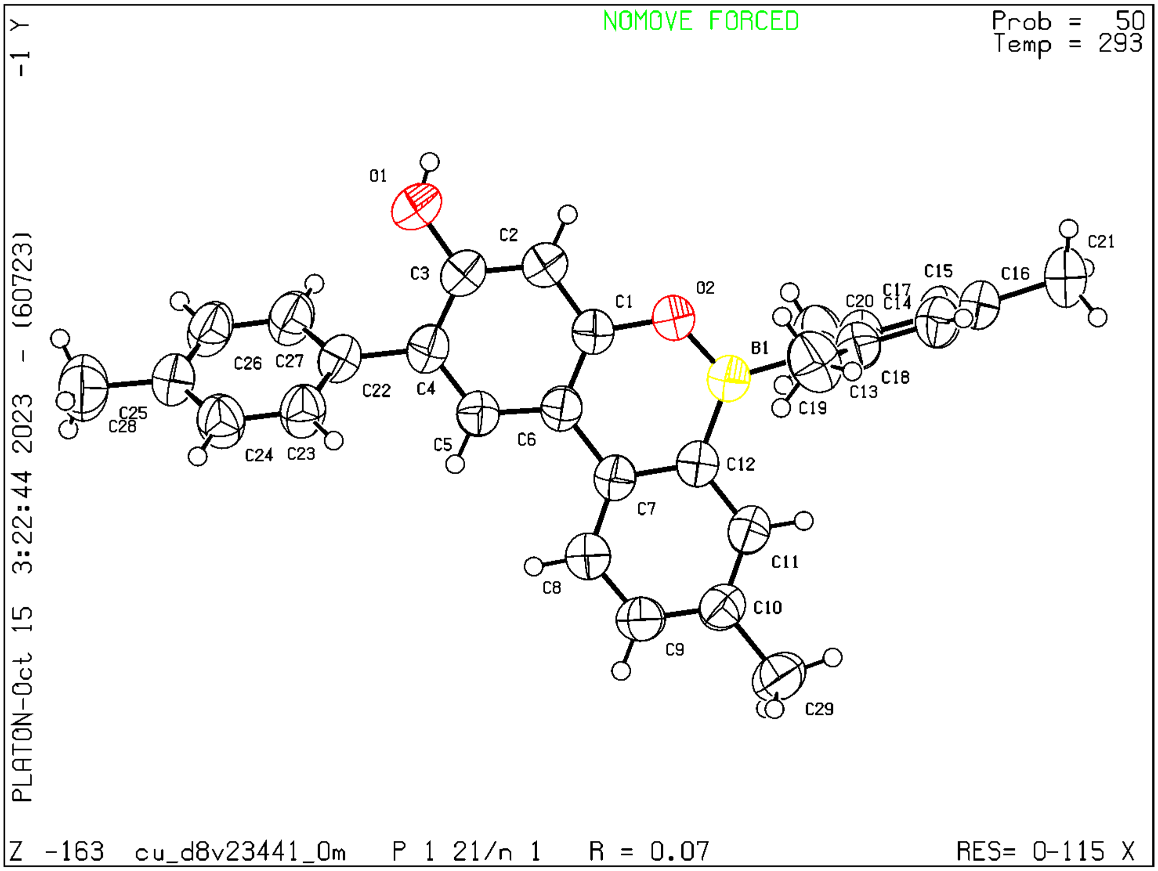

Supplement: Supplementary file 7 — Supplementary Data 4 [file 41467_2023_42973_MOESM7_ESM.pdf]
